# Whole-genome sequencing and gene network modules predict gemcitabine/carboplatin-induced myelosuppression in non-small cell lung cancer patients

**DOI:** 10.1038/s41540-020-00146-6

**Published:** 2020-08-24

**Authors:** Niclas Björn, Tejaswi Venkata Satya Badam, Rapolas Spalinskas, Eva Brandén, Hirsh Koyi, Rolf Lewensohn, Luigi De Petris, Zelmina Lubovac-Pilav, Pelin Sahlén, Joakim Lundeberg, Mika Gustafsson, Henrik Gréen

**Affiliations:** 1 Clinical Pharmacology, Division of Drug Research, Department of Biomedical and Clinical Sciences, Linköping University, Linköping, Sweden; 2 Bioinformatics, Department of Physics, Chemistry and Biology, Linköping University, Linköping, Sweden; 3 School of Bioscience, Systems Biology Research Centre, University of Skövde, Skövde, Sweden; 4 Science for Life Laboratory, School of Engineering Sciences in Chemistry, Biotechnology and Health, Department of Gene Technology, KTH Royal Institute of Technology, Solna, Sweden; 5 Department of Respiratory Medicine, Gävle Hospital, Gävle, Sweden; 6 Centre for Research and Development, Uppsala University/Region Gävleborg, Gävle, Sweden; 7 Thoracic Oncology Unit, Tema Cancer, Karolinska University Hospital, and Department of Oncology-Pathology, Karolinska Institutet, Stockholm, Sweden; 8 Department of Forensic Genetics and Forensic Toxicology, National Board of Forensic Medicine, Linköping, Sweden

**Keywords:** Genetic interaction, Cancer, Genetic interaction, Systems analysis

## Abstract

Gemcitabine/carboplatin chemotherapy commonly induces myelosuppression, including neutropenia, leukopenia, and thrombocytopenia. Predicting patients at risk of these adverse drug reactions (ADRs) and adjusting treatments accordingly is a long-term goal of personalized medicine. This study used whole-genome sequencing (WGS) of blood samples from 96 gemcitabine/carboplatin-treated non-small cell lung cancer (NSCLC) patients and gene network modules for predicting myelosuppression. Association of genetic variants in PLINK found 4594, 5019, and 5066 autosomal SNVs/INDELs with *p* ≤ 1 × 10^−3^ for neutropenia, leukopenia, and thrombocytopenia, respectively. Based on the SNVs/INDELs we identified the toxicity module, consisting of 215 unique overlapping genes inferred from MCODE-generated gene network modules of 350, 345, and 313 genes, respectively. These module genes showed enrichment for differentially expressed genes in rat bone marrow, human bone marrow, and human cell lines exposed to carboplatin and gemcitabine (*p* < 0.05). Then using 80% of the patients as training data, random LASSO reduced the number of SNVs/INDELs in the toxicity module into a feasible prediction model consisting of 62 SNVs/INDELs that accurately predict both the training and the test (remaining 20%) data with high (CTCAE 3–4) and low (CTCAE 0–1) maximal myelosuppressive toxicity completely, with the receiver-operating characteristic (ROC) area under the curve (AUC) of 100%. The present study shows how WGS, gene network modules, and random LASSO can be used to develop a feasible and tested model for predicting myelosuppressive toxicity. Although the proposed model predicts myelosuppression in this study, further evaluation in other studies is required to determine its reproducibility, usability, and clinical effect.

## Introduction

Lung cancer is a common and deadly form of cancer. It represents close to a fifth (18.4%) of all cancer deaths worldwide^1^. The primary treatment of non-small cell lung cancer (NSCLC) includes the use of PD-1 inhibitors or targeted therapies. However, depending on their success, the continuation of the treatment using a classical combination chemotherapy consisting of gemcitabine and carboplatin is common. It is well known that the use of classical chemotherapeutic agents is associated with the induction of considerable adverse drug reactions (ADRs). This is also the case for gemcitabine/carboplatin treatment, which commonly induces severe myelosuppression (mainly expressed in the form of neutropenia, leukopenia, and thrombocytopenia) that may lead to non-optimal treatments in terms of postponements, reduction, or discontinuation^2–6^. Severe myelosuppression of Common Terminology Criteria for Adverse Events (CTCAE) grade 3–4 is roughly experienced by 50% of treated patients, while many other patients exhibit no or mild symptoms. The underlying germline genetic variation is thought to be a contributing factor to the vast inter-individual differences in ADRs^5–9^.

Being able to predict patients at risk of ADRs using genetic biomarkers and adjust doses and treatments accordingly before the start of treatment would likely be beneficial for both patient well-being and response to treatment^9^. Many studies preceding this one have investigated chemotherapy-induced myelosuppression with the long-term goal of predicting patients at risk of severe toxicity. These studies include candidate gene studies, genome-wide association studies (GWASs), and exome sequencing studies^5,6,10–14^. Although these studies have found various genetic biomarkers that have shown some predictive power, they have to date had low clinical impact and have been hard to replicate.

In the present study, we expanded the use of genetic information further by whole-genome sequencing (WGS) germline blood sample DNA from 96 NSCLC patients treated with gemcitabine/carboplatin. Transitioning to WGS not only allows us to utilize the full genome, it is also suitable for high-quality clinical sequencing approaches with more reliable genotype calls, and it is now becoming more available at decreasing sequencing prices^15–17^. Further, in this study, we applied graph-theoretic clustering algorithms, such as molecular complex detection (MCODE)^18^ for module inference and the random least absolute shrinkage and selection operator (LASSO) for the reduction and selection of genetic variants^19^. Module-based and network-based omic analyses as reviewed by Gustafsson et al.^20^ have previously shown important roles for further understanding, for example, of allergy^21^, asthma^22^, and multiple sclerosis^23^, where thousands of genes and their interactions are affected and involved. The involvement of multiple genes with complex interactions is likely also a contributing factor to the vast inter-individual differences seen in the commonly induced ADRs for patients undergoing chemotherapy. To find these, we combined WGS, gene network modules, and the random LASSO to predict high (CTCAE 3–4) and low (CTCAE 0–1) myelosuppressive toxicity in gemcitabine/carboplatin-treated NSCLC patients.

## Results

### Patient characteristics, toxicity, and WGS

The characteristics of the 96 patients selected based on their toxicity are listed in Table 1. The patient toxicity level categorized using the CTCAE scale, for neutropenia, leukopenia, thrombocytopenia, and the maximal toxicity are listed in Table 2.

**Table 1:** Patient baseline characteristics.

	All patients (*n* = 96)		Maximal myelosuppressive toxicity					
			High toxicity (*n* = 54)		Intermediate (*n* = 8)		Low toxicity (*n* = 34)	
*Gender, N (%)*								
Male	47	(49.0%)	26	(48.1%)	3	(37.5%)	18	(52.9%)
Female	49	(51.0%)	28	(51.9%)	5	(62.5%)	16	(47.1%)
*Age, in years, median (range)*								
All	65.5	(47–82)	67	(51–82)	64	(47–76)	63	(48–76)
Male	68	(51–82)	69	(57–82)	64	(56–74)	61	(51–76)
Female	63	(47–80)	64	(51–80)	64	(47–76)	63	(48–76)
*Clinical stage, N (%)*								
Stage I	17	(17.7%)	12	(22.2%)	2	(25.0%)	3	(8.8%)
Stage II	11	(11.5%)	7	(13.0%)	–	–	4	(11.8%)
Stage III	34	(35.4%)	15	(27.8%)	2	(25.0%)	17	(50.0%)
Stage IV	32	(33.3%)	18	(33.3%)	4	(50.0%)	10	(29.4%)
Not specified	2	(2.1%)	2	(3.7%)	–	–	–	–
*Histological classifications, N (%)*								
Adenocarcinoma (AC)	58	(60.4%)	34	(63.0%)	5	(62.5%)	19	(55.9%)
Squamous cell carcinomas (SCC)	19	(19.8%)	10	(18.5%)	1	(12.5%)	8	(23.5%)
Non-small cell lung cancer (NSCLC)	13	(13.5%)	8	(14.8%)	–	–	5	(14.7%)
Large cell carcinoma (LLC)	6	(6.3%)	2	(3.7%)	2	(25.0%)	2	(5.9%)
*Smoking history, N (%)*								
Current	40	(41.7%)	18	(33.3%)	4	(50.0%)	18	(52.9%)
Former	46	(47.9%)	30	(55.6%)	4	(50.0%)	12	(35.3%)
Never	10	(10.4%)	6	(11.1%)	–	–	4	(11.8%)

**Table 2:** First cycle myelosuppressive toxicity graded according to the Common Terminology Criteria for Adverse Events (CTCAE) version 4.03.

CTCAE grade	Neutropenia		Leukocytopenia		Thrombocytopenia		Maximal toxicity	
0	44	45.8%	39	40.6%	27	28.1%	23	24.0%
1	0	0.0%	7	7.3%	12	12.5%	11	11.5%
2	6	6.3%	22	22.9%	14	14.6%	8	8.3%
3	18	18.8%	24	25.0%	23	24.0%	15	15.6%
4	28	29.2%	4	4.2%	20	20.8%	39	40.6%

The WGS of the 96 samples passed the internal quality control setup at the sequencing facility of Science for Life Laboratory (SciLifeLab, Stockholm, Sweden). The sequencing outputted, on average, 722 million reads/sample with the average median insert size of 341 base pairs. On average, 99.37% of the reads were aligned, and the average coverage was 34×. Further, 63% of the reference genome was covered with ≥30×, and the average GC-content was 41%. The raw VCF file included a total of 17,934,566 single-nucleotide variants (SNVs) and insertions/deletions (INDELs), after filtering 15,751,023 bi-allelic loci remained on chromosomes 1–22, X, and Y.

### SNV/INDEL association analysis

**Fig. 1:**
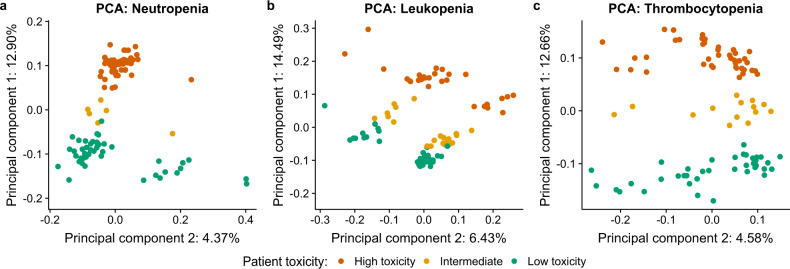
Principal component analysis (PCA). PCA using all nominally significant (*p* ≤ 1 × 10^−3^) SNVs/INDELs for **a** neutropenia, **b** leukopenia, and **c** thrombocytopenia. Plotting principal component 1 against principal component 2 shows that these genetic variants can separate the patients into clusters of high (red) and low (green) toxicity with the intermediates (yellow) in-between.

Fisher’s exact test identified 4594 (5743), 5019 (6063), and 5066 (5959) autosomal (total numbers in parentheses) nominally significant (*p* ≤ 1 × 10^−3^) genetic variants (SNVs/INDELs) for neutropenia, leukopenia, and thrombocytopenia, respectively. All these genetic variants are listed in Supplementary Tables 1–3. There was some overlap between the genetic variants, as visualized in Supplementary Fig. 1. PCA clearly showed that the respective nominally significant autosomal germline genetic variants have the potential for stratifying patients into high (CTCAE 3–4), intermediate (CTCAE 2), or low (CTCAE 0–1) toxicity for neutropenia (Fig. 1a), leukopenia (Fig. 1b), and thrombocytopenia (Fig. 1c). This was expected as the genetic variants used were selected based on their association (*p* ≤ 1 × 10^−3^) with toxicity (as determined by Fisher’s exact test). Interestingly, the intermediates not included in the statistical tests ended up in between, separated from both low and high toxicity samples. Further, when using all SNVs/INDELs for PCA, no apparent clustering based on toxicity was seen (Supplementary Fig. 2). Genome annotation enrichment analysis shows that most SNVs/INDELs are distal intergenic variants, and a slight enrichment of the proportion of distal intergenic variants for the nominally significant genetic variants compared to the background was found (Supplementary Fig. 3). Stronger association (*p* ≤ 1 × 10^−5^) was found for only 55, 107, and 149 genetic variants for neutropenia, leukopenia, and thrombocytopenia, respectively. We therefore concluded that the WGS data needed to be combined with other statistical testing to increase the power. We proceeded to gene network analysis in order to prioritize functionally relevant gene sets to the three toxicity phenotypes.

### Gene network modules

**Fig. 2:**
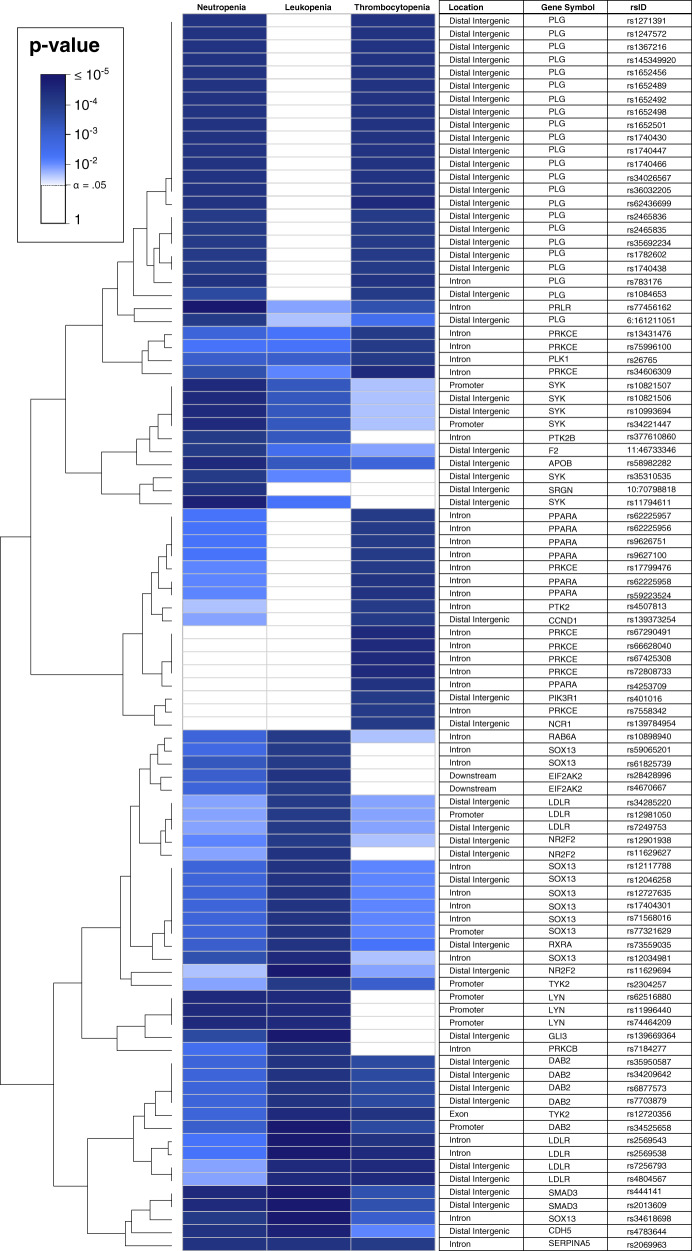
SNVs/INDELs and seed genes in the toxicity module. Heatmap showing the 95 nominally significant (*p* ≤ 1 × 10^−3^) genetic variants (SNVs/INDELs) that mapped to the seed genes in the toxicity module. It also shows the overlap of the seed variants from neutropenia, leukopenia, and thrombocytopenia along with their annotated location and the gene they mapped to.

**Fig. 3:**
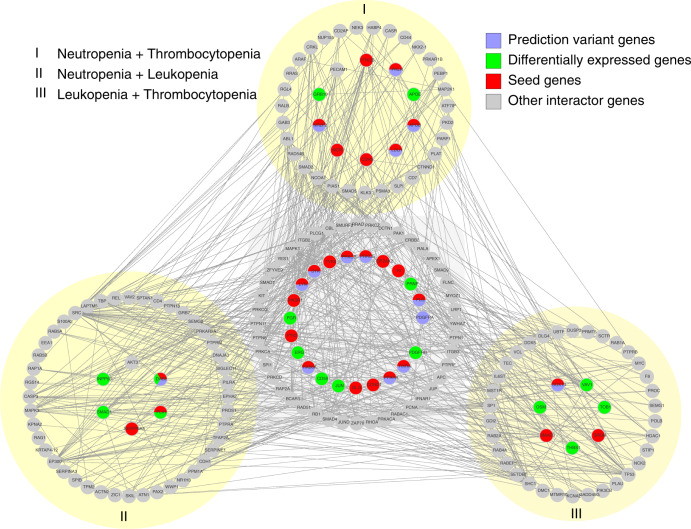
Gene network module. The entire gene network module for the toxicity module consisting of all 215 genes. The middle part shows the genes shared by all three toxicities, while the three outer parts on yellow-shaded backgrounds show the genes shared by two of the toxicities, (I) for neutropenia and thrombocytopenia, (II) for neutropenia and leukopenia, and (III) for leukopenia and thrombocytopenia. Further, the colors show if the genes include predictor variants (blue), are differentially expressed (green), are seeds (red), or if they are other interactor genes in the gene network module (gray).

The nominally significant SNVs/INDELs for the three toxicity phenotypes were mapped to 896, 937, and 999 protein-coding genes for neutropenia, leukopenia, and thrombocytopenia, respectively. This was performed to understand the long-range interactions across the entire genome, and they are referred to as seed genes. After this, modules for each toxicity were constructed using MCODE together with the String PPI network^24^, whereby gene modules of size 350 (24 seed genes), 345 (21), and 313 (14) were identified for neutropenia, leukopenia, and thrombocytopenia, respectively. All MCODE module genes are listed in Supplementary Table 4. We also tested other relevant standard methods for module construction, such as DIAMOnD^25^, CliqueSuM^26^, and ModuleDiscoverer^27^. These modules yielded consistently lower enrichment in our downstream analyses presented below. Interestingly, 215 of the MCODE modules genes were shared across at least two of the modules (Supplementary Fig. 4), which hereafter is referred to as the toxicity module. The 95 genetic variants used as seeds are shown in Fig. 2, and the complete gene network module is visualized in Fig. 3. We next proceeded with functional enrichment analysis of the different modules and seed genes using independent gene expression data.

### Functional enrichment: gemcitabine/carboplatin-treated bone marrow from rats and humans

**Fig. 4:**
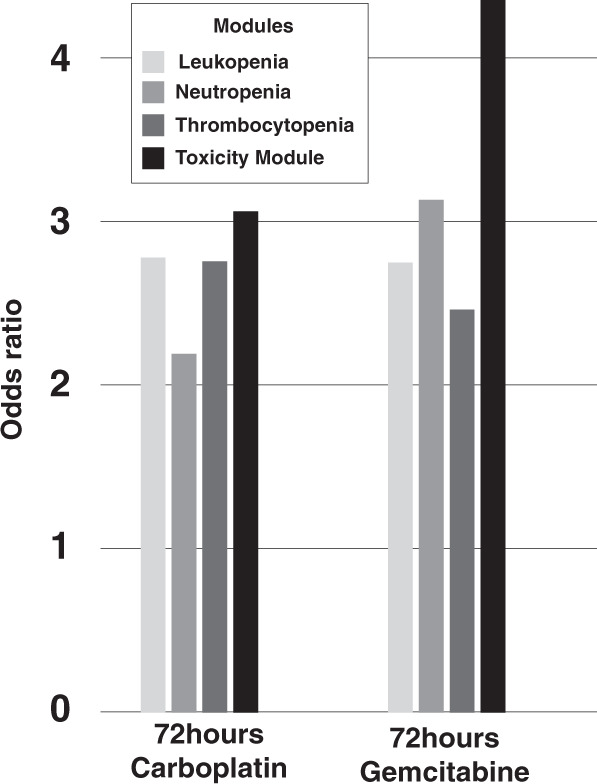
Module genes and rat bone marrow data. Odds ratios (ORs) of module genes overlap with gemcitabine-treated and carboplatin-treated rat bone marrow gene expression data.

To statistically validate the relevance of the different modules, based on human WGS data, we first performed enrichment analysis using genes differentially expressed upon stimulation specifically from gemcitabine and carboplatin. For this purpose, we used homologous genes from rat bone marrow data (GSE59894) that included 208 carboplatin and 673 gemcitabine differentially expressed genes upon 72 hours of exposure. Enrichment analysis showed that the toxicity module showed the highest enrichment for both gemcitabine (Fisher’s exact test *p* = 3.9 × 10^−9^, odds ratio (OR) = 4.4) and carboplatin (*p* = 0.02, OR = 3.1) (see Fig. 4). This enrichment was consistently higher than all other modules and the seed gene lists independently. The full comparison is available in Supplementary Table 5. We also found significant overlaps for carboplatin (*p* = 2.0 × 10^−3^, OR = 5.3, *n* = 5) and gemcitabine (*p* = 1.0 × 10^−3^, OR = 5.9, *n* = 5) with the human bone marrow and kidney meta-analysis gene expression data. This ensures that the module is effectively translated back to a human level. However, to strengthen and increase the resolution of this finding we also performed a human cell line RNA-seq study.

### Functional enrichment: RNA-seq of gemcitabine/carboplatin-treated human cell lines

The RNA-seq yielded, on average, 26 million reads/sample, of which, on average, 17 million reads (65%) mapped uniquely. From this, featureCounts uniquely summarized, on average, 15 million reads to gene regions for each sample. Of the 215 genes in the toxicity module, 152 were found to be expressed in the cell lines (TPM > 1 in ≥2 samples) listed in Supplementary Table 6. This overlap was significantly greater than expected by chance, as proven by both Fisher’s exact test (OR = 1.6, *p* = 4.2 × 10^−15^, *n* = 152) and 10,000 permutations found, on average, 95.5 genes expressed at the same level (Supplementary Fig. 5). Of the 152 expressed genes, 17 were module seed genes.

**Fig. 5:**
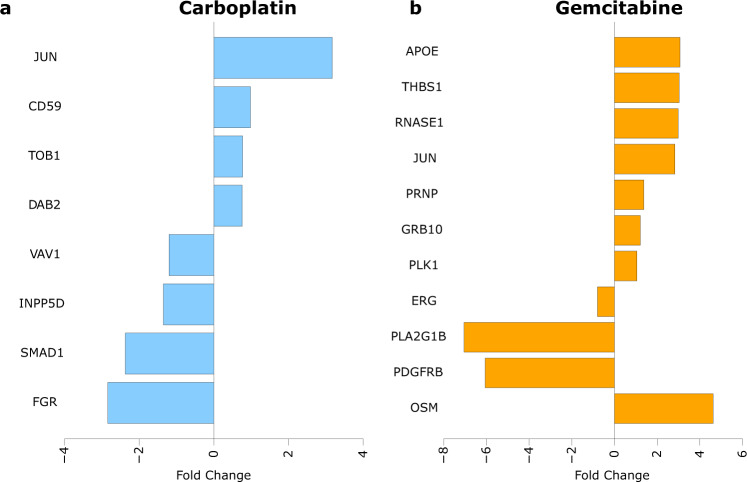
Differentially expressed genes. Shows the fold change (log_2_) of the differentially expressed genes from the toxicity module in the cell lines after treatment with **a** carboplatin and **b** gemcitabine.

Further, differential expression analysis showed that, compared to the respective untreated cell lines, some module gene expressions were altered (*p* ≤ 0.05). In total, 18 genes from the toxicity module were differentially expressed, as visualized in Fig. 5a, b for carboplatin and gemcitabine, respectively. The differentially expressed genes are also listed in Supplementary Table 7. Two of the differentially expressed genes, *DAB2* and *PLK1*, were module seed genes. Interestingly, carboplatin mainly affected the expression of genes in K562; in contrast, gemcitabine mainly affected the expression of genes in MOLM-1.

### Functional enrichment: KEGG pathway and GO enrichment

**Fig. 6:**
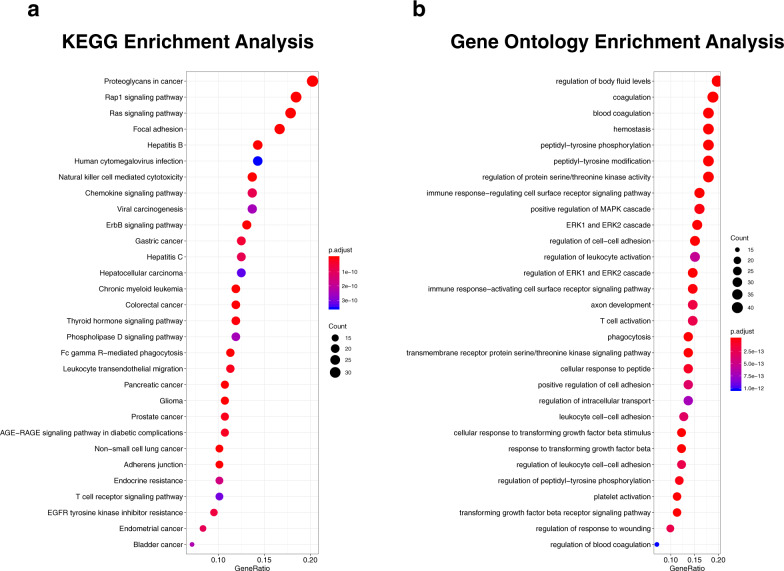
Enrichment analysis. Overview of the top 30 most enriched **a** KEGG pathways and **b** gene ontologies.

The top 30 most enriched KEGG pathways (FDR adjusted *p*-values ≤ 3.55 × 10^−10^) and GOs (FDR adjusted *p*-values ≤ 1.02 × 10^−12^) are shown in Fig. 6a, b, respectively, and are listed in Supplementary Table 8. The top KEGG pathways were mainly cancer-related, where the pathways “*Non-small cell lung cancer*” and “*Chronic myeloid leukemia*” stuck out as the first is related to the disease of the patients in the present study, and the second possibly share many genes that might be of importance for the development of myelosuppressive toxicities and malignancies. Of the GO terms found, several were related to the myelosuppressive toxicities investigated, for example, “*hemostasis*”, “*regulation of leukocyte activation*”, “*leukocyte cell–cell adhesion*”, “*blood coagulation*”, and “*platelet activation*”.

### Toxicity prediction models

**Fig. 7:**
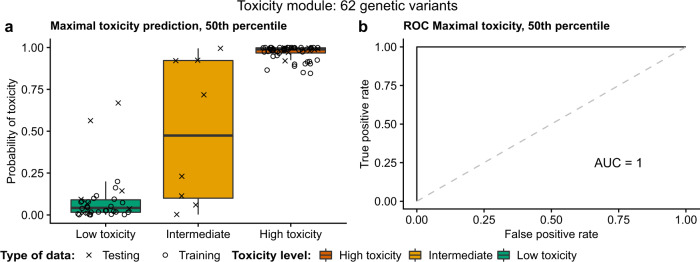
Toxicity module prediction model. Shows the best maximal toxicity prediction model based on the toxicity module. It consists of 62 genetic variants (the 50th percentile most used variants in the random LASSO permutations). **a** Patients (separated by registered maximal toxicity) and their predicted probability of maximal toxicity. **b** ROC curve of the model’s predictions of high and low maximal toxicity. Note that the intermediates were not used for calculating the ROC. The box-plot elements should be interpreted as the following: centerline, median; box limits, upper and lower quartiles; whiskers, 1.5× interquartile range.

Lastly, we aimed to test the capability of the toxicity module to separate and predict the toxicity. For this purpose, we started from the 123 nominally significant genetic variants that mapped to genes in the toxicity module. We utilized random LASSO permutation (*n* = 100,000) analyses to reduce the number of genetic variants into a smaller set that would still predict the maximal toxicity experienced by patients. After the permutations, sets of genetic variants, based on quantiles of the number of times the genetic variants were randomly selected by the LASSO, were evaluated further by running LASSOs without shrinkage to determine the genetic variants’ final coefficients for predictions. By checking the ROC and AUC, we constructed a model that can predict both the training and the test data perfectly, with a ROC AUC = 1.0. The prediction model is based on 62 genetic variants, the 50th percentile most selected genetic variants, listed in Table 3, from the toxicity module. The maximal toxicity predictions of this model are shown in Fig. 7a, and the ROCs in Fig. 7b. The eight intermediate samples (were only used for predictions not for training or testing) are shown in Fig. 7a to have both high and low toxicity characteristics. By applying the model, 3–4 intermediate patients would be predicted to have a high and 4–5 intermediate patients would be predicted to have a low probability of toxicity, although the mean intermediate probability of toxicity ends up in between at roughly 50%. Two of the low toxicity test samples were predicted to have a quite high probability of toxicity using the toxicity module, however, they remain classified as low toxicity samples as they are separable from the red high toxicity cluster in the right-hand upper corner of Fig. 7a. Further, by applying the genetic variants from the toxicity module maximal toxicity prediction models to neutropenia, leukopenia, and thrombocytopenia, Supplementary Fig. 6 shows that these models are fairly good in determining the specific high and low toxicities, however, not as accurate as the prediction of maximal toxicity. Supplementary Table 9 lists all genetic variants and their respective prediction model coefficients used for the prediction models visualized in Fig. 7 and Supplementary Fig. 6. This test shows that the identified module is both functionally and statistically sound and therefore a good candidate for clinical testing.

**Table 3:** Information on the 62 genetic variants included in the final maximal toxicity prediction model based on the toxicity module.

rsID	Annotation	Chromosome	Gene Name	Neutropenia *p*-value	Leukopenia *p*-value	Thrombocytopenia *p*-value	Neutropenia seed	Leukopenia seed	Thrombocytopenia seed
rs10898940	Intron	11	RAB6A	6.95E−03	6.81E−04	4.12E−02	False	True	False
rs11629627	Distal intergenic	15	NR2F2	3.37E−02	5.00E−04	6.11E−02	False	True	False
rs11629694	Distal intergenic	15	NR2F2	3.89E−02	8.62E−05	2.97E−02	False	True	False
rs11794611	Distal intergenic	9	SYK	1.66E−04	1.56E−02	1.70E−01	True	False	False
rs11996440	Promoter	8	LYN	3.13E−04	2.68E−04	3.77E−01	True	True	False
rs12034981	Intron	1	SOX13	2.18E−03	3.72E−04	4.10E−02	True	True	False
rs12046258	Distal intergenic	1	SOX13	6.90E−03	5.90E−04	2.35E−02	True	True	False
rs12117788	Intron	1	SOX13	6.90E−03	5.90E−04	2.35E−02	True	True	False
rs1247572	Distal intergenic	6	PLG	4.27E−04	7.78E−02	4.67E−04	True	False	True
rs12981050	Promoter	19	LDLR	3.49E−02	8.68E−04	3.59E−02	False	True	True
rs13431476	Intron	2	PRKCE	8.77E−03	1.82E−02	7.89E−04	False	False	True
rs1367216	Distal intergenic	6	PLG	4.27E−04	7.78E−02	4.67E−04	True	False	True
rs1498830	Intron	4	PDGFRA	1.02E−03	3.57E−03	1.46E−04	False	False	False
rs1652456	Distal intergenic	6	PLG	4.27E−04	7.78E−02	4.67E−04	True	False	True
rs1740430	Distal intergenic	6	PLG	4.27E−04	7.78E−02	4.67E−04	True	False	True
rs17404301	Intron	1	SOX13	6.90E−03	5.90E−04	2.35E−02	True	True	False
rs2465836	Distal intergenic	6	PLG	8.12E−04	2.12E−01	8.96E−04	True	False	True
rs2569538	Intron	19	LDLR	1.84E−02	1.03E−04	3.75E−04	False	True	True
rs2569543	Intron	19	LDLR	1.80E−02	8.23E−05	4.09E−04	False	True	True
rs2590763	Intron	4	PDGFRA	1.75E−03	3.89E−03	2.85E−04	False	False	False
rs2590807	Intron	4	PDGFRA	3.47E−04	2.00E−03	4.09E−05	False	False	False
rs2590827	Intron	4	PDGFRA	3.04E−03	1.17E−02	5.88E−04	False	False	False
rs2590829	Intron	4	PDGFRA	1.66E−03	1.10E−02	9.66E−04	False	False	False
rs2616405	Intron	4	PDGFRA	3.04E−03	1.17E−02	5.88E−04	False	False	False
rs2616431	Intron	4	PDGFRA	6.02E−04	3.57E−03	7.82E−05	False	False	False
rs2616433	Intron	4	PDGFRA	6.02E−04	3.57E−03	1.46E−04	False	False	False
rs34285220	Distal intergenic	19	LDLR	3.49E−02	8.68E−04	3.59E−02	False	True	True
rs4864488	Intron	4	PDGFRA	1.02E−03	3.57E−03	1.46E−04	False	False	False
rs4864820	Intron	4	PDGFRA	1.02E−03	3.57E−03	1.46E−04	False	False	False
rs531650	Intron	1	PRRX1	3.17E−04	2.50E−04	4.04E−01	True	True	False
rs5858236	Intron	4	PDGFRA	9.85E−04	9.53E−03	1.36E−04	False	False	False
rs5858241	Intron	4	PDGFRA	1.02E−03	3.57E−03	1.46E−04	False	False	False
rs58982282	Intron	2	APOB	3.68E−04	3.06E−03	8.61E−03	True	False	False
rs59065201	Intron	1	SOX13	1.00E−02	8.20E−04	5.47E−02	True	True	False
rs59481386	Intron	4	PDGFRA	3.93E−03	8.64E−03	7.33E−04	False	False	False
rs61825739	Intron	1	SOX13	3.84E−03	8.51E−04	6.47E−02	True	True	False
rs62225956	Intron	22	PPARA	1.63E−02	1.34E−01	9.64E−04	False	False	True
rs62225957	Intron	22	PPARA	1.63E−02	1.34E−01	9.64E−04	False	False	True
rs62225958	Intron	22	PPARA	2.58E−02	1.32E−01	4.29E−04	False	False	True
rs62436699	Distal intergenic	6	PLG	4.01E−04	5.80E−02	3.56E−04	True	False	True
rs66628040	Intron	2	PRKCE	6.69E−02	1.47E−01	3.63E−04	False	False	True
rs67425308	Intron	2	PRKCE	6.69E−02	1.47E−01	3.63E−04	False	False	True
rs6815433	Intron	4	PDGFRA	1.77E−03	6.43E−03	5.22E−04	False	False	False
rs6826915	Intron	4	PDGFRA	3.47E−04	2.00E−03	4.09E−05	False	False	False
rs6828755	Intron	4	PDGFRA	1.02E−03	3.57E−03	1.46E−04	False	False	False
rs6842780	Intron	4	PDGFRA	3.93E−03	8.64E−03	7.33E−04	False	False	False
rs6877573	Distal intergenic	5	DAB2	8.41E−03	5.56E−04	1.40E−03	False	True	False
rs7184277	Intron	16	PRKCB	1.22E−02	5.29E−04	1.00E+00	False	True	False
rs7249753	Distal intergenic	19	LDLR	3.49E−02	8.68E−04	3.59E−02	False	True	True
rs7256793	Distal intergenic	19	LDLR	3.30E−02	3.02E−04	3.75E−04	False	True	True
rs72808733	Intron	2	PRKCE	6.69E−02	1.47E−01	3.63E−04	False	False	True
rs73559035	Distal intergenic	9	RXRA	5.18E−03	4.40E−04	1.76E−02	False	True	False
rs7378471	Intron	4	PDGFRA	1.64E−03	2.07E−03	2.49E−04	False	False	False
rs74464209	Promoter	8	LYN	3.13E−04	2.68E−04	3.77E−01	True	True	False
rs7703879	Distal intergenic	5	DAB2	8.41E−03	5.56E−04	1.40E−03	False	True	False
rs77321629	Promoter	1	SOX13	6.90E−03	5.90E−04	2.35E−02	True	True	False
rs77456162	Intron	5	PRLR	1.24E−04	3.08E−02	1.65E−03	True	False	False
rs783176	Intron	6	PLG	4.27E−04	2.12E−01	4.67E−04	True	False	True
rs904414	Intron	4	PDGFRA	7.69E−03	2.69E−02	5.88E−04	False	False	False
rs9626751	Intron	22	PPARA	1.63E−02	1.34E−01	9.64E−04	False	False	True
rs9627100	Intron	22	PPARA	1.63E−02	1.34E−01	9.64E−04	False	False	True
rs9759545	Intron	4	PDGFRA	3.04E−03	6.84E−03	5.88E−04	False	False	False

### Additional validation analysis

**Fig. 8:**
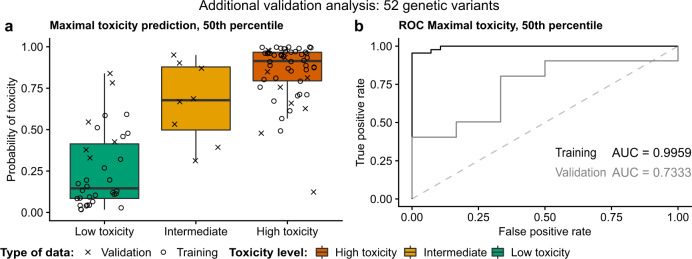
Additional prediction model. Shows the best maximal toxicity prediction model based on the approach starting from only 80% of the patient samples using the remaining 20% of samples for validating the prediction model. It consists of 52 genetic variants (the 50th percentile most used variants in the random LASSO permutations). **a** Patients (separated by registered maximal toxicity) and their predicted probability of maximal toxicity. **b** ROC curves of the model’s predictions of high and low maximal toxicity for the training samples in black and testing samples in dark gray. Note that the intermediates were not used for calculating the ROC. The box-plot elements should be interpreted as the following: centerline, median; box limits, upper and lower quartiles; whiskers, 1.5× interquartile range.

Using 80% of the samples for Fisher’s exact test yielded 4359, 5328, and 4467 autosomal nominally significant genetic variants mapping to 879, 821, and 846 protein-coding genes for neutropenia, leukopenia, and thrombocytopenia, respectively. Subsequently, these genes were used to identify gene modules of size 316, 322, and 321 for neutropenia, leukopenia, and thrombocytopenia, respectively. Here we used the more stringent criteria that genes had to overlap all three toxicities leading to a final set of 108 genes. We then used the 104 nominally significant genetic variants mapping to these 108 genes for the random LASSO permutations, which showed that the 50th percentile of the most selected genetic variants yielded the best predictions. Figure 8 shows that this prediction model using 52 genetic variants predicts the training samples with a ROC AUC of 99.6% and the validation samples with a ROC AUC of 73.3%.

## Discussion

Patients undergoing treatment that includes gemcitabine/carboplatin commonly experience myelosuppressive ADRs. These severe toxicities are dose-limiting, often rendering the treatment to be non-optimal. Even though the treatment is currently adjusted to body surface area and renal function, there still is significant variation in the toxicity experienced by patients. Being able to predict patients at risk of severe toxicity and adjusting treatments accordingly would likely improve both patient well-being and response to the treatment. This is a major cornerstone needed for personalized medicine. For this purpose, we whole-genome sequenced 96 NSCLC patients homogeneously treated with gemcitabine/carboplatin. The cohort was carefully selected and monitored closely in a controlled manner according to the treatment protocols used at the time of inclusion. The study focused on finding new means for predicting the risk of myelosuppressive toxicities using germline genetics in models that can be used for implementing personalized medicine and predicting toxicity in the future.

The initial association of SNVs/INDELs using Fisher’s exact test identified 4500–6000 nominally significant (*p* ≤ 1 × 10^−3^) genetic variants, depending on the toxicity phenotype. Using all these genetic variants for predicting toxicity is not easily implementable at a clinical level as it requires considerable genotyping and computational infrastructure. There is a need for smaller prediction models that use a reduced number of genetic parameters while still predicting toxicity. A complex phenotype, such as toxicity, can be an interplay of multiple genetic parameters rather than a consequence of an abnormality in only one gene or SNV/INDEL. However, all the nominally significant genetic variants are reported along with their individual *p*-values in Supplementary Tables 1–3, because they could be important and of interest to the research community.

The nominally significant SNVs/INDELs were mapped to their nearest protein-coding gene to obtain a functionally annotated framework for identifying the highly interacting components of the genetic variants underlying myelosuppressive toxicity. The *p*-values obtained from the WGS were attributed as the mapped protein-coding gene’s weight input on the interactome constructed from the String PPI version 10.5^24^. We initially tested four different gene network module algorithms: MCODE^18^, DIAMOnD^25^, CliqueSuM^26^, and ModuleDiscoverer^27^. Interestingly, we found that modules constructed using MCODE had stronger enrichment for genes affected by carboplatin and gemcitabine exposure (Supplementary Table 5). Though there are several algorithms available for identifying gene network modules, MCODE^18^ performed best in the presented study in terms of significant functional enrichments, because it is solely based on the connectivity topology in the network and is not affected by false positives in high-throughput sequencing data. This reduced the number of mapped genes for the prediction model considerably, from roughly a 1000 to 300 for each toxicity phenotype. Using gene network modules as biomarkers have shown promising results^20–23^, but they have previously not been used for chemotherapy-induced toxicity. We propose the gene network module called the toxicity module for the prediction of maximal myelosuppressive toxicity.

For demonstrating that the toxicity module is hypothetically built up by functional elements likely affected by gemcitabine/carboplatin, we performed several independent enrichment analyses using differential expression of the toxicity module genes. This confirmed that the toxicity module was enriched for functional elements consisting of both human and rat–human homolog genes targeted specifically by carboplatin and gemcitabine. The RNA-seq analysis of human myelogenous cell lines showed that 70% of the toxicity module genes were expressed, of which 18 were differentially expressed after exposure to gemcitabine and carboplatin in line with the results stated above. Next, the analyses of KEGG pathways and GOs provided more support for the toxicity module genes relevance through the significant enrichment of both cancer-related KEGG pathways and hemostasis, platelet, and leukocyte-related GOs. The enrichment of hemostasis, platelet, and leukocyte-related GOs is well in line with our previously published study^6^, which showed that genetic variation in genes involved in hematopoiesis-related pathways is important for gemcitabine/carboplatin-induced thrombocytopenia. The enrichment of cancer-related KEGG pathways could stem from that the study participants might have an underlying predisposition for cancer, and thus possibly also underlying genetic variation in genes associated with cancer. Further, cancer pathways also to a great extent include genes and effects that are important for cell growth and proliferation, which are mechanisms known to be important for myelosuppressive toxicity. Proliferation is also the underlying target of the drugs. So that the gene network modules identified in the presented analysis are constituted of genes involved in cancer and proliferation-related KEGG pathways and GOs is not farfetched and speaks in favor of the gene network modules relevance. Though functional enrichment analyses show the presence of genes and pathways affected by carboplatin and gemcitabine, we must keep in mind that the modules were constructed using only high confidence interactions from the STRING PPI network. Owing to the interactome incompleteness and limited knowledge of toxicity-associated genes, it was not obvious if the available data in STRING have enough coverage to map out modules associated with each toxicity phenotype. However, previous studies using overlapping gene network modules were able to predict molecular commonality among distinct phenotypes^28^. Simultaneously, our findings show that a gene network module approach can be used on high-throughput sequencing data to extract a module consisting of genes that are not only expressed in relevant tissues, pathways, and gene ontologies, but genes that are also differentially expressed after exposure to the drugs in question.

These results suggest that if the regulation of the module gene expression is disrupted by genetic variation, patients drug sensitivity and probability of developing toxicity could possibly be affected. Strikingly, the random LASSO prediction model based on the toxicity module could classify and predict maximal myelosuppressive toxicity with a ROC AUC of 100% (see Fig. 7b), utilizing only 62 genetic variants. While interpreting the results it should be noted that the prediction model cannot classify intermediates, as it is binomial and can only distinguish between the high and low toxicity for which it is trained and designed, therefore, the intermediates are not (and cannot) be used for calculating the ROC. However, they were included to show how they would be classified in the predictions and from it we saw (Fig. 7a) that they have characteristics of both high and low toxicity. Compared to using all nominally significant genetic variants for the predictions, we have shown that the refined model was robust enough to predict both the training and test data while increasing the model’s clinical feasibility by reducing the number of used parameters. We believe that predicting the risk of maximal toxicity is of the greatest importance. However, the toxicity module could also, to some extent, predict the individual risks of neutropenia, leukopenia, and thrombocytopenia. However, these predictions with, an average, ROC AUC of 98.8% for the individual toxicity phenotypes, were not as accurate as the maximal toxicity predictions. Using a gene network module approach and random LASSO, we not only reduced the number of parameters for the prediction model, but we also showed that there is an underlying functional interplay of the module genes supposedly relevant for myelosuppression.

In the additional validation analysis with 20% of the samples as a validation (withheld through the whole analysis), we were able to show that the resulting prediction model was still accurate enough to predict the validation samples with a ROC AUC of 73.3%. This gives an indication of how well the finalized toxicity module can perform in upcoming studies when trying to predict never seen validation samples. We are aware that the initial toxicity module approach could have imposed some overfitting problems, however, it makes the most sense to use all of the 96 samples for the gene network module construction. This enabled us to build robust and valid modules with a higher likelihood of reflecting the true underlying genes (which was also confirmed using functional enrichment) and genetic variants of importance to gemcitabine/carboplatin-induced myelosuppression.

As introduced, there are several previous studies on chemotherapy-induced myelosuppression and genetics^5,6,10–14^, When comparing the genes and genetic variants in the toxicity module with the previously reported genes and genetic variants only a few of the genes and none of the genetic variants have been previously reported with respect to chemotherapy-induced myelosuppression. These include *NCK2* and *PRKCZ* in Low et al.^13^, *SERPINA5* in Björn et al. ^6^, and *SEMG2* and *PLG* in Gréen et al. ^5^. That the overlap with our previous studies^5,6^, partly based on the same patient material, is small is likely dependent on the use of different sets of the patients, different toxicity parameters, different statistical approaches, but mainly because this study used WGS as opposed to WES in our previous studies. The other studies^10–14^ are based on different patient populations, were the underlying treatment schedule and disease is not always coherent, and some are candidate gene studies, while some are GWAS which are all factors that can affect the results and their similarity with our results. However, the main reason applies to both our and others’ studies which is the fact that the presented study is a WGS study applying a new strategy combining gene network modules and random LASSO.

Deeper into the analysis, to derive where in the genome the genetic variants were located, we used the annotations visualized in Supplementary Fig. 3. Interestingly, none of the 62 genetic variants in the toxicity module were exonic: 16 were distal intergenic, 42 were intronic, and 4 were found in promoter regions. Though mapping SNVs/INDELs to their nearest gene is debatable in terms of functional annotation, the reduced random LASSO model rendered using the gene network module approach in the presented study, we were able to predict toxicity. Among the module genes, *PDGFRA*, in which somatic mutations can lead to hematological malignancies^29,30^, contributed with over 20 nominally significant genetic variants that were included in the final prediction model. The only differentially expressed gene represented in the prediction model was *DAB2*. Interestingly, *DAB2*’s promoter is known to be methylated in oral carcinomas^31^, low *DAB2* expression promotes esophageal squamous cell carcinoma tumor progression and poor prognosis^32^, and *DAB2* is functionally linked to thrombin signaling and platelet activation in humans^33^. The gene *PLG* involved in the presented prediction model and found in our previous study^5^ is an important enzyme known to have functions related to blood cells^34–36^. Further, the tyrosine kinase-encoding genes *LYN* and *SYN* were also represented by genetic variants in the prediction model. *LYN* is in many ways involved in cancer as an oncotarget in cervical cancer^37^, associated with poor prognosis in renal cancer^38^, and as a response predictor to dasatinib in lung adenocarcinoma subpopulations^39^. *SYN* is a candidate oncogene and biomarker in some small-cell lung cancers^40^, increased *SYN* activity has also been linked to worse outcome in acute myeloid leukemia patients^41^, and it is known to be involved in agglutination and aggregation of platelets in humans^42^. This together with the functional enrichment, KEGG, and GO analyses show that the non-coding genetic variants in the prediction model identified using MCODE are associated with genes active and expressed in systems that are relevant for the treatment, cancer, and myelosuppression investigated in this study.

If the final toxicity module genes account for the underlying mechanism of action of gemcitabine and carboplatin, we expect interactors of the model genes or variants to be involved either with toxicity or the mechanism of action of the drugs. Interestingly, others have shown that cell lines with functional *TP53* show increased anti-proliferative effect when treated with carboplatin^43^, which indicates a possible direct interaction leading to toxicity. Further, pancreatic duct adenocarcinoma cells showed increased resistance to gemcitabine following *CBL* knockdown^44^, which suggests that *CBL* is important for the mechanism of action of gemcitabine. Another chemotherapeutic drug, bosutinib, in combination with gemcitabine, demonstrated antitumor activity in biliary tract carcinoma cells by inhibition of *SRC*, a known non-receptor tyrosine kinase^45^. In addition, another study showed that massively parallel sequencing coupled with dose-adjusted gemcitabine/carboplatin treatment of metastatic cancers with mutations in *PDGFRA*, *SMAD4*, and *CDKN2A* may lead to improved outcome^46^. Together with this, we have shown that the toxicity module genes are involved in cancer and hematopoiesis-related KEGG pathways and GOs. Based on this we hypothesize, in line with our previous publication^6^, that the underlying genetic differences captured in the toxicity module are likely affecting how patients bone marrow is affected by gemcitabine/carboplatin. The genetic variation might make cells in the bone marrow more sensitive to gemcitabine/carboplatin, or alter the proliferation and quality of mature blood cells, which in the end render some patients to be easily and/or harder affected by the drugs.

The proposed prediction model is solely based on germline genetics and does not utilize patient characteristics or patient baseline blood status. The patient characteristics of the high and low maximal myelosuppressive toxicity groups are homogenously distributed and listed in Table 1. This indicates that there is a significant genetic component behind the risk of chemotherapy-induced toxicity that likely includes genetic differences that affect drug pharmacokinetics and pharmacodynamics, along with the regulation, formation, and function of blood cells.

### Conclusions

The present study is, to the best of our knowledge, the most comprehensive WGS study focused on myelosuppressive toxicity induced by gemcitabine/carboplatin treatment. To conclude, we propose the toxicity module, which is associated with maximal myelosuppressive toxicity, and a model for predicting this toxicity based on 62 genetic variants. This study showcases the capability of using WGS data together with a gene network-based approach as a personalized medicine tool for the prediction of complex phenotypes, such as toxicities and ADRs. At the same time, this approach suggests an important role for the distal intergenic variation underlying myelosuppressive toxicity. We have shown that our proposed model predicts toxicity in this study, however, the model requires further evaluation and replication in other studies and in a clinical setting to be able to determine its reproducibility, usability, and clinical effect. Our presented approach and results support the usage of genetic markers for prediction of gemcitabine/carboplatin-induced myelosuppression in NSCLC patients. However, this approach is not limited to the specific toxicity, drugs, and disease, it can potentially be used for many other complex phenotypes.

## Methods

### Patient inclusion and ethical approval

A total of 215 patients diagnosed with NSCLC between 2006 and 2008 at Karolinska University Hospital, Stockholm, Sweden, were recruited for the study and included after providing written informed consent, in accordance with the Helsinki Declaration. The study received ethical approval from the regional ethics committee in Stockholm (DNR-03-413 with amendment 2016/258-32/1). These patients are part of the material included in previously published studies^5,6^.

### Treatment schedule

All patients received at least one cycle of the standard treatment protocol for NSCLC patients at the time and place of the study. Specifically, this consisted of carboplatin (target area under the concentration versus time curve = 5, on day 1) together with gemcitabine (1250 mg/m^2^ on day 1 and day 8).

### Toxicity

Neutrophil, leukocyte, and platelet counts were registered at baseline and monitored on days 8, 15, and 21 throughout the first cycle. The Nadir values of neutrophils, leukocytes, and platelets were graded according to the CTCAE version 4.03 (CTCAE grade: 0—no adverse event, 1—mild, 2— moderate, 3—severe, 4—life-threatening, 5—death related to the adverse event). The CTCAE grades were then used as the toxicity endpoint parameters for neutropenia, leukopenia, and thrombocytopenia together with the maximal toxicity registered.

### Patient selection

From the whole cohort of 215 included NSCLC patients, a subset of 96 patients were selected and used for the present study. These 96 samples were selected based on toxicity (low or high) that they experienced during the first chemotherapy cycle. In order to maximize the number of patients with low toxicity (CTCAE 0–1) or high toxicity (CTCAE 3–4) all three toxicity phenotypes, neutropenia, leukopenia, and thrombocytopenia, were considered simultaneously. During this procedure, we controlled for the distribution of the patient characteristics to be as similar as possible among the 96 selected patients to that of the whole cohort.

### DNA extraction and WGS

The QIAamp DNA Mini Kit (Qiagen) was used according to the manufacturer’s protocol to extract DNA from patient blood samples collected at baseline before treatment start. Sequencing libraries were then prepared with the TruSeq DNA PCR-Free Library Preparation kit (Illumina), according to the manufacturer’s protocol, before the samples were whole-genome sequenced at the Science for Life Laboratory (SciLifeLab, Stockholm, Sweden) using the HiSeq X Ten platform (Illumina).

### Alignment and variant calling of WGS data

Initially, cutadapt version 1.9.1^47^ was used for quality and adapter trimming the raw sequencing reads. The reads were then mapped to the human reference genome, GRCh37, using BWA aligner version 0.7.12^48^. Then Picard Tools (http://www.picard.sourceforge.net/) was used to discard any duplicate reads and SAMtools version 0.1.19^49^ was used to filter out reads not primary aligned or not in proper pairs. Thereafter, variants were called using the Genome Analysis Toolkit (GATK) version 3.3.0^50^ applying their best practices^51^. Quality was monitored throughout the process using QualiMap version 2.0^52^. After variant calling, VCFtools version 0.1.14^53^ was applied to filter out variants not labeled as PASS, with a genotyping rate <0.95, a coverage <5, or a mean coverage <10 across all samples.

### SNV/INDEL association analysis

Case/control implementation of two-sided Fisher’s exact test in an allelic fashion^54^ was performed using PLINK version 1.90^55^ for association analysis between bi-allelic SNVs and INDELs to neutropenia, leukopenia, and thrombocytopenia. For these analyses, patients with CTCAE grades 0–1 were used as controls, and patients with grades 3–4 were used as cases. This means that patients with intermediate toxicity (CTCAE grade 2) were left out of the respective statistical analyses.

Principal component analysis (PCA) was performed with the function snpgdsPCA in the package SNPRelate version 1.16.0^56^ for R version 3.5.2^57^ using all SNVs/INDELs and only the nominally significant (*p* ≤ 1 × 10^−3^) SNVs/INDELs. Further, the VCF file was annotated using the R-package ChIPseeker version 1.18.0^58^. The same package was also used for mapping all genetic variants to their respective closest genes. Plots were constructed using the R packages ggplot2 version 3.1.1 and ggpubr version 0.2.

### Gene network modules

All autosomal nominally significant SNVs/INDELs were mapped to their nearest protein-coding gene within a 3000 kilobase distance upstream and downstream. The mapped genes were used as seeds to identify gene network modules. The background network used was the STRING protein–protein interactions (PPI) network version 10.5^24^ with all the high confidence (combined score >700) interactions. The graph-theoretic clustering algorithms MCODE^18^, DIAMOnD^25^, CliqueSuM^26^, and ModuleDiscoverer^27^ were implemented for overlaying the seeds on the network and inferring the modules. These algorithms are seed based but use different network properties for module inference. DIAMOnD is an iterative algorithm which uses connectivity significance to infer the large connected component in the background network starting from the input seed genes^25^. MCODE is an algorithm based on vertex weighting and *k*-means clustering allowing cluster interconnectivity to infer modules^18^. CliqueSuM and ModuleDiscoverer are clique-based algorithms in which the maximal cliques from the network are identified and compared against random subgraphs of equal size for calculating significance^26,27^. The analyses were performed using the R package MODifieR version 0.1.4 (https://gitlab.com/Gustafsson-lab/MODifieR)^59^ for module inference.

### Analysis of bone marrow from rats and humans

For validating the different gene network modules relevance for myelosuppressive toxicities we performed enrichment analysis using genes differentially expressed in rat bone marrow upon exposure to gemcitabine and carboplatin. Specifically, we used bone marrow gene expression data from rats treated with 78 drugs available under the accession number GSE59894 in the NCBI Gene Expression Omnibus database (https://www.ncbi.nlm.nih.gov/geo/). In order to identify differentially expressed genes (DEGs) affected by drugs, we implemented Tukey’s post hoc analysis on the timepoint with 72 hours of drug exposure, independently comparing gemcitabine and carboplatin with all other drugs. From the findings of this step, the specific effects of carboplatin and gemcitabine were obtained by combining all the *p*-values of comparisons with the other drugs at the 72-hour timepoint using Fisher’s method. Next, the genes significant after Bonferroni correction (adjusted *p*-values with false discovery rate (FDR) < 0.05) were mapped to their human homologous genes using the R package Biomart version 2.40.3^60,61^. The resultant list of DEGs for gemcitabine and carboplatin were then used to test their overlap with the constructed gene network modules using two-sided Fisher’s exact test.

In addition to rat bone marrow data, we also compared the enrichment of the gene network modules with our previous meta-analysis gene expression data from human bone marrow and kidney concerning treatment with platinum analogs and/or gemcitabine, as described and obtained in refs. ^5,62^.

### Cell lines for RNA-seq

Two human cell lines exhibiting megakaryocyte-like properties, CMK (ACC-392)^63,64^ and MOLM-1 (ACC-720)^65–67^, both from the Leibniz-Institute DSMZ—German Collection of Microorganisms and Cell Cultures, and the myelogenous cell line K562 (CCL-243)^68–70^ from the American Type Culture Collection were used.

### Cell culturing

The cell lines were cultured using RPMI 1640 supplemented with 10% FBS and 2% penicillin/streptomycin, all from Gibco, Life Technologies. They were passaged every 3–4 days and kept at 37 °C in a humidified atmosphere containing 5% CO_2_ and the passage numbers were kept below 15 from the acquisition. The cells were tested (negative) for mycoplasma infections utilizing the service Mycoplasmacheck (GATC Biotech) following the manufacturer’s instructions.

### Drug incubations

Experiments were initialized by seeding 10 million cells in 15 ml of RPMI 1640 with 10% FBS without antibiotics and treating them for 24 hours with gemcitabine (Toronto Research Chemicals), carboplatin (Toronto Research Chemicals), or no drug (as a control). The drug concentrations used for the 24-hour treatments of K562, CMK, and MOLM-1 were the 72-hour half-maximal inhibitory concentration (IC50) concentrations for each respective cell line, which were specifically determined, using the MTT assay (Molecular Probes, Life Technologies), to be 14.29 ng/ml, 24.84 ng/ml, and 34.67 ng/ml for gemcitabine, and 29.58 µg/ml, 1.61 µg/ml, and 14.27 µg/ml for carboplatin, for K562, CMK, and MOLM-1, respectively. All treatments were carried out in duplicate, resulting in 18 samples. Duplicate samples were run at different times to ensure biological replication.

### RNA extraction and sequencing

After the drug incubations, RNA from 1 ml of cell suspension of each sample was extracted using the RNeasy Mini Kit (Qiagen) and QIAshredder (Qiagen) according to the manufacturer’s protocol. Ribosomal RNA was depleted using RiboCop rRNA Depletion Kit version 1.2 (Lexogen), and sequencing libraries were prepared with SENSE Total RNA-Seq Library Prep Kit (Lexogen) following the manufacturer’s protocol. Libraries were sequenced at Science for Life Laboratory (SciLifeLab, Stockholm, Sweden) using the HiSeq 2500 (Illumina) with HiSeq Rapid SBS Kit v2 chemistry and a 1 × 51 setup.

### Alignment and read summarization of RNA-seq data

The raw RNA-seq reads were quality and adapter trimmed using TrimGalore! version 0.4.4 (http:// www.bioinformatics.babraham.ac.uk/projects/trim_galore/) and cutadapt version 1.13^47^. STAR version 2.5.3a^71^ was used for aligning the reads to the human reference genome, GRCh37. Thereafter, the aligned sam files were converted to bam files and sorted using SAMtools version 1.9. Only uniquely mapping reads were used in the subsequent analyses, and read summarization was conducted using featureCounts, which is available in the software package Subread version 1.5.2^72^, to summarize the number of reads per gene region. Only reads spanning one gene region were counted. The quality of the data was monitored through all steps using FastQC version 0.11.5, QualiMap version 2.2^52^, and MultiQC version 1.6^73^.

### Gene expression analysis

The output matrix with read/transcript counts from featureCounts was used as input to R version 3.5.2^57^. Transcripts per million (TPM) were calculated, and differential expression analysis was conducted separately for the three cell lines and their respective treatments, using edgeR version 3.18.1^74,75^. Only fragments with TPM > 1 in ≥2 samples were used for the differential gene expression analysis, and they were normalized using the TMM method^76^.

Both the TPM and differential expression results were filtered to only output data on the genes included in the finally obtained toxicity module from MCODE (see the results in the section “Gene network modules”). We also performed 10,000 permutations by randomly taking genes (*n* = 215) equal to the size of the toxicity module (after fragments with TPM = 0 in all 18 samples had been removed) and counting how many genes were expressed with TPM > 1 in ≥2 samples for all permutations. This was compared to see if more module genes were expressed than expected by chance.

### KEGG pathway and Gene Ontology (GO) enrichment analysis

The R package clusterProfiler version 3.12.0^77^ was used for KEGG pathway and GO-enrichment analyses of the toxicity module genes.

### Prediction of toxicity using random LASSO

To categorize the patients as high or low toxicity based on their genetics, the random LASSO^19^ was implemented for variable selection in generalized linear models using the function cv.glmnet in the R package glmnet version 2.0-16. To do this, all nominally significant genetic variants (SNVs/INDELs) that mapped to the genes in the gene network modules found using MCODE^18^ were used. The function cv.glmnet used 10-fold cross-validation, a randomized normally distributed penalization factor, *α* = 1, and nlambda = 100. It was permuted 100,000 times against the binomial traits low or high myelosuppressive toxicity using the model fits with the lowest cross-validation error (lambda.min). For validating the model, 20% of the samples with high toxicity and low toxicity were withheld as test data. The numbers of low and high toxicity samples, along with the numbers of training and test samples, are listed in Supplementary Table 10. Sets of the quantiles of the genetic variants (based on their selection frequency) from the random LASSO permutations were evaluated further to determine their specific lambda values using the same function as above, however, with *α* = 0 (i.e. no further shrinkage). The set of genetic variants with the best predicting capacity, determined by evaluating the receiver operating characteristic (ROC) and AUC when predicting the training and test data, is presented as the final prediction model for maximal toxicity.

### Additional validation analysis

In this analysis the 96 samples were independently of the previous analysis split up into 80% training and 20% validation based on maximal toxicity: 44/10 (training/validation) high toxicity (CTCAE 3–4), 0/8 intermediate (CTCAE 2), and 28/6 low toxicity (CTCAE 0–1). The training samples were taken through Fisher’s exact test, before gene network module construction using MCODE and STRING PPI. Since the training data now has a little lower power due to smaller number of samples, the module generation using MCODE required a change of a parameter called vertex weight percentage (VWP) from the default value (0.5) to 0.1. The density and size of the module will be defined by this parameter^18^. We tuned this parameter to have optimal size of the module that is comparable with the previous analysis. After this the nominally significant genetic variants overlapping between all three toxicity phenotype modules were used for 100,000 random LASSO permutations to elucidate the set of genetic variants with the best prediction capacity. Lastly, the best prediction model using this approach was used to predict the never seen validation samples.

### Reporting summary

Further information on research design is available in the Nature Research Reporting Summary linked to this article.

## Supplementary information

Supplementary file

Reporting summary

## Data Availability

The datasets generated during and/or analyzed during the current study are not publicly available due to that this is not permitted by the ethical approval of the study but are available from the corresponding author (N.B., niclas.bjorn@liu.se) on reasonable request together with the appropriate ethical approval.

## Code availability

We utilized freely available open-source functions and programs all referred to along with the specific version numbers indicated in the Methods section.
