# Supplementary material for: Whole-genome sequencing and gene network modules predict gemcitabine/carboplatin-induced myelosuppression in non-small cell lung cancer patients
Source: NPJ Syst Biol Appl. 2020 Aug 24;6:25. doi: 10.1038/s41540-020-00146-6 (PMC7445166; doi:10.1038/s41540-020-00146-6)
Supplement: Supplementary file 1 — Supplementary file [file 41540_2020_146_MOESM1_ESM.pdf]

Whole-genome sequencing and gene network modules predict gemcitabine/carboplatin-induced myelosuppression in non-small cell lung cancer patients

Niclas Björn[1*], Tejaswi Venkata Satya Badam[2,3], Rapolas Spalinskas[4], Eva Brandén[5,6], Hirsh Koyi[5,6], Rolf Lewensohn[7], Luigi De Petris[7], Zelmina Lubovac-Pilav[3], Pelin Sahlén[4], Joakim Lundeberg[4], Mika Gustafsson[2], and Henrik Gréen[1,4,8]

[1] Clinical Pharmacology, Division of Drug Research, Department of Biomedical and Clinical Sciences, Linköping University, Linköping, Sweden.

[2] Bioinformatics, Department of Physics, Chemistry and Biology, Linköping University, Linköping, Sweden.

[3] School of Bioscience, Systems Biology Research Centre, University of Skövde, Skövde, Sweden.

[4] Science for Life Laboratory, School of Engineering Sciences in Chemistry, Biotechnology and Health, Department of Gene Technology, KTH Royal Institute of Technology, Solna, Sweden.

[5] Department of Respiratory Medicine, Gävle Hospital, Gävle, Sweden.

[6] Centre for Research and Development, Uppsala University/Region Gävleborg, Gävle, Sweden.

[7] Thoracic Oncology Unit, Tema Cancer, Karolinska University Hospital, and Department of Oncology-Pathology, Karolinska Institutet, Stockholm, Sweden.

[8] Department of Forensic Genetics and Forensic Toxicology, National Board of Forensic Medicine, Linköping, Sweden.

* Corresponding author: Niclas Björn (niclas.bjorn@liu.se).

Supplementary File

In this PDF the Supplementary Figures (1-6) and Supplementary Tables (1-10) are presented.

Supplementary Figures

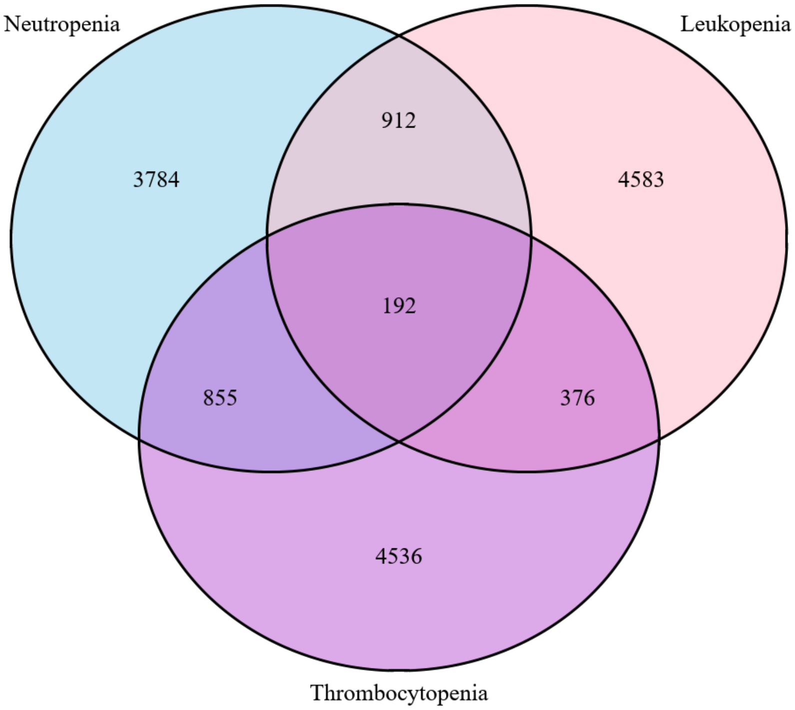

Supplementary Figure 1. Venn diagram showing the number of nominally significant ($p \leq 1 \times 10^{-3}$) genetic variants (SNVs/INDELs) for the three toxicity phenotypes neutropenia, leukopenia, and thrombocytopenia as well as their overlaps.

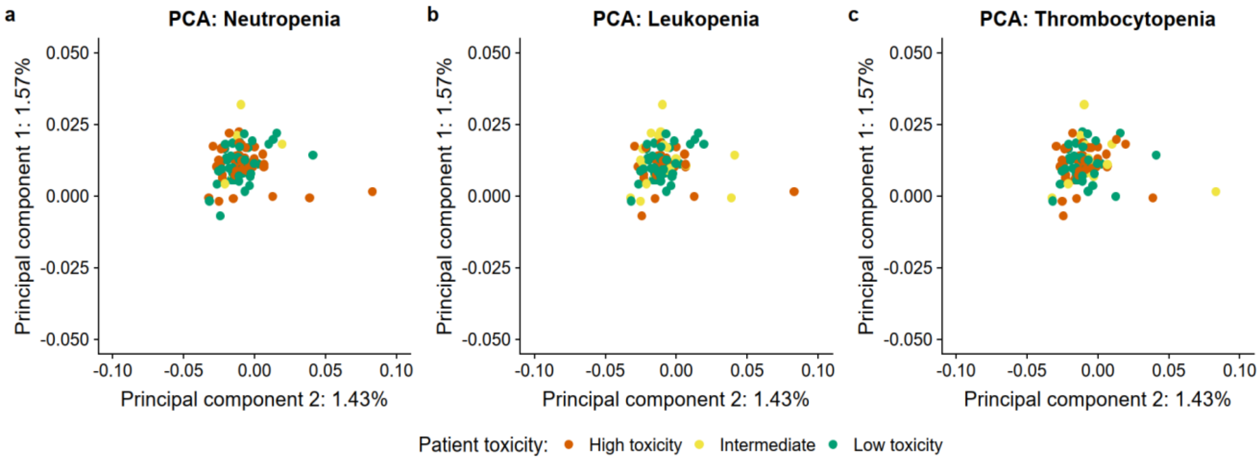

Supplementary Figure 2. Principal component analysis (PCA) using all filtered SNVs/INDELs. The first principal component explains 1.57% of the variability while the second principal component explains 1.43%. No apparent clustering can be observed when color-coding the samples according to the respective toxicities neutropenia (a), leukopenia (b), or thrombocytopenia (c).

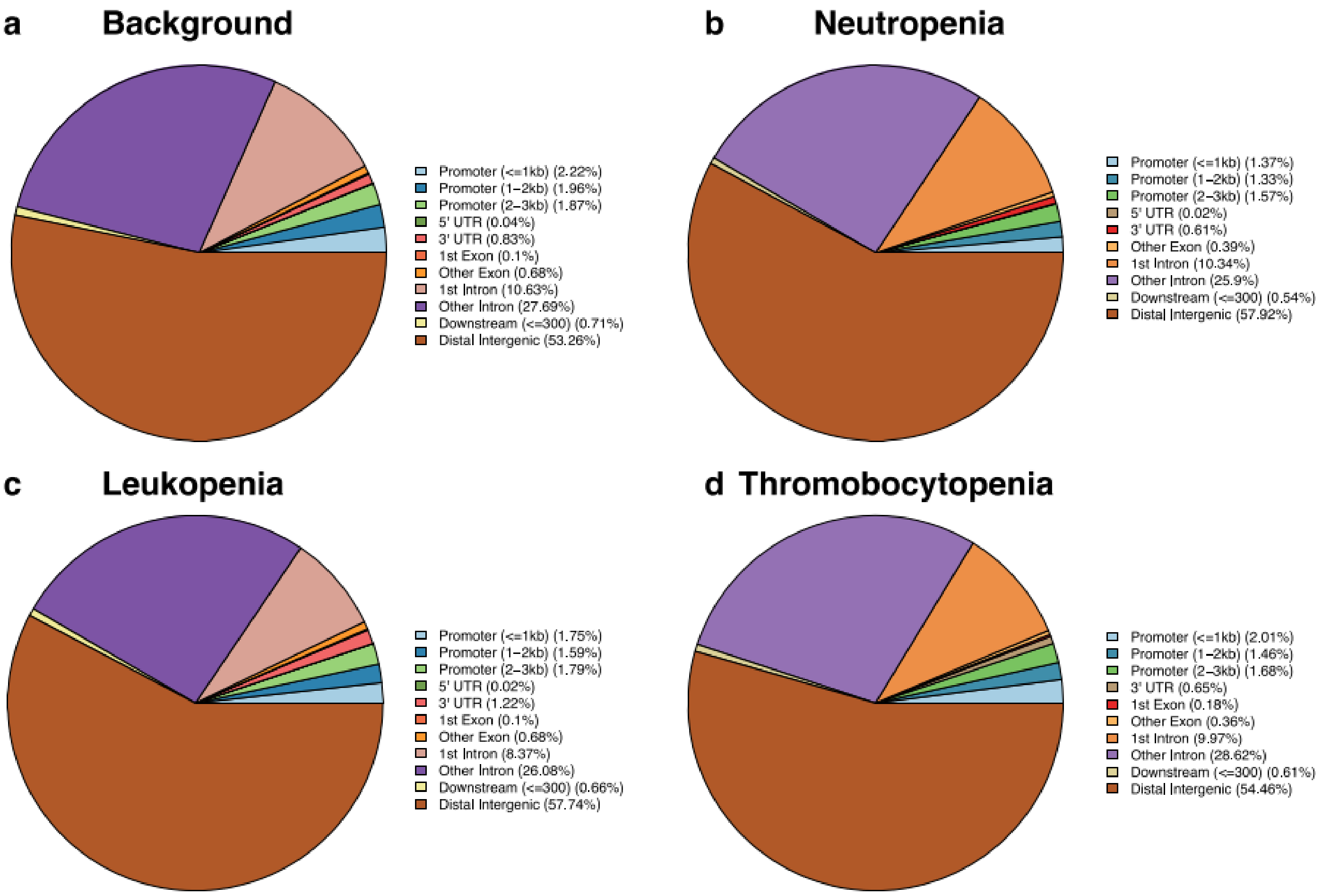

Supplementary Figure 3. Annotation of genetic variants. The distribution of all genetic variants, i.g. the background distribution is shown in (a). The distributions of the nominally significant ($p \leq 1 \times 10^{-3}$) genetic variants are shown for neutropenia (b), leukopenia (c), and thrombocytopenia (d). Interestingly, a slight enrichment of the proportion of distal intergenic variants is seen in (b), (c), and (d) compared to the background in (a).

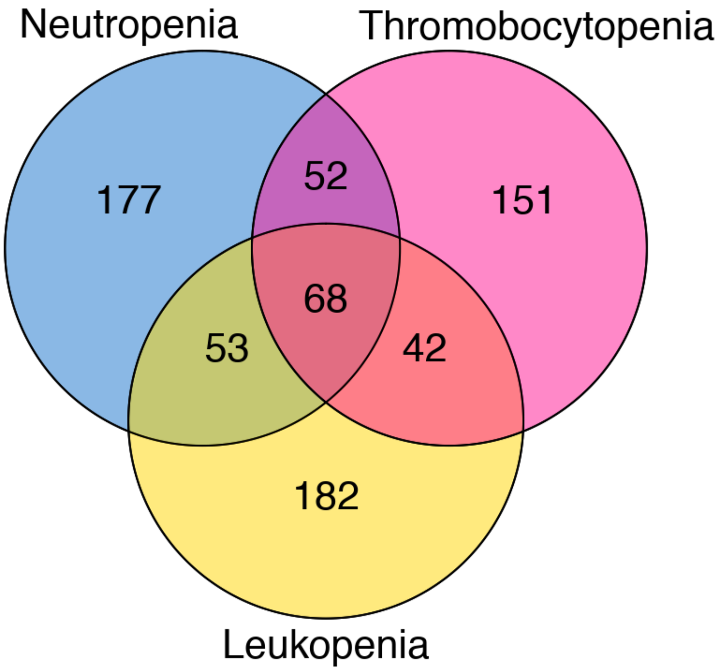

Supplementary Figure 4. Venn diagram showing the number of genes in the MCODE modules for the three toxicity phenotypes neutropenia, leukopenia, and thrombocytopenia as well as their overlap.

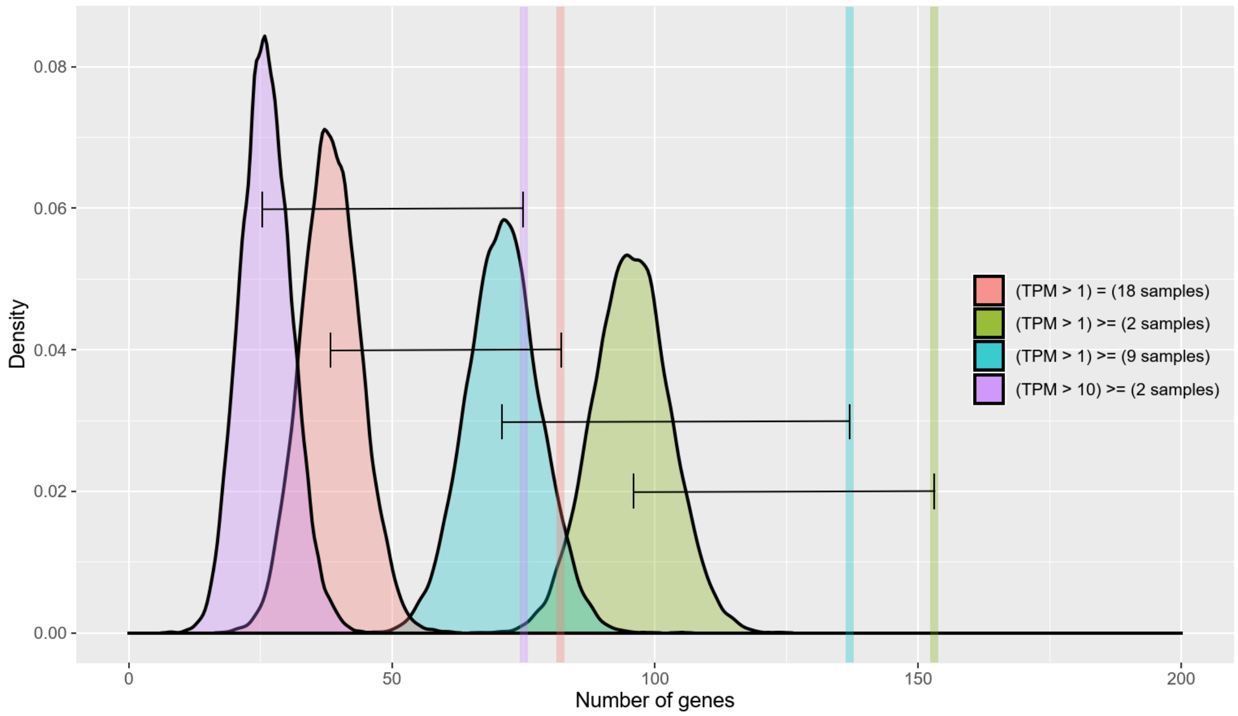

Supplementary Figure 5. The number of genes expressed in the 18 samples (duplicates of 3 cell lines under 3 conditions (gemcitabine, carboplatin or untreated)) above the specific transcripts per million (TPM) thresholds by our 215 toxicity module genes are represented by the four lines in the graph. We also did 10000 permutations randomly selecting 215 genes from the RNA-seq data (after removing zeros in the data the random selections were made from 27424 gene fragments (of which 12166 have TPM >1 in at least 2 samples)) and the histograms represent the distribution of the number of fragments over the specific TPM thresholds. We can see that more genes are expressed in the cell lines, at all TPM thresholds, than what is expected by chance.

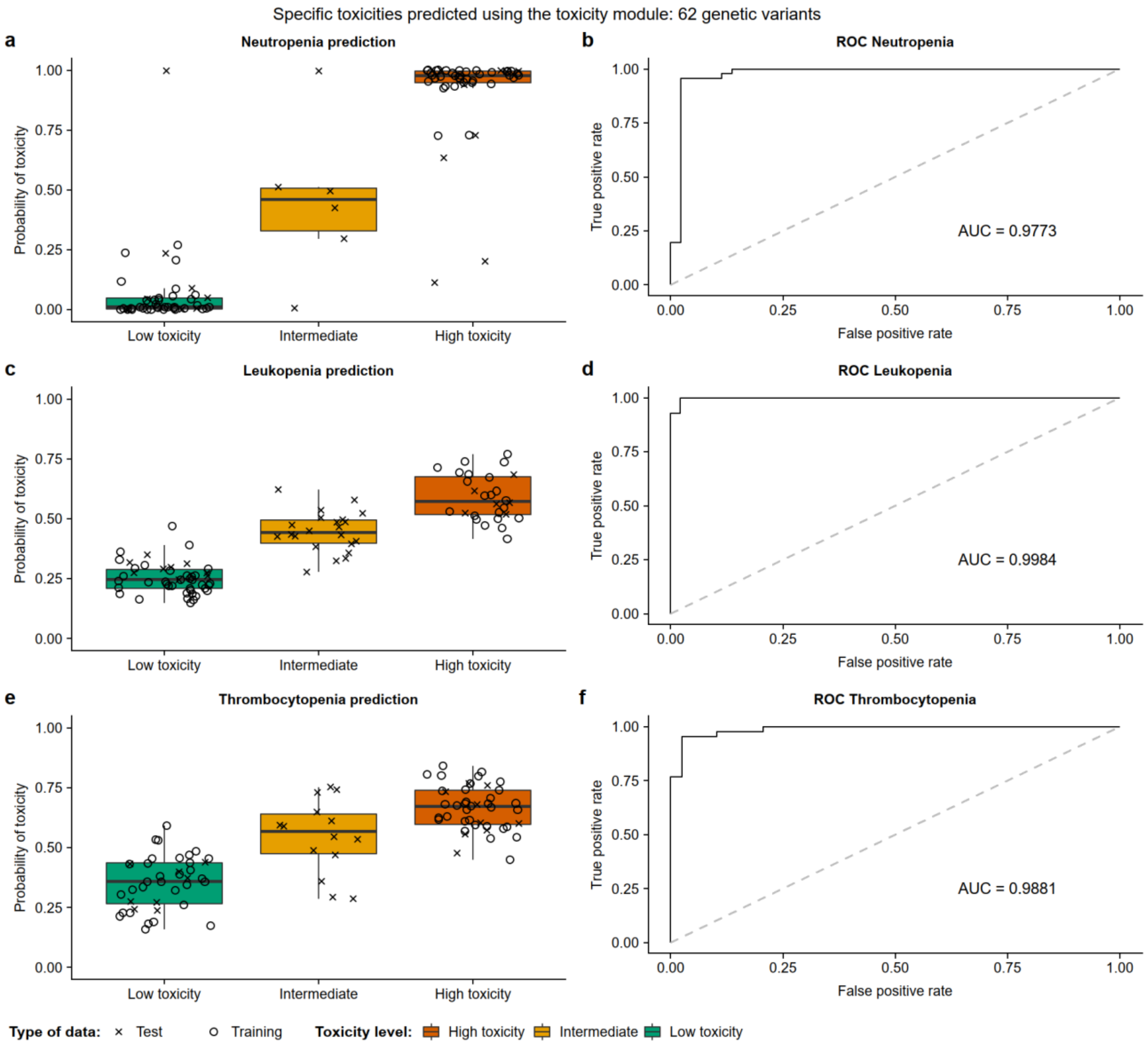

Supplementary Figure 6. Shows the prediction of neutropenia, leukopenia, and thrombocytopenia using the 62 genetic variants from the best maximal toxicity prediction model based on the toxicity module. a) Patients (separated by registered neutropenia) and their predicted probability of neutropenia. b) ROC curve of the model's predictions of high and low neutropenia. c) Patients (separated by registered leukopenia) and their predicted probability of leukopenia. d) ROC curve of the model's predictions of high and low leukopenia. e) Patients (separated by registered thrombocytopenia) and their predicted probability of thrombocytopenia. f) ROC curve of the model's predictions of high and low thrombocytopenia.

Note that the intermediates are not used for calculating the ROC. The box-plot elements should be interpreted as the following: centerline, median; box limits, upper and lower quartiles; whiskers, 1.5x interquartile range.

Supplementary Tables

On the following pages the supplementary tables are presented. These supplementary tables are also available in their original .xlsx format from the corresponding author (NB, niclas.bjorn@liu.se).

Supplementary Table 1. Nominally significant genetic variants for neutropenia.
Supplementary Table 2. Nominally significant genetic variants for leukopenia.
Supplementary Table 3. Nominally significant genetic variants for thrombocytopenia.
Supplementary Table 4. All MCODE module genes.
Supplementary Table 5. Module rat data enrichment comparison.
Supplementary Table 6. Toxicity module genes expressed in cell lines.
Supplementary Table 7. Differentially expressed toxicity module genes.
Supplementary Table 8. Enriched KEGG pathways and Gene Ontologies.
Supplementary Table 9. Coefficients for all prediction models.
Supplementary Table 10. Number of training and test data samples.

Supplementary Table 1. All nominally significant (p≤0.001) genetic variants (SNVs/INDELs) for neutropenia as determined by Fisher's exact test.

rsID	Chromosome	Position	Minor allele	Minor allele frequency among cases	Minor allele frequency among controls	Major allele	p-value
rs202004563	1	63643	G	11	0	A	0.0006945
rs201637762	1	103058	T	11	0	C	0.0007228
rs375234444	1	103234	A	11	0	G	0.0007086
rs368811019	1	124064	A	11	0	C	0.0007274
1:668641	1	668641	G	17	2	T	0.0004014
rs28595293	1	1122915	G	13	32	A	0.0009401
rs6684820	1	1124663	A	13	32	G	0.0009401
rs78174849	1	1128778	CTTA	18	39	C	0.0004141
rs9727857	1	1130843	G	12	32	A	0.0004387
rs9329409	1	1131581	C	13	32	T	0.0009401
rs2274789	1	1133077	G	12	32	A	0.0004387
rs12031928	1	1133273	C	12	32	T	0.0004387
rs12405246	1	1133787	C	12	32	T	0.0004387
rs35009659	1	1133815	C	12	32	T	0.0004387
rs9729550	1	1135242	C	12	32	A	0.0004387
rs1815606	1	1140435	T	20	42	G	0.0002952
rs12760917	1	4770065	C	46	21	T	0.0003668
rs2235516	1	14140457	A	20	4	G	0.0007606
rs2697960	1	14153837	A	23	5	G	0.0003619
rs2744677	1	14154723	G	30	8	T	0.0001064
rs149580705	1	14154806	A	29	8	AGTAAACTTACCTGTTTCTG	0.0001956
rs2697983	1	14157577	A	30	8	G	0.0001064
rs195738	1	24806560	C	41	17	T	0.0007198
rs4649044	1	25342567	C	5	21	T	0.0005184
rs11802335	1	25344494	C	5	23	T	0.0001426
rs11588079	1	25367084	G	22	43	T	0.0006167
rs4649056	1	25371793	A	21	41	G	0.0009669
rs12753065	1	25374586	G	21	41	C	0.0009669
rs4649057	1	25375177	C	28	50	T	0.0005077
rs4649058	1	25375468	A	29	50	G	0.0009027
rs11248970	1	25375561	G	29	50	A	0.0009027
rs56127605	1	25381311	A	33	55	G	0.0005652
rs12075549	1	25381526	C	51	27	T	0.0009437
rs10437112	1	25384437	G	49	24	A	0.0004678
rs4649060	1	25384533	T	50	24	G	0.0002688
rs182642985	1	25385193	G	51	24	A	0.0001515
rs137983526	1	25385759	A	50	24	C	0.0002688
1:33442122	1	33442122	C	3	18	CACACACACACA	0.0007476

rs143567912	1	34140788	G	1	14	T	0.0002433
1:34672864	1	34672864	G	28	50	GT	0.0005077
rs58814335	1	34672865	G	26	48	T	0.0004647
rs3917937	1	36943141	A	51	22	G	0.00003821
rs3917932	1	36943916	C	54	26	G	0.00009512
rs4026505	1	36950775	A	48	24	T	0.0007984
rs6667860	1	36958213	C	57	32	T	0.0006468
rs2247886	1	38931754	A	23	46	C	0.0002182
rs2235544	1	54375570	C	33	55	A	0.0005652
rs61674130	1	54389774	TAG	33	55	T	0.0005652
rs12127960	1	54419526	T	32	54	A	0.0005601
rs651381	1	54450998	G	33	55	A	0.0005652
rs607776	1	54459574	A	53	31	C	0.0007953
rs12036530	1	54476323	C	32	53	T	0.0009656
rs41366847	1	54476967	G	32	53	C	0.0009656
rs10158824	1	54480398	C	33	54	G	0.0009764
rs1537321	1	54482236	A	31	53	C	0.0005517
rs1537322	1	54482306	T	31	53	G	0.0005517
rs1537323	1	54482523	G	31	53	T	0.0005517
rs11804837	1	54482915	A	31	53	G	0.0005517
rs7540270	1	54484682	G	31	53	A	0.0005517
rs67676007	1	54485779	A	30	52	G	0.00054
rs79534351	1	58501725	G	2	15	A	0.0006243
rs34827621	1	68642064	G	2	15	A	0.0006243
rs12568058	1	69987293	G	30	10	T	0.0006347
rs7516464	1	71518153	T	33	8	A	0.00001568
rs880098	1	71520103	C	57	32	T	0.0006468
rs3765414	1	71520652	T	18	2	C	0.000203
rs34797173	1	71521464	CA	33	8	C	0.00001568
rs11365325	1	71521607	CT	57	32	C	0.0006468
rs2817869	1	71522404	C	49	22	T	0.0001287
rs60800640	1	71524239	AC	50	24	A	0.0002688
rs4650099	1	71524310	A	57	32	G	0.0006468
rs4128461	1	71526710	T	57	32	C	0.0006468
rs4579709	1	71527491	G	18	2	A	0.000203
rs4520371	1	71527548	C	57	32	T	0.0006468
rs7411	1	71529401	T	50	24	C	0.0002688
rs66461782	1	71533287	T	33	9	A	0.00004367
rs1138177	1	71535608	C	33	8	T	0.00001568
rs11209745	1	71537943	G	33	8	A	0.00001568

rs9425055	1	71541003	C	18	2	T	0.000203
rs4650100	1	71543877	C	57	32	A	0.0006468
rs12035210	1	71545970	A	33	8	G	0.00001568
rs7549846	1	71548122	G	18	3	C	0.0008121
rs7519104	1	71548467	G	33	9	T	0.00003093
rs58864545	1	71554002	GCA	47	17	G	0.00001055
rs12044810	1	71554014	G	18	3	A	0.0008121
rs12084460	1	71556842	A	33	9	G	0.00004367
rs6680193	1	71556899	G	50	24	A	0.0002688
rs7522835	1	71557241	T	18	3	C	0.0008121
rs9424959	1	71558452	G	18	3	A	0.0008121
rs4272572	1	71561084	A	50	24	G	0.0002688
rs9783035	1	71562923	C	18	3	T	0.0008121
rs4146736	1	71569333	G	18	3	A	0.0008121
rs11209746	1	71572114	G	50	24	A	0.0002688
rs56254112	1	71574825	G	34	9	C	0.00002308
rs201695113	1	71575618	C	18	3	T	0.0008121
rs9425057	1	71576970	G	50	24	A	0.0002688
rs150907838	1	71577894	T	18	3	G	0.0008121
rs5775050	1	71583630	C	33	9	CCTT	0.00004367
rs67335874	1	71584260	ATATCT	50	24	A	0.0002688
rs2817842	1	71585378	C	50	24	A	0.0002688
rs9424960	1	71586388	C	50	24	T	0.0002688
rs4390127	1	71586909	A	50	24	G	0.0002688
rs2817845	1	71587698	C	50	24	G	0.0002688
rs7537624	1	71588369	A	50	24	C	0.0002688
rs9425060	1	71589384	G	18	3	A	0.0008121
rs71095930	1	71590995	T	33	6	TC	0.000002815
rs34717496	1	71591196	TTC	50	24	T	0.0002688
rs2817847	1	71593676	T	18	3	G	0.0008121
rs2817848	1	71595348	G	33	9	A	0.00004367
rs4367753	1	71596192	G	46	17	T	0.00002027
rs1578681	1	71598851	A	50	24	G	0.0002688
rs12565508	1	71599507	G	18	3	A	0.0008121
rs2796196	1	71599651	T	50	24	G	0.0002688
rs2796197	1	71603644	T	50	24	C	0.0002688
rs2817851	1	71603703	A	50	24	G	0.0002688
rs5775051	1	71604136	T	33	9	TA	0.00004367
rs2796198	1	71604867	G	33	9	A	0.00004367
rs12144622	1	71605107	C	50	24	G	0.0002688

rs2796199	1	71609102	C	33	9	T	0.00004367
rs2817853	1	71614212	T	50	24	C	0.0002688
rs10889909	1	71615678	T	50	24	C	0.0002688
rs10465815	1	71615858	G	33	9	A	0.00004367
rs6424420	1	71617388	C	18	3	A	0.0008121
rs1832900	1	71619556	A	50	24	C	0.0002688
rs1418449	1	71632687	T	46	17	G	0.00002027
rs12747444	1	71634219	A	18	3	G	0.0008121
rs4425938	1	71637113	T	46	17	C	0.00002027
rs6424422	1	71642471	T	46	17	A	0.00002027
rs59167530	1	71650947	A	31	10	AT	0.0003611
rs113319318	1	71651728	A	31	10	ATCAC	0.0003611
rs74365843	1	71652676	T	47	16	C	0.000004496
rs1417709	1	71656481	A	47	17	G	0.00001055
rs1954286	1	71657212	A	51	24	G	0.0001515
rs67184325	1	71658411	AT	47	18	A	0.00002365
rs2639329	1	71660120	G	47	17	A	0.00001055
rs2796201	1	71660796	C	47	17	T	0.00001055
rs2796202	1	71662048	A	51	24	G	0.0001515
rs1954285	1	71662977	T	47	16	A	0.000004496
rs952337	1	71663414	T	47	16	A	0.000004496
rs71095934	1	71664305	GGAGGCTGAGGCAA	57	32	G	0.0006468
rs2796203	1	71665671	T	47	16	C	0.000004496
rs2066371	1	71667881	G	47	17	A	0.00001055
rs1573061	1	71668906	C	47	17	T	0.00001055
rs2639338	1	71672398	A	47	16	G	0.000004496
rs2639339	1	71672677	G	47	16	C	0.000004496
rs2639340	1	71673009	A	51	24	G	0.0001515
rs12406316	1	71673738	T	47	16	C	0.000004496
rs2639335	1	71676127	A	47	16	G	0.000004496
rs2639336	1	71676720	A	31	10	C	0.0003611
rs2639334	1	71677428	G	47	17	A	0.00001055
rs2639333	1	71678935	G	51	25	A	0.0002873
rs2639332	1	71679004	A	31	10	C	0.0003611
rs7513433	1	71679679	T	18	3	C	0.0008121
rs2249759	1	71681377	C	47	17	T	0.00001055
rs375061957	1	71682194	C	51	25	G	0.0002873
1:71682226	1	71682226	G	51	25	A	0.0002873
rs370402759	1	71683102	G	47	17	C	0.00001055
rs138432234	1	71684405	C	57	32	T	0.0006468

rs12759919	1	71685633	T	47	16	G	0.000004496
rs200781733	1	71685857	G	18	3	A	0.0008121
rs9726375	1	71686237	C	47	16	A	0.000004496
rs11588351	1	71689021	A	57	32	G	0.0006468
rs2796186	1	71692659	A	51	24	G	0.0001515
rs11209753	1	71693046	A	57	32	G	0.0006468
rs2796187	1	71693853	A	51	25	G	0.0002873
rs2796188	1	71694861	G	51	25	A	0.0002873
rs2796189	1	71694883	C	51	25	T	0.0002873
rs2796190	1	71698274	A	47	16	G	0.000004496
rs2639331	1	71698461	T	47	16	C	0.000004496
rs2796192	1	71698946	C	47	17	G	0.00001055
rs2639330	1	71699013	T	47	16	C	0.000004496
rs34196037	1	71700130	C	48	21	T	0.0001169
1:71700132	1	71700132	C	48	19	T	0.00002719
rs12119958	1	71703438	A	57	32	G	0.0006468
rs2694955	1	71705104	A	51	25	T	0.0002873
rs2694954	1	71705578	G	51	25	T	0.0002873
rs1431503	1	71706106	T	18	3	C	0.0008121
rs1431504	1	71712759	G	31	11	A	0.0008085
rs2790675	1	71718838	G	44	16	A	0.00003243
rs2790674	1	71719363	C	44	15	T	0.00001416
rs1546809	1	71723278	G	44	16	T	0.00003243
rs11209757	1	71728900	G	48	23	T	0.0004351
rs7527741	1	71740325	C	32	9	A	0.00008145
rs1367768	1	71745710	A	33	9	G	0.00004367
rs35251740	1	71746844	CT	21	4	C	0.0001738
rs7415084	1	71751307	C	32	9	T	0.00008145
rs6658862	1	71752476	G	32	10	T	0.0002023
rs7540435	1	71753041	C	33	9	T	0.00004367
rs34086176	1	71753953	C	35	10	CA	0.0000652
rs6695355	1	71762203	G	32	9	A	0.00008145
rs11209761	1	71764175	T	48	24	C	0.0007984
rs12723074	1	81471586	G	17	37	T	0.0006349
rs76879528	1	85234319	A	24	6	T	0.0005592
1:85234608	1	85234608	GTGTGTA	24	6	G	0.0005592
rs77512569	1	85235551	A	24	6	C	0.0005592
rs74510508	1	85235837	T	24	6	C	0.0005592
rs57189278	1	85235859	A	24	6	G	0.0005592
rs77176056	1	85236425	G	24	6	C	0.0005592

rs17369401	1	85314611	A	23	4	G	0.0001119
rs17369470	1	85318376	A	14	1	G	0.0006347
rs17378045	1	85318907	A	23	4	G	0.0001119
rs74364002	1	85328742	T	14	1	C	0.0006103
rs368186205	1	85615108	G	28	8	A	0.0003134
rs11165287	1	95498978	T	1	15	C	0.0001157
rs116733964	1	99906088	A	11	0	G	0.0007274
1:142577113	1	142577113	G	21	4	A	0.0004069
rs56801406	1	157044993	G	25	7	C	0.0008092
rs59462822	1	157045213	A	25	7	G	0.0008092
rs35188764	1	157046198	G	22	5	A	0.0006676
rs11587380	1	157048220	G	25	7	C	0.0008092
rs11578226	1	157051348	C	25	7	T	0.0008092
rs149171885	1	157052071	TCAGTTTGGCATTTTAAACA(	25	7	T	0.0008092
rs34414479	1	157052224	A	25	7	G	0.0008092
rs1176561	1	157053342	T	22	5	C	0.0006676
rs12136960	1	157062739	G	30	9	C	0.0002709
rs76614224	1	157453597	G	11	30	C	0.0006171
rs12120811	1	157454010	G	18	39	A	0.0004141
rs10918046	1	164526190	G	16	1	A	0.0001529
rs7549447	1	165288621	A	48	24	T	0.0007984
rs6688855	1	165291536	G	48	24	T	0.0007984
rs12564138	1	165292079	G	48	24	C	0.0007984
rs4656444	1	165293947	T	48	24	C	0.0007984
rs1504702	1	165294398	C	48	24	T	0.0007984
rs1504703	1	165294528	C	48	24	G	0.0007984
rs2553640	1	165466877	A	3	17	G	0.0006426
rs285498	1	165467808	A	3	17	C	0.0006426
rs2766085	1	165471470	G	2	17	A	0.0001534
rs374659495	1	168545583	G	2	16	A	0.0003115
rs367943926	1	168545584	T	2	16	C	0.0003115
rs3917734	1	169580463	A	12	33	G	0.0002456
rs3917733	1	169580510	T	12	33	C	0.0002456
rs3917731	1	169580717	C	12	33	T	0.0002456
rs3917710	1	169584521	C	12	33	T	0.0002456
rs3917690	1	169590911	C	12	33	T	0.0002456
rs3917687	1	169591093	G	12	33	A	0.0002456
rs3917678	1	169591895	A	12	33	C	0.0002456
rs17523783	1	169602241	T	12	33	G	0.0002456
rs72712029	1	169606412	T	12	33	C	0.0002456

rs72712030	1	169606590	A	12	33	C	0.0002456
rs369654643	1	169610477	A	12	33	ATATAAG	0.0002456
rs17589589	1	169610886	G	12	33	A	0.0002456
rs17589700	1	169614949	A	12	33	G	0.0002456
rs531650	1	170657994	C	16	37	T	0.0003171
rs7884276	1	171132644	C	11	0	G	0.0007274
rs74970368	1	171134688	C	11	0	G	0.0007274
rs7887919	1	171137772	A	11	0	C	0.0007274
rs74122249	1	171138389	C	11	0	T	0.0007274
rs76506870	1	171138483	A	11	0	G	0.0007274
rs79631707	1	171141237	T	11	0	C	0.0007274
rs77720244	1	171146081	C	11	0	T	0.0007274
rs79906093	1	171148864	A	11	0	T	0.0007274
rs75643254	1	171149489	T	11	0	C	0.0007274
rs6677549	1	171150840	C	11	0	T	0.0007274
rs6700357	1	171151193	T	11	0	G	0.0007086
rs28369789	1	171153577	G	11	0	A	0.0007274
rs76414019	1	171154643	A	11	0	AG	0.0007274
rs2020870	1	171154959	G	11	0	A	0.0007274
rs28369802	1	171156034	C	11	0	T	0.0007274
rs28369803	1	171156100	G	11	0	A	0.0007274
rs28369818	1	171158008	A	11	0	T	0.0007274
rs7526747	1	175071061	G	10	29	A	0.0002108
rs7526749	1	175071068	T	10	29	A	0.0002108
rs12088035	1	175071568	T	11	29	C	0.0004994
rs6678745	1	175072628	C	11	29	A	0.0004994
rs11584067	1	175075286	C	11	29	T	0.0004994
rs7552526	1	175075759	T	11	29	G	0.0004994
rs7552543	1	175075826	A	11	29	G	0.0004994
rs78664997	1	175165977	T	0	10	C	0.0005917
rs4549996	1	186707043	C	7	28	G	0.00005252
rs9442053	1	188643703	A	22	5	G	0.0006676
rs4650772	1	188649529	C	22	5	T	0.0006676
rs1902894	1	188650432	T	22	5	A	0.0006676
rs1902890	1	188650940	A	22	5	C	0.0006676
rs1902889	1	188651089	A	22	5	G	0.0006676
rs4651477	1	188651853	T	22	5	C	0.0006676
rs28659919	1	188652869	A	22	5	G	0.0006676
rs28882399	1	188655501	A	22	5	C	0.0006676
rs28407051	1	188657486	A	22	5	G	0.0006676

rs1870284	1	188658760	A	22	5	C	0.0006676
rs61832752	1	188659299	G	22	5	A	0.0006676
rs28716667	1	188659554	C	28	7	T	0.0001348
rs28470533	1	188663320	C	22	5	T	0.0006676
rs4628463	1	188663907	T	22	5	C	0.0006676
rs1902895	1	188664093	A	22	5	G	0.0006676
rs2125282	1	188664538	T	22	5	C	0.0006676
rs3076948	1	188664700	C	22	5	CTTTGA	0.0006676
rs2125283	1	188664796	A	22	5	C	0.0006676
rs12125711	1	188665560	G	22	5	C	0.0006676
rs7539954	1	188665816	C	22	5	A	0.0006676
rs28819458	1	188666185	G	22	5	T	0.0006676
rs7550119	1	188666925	T	22	5	C	0.0006676
rs7545804	1	188667358	C	22	5	T	0.0006676
rs4412594	1	188667533	G	22	5	A	0.0006676
rs76858875	1	188667613	T	20	4	G	0.0007481
rs2312277	1	188668304	T	22	5	C	0.0006676
rs2312278	1	188668379	C	22	5	T	0.0006676
rs1902877	1	188668620	A	22	5	C	0.0006676
rs1902876	1	188668668	T	22	5	C	0.0006676
rs10801018	1	188669367	A	22	5	T	0.0006676
rs10801020	1	188669435	T	22	5	G	0.0006676
rs7514277	1	188669462	T	22	5	C	0.0006676
rs4575089	1	188669624	T	22	5	C	0.0006676
rs4459111	1	188669689	C	22	5	T	0.0006676
rs4639766	1	188669859	T	22	5	A	0.0006676
rs61270430	1	188670298	CTT	22	5	C	0.0006676
rs28879483	1	188670518	T	22	5	C	0.0006676
rs28729428	1	188670708	A	22	5	G	0.0006676
rs12131857	1	188671055	G	22	5	C	0.0006676
rs12119328	1	188671215	C	22	5	T	0.0006676
rs10920924	1	188671252	A	22	5	G	0.0006676
rs7533774	1	188671315	G	22	5	C	0.0006676
rs7533904	1	188671432	G	22	5	T	0.0006676
rs7533919	1	188671462	G	22	5	A	0.0006676
rs34304001	1	188671467	A	22	5	G	0.0006676
rs7526057	1	188671469	A	22	5	C	0.0006676
rs7513673	1	188671524	C	22	5	T	0.0006676
rs6428054	1	188671656	A	22	5	T	0.0006676
rs6428055	1	188671850	A	22	5	G	0.0006676

rs6428056	1	188672106	T	22	5	A	0.0006676
rs4397657	1	188672537	G	22	5	A	0.0006676
rs4480360	1	188672581	G	22	5	C	0.0006676
rs4483411	1	188672658	G	22	5	A	0.0006676
rs4651480	1	188672838	C	22	5	T	0.0006676
rs4651481	1	188672913	T	22	5	C	0.0006676
rs28853365	1	188673023	G	22	5	A	0.0006676
rs4651482	1	188673359	T	22	5	C	0.0006676
rs28884294	1	188673361	T	22	5	C	0.0006676
rs2100666	1	188673445	C	22	5	G	0.0006676
rs2100667	1	188673494	G	22	5	A	0.0006676
rs4434839	1	188673548	A	22	5	G	0.0006676
rs4650773	1	188674062	T	22	5	G	0.0006676
rs4650774	1	188674113	G	22	5	A	0.0006676
rs4247158	1	188674188	T	22	5	C	0.0006676
rs28801114	1	188674205	G	22	5	T	0.0006676
rs4650775	1	188674220	T	22	5	A	0.0006676
rs6668661	1	188674687	C	22	5	A	0.0006676
rs57037772	1	188674715	GAAAA	22	5	G	0.0007368
rs4481852	1	188674740	C	22	5	A	0.0006676
rs4492623	1	188675005	G	22	5	A	0.0006676
rs4456095	1	188675152	C	22	5	T	0.0006676
rs7554587	1	188675544	C	22	5	A	0.0006676
rs4638130	1	188676018	T	22	5	G	0.0006676
rs370377948	1	188676257	A	22	5	ATTT	0.0006676
rs12120770	1	188676306	A	22	5	G	0.0006676
rs10921023	1	188676369	A	22	5	G	0.0006676
rs10921026	1	188676538	A	22	5	C	0.0006676
rs10921033	1	188676735	G	22	5	T	0.0006676
rs12123567	1	188676897	A	22	5	G	0.0006676
rs12120953	1	188676908	A	22	5	G	0.0006676
rs10921037	1	188676938	C	22	5	G	0.0006676
rs4474247	1	188677169	C	22	5	T	0.0006676
rs4396125	1	188677486	A	22	5	G	0.0006676
rs10921058	1	188678525	A	22	5	C	0.0006676
rs34085431	1	188679244	C	22	5	T	0.0006676
rs10801134	1	188684394	A	28	8	G	0.0003544
rs12028712	1	188686273	G	28	8	T	0.0003544
rs10436899	1	188693998	T	28	8	A	0.0003544
rs11801703	1	189825109	T	16	35	C	0.000956

1:198804284	1	198804284	A	15	38	AG	0.00007582
1:198804286	1	198804286	A	15	39	G	0.00004037
1:198804287	1	198804287	A	15	38	AAGGAAGGAAGGAAGGAAGG	0.00007582
rs12049427	1	199580285	G	27	8	A	0.0006326
rs113906106	1	200670819	T	11	0	C	0.0007274
rs2281845	1	201081943	T	47	22	C	0.0004013
rs3738262	1	201083343	G	47	23	A	0.0007443
rs3738263	1	201083506	G	47	23	A	0.0007443
rs7516023	1	201085521	T	47	23	C	0.0007443
rs6413915	1	201091041	A	45	20	C	0.000332
rs6704363	1	201096383	G	45	20	A	0.000332
rs7549337	1	201097609	G	45	19	T	0.0001682
rs200517518	1	201100129	A	41	17	AT	0.0004047
rs200108640	1	201100133	A	41	17	T	0.0004047
rs831762	1	201153168	T	3	17	G	0.0006426
rs61136828	1	202167795	G	23	5	GGTTTTTTTTTTT	0.0004042
rs12033091	1	202563966	T	12	0	C	0.0003422
rs12033827	1	202564040	A	12	0	C	0.0003422
rs375004536	1	202565747	A	12	0	AAAAC	0.0003422
rs629227	1	202570360	G	12	0	C	0.0003422
rs10800842	1	202570776	A	12	0	G	0.0003422
rs12037647	1	202573406	T	11	0	C	0.0007274
rs199940288	1	203750337	A	13	0	AAAG	0.0001796
rs34618698	1	204056948	A	18	3	G	0.0008121
rs10900600	1	204559707	G	14	33	T	0.0007122
rs10900601	1	204572050	A	13	32	G	0.0009401
rs4951082	1	204575807	A	56	31	G	0.0006352
rs898387	1	204579373	G	13	32	A	0.0009401
rs898386	1	204579697	A	13	32	G	0.0009401
rs4951083	1	204582730	A	13	32	G	0.0009401
rs884108	1	204591237	A	7	25	G	0.0003419
rs7551222	1	204599295	G	14	33	A	0.0007122
rs7553683	1	204599461	G	14	33	A	0.0007122
rs12024827	1	204607874	T	24	45	C	0.0007128
rs4951085	1	204608284	C	24	45	G	0.0007128
rs60168297	1	204608585	AC	20	40	A	0.0008835
rs4951086	1	204608643	T	17	40	C	0.0001139
rs4951087	1	204609194	A	24	45	G	0.0007128
rs4950954	1	204610475	G	24	45	A	0.0007128
rs4951088	1	204610780	T	24	45	C	0.0007128

rs4951089	1	204610898	T	24	45	C	0.0007128
rs138881554	1	204611658	C	24	45	CAAGGGAGGG	0.0007128
rs11240234	1	204612138	T	24	45	C	0.0007128
rs11240235	1	204612200	T	24	45	C	0.0007128
rs11240236	1	204612463	T	24	45	G	0.0007128
rs11240237	1	204612518	A	24	45	G	0.0007128
rs11240238	1	204612537	T	24	45	C	0.0007128
rs11240239	1	204612804	A	24	45	G	0.0007128
rs4279904	1	204613764	A	18	43	G	0.00003832
rs10900418	1	204614166	T	18	43	C	0.00003832
rs2772227	1	204615400	T	16	39	A	0.00009784
rs1329631	1	204616390	G	21	43	A	0.0003256
rs2815832	1	204617240	A	19	40	G	0.0004646
rs12127178	1	204620354	A	24	45	G	0.0007128
rs2772244	1	204621178	G	17	42	A	0.00003305
rs2492985	1	204621247	A	18	42	G	0.00007147
rs2480468	1	204621329	A	21	42	G	0.0005665
rs2480467	1	204624957	A	20	41	G	0.0005156
rs2451696	1	210282346	T	35	13	G	0.0006539
rs2451697	1	210284025	G	35	13	A	0.0006539
rs2484033	1	210287837	A	35	13	G	0.0006539
rs2484030	1	210296910	G	35	13	T	0.0006539
rs2484031	1	210303614	C	35	13	A	0.0006539
rs61848993	1	211095666	T	45	20	C	0.000305
rs150857619	1	212141784	A	0	10	C	0.0005917
rs139943545	1	212197700	G	0	10	T	0.0005917
rs200877944	1	212813000	A	53	27	G	0.0005244
rs1933642	1	218355332	T	22	5	A	0.0006676
rs12067336	1	218357940	T	22	5	C	0.0006676
rs12116939	1	218362082	C	22	5	T	0.0006676
rs57080194	1	218368208	A	22	5	G	0.0006676
rs59489398	1	218368361	T	22	5	C	0.0006676
rs1890357	1	218369926	T	25	6	A	0.0003059
rs12118865	1	218370252	T	22	5	A	0.0006676
rs4846265	1	218372593	T	22	5	C	0.0006676
rs146197820	1	220565631	TC	46	21	T	0.0003668
1:223087095	1	223087095	G	0	10	A	0.0005126
rs56403019	1	224762915	A	7	24	G	0.0006277
rs200807437	1	224773179	CT	6	22	C	0.0007426
rs904506	1	224779653	C	7	24	T	0.0006277

rs6698799	1	224806555	G	7	24	A	0.0006277
rs880496	1	224808134	T	7	24	G	0.0006277
rs6672413	1	224817129	C	7	25	T	0.0003419
rs6672806	1	224817506	A	7	25	T	0.0003419
rs56039603	1	224818840	A	8	26	G	0.0004835
rs58715712	1	224834621	G	4	21	A	0.0001601
rs59962884	1	224835339	A	4	21	G	0.0001601
rs17513562	1	224835372	C	4	21	T	0.0001601
1:228614740	1	228614740	C	44	21	CT	0.0005409
1:228614744	1	228614744	C	44	21	CTCCTTCCCTCCCTTCCTTTCTCC	0.0005409
rs34776739	1	232637687	C	37	15	CT	0.0009053
rs6674092	1	232638409	A	37	15	G	0.0009053
rs6694142	1	232638547	C	37	15	T	0.0009053
rs6676856	1	232638674	A	37	15	G	0.0009053
rs6686543	1	232638703	T	37	15	C	0.0009053
rs720806	1	232639262	A	37	15	T	0.0009053
rs6659527	1	234503848	G	29	50	A	0.0009027
rs12568273	1	234506616	G	18	41	A	0.0001308
rs45577939	1	234509865	G	25	48	T	0.000423
rs4300255	1	234510262	G	26	47	A	0.0007993
rs6586385	1	234511213	C	28	50	G	0.0005077
rs7554965	1	234511541	A	18	43	G	0.00003832
rs11285466	1	234512010	T	18	43	TC	0.00003832
rs6671513	1	234512169	G	18	43	A	0.00003832
rs10429821	1	234512471	C	28	49	T	0.0008723
rs10429822	1	234512519	C	28	50	T	0.0005077
rs10429823	1	234512583	C	18	43	T	0.00003832
rs10429824	1	234512593	T	28	50	A	0.0005077
rs7513305	1	234512685	C	28	50	G	0.0005077
rs10752783	1	234513512	G	28	50	C	0.0005077
rs10752784	1	234513707	G	28	50	A	0.0005077
rs2752	1	234513871	A	28	50	C	0.0005077
rs6681436	1	234514025	C	18	43	T	0.00003832
rs6664462	1	234514485	T	18	43	G	0.00003832
rs10797537	1	234514613	T	15	41	G	0.0000218
rs10797538	1	234514672	A	15	39	G	0.00001565
rs12024011	1	234514725	A	15	39	G	0.00003647
rs6681034	1	234515135	A	28	50	G	0.0005077
rs55884696	1	234515265	T	18	43	C	0.00003832
rs6684927	1	234516617	A	18	43	G	0.00003832

rs4477318	1	234517227	T	18	43	A	0.00003832
rs12401853	1	234517474	C	18	44	A	0.00002016
rs10910423	1	234517594	G	28	50	A	0.0005077
rs149002825	1	234518776	AGAGAAG	12	40	A	0.000001142
rs10797539	1	234519385	C	18	43	T	0.00003832
rs60111174	1	234520784	ACTT	18	43	A	0.00003832
rs10910424	1	234521024	T	18	43	C	0.00003832
rs10910425	1	234521118	C	18	43	T	0.00003832
rs10752785	1	234521718	T	25	47	G	0.0004398
rs7527165	1	234524045	T	18	43	C	0.00003832
rs12023971	1	234525333	A	18	43	G	0.00003832
rs6669190	1	234525940	C	30	56	T	0.00004926
rs12402835	1	234526732	C	18	43	G	0.00003832
rs7524822	1	234526868	T	54	29	C	0.0006016
rs3862040	1	234527575	T	28	52	C	0.0001612
rs3754318	1	234527992	A	54	29	G	0.0006016
rs3754317	1	234528006	A	54	29	G	0.0006016
rs7523423	1	234528373	T	28	52	C	0.0001612
rs2175594	1	234529552	T	27	53	C	0.00004506
rs2175593	1	234529570	A	27	52	G	0.00008446
rs10910426	1	234530325	G	28	52	A	0.0001612
rs10797540	1	234530370	A	28	52	G	0.0001612
rs4027040	1	234531009	A	54	29	G	0.0006016
rs61825875	1	234538524	C	10	28	G	0.000853
rs10910427	1	234539157	T	25	54	C	0.000005411
rs3768288	1	234540547	T	52	27	C	0.0005555
rs1141264	1	234546245	T	25	47	C	0.0004398
rs56945246	1	234729204	C	3	18	T	0.0003291
rs72767248	1	234729970	G	3	18	T	0.0003291
rs56056213	1	234730150	T	3	17	C	0.0006426
rs72749953	1	234732072	T	3	18	C	0.0003291
rs72749954	1	234732892	C	3	18	A	0.0003291
rs72749956	1	234733215	G	3	18	A	0.0003291
rs72749957	1	234733397	G	3	18	A	0.0003291
rs147711727	1	234735006	GC	3	18	G	0.0003291
1:234746921	1	234746921	C	5	21	G	0.0005184
rs5782030	1	238568643	T	43	20	TA	0.0004933
rs12074023	1	240631508	A	0	12	G	0.0001421
1:248659099	1	248659099	T	1	13	C	0.0004468
rs77038252	2	1579459	C	15	1	A	0.0003131

rs10929629	2	5666520	C	20	4	T	0.0007606
rs41439744	2	5669317	G	20	4	T	0.0007606
rs2882275	2	5689487	T	19	41	C	0.0002649
rs1561208	2	5689697	A	19	41	G	0.0002649
rs13397969	2	5692633	A	19	41	G	0.0002649
rs112423392	2	5694887	A	16	2	AC	0.0007845
rs1622254	2	7130521	G	2	16	A	0.0003502
rs59730520	2	8739217	T	1	16	TATG	0.00005445
rs72784647	2	8745931	T	1	16	C	0.00005445
rs66932920	2	8747957	C	1	16	T	0.00005445
rs201252070	2	8748478	A	1	13	AC	0.0003973
rs10803714	2	10163328	T	42	19	C	0.0008809
rs62113307	2	11886952	C	2	15	G	0.0006952
rs80119710	2	13181358	A	20	4	G	0.0007606
rs112051872	2	13186061	C	20	4	A	0.0007606
rs12477177	2	13209268	T	20	4	C	0.0007606
rs76680758	2	13224171	T	20	4	C	0.0007606
rs72771713	2	13487530	G	3	19	C	0.0001662
rs66615117	2	14398590	T	0	10	G	0.0005917
rs10715429	2	15843396	A	28	9	G	0.0008484
rs7566672	2	15843584	T	28	9	A	0.0008484
rs58206246	2	15847021	TA	29	9	T	0.0004831
rs58982282	2	21239855	CAAA	3	18	C	0.0003675
rs2593484	2	30943137	T	54	29	C	0.0006016
rs13385162	2	31724929	T	19	39	C	0.0007998
rs11684698	2	31727524	A	18	39	T	0.0004141
rs4952218	2	31731120	A	19	39	T	0.0007998
rs6543630	2	31740034	A	17	38	G	0.0003648
rs4952197	2	31767131	A	16	35	G	0.000956
rs58811103	2	31801180	G	19	40	C	0.0004646
rs2754530	2	31803403	T	19	41	C	0.0002649
rs2268799	2	31803495	T	19	41	C	0.0002649
rs522638	2	31805675	A	19	39	G	0.0007998
rs632148	2	31806031	C	19	39	G	0.0007998
rs806645	2	31807940	T	19	41	C	0.0002649
rs11403363	2	31807998	A	19	40	AT	0.0004646
rs676033	2	31808970	T	19	39	C	0.0007998
rs502139	2	31811268	T	19	40	C	0.0004646
rs599300	2	31811306	G	22	43	A	0.0006167
rs499362	2	31811572	A	22	43	C	0.0006167

rs633376	2	31815191	T	16	38	G	0.0001777
rs809364	2	31815453	A	16	38	G	0.0001777
rs646674	2	31815844	A	16	36	G	0.0005556
rs806646	2	31816197	A	12	33	C	0.0002456
rs1090846	2	31816341	T	15	38	C	0.00008268
rs600015	2	31820300	G	20	41	C	0.0005156
rs655548	2	31836658	A	20	41	C	0.0005156
rs477517	2	31848637	C	19	39	T	0.0007998
rs546935	2	31853674	A	20	41	G	0.0005156
rs35826411	2	31859226	C	18	39	CA	0.0004141
rs71446010	2	31859714	C	2	17	T	0.0001534
rs481344	2	31859919	A	18	39	G	0.0004141
rs681482	2	31871256	A	19	41	G	0.0002649
rs589427	2	31872513	A	19	39	T	0.0007998
rs1580329	2	32016677	G	20	41	A	0.0005156
rs223675	2	32026249	A	15	37	G	0.0001512
rs10779914	2	32106040	A	17	37	C	0.0006349
2:32194661	2	32194661	G	23	6	T	0.0009286
rs12478134	2	32240783	G	15	34	C	0.0008313
rs2280968	2	32290040	T	16	36	G	0.0005556
rs13017027	2	32291903	T	13	33	C	0.0005413
rs7556942	2	32295694	A	16	36	G	0.0005556
rs17011801	2	32298072	T	16	36	A	0.0005556
rs35112079	2	32302079	A	16	36	G	0.0005556
rs11695080	2	32316886	C	11	30	G	0.0006171
rs7557034	2	32316910	A	13	32	T	0.0009401
rs7569264	2	32316921	C	13	32	A	0.0009401
rs3769605	2	32322456	G	16	37	A	0.0003171
rs200363219	2	32327754	A	16	38	G	0.0001777
rs6543648	2	32330314	A	16	36	G	0.0005556
rs11676456	2	32331511	G	13	33	A	0.0005413
rs6756085	2	32336522	G	16	36	A	0.0005556
rs11691939	2	32339561	T	16	36	C	0.0005556
rs7574000	2	32342888	C	16	36	A	0.0005556
rs13006880	2	32355539	A	13	33	G	0.0005413
rs13013695	2	32391231	A	14	33	G	0.0007122
rs212682	2	32432864	A	17	36	G	0.0009486
rs212683	2	32432934	A	16	36	T	0.0005556
rs212702	2	32444250	A	14	33	G	0.0007122
rs212736	2	32473126	T	14	33	C	0.0007122

rs410469	2	32476992	T	16	36	G	0.0005556
rs7562653	2	32478629	T	16	36	C	0.0005556
rs805831	2	32484228	C	16	36	G	0.0005556
2:33053011	2	33053011	T	4	20	G	0.0003114
rs10209371	2	34499096	T	12	0	G	0.0003422
rs4670081	2	34502152	A	12	0	T	0.0003422
rs12467806	2	34505794	A	14	0	T	0.00007436
rs12471653	2	34505864	G	14	0	A	0.00007436
rs12464598	2	34506082	A	14	0	G	0.00007436
rs12472526	2	34506118	T	14	0	A	0.00007436
rs62161745	2	34507526	G	11	0	T	0.0007274
rs62161746	2	34507719	T	11	0	C	0.0007274
rs113919160	2	34508173	C	11	0	CA	0.0007274
rs60870684	2	34508506	T	11	0	G	0.0007274
rs62161748	2	34508621	A	11	0	G	0.0007274
rs72784834	2	34508878	A	11	0	G	0.0007274
rs72784835	2	34509004	A	11	0	G	0.0007274
rs7560487	2	34509206	T	12	0	C	0.0003422
rs2042116	2	34509330	T	12	0	C	0.0003422
rs12233151	2	34509401	T	12	0	A	0.0003422
rs62161749	2	34510104	T	12	0	A	0.0003422
rs62161750	2	34510193	T	12	0	C	0.0003422
rs60878936	2	34515125	T	12	0	G	0.0003422
rs7563223	2	34515235	T	11	0	C	0.0007274
rs62161754	2	34517349	C	12	0	T	0.0003422
2:34517480	2	34517480	G	12	0	C	0.0003567
rs73928244	2	34517528	T	12	0	C	0.0003422
rs72784842	2	34517583	G	12	0	A	0.0003422
rs62163160	2	34517873	T	12	0	C	0.0003422
rs6728480	2	34519448	T	12	0	C	0.0003422
rs4327215	2	34529017	C	12	0	T	0.0003422
rs60147661	2	34530231	C	12	0	T	0.0003422
rs57032414	2	34530485	C	12	0	A	0.0003422
rs305178	2	36365019	T	18	39	C	0.0004141
rs1397444	2	36365804	G	18	39	A	0.0004141
rs959734	2	36365806	G	18	39	A	0.0004141
rs305179	2	36366492	G	18	39	C	0.0004141
rs11903069	2	38558787	T	40	16	C	0.0006273
rs10177042	2	38563527	G	38	15	C	0.0005267
rs13020216	2	38592175	C	30	10	G	0.0006142

rs112517328	2	38596390	G	36	14	A	0.0007756
rs78494590	2	42151474	T	2	15	C	0.0006243
rs4952547	2	42157310	A	2	15	G	0.0006243
rs11888637	2	45517967	A	1	13	G	0.0005059
rs17036673	2	47788177	T	25	7	C	0.0008092
rs1922043	2	47794960	C	5	22	T	0.000274
rs66500421	2	50828081	AT	31	53	A	0.0005517
rs10186698	2	50835669	A	51	27	G	0.0009437
2:50835683	2	50835683	AGTTGC	31	53	A	0.0005517
2:50835684	2	50835684	A	31	53	AAAAT	0.0005517
rs372996983	2	52271700	T	14	37	C	0.0001176
rs34393418	2	52274004	C	19	40	G	0.0004646
rs4504033	2	52280819	G	19	40	C	0.0004646
2:60676657	2	60676657	C	8	26	T	0.0005183
rs115595309	2	67226030	A	14	1	C	0.0006347
rs62146967	2	68717683	G	0	11	A	0.0002715
rs113302059	2	68773407	C	0	10	T	0.0005917
rs6546916	2	74872278	A	0	10	C	0.0005917
2:76784102	2	76784102	G	12	31	GCAGGAGTTGGGCTGAGATTA[illegible]	0.0007989
rs10865433	2	78608101	A	33	55	G	0.0005652
2:80215593	2	80215593	T	12	0	TG	0.0003315
2:80215595	2	80215595	T	12	0	TG	0.0003422
2:81836822	2	81836822	G	21	4	A	0.0003768
rs112093957	2	81836835	T	21	4	C	0.0004069
rs113406922	2	81836843	C	21	4	T	0.0004069
rs7589987	2	83349542	G	0	10	C	0.0005917
rs112124898	2	84581600	A	0	13	G	0.00005596
rs77159567	2	84614874	A	0	13	T	0.00005596
rs11126962	2	84638116	C	0	13	A	0.00005596
2:84647251	2	84647251	T	0	10	TA	0.0005917
rs142296229	2	84672087	T	0	13	C	0.00005596
rs76990652	2	84676268	C	0	12	T	0.0001237
rs111878765	2	84714899	A	0	13	G	0.00005596
rs11687501	2	85009989	A	0	12	G	0.0001237
rs183611857	2	85056725	A	0	11	G	0.0002715
rs11686064	2	85063529	A	0	11	G	0.0002715
rs192817406	2	85232419	T	1	13	A	0.0004468
rs74200635	2	92085478	A	16	1	G	0.0001529
2:95489456	2	95489456	CA	0	10	C	0.0005917
rs11679277	2	104572488	G	3	17	A	0.0006426

rs72823440	2	112490418	A	32	12	G	0.0008741
rs369400020	2	112490536	C	29	10	G	0.0009257
rs199891994	2	112495314	T	32	11	TGAG	0.0004661
2:112508450	2	112508450	A	21	4	G	0.0004416
rs926894	2	114302987	A	32	55	G	0.0005264
rs13022237	2	116416128	G	9	31	A	0.00005656
rs13027654	2	116416368	G	9	31	A	0.00005656
rs7589670	2	116419064	C	9	27	T	0.000654
rs13022167	2	116419585	C	9	31	G	0.00005656
rs35366441	2	116421476	T	9	27	C	0.000654
rs34140584	2	116421875	G	9	27	A	0.000654
rs6734750	2	116422034	C	9	28	G	0.0003633
rs148563780	2	116434973	A	9	27	AAAATGATAGGAGT	0.000654
rs139435503	2	116435048	TA	9	27	T	0.000654
rs72828530	2	116444588	A	9	27	G	0.000654
rs4849823	2	120873745	T	0	10	A	0.0005917
rs149180687	2	120945130	A	0	11	G	0.0002715
rs113551833	2	120962553	A	0	11	G	0.0002715
rs3930389	2	130983388	G	36	57	A	0.0006352
2:133251079	2	133251079	A	48	24	AT	0.0007984
rs12993602	2	133348884	T	0	10	C	0.0005917
2:142514117	2	142514117	T	24	6	C	0.0005592
rs7560287	2	144093803	A	39	16	C	0.0006105
rs4972338	2	148974872	T	45	21	A	0.0006315
rs77388016	2	151733840	T	1	13	C	0.0005059
rs12151724	2	151734715	A	1	13	T	0.0005059
rs79308412	2	151737087	A	1	13	G	0.0005059
rs57209901	2	155227443	A	12	0	G	0.0003422
rs707058	2	155281517	C	11	0	G	0.0007274
rs707060	2	155281911	T	11	0	C	0.0007274
rs707061	2	155282560	A	11	0	G	0.0007274
rs150215636	2	155288342	G	11	0	GC	0.0007274
rs75739412	2	155289152	T	11	0	C	0.0007274
rs1405934	2	155294658	C	18	3	T	0.0008121
rs1527862	2	155294795	T	18	3	C	0.0008121
rs1527861	2	155294870	A	18	3	G	0.0008121
rs113055622	2	155298558	T	11	0	TTC	0.0007274
rs4140692	2	155298682	A	11	0	G	0.0007274
rs74986530	2	155308126	A	11	0	G	0.0007274
rs201005680	2	162141748	T	11	0	TATC	0.0007274

rs115432269	2	162184233	A	11	0	G	0.0007274
rs138541092	2	162252833	A	11	0	G	0.0007274
rs62187573	2	162378064	A	1	13	G	0.0005059
rs2021253	2	167864215	T	9	30	A	0.0001068
rs5836182	2	169137688	A	5	20	ACTT	0.0009659
rs4458184	2	169140909	A	5	20	C	0.0009659
rs73971596	2	169143262	A	2	15	C	0.0006243
rs377300030	2	169146356	T	2	15	TTTCAATTATTA	0.0006243
rs79898710	2	169156742	A	2	16	G	0.0003115
rs77343714	2	169160724	T	2	16	C	0.0003115
rs10172808	2	169163422	C	2	16	T	0.0003115
rs9646776	2	169165476	T	2	16	C	0.0003115
rs182471681	2	171688146	G	29	9	A	0.000837
rs10198915	2	172361623	T	2	15	C	0.0006243
rs7575777	2	178775157	C	1	13	A	0.0005059
rs62177379	2	185032159	C	1	13	G	0.0005059
rs62177381	2	185033003	T	1	13	C	0.0005059
rs2887422	2	185033177	A	1	13	C	0.0005059
rs62177383	2	185034151	T	1	13	C	0.0005059
rs62177384	2	185034415	A	1	13	G	0.0005059
rs62177385	2	185034736	A	1	13	T	0.0005059
rs62177386	2	185034972	A	1	13	G	0.0005059
rs62177387	2	185035801	A	1	13	T	0.0005059
rs62177389	2	185036793	G	1	13	T	0.0005059
rs72899135	2	185037027	G	1	13	A	0.0005059
rs62177390	2	185037166	T	1	13	A	0.0005059
rs76252472	2	185037434	C	1	13	T	0.0005059
rs62177391	2	185037945	C	1	13	T	0.0005059
rs62177392	2	185039059	C	1	13	T	0.0005059
rs17731073	2	185039398	G	1	13	A	0.0005059
rs1997131	2	185043362	A	1	14	G	0.0002433
rs62174972	2	185046363	T	1	13	C	0.0005059
rs143304053	2	185047784	T	1	13	TA	0.0005059
rs1526197	2	185050101	A	1	14	G	0.0002433
rs6434076	2	185052186	A	1	14	C	0.0002433
rs1526221	2	185053799	T	1	13	C	0.0005059
rs62174975	2	185054451	G	1	13	A	0.0005059
rs62174979	2	185063778	T	1	13	C	0.0005059
rs62174980	2	185065710	G	1	13	A	0.0005059
rs62174981	2	185069101	T	1	13	C	0.0005059

rs62174982	2	185072575	A	1	13	G	0.0005059
rs62174984	2	185073913	T	1	13	C	0.0005059
rs35785665	2	185074545	CAAAG	1	13	C	0.0005059
rs62174985	2	185075253	G	1	13	T	0.0005059
rs62174986	2	185075686	G	1	13	A	0.0005059
rs11898206	2	185076065	A	1	13	G	0.0005059
rs62174989	2	185078573	T	1	13	C	0.0005677
rs62174991	2	185079639	G	1	13	A	0.0005059
rs62175002	2	185080161	T	1	13	C	0.0005059
rs62175003	2	185080522	T	1	13	C	0.0005059
rs137891463	2	185081236	C	1	13	CTTG	0.0005059
rs62175004	2	185081554	G	1	13	T	0.0005059
rs6761905	2	185084032	A	1	13	G	0.0005059
rs62175006	2	185085379	A	1	13	T	0.0005059
rs6740051	2	185089218	G	1	13	T	0.0005059
rs62175008	2	185094412	A	1	13	G	0.0005059
rs62175009	2	185095250	A	1	13	G	0.0005059
rs62175010	2	185095398	A	1	13	T	0.0005059
rs199679700	2	185095737	G	1	13	GA	0.0005059
2:185095973	2	185095973	G	1	13	A	0.0005059
rs140916704	2	185097051	A	1	13	T	0.0005059
rs192589595	2	185097484	T	1	13	G	0.0005059
rs114033272	2	185098646	A	1	13	G	0.0005059
rs374539716	2	185098987	C	1	13	T	0.0005059
rs62175014	2	185099359	G	1	13	A	0.0005059
rs72901145	2	185101311	G	1	13	A	0.0005059
rs62175015	2	185102632	A	1	13	T	0.0005059
rs144666042	2	185106285	GT	1	13	G	0.0005059
rs7573114	2	185106611	G	1	13	A	0.0005059
rs72901158	2	185107454	T	1	13	C	0.0005059
rs62175018	2	185107950	T	1	13	C	0.0005059
rs146114313	2	185108358	T	1	13	G	0.0005059
rs75506748	2	185108659	T	1	13	C	0.0005059
rs145128486	2	185110879	C	1	13	G	0.0005059
rs114689984	2	185110884	A	1	13	G	0.0005059
rs62175039	2	185111974	G	1	13	A	0.0005059
rs145452825	2	185123754	A	1	13	AG	0.0005059
rs62175045	2	185127055	G	1	13	A	0.0005059
rs62175046	2	185128471	G	1	13	T	0.0005059
rs141835155	2	185130171	A	1	13	G	0.0005059

rs7583823	2	185131751	C	1	13	G	0.0005059
rs62175048	2	185132406	A	1	13	C	0.0005059
rs62175049	2	185133991	A	1	13	G	0.0005059
rs62175050	2	185134523	C	1	13	T	0.0005059
rs62175051	2	185134819	A	1	13	G	0.0005059
rs62175052	2	185139542	T	1	13	C	0.0005059
rs62175053	2	185141889	C	1	13	T	0.0005059
rs62175054	2	185142492	G	1	13	T	0.0005059
rs62175055	2	185142687	G	1	13	C	0.0005059
rs62175056	2	185142987	C	1	13	T	0.0005059
rs62175057	2	185142997	C	1	13	G	0.0005059
rs62175058	2	185143454	A	1	13	G	0.0005059
rs62175059	2	185144017	T	1	13	A	0.0005059
rs62176360	2	185144106	T	1	13	C	0.0005059
rs62176361	2	185144746	T	1	13	C	0.0005059
rs62176362	2	185145036	C	1	13	T	0.0005059
rs62176363	2	185145087	T	1	13	C	0.0005059
rs62176365	2	185146314	T	1	13	C	0.0005059
rs371708927	2	185147621	T	1	13	TA	0.0005059
rs114146281	2	185149046	C	1	13	T	0.0005059
rs62176368	2	185149266	C	1	13	G	0.0005059
rs369214888	2	185149958	A	1	13	ACTT	0.0005059
rs114288133	2	185150059	G	1	13	A	0.0005059
rs62176370	2	185151717	C	1	13	T	0.0005059
rs62176371	2	185151931	T	1	14	C	0.0002433
rs138276885	2	185152116	T	1	13	TG	0.0005059
rs62176372	2	185153440	A	1	13	T	0.0005059
rs62176373	2	185155330	T	1	13	C	0.0005059
rs62176374	2	185156242	G	1	13	T	0.0005059
rs62176375	2	185156417	C	1	13	T	0.0005059
rs62176376	2	185157153	C	1	13	A	0.0005059
rs72902844	2	185160201	C	1	12	T	0.0009301
rs62176377	2	185161614	T	1	13	C	0.0005059
rs78132165	2	185161691	CAGT	1	13	C	0.0005059
rs62176378	2	185162261	T	1	13	C	0.0005059
rs62176379	2	185162676	A	1	13	C	0.0005059
rs62176403	2	185162915	G	1	13	A	0.0005059
rs62176404	2	185163029	A	1	13	G	0.0005059
rs62176405	2	185164825	C	1	13	T	0.0005059
rs141925621	2	185165351	C	1	13	CTCTTT	0.0005059

rs62176406	2	185165485	G	1	13	T	0.0005059
rs78692314	2	185165873	C	1	13	CAAAA	0.0005059
rs76479285	2	185166232	G	1	13	A	0.0005059
rs72902855	2	185166276	T	1	13	C	0.0005059
rs62176407	2	185166354	T	1	13	C	0.0005059
rs62176408	2	185167352	T	1	13	A	0.0005059
rs372320639	2	185167618	G	1	13	GA	0.0005059
rs72902858	2	185167658	T	1	13	A	0.0005059
rs72902859	2	185167981	A	1	13	G	0.0005059
rs62176409	2	185168320	T	1	13	C	0.0005059
rs62176410	2	185169634	G	1	13	A	0.0005059
rs62176411	2	185169886	G	1	13	A	0.0005059
rs72902865	2	185169986	G	1	13	A	0.0005059
rs75542286	2	185170297	C	1	13	CAT	0.0005059
rs149343308	2	185170458	T	1	13	TGC	0.0005059
rs62176412	2	185170949	C	1	13	A	0.0005059
rs72902870	2	185171140	G	1	13	C	0.0005059
rs62176413	2	185172097	A	1	13	G	0.0005059
rs62176414	2	185172128	C	1	13	T	0.0005059
rs62176415	2	185172414	A	1	13	G	0.0005059
rs142990632	2	185172860	A	1	13	AG	0.0005059
rs62176416	2	185174164	G	1	13	A	0.0005059
rs145121164	2	185174492	G	1	13	GTA	0.0005059
rs72902879	2	185174679	C	1	13	T	0.0005059
rs62176417	2	185174954	A	1	13	C	0.0005059
rs62176418	2	185175198	A	1	13	C	0.0005059
rs62176419	2	185175735	G	1	13	A	0.0005059
rs62176420	2	185175888	G	1	13	A	0.0005059
rs62176421	2	185176240	G	1	13	T	0.0005059
rs62176422	2	185176824	A	1	13	G	0.0005059
rs62176423	2	185176938	T	1	13	A	0.0005059
rs62176424	2	185177445	G	1	13	T	0.0005059
rs72902884	2	185177666	C	1	13	G	0.0005059
rs72902886	2	185177667	T	1	13	C	0.0005059
rs62176425	2	185178438	T	1	13	C	0.0005059
rs62176426	2	185178614	G	1	13	A	0.0005059
rs62176427	2	185178930	G	1	13	C	0.0005059
rs72902891	2	185179144	G	1	13	A	0.0005059
rs62176428	2	185179190	G	1	13	A	0.0005059
rs62176430	2	185179619	A	1	13	C	0.0005059

rs62176431	2	185179701	G	1	13	A	0.0005059
rs62176434	2	185180247	C	1	13	T	0.0005059
rs72902897	2	185181063	G	1	13	A	0.0005059
rs62176436	2	185181923	T	1	13	C	0.0005059
rs62176437	2	185182064	A	1	13	G	0.0005059
rs147000256	2	185182163	G	1	13	GT	0.0005059
rs72902901	2	185182222	A	1	13	G	0.0005059
rs72904804	2	185182521	T	1	13	G	0.0005059
rs62176438	2	185182987	G	1	13	T	0.0005059
rs72904810	2	185183783	G	1	13	A	0.0005059
rs62176439	2	185184017	C	1	13	T	0.0005059
rs62176440	2	185184711	A	1	13	G	0.0005059
rs72904818	2	185185261	A	1	13	G	0.0005059
rs62176441	2	185187646	T	1	13	C	0.0005059
2:185187774	2	185187774	ATC	1	13	A	0.0005059
rs78434879	2	185187780	C	1	13	G	0.0005059
rs10177272	2	185189820	A	1	13	G	0.0004468
rs376234709	2	185196329	G	1	14	A	0.0002433
rs13023807	2	185295053	G	11	30	A	0.0006452
2:206745639	2	206745639	CAA	35	12	C	0.0001485
rs71422753	2	212603099	G	58	33	T	0.0009818
rs62183021	2	212611713	A	41	16	G	0.000199
rs74436564	2	212614240	T	41	16	TGG	0.000199
rs7601502	2	212617805	A	41	16	G	0.000199
rs72948506	2	212618440	A	41	17	G	0.0004047
rs62183023	2	212618746	T	40	17	C	0.0006981
rs62183024	2	212618836	T	41	17	C	0.0004047
rs61079605	2	212621761	T	41	17	A	0.0004047
rs7600261	2	212622818	T	41	17	C	0.0004047
rs55758468	2	212629822	A	42	17	G	0.0002305
rs62183028	2	212631483	T	42	18	G	0.0004599
rs62183029	2	212632452	A	42	18	G	0.0004599
rs55769536	2	212634084	T	43	17	A	0.000129
rs7583068	2	212634108	A	46	21	T	0.0003668
rs4132462	2	212635589	T	41	18	C	0.0007885
rs7576466	2	212635881	G	25	46	T	0.0007575
rs62183031	2	212636271	C	41	17	A	0.0004047
rs62183032	2	212638862	A	25	47	G	0.0004398
rs72948544	2	212667092	C	20	4	T	0.0007606
rs11677054	2	214951436	A	50	26	G	0.0008986

2:214973202	2	214973202	C	20	4	CA	0.0007481
rs35422889	2	219809760	T	9	29	C	0.0001986
rs13000521	2	228094994	A	22	5	G	0.0006676
rs11691091	2	228096921	A	22	5	G	0.0006676
rs12478772	2	228101249	A	22	5	T	0.0006676
rs12694723	2	228101905	G	22	5	A	0.0006676
rs10167041	2	228123661	G	54	28	A	0.0003346
rs4332918	2	228124702	G	53	28	A	0.00058
rs57569440	2	228125276	GA	54	28	G	0.0003346
rs6436670	2	228126494	C	19	48	T	0.000003024
rs4643535	2	228133809	G	21	43	A	0.0003256
rs4608502	2	228134155	T	21	43	C	0.0003256
rs7606754	2	228135180	A	21	43	G	0.0003256
rs143937055	2	228143966	T	21	43	TTTG	0.0003256
rs62279162	2	228162280	G	21	43	T	0.0003256
rs62279163	2	228168099	A	21	43	C	0.0003256
rs12471801	2	228171326	C	21	43	A	0.0003256
rs4305262	2	228174518	A	21	43	T	0.0003256
rs57817160	2	228179238	A	21	43	G	0.0003256
rs59257065	2	228179328	T	21	43	C	0.0003256
rs10173844	2	228185809	A	20	43	G	0.0001658
rs58670479	2	228192473	T	21	43	G	0.0003256
2:228196864	2	228196864	AT	21	43	A	0.0003256
rs62279170	2	228197701	G	20	43	C	0.0001658
rs6754433	2	228198773	G	21	43	A	0.0003256
rs62279171	2	228199759	T	21	43	C	0.0003256
rs62279172	2	228203142	A	21	42	G	0.0005665
rs61293565	2	228206567	G	21	43	A	0.0003256
rs62279174	2	228207842	C	21	43	T	0.0003256
rs6721179	2	228210499	G	21	43	A	0.0003256
rs56022659	2	228212798	C	20	43	G	0.0001658
rs59419007	2	228214716	G	20	43	A	0.0001658
rs12478005	2	228220528	C	20	43	T	0.0001658
rs7560053	2	228220769	T	20	43	C	0.0001658
rs8222	2	228222293	G	20	43	C	0.0001658
rs4675171	2	228225144	T	20	43	C	0.0001658
rs62189960	2	228619651	T	11	0	C	0.0007274
rs72969092	2	229017492	C	31	11	T	0.0008085
rs75339037	2	229022874	G	25	6	A	0.0003059
rs76762410	2	229022909	A	25	6	G	0.0003059

rs6722800	2	229023710	T	25	7	C	0.0008092
rs6723112	2	229023821	C	25	7	G	0.0008092
rs13400035	2	229024387	G	25	7	A	0.0008092
rs78117385	2	229025814	A	25	7	G	0.0008092
rs12479323	2	229026121	A	25	7	G	0.0008092
2:229026423	2	229026423	CAAAAT	25	7	C	0.0008092
rs61383612	2	229027437	A	25	7	G	0.0008092
rs59742648	2	229027581	AT	25	7	A	0.0008092
rs12465409	2	229027869	A	25	7	G	0.0008092
rs12465384	2	229027919	T	25	7	C	0.0008092
rs35867399	2	229028923	T	25	7	TA	0.0008092
rs4471882	2	229029290	A	25	7	T	0.0008092
rs4299327	2	229029307	T	25	7	C	0.0008092
rs4305256	2	229029318	C	25	7	T	0.0008092
rs4327223	2	229029615	C	25	7	T	0.0008092
rs4525684	2	229029654	C	25	7	T	0.0008092
rs13409234	2	229029823	C	25	7	T	0.0008092
rs13409651	2	229030197	G	25	7	T	0.0008092
rs79663725	2	229030283	T	25	7	C	0.0008092
rs13405515	2	229030704	G	25	7	A	0.0008092
rs13431126	2	229030793	T	25	7	C	0.0008092
rs13394274	2	229031301	C	25	7	T	0.0008092
rs13406252	2	229031435	G	25	7	A	0.0008092
rs13409017	2	229031613	T	25	7	A	0.0008092
rs13409131	2	229031700	G	25	7	A	0.0008092
rs12615268	2	229032048	C	25	7	T	0.0008092
rs12611873	2	229032110	T	25	7	C	0.0008092
rs12611925	2	229032333	T	25	7	C	0.0008092
rs13384573	2	229032373	A	25	7	G	0.0008092
rs5839255	2	229032907	AT	25	7	A	0.0008092
rs4337464	2	229033111	G	25	7	A	0.0008092
rs4341920	2	229033188	G	25	7	A	0.0008092
rs4616469	2	229033372	C	25	7	T	0.0008092
rs13413606	2	229033525	C	25	7	A	0.0008092
rs4350738	2	229033739	T	25	7	C	0.0008092
rs375788592	2	229033789	G	25	7	GAAGAA	0.0008092
rs4574108	2	229033814	A	25	7	G	0.0008092
rs4344926	2	229033990	T	25	7	A	0.0008092
rs10173045	2	229034108	G	25	7	C	0.0008092
rs10196700	2	229034233	G	25	7	A	0.0008092

rs77131468	2	229034349	C	25	7	G	0.0008092
rs28814657	2	229034629	T	25	7	G	0.0008092
rs4630759	2	229035357	A	25	7	T	0.0008092
rs4334483	2	229035442	T	25	7	A	0.0008092
rs4364010	2	229035509	T	25	7	C	0.0008092
rs10179300	2	229035717	T	25	7	C	0.0008092
rs10179408	2	229035869	A	25	7	C	0.0008092
rs10203311	2	229036257	G	25	7	A	0.0008092
rs10203410	2	229036304	G	25	7	A	0.0008092
rs9653368	2	229036780	A	25	7	G	0.0008092
rs13415645	2	229037167	C	25	7	T	0.0008092
rs13402484	2	229037409	T	25	7	C	0.0008092
rs13418687	2	229037643	C	25	7	T	0.0008092
rs139083559	2	229037654	AAGG	25	7	A	0.0008092
rs13405360	2	229037779	T	25	7	C	0.0008092
rs10188661	2	229043855	T	25	7	G	0.0008092
rs6739369	2	229912852	C	11	0	A	0.0007274
rs6748136	2	229915436	T	11	0	A	0.0007274
rs10048696	2	229916288	C	11	0	A	0.0007274
rs10176362	2	229917796	A	11	0	T	0.0007274
rs16825626	2	229918641	C	11	0	T	0.0007274
rs61410848	2	229918816	AG	11	0	A	0.0007274
rs10490033	2	229918898	A	11	0	G	0.0007274
rs60550348	2	229919102	T	11	0	C	0.0007274
rs7599580	2	229919490	C	11	0	T	0.0007274
rs11896929	2	229919667	G	11	0	A	0.0007274
rs35907947	2	230495698	TG	29	50	T	0.0009027
rs13006528	2	230498419	G	29	50	A	0.0009027
rs2007965	2	230508050	A	26	50	G	0.0001475
rs34056484	2	230508670	C	27	50	T	0.0002777
rs35596599	2	230508778	C	27	50	T	0.0002777
rs34851081	2	230508800	C	27	50	T	0.0002777
rs11676294	2	230517972	T	19	40	G	0.0004646
2:230683757	2	230683757	C	30	6	CACACA	0.00002435
rs201762374	2	233200229	G	27	7	C	0.0002486
rs115624040	2	233234727	A	0	11	C	0.0002715
rs582082	2	234932954	T	51	26	G	0.0005284
rs149600429	2	239768441	T	16	2	C	0.0007845
rs139338240	2	239768515	T	16	2	C	0.0007845
rs2019849	2	241048221	C	56	23	T	0.000002389

rs4854030	2	241049147	A	30	51	G	0.0009286
rs4854029	2	241049148	A	30	51	G	0.0009286
rs6740952	2	241049626	A	30	51	G	0.0009286
rs10200500	2	241049977	C	30	51	T	0.0009286
rs10191379	2	241050051	G	29	51	T	0.0005253
2:241050226	2	241050226	TCCTCCTCCCCTCCTCCTCTCT	30	50	T	0.0008697
rs13414100	2	241050480	G	30	51	A	0.0009286
rs7572067	2	241051245	C	30	51	T	0.0009286
rs6721817	2	241051464	C	29	51	T	0.0005253
rs12478533	2	241250620	T	54	29	G	0.0006016
rs12478193	2	241252539	C	54	29	T	0.0006016
rs6748055	2	241262641	A	55	30	G	0.0006201
rs2675296	3	2750753	C	42	19	T	0.0008809
rs9870347	3	3429642	T	30	10	C	0.0006347
rs9831715	3	3429710	A	27	8	G	0.0006326
rs9818655	3	3429751	A	30	10	T	0.0006347
rs9814703	3	3429755	G	30	10	A	0.0006347
rs11915570	3	3429941	T	30	10	A	0.0006347
rs6771545	3	3457052	T	31	11	C	0.0008085
rs7636780	3	3474111	C	28	9	T	0.0008484
rs6786006	3	3481956	T	28	9	C	0.0008484
rs1396415	3	3496754	A	25	7	G	0.0008092
rs142935847	3	4584368	G	3	17	GGT	0.0007114
rs4685951	3	5631147	A	27	8	G	0.0006326
rs17658611	3	6401765	T	28	49	C	0.0008723
rs2217845	3	6406809	A	28	49	G	0.0008723
rs9820568	3	7083544	A	12	0	C	0.0003422
rs71055876	3	11493487	C	12	31	CTTGGGGGG	0.0007705
rs2442772	3	11493926	A	12	32	G	0.0004387
rs2454505	3	11497325	C	12	32	T	0.0004387
rs2454496	3	11502552	T	12	31	G	0.0007705
rs884466	3	13142274	C	29	10	T	0.0009257
rs12495985	3	13142638	C	23	5	T	0.0003619
rs35220159	3	13631296	G	7	24	A	0.0006277
rs62232966	3	13640852	A	7	24	C	0.0006277
3:15506747	3	15506747	ATTTATTTATTTATTTATTTAT	16	0	A	0.00001575
rs150826285	3	26036390	A	0	10	C	0.0005917
rs140189968	3	26036393	G	0	10	C	0.0005917
rs76666691	3	26048549	T	0	10	C	0.0005917
rs75969402	3	26119136	A	0	10	G	0.0005917

rs2290533	3	31789724	T	52	28	C	0.0009847
rs4955112	3	31791851	T	52	28	G	0.0009847
rs78884871	3	32239143	A	8	25	G	0.000871
rs342539	3	42510528	A	6	23	G	0.0004533
rs34544831	3	43204651	C	0	10	CTT	0.0005917
rs2234358	3	45989044	T	33	54	G	0.0009764
rs1994489	3	45993226	G	33	54	C	0.0009764
rs4682799	3	45993606	T	33	54	C	0.0009764
rs9311380	3	45996047	T	33	54	C	0.0009764
rs6441936	3	45997822	A	33	54	T	0.0009764
rs4682800	3	46002005	A	33	54	C	0.0009764
rs1601867	3	46002962	T	34	55	C	0.0009818
rs3733100	3	46009864	C	30	51	G	0.0009286
3:53554931	3	53554931	C	4	18	CT	0.0009855
rs979220	3	53556223	C	34	11	T	0.0001478
rs12629517	3	53561686	G	57	32	A	0.0006468
rs35555534	3	53562519	C	25	6	T	0.0003059
rs6777794	3	53562586	G	57	32	T	0.0006468
rs6777870	3	53562602	C	57	32	T	0.0006468
rs35306382	3	53579944	A	26	7	C	0.0004518
rs13100574	3	53596184	G	26	7	A	0.0004518
rs61158509	3	60128998	T	8	25	C	0.000871
rs35022605	3	60130882	A	14	33	AT	0.0007122
rs62240253	3	60132040	T	14	33	C	0.0007122
rs12632865	3	60132390	T	14	33	C	0.0007122
rs785159	3	62296870	G	8	25	T	0.000871
rs2371863	3	65099625	A	43	19	G	0.0005165
rs13078431	3	65103351	A	44	20	G	0.0005739
rs17403692	3	65104083	T	44	20	C	0.0005739
rs150930606	3	65108257	T	44	20	C	0.0005739
rs35642529	3	65108915	GA	44	20	G	0.0005739
rs28880106	3	65109051	T	44	20	C	0.0005739
rs148294188	3	65109486	C	44	20	G	0.0005739
3:65110228	3	65110228	C	43	20	T	0.0009741
rs36159406	3	65110301	C	44	20	A	0.0005739
rs13093312	3	65110712	G	44	20	A	0.0005739
rs13100314	3	65112021	A	44	20	G	0.0005739
rs13100221	3	65112138	G	44	20	C	0.0005739
rs1482607	3	65113730	C	44	20	T	0.0005739
rs2012632	3	65119822	T	44	20	C	0.0005739

rs7636917	3	65123765	G	31	54	A	0.0003134
rs35471759	3	65184649	A	42	19	T	0.0008809
rs4855329	3	68353494	T	8	25	C	0.000871
rs4345072	3	68353956	C	7	25	A	0.0003419
rs6549115	3	68355872	A	7	25	T	0.0003419
rs9822608	3	68357852	T	7	25	G	0.0003419
rs57935782	3	68360456	T	7	25	TGTCATATA	0.0003419
rs59158231	3	68360460	A	7	25	ACGTT	0.0003419
rs2131042	3	68361179	G	7	25	A	0.0003419
rs4378971	3	68363844	A	7	25	T	0.0003419
rs10155009	3	68368649	G	9	29	A	0.0001986
rs1491757	3	68369111	G	9	29	T	0.0001986
rs1491756	3	68369288	G	10	29	A	0.0004811
rs1491755	3	68369391	G	10	29	A	0.0004811
rs1552710	3	68386395	A	24	45	G	0.0007128
rs1552711	3	68386397	G	24	45	C	0.0007128
rs2220668	3	68388269	A	30	51	G	0.0009286
rs1388500	3	68388365	G	29	51	C	0.0005253
rs7613895	3	68389846	C	29	51	G	0.0005253
rs262207	3	68390354	G	33	54	T	0.0009764
rs262209	3	68390763	G	33	54	T	0.0009764
rs262210	3	68391629	T	33	54	G	0.0009764
rs262211	3	68391836	C	33	54	G	0.0009764
rs262212	3	68393055	G	29	51	A	0.0005253
rs262214	3	68393442	A	29	51	T	0.0005253
rs262215	3	68393781	G	30	51	T	0.0009286
rs262250	3	68403593	C	29	50	G	0.0009027
rs262248	3	68405259	T	30	52	C	0.00054
rs2220667	3	68419898	G	18	3	C	0.0007671
rs171892	3	68430620	C	29	53	T	0.0001666
rs58640476	3	68433974	A	19	3	ACTTT	0.0004265
rs115620664	3	68441708	G	25	46	A	0.0007575
rs17831048	3	68475353	C	43	18	T	0.0002635
rs113071699	3	70386328	T	41	17	TTAA	0.0004047
rs112187311	3	74102537	A	18	39	G	0.0004141
rs77764654	3	79732148	G	28	8	A	0.0003544
rs76623516	3	79732914	T	28	8	G	0.0003544
rs78727982	3	79732986	G	28	8	T	0.0003544
3:82897854	3	82897854	T	10	29	TC	0.0004811
3:82897861	3	82897861	T	8	29	TTCTTTC	0.00007515

3:88950094	3	88950094	AGAT	20	44	A	0.00009139
rs71623597	3	98146300	A	18	39	AAC	0.0003337
rs6439853	3	98153919	C	18	38	T	0.0007166
3:106522026	3	106522026	A	18	46	T	0.000005263
rs9631453	3	115394563	C	33	11	G	0.0002645
rs6804592	3	115401998	G	3	17	A	0.0007114
rs6777348	3	117137202	T	27	8	C	0.0006326
rs4632551	3	117148573	C	27	8	G	0.0006326
rs201579536	3	118242257	T	10	28	TG	0.000853
rs75752354	3	118242263	T	10	28	A	0.000853
rs6438453	3	118275310	C	6	24	A	0.0002287
rs6438454	3	118275339	C	6	24	G	0.0002287
rs7620329	3	118275517	A	6	24	G	0.0002287
rs9814010	3	118281297	C	6	24	T	0.0002287
rs4353799	3	118283506	T	6	24	C	0.0002287
rs4605536	3	118283580	T	6	24	C	0.0002287
rs111854688	3	122108236	G	22	44	A	0.0003557
rs13079305	3	123953526	T	2	15	C	0.0006243
rs201570082	3	140987054	A	0	11	T	0.0002637
rs200964710	3	141015446	T	20	4	C	0.0007606
rs75704232	3	141016370	C	20	4	G	0.0007606
rs9862069	3	141894430	T	13	33	C	0.0005413
rs9289644	3	141896979	T	13	33	C	0.0005413
rs6764361	3	141898980	C	13	33	T	0.0005413
rs6805279	3	141916255	T	13	33	C	0.0005413
rs11927535	3	141917185	T	13	33	C	0.0005413
rs9818462	3	141986658	C	5	20	G	0.0009659
rs9871394	3	141989460	G	5	20	A	0.0009659
rs28729685	3	141992387	A	5	20	T	0.0009659
rs275688	3	148347114	T	11	0	A	0.0007274
rs275687	3	148347582	C	11	0	A	0.0007274
rs275686	3	148347714	C	11	0	G	0.0007274
rs275684	3	148347952	T	11	0	C	0.0007274
rs275683	3	148347959	G	11	0	A	0.0007274
rs649342	3	148350663	T	11	0	A	0.0007274
rs444752	3	148351096	A	11	0	C	0.0007274
rs413671	3	148351517	T	11	0	A	0.0007274
rs387951	3	148351790	A	11	0	G	0.0007274
rs375839	3	148351839	G	11	0	A	0.0007274
rs201943	3	148352362	C	11	0	T	0.0007274

rs275695	3	148352651	T	11	0	A	0.0007274
rs4058143	3	148352896	CAATT	11	0	C	0.0007274
rs275696	3	148353206	G	11	0	A	0.0007274
rs275697	3	148353740	C	11	0	T	0.0007274
rs275698	3	148353866	T	11	0	C	0.0007274
rs62269231	3	149979842	G	8	27	T	0.0002641
rs13078289	3	152530072	T	4	19	A	0.0005969
rs9881146	3	152681677	A	30	51	G	0.0009286
rs11712121	3	152698743	A	1	14	G	0.0002433
rs1160148	3	159156005	G	8	25	T	0.000871
rs1907860	3	163546423	A	0	11	G	0.0002715
rs79902688	3	163549268	G	0	11	T	0.0002715
rs62293633	3	163557077	G	0	11	C	0.0002715
rs36022510	3	163557357	G	0	11	A	0.0002715
rs71156808	3	163562172	GT	0	11	G	0.0002715
rs62293636	3	163563579	C	0	11	T	0.0002715
rs12491031	3	163564559	C	0	11	T	0.0002715
rs13059483	3	163567068	A	0	11	C	0.0002715
rs13060209	3	163567299	C	0	11	G	0.0002715
3:163568079	3	163568079	AAT	0	11	A	0.0002715
rs12489971	3	163572245	A	0	11	C	0.0002715
rs2365589	3	163572825	G	0	11	T	0.0002715
rs141466002	3	163573679	A	0	11	G	0.0002715
rs34247696	3	163574006	T	0	11	G	0.0002715
rs71630481	3	163574522	C	0	11	CA	0.0002715
rs66817151	3	163574558	A	0	11	C	0.0002715
rs147941663	3	163575270	TTG	0	11	T	0.0002715
rs11412870	3	172336474	CA	18	39	C	0.0004141
rs234028	3	172338633	G	18	38	A	0.0007166
rs234027	3	172339351	G	18	38	A	0.0007166
rs234026	3	172340263	T	18	38	C	0.0007166
rs234022	3	172342028	G	24	51	A	0.00006488
rs234021	3	172342272	A	18	38	G	0.0007166
rs234020	3	172342303	C	18	38	T	0.0007166
rs234019	3	172342922	A	18	38	G	0.0007166
rs234018	3	172344389	A	54	29	C	0.0006016
rs234016	3	172347134	G	54	29	A	0.0006016
rs5854477	3	172354182	CGAAT	20	41	C	0.0005156
rs234007	3	172357122	T	19	39	G	0.0007998
rs234005	3	172358320	T	19	39	C	0.0007998

rs234004	3	172359223	C	19	39	T	0.0007998
rs98106	3	172362707	A	18	39	G	0.0004141
rs113829793	3	172364153	ATAAAT	20	41	A	0.0005156
rs9854527	3	174216289	T	13	32	C	0.0009401
rs59036126	3	182742406	C	1	13	CA	0.0005601
rs11423070	3	182780982	A	1	13	AG	0.0005059
rs11313652	3	182788521	CA	2	16	C	0.0002759
rs6802040	3	183109397	T	30	8	G	0.0001064
rs9647371	3	184284279	T	21	4	C	0.0004069
rs9835850	3	184285655	T	18	3	C	0.0008121
rs9860415	3	184286171	C	18	3	T	0.0008121
rs7644377	3	184295316	T	27	4	C	0.000007245
rs13071695	3	187185791	G	53	28	A	0.000519
rs710496	3	188699330	G	28	9	C	0.0008484
rs13092581	3	188699808	G	28	8	A	0.0003544
3:190280484	3	190280484	CAT	24	6	C	0.0005099
rs200759056	3	190280486	T	24	5	C	0.0001934
rs62293019	3	192336707	A	4	19	G	0.0005969
rs62293020	3	192336826	T	4	19	G	0.0005969
rs62293021	3	192338033	A	3	18	G	0.0003675
rs6780484	3	194243230	C	21	4	G	0.0004069
rs77618188	3	195689376	C	0	12	T	0.0001237
3:195803487	3	195803487	AT	1	13	A	0.0005059
rs4021907	3	197570134	A	30	51	C	0.0009286
rs9880944	3	197647970	A	2	15	C	0.0006243
rs113481869	3	197647973	T	2	15	C	0.0006243
rs111656082	3	197647976	G	2	15	A	0.0006243
3:197876728	3	197876728	C	3	17	T	0.0006426
rs4689202	4	4175894	G	36	12	A	0.0001705
rs10937668	4	5771244	G	31	52	C	0.0009496
rs4688959	4	5771262	T	31	52	C	0.0009496
rs4689308	4	5771295	G	31	52	A	0.0009496
rs5855887	4	5779563	T	55	30	TTTG	0.0006201
rs149986545	4	5781470	T	57	31	C	0.0003656
rs11464608	4	6017245	G	9	27	GA	0.0006689
rs10461053	4	6031632	G	15	36	T	0.0002717
rs739778	4	7703265	A	45	20	G	0.000332
4:9033194	4	9033194	ACG	34	10	A	0.00004996
4:9033196	4	9033196	G	34	11	A	0.0001478
4:12151990	4	12151990	G	28	8	GT	0.0003544

rs62297928	4	19171165	C	0	11	A	0.0002715
rs62297932	4	19201797	A	0	11	G	0.0002715
rs58918138	4	20353473	T	1	19	G	0.000005333
rs77041696	4	20358927	G	1	19	C	0.000005333
rs4552454	4	20386044	A	1	19	C	0.000005333
rs10027333	4	20390198	G	1	19	A	0.000005333
rs7435615	4	20399168	A	1	19	G	0.000005333
rs10006234	4	20408421	G	16	38	T	0.0009291
rs57411082	4	20408564	C	1	19	CTG	0.000005333
rs10026931	4	20430582	G	1	18	C	0.00001169
rs11728803	4	20851589	T	4	19	C	0.0005969
rs71940315	4	20865321	C	4	19	CTT	0.0005969
rs12498200	4	25052381	G	22	47	A	0.00006058
rs759245	4	25058232	G	27	49	A	0.0004874
rs759244	4	25058323	A	27	49	T	0.0004874
rs6821216	4	25058776	G	27	49	C	0.0004874
rs6835786	4	25061194	A	27	49	C	0.0004874
rs7672992	4	25613341	C	31	56	T	0.0001617
rs11096924	4	38151366	A	43	20	G	0.0009741
rs147312727	4	38245334	G	48	24	GGT	0.000451
rs62295295	4	38245657	G	51	26	A	0.0005284
rs73243338	4	38245785	T	49	23	C	0.0002495
rs112873879	4	38246039	C	51	26	T	0.0005284
rs16994359	4	38246265	T	51	26	C	0.0005284
rs62295298	4	38246414	T	51	27	C	0.0009437
rs4832756	4	38246943	A	33	55	T	0.0005652
4:47142112	4	47142112	A	25	50	C	0.00007604
4:49209131	4	49209131	C	24	6	T	0.0005592
rs2616434	4	54616649	G	55	29	A	0.000347
rs2616433	4	54617199	A	54	29	C	0.0006016
rs5858236	4	54617655	C	52	28	CA	0.0009847
rs2616431	4	54618315	C	54	29	G	0.0006016
rs2590807	4	54621567	A	55	29	T	0.000347
rs6826915	4	54622017	A	55	29	G	0.000347
rs62303692	4	56311447	T	19	39	C	0.0007998
rs17218035	4	58930432	G	56	31	A	0.0006352
rs270231	4	58932326	C	58	31	T	0.0002058
rs1605463	4	58934608	G	34	55	A	0.0009818
rs62305808	4	60515628	A	11	32	C	0.000192
rs7676888	4	62164761	G	41	18	A	0.0007885

rs2342454	4	62167519	G	41	18	A	0.0007885
rs200738201	4	64476893	T	31	53	A	0.0005517
4:66104499	4	66104499	CAT	11	0	C	0.0007274
rs1220858	4	70805831	T	49	21	A	0.00006416
rs75408729	4	77039736	T	6	22	G	0.0008352
rs6835713	4	78168932	T	32	53	G	0.0009656
4:82709303	4	82709303	G	12	0	GTATATATACA	0.0003567
rs72659283	4	84907590	T	15	1	C	0.0003131
rs2135284	4	84908159	G	15	1	A	0.0003131
rs72659284	4	84910400	G	15	1	A	0.0003131
rs72659286	4	84916214	G	15	1	T	0.0003131
rs72659290	4	84917345	C	14	1	T	0.0006347
rs72659295	4	84934184	A	15	1	G	0.0003131
rs74476563	4	84935594	GT	15	1	G	0.0003131
rs72659296	4	84936325	T	15	1	G	0.0003131
rs72659297	4	84938960	C	15	1	G	0.0003131
rs4129091	4	84941953	A	15	1	G	0.0003131
rs113134562	4	84951643	T	15	1	C	0.0003131
rs17007881	4	84953207	G	17	1	A	0.00007397
rs1921883	4	84953383	C	17	1	T	0.00007397
rs72660815	4	84954205	G	17	1	T	0.00007397
rs72660816	4	84954602	C	17	1	A	0.00007397
rs72660817	4	84955535	G	17	1	A	0.00007397
rs72660819	4	84956002	C	17	1	A	0.00007397
rs1317020	4	84956277	T	17	1	C	0.00007397
rs56004187	4	84956736	C	17	1	T	0.00007397
rs1921880	4	84957198	T	17	1	G	0.00007397
rs1317519	4	84957652	T	17	1	C	0.00007397
rs2091745	4	84961080	G	28	9	A	0.0008484
rs72660826	4	84965875	G	17	2	A	0.0004014
rs62306398	4	90055581	T	29	9	C	0.0004831
rs713175	4	90073235	T	28	9	G	0.0008484
rs62304660	4	90077751	C	28	8	T	0.0003544
rs13127840	4	90078734	A	28	9	T	0.0008484
rs756004	4	90164712	G	20	40	C	0.0008835
rs1160489	4	99773191	T	14	1	G	0.0006347
rs6851731	4	101248226	A	49	25	G	0.00085
rs11420114	4	102025611	AT	17	36	A	0.0008922
4:109041155	4	109041155	T	8	33	G	0.000005054
rs441216	4	119630402	A	24	6	G	0.0005592

4:119987369	4	119987369 G	28	9	A	0.0008484
rs111866828	4	120124537 A	19	3	G	0.0004265
rs9997559	4	123785086 G	25	6	A	0.0003059
rs7674648	4	123785314 C	25	6	T	0.0003059
rs10000779	4	123786114 G	25	6	A	0.0003059
rs10023647	4	123786531 T	25	6	C	0.0003059
rs72672991	4	123787692 T	25	6	G	0.0003059
rs9996546	4	123788027 C	25	6	T	0.0003059
rs7694627	4	123788495 C	25	6	T	0.0003059
rs7670829	4	123788691 C	25	6	G	0.0003059
rs6817113	4	123790067 C	25	6	T	0.0003059
rs13435300	4	123791710 G	25	6	A	0.0003059
rs28449370	4	123792219 T	25	6	C	0.0003059
rs45585532	4	123792343 CTGTT	25	6	C	0.0003059
rs10012814	4	123795337 T	25	6	A	0.0003059
rs9990554	4	123795408 A	25	6	G	0.0003059
rs3804155	4	123795653 G	25	6	A	0.0003059
rs45565835	4	123796735 G	25	6	A	0.0003059
rs7700205	4	123798183 T	25	6	C	0.0003059
rs17474021	4	123798711 C	25	6	T	0.0003059
rs41537948	4	123799325 G	25	6	A	0.0003059
rs34350503	4	123827911 A	35	11	G	0.00008135
rs12513181	4	123835656 C	35	11	A	0.00008135
rs12648093	4	123838758 A	35	11	G	0.00008135
rs62325775	4	123947268 A	27	8	G	0.0006326
rs13142857	4	124054093 G	30	10	A	0.0006347
rs304653	4	124080712 C	30	10	T	0.0006347
rs376830774	4	124487236 A	46	21	ATCTG	0.0003668
4:146615412	4	146615412 A	12	0	G	0.0003368
rs6419330	4	146675915 C	14	33	T	0.0007122
rs70949643	4	154070887 GA	6	21	G	0.0007337
rs12504642	4	154091900 A	16	39	G	0.00009784
rs6535901	4	154093408 C	33	54	T	0.0009764
rs6820726	4	154093464 T	33	55	C	0.0005652
rs10023596	4	154094001 G	34	55	A	0.0009818
rs11099874	4	154094107 C	33	55	G	0.0005652
rs11099875	4	154094109 C	33	55	G	0.0005652
rs11099876	4	154094235 G	33	55	C	0.0005652
rs10002011	4	154094675 A	32	55	G	0.0003175
rs10001881	4	154094682 T	32	55	C	0.0003175

rs7671132	4	154094793	T	33	55	C	0.0005652
rs7696186	4	154095073	C	33	55	T	0.0005652
rs142792673	4	154096164	GTTTTGT	56	30	G	0.0001481
rs4145338	4	154654323	C	11	0	T	0.0007274
rs10434139	4	154654665	A	11	0	G	0.0007274
rs56256921	4	154655929	A	11	0	G	0.0007274
rs11931479	4	154664523	G	11	0	A	0.0007274
rs17030417	4	154670813	G	11	0	A	0.0007274
rs11946635	4	154674080	A	11	0	G	0.0007274
rs11946638	4	154674095	T	11	0	G	0.0007274
4:155246473	4	155246473	A	6	23	G	0.000429
rs71605285	4	157055724	G	37	13	C	0.0002102
4:161072770	4	161072770	G	9	27	A	0.000654
rs145598110	4	161077302	C	12	32	T	0.0004387
rs115431006	4	161077729	T	12	32	C	0.0004387
rs2349527	4	161078051	A	12	32	T	0.0004387
rs2882184	4	161078069	A	12	32	G	0.0004387
rs2349529	4	161078136	A	12	32	G	0.0004387
rs2349530	4	161078142	T	12	32	G	0.0004387
rs55675443	4	161078540	A	12	32	C	0.0004387
rs1573797	4	161078915	A	12	32	T	0.0004387
rs1573795	4	161079052	A	12	32	G	0.0004387
rs2349533	4	161079172	C	12	32	A	0.0004387
rs6852275	4	161079345	A	12	32	G	0.0004387
rs1882685	4	161080394	A	12	32	G	0.0004387
rs4519753	4	161080636	T	12	32	C	0.0004387
rs28557270	4	161080971	C	12	32	T	0.0004387
rs28880970	4	161081088	G	12	32	A	0.0004387
rs3905353	4	161082121	C	12	32	G	0.0004387
rs1579025	4	161082372	G	12	32	C	0.0004387
rs55803229	4	161084958	G	12	32	C	0.0004387
rs72691371	4	161092040	C	12	32	G	0.0004387
4:163515735	4	163515735	A	3	22	AAATAAATAAATAAAT	0.00001976
rs9995462	4	163515736	T	5	25	A	0.00003701
rs62355242	4	167715106	A	24	6	T	0.0005592
rs901236	4	172740734	T	21	43	G	0.0003256
rs4396959	4	172743234	G	34	55	A	0.0009818
rs346100	4	179603662	G	44	18	A	0.0001482
rs2378925	4	179608265	G	45	21	A	0.0006315
rs118806	4	179609480	G	44	18	A	0.0001482

rs382249	4	179610406	G	44	18	T	0.0001482
rs393318	4	179611902	C	43	20	T	0.0009741
rs427796	4	179612525	A	43	20	C	0.0009741
rs10033023	4	179613705	T	42	17	C	0.0002305
4:181184074	4	181184074	AC	24	5	A	0.0002188
rs17834666	4	181229200	A	21	4	G	0.0004069
rs58393532	4	181232190	G	21	42	GT	0.000319
rs73869758	4	181234993	T	21	4	C	0.0004069
rs150126595	4	181246227	G	28	8	GT	0.0003544
4:184660581	4	184660581	TCAC	11	30	T	0.0006171
4:184660582	4	184660582	T	11	30	G	0.0006171
rs11282148	4	187992506	T	2	17	TTTCTTTCC	0.0001534
rs75794090	4	187992531	C	1	16	T	0.00005445
rs78869334	4	187992532	C	1	16	T	0.00005445
rs6822725	4	187992812	C	4	21	T	0.0001601
rs4323164	4	187996662	A	7	25	G	0.0003419
rs13141650	4	187999052	C	8	25	T	0.000871
rs7662795	4	188004538	A	8	25	C	0.000871
rs2029256	4	188006491	G	8	25	A	0.000871
rs7679189	4	188008668	A	8	25	T	0.000871
rs11936290	4	188010829	T	9	27	C	0.000654
rs11936363	4	188010846	A	9	27	G	0.000654
rs35927269	4	188010890	T	9	27	TTG	0.000654
rs4862765	4	188011453	C	9	27	T	0.000654
rs4862766	4	188011455	T	9	27	C	0.000654
rs28520426	4	188011697	G	9	27	T	0.000654
rs28495215	4	188011891	G	9	27	T	0.000654
rs57534287	4	188012649	C	9	27	G	0.000654
rs10625579	4	188012913	CAAT	9	27	C	0.000654
rs4862767	4	188012991	C	9	27	T	0.000654
rs4862768	4	188013024	G	9	27	A	0.000654
rs11332958	4	188013035	A	9	27	AG	0.000654
rs10013620	4	188013428	T	9	27	C	0.000654
rs7687788	4	188013656	T	9	27	G	0.000654
rs7665787	4	188013721	C	9	27	T	0.000654
rs6553035	4	188014134	G	9	27	A	0.000654
rs1994430	4	188016649	T	9	27	A	0.000654
rs62353542	4	188704488	A	36	14	G	0.0007756
rs62353544	4	188706790	T	36	14	G	0.0007756
rs7696678	4	189015024	T	21	41	C	0.0009669

4:189191970	4	189191970	AG	24	6	A	0.0005592
rs62340474	4	189975229	T	5	21	C	0.0005184
rs34465723	4	190008501	A	48	24	G	0.0007984
rs2101911	4	190009975	T	48	24	C	0.0007984
rs11132604	4	190010157	C	31	52	T	0.0009496
rs1566102	4	190025016	G	21	41	T	0.0009669
rs200541588	4	190861229	G	13	32	T	0.0009401
rs113183696	4	190861231	A	15	34	C	0.0008313
rs4246755	5	2078006	T	30	51	G	0.0009286
rs16868033	5	2335750	G	20	4	C	0.0007606
rs469381	5	2344502	T	18	3	C	0.0008121
rs465950	5	2354762	C	20	4	T	0.0007606
rs460672	5	2355032	G	20	3	C	0.0002211
rs462906	5	2355389	T	19	3	C	0.0004265
rs458006	5	2355587	A	18	3	C	0.0008121
rs113602711	5	2355658	T	19	3	TATGCTAATGTCAGAAAGAG	0.0004265
rs58937979	5	2355682	T	19	3	C	0.0004265
rs2641215	5	2355878	T	19	3	G	0.0004265
rs456732	5	2358645	G	19	3	A	0.0004265
rs465527	5	2359450	A	16	2	G	0.0007845
rs2628160	5	2361180	T	16	2	G	0.0007845
rs72180957	5	3067375	C	50	25	CTT	0.000499
rs1173180	5	4803992	G	52	28	C	0.0009847
rs13164733	5	9893560	T	31	52	G	0.0009496
rs27478	5	13168261	G	20	40	A	0.0008835
rs39963	5	13182557	T	16	37	G	0.0003171
rs32540	5	13184160	T	23	46	C	0.0002182
rs27481	5	13185887	A	27	53	C	0.00004506
rs28013	5	13186559	C	16	37	T	0.0003171
rs26228	5	13188629	G	16	37	C	0.0003171
rs26226	5	13189555	C	27	53	G	0.00004506
rs26225	5	13191338	A	23	46	C	0.0002182
rs27763	5	13192570	T	16	37	G	0.0003171
rs2067324	5	13193768	A	17	37	AATC	0.0006349
rs28023	5	13194952	C	16	37	T	0.0003171
rs32545	5	13205011	A	17	37	G	0.0006349
rs32547	5	13207696	G	16	37	A	0.0003171
rs250545	5	13210834	C	23	46	A	0.0002182
rs32521	5	13215417	A	16	37	G	0.0003171
rs26223	5	13225937	C	16	37	A	0.0003171

rs27488	5	13227153	C	16	37	T	0.0003171
rs26221	5	13227610	G	20	44	T	0.00009139
rs26220	5	13228033	C	16	37	G	0.0003171
rs113858514	5	13228691	AATAT	17	37	A	0.0006349
rs32523	5	13229322	G	17	37	C	0.0006349
rs32524	5	13229435	A	16	37	G	0.0003171
rs28186	5	13229659	C	17	37	T	0.0006349
rs250547	5	13230263	A	17	38	G	0.0003648
rs250546	5	13230705	T	16	37	G	0.0003171
rs13162652	5	13232017	C	16	37	T	0.0003171
rs962673	5	13235570	T	16	37	C	0.0003171
rs962674	5	13235825	T	16	37	C	0.0003171
rs962675	5	13235909	A	16	37	G	0.0003171
rs1596773	5	13236478	G	16	37	A	0.0003171
rs2197494	5	13236709	A	16	37	G	0.0003171
rs2197495	5	13236731	T	16	37	C	0.0003171
rs12173099	5	13240427	T	16	37	C	0.0003171
rs12173100	5	13240611	A	16	37	C	0.0003171
rs10066447	5	13242974	T	19	39	C	0.0007998
rs62341417	5	13244274	T	17	37	C	0.0006349
rs11948770	5	13246336	C	16	37	T	0.0003171
rs13163485	5	13249488	A	27	52	G	0.00008446
5:13249768	5	13249768	TA	17	37	T	0.0006349
rs200425009	5	13249770	A	17	37	AT	0.0006349
rs139530285	5	13249773	G	17	37	GATAACT	0.0006349
rs13164139	5	13249816	C	27	52	T	0.00008446
rs4292447	5	13251163	C	17	37	G	0.0006349
rs11748417	5	13252122	A	17	37	G	0.0006349
rs36036547	5	13252567	G	17	37	A	0.0006349
rs34482579	5	13252592	T	17	37	A	0.0006349
rs12522258	5	13252798	C	27	52	A	0.00008446
rs10045395	5	13253744	T	17	37	C	0.0006349
rs7737522	5	13254339	C	27	52	T	0.00008446
rs4077925	5	13261898	G	17	37	C	0.0006349
rs4077927	5	13262146	A	17	37	G	0.0006349
rs6874976	5	13263052	A	27	52	G	0.00008446
rs34774752	5	13263229	C	27	52	CT	0.00008446
rs13173139	5	13264123	G	17	37	A	0.0006349
rs62339660	5	13265236	G	27	52	A	0.00008446
rs62339661	5	13265254	G	27	52	A	0.00008446

5:13265449	5	13265449	A	31	54	ACG	0.0001341
rs79357435	5	13265572	C	17	37	T	0.0006349
rs36165772	5	13266309	A	28	52	G	0.0002748
rs55706056	5	13266798	C	17	37	T	0.0006349
rs4626338	5	13267566	T	17	37	C	0.0006349
rs4476721	5	13269157	T	17	37	C	0.0006349
rs7729579	5	13271019	A	27	52	G	0.00008446
rs6861775	5	13273222	A	27	52	G	0.00008446
rs11739128	5	13273470	G	17	37	A	0.0006349
rs4377703	5	13274310	T	28	52	C	0.0001612
rs7713535	5	13275208	A	27	52	G	0.00008446
rs62339663	5	13276050	C	27	52	T	0.00008446
rs6859654	5	13277990	A	24	50	G	0.00003799
rs6859695	5	13278059	A	27	52	C	0.00008446
rs13153960	5	13278199	T	17	37	C	0.0006349
rs35651716	5	13278400	C	17	37	G	0.0006349
rs13189083	5	13280592	T	27	52	C	0.00008446
rs13169762	5	13281103	G	17	37	A	0.0006349
rs4235626	5	13281305	T	27	52	C	0.00008446
rs11133733	5	13290183	T	26	48	A	0.0004647
rs12187447	5	13295099	C	26	48	T	0.0004647
rs11747612	5	13305637	C	27	48	T	0.0008377
5:13309772	5	13309772	C	17	35	CGTGTATATACACGTGTATATA(	0.0009157
rs1470469	5	13317993	C	26	48	A	0.0004647
rs1446057	5	13318740	C	27	48	T	0.0008377
rs2166397	5	13329402	C	26	48	T	0.0004647
5:13330005	5	13330005	AACATGTAATATTTTATATAT	26	48	A	0.0004647
rs1530514	5	13332031	C	26	48	T	0.0004647
rs4702930	5	13332729	C	26	48	A	0.0004647
rs11133742	5	13334744	T	27	49	C	0.0002589
rs6876615	5	13338575	A	26	48	C	0.0004647
rs7714774	5	13341369	T	21	42	C	0.0005665
rs2218533	5	13341742	C	26	48	G	0.0004647
rs13153366	5	13342831	C	26	48	A	0.0004647
rs978845	5	13348985	T	27	48	C	0.0008377
rs71585699	5	13349598	C	29	52	T	0.0001305
rs7715889	5	13351114	G	26	48	A	0.0004647
rs13168766	5	13351173	C	17	37	G	0.0006349
rs1466015	5	13353683	C	26	48	T	0.0004647
rs11133748	5	13364690	T	26	48	C	0.0004647

rs12153106	5	13370624	C	26	49	T	0.0002646
rs62342776	5	13395422	A	15	34	G	0.0008313
rs66904646	5	13489425	T	15	34	C	0.0008313
rs68032177	5	13490410	T	15	34	A	0.0008313
rs151145	5	13491184	G	15	34	C	0.0008313
rs151143	5	13492001	C	15	34	T	0.0008313
rs11949792	5	13492497	A	15	34	G	0.0008313
rs230681	5	13494184	C	15	34	T	0.0008313
rs34206111	5	13495880	C	15	34	T	0.0008313
rs35521632	5	13496453	C	15	34	T	0.0008313
rs230052	5	13496833	A	15	34	C	0.0008313
rs230045	5	13499857	C	15	34	T	0.0008313
rs3868287	5	13503064	G	15	34	T	0.0008313
5:16796588	5	16796588	AAAG	22	43	A	0.000565
rs143565175	5	19187627	G	31	11	C	0.0008085
rs373271730	5	20909293	G	30	51	GAT	0.0009286
rs6451918	5	20909303	G	29	50	T	0.0009027
rs67589059	5	20915694	T	30	51	C	0.0009286
rs2201403	5	20919166	G	30	51	C	0.0009286
rs2201404	5	20919200	G	30	51	A	0.0009286
rs184303633	5	21357168	C	15	0	G	0.00003259
rs71607499	5	25608509	C	16	35	CT	0.000956
rs36999	5	25611107	C	15	35	T	0.0004795
rs12513781	5	25620280	A	14	36	G	0.0001265
rs12519886	5	25621441	G	15	36	C	0.0002717
rs62355015	5	25622323	G	15	35	C	0.0004795
rs62355019	5	25625327	C	15	36	T	0.0002717
rs10057753	5	25626830	T	21	41	A	0.0009669
rs34477507	5	25627052	C	15	36	G	0.0002717
rs6450656	5	28648576	A	35	57	C	0.0003656
rs7722988	5	28648707	T	35	57	C	0.0003656
rs6893661	5	34732224	A	57	31	G	0.0003656
rs11747914	5	34831252	A	35	13	G	0.0006539
rs1056115	5	34838085	C	39	16	G	0.0006105
rs62355748	5	34841579	G	40	16	A	0.0003516
rs3846629	5	34841785	G	40	16	C	0.0003516
rs3846630	5	34841812	G	40	16	C	0.0003516
rs16868532	5	34856805	A	41	18	G	0.0007885
rs56015042	5	34856818	G	41	18	A	0.0007885
rs16868533	5	34856874	C	41	17	A	0.0004047

rs16868534	5	34857445	G	41	18	A	0.0007885
rs16868536	5	34857470	T	41	18	C	0.0007885
rs58930292	5	34858091	A	41	18	AT	0.0007885
rs6869117	5	34858483	G	41	18	A	0.0007885
rs72732819	5	34858595	T	41	18	C	0.0007885
rs72732820	5	34858662	C	41	18	A	0.0007885
rs146615654	5	34858788	TA	41	18	T	0.0007885
rs72732821	5	34859011	A	41	18	C	0.0007885
rs77456162	5	35113923	T	0	12	C	0.0001237
rs1049524	5	36687393	A	28	50	G	0.0005077
rs60229781	5	36690843	C	27	48	G	0.0008377
rs61496493	5	36690844	A	27	48	G	0.0008377
rs158790	5	36837911	C	44	18	T	0.0001482
rs158786	5	36850887	G	45	20	A	0.000332
rs11347966	5	37158892	A	13	0	AT	0.0001541
rs13356615	5	37208782	G	14	1	C	0.000663
5:57402182	5	57402182	A	12	34	G	0.0001353
rs346406	5	62490220	G	24	6	A	0.0005592
rs186968	5	62498557	C	24	6	T	0.0005592
rs59857064	5	73735191	T	12	0	A	0.0003422
rs201096991	5	83266476	G	0	10	A	0.0005917
rs6859947	5	87901547	A	55	30	C	0.0006201
rs6452797	5	87901964	A	55	30	G	0.0006201
rs324887	5	87905818	A	55	30	G	0.0006201
rs192015	5	87906959	T	55	30	C	0.0006201
rs324888	5	87907314	T	55	30	C	0.0006201
rs184453	5	87908427	T	55	30	G	0.0006201
rs226489	5	87909517	T	55	29	C	0.000347
rs324890	5	87909843	C	55	30	A	0.0006201
rs1644040	5	87909882	T	55	30	C	0.0006201
rs55855860	5	87912634	AAAT	55	30	A	0.0006201
rs324897	5	87914467	A	55	30	C	0.0006201
rs324900	5	87917231	G	55	30	A	0.0006201
rs188971	5	87921066	G	55	30	A	0.0006201
rs1628054	5	87922342	T	55	30	C	0.0006201
rs1659072	5	87923770	G	55	30	A	0.0006201
rs2545694	5	87928570	A	55	30	G	0.0006201
rs1659070	5	87929515	G	55	30	C	0.0006201
rs10053136	5	87930488	A	55	30	G	0.0006201
rs2009730	5	87933568	A	55	30	G	0.0006201

5:89461167	5	89461167	G	23	47	GT	0.0001212
5:89461168	5	89461168	G	23	47	T	0.0001212
rs10514355	5	92051536	T	11	0	C	0.0007274
5:94344766	5	94344766	A	36	14	G	0.000808
5:94344769	5	94344769	G	37	14	A	0.0004701
5:94344774	5	94344774	A	36	14	G	0.000808
rs80343556	5	98624487	GT	6	22	G	0.0007921
rs17166834	5	98624513	G	6	22	A	0.0007921
rs10477869	5	105959382	C	42	18	A	0.0004599
rs202030174	5	105960773	C	42	17	A	0.0002305
rs74957701	5	105960906	C	42	17	G	0.0002305
rs190734124	5	105962622	A	42	17	C	0.0002305
rs183331089	5	105962624	G	42	17	C	0.0002305
rs6873840	5	105965170	G	42	17	A	0.0002305
rs138302217	5	105974033	T	41	17	C	0.0004047
rs58151039	5	105975178	C	42	17	A	0.0002305
rs5870212	5	105976556	G	42	17	GA	0.0002305
rs4616879	5	105982997	C	42	17	T	0.0002305
rs9327970	5	105985135	G	42	17	A	0.0002305
rs1158614	5	105985695	C	41	17	T	0.0004047
rs966225	5	105986040	T	42	17	A	0.0002305
rs1370474	5	105990288	C	41	17	T	0.0004047
rs770165	5	107046018	T	26	5	G	0.00005139
rs140935191	5	113218057	G	2	15	T	0.0006952
rs61684041	5	113457167	T	27	8	C	0.0006326
5:115814626	5	115814626	G	30	6	GT	0.00002435
5:115814627	5	115814627	G	32	6	T	0.000003036
rs13167197	5	125408090	C	41	18	T	0.0008624
rs10463817	5	125471402	G	43	20	T	0.0009741
rs10477666	5	125478358	C	43	20	A	0.0009741
rs374455672	5	125482719	ATAAAG	44	20	A	0.0005739
rs140272168	5	125486927	G	43	20	A	0.0009741
rs140084416	5	125487289	CTCTG	43	20	C	0.0009741
rs6888912	5	125489390	C	43	20	T	0.0009741
rs6870502	5	125489767	T	43	20	C	0.0009741
rs3843819	5	125496145	C	34	11	T	0.0001478
rs3898923	5	125498708	C	37	15	T	0.0009053
rs3909804	5	125507018	A	35	13	C	0.0006539
rs10061705	5	125507903	T	35	13	C	0.0006539
rs10900783	5	125512816	T	35	13	C	0.0006539

rs13359614	5	125550942	C	35	13	T	0.0006539
rs2194080	5	126634444	G	11	0	C	0.0007274
rs864117	5	143142455	T	51	26	A	0.0005284
rs2351023	5	153265309	C	51	27	T	0.0009437
rs919221	5	153271076	T	54	29	C	0.0006016
rs1366095	5	153271756	G	54	29	A	0.0006016
rs2560037	5	153271973	G	54	29	A	0.0006016
rs1366096	5	153272041	A	54	29	G	0.0006016
rs35870631	5	153272504	C	52	29	CA	0.0008265
rs1461242	5	153282305	C	54	29	T	0.0006016
rs2964000	5	153290715	C	56	31	T	0.0006352
rs11330171	5	153297804	T	55	31	TA	0.0005984
rs973319	5	153303618	C	30	53	T	0.0003072
rs1461238	5	153304416	C	30	53	T	0.0003072
rs35257267	5	153304538	T	30	53	C	0.0003072
rs970751	5	153305796	T	30	53	C	0.0003072
rs386248	5	153308317	T	30	53	G	0.0003072
rs35910531	5	153310336	TAAAA	30	54	T	0.0001708
rs406708	5	153311032	A	30	53	G	0.0003072
rs432891	5	153311464	C	31	53	T	0.0005517
rs394446	5	153311841	G	30	53	A	0.0003072
rs381582	5	153312282	A	30	53	G	0.0003072
rs454317	5	153312771	A	30	53	G	0.0003072
rs201644188	5	153313518	G	30	53	GAGACA	0.0003072
rs448234	5	153315300	A	30	53	G	0.0003072
rs1438586	5	153315743	C	30	53	A	0.0003072
rs11167651	5	153322296	A	30	53	C	0.0003072
rs4349768	5	153323126	A	30	53	C	0.0003072
rs690091	5	153323948	G	30	53	A	0.0003072
rs557697	5	153324256	C	30	53	A	0.0003072
rs689836	5	153324406	G	30	53	A	0.0003072
rs2560068	5	153330453	T	30	53	C	0.0003072
rs5872353	5	153336184	CT	30	53	C	0.0003072
rs11355335	5	153337255	TA	30	53	T	0.0003072
rs286958	5	153338048	T	30	53	C	0.0003072
rs286959	5	153338516	C	30	53	T	0.0003072
rs286961	5	153341404	C	30	53	G	0.0003072
rs2560050	5	153346190	G	30	53	C	0.0003072
rs11267325	5	153347553	GAAGTCTATGGTATCA	31	52	G	0.0009496
rs4958688	5	153351519	G	30	53	A	0.0003072

rs4958689	5	153351720	T	30	53	C	0.0003072
rs10053993	5	153355854	A	31	53	G	0.0005517
rs199679691	5	154592152	G	39	15	A	0.0003228
rs1095103	5	158088445	A	33	12	G	0.0005855
rs1095104	5	158088897	A	33	12	G	0.0005855
rs824863	5	158090913	C	33	12	G	0.0005855
rs403334	5	158096651	T	33	12	G	0.0005855
rs33140	5	158097381	G	33	12	C	0.0005855
rs184945	5	158100084	T	33	12	C	0.0005855
rs33192	5	158103549	A	33	12	C	0.0005855
rs12153343	5	158964827	T	27	50	A	0.0002777
5:159308310	5	159308310	CTCTG	18	2	C	0.0001998
rs35019914	5	161444006	C	18	3	T	0.0008121
rs189855007	5	161875773	A	13	0	G	0.00016
rs12189109	5	162746500	T	12	0	C	0.0003422
rs12186371	5	162748604	T	12	0	C	0.0003422
rs1019447	5	162751240	T	12	0	C	0.0003422
rs2901732	5	162752132	T	12	0	C	0.0003422
rs1363151	5	162756297	A	13	0	T	0.00016
rs1421840	5	162773548	G	21	4	A	0.0004069
rs13354373	5	162775966	G	29	50	A	0.0009027
rs13360947	5	162776086	G	29	50	C	0.0009027
rs58349075	5	162777481	AGTTTT	29	50	A	0.0009027
rs10078474	5	162777764	T	29	50	C	0.0009027
rs9313959	5	162777927	C	29	50	T	0.0009027
rs10056393	5	162777944	G	29	50	A	0.0009027
rs9313960	5	162778229	C	29	50	T	0.0009027
rs10214000	5	162778238	C	29	50	G	0.0009027
rs2216559	5	162778631	G	23	5	A	0.0003619
rs9313962	5	162778654	A	29	50	G	0.0009027
rs10040552	5	162778735	C	29	50	G	0.0009027
rs10038068	5	162778786	G	29	50	A	0.0009027
rs11953070	5	162779652	C	29	50	T	0.0009027
rs10060724	5	162780014	T	29	50	A	0.0009027
rs10062341	5	162780026	C	29	50	T	0.0009027
rs10060726	5	162780028	G	29	50	A	0.0009027
rs10060800	5	162780114	C	29	50	A	0.0009027
rs10062403	5	162780137	C	29	50	T	0.0009027
rs34587245	5	162780723	A	29	50	G	0.0009027
rs35466466	5	162780761	A	29	50	G	0.0009027

rs56409434	5	162780930	G	29	50	C	0.0009027
rs10073855	5	162780989	T	29	50	C	0.0009027
rs4869037	5	162781126	C	29	50	G	0.0009027
rs4869038	5	162781393	C	29	50	T	0.0009027
rs12189058	5	162781710	T	29	50	C	0.0009027
rs12186706	5	162781814	C	29	50	A	0.0009027
rs4869039	5	162782331	T	29	50	C	0.0009027
rs4869041	5	162782387	T	29	50	C	0.0009027
rs61427225	5	162782877	T	29	50	G	0.0009027
rs34901049	5	162782939	A	29	50	G	0.0009027
rs1895175	5	162803569	A	31	11	T	0.0008085
rs35167126	5	162803828	G	31	11	A	0.0008085
rs70997044	5	162810722	AAGGG	21	4	A	0.000448
rs55982071	5	168253296	A	1	13	G	0.0005059
rs59344702	5	168254496	C	1	13	T	0.0005059
rs7726584	5	168254897	G	1	13	T	0.0005059
rs7707369	5	168255217	A	1	13	G	0.0005059
rs11956426	5	168255738	T	0	13	G	0.00005596
rs61430969	5	168257250	A	0	13	G	0.00005596
rs10607223	5	168258035	C	0	13	CACAG	0.00006504
rs6883428	5	168258281	G	0	13	A	0.00005596
rs72820025	5	172807846	A	6	23	G	0.000429
rs4868495	5	174611803	C	0	12	T	0.0001237
rs4868496	5	174611877	T	0	12	A	0.0001237
rs60545442	5	174615815	TG	0	14	T	0.00002513
rs6601202	5	177486345	T	21	44	C	0.0001836
rs11950505	5	177487180	T	21	44	C	0.0001836
rs10038486	5	177487613	G	21	44	A	0.0001836
rs6601205	5	177500019	G	19	45	A	0.00002313
rs11739583	5	177504116	G	17	40	A	0.0001139
rs11748761	5	177504158	A	17	40	G	0.0001139
rs34491975	5	177506311	C	17	40	T	0.0001139
rs62390499	5	177506330	T	17	40	C	0.0001139
rs113305754	5	177506427	CA	17	40	C	0.0001139
rs55702409	5	177506578	A	17	40	G	0.0001139
rs56395215	5	177506636	G	17	40	A	0.0001139
rs59413527	5	177506695	C	17	40	CA	0.0001139
rs4470773	5	177507080	G	16	40	C	0.00005288
rs4501359	5	177507167	T	17	40	C	0.0001139
rs4519941	5	177507287	A	17	40	G	0.0001139

rs4518421	5	177507302	C	17	40	T	0.0001139
rs4526126	5	177507306	C	17	40	T	0.0001139
rs4976806	5	177507767	T	15	37	C	0.0001512
rs4976807	5	177507914	G	15	37	C	0.0001512
rs4976808	5	177507917	G	15	37	A	0.0001512
rs62390501	5	177508392	A	15	37	C	0.0001512
rs34890405	5	177508458	C	15	37	G	0.0001512
rs58184656	5	177508895	C	15	37	T	0.0001512
rs62390504	5	177508944	G	15	37	C	0.0001512
rs67596535	5	177509098	C	15	37	T	0.0001512
rs4501357	5	177510483	A	15	37	G	0.0001512
rs4370294	5	177510515	G	15	38	A	0.00008268
rs4608937	5	177510571	A	15	37	G	0.0001512
rs12656767	5	177511433	A	18	38	G	0.0007166
rs12652997	5	177524504	A	22	45	G	0.0002011
rs35312700	5	177525307	G	22	46	C	0.0001115
rs146888227	5	177525995	A	22	46	ATTTTAT	0.0001115
rs2296576	6	12008262	G	42	19	C	0.0008809
rs6456473	6	22221924	G	40	16	C	0.0003516
rs1891086	6	22222129	A	39	15	C	0.0003011
rs1361550	6	22230762	C	20	4	T	0.0007606
6:23291477	6	23291477	A	35	16	ACATACATTATACATAGTATAT/	0.0008457
6:23406822	6	23406822	G	50	26	T	0.0008986
rs10456346	6	26966846	G	0	10	A	0.0005917
rs10456347	6	26966853	G	0	10	A	0.0005917
rs10456348	6	26969033	A	0	10	T	0.0005917
rs146539976	6	26977290	G	0	10	T	0.0005917
rs78512949	6	28328293	A	12	0	G	0.0003422
rs112181044	6	28329677	T	14	1	C	0.0006347
rs76043707	6	28338473	C	12	0	T	0.0003422
rs78620132	6	28339253	C	12	0	T	0.0003422
rs111669150	6	28362987	T	12	0	C	0.0003422
rs17393066	6	28364793	G	12	0	A	0.0003422
rs2232419	6	28367768	A	12	0	G	0.0003422
rs10484541	6	28371346	C	12	0	G	0.0003422
rs143000906	6	28374338	TA	11	0	T	0.0007274
rs56241479	6	28389174	T	13	0	G	0.00016
rs200981348	6	28390484	TA	12	0	T	0.0003786
rs61653240	6	28403541	TA	13	0	T	0.0001681
rs2531807	6	28420013	G	12	0	A	0.0003422

rs113145976	6	28438650	C	13	0	T	0.00016
rs9391676	6	28683743	T	29	51	C	0.0005253
rs200314214	6	28685339	T	56	29	C	0.0001006
rs1233576	6	28710244	C	32	50	T	0.0007785
rs1233577	6	28710303	T	31	54	G	0.0005212
rs4713208	6	29283579	T	46	18	C	0.00004442
rs720831	6	29284518	A	46	18	G	0.00004442
rs10447393	6	29316852	T	46	20	C	0.0001885
rs3094561	6	29404157	T	31	52	G	0.0009496
rs3117194	6	29426008	T	31	52	C	0.0009496
rs3128854	6	29431698	A	31	52	G	0.0009496
rs2107191	6	29434295	C	31	52	T	0.0009496
6:32670159	6	32670159	G	16	2	GTGT	0.0008266
rs78991495	6	47730859	A	21	4	G	0.0009338
rs62451863	6	51164272	A	2	16	G	0.0003115
rs5001609	6	53664396	C	26	47	G	0.0007993
rs4342426	6	53664707	C	26	47	T	0.0007993
rs4276503	6	53665789	T	26	47	G	0.0007993
rs9382239	6	53668765	A	26	47	T	0.0007993
rs3000998	6	53669994	A	26	47	G	0.0007993
rs4269375	6	53674055	G	26	47	A	0.0007993
rs4475321	6	53674284	C	26	47	T	0.0007993
rs4715425	6	53676354	G	26	47	A	0.0007993
rs6458960	6	53679557	A	26	47	G	0.0007993
rs3001003	6	53682419	G	26	47	A	0.0007993
rs9367542	6	53682971	T	26	47	G	0.0007993
rs6934444	6	53709891	C	26	47	A	0.0007993
rs6900929	6	53716382	C	26	47	T	0.0007993
rs6458970	6	53723814	C	26	47	T	0.0007993
rs9370236	6	53724274	T	26	47	C	0.0007993
rs4587161	6	53741885	G	26	47	A	0.0007993
rs7767443	6	53744589	G	26	48	A	0.0004647
rs9349686	6	53750470	A	26	47	G	0.0007993
rs4715431	6	53756628	A	26	47	G	0.0007993
rs9357774	6	53758724	A	26	47	G	0.0007993
rs9357775	6	53766293	G	26	47	A	0.0007993
6:68054293	6	68054293	G	2	15	GT	0.0006952
rs200763254	6	69721029	A	31	53	G	0.0005517
rs2273428	6	70643018	G	28	51	A	0.0002892
rs681089	6	71036220	A	54	29	G	0.0006016

rs9354914	6	71036721	A	28	49	G	0.0008723
rs12524645	6	71036888	A	28	49	G	0.0008723
rs13216351	6	71037050	G	28	49	A	0.0008723
rs13199908	6	71037205	A	28	49	G	0.0008723
rs34123608	6	71037266	G	28	49	GA	0.0008723
rs11757241	6	71037412	T	28	49	C	0.0008723
rs2203664	6	71037500	A	28	49	C	0.0008723
rs718352	6	71039156	T	28	49	C	0.0008723
rs4299788	6	71041772	T	28	49	C	0.0008723
rs1829198	6	71045723	A	28	49	G	0.0008723
rs9364081	6	71049401	T	28	49	A	0.0008723
rs1830499	6	71050871	T	28	49	C	0.0008723
rs9455058	6	71052661	G	28	49	A	0.0008723
rs9342786	6	71053744	A	28	49	G	0.0008723
rs4707782	6	71053876	T	28	49	C	0.0008723
rs9455063	6	71055346	A	28	49	T	0.0008723
rs781115	6	71055814	C	34	55	T	0.0009818
6:74067800	6	74067800	TTATC	5	20	T	0.000897
6:74067804	6	74067804	C	5	20	T	0.000897
rs9446978	6	74385013	T	30	9	A	0.000506
rs13197717	6	79081008	A	48	23	G	0.0004351
rs1452532	6	79091870	C	48	23	A	0.0004351
6:79106259	6	79106259	T	18	2	TG	0.00022
6:79106260	6	79106260	A	18	2	G	0.000203
rs9361404	6	79148758	A	48	23	T	0.0004351
rs374448031	6	79181262	G	2	16	C	0.0003115
6:83237475	6	83237475	C	11	0	CT	0.0007274
rs56164195	6	92582923	A	24	6	AC	0.0005592
rs4707701	6	92584877	T	24	6	C	0.0005592
rs426370	6	95009400	A	13	32	G	0.0009401
rs10944744	6	95190344	A	30	52	G	0.00054
rs9342418	6	95197387	C	28	49	T	0.0008723
rs1022787	6	95202197	G	28	49	A	0.0008723
rs222550	6	95211989	C	28	49	T	0.0008723
rs6905809	6	95278921	C	13	32	A	0.0005036
rs946120	6	97544329	A	55	27	G	0.0001001
rs1737131	6	97558907	C	30	53	G	0.0003072
rs1737132	6	97558912	T	30	53	G	0.0003072
rs1206160	6	97561059	C	29	52	T	0.0002991
rs1206161	6	97561088	C	29	52	T	0.0002991

rs1206146	6	97563222	T	29	52	C	0.0002991
rs25637	6	97563868	CTGTT	29	53	C	0.0001666
rs1206147	6	97565173	G	29	53	A	0.0001666
rs1206148	6	97566464	T	29	51	C	0.0002768
rs1206149	6	97566938	A	27	48	G	0.0008377
rs1206150	6	97568482	G	30	52	A	0.00054
rs1206151	6	97568613	T	30	52	C	0.00054
rs1206152	6	97568963	C	30	52	A	0.00054
rs1206166	6	97572581	C	30	52	T	0.00054
rs1206167	6	97572903	G	30	52	A	0.00054
rs1206168	6	97573678	C	30	52	A	0.00054
rs1206169	6	97573953	T	30	52	C	0.00054
rs2206094	6	97575196	T	30	52	C	0.00054
rs1612775	6	97575423	C	30	52	G	0.00054
rs1766531	6	97576033	C	30	52	T	0.00054
rs1737135	6	97577031	G	30	52	A	0.00054
rs6568746	6	97578265	C	30	51	T	0.0009286
rs1206145	6	97585786	G	30	51	C	0.0009286
rs1206176	6	97587527	A	30	51	T	0.0009286
rs1206177	6	97590623	T	30	51	C	0.0009286
rs1206178	6	97591680	A	30	51	G	0.0009286
rs1206134	6	97601582	T	30	51	A	0.0009286
rs140706	6	97603195	A	30	51	AATTGTT	0.0009286
rs1206137	6	97605268	G	30	51	A	0.0009286
rs1206139	6	97606816	C	30	51	G	0.0009286
rs1766513	6	97607265	G	30	51	T	0.0009286
rs1737140	6	97607266	A	30	51	C	0.0009286
rs1206141	6	97608206	T	30	51	G	0.0009286
rs3061513	6	97612420	CAAAAT	30	51	C	0.0009286
rs1206124	6	97612708	G	30	51	T	0.0009286
rs1209290	6	97613163	C	30	51	T	0.0009286
rs1206125	6	97616717	G	30	51	A	0.0009286
rs1623061	6	97621318	T	30	51	A	0.0009286
rs1623056	6	97621320	T	30	51	C	0.0009286
rs3061515	6	97621860	G	30	51	GCTC	0.0009286
rs1206129	6	97622500	A	29	51	G	0.0005253
rs6926003	6	97624248	C	30	51	T	0.0009286
rs1766470	6	97625034	C	30	51	T	0.0009286
rs1014976	6	97628442	T	30	51	G	0.0009286
rs6913262	6	97629155	C	30	51	T	0.0009286

rs1206170	6	97629381	G	30	51	C	0.0009286
rs1206171	6	97629689	A	30	51	C	0.0009286
rs717020	6	97630619	T	30	51	C	0.0009286
rs1206172	6	97631143	C	30	51	T	0.0009286
rs1209293	6	97632347	T	30	51	C	0.0009286
rs1206174	6	97637114	T	29	49	C	0.0008479
rs1206175	6	97639120	C	30	51	T	0.0009286
rs1206163	6	97642227	T	30	51	C	0.0009286
rs6931754	6	97644798	T	30	51	C	0.0009286
rs6932480	6	97645222	G	30	51	C	0.0009286
rs72941463	6	99484928	A	9	27	G	0.000654
rs34657809	6	102322607	T	33	54	TG	0.0009764
rs1417182	6	102333341	A	55	30	G	0.0006201
6:110302614	6	110302614	A	6	23	AT	0.000429
6:110302616	6	110302616	C	6	23	CTAT	0.000429
rs35422813	6	113672328	G	14	33	GT	0.0007122
rs9387133	6	113680017	T	14	33	C	0.0007122
rs34282960	6	129981642	G	42	19	GA	0.0008809
rs9402159	6	129982039	T	43	19	A	0.0005165
rs9388722	6	129982285	G	42	17	A	0.0002559
rs4482995	6	129984362	A	44	19	T	0.0002974
rs13203844	6	129985147	C	44	19	T	0.0002974
rs12193673	6	129985921	A	44	19	G	0.0002974
rs9398914	6	129986453	G	41	18	C	0.0007885
rs6899523	6	129986588	G	44	19	A	0.0002974
rs6922711	6	129986792	C	44	19	T	0.0002974
rs9402160	6	129987987	G	44	19	A	0.0002974
rs11450417	6	129988030	GC	44	19	G	0.0002974
rs6906744	6	129988135	T	44	19	C	0.0002974
rs78917176	6	129988199	G	43	19	T	0.0005165
rs79069340	6	129988200	A	43	19	T	0.0005165
rs76543028	6	129988201	A	43	19	C	0.0005165
rs9398915	6	129988553	A	44	19	T	0.0002974
rs9402162	6	129988892	C	44	19	G	0.0002974
rs7750456	6	129989333	T	44	19	C	0.0002974
rs4379317	6	129989626	G	44	19	A	0.0002974
rs9388723	6	129997515	G	39	16	A	0.0006105
rs6569619	6	129997885	G	42	17	C	0.0002305
rs6569620	6	129999270	C	42	17	G	0.0002305
6:140587386	6	140587386	G	17	2	T	0.0004014

rs1889552	6	150473849	T	30	10	C	0.0006347
rs4870319	6	150479530	T	30	10	C	0.0006347
rs9384195	6	150480683	G	30	10	A	0.0006347
rs58569771	6	150482412	T	34	12	TA	0.0009752
rs2504068	6	152097697	C	53	27	G	0.0003204
rs1285054	6	152099593	G	56	30	T	0.0003574
rs35801479	6	152100077	G	31	55	GT	0.0001739
rs655459	6	152102282	T	54	28	G	0.0003346
rs112556235	6	155762435	ATTGT	30	52	A	0.00054
rs9322527	6	155900525	T	22	44	A	0.0003557
rs72994685	6	155908174	G	19	40	C	0.0004646
rs72994693	6	155910583	G	19	40	T	0.0004646
rs4869864	6	155912940	C	19	40	T	0.0004646
rs11156031	6	155941911	A	21	41	G	0.0009669
rs12204654	6	155966445	G	21	41	T	0.0009669
rs4870385	6	156007722	G	21	42	T	0.0005665
rs1391661	6	156008329	A	20	42	T	0.0002952
rs72998695	6	156008898	C	20	42	T	0.0002952
rs72998698	6	156008906	T	20	42	C	0.0002952
rs9654608	6	156010248	C	20	42	T	0.0002952
rs9480188	6	156011108	A	21	42	C	0.0005665
rs9478673	6	156012817	T	20	42	C	0.0002952
rs9480190	6	156012974	T	20	42	G	0.0002952
rs111898961	6	156014193	T	20	41	C	0.0005156
rs9397834	6	156015299	T	33	54	C	0.0009764
rs13203482	6	158865022	C	14	1	T	0.0006347
rs9295108	6	160123373	A	12	32	G	0.0004387
rs9295109	6	160123425	C	15	35	T	0.0004795
rs12194483	6	160131463	T	16	35	G	0.000956
rs783176	6	161170835	G	19	3	A	0.0004265
rs35692234	6	161182859	CA	18	3	C	0.0008121
rs2465835	6	161186537	G	18	3	A	0.0008121
rs2465836	6	161187044	A	18	3	G	0.0008121
rs1271391	6	161190851	G	19	3	T	0.0004265
rs62436699	6	161190945	C	17	2	G	0.0004014
rs1652498	6	161191645	G	19	3	T	0.0004265
rs36032205	6	161191858	A	19	3	AG	0.0004265
rs1740447	6	161193779	A	19	3	G	0.0004265
rs1652501	6	161193959	G	19	3	C	0.0004265
rs1782602	6	161199887	A	20	4	G	0.0007606

rs1740438	6	161199900	C	20	4	T	0.0007606
rs1652489	6	161200191	A	19	3	G	0.0004265
rs1652492	6	161201024	A	19	3	G	0.0004265
rs1367216	6	161203454	C	19	3	T	0.0004265
6:161211051	6	161211051	A	16	2	AAAGAGGTGAGAAATCCCTGG	0.0007845
rs1740430	6	161211997	A	19	3	G	0.0004265
rs1652456	6	161215051	T	19	3	A	0.0004265
rs1247572	6	161222490	G	19	3	A	0.0004265
rs1740466	6	161225836	T	19	3	C	0.0004265
rs145349920	6	161225900	TA	19	3	T	0.0004265
rs34026567	6	161225901	G	19	3	T	0.0004265
rs201615835	6	163817782	GTA	4	19	G	0.0006572
6:165997511	6	165997511	A	11	0	T	0.0007274
rs72079749	6	167450849	C	2	15	CAATT	0.0006243
rs12525522	6	169212877	T	17	37	A	0.0006349
rs4360107	6	169222987	C	17	37	A	0.0006349
rs201969550	7	6476788	A	1	13	AT	0.0005601
rs6968854	7	7034807	C	42	19	A	0.0008809
rs6463624	7	7034996	C	42	19	T	0.0008809
rs10276023	7	7037349	A	43	16	T	0.00006043
rs4074750	7	7040309	T	44	20	C	0.0005739
rs4074749	7	7040475	C	44	20	T	0.0005739
rs4074748	7	7040500	C	44	20	T	0.0005739
rs6463625	7	7040611	C	44	20	T	0.0005739
rs6463626	7	7040695	T	44	20	C	0.0005739
rs6463628	7	7041258	A	43	19	G	0.0005165
rs2191318	7	8112014	C	28	49	T	0.0008723
rs4140809	7	8132006	T	42	19	C	0.0008809
rs2058277	7	8132877	T	43	19	C	0.0005165
rs2189906	7	8464974	G	30	10	A	0.0006347
rs2709051	7	9644581	A	15	34	G	0.0008313
rs969419	7	14114798	A	23	5	G	0.0003619
rs55909410	7	20489589	A	21	4	G	0.0004069
rs12667191	7	21401566	T	0	11	A	0.0002715
rs10950846	7	21401732	G	0	11	A	0.0002715
rs10486426	7	21403759	C	0	11	A	0.0002715
rs7780821	7	21413354	G	1	13	A	0.0005059
rs34927723	7	21422925	T	2	16	C	0.0003115
rs9691059	7	21429939	C	4	21	T	0.0001601
rs7810409	7	21438392	C	2	18	T	0.00007463

rs34079228	7	21440066	G	1	13	A	0.0005059
rs79251582	7	21440495	AGT	2	18	A	0.00007463
rs11979569	7	21441999	T	2	18	C	0.00007463
rs11983219	7	21442234	C	2	18	T	0.00007463
rs73083192	7	22512008	C	25	7	T	0.0008092
rs73083195	7	22512641	C	25	7	T	0.0008092
rs73083202	7	22515488	C	25	7	A	0.0008092
rs79921061	7	22516287	C	25	7	A	0.0008092
rs55985142	7	22517874	T	25	7	C	0.0008092
rs56075961	7	22517890	A	25	7	T	0.0008092
rs73085115	7	22519251	T	26	7	C	0.0004518
rs73085119	7	22520389	T	26	7	C	0.0004518
rs73085122	7	22520686	C	26	7	G	0.0004518
rs73085126	7	22521162	A	26	7	G	0.0004518
rs17364835	7	22522178	A	26	7	C	0.0004518
rs73085131	7	22525284	G	25	7	A	0.0008092
7:22818527	7	22818527	T	2	16	A	0.0003115
rs73095715	7	26286936	A	2	15	G	0.0006243
rs4722799	7	28492782	A	12	32	G	0.0004387
rs113362450	7	29594562	T	14	1	C	0.0006347
rs77499142	7	32007106	G	37	14	C	0.0004475
rs117874121	7	37348682	A	18	3	G	0.0008121
rs75702393	7	37401837	AT	33	12	A	0.0005855
7:37401838	7	37401838	ATAT	33	12	A	0.0005855
rs1987067	7	37414537	T	22	5	C	0.0006676
rs2329396	7	39534540	A	51	27	G	0.0009437
rs1476702	7	40924632	G	42	19	A	0.0008809
rs62449443	7	40946739	A	12	0	T	0.0003547
rs6956024	7	46568634	T	20	40	G	0.0008835
rs62452707	7	46570171	A	20	40	T	0.0008835
rs2057869	7	46572992	A	20	40	G	0.0008835
rs723148	7	46577400	C	20	40	A	0.0008835
rs1859519	7	46579424	C	20	40	T	0.0008835
rs12673331	7	46598061	G	20	40	A	0.0008835
rs28783507	7	46600905	A	20	40	G	0.0008835
rs60985485	7	46603966	CAT	20	40	C	0.0008835
rs369251775	7	47136324	G	17	2	GA	0.0004751
rs35341942	7	47151123	T	21	2	TA	0.00002397
rs4557608	7	47154807	C	23	4	T	0.0001119
rs11767755	7	47155356	A	23	4	G	0.0001119

rs2708857	7	48098986	C	0	10	T	0.0005917
7:48259483	7	48259483	C	9	27	A	0.0003604
7:52765162	7	52765162	C	1	14	CACGACTTGTTTTGA	0.0002433
7:52765179	7	52765179	A	1	14	AACAGAACACAATAGAAGT	0.0002433
rs79990624	7	52767350	A	1	13	C	0.0005059
rs17135257	7	52768266	T	1	13	C	0.0005059
rs73121944	7	52782137	T	1	14	C	0.0002433
rs6949581	7	52783916	C	1	14	T	0.0002433
rs73121956	7	52786522	C	1	14	T	0.0002433
rs115950177	7	52786585	A	1	14	C	0.0002433
7:52787115	7	52787115	C	1	12	CATATATACACACATATGTATA	0.0009301
rs9690813	7	52788107	A	1	14	G	0.0002433
rs73121961	7	52788814	C	1	14	T	0.0002433
rs58303022	7	52790941	T	1	14	C	0.0002433
rs73106208	7	52791522	T	1	14	C	0.0002433
rs1404908	7	55262627	T	20	40	C	0.0008835
rs2740763	7	55265354	A	20	40	T	0.0008835
7:57958184	7	57958184	A	3	17	G	0.0007114
7:57958193	7	57958193	G	3	17	T	0.0007114
7:57958195	7	57958195	A	3	17	C	0.0005686
rs140073039	7	61100611	T	27	8	C	0.0005667
7:61911025	7	61911025	A	21	3	AG	0.0001132
7:64556840	7	64556840	A	16	2	AC	0.0007845
rs138614064	7	66054824	A	2	15	G	0.0006952
rs10252133	7	66065059	T	2	15	C	0.0006952
rs143269646	7	66074643	T	1	14	G	0.0002433
rs10235638	7	66396940	T	2	15	C	0.0006243
rs145651753	7	66730116	A	4	21	G	0.0003913
rs11973746	7	66746001	A	4	19	G	0.0005324
rs10551417	7	71563744	G	23	4	GGC	0.00009382
rs59350428	7	75240405	A	26	48	AT	0.000695
rs200981755	7	79359478	T	15	46	TAAATATTTAAAATATTGATTTA	0.0000003411
rs34952316	7	81305715	T	4	19	G	0.0005969
rs71555095	7	81311717	G	4	19	C	0.0005969
rs13227751	7	81312093	C	4	19	G	0.0005969
rs13228550	7	81312150	T	4	19	C	0.0005969
rs34132214	7	81312484	T	4	19	TC	0.0005969
rs35416833	7	81312695	G	4	19	A	0.0005969
rs35697894	7	81312705	C	4	19	T	0.0005969
rs34187512	7	81312906	T	4	19	G	0.0005969

rs1429746	7	85005259	A	40	17	G	0.0006981
rs9640962	7	85005612	C	37	14	A	0.0004475
rs35553509	7	85005734	T	37	14	TC	0.0004475
rs13228617	7	85006563	A	37	14	G	0.0004475
7:85006794	7	85006794	A	36	12	AAT	0.0001705
rs67336694	7	85006795	A	14	38	AT	0.00003336
rs374778279	7	85006798	A	10	35	AAT	0.0000102
rs2435272	7	85007066	T	21	47	G	0.00002923
rs2463468	7	85009349	T	21	47	C	0.00002923
rs12333803	7	85011100	A	37	14	G	0.0004475
rs6951365	7	85012428	A	37	14	T	0.0004475
rs10234933	7	85013956	G	37	14	A	0.0004475
rs2030624	7	85013958	C	21	47	T	0.00002923
rs1403786	7	85014355	C	37	14	T	0.0004475
rs2463475	7	85016000	T	21	47	C	0.00002923
rs2462058	7	85019297	T	21	47	A	0.00002923
rs1589708	7	85021022	T	37	14	C	0.0004475
rs35429293	7	85021116	T	37	14	A	0.0004475
rs34643208	7	85021633	C	37	14	T	0.0004475
rs1403785	7	85021953	A	21	47	G	0.00002923
rs2462057	7	85024037	T	21	47	C	0.00002923
rs1960550	7	85024643	T	21	47	C	0.00002923
rs2077770	7	85026097	T	21	47	A	0.00002923
rs12666995	7	85026991	C	37	14	T	0.0004475
rs2463478	7	85030592	G	21	47	T	0.00002923
rs13236638	7	85033062	T	37	14	C	0.0004475
rs7783195	7	85033683	T	37	14	C	0.0004475
rs1583081	7	85034227	T	21	47	G	0.00002923
rs13227430	7	85035874	C	37	14	A	0.0004475
rs2463480	7	85036389	T	21	47	C	0.00002923
rs2435264	7	85036405	T	21	47	A	0.00002923
rs2463481	7	85037374	G	19	42	A	0.0001482
rs17159908	7	85042188	A	37	14	G	0.0004475
rs148525782	7	85045118	A	21	47	AT	0.00002923
rs112461735	7	85045121	C	21	47	A	0.00002923
rs7794512	7	85045881	A	19	42	G	0.0001482
rs34140074	7	85054102	A	31	52	AT	0.0009496
rs11768400	7	85055535	G	19	39	A	0.0007998
rs1589707	7	85056320	A	19	39	G	0.0007998
rs6465004	7	85056906	C	19	39	T	0.0007998

rs1829902	7	85057001	C	31	52	G	0.0009496
rs35288706	7	85058425	GA	31	52	G	0.0009496
rs34970164	7	85058967	GA	31	52	G	0.0009496
rs12704128	7	85059181	A	31	52	G	0.0009496
rs2115746	7	85063464	G	19	39	A	0.0007998
rs13237273	7	85063736	C	31	52	T	0.0009496
rs10952847	7	85064804	C	31	52	T	0.0009496
rs17159934	7	85070736	A	30	52	C	0.00054
rs17159935	7	85070949	A	30	52	C	0.00054
rs1560315	7	85075150	G	30	52	C	0.00054
rs11762648	7	85077205	T	18	39	C	0.0004141
rs10499888	7	85078623	A	31	52	G	0.0009496
rs73193990	7	85079015	A	31	52	G	0.0009496
rs12704129	7	85079348	A	19	39	T	0.0007998
rs11769386	7	85083869	C	18	39	G	0.0004141
rs35523949	7	85085202	G	31	52	A	0.0009496
rs143204611	7	85086268	TTTAATTATGGTCTGG	30	52	T	0.00054
rs35922536	7	85091656	G	18	39	A	0.0004141
rs35295520	7	85091841	G	30	52	GA	0.00054
rs13240019	7	85097468	T	19	39	C	0.0007998
rs2462051	7	85099770	G	24	45	T	0.0007128
rs2462049	7	85099898	G	25	47	T	0.0004398
rs4728797	7	88664515	C	36	14	T	0.0007756
rs34691685	7	88709608	T	36	13	TA	0.0003885
rs34120249	7	88716652	T	36	14	TA	0.0007756
rs10282116	7	94566679	T	26	48	C	0.0004647
rs10246268	7	94567965	A	27	48	T	0.0008377
rs854717	7	94593140	A	27	48	G	0.0008377
rs11981513	7	94648239	A	57	31	G	0.0003656
7:96459804	7	96459804	C	0	10	A	0.0005917
rs10226446	7	98832759	T	3	18	C	0.0003291
rs2396112	7	109126187	T	27	6	C	0.0000877
rs61005954	7	109925479	C	28	9	G	0.0008484
rs38756	7	109939445	T	28	8	G	0.0003544
rs139582502	7	111450919	G	0	11	A	0.0002715
7:123237974	7	123237974	GTGTGTGTGTA	0	11	G	0.0007003
rs200944680	7	124821228	G	40	17	C	0.0006981
rs201895570	7	124821237	G	40	17	C	0.0006981
rs12532812	7	124859997	A	41	17	C	0.0004047
rs9649011	7	125202672	G	23	47	A	0.0002094

rs2694006	7	125203203	C	22	45	T	0.0002011
rs792416	7	125203939	T	22	46	G	0.0001115
rs5887245	7	125207215	G	23	45	GACTTT	0.0003849
rs2692072	7	125211768	T	23	44	G	0.0006657
rs792160	7	125214809	A	23	44	T	0.0006657
rs792162	7	125216928	T	23	44	C	0.0006657
rs813781	7	125219237	A	23	44	G	0.0006657
rs1833082	7	125219904	G	22	44	A	0.0003557
rs1833083	7	125219953	C	23	44	T	0.0006657
rs34587897	7	125220184	GA	23	44	G	0.0006657
rs35958199	7	125225669	GT	22	43	G	0.0006167
rs792191	7	125225892	C	23	44	G	0.0006657
rs792195	7	125227341	G	23	44	A	0.0006657
rs792196	7	125228133	G	23	44	T	0.0006657
rs792197	7	125228294	T	23	44	C	0.0006657
rs792200	7	125230303	C	23	44	T	0.0006657
rs34672743	7	125231418	A	23	44	AT	0.0006657
rs2524888	7	125234107	G	23	44	A	0.0006657
rs986279	7	125235239	G	23	44	A	0.0006657
rs792171	7	125238557	G	23	44	A	0.0006657
rs792172	7	125238890	G	23	44	C	0.0006657
rs7780182	7	125254448	A	32	53	G	0.0009656
rs7790718	7	125256543	C	32	53	G	0.0009656
rs35729604	7	125262935	C	33	54	CT	0.0009764
rs4731257	7	125266497	A	33	54	G	0.0009764
rs140321097	7	125280738	T	31	52	TATATA	0.0009496
rs17149636	7	126018952	G	8	25	A	0.000871
rs756969	7	126051716	G	8	25	T	0.000871
rs2107740	7	139865545	C	57	32	G	0.0006468
rs11440764	7	139865645	G	50	25	GA	0.000499
rs2189852	7	139870793	A	50	25	G	0.000499
rs1005261	7	139884494	G	50	26	A	0.0008986
rs6464585	7	139896424	T	54	29	A	0.0006016
rs6947196	7	139898709	T	57	32	C	0.0006468
rs58730464	7	139934900	T	9	27	C	0.000654
rs117919318	7	140083539	T	24	4	C	0.00005751
rs10225228	7	141687327	C	0	11	T	0.0002715
7:142196523	7	142196523	AC	0	10	A	0.0005917
7:142196526	7	142196526	G	0	10	C	0.0005917
7:142196533	7	142196533	G	0	10	A	0.0005917

rs13234300	7	144882368	A	27	5	G	0.00002724
rs10228815	7	144882889	G	25	5	T	0.00009108
rs71528967	7	144884334	A	26	5	G	0.00005306
rs7799776	7	144888698	T	26	5	C	0.00005306
rs7788595	7	144893242	A	26	5	C	0.00005306
rs34694182	7	144893767	A	28	7	AT	0.0001348
rs2371843	7	144895554	A	23	4	G	0.0001119
rs2371844	7	144895575	A	23	4	G	0.0001119
rs12703663	7	144895979	G	23	4	A	0.0001119
rs13247774	7	144896721	A	23	4	G	0.0001119
rs71528968	7	144901501	T	26	7	C	0.0004518
rs150195176	7	144911246	T	23	4	C	0.0001119
rs12703667	7	144915712	T	23	5	C	0.0003619
rs139336131	7	144935515	A	16	2	G	0.0007845
rs12703668	7	144938998	C	22	4	T	0.0002148
rs66706284	7	144946452	A	23	4	G	0.0001119
rs73170827	7	144947604	T	18	3	A	0.0008121
rs34223395	7	144950375	T	23	4	C	0.0001119
rs12703672	7	144965537	C	23	5	A	0.0003619
rs11971766	7	149017964	C	49	24	T	0.0004678
7:151092596	7	151092596	CGG	12	0	C	0.0003422
rs6967853	7	152737574	C	6	22	T	0.0007921
rs6967855	7	152737578	C	6	22	T	0.0007921
rs954360	7	152737659	T	6	22	C	0.0007921
rs3898061	7	152737701	A	6	22	G	0.0007921
rs954359	7	152737733	C	6	22	T	0.0007921
rs11388703	7	152738308	T	6	22	TG	0.0007921
rs143970329	7	153517490	G	22	5	T	0.0006676
rs11451890	7	153520700	G	36	14	GT	0.0007756
rs4725514	7	153520711	C	36	14	A	0.0007756
rs2622260	7	153521183	A	37	13	G	0.0002102
rs6464368	7	153521224	G	37	13	A	0.0002102
rs7795045	7	153521738	T	37	13	C	0.0002102
rs13223976	7	154976046	C	26	55	T	0.000005709
rs61507403	7	154976104	G	38	13	A	0.0001161
rs113045737	7	154976533	T	38	13	G	0.0001161
rs12537781	7	154978183	T	35	13	C	0.0006539
rs6597471	7	154978788	G	27	55	C	0.00001197
rs6597473	7	154978979	A	27	55	G	0.00001197
7:154979133	7	154979133	C	27	55	CTT	0.00001197

7:154979135	7	154979135	C	27	55	CTGCCTCATTGG	0.00001197
rs4960695	7	154979628	A	27	55	C	0.00001197
rs4960696	7	154979643	C	27	55	T	0.00001197
rs7782593	7	154979894	C	27	54	G	0.0000235
rs7794634	7	154980152	T	26	55	C	0.000005709
rs7804702	7	154980153	G	27	55	A	0.00001197
rs7790169	7	154980168	A	27	55	G	0.00001197
rs7790310	7	154980243	A	27	55	G	0.00001197
rs10279840	7	154980616	A	26	55	C	0.000005709
rs10274085	7	154980631	C	27	55	T	0.00001197
rs58225134	7	154980766	T	35	13	C	0.0006539
rs35191725	7	154980990	T	27	55	C	0.00001197
rs11975494	7	154981034	G	27	55	C	0.00001197
rs11978131	7	154981062	A	35	13	C	0.0006539
rs4273809	7	154981157	A	27	55	G	0.00001197
rs1465783	7	154981313	A	27	55	T	0.00001197
rs1465784	7	154981331	G	27	55	A	0.00001197
rs11243341	7	154981454	T	27	55	C	0.00001197
rs6597474	7	154981756	T	27	55	G	0.00001197
rs6597475	7	154981760	G	27	55	A	0.00001197
rs117945097	7	154982295	T	21	41	C	0.0009669
rs11982355	7	154984001	C	37	15	A	0.0009053
rs35656216	7	154997261	A	32	57	AC	0.00009501
rs11762374	7	154997735	G	27	52	A	0.00008446
rs35593222	7	154997767	CTG	27	52	C	0.00008446
rs35294146	7	154997789	T	27	52	TTTGC	0.00008446
rs34246874	7	154997831	T	27	52	TGA	0.0002194
7:154997859	7	154997859	AAT	27	52	A	0.00008446
rs11766437	7	154997860	G	27	52	A	0.00008446
rs35092901	7	154997864	G	27	52	GCA	0.00008446
rs34316776	7	154997901	T	27	52	TGA	0.00008446
rs71895605	7	154997965	AGT	28	52	A	0.0001612
rs56388829	7	154997975	CGT	27	52	C	0.00003426
7:154997978	7	154997978	G	27	52	GTGTGTT	0.00008446
rs34406403	7	154998031	C	27	52	T	0.00008446
rs34544564	7	154998178	A	34	57	G	0.0002058
rs12719664	7	154998349	C	32	57	T	0.00009501
rs4960580	7	154998951	T	27	52	C	0.00008446
rs4960581	7	154998952	G	27	52	A	0.00008446
rs11768317	7	154999104	T	27	52	C	0.00008446

rs11763457	7	154999108	G	27	52	A	0.00008446
rs34931632	7	154999339	T	28	51	C	0.0008049
rs13306879	7	155000929	A	26	52	G	0.00004297
rs13231870	7	155001121	C	27	52	G	0.00008446
rs12719667	7	155002319	A	26	52	G	0.00004297
rs12719668	7	155002664	A	27	52	C	0.00008446
rs11766971	7	155002747	C	57	31	T	0.0003656
rs11243344	7	155003395	G	57	31	A	0.0003656
rs11521121	7	155003436	C	57	31	T	0.0003656
rs11515018	7	155003562	C	27	52	T	0.00008446
rs722467	7	155003909	C	57	31	A	0.0003656
rs722465	7	155003983	C	57	31	T	0.0003656
rs722466	7	155004242	A	26	52	G	0.00004297
rs893107	7	155004336	C	57	31	T	0.0003656
rs893106	7	155004371	G	26	52	C	0.00004297
rs893105	7	155004502	A	57	31	G	0.0003656
rs2317169	7	155004567	T	57	31	A	0.0003656
rs2317168	7	155004733	C	57	31	T	0.0003656
rs2317166	7	155004783	A	57	31	G	0.0003656
rs2317165	7	155004833	T	26	52	C	0.00004297
rs2076919	7	155005098	A	57	31	G	0.0003656
rs1106540	7	155005209	G	57	31	A	0.0003656
rs12719690	7	155009761	A	27	48	G	0.0008377
rs6943455	7	155011946	C	31	56	T	0.00009427
rs76207723	7	155018817	T	20	3	C	0.0002211
7:155817937	7	155817937	TGTGTGTGTGGGTGGGTGG[illegible]	15	1	T	0.0003322
rs36015218	7	158069312	G	29	9	GC	0.000837
rs114651407	8	5146438	C	0	10	T	0.0005917
rs4875706	8	6042598	T	22	44	C	0.0003557
rs10106314	8	6043416	G	22	44	T	0.0003557
rs9644299	8	6044660	C	22	44	T	0.0003557
rs9314596	8	6044784	A	22	44	T	0.0003557
rs2552205	8	6044988	C	19	41	G	0.0004457
rs2570648	8	6046026	C	22	44	G	0.0003557
rs2552207	8	6046104	G	22	44	C	0.0003557
rs2570647	8	6046150	C	22	44	A	0.0003557
rs34935564	8	6047368	ATT	22	44	A	0.0003557
rs34662381	8	6047903	A	22	44	G	0.0003557
rs144571109	8	6051763	G	28	49	C	0.0008723
rs2442591	8	6408216	A	2	15	T	0.0006243

rs4840457	8	6408682	C	1	13	T	0.0005059
rs4841262	8	6408683	G	1	13	A	0.0005059
rs10708602	8	6408750	A	4	20	AT	0.0003114
rs12543837	8	6413948	G	4	20	A	0.0003114
rs12546072	8	6414067	A	4	21	G	0.0001601
rs2515504	8	6414226	C	4	21	T	0.0001601
rs17624022	8	6414682	C	4	21	A	0.0001601
rs74417083	8	6415206	A	4	21	G	0.0001372
rs12545089	8	6415257	G	4	21	T	0.0001601
rs370141139	8	6417065	A	4	21	C	0.0001601
rs141510915	8	6417067	CAA	4	21	C	0.0001601
rs67706592	8	6417068	CTA	4	21	C	0.0001601
rs78829996	8	6417364	C	1	14	T	0.0002433
rs73661404	8	6417771	C	4	21	T	0.0001601
rs12547077	8	6417834	C	4	21	T	0.0001601
rs921291	8	6417876	A	4	21	C	0.0001601
rs11996740	8	6418830	A	4	20	G	0.0003114
rs3739391	8	6420534	A	5	20	G	0.0009659
rs3739392	8	6420574	G	4	19	A	0.0005969
rs2442528	8	6423992	G	5	20	A	0.0009659
rs2442532	8	6424286	T	5	20	C	0.0009659
rs2442548	8	6425184	C	5	20	G	0.0009659
rs2515514	8	6427055	A	5	20	T	0.0009659
rs2515516	8	6430340	C	4	19	G	0.0005969
rs2442553	8	6430770	A	4	19	T	0.0005969
rs79031630	8	6431167	G	4	19	GA	0.0005969
rs2442557	8	6433406	C	5	21	G	0.0005184
rs2959778	8	6434185	C	8	26	T	0.0004835
rs2442590	8	6434895	A	1	16	G	0.00005445
8:9113931	8	9113931	C	1	21	T	0.000001269
8:10442680	8	10442680	G	4	20	GT	0.0003114
8:10442682	8	10442682	G	4	20	GTGT	0.0003114
8:10442683	8	10442683	A	4	20	T	0.0003114
rs67367418	8	13660898	T	15	34	TC	0.0008313
rs12676512	8	13661548	T	15	34	C	0.0008313
rs34389480	8	13979844	A	51	27	C	0.0009437
rs10658484	8	13980338	T	51	27	TATC	0.0009437
rs34078873	8	13980364	G	51	27	A	0.0009437
rs59464798	8	18578805	AAGGGGTGGGGAAGGCAC	53	28	A	0.0009334
rs73206580	8	19698505	G	0	10	A	0.0005917

rs4871974	8	22084221	A	25	5	G	0.000102
rs377610860	8	27236131	C	30	10	T	0.0006347
rs12681090	8	28087891	C	21	41	T	0.0009669
rs12678500	8	28088201	T	21	41	C	0.0009669
rs12549755	8	28089596	C	21	41	T	0.0009669
rs11783771	8	28091463	A	21	41	C	0.0009669
rs7816330	8	28093152	T	21	41	G	0.0009669
rs7817972	8	28093589	A	21	41	C	0.0009669
rs6996068	8	28094807	T	21	41	C	0.0009669
rs10099875	8	28095050	C	21	41	A	0.0009669
rs10112778	8	28095291	A	21	41	G	0.0009669
rs7012040	8	28096371	T	21	41	C	0.0009669
rs7010836	8	28096383	A	21	41	G	0.0009669
rs7002331	8	28096888	C	21	41	T	0.0009669
rs7008686	8	28098720	G	24	45	A	0.0007128
rs9886609	8	28099383	A	21	41	C	0.0009669
rs9886614	8	28099667	G	21	41	C	0.0009669
rs9886492	8	28099770	G	21	41	A	0.0009669
rs10098959	8	28100307	C	21	41	A	0.0009669
rs33927304	8	28106349	C	21	41	CG	0.0009669
rs67133221	8	28107702	A	21	41	AGCTT	0.0009669
rs5890733	8	33391839	T	11	0	TG	0.0007086
rs78644235	8	33401574	AT	12	0	A	0.0003422
rs80205709	8	33466818	T	11	0	C	0.0007274
8:37007617	8	37007617	TATACACATATATATCATATA	18	38	T	0.0007166
8:37007644	8	37007644	CATATACATGATATATATCAT	17	39	C	0.0001615
rs2588065	8	54052006	C	56	31	T	0.0006352
rs2588061	8	54053143	G	56	31	T	0.0006352
rs1346115	8	54058042	G	56	31	A	0.0006352
rs2553887	8	54061485	T	56	31	A	0.0006352
rs58532315	8	54063875	T	57	32	TA	0.0006468
rs1365096	8	54078097	A	56	31	T	0.0006352
rs1425911	8	54079284	A	56	31	G	0.0006352
rs148996977	8	55420141	A	52	30	ACTTTCTTTCTTG	0.0007849
rs66772979	8	55453589	A	22	43	G	0.0006167
rs117033707	8	55603134	C	0	11	G	0.0002715
rs75736542	8	55670027	T	0	10	C	0.0005917
rs144337474	8	55716390	C	0	11	G	0.0002715
rs62516880	8	56789408	T	15	1	C	0.0003131
rs11996440	8	56789956	T	15	1	C	0.0003131

rs74464209	8	56792994	G	15	1	C	0.0003131
rs118102319	8	63536600	T	3	18	C	0.0003291
rs10957278	8	64194195	G	20	42	A	0.0002952
rs7462877	8	64201960	C	26	47	G	0.0007993
rs13273312	8	64204622	A	26	47	C	0.0007993
rs7459968	8	64205487	T	28	51	C	0.0002892
rs13250946	8	64206351	C	28	52	G	0.0001612
rs147167979	8	64207845	C	28	52	CACACACAT	0.0001612
rs62509184	8	64209904	C	21	41	G	0.0009669
rs7459685	8	64210501	C	31	54	A	0.0003134
rs7459882	8	64211222	G	29	53	A	0.0001666
rs5891898	8	64212297	T	28	53	TGC	0.00008787
rs13266776	8	64213227	G	29	50	C	0.0009027
rs12549693	8	64213235	C	30	51	A	0.0009286
8:66332579	8	66332579	A	14	0	G	0.00007436
rs35524685	8	67248656	ATG	32	10	A	0.0001942
rs10808755	8	68516681	T	49	25	G	0.00085
rs11781284	8	68517124	T	49	25	C	0.00085
rs7818863	8	68518795	T	49	25	C	0.00085
rs408802	8	68523594	T	49	25	C	0.00085
rs379025	8	68526546	T	49	25	G	0.00085
rs5009543	8	69883776	T	43	18	A	0.0002635
rs57385713	8	70398557	T	42	19	TA	0.0008809
rs34587091	8	70398666	GA	42	19	G	0.0008809
rs2704033	8	70399367	T	42	19	C	0.0008809
rs62518663	8	72723754	C	33	12	T	0.0005855
rs10108922	8	72724089	C	33	12	T	0.0005855
rs28460598	8	72729815	T	33	12	C	0.0005855
rs61670134	8	72739815	C	33	12	G	0.0005855
rs62518682	8	72739855	T	33	12	C	0.0005855
rs139658753	8	72740975	TTCTC	33	12	T	0.0005855
rs11346090	8	73740051	C	21	43	CT	0.0008565
rs60648779	8	75575094	T	0	11	TG	0.0007003
rs34445833	8	80770915	C	54	27	G	0.000181
rs10955649	8	87055718	T	18	38	G	0.0007166
rs77155700	8	89853307	C	4	21	T	0.0001601
rs79954560	8	89859652	T	4	21	C	0.0001601
rs148963199	8	89861554	A	4	20	C	0.0003114
rs141566638	8	89861558	A	4	20	C	0.0003114
rs140507702	8	89861766	C	5	21	T	0.0005184

rs6994018	8	89863807	C	5	21	T	0.0005184
rs78487328	8	89868341	A	4	20	G	0.0003114
rs370170108	8	89868860	T	4	21	C	0.0001601
8:89869753	8	89869753	C	4	21	T	0.0001601
rs148238781	8	89870444	A	4	20	G	0.0003114
rs7841185	8	89871813	A	4	21	G	0.0001601
rs2454338	8	89872798	T	4	20	G	0.0003114
rs2464419	8	89876276	C	4	20	T	0.0003114
rs74305800	8	89879504	A	4	20	G	0.0003114
rs113304118	8	89879992	C	3	19	G	0.0001617
rs2454347	8	89880356	A	4	20	G	0.0003114
rs77504040	8	89881264	G	4	20	A	0.0003114
rs2454340	8	89886314	T	4	20	A	0.0003114
rs7006924	8	89888775	C	4	20	T	0.0003114
rs79707489	8	89889047	T	4	20	A	0.0003114
rs17360267	8	89891244	T	4	20	C	0.0003114
rs1391629	8	89891698	G	4	21	A	0.0001601
rs79888923	8	89896133	A	4	21	T	0.0001601
rs142214846	8	89896412	GA	4	21	G	0.0001601
rs79450712	8	89897429	C	4	21	T	0.0001601
rs79342344	8	89897987	C	4	21	A	0.0001601
rs12545589	8	89898996	G	4	21	A	0.0001601
rs149454363	8	89899264	TTTC	4	20	T	0.0003114
rs78224495	8	89899505	T	4	20	A	0.0003114
rs112165299	8	89900231	A	4	20	G	0.0003114
rs13261365	8	89900493	A	4	19	T	0.0005969
rs12544103	8	89902391	G	4	20	A	0.0003114
rs147814724	8	89902943	TA	4	20	T	0.0003114
rs12545320	8	89903532	C	4	20	T	0.0003114
rs76693810	8	89904752	C	4	20	T	0.0003114
rs75997451	8	89906558	G	4	19	A	0.0005969
rs77576803	8	89907144	G	4	19	C	0.0005969
rs78255467	8	89907313	G	4	19	A	0.0005969
rs79092366	8	89907391	C	4	19	T	0.0005969
rs78252561	8	89909036	G	4	19	C	0.0005969
rs76957720	8	89910229	C	4	19	T	0.0005969
rs201562877	8	89912359	GT	4	19	G	0.0005969
rs141898345	8	89913369	C	4	19	T	0.0005969
rs147503841	8	89913709	G	4	19	A	0.0005969
rs77605958	8	89916995	A	4	19	G	0.0005969

rs77438689	8	89919805	T	4	19	A	0.0005969
rs16888626	8	89924325	A	4	19	T	0.0005969
rs75422852	8	89924438	A	4	19	G	0.0005969
rs10504868	8	89924814	G	4	19	A	0.0005969
rs147372656	8	89925045	A	4	19	ATTGT	0.0005969
rs7003639	8	89930086	C	4	19	T	0.0005969
rs6986236	8	89931641	T	4	19	C	0.0005969
rs7835231	8	89933717	A	4	19	G	0.0005969
rs56032547	8	89938031	C	3	19	T	0.0001662
rs28535979	8	89940602	G	3	19	A	0.0001662
rs12550183	8	89941029	G	3	19	A	0.0001662
rs12550778	8	89941428	C	3	19	T	0.0001662
rs7386824	8	89943382	T	3	19	G	0.0001662
rs7832907	8	89945449	C	3	19	G	0.0001662
rs6981959	8	89948142	T	3	19	C	0.0001662
rs28580081	8	89949868	A	3	19	G	0.0001662
rs7845473	8	89950655	A	3	19	T	0.0001662
rs6980555	8	89951727	T	3	19	A	0.0001662
rs6989186	8	89958433	C	3	19	A	0.0001662
rs28571731	8	89959481	A	3	19	T	0.0001662
rs7005222	8	89961346	G	3	19	A	0.0001662
rs199520323	8	89963649	C	3	19	CT	0.0001662
rs7812413	8	89968423	C	3	19	T	0.0001662
rs1994443	8	89969412	G	5	23	C	0.0001426
rs10095833	8	89971816	G	5	23	C	0.0001426
rs35561412	8	89975371	A	3	19	ATTATTTTAATTCCAT	0.0001662
rs10109745	8	89976774	G	3	19	A	0.0001662
rs10102990	8	89978522	T	3	19	C	0.0001662
rs11267246	8	89983069	T	3	19	TTCTGTGACCTTGGATAGGTTA	0.0001662
rs79230440	8	89984014	A	3	19	G	0.0001662
rs12056804	8	89984466	A	8	26	G	0.0004835
rs2200642	8	89986840	C	3	19	T	0.0001662
rs7842838	8	89987721	A	8	26	C	0.0004835
rs10283336	8	89990848	T	3	19	C	0.0001662
rs1843977	8	89993025	T	8	26	C	0.0004835
rs10429371	8	89993488	C	8	26	T	0.0004835
rs142671358	8	89993687	AT	3	19	A	0.0001662
rs1872892	8	89996790	C	4	20	T	0.0003114
rs4961107	8	89997737	C	2	15	T	0.0006243
rs1487794	8	89998814	G	2	15	A	0.0006243

rs11294484	8	90000670	CA	4	19	C	0.0006572
rs75975346	8	90001592	T	2	15	TAAAC	0.0006243
rs1565148	8	90002125	C	2	15	T	0.0006243
rs62534091	8	90002857	T	2	15	C	0.0006243
rs10504870	8	90003188	A	2	15	G	0.0006243
rs17677468	8	90009992	G	2	15	A	0.0006243
rs56336164	8	90015581	C	2	15	A	0.0006243
rs62532181	8	90021378	T	2	15	C	0.0006243
rs199810687	8	93020618	T	18	42	A	0.0001244
8:93020620	8	93020620	T	18	42	A	0.00007147
rs185504961	8	94163993	C	31	9	A	0.0001497
rs55885345	8	94165158	G	29	9	C	0.0004831
rs11994496	8	94167808	A	29	9	T	0.0004831
rs11997850	8	94168257	A	29	9	G	0.0004831
rs11994583	8	94168269	G	29	9	A	0.0004831
rs58956972	8	94168702	A	29	9	G	0.0004831
rs59244644	8	94171932	G	29	9	C	0.0004831
rs11988363	8	94178447	A	29	9	G	0.0004831
rs9649976	8	94181602	G	28	9	A	0.0008484
rs1348913	8	94184259	A	28	9	G	0.0008484
rs16915696	8	94185302	T	28	9	C	0.0008484
rs368828525	8	94186120	A	28	9	ACTCT	0.0008484
rs16915699	8	94186262	A	28	9	T	0.0008484
rs56105257	8	94189529	T	28	9	C	0.0008484
rs59851916	8	94190441	C	30	8	G	0.0001064
rs16915713	8	94190752	A	30	9	G	0.0002709
rs72662246	8	94191231	A	30	9	T	0.0002709
rs2197673	8	94192043	A	30	9	T	0.0002709
rs1374631	8	94192133	C	30	9	A	0.0002709
rs1374630	8	94192137	A	30	9	G	0.0002709
rs1374629	8	94192318	T	30	9	C	0.0002709
rs148531481	8	94192592	C	30	9	CT	0.0002709
rs16915718	8	94192856	C	30	9	T	0.0002709
rs66461754	8	94193026	A	30	9	G	0.0002709
rs10504927	8	94193643	T	30	9	A	0.0002709
rs61578878	8	94196408	G	30	9	T	0.0002709
rs148082725	8	94983641	GGGGAGA	41	18	G	0.0007388
rs3134244	8	96594225	T	43	19	A	0.0005165
rs4346965	8	101422896	G	4	21	A	0.0001601
rs117769774	8	109102918	A	0	10	G	0.0005917

rs77767830	8	109268396	T	0	10	A	0.0005917
rs28460811	8	115635497	C	32	11	T	0.0007744
rs1156022	8	115641238	C	32	11	T	0.0007744
rs7846669	8	115641841	C	32	11	T	0.0007744
rs10087123	8	115643325	T	32	11	C	0.0007744
rs201102203	8	115644084	A	14	1	C	0.0006677
rs755175	8	120587966	G	56	30	C	0.0003574
rs2875905	8	120591785	C	57	30	A	0.0002015
rs4871360	8	120593737	T	56	30	C	0.0003574
rs74697424	8	122745546	C	31	9	A	0.0001497
rs73324837	8	122747762	G	31	9	A	0.0001497
rs16895935	8	122752496	A	31	9	G	0.0001497
rs4871211	8	122755282	A	1	13	C	0.0005059
rs57880755	8	122756405	G	31	9	C	0.0001497
rs61527129	8	122757085	A	31	9	C	0.0001497
rs12541500	8	122760759	A	1	13	G	0.0005059
rs56339311	8	122767658	A	31	10	G	0.0003611
rs60059140	8	122781270	G	31	9	GA	0.0001497
rs73324878	8	122791268	T	36	12	C	0.0001705
rs74512397	8	122792014	C	1	13	T	0.0005059
rs80348309	8	122799461	A	36	12	T	0.0001705
rs77077293	8	122801499	T	1	13	C	0.0005059
rs279657	8	122804568	T	44	20	C	0.0005739
rs16896103	8	122806329	C	37	14	T	0.0004475
rs12681688	8	122845858	T	31	11	C	0.0008085
rs111416674	8	122846406	T	31	11	C	0.0008085
rs4397409	8	123481414	T	48	24	C	0.0007984
8:124856940	8	124856940	T	23	5	G	0.0003619
8:130622402	8	130622402	G	37	13	A	0.0001083
rs7840584	8	130622403	T	36	14	C	0.0007756
8:130622405	8	130622405	T	30	9	C	0.0002709
rs6578129	8	141656890	G	22	45	A	0.0002011
rs938724	8	141657133	G	22	45	A	0.0002011
rs6997840	8	141658361	T	21	45	C	0.0001015
rs11166988	8	141661663	A	23	45	G	0.0003849
rs2288998	8	142225990	A	36	13	G	0.0003739
9:30098	9	30098	T	11	0	C	0.0007274
rs7042540	9	343859	C	19	40	T	0.0004646
rs10964337	9	2000062	G	10	28	A	0.000853
rs59601268	9	2931638	AG	12	31	A	0.0007705

rs12348338	9	2932407	A	12	31	C	0.0007705
rs10813032	9	2933171	T	5	20	C	0.0009659
rs72685681	9	3998736	G	1	13	T	0.0005059
rs7024586	9	5579213	A	8	28	G	0.000142
rs10738365	9	14512767	T	54	29	A	0.0006016
rs10756563	9	14514644	G	53	28	A	0.00058
rs142454823	9	14656001	GA	18	3	G	0.0008121
rs10810202	9	14656231	C	18	3	G	0.0008121
rs10810203	9	14658112	C	18	3	A	0.0008121
rs7867448	9	20199527	A	20	3	C	0.0002211
rs12237376	9	20205568	C	20	4	G	0.0007606
rs7851999	9	20218125	G	21	4	A	0.0004069
rs10465048	9	20218695	A	21	4	G	0.0004069
rs2034875	9	20219315	A	21	4	G	0.0004069
rs2004294	9	20235881	A	20	4	C	0.0007606
rs71506787	9	23946141	T	10	29	G	0.0004811
rs7850545	9	26671842	C	15	35	G	0.0004795
rs2150989	9	28034155	G	31	11	C	0.0008085
rs1343457	9	28035209	C	31	11	T	0.0008085
9:29708965	9	29708965	G	0	10	A	0.0006636
rs117400746	9	32908910	T	5	20	C	0.0009429
rs1530308	9	32911524	C	8	27	T	0.0002641
rs28572375	9	43702852	A	4	20	C	0.000303
9:66460369	9	66460369	G	0	10	T	0.0005917
rs199578430	9	67328446	A	14	1	G	0.0006347
9:69045346	9	69045346	T	3	17	C	0.0006426
9:74888797	9	74888797	C	0	10	G	0.0005917
rs7041453	9	75715270	T	9	29	C	0.0001986
rs11143515	9	75790576	G	29	8	C	0.0001956
rs4571790	9	75833403	A	14	1	C	0.0006347
rs35750820	9	75835437	CTT	17	2	C	0.0004014
rs4581122	9	75836057	A	17	2	C	0.0004014
rs9657735	9	75838758	T	17	2	C	0.0004014
rs9657736	9	75840034	G	17	2	A	0.0004014
rs28796899	9	75841710	T	19	3	A	0.0004632
rs28833833	9	75841713	A	17	2	T	0.0004307
rs4366143	9	75843433	T	17	2	C	0.0004014
rs6560325	9	75844545	T	17	2	C	0.0004014
rs7863612	9	75845098	T	17	2	G	0.0004014
rs4495499	9	75845497	T	17	2	C	0.0004014

rs4745226	9	75846009	A	17	2	T	0.0004014
rs4510925	9	75846595	G	17	2	T	0.0004014
rs5898293	9	75847204	G	17	2	GA	0.0004014
rs4400456	9	75857034	T	19	3	C	0.0004265
rs67538661	9	75858583	T	19	3	TA	0.0004265
rs4276752	9	75860197	C	19	3	T	0.0004265
rs4282636	9	75906317	A	19	1	C	0.00001683
rs4474087	9	75906822	A	19	1	G	0.00001683
rs10869248	9	75907820	A	16	1	C	0.0001529
rs11143561	9	75926372	T	19	1	C	0.00001683
rs1414159	9	75932545	C	19	1	A	0.00001683
rs35789695	9	75943778	C	19	1	CTCTT	0.00001683
rs1335387	9	75974289	G	19	1	A	0.00001683
rs6560339	9	76001473	A	19	1	G	0.00001683
rs2013971	9	76010849	C	19	1	A	0.00001683
rs1414161	9	76025984	G	19	1	A	0.00001683
rs1034264	9	76027946	T	19	1	C	0.00001683
rs280564	9	76052211	G	19	1	T	0.00001683
rs280573	9	76074106	C	22	5	T	0.0006676
rs2484348	9	76078030	T	16	1	G	0.0001529
rs375914	9	76085223	G	16	1	T	0.0001529
rs278742	9	76094975	A	16	1	T	0.0001529
rs278738	9	76098252	A	16	1	C	0.0001529
rs278733	9	76110042	T	16	1	C	0.0001529
rs383182	9	76115818	C	16	1	T	0.0001529
rs278763	9	76127267	A	16	1	G	0.0001529
rs166767	9	76137194	C	16	1	T	0.0001529
rs278753	9	76141288	G	16	1	A	0.0001529
rs2492826	9	76150936	A	16	1	T	0.0001529
rs278711	9	76154116	A	16	1	G	0.0001529
rs278731	9	76162393	T	16	1	C	0.0001529
rs382417	9	76164453	G	16	1	A	0.0001529
rs451914	9	76164728	G	16	1	A	0.0001529
rs10869268	9	76167213	C	20	4	T	0.0007606
rs275530	9	76174599	G	16	1	A	0.0001529
rs157119	9	76182983	G	16	1	C	0.0001529
rs4744778	9	78553139	C	32	53	T	0.0009656
rs7044428	9	78555026	A	30	51	G	0.0009286
rs7048126	9	78555287	T	30	51	G	0.0009286
rs11144691	9	78556111	G	33	55	A	0.0005652

rs116957104	9	78558948	G	24	6	A	0.0005592
rs11144696	9	78567807	G	42	18	A	0.0004599
rs10869673	9	78580233	T	30	52	C	0.00054
rs11432519	9	78587997	TA	50	24	T	0.0002688
rs142618397	9	78658085	AC	28	9	A	0.0008484
rs1258095	9	78670393	A	28	9	G	0.0008484
rs10869765	9	78971232	G	32	55	A	0.0003175
rs2377637	9	78975569	A	27	5	G	0.00002724
rs11144853	9	78976290	C	27	5	A	0.00002724
rs11144855	9	78977568	A	27	5	G	0.00002724
rs7858410	9	78979879	C	32	55	T	0.0003175
rs78481373	9	78980882	GCA	26	4	G	0.00001463
rs34662513	9	78980960	CTG	43	17	C	0.0000279
rs7862262	9	78981413	G	31	55	C	0.0001739
rs11454957	9	78981576	TA	26	5	T	0.00005306
rs143198459	9	79101832	AAAAC	23	44	A	0.0006657
rs590666	9	79103366	T	29	50	C	0.0009027
rs503222	9	79104807	T	26	49	C	0.0002646
rs608697	9	79105108	A	27	49	G	0.0004874
rs620295	9	79105317	T	27	49	C	0.0004874
rs2476940	9	79105850	T	27	49	C	0.0004874
rs553927	9	79106713	A	54	29	G	0.0006016
rs553806	9	79106751	T	54	29	C	0.0006016
rs2476941	9	79107595	T	28	49	C	0.0008723
rs1085341	9	79107749	T	28	49	C	0.0008723
rs664013	9	79108199	G	28	49	T	0.0008723
rs496253	9	79108382	A	54	29	G	0.0006016
rs619429	9	79111675	A	28	49	C	0.0008723
rs631887	9	79112200	T	54	29	C	0.0006016
rs634064	9	79112688	A	27	49	C	0.0004874
rs570212	9	79112701	T	55	29	C	0.000347
rs566470	9	79113087	G	28	49	A	0.0008723
rs785927	9	79113497	A	28	49	T	0.0008723
rs688268	9	79139590	T	14	33	G	0.0007122
rs639898	9	79150262	G	22	43	A	0.0006167
rs652061	9	79150662	A	22	43	G	0.0006167
rs653026	9	79150903	A	22	43	G	0.0006167
rs478699	9	79258395	T	29	50	C	0.0009027
rs478792	9	79258419	G	29	51	C	0.0005253
rs511040	9	79259592	C	31	53	T	0.0005517

rs370347624	9	79264272	TATC	32	53	T	0.0009656
rs488165	9	79312409	G	26	50	A	0.0001475
rs489069	9	79312491	C	26	50	A	0.0001475
rs684639	9	79312579	A	26	50	T	0.0001475
rs685011	9	79312627	C	26	50	G	0.0001475
rs2487263	9	79313293	A	26	50	G	0.0001475
rs519983	9	79313525	A	26	50	G	0.0001475
rs551262	9	79314660	T	26	50	A	0.0001475
rs620985	9	79318921	A	26	50	G	0.0001475
rs683866	9	79322068	G	26	50	T	0.0001475
rs2307907	9	79324135	A	26	50	ATGACTGTTGCAG	0.0001475
rs530978	9	79324179	G	26	50	A	0.0001475
rs557352	9	79324741	T	26	50	C	0.0001475
rs600859	9	79325558	G	26	50	C	0.0001475
rs626837	9	79326770	A	26	50	G	0.0001475
rs630423	9	79327585	G	26	50	A	0.0001475
rs641088	9	79327670	A	26	50	G	0.0001475
rs537903	9	79327732	T	26	50	C	0.0001475
rs571960	9	79329205	G	26	50	T	0.0001475
rs658548	9	79329257	T	26	50	C	0.0001475
rs659003	9	79329337	G	26	50	T	0.0001475
rs35230302	9	79329462	CA	26	50	C	0.0001475
rs491798	9	79330169	T	26	50	A	0.0001475
rs1854439	9	79335763	T	51	27	C	0.0004482
rs10781372	9	79339715	C	51	26	T	0.0005284
rs4744804	9	79340064	G	51	27	A	0.0009437
rs10746999	9	79342925	T	51	26	C	0.0005284
rs10735649	9	79343326	C	51	27	T	0.0009437
rs7047291	9	80085964	G	31	52	C	0.0009496
rs2120397	9	80101199	G	22	43	A	0.0006167
rs12379817	9	87624702	A	8	26	G	0.0004835
rs12376615	9	87628880	G	8	26	A	0.0004835
rs76736475	9	87631970	A	8	26	T	0.0004835
rs12378021	9	87635540	T	8	27	C	0.0002641
rs113758704	9	87643898	C	6	23	CTCTTCTTTTCTT	0.000429
rs1073049	9	87647100	C	6	23	T	0.000429
rs972604	9	87648729	T	6	23	C	0.000429
rs11791017	9	87654600	G	6	23	C	0.000429
rs56132626	9	87656845	C	6	23	A	0.000429
rs9886759	9	87657757	A	6	23	G	0.000429

rs17334250	9	87657989	C	6	23	A	0.000429
rs11140837	9	87659842	C	6	23	G	0.000429
rs55803300	9	87659954	T	6	23	C	0.000429
rs1038763	9	87662368	C	6	23	A	0.000429
rs55737088	9	87662734	AT	6	23	A	0.000429
rs11140840	9	87663761	T	6	23	G	0.000429
rs17419081	9	87665585	A	6	23	G	0.000429
rs17334480	9	87665823	T	6	23	C	0.000429
rs7047067	9	87666242	T	6	23	C	0.000429
rs7019906	9	87666637	T	6	23	C	0.000429
rs12338789	9	87668792	C	6	23	A	0.000429
rs57018861	9	87669383	T	6	23	C	0.000429
rs60316689	9	87669393	G	6	23	A	0.000429
rs55936438	9	87670756	G	6	23	A	0.000429
rs1490402	9	87673253	C	6	23	T	0.000429
rs58921047	9	87673578	TA	4	18	T	0.0009654
rs7027411	9	87673780	G	6	23	A	0.000429
rs7032009	9	87674914	T	6	23	A	0.000429
rs12350350	9	87676170	G	6	23	C	0.000429
rs58901408	9	87676180	G	6	23	A	0.000429
rs55801664	9	87676560	C	6	23	T	0.000429
rs56860108	9	87677422	G	6	23	GTTCT	0.000429
9:87677427	9	87677427	G	6	23	T	0.000429
rs1490401	9	87677645	C	6	23	T	0.000429
rs72739980	9	87678321	C	6	23	G	0.000429
rs56128419	9	87679040	A	6	23	G	0.000429
rs59715785	9	87679240	A	6	23	G	0.000429
rs72739981	9	87679596	A	6	23	G	0.000429
rs73649505	9	87679744	A	6	23	C	0.000429
rs7862675	9	87681108	G	6	23	A	0.000429
rs56320974	9	87683008	C	6	23	G	0.000429
rs56332820	9	87685187	C	6	23	G	0.000429
rs1982841	9	87686259	C	6	23	T	0.000429
rs4373600	9	87686621	C	6	22	T	0.0007921
rs4642728	9	87686692	C	5	23	T	0.0001378
rs4475585	9	87686716	A	5	23	C	0.0001307
rs55860493	9	87687029	A	6	23	G	0.000429
rs9695661	9	87688329	T	6	23	C	0.000429
rs7024408	9	87688748	C	6	23	T	0.000429
9:87690060	9	87690060	C	6	23	A	0.000429

rs140872421	9	87691466	C	6	23	T	0.000429
rs369543529	9	87691952	A	6	23	G	0.000429
rs147238133	9	87692263	A	6	23	G	0.000429
rs56081839	9	87692517	G	6	23	A	0.000429
rs139986476	9	87693567	A	6	23	AAAG	0.000429
rs7038454	9	87694454	G	6	23	A	0.000429
9:88283892	9	88283892	A	2	15	G	0.0006243
rs12343261	9	89205672	T	41	16	C	0.000199
rs10780772	9	89206095	A	41	15	T	0.00009342
rs13298180	9	89213222	T	43	20	C	0.0009741
rs11315810	9	89213749	C	44	19	CT	0.0002974
rs1010176	9	89217172	G	46	22	A	0.0006885
rs34351553	9	89219656	G	46	22	A	0.0006885
rs868823	9	89219781	G	46	22	A	0.0006885
rs200577254	9	89223235	G	46	22	A	0.0006885
rs7019542	9	89225229	T	46	22	C	0.0006885
rs12338699	9	89227192	T	48	22	G	0.0002295
rs12338628	9	89227242	T	50	26	C	0.0008986
rs11788864	9	93445598	T	3	18	C	0.0003291
rs35310535	9	93521114	A	3	17	G	0.0006426
rs11794611	9	93534941	G	3	19	A	0.0001662
rs10993694	9	93559029	C	34	12	G	0.0003362
rs10821506	9	93560316	T	34	12	C	0.0003362
rs10821507	9	93561614	T	34	12	G	0.0003362
rs34221447	9	93563884	T	34	12	C	0.0003362
rs9409462	9	94546619	T	10	28	C	0.000853
rs62565672	9	94629940	C	0	11	G	0.0002355
rs2698999	9	102735865	A	58	30	C	0.0001111
rs2791335	9	102736844	A	57	30	G	0.0002015
rs2791336	9	102739446	C	58	30	G	0.0001111
rs2791337	9	102741443	C	56	29	T	0.000146
rs7862460	9	102748888	G	58	30	C	0.0001111
rs12685650	9	102749175	G	58	30	C	0.0001111
rs10819714	9	102751188	T	58	30	C	0.0001111
rs7874449	9	102752182	A	58	30	G	0.0001111
rs10988934	9	102752686	T	58	30	C	0.0001111
rs6478993	9	102754426	A	23	44	G	0.0006657
rs58414815	9	102756707	T	57	30	TCTCA	0.0002015
rs10760711	9	102766119	C	57	30	G	0.0002015
rs10819715	9	102769559	C	58	30	G	0.0001111

rs2209082	9	102770884	C	58	30	T	0.0001111
rs145295727	9	102772214	A	57	30	AGGAAAATTCTTTTTTCTAAACT	0.0002015
rs10988945	9	102776091	T	58	30	A	0.0001111
rs7870585	9	102779098	A	58	30	G	0.0001111
rs4742779	9	102781837	C	58	29	A	0.00005841
rs1578646	9	102798031	G	58	30	A	0.0001111
rs2093696	9	102799758	A	58	30	G	0.0001111
rs35493819	9	102803899	A	58	30	G	0.0001111
rs7858606	9	102805864	T	57	30	C	0.0002015
rs4743379	9	102807181	A	58	30	C	0.0001111
rs4743380	9	102807279	G	58	30	A	0.0001111
rs3052013	9	102812595	C	58	30	CAT	0.0001111
rs41346545	9	102816751	A	58	30	C	0.0001111
rs4743381	9	102817835	C	58	30	T	0.0001111
rs4743382	9	102817841	T	58	30	C	0.0001111
rs942163	9	102820002	C	58	30	T	0.0001111
rs7042194	9	102821369	A	58	30	G	0.0001111
rs7027536	9	102829705	G	58	30	C	0.0001111
rs10988961	9	102829806	A	58	30	G	0.0001111
rs2900234	9	102830208	T	57	29	C	0.0001082
rs146685377	9	102831618	A	56	29	AAAG	0.0001959
rs7036518	9	102839699	C	58	30	T	0.0001111
rs7047092	9	102839709	A	58	30	G	0.0001111
rs7018981	9	102842941	C	58	30	T	0.0001111
rs1342147	9	102844842	A	58	30	C	0.0001111
rs1529191	9	102845908	A	58	30	G	0.0001111
rs4517187	9	102850723	A	58	30	C	0.0001111
rs10988968	9	102852401	A	57	30	T	0.0002015
rs10988969	9	102852444	G	58	30	A	0.0001111
rs1361668	9	102856306	C	57	30	G	0.0002015
rs72548315	9	102857088	TA	56	30	T	0.0001878
rs2181579	9	102863821	A	58	30	C	0.0001111
rs12552646	9	102868475	T	58	29	C	0.00005841
rs10988973	9	102869846	A	58	30	G	0.0001111
rs1539297	9	102871586	G	58	30	A	0.0001111
rs10988974	9	102871835	T	58	30	C	0.0001111
rs4743386	9	102872241	A	58	30	G	0.0001111
rs34439340	9	102875543	G	57	30	GTTA	0.0002015
rs9408993	9	102877633	G	57	30	A	0.0002015
rs2057476	9	102878742	C	57	29	G	0.0001082

rs2485746	9	102879336	A	24	45	G	0.0007128
rs846754	9	102881455	A	57	30	T	0.0002015
rs846755	9	102881777	G	57	30	C	0.0002015
rs846759	9	102885911	A	56	30	G	0.0003574
rs846762	9	102888321	C	56	30	T	0.0003574
rs1535667	9	102894255	T	57	30	C	0.0002015
rs1099908	9	102904991	C	57	30	T	0.0002015
rs2476439	9	102908076	T	57	30	G	0.0002015
rs846763	9	102908721	A	57	30	G	0.0002015
rs11333350	9	102911331	C	56	27	CA	0.00002655
rs846766	9	102912720	A	57	30	G	0.0002015
rs863668	9	102926619	G	57	30	A	0.0002015
rs1099910	9	102940575	C	57	30	A	0.0002015
rs143374251	9	102942626	GT	54	30	G	0.000587
rs7856844	9	102950726	T	57	30	G	0.0002015
rs10819739	9	102957705	A	57	30	C	0.0002015
rs71370984	9	102957775	A	56	31	AT	0.0005608
rs1999877	9	102973719	C	57	30	A	0.0002015
rs1014651	9	102976644	G	58	30	A	0.0001111
rs2787364	9	102979244	A	57	30	G	0.0002015
rs1572607	9	102980669	C	57	30	T	0.0002015
rs1338118	9	102982419	T	57	30	A	0.0002015
rs2806681	9	102984967	G	57	30	A	0.0002015
rs2806684	9	102992336	C	57	30	T	0.0002015
rs2491096	9	102998581	T	57	30	G	0.0002015
rs2806685	9	102999032	A	57	30	C	0.0002015
rs2787396	9	103003632	C	57	31	A	0.0003656
rs2806687	9	103005815	T	56	30	C	0.0003574
rs2806688	9	103006156	A	57	30	G	0.0002015
rs2787393	9	103009415	G	57	30	T	0.0002015
rs10559514	9	103009954	T	30	53	TAAA	0.0005124
rs142007666	9	103011275	G	31	54	GCTT	0.0003134
rs112776912	9	103012828	GTGTT	33	56	G	0.0003196
rs1338121	9	103015018	C	54	29	T	0.0006016
rs9408998	9	103016838	C	54	29	T	0.0006016
rs7865678	9	103017106	A	54	29	G	0.0006016
rs2806689	9	103021534	C	54	29	T	0.0006016
rs2806690	9	103021647	A	54	29	G	0.0006016
rs1338126	9	103038653	C	54	29	G	0.0006016
rs2787382	9	103039197	T	54	29	C	0.0006016

rs16920204	9	104097900	G	17	38	A	0.0003648
rs1341763	9	104127127	C	19	39	T	0.0007998
rs10989484	9	104132088	G	20	40	A	0.0008835
rs1572983	9	104133628	C	20	40	T	0.0008835
rs1891218	9	104134777	C	20	40	T	0.0008835
rs10819923	9	104137818	G	19	39	A	0.0007998
rs1226146	9	104140630	C	20	40	A	0.0008835
rs1226149	9	104147083	C	20	40	T	0.0008835
rs10118966	9	104152155	C	20	40	T	0.0008835
rs10989486	9	104153155	C	19	39	G	0.0007998
rs10760794	9	104159217	T	51	27	C	0.0009437
rs963584	9	104159859	T	51	27	C	0.0009437
rs12554908	9	105697991	C	17	1	T	0.00007397
rs28473692	9	105714399	C	17	1	T	0.00007397
rs72742480	9	105727753	A	17	1	G	0.00007397
rs170621	9	107803370	G	30	51	A	0.0009286
rs2812301	9	108208066	A	18	38	G	0.0007166
rs59463718	9	108208647	C	18	38	CTTAT	0.0007166
rs2771039	9	108221252	A	18	38	G	0.0007166
rs2812306	9	108221369	A	18	38	T	0.0007166
rs62575095	9	108224124	A	18	38	G	0.0007166
rs145768242	9	108228282	A	18	38	ACT	0.0007166
rs2771044	9	108242839	G	18	38	C	0.0007166
rs7865377	9	108256602	A	18	38	G	0.0007166
rs7865160	9	108256610	T	18	38	C	0.0007166
rs62575103	9	108284699	A	18	38	G	0.0007166
rs62575104	9	108290049	T	18	38	C	0.0007166
rs74442717	9	108291374	T	18	38	G	0.0007166
rs57432946	9	108305782	C	18	38	G	0.0007166
rs149664708	9	108309573	TGTGTTTG	18	38	T	0.0007166
rs4742651	9	108315424	T	18	38	C	0.0007166
rs62575112	9	108328228	A	18	38	G	0.0007166
rs4742653	9	108328588	C	18	38	A	0.0007166
rs76375684	9	108329464	A	18	38	C	0.0007166
rs7024265	9	108331234	A	18	38	G	0.0007166
rs4742953	9	108335958	C	18	38	G	0.0007166
rs62575116	9	108339846	C	18	38	A	0.0007166
rs17309137	9	108348138	A	18	38	G	0.0007166
rs17309319	9	108350328	A	18	38	T	0.0007166
rs7037972	9	108351816	T	18	38	A	0.0007166

rs59750891	9	108353179	A	18	38	AC	0.0007166
rs11399147	9	108394700	TA	20	43	T	0.0001658
rs12349610	9	109404422	A	3	18	G	0.0003291
rs12350586	9	109404541	A	3	18	G	0.0003291
rs2417753	9	109405667	G	4	19	A	0.0005969
rs10978580	9	109410929	T	4	22	C	0.00008114
rs10816411	9	109411613	T	4	22	C	0.00008114
rs7859930	9	109412031	T	4	22	A	0.00008114
rs12343926	9	109412469	C	2	17	A	0.0001534
rs10816412	9	109412764	T	4	22	G	0.00008114
rs10816413	9	109413318	A	5	23	G	0.0001426
rs56108151	9	109514426	G	3	17	A	0.0006426
rs149747210	9	109519072	C	1	14	A	0.0002433
rs77073159	9	109521642	C	1	14	T	0.0002433
rs62567160	9	111122958	A	27	8	C	0.0006326
9:115158488	9	115158488	G	0	10	T	0.0005917
9:115158492	9	115158492	A	0	10	C	0.0005917
rs7857688	9	116887845	A	5	21	G	0.0005184
rs17237455	9	117483259	C	8	25	A	0.000871
rs62564879	9	119790692	A	0	11	G	0.0002715
9:126708698	9	126708698	CACACACACACACACACA	12	0	C	0.0003422
9:126708699	9	126708699	C	12	0	A	0.0003422
rs3012790	9	137807802	G	18	39	C	0.0004141
rs2989724	9	137807917	T	15	36	C	0.0002717
rs2989725	9	137808005	C	18	39	A	0.0004141
rs11103648	9	137941317	A	12	0	G	0.0003422
rs564676	9	137942526	C	43	19	T	0.0005165
rs11103650	9	137942656	A	12	0	G	0.0003422
rs684270	9	137943925	G	42	19	A	0.0008809
rs35973213	9	137960753	G	14	0	A	0.00007436
rs75796057	9	137961094	A	15	0	G	0.00003433
rs1679644	9	137962381	A	24	4	T	0.00005751
rs112072815	10	1990334	T	0	14	C	0.00002513
rs11250953	10	1991144	C	0	15	G	0.0000112
rs17157353	10	1992873	T	0	15	C	0.0000112
rs11250954	10	1993586	T	0	15	G	0.0000112
10:2362186	10	2362186	C	4	20	CACACA	0.0003114
rs138359959	10	3318900	C	16	1	CTCAAACCCAAATCCCGAAACA	0.0001802
rs34380691	10	6388161	G	27	8	GA	0.0006326
rs7074425	10	6984652	A	5	23	T	0.0001426

rs7087785	10	6984954	A	6	23	C	0.000429
rs10905091	10	7049985	T	27	7	C	0.0002486
rs77372529	10	7066921	A	22	4	G	0.0002148
rs2277225	10	7244675	A	14	36	G	0.0001265
rs7920088	10	7250148	T	14	36	C	0.0001265
rs61835084	10	7250882	T	14	36	C	0.0001265
rs61835085	10	7250885	T	14	36	C	0.0001265
10:10105662	10	10105662	C	28	8	CA	0.0002958
10:10105663	10	10105663	C	28	7	A	0.0001108
rs78762327	10	10750115	A	11	0	G	0.0007274
10:13898218	10	13898218	G	52	28	GTGTGTGTGTGTGTGT	0.0009847
rs2883453	10	16008550	G	32	54	A	0.0005601
rs4748234	10	16029510	C	29	50	T	0.0009027
rs11305639	10	16029660	A	28	50	AT	0.0005077
rs950220	10	16052784	C	32	55	A	0.0003175
rs10752031	10	16060056	G	30	51	T	0.0009286
rs151110139	10	18234143	A	21	4	ATTTTC	0.0004069
rs35032081	10	18237482	G	18	2	GA	0.000203
rs12242670	10	18238054	T	17	2	C	0.0004014
rs35598022	10	18245990	G	19	2	T	0.0001015
rs11011704	10	18247677	T	19	2	G	0.0001015
rs11593092	10	18248136	A	19	2	G	0.0001015
rs7074683	10	18249973	A	19	2	G	0.0001015
rs12260894	10	18252211	G	19	2	C	0.0001015
rs12761871	10	18252449	A	19	2	G	0.0001015
rs61022323	10	18256517	G	19	2	A	0.0001015
rs12263430	10	18257612	G	19	2	A	0.0001015
rs11592271	10	18262041	C	19	2	A	0.0001015
rs76522551	10	19411160	A	14	1	G	0.0006347
rs12358258	10	20311687	T	27	8	C	0.0006326
rs786871	10	26765474	G	36	14	T	0.0007756
rs12412826	10	26778606	A	2	15	G	0.0006243
rs112903628	10	26780794	A	2	15	G	0.0006243
rs72803274	10	26782556	A	2	15	G	0.0006243
rs2039976	10	29192769	C	29	8	T	0.0001956
rs2039977	10	29192854	C	29	8	T	0.0001956
rs61838422	10	29194184	C	21	4	T	0.0004069
rs11007308	10	29196847	C	29	9	T	0.0004831
rs7084821	10	29200793	G	30	8	A	0.0001064
rs2047242	10	29201860	A	30	8	G	0.0001064

rs2047241	10	29201960	G	30	9	A	0.0002709
rs35575195	10	29203067	A	5	20	C	0.0009659
10:30752552	10	30752552	T	0	11	C	0.0007003
rs12773194	10	34954301	T	23	44	A	0.000608
rs61843267	10	35567246	G	25	7	C	0.0008092
rs61850546	10	35808629	A	25	7	G	0.0008092
rs200789076	10	39102188	T	1	13	A	0.0005059
10:42711053	10	42711053	A	22	3	T	0.00005724
rs201165034	10	47381269	A	27	8	G	0.0006326
rs72792266	10	49518130	A	15	1	G	0.0003131
rs12767241	10	53038459	A	29	9	G	0.0004831
rs74132547	10	53407805	T	0	10	C	0.0005917
rs2264662	10	54786789	G	29	10	A	0.0009776
rs4935388	10	54858559	T	36	14	G	0.0007756
rs4935389	10	54858786	A	36	14	T	0.0007756
rs12241649	10	54861336	G	36	14	A	0.0007756
rs10218930	10	54865321	G	35	13	A	0.0006539
rs11003388	10	54865331	T	35	13	A	0.0006539
rs11003390	10	54867026	G	36	14	A	0.0007756
rs10824889	10	54868196	C	36	14	T	0.0007756
rs4007215	10	56337604	A	42	9	AAATAAATG	0.0000001416
rs72051960	10	56337612	G	34	5	GAATA	0.0000001264
10:56982508	10	56982508	C	44	20	T	0.0009594
rs10825992	10	58948207	G	17	37	T	0.0006349
rs10825993	10	58948297	A	16	36	C	0.0005556
rs10825994	10	58948493	A	16	36	C	0.0005556
rs10763457	10	58949188	C	16	36	G	0.0005556
rs35674272	10	58951433	C	16	36	T	0.0005556
rs7085570	10	58952843	G	16	36	C	0.0005556
rs2393225	10	58954023	A	16	36	C	0.0005556
rs11005689	10	58957056	A	16	36	G	0.0005556
10:61618959	10	61618959	A	0	12	T	0.0001421
10:66724249	10	66724249	G	22	3	A	0.00005724
rs1462829	10	67978350	T	19	2	C	0.0001015
rs6480148	10	67979752	A	19	2	G	0.0001015
rs17835285	10	67981102	C	19	2	T	0.0001015
rs138734267	10	67983620	G	19	2	GT	0.0001015
rs56350854	10	67985736	C	19	2	G	0.0001015
rs78558138	10	67989485	A	19	2	AT	0.0001015
rs72797255	10	67990772	T	19	2	C	0.0001015

rs72797256	10	67991072	C	19	2	T	0.0001015
rs72797257	10	67991105	C	19	2	T	0.0001015
rs72797258	10	67991893	A	19	2	C	0.0001015
rs72797259	10	67991947	T	19	2	G	0.0001015
rs147898764	10	67992698	A	19	2	G	0.0001015
rs141673860	10	67992739	A	19	2	C	0.0001015
rs72797265	10	67993744	G	19	2	T	0.0001015
rs72797267	10	67993887	A	19	2	G	0.0001015
rs72797269	10	67994426	A	19	2	G	0.0001015
rs6480151	10	67994452	T	34	12	A	0.0003362
rs67876961	10	67994904	G	34	12	A	0.0003362
rs72797275	10	67997207	T	19	2	C	0.0001015
rs118051852	10	70666068	C	0	10	G	0.0005917
rs117613158	10	70675979	A	0	10	G	0.0005917
10:70689049	10	70689049	CT	2	17	C	0.0003704
10:70689077	10	70689077	C	0	10	CTCTT	0.0005917
rs12416545	10	70689108	C	0	10	T	0.0005917
rs143763855	10	70726465	A	0	10	G	0.0005917
rs188524170	10	70759912	T	0	10	C	0.0005917
rs142684333	10	70769102	G	0	10	A	0.0005917
rs117103198	10	70778071	C	0	11	T	0.0002715
rs138629322	10	70779248	C	0	11	G	0.0002715
rs79346133	10	70783648	T	0	11	C	0.0002715
rs114000842	10	70785490	G	0	11	T	0.0002715
10:70798818	10	70798818	T	0	10	TCTTTC	0.0005917
rs10823635	10	72660331	T	32	11	C	0.0004661
rs146991153	10	72665149	C	15	1	CT	0.0003131
rs59844598	10	72665926	T	28	8	C	0.0003544
rs78785419	10	72666056	A	28	8	AG	0.0003544
rs827344	10	72667037	G	42	17	C	0.0002305
rs61851192	10	72668090	G	28	8	A	0.0003544
10:78931156	10	78931156	T	12	0	TGC	0.0003422
rs11188859	10	82964158	A	0	10	G	0.0005917
10:83017314	10	83017314	C	9	31	T	0.00002809
rs9664118	10	85056923	A	0	10	G	0.0006636
rs11200564	10	85536772	A	26	7	G	0.0004518
rs55637662	10	95154278	T	3	17	C	0.0007114
rs35145082	10	108418072	TA	13	0	T	0.0001585
rs12775106	10	108600601	T	39	17	A	0.0006713
rs67682061	10	108600620	T	36	14	TA	0.0007721

rs140471525	10	109403372	A	11	0	G	0.0007274
rs12220594	10	112997939	C	11	0	T	0.0007274
rs10787977	10	121298412	T	1	13	C	0.0005059
rs7895986	10	129234522	G	21	41	T	0.0009669
rs1887097	10	129236512	G	21	44	A	0.0001836
rs2275052	10	129237580	T	14	36	C	0.0001265
rs11815834	10	129238058	G	14	36	A	0.0001265
rs56042331	10	129764846	A	1	13	G	0.0005059
rs201546056	11	133399	G	11	0	C	0.0007228
rs17225881	11	4699499	G	13	0	A	0.00016
rs12363468	11	6074049	G	1	13	A	0.0005059
rs7121476	11	8258184	G	48	23	A	0.0004351
rs439667	11	8259358	C	38	12	G	0.00005024
rs55925157	11	8273447	A	26	46	AGTGTGTGGTGG	0.0007601
rs11041827	11	8274699	T	29	51	C	0.0005253
rs4256972	11	8275004	T	29	51	C	0.0005253
rs11041828	11	8275294	T	29	51	C	0.0005253
rs4237770	11	8275835	C	29	51	T	0.0005253
rs369776490	11	8276156	T	29	51	TATGGTCCCTCCCCACC	0.0005253
rs373931277	11	8276174	A	29	51	AGGCTGGC	0.0005253
rs149239827	11	8276184	T	29	51	TGG	0.0005253
rs1454439	11	8276219	C	29	51	A	0.0005253
rs4758055	11	8277435	C	35	55	G	0.0005984
rs12802521	11	8277575	G	34	56	T	0.0005669
rs12797411	11	8277594	G	34	57	A	0.0002058
rs71463719	11	8277624	G	57	31	A	0.0003656
rs11041830	11	8277807	A	57	31	C	0.0003656
rs10840009	11	8323539	C	13	34	A	0.0001896
rs4758334	11	8330158	A	14	34	C	0.0004075
rs897104	11	8330616	A	14	34	G	0.0004075
rs10743067	11	8330987	A	14	33	G	0.0007122
rs202017636	11	9624708	CT	22	44	C	0.0009242
rs1791839	11	21178709	A	19	39	G	0.0007998
rs2593688	11	22389031	C	24	46	T	0.0004131
rs1374720	11	22389827	A	24	46	T	0.0004131
rs2593685	11	22391419	C	24	46	T	0.0004131
rs2665677	11	22391534	G	24	46	A	0.0004131
rs764021	11	22393124	C	24	46	T	0.0004131
rs764022	11	22393157	C	24	46	G	0.0004131
rs2248436	11	22397739	A	24	46	C	0.0004131

rs2665699	11	22402276	A	24	46	G	0.0004131
rs2665704	11	22408655	G	22	45	A	0.0002011
rs2665705	11	22408803	T	22	45	C	0.0002011
rs721840	11	22411865	C	22	45	T	0.0002011
rs721841	11	22411969	C	22	45	G	0.0002011
rs2593649	11	22413033	T	22	45	C	0.0002011
rs2665712	11	22414861	C	22	45	G	0.0002011
rs2665713	11	22417107	A	22	45	G	0.0002011
rs1447504	11	22417712	A	22	45	T	0.0002011
rs726858	11	22418220	T	22	45	G	0.0002011
rs726859	11	22418570	A	22	45	G	0.0002011
rs2593651	11	22420611	G	22	45	A	0.0002011
rs2593652	11	22421015	T	22	45	C	0.0002011
rs2593653	11	22422503	G	21	45	C	0.0001015
rs111974052	11	22423006	A	22	45	AATGACTATTTG	0.0002011
rs2593657	11	22423166	T	22	45	C	0.0002011
rs35078276	11	22423689	C	22	45	T	0.0002011
rs34754498	11	22423691	G	22	45	T	0.0002011
rs34550272	11	22423693	C	22	45	T	0.0002011
rs4923000	11	22423752	A	22	45	C	0.0002011
rs2593660	11	22424782	C	22	46	A	0.0001115
rs5790261	11	22425340	TG	21	45	T	0.0001015
rs2593663	11	22428384	C	22	45	T	0.0002011
rs2665718	11	22428882	G	22	45	A	0.0002011
rs3106185	11	22435111	C	22	45	A	0.0002011
rs71445462	11	23567476	TA	8	26	T	0.0004835
rs7131015	11	24787901	T	17	2	G	0.0004014
rs11028152	11	24788677	A	17	2	G	0.0004014
rs1065138	11	34379280	A	0	13	G	0.00005596
rs150240560	11	34402655	T	0	13	TC	0.00005596
rs10160662	11	34405010	A	0	13	C	0.00005596
rs11032644	11	34410017	T	0	13	C	0.00005596
rs12276589	11	34414176	A	0	13	T	0.00005596
rs12271710	11	34415592	A	0	13	G	0.00005596
11:37161690	11	37161690	TTTAA	26	48	T	0.0004647
11:38232883	11	38232883	AAGAAAAGAAAAGAAAAGA	0	11	A	0.0002715
rs117740926	11	40776914	T	0	11	C	0.0002715
11:45693756	11	45693756	GT	28	49	G	0.0008723
11:45693757	11	45693757	T	28	49	G	0.0008723
11:45693758	11	45693758	GTGTGTGT	28	49	G	0.0008723

rs201302583	11	45693759	T	26	47	TGA	0.0007993
11:46733346	11	46733346	A	4	19	T	0.0006572
11:48890092	11	48890092	C	2	16	T	0.0003115
rs200512388	11	49292241	AAC	35	13	A	0.0006539
rs1164663	11	49294723	T	35	13	A	0.0006539
rs1164664	11	49295542	G	35	13	T	0.0006539
rs1164665	11	49295630	C	35	13	T	0.0006539
rs35301434	11	49511910	CT	36	14	C	0.0007721
rs7945981	11	50189879	G	28	9	A	0.0006921
11:55471124	11	55471124	CACAG	44	20	C	0.0005739
rs4939191	11	57988941	A	12	31	G	0.0007705
rs200541435	11	61702896	A	4	19	AAAAAAAAAAAAAAAG	0.0005969
rs10897275	11	62203865	A	42	19	G	0.0008809
rs9645690	11	62206288	T	42	19	C	0.0008809
rs56299751	11	62208039	A	43	19	G	0.0005165
rs11231093	11	62210822	A	42	17	G	0.0002559
rs10897278	11	62213068	A	43	19	G	0.0005165
11:71910931	11	71910931	T	41	18	TGTG	0.0003968
rs377432	11	72358817	T	26	7	C	0.0004518
rs459854	11	72359259	A	26	7	T	0.0004518
rs163693	11	72360787	T	26	7	C	0.0004518
rs17369284	11	81022917	G	0	10	A	0.0005917
rs56177670	11	81023418	A	0	10	G	0.0005917
rs78577737	11	81026664	T	0	10	C	0.0005917
rs55822564	11	81030553	G	0	10	A	0.0005917
rs55811617	11	81030577	T	0	10	C	0.0005917
rs17369881	11	81031398	C	0	10	A	0.0005917
rs79645796	11	81031525	G	0	10	A	0.0005917
rs74590293	11	81031537	G	0	10	T	0.0005917
rs17369902	11	81031588	T	0	10	G	0.0005917
rs142751767	11	81031639	C	0	10	CA	0.0005917
rs17435637	11	81032637	A	0	10	G	0.0005917
rs78294947	11	81033308	C	0	10	T	0.0005917
rs115120279	11	81033409	C	0	10	T	0.0005917
rs17369997	11	81034927	C	0	10	A	0.0005917
rs56334566	11	81035122	T	0	10	TC	0.0005917
rs17141960	11	81035167	T	0	10	C	0.0005917
rs77455791	11	81036467	A	0	10	T	0.0005917
rs78348012	11	81036606	T	0	10	C	0.0005917
rs76083398	11	81037599	T	0	10	G	0.0005917

rs17370219	11	81037858	C	0	10	T	0.0005917
rs116957049	11	81038128	G	0	10	A	0.0005917
rs200508407	11	81038215	CA	0	10	C	0.0005917
rs77489824	11	81042247	A	0	10	C	0.0005917
rs79001473	11	81043432	G	0	10	A	0.0005917
rs77224744	11	81044607	T	0	10	C	0.0005917
rs114629989	11	81044656	A	0	10	G	0.0005917
rs182228872	11	81053076	G	0	10	T	0.0005917
rs185331674	11	81053077	T	0	10	C	0.0005917
rs80328220	11	81053637	T	0	10	A	0.0005917
rs147003829	11	81054364	AT	0	10	A	0.0005917
rs78005260	11	81054839	A	0	10	G	0.0005917
rs76081894	11	81057977	C	0	10	T	0.0005917
rs17371832	11	81059371	T	0	10	C	0.0005917
rs151150014	11	81060030	CATTT	0	10	C	0.0005917
rs56240555	11	81062072	A	0	10	G	0.0005917
rs7114073	11	81063336	A	0	10	T	0.0005917
rs55908086	11	81064868	T	0	10	C	0.0005917
rs139935298	11	81069039	T	0	10	G	0.0005917
rs116471067	11	81069721	G	0	10	A	0.0005917
rs146868223	11	81071462	TAC	0	10	T	0.0005917
rs140721601	11	81071566	T	0	10	TAC	0.0005917
rs17372055	11	81073164	T	0	10	C	0.0005917
rs17437784	11	81074310	T	0	10	C	0.0005917
rs56314671	11	81074741	C	0	10	T	0.0005917
rs72117550	11	81074777	C	0	10	CTTG	0.0005917
rs7358497	11	81075865	A	0	10	G	0.0005917
rs150387842	11	81079159	A	0	10	G	0.0005917
rs56085595	11	81081353	T	0	10	C	0.0005917
rs200435150	11	81085102	T	0	10	TTC	0.0005917
rs56025716	11	81086580	G	0	10	T	0.0005917
rs56032706	11	81086849	T	0	10	A	0.0005917
rs80138882	11	81090085	A	0	10	C	0.0005917
rs74639108	11	81091305	G	0	10	A	0.0005917
rs56043695	11	81092528	A	0	10	G	0.0005917
rs78927185	11	81093572	T	0	10	A	0.0005917
rs57921519	11	81095156	T	0	10	G	0.0005917
rs12292395	11	81096278	G	0	10	A	0.0005917
rs12288180	11	81097453	C	0	10	T	0.0005917
rs78410493	11	81101455	G	0	10	C	0.0005917

rs75692693	11	81104851	A	0	10	C	0.0005917
rs74374738	11	81107512	A	0	10	G	0.0005917
rs17439276	11	81108596	C	0	10	T	0.0005917
rs77564017	11	81118376	C	0	10	T	0.0005917
rs74941333	11	81119042	T	0	10	G	0.0005917
rs17439996	11	81122694	C	0	10	T	0.0005917
rs56135807	11	81126313	T	0	10	C	0.0005917
rs74502333	11	81127188	T	0	10	C	0.0005917
11:81127561	11	81127561	C	0	10	CCTCTGTGTGTGT	0.0005917
rs17440143	11	81128565	C	0	10	T	0.0005917
rs139433786	11	81128988	TGATC	0	10	T	0.0005917
rs147579820	11	81129670	CTG	0	10	C	0.0005917
rs17440276	11	81130250	C	0	10	G	0.0005917
rs148887234	11	81130953	TGA	0	10	T	0.0005917
rs17440290	11	81131518	C	0	10	A	0.0005917
rs17440339	11	81132432	A	0	10	C	0.0005917
rs5792972	11	81525938	C	6	22	CTT	0.0007482
11:83019686	11	83019686	G	35	12	A	0.0003064
11:83019706	11	83019706	T	30	10	A	0.0006347
rs10501586	11	84756077	C	34	12	T	0.0003362
rs11234273	11	84762531	T	36	14	C	0.0007756
rs10898349	11	84766179	A	33	12	T	0.0005855
rs10898350	11	84766288	T	33	12	A	0.0005855
rs11234277	11	84774564	A	34	12	G	0.0003362
rs34936538	11	84776609	A	34	12	AT	0.0003362
rs7127854	11	84777176	C	35	12	T	0.0003064
rs4402316	11	84780098	C	36	14	G	0.0007756
rs4424693	11	84780124	G	34	12	T	0.0003362
rs10898358	11	84782126	T	37	14	C	0.0004475
rs4944507	11	84783389	T	33	12	G	0.0005855
rs11234289	11	84783934	C	40	17	T	0.0006981
rs1826613	11	84786132	T	36	14	C	0.0007756
rs10898359	11	84786445	C	37	14	T	0.0004475
rs7118119	11	84787554	C	37	14	T	0.0004475
rs7102107	11	84787736	G	37	14	A	0.0004475
rs1377302	11	84799264	T	37	14	C	0.0004475
rs1377303	11	84799479	A	37	14	G	0.0004475
rs9634002	11	84799726	A	37	14	C	0.0004475
rs9634003	11	84799851	A	37	14	G	0.0004475
rs9634018	11	84799874	C	37	14	T	0.0004475

rs9634004	11	84800022	C	37	14	A	0.0004475
rs57719262	11	87207380	A	12	0	G	0.0003422
rs79744783	11	87211218	T	11	0	C	0.0007274
11:87337925	11	87337925	C	11	0	A	0.0007274
rs117152419	11	87362825	G	11	0	A	0.0007274
rs308788	11	88285408	C	3	18	T	0.0003291
rs308787	11	88285704	C	3	18	T	0.0003291
rs308786	11	88285887	G	3	18	A	0.0003291
rs308785	11	88286031	G	3	18	T	0.0003291
rs308763	11	88289360	A	3	17	G	0.0006426
rs16914244	11	88295675	G	2	15	T	0.0006243
rs308872	11	88295873	G	3	17	C	0.0006426
rs308874	11	88297066	T	3	17	C	0.0006426
rs7949902	11	88297748	C	2	15	T	0.0006243
rs60953957	11	88308583	A	2	15	ATTTAT	0.0006243
rs3913664	11	88313677	T	2	15	C	0.0006243
rs565969	11	95115165	T	26	7	C	0.0004518
rs522405	11	103300023	C	5	20	T	0.0009659
rs646311	11	103300854	A	5	20	C	0.0009659
rs617658	11	103302733	G	5	20	C	0.0009659
rs637166	11	103308611	A	5	20	G	0.0009659
rs546524	11	103309088	C	5	20	T	0.0009659
rs146675248	11	103361949	ACCACTGAAAGATGGTTT	2	15	A	0.0006243
rs34239719	11	103863119	G	14	1	A	0.0006347
11:104241136	11	104241136	A	5	24	ATG	0.00007319
11:104241140	11	104241140	A	5	24	ATATG	0.00007319
11:113378629	11	113378629	G	21	42	T	0.00006852
rs10892138	11	117437699	G	15	1	T	0.0003131
rs59765231	11	117438189	C	15	1	T	0.0003131
rs648740	11	117447373	A	14	1	G	0.0006347
rs12799825	11	117448013	A	15	1	C	0.0003131
rs681046	11	117452212	C	15	1	T	0.0003131
rs512393	11	119823440	C	7	25	G	0.0003419
rs595167	11	119853430	T	8	25	C	0.000871
rs5795209	11	119882854	G	20	40	GT	0.0008835
rs61897906	11	119887951	A	20	40	G	0.0008835
11:119889757	11	119889757	CCG	20	40	C	0.0008835
rs668400	11	119901095	A	33	55	C	0.0005652
rs10892517	11	119902255	T	33	54	C	0.0009764
rs11605919	11	119902691	C	33	55	T	0.0005652

rs11217646	11	119903119	G	33	56	A	0.0003196
rs1787023	11	119903704	T	22	45	C	0.0002011
rs11217650	11	119905180	T	39	16	G	0.0006105
rs1530919	11	119905908	A	18	40	G	0.0002349
rs12420530	11	119907605	T	18	40	G	0.0002349
rs11217652	11	119909306	C	18	40	G	0.0002349
rs5795211	11	119910438	T	18	40	TC	0.0002349
rs2084893	11	119914182	G	18	40	C	0.0002349
rs1597866	11	119914915	G	18	40	A	0.0002349
rs2100357	11	119915395	T	18	40	C	0.0002349
rs2084892	11	119915651	A	18	40	G	0.0002349
rs2887378	11	119916226	A	18	40	G	0.0002349
rs11217656	11	119918703	A	18	40	G	0.0002349
rs11217660	11	119920139	A	18	40	G	0.0002349
rs12421541	11	119922558	T	18	40	A	0.0002349
rs666640	11	119923259	T	18	40	A	0.0002349
rs1001973	11	119923643	A	18	40	G	0.0002349
rs664845	11	119923652	A	18	40	G	0.0002349
rs628861	11	119925575	A	18	40	C	0.0002349
rs4938762	11	119927043	T	18	40	C	0.0002349
rs11217663	11	119927491	C	18	40	T	0.0002349
rs1545509	11	119927888	A	18	40	G	0.0002349
rs10444336	11	119928883	A	19	40	C	0.0004646
rs10444337	11	119929276	A	19	41	G	0.0002649
rs10444338	11	119929445	C	19	40	T	0.0004646
rs56937356	11	119932050	T	18	39	C	0.0004141
rs78722037	11	122920668	T	23	6	A	0.0009874
rs4265607	11	124108041	T	51	28	C	0.0007148
rs11607806	11	124116657	T	48	25	G	0.0008213
rs4935875	11	124130019	C	42	19	G	0.0008809
rs35020430	11	124166986	T	39	16	TA	0.0003359
rs34845439	11	124212130	TTG	44	18	T	0.0002616
11:124230151	11	124230151	AAGGG	39	16	A	0.0006105
rs3901494	11	124235672	C	43	20	T	0.0009741
rs10893290	11	124704994	T	40	17	C	0.0006981
rs10893292	11	124705936	A	40	17	T	0.0006981
rs11220588	11	126586008	T	2	15	G	0.0006243
rs4581457	11	126586154	A	2	15	G	0.0006243
rs4499015	11	126586335	G	2	15	A	0.0006243
rs4099578	11	126597139	T	4	19	C	0.0005969

rs73024493	11	127709073	A	18	3	G	0.0008121
rs73024494	11	127709327	T	19	3	G	0.0004265
rs79219070	11	130642010	A	12	0	ATG	0.0003422
rs4128590	11	131224448	C	22	5	T	0.0006676
rs12303649	12	5998330	C	25	7	G	0.0008092
rs11609265	12	6023222	C	19	41	T	0.0002649
rs205543	12	11844691	A	30	54	G	0.0001708
rs2710304	12	11957231	T	41	18	C	0.0007885
rs111648492	12	12828685	C	1	13	T	0.0005059
rs74475305	12	12839118	C	1	13	T	0.0005059
rs11610142	12	12845381	C	1	13	G	0.0005059
rs17820421	12	12847990	G	1	13	A	0.0005059
rs17758597	12	12849241	G	1	13	A	0.0005059
rs138550114	12	12869553	TG	1	14	T	0.0002433
rs60120298	12	16155276	A	32	11	AC	0.0004661
rs55850346	12	16155283	A	32	11	G	0.0004661
rs1913823	12	20885641	T	29	50	C	0.0009027
rs11046890	12	23479654	G	2	15	A	0.0006952
rs17408900	12	24277459	C	6	22	T	0.0007921
rs1039228	12	24285197	C	6	22	G	0.0007921
rs11047323	12	24384154	G	16	36	A	0.0005556
rs113537256	12	29752901	A	1	14	ATG	0.0002716
12:29752905	12	29752905	A	1	14	G	0.0002716
rs1402360	12	43276559	T	5	26	G	0.00001845
rs12721373	12	48275007	A	5	20	T	0.0009659
12:57058121	12	57058121	ACG	3	17	A	0.0006426
12:57058148	12	57058148	C	3	17	T	0.0006426
rs117120119	12	57237463	G	12	0	C	0.0003422
rs199578813	12	58091715	A	1	13	C	0.0005059
rs1515907	12	59557898	A	33	12	G	0.0005855
rs10877236	12	59585251	A	33	12	G	0.0005855
rs6581238	12	59586950	G	33	12	T	0.0005855
rs35877988	12	59591495	C	33	12	G	0.0005855
rs10877241	12	59599105	G	33	12	T	0.0005855
rs10877843	12	62823200	T	50	25	A	0.000499
rs6581895	12	69842643	C	32	10	T	0.0002023
rs35773387	12	70780867	AT	6	22	A	0.0007921
rs10784841	12	70783297	A	6	23	G	0.000429
12:73570125	12	73570125	C	0	13	CACACACACACACACACACACA	0.00005596
rs12316906	12	77266718	C	2	15	G	0.0006243

rs10862707	12	77270548	A	2	15	C	0.0006243
rs12321227	12	77271020	T	2	15	C	0.0006243
rs146922274	12	77467304	T	1	13	TTTTA	0.0005059
rs61932809	12	77468993	T	1	13	C	0.0005059
rs61930667	12	77472384	A	1	13	T	0.0005059
rs61930670	12	77475922	G	1	13	A	0.0005059
rs61930671	12	77478571	T	1	13	C	0.0005059
rs11610700	12	77482684	A	1	14	C	0.0002433
rs310821	12	77491230	C	24	45	T	0.0007128
rs310820	12	77491399	T	24	45	A	0.0007128
rs310819	12	77492074	A	24	45	G	0.0007128
rs310818	12	77492770	G	24	45	T	0.0007128
rs310816	12	77493555	A	24	45	G	0.0007128
rs310815	12	77493588	A	24	45	G	0.0007128
rs10863092	12	77494328	T	24	45	C	0.0007128
rs76566838	12	77495670	A	1	13	AGCC	0.0005059
rs61930294	12	77496073	A	1	13	G	0.0005059
rs11614473	12	77496878	A	1	12	G	0.0008359
rs74633707	12	77499208	A	1	13	G	0.0005059
rs56149569	12	77500698	C	1	13	T	0.0005059
rs9971921	12	77501316	T	1	13	C	0.0005059
rs61930296	12	77502598	A	1	13	G	0.0005059
rs78152080	12	77503218	T	1	13	C	0.0005059
rs75366818	12	77503221	C	1	13	T	0.0005059
rs59223587	12	77503897	T	1	13	C	0.0005059
rs61930298	12	77504676	G	1	13	A	0.0005059
rs10506730	12	77508673	G	1	13	A	0.0005059
rs10506731	12	77509089	G	1	13	A	0.0005059
rs11610352	12	77510711	G	1	13	A	0.0005059
rs7487338	12	77520190	G	0	10	A	0.0004472
rs12370635	12	84468344	G	11	0	A	0.0007274
rs10858653	12	88253135	G	8	25	C	0.000871
rs930975	12	88257983	G	8	25	A	0.000871
rs1461288	12	88261010	G	8	25	A	0.000871
rs7296180	12	88262227	C	8	25	A	0.000871
rs990398	12	88264362	T	8	25	G	0.000871
rs1461289	12	88268444	C	8	25	T	0.000871
rs10777073	12	88268569	T	8	25	C	0.000871
rs10777074	12	88269137	A	8	25	G	0.000871
rs10745453	12	88269390	G	8	25	A	0.000871

rs10745454	12	88269432	T	8	25	G	0.000871
rs7398418	12	88269783	G	8	25	A	0.000871
rs7397529	12	88269866	T	8	25	G	0.000871
rs141370509	12	88270347	C	8	25	CA	0.000871
rs11104654	12	88270856	T	8	25	C	0.000871
rs10858655	12	88273017	G	8	25	A	0.000871
rs150428505	12	88273400	GAATA	8	25	G	0.000871
rs34611513	12	88273554	CT	8	25	C	0.000871
rs7307395	12	88277170	A	8	25	G	0.000871
rs7307899	12	88277528	A	8	25	G	0.000871
rs150532796	12	88277990	GAATT	8	25	G	0.000871
rs10858657	12	88278061	T	8	25	C	0.000871
rs6538117	12	88280181	C	8	25	G	0.000871
rs10777075	12	88282202	C	7	24	T	0.0005769
rs9705744	12	88282495	G	8	25	C	0.000871
rs7979676	12	88284484	G	8	25	C	0.000871
rs11410172	12	88285087	T	8	26	TA	0.0004835
rs2061594	12	88285434	G	8	25	A	0.000871
rs7309469	12	88286425	T	8	25	C	0.000871
rs6538120	12	88287952	T	8	25	C	0.000871
rs1964534	12	88290182	G	8	25	C	0.000871
rs1963507	12	88290353	C	8	25	T	0.000871
rs10777076	12	88291567	C	8	25	T	0.000871
rs11104657	12	88291872	T	8	25	C	0.000871
rs2199360	12	88292123	A	8	25	G	0.000871
rs5799842	12	88292262	TA	8	25	T	0.000871
rs1381144	12	88293518	G	8	25	A	0.000871
rs10745456	12	88294144	T	8	25	C	0.000871
rs60368479	12	88294154	T	8	25	TCCC	0.000871
rs4393402	12	88295289	T	8	25	G	0.000871
rs4842583	12	88296249	T	8	26	A	0.0004835
rs1461295	12	88296431	G	8	26	A	0.0004835
rs3990711	12	88297044	G	8	26	GAAAT	0.0004835
rs1471310	12	88297553	T	8	26	C	0.0004835
rs2169320	12	88298289	T	8	26	C	0.0004835
rs1947691	12	88298411	T	8	26	C	0.0004835
rs4842584	12	88298828	G	8	26	A	0.0004835
rs7303524	12	88299317	A	8	26	G	0.0004835
rs1903448	12	88300103	A	8	26	G	0.0004835
rs4482112	12	88300393	A	8	26	G	0.0004835

rs2100815	12	88302045	T	8	26	G	0.0004835
rs2085867	12	88302244	A	8	26	G	0.0004835
rs12304843	12	88303453	T	8	26	C	0.0004835
rs4842587	12	88305128	A	8	26	G	0.0004835
rs7302509	12	88305874	A	8	26	G	0.0004835
rs7303385	12	88306216	G	8	26	T	0.0004835
rs11104664	12	88306519	G	8	26	A	0.0004835
rs11610485	12	88306600	G	8	26	T	0.0004835
rs11610898	12	88306816	C	8	26	T	0.0004835
rs11104665	12	88307756	G	8	26	A	0.0004835
rs11104666	12	88307820	T	8	26	C	0.0004835
rs11104667	12	88308240	T	8	26	A	0.0004835
rs10858662	12	88308413	T	8	26	C	0.0004835
rs1824556	12	88309252	G	8	26	A	0.0004835
rs1824557	12	88309371	T	8	26	C	0.0004835
rs1824558	12	88309378	A	8	26	G	0.0004835
rs2045696	12	88309771	G	8	26	A	0.0004835
rs10858663	12	88310520	C	8	26	A	0.0004835
rs7966738	12	88310899	G	8	26	A	0.0004835
rs7966828	12	88310944	G	8	26	T	0.0004835
rs7966894	12	88311235	A	8	26	G	0.0004835
rs10858664	12	88311434	C	8	26	T	0.0004835
rs1461291	12	88312284	A	8	26	T	0.0004835
rs5799843	12	88313577	TA	8	27	T	0.0002641
rs4842589	12	88314167	C	5	20	T	0.0009659
rs10623160	12	88314247	C	5	20	CCT	0.0009659
rs1461292	12	88314884	A	5	20	G	0.0009659
rs1461293	12	88314943	A	5	20	G	0.0009659
rs7954853	12	88315135	G	5	20	A	0.0009659
rs4558240	12	88315366	C	5	20	T	0.0009659
rs4257059	12	88317005	A	5	20	C	0.0009659
rs7304833	12	88318783	T	5	20	C	0.0009659
rs4842590	12	88319381	A	5	20	G	0.0009659
rs4842591	12	88319474	A	5	20	G	0.0009659
rs4842592	12	88319723	G	5	20	A	0.0009659
rs10858666	12	88321186	T	5	20	G	0.0009659
rs71082414	12	88321425	C	5	20	CACATGG	0.0009659
rs11104674	12	88321574	G	5	20	A	0.0009659
rs4385971	12	88322247	C	5	20	T	0.0009659
rs11402841	12	88323117	A	5	20	AT	0.0009659

rs11104676	12	88323588	C	5	20	A	0.0009659
rs10858667	12	88324226	A	5	20	G	0.0009659
rs79150298	12	90433721	A	0	10	G	0.0005917
12:90989945	12	90989945	T	18	37	TATACACACATATATACACGTA[illegible]	0.0005937
rs10777701	12	95935574	T	39	16	C	0.0006105
rs60149422	12	95936869	A	35	13	AAAAAAAG	0.0005883
rs11445609	12	96242184	C	55	29	CT	0.000347
12:105526569	12	105526569	A	50	24	T	0.0002688
rs657710	12	118169666	A	45	20	C	0.000305
rs375510578	12	118604654	T	20	4	C	0.0007606
rs3916065	12	120934624	C	16	37	A	0.0009189
rs11058858	12	127393386	T	1	13	C	0.0005059
rs57870255	12	129001148	G	20	40	A	0.0008835
rs78957821	12	130092534	T	14	1	C	0.0006347
rs73148954	12	130694644	C	6	22	T	0.0007921
rs61943885	12	132680369	A	0	10	G	0.0005917
rs28693604	12	132866526	A	6	22	G	0.0007921
rs9552087	13	20710492	T	27	8	A	0.0006326
rs9508336	13	29863014	G	14	37	C	0.00006864
rs7319242	13	29863365	T	14	36	C	0.0001265
rs4769709	13	29865808	A	20	40	G	0.0008835
rs11365604	13	29865972	C	14	36	CA	0.0001265
rs1926468	13	36453053	T	34	55	A	0.0009818
rs4941822	13	36453613	A	34	55	C	0.0009818
rs4943350	13	36453860	T	34	55	C	0.0009818
rs2051090	13	36454193	A	34	55	G	0.0009818
rs1936002	13	36456084	T	34	55	C	0.0009818
rs9545602	13	36456503	C	34	55	T	0.0009818
rs6563206	13	36457734	T	35	56	C	0.0006468
13:39286788	13	39286788	C	44	20	CA	0.0005739
13:39286789	13	39286789	C	44	20	A	0.0005739
13:39286790	13	39286790	C	44	20	CACA	0.0009594
rs7327319	13	40928617	A	11	30	C	0.0006171
rs12867977	13	40930824	C	8	27	G	0.0002641
rs9526773	13	52080679	A	26	50	G	0.0001475
rs9568615	13	52080884	A	22	43	C	0.0006167
rs11617426	13	52081231	G	24	46	C	0.0004131
rs9646005	13	52081846	T	24	46	C	0.0004131
13:52085316	13	52085316	AGGATTGACTTG	16	35	A	0.0004279
rs142218030	13	52086144	G	25	46	A	0.0007575

rs148731067	13	52086145	C	23	46	T	0.0002182
rs9535704	13	52087589	G	25	46	A	0.0007575
rs144821576	13	52088239	AC	25	46	A	0.0007575
rs11619681	13	52088867	C	25	46	G	0.0007575
rs12866822	13	52089286	G	25	46	A	0.0007575
rs11617690	13	52089453	G	25	46	A	0.0007575
rs11617721	13	52089629	G	25	46	A	0.0007575
rs11619951	13	52089631	A	25	46	G	0.0007575
rs12861627	13	52090275	A	23	46	G	0.0002182
rs4943004	13	52090864	T	25	46	A	0.0007575
rs11616977	13	52092247	C	25	46	A	0.0007575
rs1537465	13	52093119	C	25	46	T	0.0007575
rs1537464	13	52093574	T	25	46	G	0.0007575
rs9535706	13	52094811	C	25	46	T	0.0007575
rs12875314	13	52096145	G	23	46	A	0.0002182
rs9535708	13	52097246	A	25	46	G	0.0007575
rs9535709	13	52097842	A	25	46	T	0.0007575
rs9526776	13	52097905	T	23	46	A	0.0002182
rs9526777	13	52097959	T	25	46	G	0.0007575
rs12867577	13	52098171	T	25	46	A	0.0007575
rs9563838	13	61307706	A	18	3	G	0.0008121
rs35122353	13	63405760	A	17	36	T	0.0009486
rs1913223	13	65370523	A	14	1	T	0.0006347
rs9592365	13	65381616	A	14	1	G	0.0006347
rs9592366	13	65382906	A	14	1	G	0.0006347
rs117707305	13	65383451	T	14	1	C	0.0006347
rs9592367	13	65386113	A	16	1	G	0.0001529
rs73200478	13	65387678	T	16	1	C	0.0001529
rs73200481	13	65390377	A	16	1	G	0.0001529
13:65397340	13	65397340	A	22	44	C	0.0003557
rs150865846	13	65400548	A	16	1	ATTT	0.0001529
rs139827942	13	65402941	A	16	2	AACTTCCAGAGAAAGGAACAG	0.0007845
rs9598840	13	65406636	G	16	1	C	0.0001529
rs12429888	13	65413569	T	16	1	C	0.0001529
rs12427871	13	65413611	G	16	2	A	0.0007845
rs67763731	13	65416328	A	16	2	T	0.0007845
rs9598841	13	65419872	T	16	1	A	0.0001529
rs11148671	13	65757407	T	35	13	C	0.0006539
rs9540374	13	65760500	C	38	15	A	0.0005267
13:65760773	13	65760773	A	32	10	T	0.0002023

13:65760777	13	65760777	A	27	7	T	0.0002412
rs1865141	13	65773972	C	38	14	G	0.0002538
rs1338176	13	65775294	G	38	14	T	0.0002538
rs17068864	13	65779354	C	35	13	A	0.0006539
rs9540377	13	65783341	T	36	13	A	0.0003739
rs9317488	13	65793993	G	35	13	C	0.0006539
rs13378409	13	65988768	C	35	13	A	0.0006539
rs9540413	13	65992984	G	35	13	A	0.0006539
rs9540414	13	65993460	C	35	13	A	0.0006539
rs9540416	13	65994439	G	35	13	A	0.0006539
rs9528918	13	65996559	T	35	13	C	0.0006539
rs9646054	13	65998265	G	35	13	A	0.0006539
rs9528920	13	65999136	A	35	13	G	0.0006539
rs7989370	13	66001504	T	35	13	C	0.0006539
rs9540422	13	66004398	T	35	13	C	0.0006539
rs9598975	13	66009510	C	35	13	A	0.0006539
rs139787440	13	66012618	C	35	13	G	0.0006539
rs141462154	13	66013483	G	35	13	T	0.0006539
rs9598979	13	66013937	G	35	13	C	0.0006539
rs1811951	13	66015942	C	35	13	T	0.0006539
rs1855224	13	66016192	A	35	13	G	0.0006539
rs1855225	13	66016193	T	35	13	G	0.0006539
rs7337746	13	66016604	C	35	12	T	0.0003064
rs4400934	13	66018238	C	35	13	T	0.0006539
rs2151344	13	66019143	A	35	13	G	0.0006539
rs2151345	13	66019195	G	35	13	T	0.0006539
rs9598982	13	66020667	T	35	13	C	0.0006539
rs9598983	13	66024713	G	35	13	A	0.0006539
rs149812567	13	66026713	T	35	13	TA	0.0006539
rs12323031	13	66026770	A	35	13	C	0.0006539
rs9540460	13	66089459	T	16	2	C	0.0007845
rs61956686	13	66523846	T	1	13	C	0.0005059
rs17790880	13	66524097	T	1	13	G	0.0005059
rs2874736	13	66529672	T	1	13	C	0.0005059
rs61956687	13	66536491	T	1	13	C	0.0005059
rs61956690	13	66553951	T	1	13	C	0.0005059
rs17790904	13	66587024	A	1	13	G	0.0005059
rs147776445	13	68282542	CAT	12	0	C	0.0003422
rs7492037	13	69418044	A	40	15	G	0.0001692
rs75020554	13	75802814	A	14	1	C	0.0006347

13:77819880	13	77819880	A	27	48	ACAC	0.0008377
13:79012528	13	79012528	G	0	18	A	0.0000007387
rs9560792	13	87428272	C	41	18	T	0.0007885
rs9556104	13	87443582	T	41	18	C	0.0007885
rs11330429	13	87540259	C	55	29	CT	0.000347
rs969501	13	87617112	G	55	30	A	0.0006201
13:87640637	13	87640637	ATG	54	28	A	0.0003346
rs9561492	13	87689497	A	46	21	T	0.0003668
rs6492695	13	87695546	G	31	52	A	0.0009496
rs72636462	13	87695784	T	37	15	C	0.0009053
rs9524345	13	87700013	C	31	52	T	0.0009496
rs9524365	13	87702747	A	31	52	T	0.0009496
rs9524367	13	87703310	T	31	52	G	0.0009496
rs9516366	13	87705456	C	31	52	T	0.0009496
rs9556359	13	87706470	T	31	52	C	0.0009496
rs9524373	13	87706576	A	31	52	G	0.0009496
rs9516372	13	87709360	C	31	52	T	0.0009496
rs9516374	13	87709690	C	31	52	T	0.0009496
rs9524401	13	87712675	A	31	52	G	0.0009496
rs9561541	13	87719368	A	31	52	T	0.0009496
rs55848046	13	87720203	TATC	46	20	T	0.0001008
rs9561544	13	87720442	G	31	52	A	0.0009496
rs72636467	13	87721053	G	38	15	A	0.0005267
rs7987894	13	87722426	G	31	52	T	0.0009496
rs5805422	13	87723820	TAGAG	31	52	T	0.0009496
rs34773214	13	87724838	TCAAA	31	52	T	0.0009496
rs9645930	13	87726002	C	31	52	T	0.0009496
rs7998755	13	87736771	A	51	27	G	0.0009437
rs7990672	13	90838056	G	36	14	T	0.0007756
rs2130837	13	90838563	C	37	14	T	0.0004475
rs2350305	13	90842611	G	37	14	A	0.0004475
rs9301657	13	90850355	C	37	14	G	0.0004475
rs9301658	13	90850542	T	37	14	C	0.0004475
rs1931138	13	90852373	G	37	14	A	0.0004475
rs4771811	13	90853343	T	37	14	A	0.0004475
rs9555986	13	90857744	G	37	14	A	0.0004475
rs9560574	13	90858269	G	37	14	A	0.0004475
rs4773558	13	90859091	A	37	14	G	0.0004475
rs9524205	13	94423978	T	23	47	A	0.0002094
rs149410208	13	95074442	TA	0	11	T	0.0002715

rs9561826	13	95926133	A	18	3	G	0.0007671
rs945897	13	98472872	A	31	52	T	0.0009496
rs114819883	13	102257824	G	45	17	C	0.00006885
rs9558112	13	104218933	T	43	17	G	0.000129
rs9554971	13	104220996	T	43	17	A	0.000129
rs10665670	13	104274004	G	21	4	GCTTT	0.0004069
rs34000346	13	104274311	G	20	4	GA	0.000711
rs9558130	13	104274429	T	20	4	C	0.0007606
rs1984042	13	104287536	C	23	5	T	0.0003619
rs9300844	13	104290048	T	23	5	C	0.0003619
rs7983029	13	104291040	G	23	5	A	0.0003619
rs1331523	13	104294778	A	23	5	G	0.0003619
rs7327778	13	104295500	G	23	5	A	0.0003619
rs7320039	13	104298034	T	23	5	G	0.0003619
rs76704943	13	104299577	C	23	5	T	0.0003619
rs1331515	13	104299600	T	23	5	A	0.0003619
rs5806374	13	104304551	TA	23	5	T	0.0003619
rs3916934	13	104345535	C	59	29	T	0.00003083
rs1274746	13	104357493	A	57	30	T	0.0002015
rs1772557	13	104365226	T	58	30	C	0.0001111
rs61347079	13	104372780	G	41	17	GT	0.0004047
rs12873078	13	105078867	G	10	28	A	0.000853
13:107735084	13	107735084	GT	50	24	G	0.0002688
13:107735085	13	107735085	T	48	24	A	0.000451
13:107735086	13	107735086	AGAGTAGAGTAGAGT	50	24	A	0.0002688
rs7997308	13	108808267	G	25	7	A	0.0008092
rs7335064	13	109731736	G	53	26	T	0.0000898
rs995292	13	110664771	C	7	24	T	0.0006277
rs2026160	13	113792893	C	16	37	A	0.0003171
rs9577875	13	114517865	T	5	22	C	0.0006023
14:19072247	14	19072247	A	8	34	AT	0.000002477
rs2733763	14	22267491	T	58	33	C	0.0009818
rs7156758	14	22279416	A	49	23	C	0.0006904
rs7156201	14	22279817	A	49	25	G	0.00085
rs12897417	14	22282904	T	49	25	C	0.00085
rs7159790	14	22325185	T	49	23	A	0.0004159
rs3742491	14	24429249	A	23	3	G	0.0000286
rs139045649	14	24432493	C	24	4	G	0.00006571
rs138982419	14	24435308	A	25	5	C	0.000102
rs141694248	14	24436047	T	21	4	C	0.0004069

rs184829013	14	24476128	G	26	4	A	0.00003311
rs151035018	14	24477842	T	26	5	C	0.00005306
rs11158499	14	24479365	A	23	5	G	0.0003619
rs11158500	14	24479370	C	23	5	T	0.0003619
rs147865522	14	24480329	C	23	5	CTCTG	0.0003619
rs17184682	14	24793762	G	4	19	T	0.0005969
rs67156008	14	24795231	CCA	5	20	C	0.0009659
rs11622863	14	24795258	C	5	20	T	0.0009659
rs17256902	14	24795495	A	5	20	G	0.0009659
rs12892993	14	25185329	C	21	43	T	0.0003256
rs6573990	14	25212506	T	18	41	G	0.0001308
rs1461650	14	25213422	T	18	41	G	0.0001308
rs7155532	14	25236724	C	34	55	T	0.0009818
rs10483313	14	26639686	T	15	34	C	0.0008313
rs17568415	14	26641090	T	15	34	C	0.0008313
rs17490061	14	26643485	T	15	34	A	0.0008313
rs11623197	14	26650521	G	15	34	T	0.0008313
rs12437100	14	26652491	T	15	34	C	0.0008313
rs7155767	14	29475867	G	7	24	A	0.0006277
rs147624411	14	29487583	T	9	27	TAC	0.000654
14:32841982	14	32841982	T	6	25	G	0.0001117
rs61980339	14	33448386	C	14	1	A	0.0006347
rs7159000	14	33921142	T	24	6	C	0.0005592
rs10130494	14	33922187	G	25	6	A	0.0003059
rs8006708	14	33922883	G	25	6	A	0.0003059
rs8012273	14	33923923	C	25	6	T	0.0003059
rs8016805	14	33924157	G	25	6	A	0.0003059
rs8003435	14	33925348	T	24	6	C	0.0005592
rs8022941	14	33925424	G	24	6	C	0.0005592
14:34374954	14	34374954	A	8	26	C	0.0004835
rs61981198	14	35220605	G	16	35	A	0.000956
rs8005659	14	35223956	G	20	40	A	0.0008835
rs61981200	14	35227456	T	16	35	C	0.000956
rs61981202	14	35228090	A	16	35	G	0.000956
rs2383676	14	35229647	A	15	36	C	0.0004515
rs59678582	14	35235160	A	20	40	G	0.0008835
rs12435859	14	35242391	T	20	40	C	0.0008835
rs7153289	14	35246534	T	20	40	C	0.0008835
rs61981232	14	35254085	T	16	35	C	0.000956
rs113025158	14	35256623	T	16	34	G	0.0008093

rs35217207	14	35261589	CA	14	35	C	0.0002137
rs5807801	14	35343464	A	43	17	AC	0.0006933
rs1400745	14	35355300	A	53	27	G	0.0003204
rs77262868	14	35524570	C	43	19	T	0.0004795
rs17103005	14	35539882	T	42	19	A	0.0008809
rs9671773	14	35542146	A	42	19	G	0.0008809
14:38510836	14	38510836	T	12	0	C	0.0003547
rs4899384	14	71625956	T	31	54	A	0.0003134
rs7151384	14	71639873	G	30	10	T	0.0006347
rs7150382	14	71639875	T	30	10	G	0.0006347
rs8019082	14	71649714	A	28	9	G	0.0008484
rs112830953	14	77299979	C	2	15	T	0.0006243
rs76970624	14	77305444	A	2	15	G	0.0006243
rs79735523	14	77307359	C	2	15	A	0.0006243
14:77319775	14	77319775	A	0	10	T	0.0005917
14:78492143	14	78492143	TGTGTGTGC	15	35	T	0.0004795
14:89482375	14	89482375	A	2	16	T	0.0003115
rs1967774	14	90142951	A	30	55	G	0.0002709
rs732914	14	90148522	A	56	31	G	0.0006352
rs7158240	14	90150146	G	45	21	A	0.0006315
rs61998683	14	90150796	C	42	19	T	0.0008809
rs8006462	14	90878676	T	41	17	C	0.0004047
rs2098254	14	90884018	C	41	18	A	0.0007885
rs2896136	14	90898875	G	41	18	T	0.000443
rs6575136	14	90899380	C	43	19	G	0.0005165
rs2069963	14	95052627	T	12	32	C	0.0004387
rs59700946	14	95093097	C	54	27	G	0.000181
14:95832714	14	95832714	AT	18	0	A	0.000003529
14:95832717	14	95832717	A	15	1	T	0.0003611
14:95832729	14	95832729	C	16	0	CAT	0.00001528
rs1892224	14	98763349	T	58	29	C	0.00005841
rs10138335	14	98772136	C	58	29	T	0.00005841
rs1807069	14	98773458	G	56	30	A	0.0001878
rs35404361	14	98777356	T	58	31	C	0.0002058
rs4365229	14	98806562	T	57	30	C	0.0002015
rs12892719	14	101489858	T	37	14	C	0.0004475
rs12586258	14	101492548	T	37	13	C	0.0002102
rs139798344	14	102994876	TAATA	1	13	T	0.0005059
rs190885263	14	103632418	T	2	15	C	0.0006243
rs141671546	14	104631874	GTC	20	40	G	0.0008835

rs10147900	14	105092031	T	20	4	C	0.0006746
14:106784161	14	106784161	T	22	4	C	0.0002148
rs10136477	14	107165757	G	23	6	A	0.0009286
rs10148746	14	107165764	A	23	6	T	0.0009286
rs149749573	15	22323899	T	31	10	TTTTC	0.0003124
rs35393720	15	25529775	T	12	0	TA	0.0003422
rs74004627	15	26885776	G	2	16	A	0.0003115
rs7173713	15	26886154	T	2	16	C	0.0003115
rs4438262	15	27674176	C	36	14	T	0.0007756
rs3922613	15	27682831	C	36	14	T	0.0007756
rs7167756	15	27709626	C	31	11	T	0.0008085
rs1258799	15	33106027	A	26	47	C	0.0007993
rs1258795	15	33107305	A	25	47	G	0.0004398
rs77537015	15	39355732	C	24	5	T	0.0001934
rs12148236	15	39374881	G	23	5	A	0.0003619
rs72739328	15	39377572	A	23	5	G	0.0003619
15:41438169	15	41438169	G	11	0	A	0.0007228
15:51326289	15	51326289	C	28	9	T	0.0008484
15:51326290	15	51326290	A	28	9	T	0.0008484
rs2470149	15	51612240	T	14	1	A	0.0006347
rs2445763	15	51618307	G	14	1	A	0.0006347
rs2470146	15	51618369	G	14	1	A	0.0006347
rs2446404	15	51618943	C	14	1	T	0.0006347
rs2470145	15	51619138	C	14	1	T	0.0006347
rs17602385	15	51624730	C	14	1	T	0.0006347
rs2470169	15	51625116	G	14	1	T	0.0006347
rs2470165	15	51627798	T	14	1	A	0.0006347
rs2446409	15	51628101	A	14	1	G	0.0006347
rs2445764	15	51633517	G	14	1	A	0.0006347
rs2445769	15	51635715	C	14	1	T	0.0006347
rs2445770	15	51637158	A	14	1	G	0.0006347
rs2446425	15	51639727	T	14	1	C	0.0006347
rs2446426	15	51641156	T	14	1	C	0.0006347
rs2470162	15	51643567	G	14	1	C	0.0006347
rs2470161	15	51644669	T	14	1	C	0.0006347
rs17525891	15	52014185	C	2	19	T	0.00003587
rs113030016	15	52060798	A	2	17	G	0.0001345
rs78565107	15	52068267	C	2	18	G	0.00007463
rs17526337	15	52086990	T	2	19	A	0.00003587
rs112889926	15	52197576	T	3	18	C	0.0003291

rs112136884	15	52225041	T	3	19	C	0.0001662
rs9744466	15	52261571	A	3	19	C	0.0001662
rs28367289	15	57868701	A	3	18	G	0.0003291
rs56227417	15	58500956	T	48	20	C	0.00005747
rs4350519	15	58501146	C	47	21	G	0.000209
rs1618701	15	58501282	T	47	20	A	0.0001051
rs1663251	15	58501481	G	50	24	T	0.0002688
rs7180071	15	58502472	A	47	20	G	0.0001051
rs2414551	15	58503128	T	47	20	C	0.0001051
rs1663253	15	58504678	C	47	22	T	0.0004013
rs2017566	15	58505040	T	47	21	C	0.000209
rs1711062	15	58508790	G	46	22	C	0.0006885
rs1663254	15	58513993	G	47	22	A	0.0004013
rs1663255	15	58514242	C	47	21	T	0.000209
rs11631950	15	58520259	C	46	22	T	0.0006885
rs35120591	15	58814992	C	25	6	T	0.0003059
rs36013682	15	65515981	G	49	25	A	0.00085
rs1561890	15	65539407	T	52	28	C	0.0009847
rs8029480	15	65540299	T	52	28	C	0.0009847
rs34711258	15	65543186	T	52	28	C	0.0009847
rs11270085	15	65543940	G	52	28	GGATCCCCAGCAAAACTTATGC	0.0009847
rs4776262	15	65544293	A	52	28	G	0.0009847
rs12906430	15	65548870	T	52	28	C	0.0009847
rs2414879	15	65549457	T	52	28	C	0.0009847
rs1961279	15	65554740	A	52	27	G	0.0005555
rs894494	15	65556442	C	52	27	T	0.0005555
rs10467935	15	65561077	T	52	26	C	0.0003045
rs8023288	15	65561829	A	52	27	C	0.0005555
rs7167901	15	65562811	G	52	27	A	0.0005555
rs1982257	15	65563649	C	52	27	T	0.0005555
rs8032837	15	65567436	A	52	26	C	0.0004998
rs10083573	15	65569083	C	52	27	T	0.0005555
rs62012559	15	65569302	C	52	25	A	0.0002708
rs62014960	15	65569312	A	51	25	C	0.0004733
rs4776679	15	65569933	G	52	27	C	0.0005555
rs4776680	15	65571977	G	52	27	A	0.0005555
rs2899707	15	65575585	C	52	27	G	0.0005555
rs34440719	15	65577548	T	51	27	TCAC	0.0005318
rs1057254	15	65579033	A	51	25	G	0.0004733
rs7171823	15	65580983	C	52	27	T	0.0005555

rs12595530	15	65591379	G	25	47	A	0.0004398
rs12905996	15	65592944	G	25	47	A	0.0004398
rs34552880	15	65593344	C	25	47	CA	0.0004398
rs12898603	15	65595522	G	25	47	A	0.0004398
rs12898396	15	65595680	A	25	47	G	0.0004398
rs526794	15	65597138	G	25	47	A	0.0004398
rs525725	15	65597284	A	25	47	G	0.0004398
rs9788655	15	65600948	G	25	47	T	0.0004398
rs491426	15	65618326	A	25	47	G	0.0004398
rs12905133	15	65619042	C	25	47	T	0.0004398
rs11071839	15	65619427	T	25	47	A	0.0004398
rs7174072	15	65620156	C	25	48	T	0.0002502
rs11853777	15	65621138	G	25	47	A	0.0004398
rs12907128	15	65621441	C	25	47	A	0.0004398
rs2013609	15	67253339	A	56	30	C	0.0003574
rs444141	15	67253847	G	56	30	C	0.0003574
15:91179365	15	91179365	C	16	2	A	0.0007845
15:91179398	15	91179398	T	19	2	G	0.0001015
rs16974971	15	95806551	T	5	20	C	0.0009659
rs12592800	15	98714007	G	27	48	A	0.0008377
rs4998382	15	99331289	C	27	7	G	0.0002206
rs7165207	15	99619610	A	28	9	G	0.0008484
rs8038925	15	99621043	A	29	9	T	0.0004831
rs1509157	15	99623607	T	29	9	C	0.0004831
rs10459737	15	99626170	T	29	9	C	0.0004831
rs1551244	15	99629260	A	29	8	G	0.0001956
rs12900689	15	1.00E+08	G	7	26	A	0.0001002
rs56767751	15	1.00E+08	C	7	24	CAAAAT	0.0006277
rs2958996	15	1.00E+08	A	7	24	G	0.0006277
rs5815006	15	101821061	CA	18	40	C	0.0006891
rs12902917	15	101823337	T	20	41	G	0.0005156
rs12909636	15	101824271	T	20	40	C	0.0008835
rs12908996	15	101824624	A	20	41	G	0.0005156
rs28415096	15	101825064	T	20	40	G	0.0008835
rs10152335	15	101828423	T	20	41	C	0.0005156
rs10152348	15	101828572	T	19	40	C	0.0004646
rs34314457	15	101828597	A	19	39	G	0.0007998
rs8037809	15	101830244	C	20	40	T	0.0008835
rs12899728	15	101830812	C	21	41	T	0.0009669
rs2402095	16	1490322	G	7	25	A	0.0003419

16:6703978	16	6703978	G	0	12	A	0.0001036
rs2270287	16	8739110	A	20	4	G	0.0007606
rs72766499	16	8740900	T	23	5	C	0.0003619
rs2908709	16	9740239	G	21	42	A	0.0005665
rs72779817	16	9740421	T	8	27	C	0.0002641
rs17550406	16	9741343	C	8	28	T	0.000142
rs16965984	16	9741412	C	8	28	G	0.000142
rs11642363	16	9742790	G	8	27	T	0.0002641
rs34497568	16	9742912	A	8	27	AT	0.0002641
rs11647032	16	9743117	A	8	27	G	0.0002641
rs72779819	16	9743465	G	8	27	A	0.0002641
rs201756757	16	13267014	G	11	0	A	0.0007274
rs7498905	16	20694067	A	5	20	G	0.0009659
rs876106	16	20694194	C	5	20	A	0.0009659
rs12050994	16	20697868	T	5	20	G	0.0009659
rs11643793	16	20700016	A	5	20	G	0.0009659
rs111601937	16	20705241	G	5	20	A	0.0009659
rs76372699	16	20706955	G	5	20	A	0.0009659
rs11643502	16	20712538	A	5	20	G	0.0009659
rs11644530	16	20712837	G	5	20	A	0.0009659
rs35974780	16	20714033	C	5	21	T	0.0005184
rs11648920	16	20715718	T	5	20	C	0.0009659
rs3848252	16	20718939	A	5	20	G	0.0009659
rs9708736	16	20719627	G	5	20	A	0.0009659
rs35390946	16	20725086	A	5	20	G	0.0009659
rs66496446	16	20726035	T	5	20	C	0.0009659
rs3848254	16	20728166	T	5	20	C	0.0009659
rs11643620	16	20729699	A	5	20	G	0.0009659
rs11645111	16	20731013	T	5	20	C	0.0009659
rs10492808	16	20732321	T	5	20	C	0.0009659
rs10852246	16	20733134	A	5	20	G	0.0009659
rs11649682	16	20734520	C	5	20	A	0.0009659
rs71374831	16	20735386	A	5	20	G	0.0009659
rs35518073	16	20740257	T	5	20	C	0.0009659
rs16970587	16	20740517	T	5	20	C	0.0009659
rs34134130	16	20742185	G	5	20	T	0.0009659
rs764139	16	20743399	G	5	20	A	0.0009659
rs11641333	16	20744154	C	5	20	T	0.0009659
rs71149175	16	20745431	C	5	20	CAAATAA	0.0009659
rs67088894	16	20746239	C	5	20	G	0.0009659

rs1136268	16	20747104	T	5	20	C	0.0009659
rs11074471	16	20748331	A	5	20	C	0.0009659
rs34900980	16	20755650	A	5	20	G	0.0009659
rs67573009	16	20755662	G	5	20	A	0.0009659
rs201528322	16	20756915	G	5	20	A	0.0009659
rs35969935	16	20757590	C	5	20	T	0.0009659
rs35137814	16	20758433	A	5	20	C	0.0009659
rs11642478	16	20759548	T	5	20	C	0.0009659
rs12934252	16	20760423	C	5	20	T	0.0009659
rs11646463	16	20761804	T	5	20	C	0.0009659
rs66885877	16	20765744	C	5	20	T	0.0009659
rs34670153	16	20767741	A	5	20	G	0.0009659
rs373842582	16	20768432	C	5	20	T	0.0009659
rs375051391	16	20768433	A	5	20	G	0.0009659
rs368774211	16	20768763	G	4	19	A	0.0005969
rs201881632	16	20768968	T	5	20	G	0.0009659
rs199852853	16	20769158	C	5	20	T	0.0009659
rs35139281	16	20770247	T	5	20	TG	0.0009659
rs2356267	16	20771530	G	5	20	C	0.0009659
rs11643290	16	20772409	T	5	20	C	0.0009659
rs34160924	16	20773108	G	5	20	T	0.0009659
rs60388194	16	20773244	C	5	20	T	0.0009659
rs66647715	16	20773562	T	5	20	C	0.0009659
rs11645969	16	20773764	A	5	20	G	0.0009659
rs11647477	16	20774919	A	5	20	G	0.0009659
rs12232379	16	20777031	T	5	20	C	0.0009659
rs11644091	16	20777932	T	5	20	C	0.0009659
rs67428664	16	20779962	T	5	20	A	0.0009659
rs67410356	16	20780262	T	5	20	C	0.0009659
rs71374832	16	20780386	T	5	20	C	0.0009659
rs1027457	16	20781194	A	5	20	C	0.0009659
rs12919474	16	20781947	G	5	20	T	0.0009659
rs35620560	16	20782373	A	5	20	G	0.0009659
rs11641265	16	20783278	G	5	20	A	0.0009659
rs57941736	16	20783737	T	5	20	TA	0.0009659
rs11642585	16	20784016	C	5	20	A	0.0009659
rs7498567	16	20785716	C	5	20	T	0.0009659
rs2071524	16	20787770	A	5	20	G	0.0009659
rs2071525	16	20787945	G	5	20	A	0.0009659
rs2074541	16	20789335	A	5	20	G	0.0009659

rs11646755	16	20790095	T	5	20	C	0.000897
rs11642081	16	20790315	C	5	20	T	0.0009659
rs71374833	16	20790688	G	4	19	A	0.0006572
rs2074542	16	20792914	C	5	20	A	0.0009659
rs13306604	16	20793129	T	5	20	A	0.0009659
rs35396346	16	20794499	T	5	20	A	0.0009659
rs12932513	16	20794983	G	5	20	A	0.0009659
rs11279671	16	20804819	T	5	20	TTAAGG	0.0009659
rs34698709	16	20850993	C	5	20	T	0.0009659
rs34400115	16	20857698	A	5	20	C	0.0009659
rs16970701	16	20862595	T	5	20	C	0.0009659
16:20886534	16	20886534	G	36	12	A	0.0001705
rs78543395	16	21022113	G	3	18	A	0.0003291
rs16970821	16	21043801	C	3	18	T	0.0003291
rs78030383	16	21044853	T	3	18	C	0.0003291
rs2112494	16	21045369	G	3	18	A	0.0003291
rs78786332	16	21047284	A	3	18	G	0.0003291
rs7187726	16	21049508	G	3	18	T	0.0003291
rs139853718	16	21063298	CTATTT	3	18	C	0.0003291
rs74943127	16	21069038	C	3	18	G	0.0003291
rs79740697	16	21073002	T	3	18	C	0.0003291
rs76178045	16	21074366	T	3	18	C	0.0003291
rs76583972	16	21077904	T	3	18	G	0.0003291
rs77578182	16	21078220	T	3	18	A	0.0003291
rs77871434	16	21080309	T	3	18	G	0.0003291
rs75686265	16	21082163	T	3	18	C	0.0003291
rs79945700	16	21084738	T	3	18	C	0.0003291
rs74813399	16	21084802	A	3	18	G	0.0003291
rs78625859	16	21086487	C	3	18	T	0.0003291
rs59311940	16	21087131	G	3	17	T	0.0006426
16:26745609	16	26745609	GTTTT	0	10	G	0.0006636
rs11150592	16	30555658	G	25	7	A	0.0008092
rs77781754	16	31790375	T	0	10	A	0.0005917
16:32600808	16	32600808	A	5	23	T	0.0001328
rs8058337	16	49677354	T	25	7	G	0.0008092
rs2540732	16	49683231	C	31	11	A	0.0008085
rs200478485	16	55832344	G	42	18	A	0.0004599
rs201754281	16	55832348	G	45	19	A	0.0001682
rs377102550	16	55832352	G	42	18	A	0.0002532
rs202037976	16	56161190	G	12	46	A	0.00000001377

rs4783644	16	66383359	C	17	2	T	0.0004014
rs202191991	16	69886186	CT	2	16	C	0.0007376
rs200106830	16	72901631	A	4	19	AAAAAG	0.0005458
16:73093273	16	73093273	G	17	2	A	0.0003798
rs10871310	16	73498929	C	43	20	T	0.0009741
rs12597797	16	73511154	A	28	9	G	0.0008484
rs4888373	16	73519608	A	31	10	G	0.0003611
rs57762438	16	73520346	T	31	10	TG	0.0003611
rs8062545	16	73520460	T	31	10	C	0.0003611
rs8057817	16	73522400	T	32	10	G	0.0002023
rs4888428	16	73535986	C	31	10	A	0.0003611
rs4888429	16	73535987	G	31	10	T	0.0003611
16:76507762	16	76507762	T	6	22	TG	0.0007482
rs200278292	16	76628424	AT	12	0	A	0.0003422
rs881083	16	80652783	C	0	13	A	0.00005596
rs12446955	16	83903941	A	21	43	C	0.0003256
rs3943925	16	83906554	A	29	51	G	0.0005253
rs9888805	16	83907379	T	29	52	G	0.0002991
rs12149164	16	84451091	A	27	8	G	0.0006326
rs72799546	16	84860112	C	6	23	G	0.000429
rs9925686	16	84869448	T	0	10	G	0.0005917
rs370910839	16	84871165	G	0	10	GGCGCGTGACACCAC	0.0005917
rs2087991	16	84871171	T	1	13	C	0.0005059
rs8047416	16	84871409	T	0	10	C	0.0005917
rs72799559	16	84871799	C	0	10	G	0.0005917
rs11640864	16	85435872	A	5	20	G	0.0009659
rs8054346	16	86287812	T	32	55	C	0.000146
rs1510204	16	86293870	A	28	49	G	0.0008723
rs1704480	16	86294325	A	28	49	G	0.0008723
rs1391228	16	86294853	A	28	49	G	0.0008723
rs4589560	16	86925582	T	0	10	C	0.0005917
rs76497033	16	86926178	T	0	10	C	0.0005917
rs76866247	16	86926315	T	0	10	C	0.0005917
rs77154859	16	86926389	C	0	10	T	0.0005917
rs78362076	16	86926406	T	0	10	C	0.0005917
rs78089027	16	86926467	A	0	10	G	0.0005917
rs77544811	16	86926569	G	0	10	C	0.0005917
rs77789822	16	86926775	G	0	10	A	0.0005917
rs77118693	16	86926788	A	0	10	C	0.0005917
rs75519305	16	86926872	C	0	10	T	0.0005917

rs79553371	16	86927603	T	0	10 C	0.0005917
16:87304715	16	87304715	T	8	29 C	0.00003867
rs11076693	16	88718552	G	13	32 A	0.0009401
rs11654249	17	1165320	A	14	34 C	0.0004075
rs7212120	17	8922842	C	12	33 A	0.0002456
rs59696502	17	9918877	C	11	0 T	0.0007274
rs62058427	17	13073591	G	41	18 C	0.0007885
rs2188891	17	13087594	G	15	34 A	0.0008313
rs59192815	17	13092617	T	13	33 C	0.0005565
rs56236744	17	13100067	C	42	16 A	0.0001106
rs7211590	17	13139032	G	13	32 A	0.0009401
rs11078155	17	13340405	C	5	20 G	0.0009659
17:15378614	17	15378614	AGAAAAGAAAAGAAAG	7	24 A	0.0005947
rs12951975	17	31906265	G	37	14 A	0.0007953
rs2189331	17	32422691	T	32	55 G	0.0003175
rs4795156	17	35226986	C	21	4 T	0.0004069
rs62074890	17	36774263	A	18	3 G	0.0008121
rs62074891	17	36774985	T	18	3 C	0.0008121
rs62074892	17	36775006	T	18	3 C	0.0008121
rs2075052	17	36776981	T	18	3 G	0.0008121
rs12450767	17	36777102	A	18	3 G	0.0008121
rs12452655	17	36777205	T	18	3 C	0.0008121
rs62074896	17	36786201	T	18	3 A	0.0008121
rs56955572	17	36788631	TA	18	3 T	0.0008121
rs79553746	17	36789069	G	18	3 T	0.0008121
rs3848400	17	36790032	G	18	3 T	0.0008121
rs3848401	17	36790097	T	18	3 C	0.0008121
rs58164585	17	36791133	T	18	3 C	0.0008121
rs62074927	17	36793834	T	18	3 C	0.0008121
rs7224109	17	36964899	C	1	13 T	0.0004979
rs12937167	17	36996181	G	1	14 A	0.0002433
rs374270062	17	38360603	G	12	0 T	0.0003547
rs112247852	17	38360604	G	12	0 T	0.0003547
17:43122073	17	43122073	C	11	0 A	0.0007274
rs3809781	17	46692573	A	14	1 C	0.0006677
rs76487714	17	48611993	A	18	3 G	0.0008121
rs2306001	17	48615329	A	20	4 G	0.0007606
rs111678638	17	48619272	A	20	4 G	0.0007606
rs112657244	17	48619290	A	20	4 G	0.0007606
rs8066067	17	49407790	C	13	0 A	0.00016

17:52506523	17	52506523	G	16	2	GGAAGGAAA	0.0008338
rs8068233	17	55450074	A	33	12	G	0.0005855
rs9916654	17	55452531	A	35	13	G	0.0006539
rs1024821	17	55607947	A	54	27	T	0.000181
rs1035173	17	55608091	A	54	28	G	0.0003346
rs62058122	17	55692602	T	1	13	C	0.0005059
rs12602545	17	64010195	A	29	9	G	0.0004831
rs72042924	17	68475663	GA	29	52	G	0.0008265
rs4793394	17	68509868	A	31	52	G	0.0009496
rs4793395	17	68509948	C	31	52	T	0.0009496
rs9894256	17	68511145	A	31	52	G	0.0009496
17:70053962	17	70053962	A	0	10	G	0.0005917
rs4793411	17	70225574	C	15	35	T	0.0004795
rs4793412	17	70226159	G	15	35	A	0.0004795
rs11654234	17	72425499	A	2	15	G	0.0006243
rs2670824	17	72438972	C	2	16	G	0.0003115
rs9893663	17	72439700	T	2	16	G	0.0003115
rs2706530	17	72440381	G	2	16	A	0.0003115
rs2706528	17	72442416	A	2	15	C	0.0006243
rs28604312	17	76625195	A	35	12	G	0.0003064
rs12952072	17	80664054	G	28	9	A	0.0008484
rs12945524	17	80664081	T	28	9	G	0.0008484
rs12943159	17	80711299	A	28	9	G	0.0008484
rs4986111	17	80784476	A	29	8	G	0.0001956
rs12937095	17	80812193	A	39	16	G	0.0006105
rs12945629	17	80834447	A	39	16	G	0.0006105
rs34424116	17	80836559	T	29	8	G	0.0001956
17:80845004	17	80845004	ACT	35	13	A	0.0006539
rs12938554	17	80849295	T	38	15	C	0.0005267
rs4986129	17	80853554	A	29	8	G	0.0001956
rs12949628	17	80874290	G	29	8	T	0.0001956
rs11077949	17	80896115	T	36	14	C	0.0007756
rs3744156	17	80897215	T	37	15	C	0.0009053
rs3603	17	80901002	T	37	15	C	0.0009053
rs11077950	17	80901750	T	37	15	C	0.0009053
rs12940475	17	80902411	T	37	15	G	0.0009053
rs7225887	17	80904844	T	37	15	C	0.0009053
rs11653058	17	80909694	T	34	13	C	0.0006133
rs35355170	17	80928674	C	38	14	G	0.0002538
rs645951	18	8858624	A	54	29	G	0.0006016

rs113667478	18	9582320	TAGGG	13	33	T	0.0005413
rs60500709	18	9582322	G	13	33	C	0.0005413
18:12597172	18	12597172	C	34	11	CA	0.0001411
18:12597173	18	12597173	C	34	11	A	0.0001411
rs1787086	18	35309697	G	36	8	T	0.000001762
18:35309698	18	35309698	G	26	6	A	0.0001602
18:35309700	18	35309700	CGATCTGTCTAT	41	11	C	0.000002663
18:35309701	18	35309701	G	28	9	T	0.0007186
18:35309702	18	35309702	G	23	6	A	0.0009874
rs1806540	18	36001799	A	29	51	G	0.0005253
rs10853454	18	36005986	G	24	42	A	0.0009531
rs2226880	18	36010239	T	27	50	G	0.0004144
rs2014682	18	36010297	T	31	52	C	0.0009496
rs5010574	18	36011213	T	28	50	A	0.0005077
rs11082076	18	36012486	T	31	52	C	0.0009496
rs11665137	18	36013023	T	25	46	C	0.0007575
rs62079638	18	36013337	A	25	46	C	0.0007575
rs17638236	18	36015382	T	25	46	C	0.0007575
rs8090757	18	36015879	G	25	46	A	0.0007575
rs8094295	18	36015953	G	25	46	A	0.0007575
rs201179490	18	36016105	A	25	46	AG	0.0007575
rs7231316	18	36017968	T	25	46	G	0.0007575
rs7232682	18	36018202	G	25	46	T	0.0007575
rs7231411	18	36018288	C	25	46	A	0.0007575
rs7231559	18	36018294	A	25	46	C	0.0007575
rs7231875	18	36018532	G	25	46	A	0.0007575
rs55813611	18	36018965	T	25	46	C	0.0007575
rs11661114	18	36019152	C	25	46	T	0.0007575
rs7242958	18	36019921	C	25	46	T	0.0007575
rs7242071	18	36019959	A	25	46	G	0.0007575
rs7243119	18	36020002	C	25	46	T	0.0007575
rs7241992	18	36020096	T	28	51	C	0.0002892
rs7242327	18	36020316	T	25	46	A	0.0007575
rs17557806	18	36020670	T	25	46	C	0.0007575
rs11877851	18	36020743	C	25	46	G	0.0007575
rs958233	18	36021110	A	25	46	C	0.0007575
rs62079639	18	36021456	G	25	46	A	0.0007575
rs72403018	18	36021461	C	25	46	CAGTT	0.0007575
rs62079641	18	36021750	A	25	46	G	0.0007575
rs62079642	18	36021915	T	25	46	G	0.0007575

18:36309093	18	36309093	T	6	22	A	0.0008352
18:36709329	18	36709329	CACACAG	36	11	C	0.00004408
18:36709331	18	36709331	CACAG	36	11	C	0.00004408
rs203191	18	38054299	A	33	54	G	0.0009764
rs169395	18	38054353	G	33	54	T	0.0009764
rs55562	18	38057808	T	32	52	C	0.0007911
rs61371567	18	38058683	AT	33	54	A	0.0009764
rs203162	18	38059386	C	33	54	T	0.0009764
rs203172	18	38068321	T	33	54	C	0.0009764
rs4132081	18	38084525	C	33	55	T	0.0005652
rs4291983	18	38106410	C	33	55	A	0.0005652
rs9956350	18	38107168	G	33	55	A	0.0005652
rs7227822	18	38107825	C	33	55	G	0.0005652
rs8091417	18	38124303	T	33	55	C	0.0005652
rs656134	18	40472219	A	20	4	G	0.0007606
rs472591	18	40483622	A	26	7	G	0.0004518
rs2945505	18	47341544	G	35	56	A	0.0006468
rs9964783	18	47365005	A	43	20	C	0.0009741
rs640179	18	47385014	A	33	54	G	0.0009764
rs79368720	18	49343233	T	0	10	A	0.0005917
rs5824918	18	49707139	A	24	6	AT	0.0006264
rs17815367	18	49710900	G	24	6	A	0.0005592
rs4940169	18	49713609	T	24	6	C	0.0005592
rs4617958	18	49716105	G	24	6	A	0.0005592
rs57940982	18	57747471	G	16	2	A	0.0007845
rs11378817	18	57828305	GT	18	3	G	0.0008121
rs58237489	18	57838331	T	18	3	C	0.0008121
rs9960729	18	73090950	C	17	38	T	0.0003648
rs372076138	18	73824652	C	29	51	G	0.0005253
rs1660179	18	77361158	A	48	22	T	0.0002295
rs56401549	18	77771523	AAG	58	32	A	0.0005669
rs118096710	18	77773182	A	3	18	G	0.0007476
rs12965486	18	77773596	A	58	32	G	0.0005669
rs4799121	18	77774886	C	58	32	A	0.0005669
rs10853394	18	77775173	C	58	31	T	0.0002058
rs10853395	18	77775318	C	58	32	T	0.0005669
rs11504776	18	77776386	A	58	32	T	0.0005669
rs11504774	18	77776428	A	58	32	G	0.0005669
rs149018972	18	77777227	C	58	32	T	0.0005669
rs200969704	18	77777636	G	58	32	T	0.0005669

rs202234556	18	77777757	T	58	32	C	0.0005669
rs139108329	19	3023905	C	20	3	CAAAAGAAAAGA	0.0002428
rs8107652	19	7561410	C	34	55	T	0.0009818
rs11879017	19	11737612	A	18	39	G	0.0004141
rs8106273	19	11737927	G	17	39	A	0.0002057
rs8105182	19	11738117	T	17	40	G	0.0001953
rs8108668	19	11738656	C	17	39	G	0.0002057
rs10419625	19	11738975	T	18	38	C	0.0007166
rs113072274	19	11739226	C	17	39	T	0.0002057
rs62111855	19	11739315	A	17	39	C	0.0002057
rs62111856	19	11739342	A	18	38	T	0.0007166
rs62111857	19	11739413	A	18	39	G	0.0004141
rs4804616	19	11739489	C	27	49	G	0.0004874
rs111772256	19	11739991	T	27	49	C	0.0004874
rs113810971	19	11740059	A	26	50	G	0.0001475
rs4052625	19	11740813	G	27	49	A	0.0004874
rs7250798	19	11743819	C	27	50	T	0.0002777
rs8109499	19	11745927	T	27	50	C	0.0002777
rs201159596	19	11772307	A	3	23	T	0.00001103
rs117861106	19	13213801	T	0	10	C	0.0006636
rs74340166	19	15248549	A	0	10	G	0.0005917
19:17208857	19	17208857	A	11	0	C	0.000718
rs1859007	19	19162186	C	0	16	G	0.000005974
19:27738902	19	27738902	A	0	10	G	0.0005917
rs8104606	19	33888460	G	41	17	A	0.0004047
rs8106165	19	33888588	A	41	16	G	0.0001053
rs2918333	19	36750539	A	0	11	G	0.0002715
rs78341134	19	39005848	G	0	10	A	0.0005917
rs11083488	19	39400495	C	24	43	T	0.0006011
rs72261926	19	39411650	CACACAGAT	30	52	C	0.00054
rs12985428	19	39414109	A	23	45	G	0.0003849
rs10416055	19	39435485	G	23	44	A	0.0006657
rs2278412	19	39437270	G	23	44	A	0.0006657
rs1865047	19	39437534	T	23	44	C	0.0006657
rs1865049	19	39437560	A	23	44	G	0.0006657
rs73040938	19	40287769	T	22	5	G	0.0006676
rs77152874	19	40288189	T	21	4	C	0.000448
rs73042737	19	40330433	A	20	4	C	0.0007606
rs200521388	19	48488329	A	0	11	AAAAAAAG	0.0003046
19:48492369	19	48492369	T	2	22	TA	0.000003708

19:48492371	19	48492371	T	2	22	TTA	0.00001019
rs734570	19	49606449	T	5	21	C	0.0005184
19:55608122	19	55608122	C	17	1	CCGCAGTTCACCACCGTCTTCG(	0.00007285
20:530314	20	530314	GAAAGAAAT	0	11	G	0.0002715
20:2627769	20	2627769	TATTTATTG	0	10	T	0.0006636
rs6033045	20	11154329	T	35	13	C	0.0006539
rs6134147	20	11154839	C	35	13	A	0.0006539
rs6040404	20	11160363	C	35	13	T	0.0006539
rs2024689	20	11161752	C	35	13	T	0.0006539
rs6108856	20	11164295	T	35	13	C	0.0006539
rs2327342	20	11166478	A	35	13	C	0.0006539
rs1972192	20	11167617	T	35	13	C	0.0006539
rs6040418	20	11168405	T	35	13	C	0.0006539
rs6033050	20	11168518	A	35	13	G	0.0006539
rs6040420	20	11170323	G	35	13	A	0.0006539
rs6040422	20	11170748	A	35	13	T	0.0006539
rs6040423	20	11171070	G	35	13	A	0.0006539
rs2876217	20	11171441	C	35	13	T	0.0006539
rs4270418	20	11171828	C	35	13	T	0.0006539
rs113907634	20	11224146	AT	41	16	A	0.0001053
rs6040470	20	11229511	T	43	18	G	0.0002635
rs4632419	20	11232115	G	43	18	T	0.0002635
rs6040827	20	11645806	C	8	25	T	0.0009004
rs6134432	20	11991888	G	47	23	A	0.0007443
rs7270764	20	12007223	T	40	15	C	0.0001692
20:13179440	20	13179440	T	48	22	TC	0.0001103
20:13179441	20	13179441	T	48	22	C	0.0001867
20:13179442	20	13179442	T	48	22	TTTCTTTC	0.0001867
20:13179448	20	13179448	T	44	20	TC	0.000281
20:13179449	20	13179449	T	44	20	C	0.0003204
rs6079176	20	13780386	A	10	28	G	0.000853
20:20160978	20	20160978	GAA	0	11	G	0.0001744
20:24416229	20	24416229	A	1	13	G	0.0005059
rs77220650	20	29443602	A	3	17	T	0.0007114
rs79078695	20	32654817	G	4	21	T	0.0001601
rs79027916	20	32654818	A	4	21	C	0.0001601
rs77628996	20	40936657	G	2	16	T	0.0003115
rs3091552	20	45440006	C	35	12	G	0.0003064
rs6066089	20	45487371	C	37	15	G	0.0009053
rs67373293	20	49741626	T	3	17	C	0.0006426

rs201827584	20	51719420	T	3	17	TTA	0.0006426
rs201274209	20	51719425	A	3	18	ATAC	0.0003291
rs56158048	20	52477216	G	19	3	C	0.0004265
rs4811888	20	56182183	C	6	22	G	0.0007921
rs12625713	20	56182533	G	6	22	C	0.0007921
rs4811889	20	56183577	A	6	22	G	0.0007921
rs71181929	20	56183820	TG	6	22	T	0.0007921
rs4810089	20	56183918	T	6	22	C	0.0007921
rs62204276	20	56183980	C	6	22	T	0.0007921
rs167847	20	56521853	A	27	7	T	0.0002486
rs2426761	20	56526972	G	37	13	A	0.0002102
rs370087	20	56527490	T	37	13	C	0.0002102
rs62206222	20	56528595	T	27	7	C	0.0002486
rs354751	20	58905494	G	28	49	A	0.0008723
rs11480224	20	60013970	TA	22	5	T	0.0006676
rs4002829	20	60093065	CCTCAGCCT	2	16	C	0.0003115
rs6062322	20	62441599	T	28	9	A	0.0008484
rs376743203	21	9476942	A	11	0	G	0.0007274
21:9969858	21	9969858	T	0	10	C	0.0005917
rs1997577	21	16371102	T	5	20	A	0.0009659
rs7280064	21	16377650	T	5	20	C	0.0009659
rs139790892	21	16377682	GC	5	20	G	0.0009659
rs2823006	21	16380994	C	5	20	G	0.0009659
21:16479311	21	16479311	G	2	18	T	0.00008557
rs66681115	21	18238562	C	45	21	CA	0.0006315
rs205676	21	19484268	C	26	7	T	0.0004518
rs60962400	21	24791277	G	16	2	GA	0.0007845
rs9982530	21	24794721	G	36	14	A	0.0007756
rs462242	21	27569980	T	20	4	C	0.0007606
rs9983828	21	32630308	C	28	52	T	0.0001612
21:33240138	21	33240138	A	0	11	G	0.0002715
21:36745167	21	36745167	A	22	43	ATATAT	0.0006167
rs722793	21	40405106	A	0	10	G	0.0005917
rs2410079	21	40406395	T	0	10	C	0.0005917
rs760159	21	40406734	G	0	10	T	0.0005917
rs760158	21	40406983	T	0	10	A	0.0005917
rs10617614	21	40407223	GTCA	0	10	G	0.0005917
rs6517504	21	40407248	G	0	10	T	0.0005917
rs516865	21	40937191	C	26	7	T	0.0008354
rs112619200	21	40937832	AC	30	9	A	0.0002709

rs523372	21	40937929	G	30	9	T	0.0002709
21:40938042	21	40938042	AT	30	9	A	0.0002709
21:40938043	21	40938043	GACGTAGAAAT	30	9	G	0.0002709
rs596320	21	40938269	C	30	9	T	0.0002709
rs550774	21	40938562	C	30	9	T	0.0002709
rs551624	21	40938643	T	30	9	C	0.0002709
rs551830	21	40938715	T	29	9	C	0.0004831
rs503729	21	40939493	C	30	9	G	0.0002709
rs506387	21	40939762	T	30	9	C	0.0002709
rs658066	21	40940223	A	30	9	G	0.0002709
rs538329	21	40940934	G	36	15	C	0.0008629
rs538333	21	40940939	C	29	9	A	0.0004831
rs561166	21	40941132	G	30	9	T	0.0002709
rs2837083	21	40941250	A	29	9	G	0.0004831
rs59155174	21	40941274	G	30	9	A	0.0002709
rs562285	21	40941308	G	30	9	C	0.0002709
rs563230	21	40941401	T	30	9	C	0.0002709
rs563259	21	40941412	T	30	9	C	0.0002709
rs564126	21	40941498	T	29	9	C	0.0004831
rs630270	21	40941891	G	30	9	A	0.0002709
rs567837	21	40941914	G	29	9	C	0.0004831
rs629432	21	40942040	G	29	9	A	0.0004831
rs628995	21	40942130	A	29	8	T	0.0001956
rs617259	21	40942493	T	29	9	C	0.0004831
rs602107	21	40943606	A	28	9	T	0.0008484
rs542531	21	40944237	A	29	9	C	0.0004831
rs34797170	21	40944570	C	29	9	CT	0.0004831
rs585092	21	40945028	T	29	9	G	0.0004831
rs376474175	21	40945600	G	24	6	GTA	0.0005592
rs1734930	21	40946124	A	29	9	G	0.0004831
rs636599	21	40947315	C	29	9	G	0.0004831
rs2837398	21	41427168	C	31	52	A	0.0009496
rs2837401	21	41428674	G	31	52	C	0.0009496
rs2837403	21	41429463	A	31	52	G	0.0009496
rs2837407	21	41433935	A	55	30	G	0.0006201
rs2837411	21	41436645	T	55	30	C	0.0006201
rs9636974	21	42144932	G	21	43	A	0.0003256
rs7277230	21	42147572	T	21	43	C	0.0003256
rs6517614	21	42150494	T	21	41	A	0.0009669
rs62223059	21	42150902	T	21	41	A	0.0009669

rs7277165	21	42154583	A	21	41	G	0.0009669
rs7277205	21	42154651	A	21	41	G	0.0009669
rs35336179	21	42178219	T	22	43	C	0.0006167
rs35463403	21	45860024	A	45	21	G	0.0006315
rs2236672	21	45863462	G	32	11	A	0.0004661
rs73233027	21	45897827	G	0	10	T	0.0005917
rs146490633	21	45899411	C	0	10	G	0.0005917
rs62214403	21	46664698	A	11	0	G	0.0007274
rs4818845	21	47966016	G	20	4	C	0.0007606
rs2522310	22	17887725	A	28	8	G	0.0003544
22:21410890	22	21410890	T	0	10	C	0.0004558
rs12627889	22	22739955	A	0	10	G	0.0005917
rs4586741	22	22740278	G	0	11	A	0.0002715
22:23103428	22	23103428	A	34	11	G	0.0001478
rs9612496	22	24221405	C	23	44	G	0.0006657
rs9612497	22	24221445	T	23	44	C	0.0006657
rs5760088	22	24232044	C	58	32	T	0.0005669
rs9621351	22	32248370	G	3	17	A	0.0006426
rs2157212	22	32262530	A	2	15	C	0.0006243
rs5994440	22	32266157	T	3	17	A	0.0006426
rs737084	22	32268400	G	3	17	A	0.0006426
rs9621357	22	32275852	A	3	17	G	0.0006426
rs738201	22	32665756	C	41	17	A	0.0004047
rs132710	22	36593245	G	30	9	A	0.0002709
rs5995288	22	36762634	T	43	20	C	0.0009741
rs933224	22	36766003	T	44	20	C	0.0005739
rs6000262	22	36770038	A	42	19	G	0.0008809
rs13054971	22	37585922	C	6	22	T	0.0007921
rs13056128	22	37587705	A	6	22	T	0.0007921
rs35593514	22	37600011	C	6	22	G	0.0007921
22:39279512	22	39279512	T	0	10	TTTTCTTTTCTTTTCTTTTCTTTT(	0.0005846
rs11704096	22	44504928	A	16	35	T	0.000956
rs71918181	22	44504958	G	16	35	GT	0.000956
rs80680	22	45911590	G	16	2	C	0.0007845
rs4990141	22	48318002	T	17	37	C	0.0006349
rs5768176	22	48322535	A	20	41	G	0.0005156
X:293456	23	293456	A	20	40	AC	0.0008835
X:1649057	23	1649057	G	26	7	C	0.0004518
X:2427310	23	2427310	T	19	3	C	0.0004265
rs6642024	23	2723666	C	12	36	T	0.00002328

rs11797301	23	2723715	G	8	29 T	0.00007515
rs11152547	23	2724389	A	8	27 T	0.0002842
rs7058222	23	2724429	C	8	27 A	0.0002641
rs7062707	23	2724448	A	8	27 T	0.0002641
rs35902344	23	2725036	CAT	10	34 C	0.00002152
rs10709559	23	2729156	C	9	34 CT	0.00001697
rs5939336	23	2729281	A	17	39 G	0.00008931
rs200022655	23	2733843	C	10	32 CAT	0.00008291
rs5939340	23	2734838	G	10	30 A	0.0002687
rs5939341	23	2734930	G	8	32 C	0.00001016
rs113131499	23	2734948	AC	8	30 A	0.00001963
rs5939342	23	2735539	G	12	33 A	0.0002456
rs5939343	23	2735621	T	12	33 C	0.0002456
rs5939344	23	2735741	T	12	33 A	0.0002456
rs6642031	23	2735926	G	12	33 A	0.0002456
rs35714686	23	2736120	TATTA	12	33 T	0.0002456
rs5939126	23	2736196	C	12	33 A	0.0002456
rs1809566	23	2736301	C	12	33 G	0.0002456
rs2316290	23	2736503	C	12	33 G	0.0002456
rs6567653	23	2737097	G	12	33 A	0.0002456
rs2018620	23	2737149	C	12	33 T	0.0002456
rs5982869	23	2737194	C	12	33 T	0.0002456
rs901321	23	2737282	A	12	31 G	0.0004121
rs34306434	23	2737833	TC	13	33 T	0.0005413
rs5939128	23	2737851	G	13	32 T	0.0009401
rs62582293	23	2747703	T	3	18 C	0.0002953
rs57068689	23	2748051	T	5	26 C	0.00002087
rs6641656	23	2748292	C	7	26 G	0.0001833
rs5939348	23	2748421	G	7	26 A	0.0001994
rs73635389	23	2925674	T	11	0 C	0.0007274
rs12398173	23	3454292	G	34	10 C	0.00006069
rs113924529	23	3454890	A	19	40 G	0.0007691
rs7473053	23	3457060	T	29	52 C	0.0002991
rs11152506	23	3458348	A	29	52 G	0.0002991
rs6567560	23	3459334	C	29	52 T	0.0002991
rs7474188	23	3459801	T	29	52 C	0.0002991
rs66534918	23	3464865	A	57	31 G	0.0007953
rs7473251	23	3472918	A	20	40 G	0.0008835
rs200267680	23	3473257	C	27	54 T	0.0000235
rs201390777	23	3473264	C	30	52 G	0.0002835

rs11152516	23	3473683	A	29	53	T	0.00008254
rs56004217	23	3474092	C	28	55	G	0.00002438
rs12397089	23	3474097	A	58	32	G	0.0005669
rs55992753	23	3474169	A	21	41	G	0.0008957
rs56038351	23	3474189	A	27	52	G	0.0001468
rs5961570	23	3474209	T	57	32	A	0.0005282
rs145303554	23	3474660	C	31	50	T	0.0008282
rs201951922	23	3474856	C	36	11	T	0.0001415
rs11152518	23	3475928	A	28	53	G	0.00008787
rs10449092	23	3477462	A	28	53	G	0.00008787
rs142408883	23	3477623	C	28	53	T	0.00008787
rs2041956	23	3478950	G	28	47	C	0.0006892
rs2041955	23	3479365	C	28	53	T	0.00008787
rs12847107	23	3484808	A	36	13	C	0.0003739
rs10442503	23	3489155	T	37	13	A	0.0002102
rs72619400	23	3509853	T	55	27	C	0.0001001
rs6567564	23	3510277	G	34	55	A	0.0009818
rs370663764	23	3547287	C	24	6	CT	0.0006157
rs17335289	23	3646417	A	31	11	C	0.0008085
rs7882598	23	3648159	G	36	14	C	0.000808
rs5962262	23	3648286	C	36	14	T	0.0007756
rs17335296	23	3648354	G	32	11	A	0.0004661
rs112303448	23	3648503	GAC	36	14	G	0.0007756
rs4826736	23	3648717	C	36	14	T	0.0007756
rs5915863	23	3649629	G	35	10	A	0.00003249
rs12688330	23	3649810	A	35	10	G	0.00003249
rs12687129	23	3649902	C	36	10	T	0.00003012
rs5915463	23	3650763	T	33	7	C	0.000009842
rs5915465	23	3650958	A	33	9	G	0.00004367
rs199679801	23	3651227	ATGGAGTTGCTC	33	9	A	0.00004367
rs5961720	23	3655467	G	32	10	A	0.0003465
rs4826685	23	3655560	C	32	10	A	0.0003465
rs5916123	23	3655986	T	19	42	C	0.0001482
rs4826699	23	3656889	C	32	10	T	0.0002023
rs58253239	23	3661855	C	29	10	A	0.0004412
rs191848608	23	3661859	C	29	10	A	0.0004412
rs60181494	23	3661863	C	29	10	A	0.0004412
rs6641619	23	3663728	G	28	9	A	0.0008484
rs6641876	23	3664074	C	28	8	T	0.0003544
rs59003681	23	3664764	G	28	8	A	0.0003544

rs67132522	23	3665013	C	28	5	T	0.00001652
rs73444704	23	3665110	T	28	7	G	0.0001348
rs67926068	23	3665113	G	28	7	A	0.0001348
rs6641877	23	3665204	A	28	7	G	0.0001348
rs5915772	23	4591677	T	10	29	C	0.0004811
rs5916687	23	4596138	A	10	31	G	0.0001457
rs5916688	23	4596415	T	10	31	C	0.0001457
rs12848207	23	4601110	A	10	32	C	0.00007826
rs3085321	23	4622803	G	55	30	GAT	0.0006201
rs5916696	23	4627822	A	23	44	G	0.0006657
rs7881910	23	4628697	A	58	31	C	0.0002058
rs5916697	23	4628916	T	32	55	C	0.0003175
rs17315343	23	5915431	C	2	17	T	0.000131
rs72609517	23	6040845	G	0	11	A	0.0007003
rs200717890	23	6041758	GGAAAA	0	11	G	0.0002676
rs1534257	23	8357507	T	28	9	C	0.0008484
rs1534256	23	8357650	C	28	9	T	0.0008484
rs2404238	23	8357913	C	28	9	T	0.0008484
rs56408029	23	8375974	A	28	9	G	0.0008484
rs56067383	23	8376177	A	28	9	AC	0.0008484
rs67848764	23	8376750	T	28	9	A	0.0008484
rs7058555	23	8376774	G	28	9	C	0.0008484
rs7057664	23	8376875	A	28	9	G	0.0008484
rs5934413	23	8379511	T	28	9	C	0.0008484
rs55853704	23	8933953	T	26	47	C	0.0007993
rs5979053	23	9202415	T	4	20	C	0.0003114
rs35252422	23	9296151	CGTGT	17	2	C	0.0004014
rs17321120	23	9685901	C	3	21	T	0.00003567
rs4830653	23	9732976	G	2	16	A	0.0002695
rs58367116	23	9904364	C	30	8	T	0.0000914
rs186107305	23	11865681	A	0	10	G	0.0005917
rs5933925	23	11866903	C	0	10	A	0.0005917
rs5935176	23	11888070	C	0	10	T	0.0005917
rs6629958	23	14291313	C	50	24	T	0.0002688
rs137905720	23	14325441	C	24	45	CCTT	0.0007128
rs7063332	23	14325565	G	24	45	A	0.0007128
rs929210	23	14326895	A	25	46	G	0.0007575
rs1015805	23	14329490	G	24	45	C	0.0007128
rs1016539	23	14333489	T	24	45	G	0.0007128
rs11797584	23	14335690	T	24	45	C	0.0007128

rs5935726	23	14336239	A	24	45	G	0.0007128
rs5935728	23	14339619	A	24	45	G	0.0007128
rs5978678	23	14340631	T	22	43	C	0.000565
rs5934166	23	14340866	T	24	45	C	0.0007128
rs5935729	23	14341240	T	24	45	C	0.0007128
rs5935730	23	14341322	C	24	45	G	0.0007128
rs5935731	23	14341387	C	24	45	T	0.0007128
rs2023720	23	14341735	C	24	45	G	0.0007128
rs1468160	23	14343597	C	30	53	T	0.0003072
rs5935734	23	14344569	C	25	46	T	0.0004176
rs2375482	23	14347263	G	24	46	A	0.0004131
rs147454328	23	14358279	T	24	45	TA	0.0007128
rs6527696	23	16682119	T	55	28	C	0.000189
rs6527697	23	16682120	G	56	28	A	0.0001045
rs6632862	23	16682634	A	55	30	G	0.0006201
rs6632863	23	16682662	C	55	30	T	0.0006201
rs6632864	23	16685391	G	53	25	T	0.0000896
rs150007863	23	19089203	G	1	13	A	0.0005059
rs6633367	23	20539729	G	3	17	A	0.0006426
rs201976263	23	20542988	T	3	17	TCA	0.0006426
rs7066051	23	20547872	C	3	17	G	0.0006426
rs12393059	23	20548370	T	3	17	C	0.0006426
rs73195325	23	20552304	G	3	17	A	0.0006426
rs114595430	23	20553163	T	3	17	G	0.0006426
rs73197242	23	20553808	G	3	17	A	0.0006426
rs73197243	23	20554789	C	3	17	A	0.0006426
rs13440692	23	20561825	C	3	17	T	0.0006426
rs7876243	23	20563226	C	3	17	T	0.0006426
rs73197245	23	20566238	A	3	17	G	0.0006426
rs73197246	23	20567524	A	3	17	G	0.0006426
rs35059902	23	20568060	CT	3	17	C	0.0006426
rs6633370	23	20572466	A	3	17	G	0.0006426
rs139700732	23	20574394	C	3	17	T	0.0006426
rs114937107	23	20576035	T	3	17	C	0.0006426
rs73197249	23	20583665	T	3	17	C	0.0006426
rs73197250	23	20585189	A	3	17	G	0.0006426
rs73197252	23	20587501	A	3	17	G	0.0006426
rs370229060	23	20592701	C	2	17	CCAAA	0.0001534
rs73197255	23	20606076	T	2	15	C	0.0006243
rs5925658	23	22761345	C	53	25	T	0.0000896

rs2465828	23	23140838	G	17	41	A	0.0001079
rs73205366	23	23399373	C	0	11	A	0.0002715
rs73205368	23	23399501	C	0	11	T	0.0002715
rs5971115	23	23405681	T	28	52	G	0.0001612
rs6627921	23	23406394	G	28	50	C	0.0005077
rs6627922	23	23406598	G	23	50	A	0.00001837
rs6418526	23	23406691	C	29	51	G	0.0005253
rs6627923	23	23406797	G	24	50	A	0.00003799
rs3810719	23	23415889	G	30	51	A	0.0009286
rs181821571	23	23442241	T	0	10	C	0.0005917
rs5926337	23	23465760	C	19	1	T	0.00001683
rs5926339	23	23470309	C	56	31	T	0.0006352
rs5971138	23	23470730	C	56	31	A	0.0006352
rs5925778	23	23472193	G	56	31	C	0.0006352
rs202224294	23	23474528	T	57	31	TTATTGCTC	0.0003656
rs5925780	23	23475262	C	57	31	G	0.0003656
rs2885476	23	23476042	T	56	32	C	0.0004905
rs7052935	23	23480785	C	57	30	T	0.0002015
rs7888798	23	23482692	C	56	30	T	0.0003574
rs4278290	23	23482926	A	56	30	C	0.0003574
rs4828863	23	23495802	T	40	16	G	0.0003516
rs4317728	23	23496583	G	55	30	C	0.0006201
rs5925789	23	23497147	C	55	26	T	0.00005151
rs5970583	23	23497728	G	55	30	T	0.0006201
rs4593719	23	23497951	C	55	30	T	0.0006201
rs4240085	23	23498240	C	55	30	T	0.0006201
rs6526294	23	23498826	G	56	30	C	0.0003574
rs4828813	23	23501880	T	54	28	C	0.0003346
rs5970585	23	23502925	A	53	28	C	0.00058
rs7062129	23	23506555	T	53	28	C	0.00058
rs5925796	23	23509238	T	56	30	C	0.0003574
rs61604846	23	23509794	GT	56	30	G	0.000584
rs4408049	23	23511493	A	54	28	C	0.0003346
rs5970737	23	23513708	C	56	30	T	0.0003574
rs35272218	23	23514456	AT	55	30	A	0.0005501
rs4828866	23	23518802	C	54	31	T	0.0007456
rs4828867	23	23521028	A	37	14	G	0.0004475
rs11094927	23	23533219	C	57	30	T	0.0002015
rs7054852	23	23533794	C	57	30	A	0.0002015
rs11094928	23	23535091	C	57	30	T	0.0002015

rs12394178	23	23536023	T	57	30	A	0.0002015
rs12388791	23	23536126	A	55	30	G	0.0006201
rs12394282	23	23536177	T	54	29	A	0.0006016
rs55794528	23	23536430	A	57	30	T	0.0002015
rs55918751	23	23536683	G	57	30	A	0.0002015
rs4551501	23	23538505	C	56	30	G	0.0003574
rs76329348	23	23538812	A	55	30	G	0.0006201
rs201371812	23	23539122	GATTTGTTTGAGTTC	56	30	G	0.0003574
rs4828869	23	23539695	G	58	31	A	0.0002058
rs4828870	23	23539699	C	58	31	T	0.0002058
rs4828815	23	23540435	C	57	30	T	0.0002015
rs7064719	23	23541432	A	13	35	G	0.0001638
rs5970745	23	23542870	A	18	38	G	0.0007166
rs5925817	23	23548275	A	52	28	G	0.0009847
rs6629690	23	23598811	G	55	28	A	0.000189
rs192430333	23	26150490	G	14	1	C	0.000663
rs143450119	23	26153200	T	14	1	C	0.0006347
rs141122379	23	26222568	A	14	1	G	0.0006347
rs146979379	23	26265498	C	14	1	G	0.0006347
rs145026071	23	26293486	G	14	1	A	0.0006347
rs140699821	23	26341000	T	14	1	G	0.0006347
rs5926577	23	28323036	T	28	7	A	0.0001348
rs12391023	23	28323501	A	28	7	C	0.0001348
rs12856271	23	28323519	G	28	7	T	0.0001348
rs35197417	23	28326346	T	28	7	C	0.0001348
rs10449058	23	28433024	C	0	10	A	0.0005917
rs12387440	23	28770293	G	30	8	A	0.0001064
rs10521946	23	28770685	A	30	8	G	0.0001064
rs12846361	23	28776628	G	30	8	A	0.0001064
rs10521948	23	28839903	A	22	5	G	0.0006676
rs35096792	23	28850298	C	22	5	G	0.0006676
rs1842879	23	28851539	A	22	4	C	0.0002148
rs199910251	23	28852390	ATCT	22	5	A	0.0006676
rs55870411	23	29366069	A	27	48	G	0.0008377
rs145046946	23	29937522	G	0	10	A	0.0005917
rs12556783	23	30513862	C	15	1	T	0.0003131
rs202070431	23	30571258	A	14	1	G	0.0006286
rs201069997	23	30571260	A	14	1	G	0.0006286
rs2405153	23	31850711	C	59	30	G	0.00005988
rs1463493	23	31852903	G	16	41	A	0.0000497

rs6653576	23	31853688	A	18	41	T	0.0001308
rs6653577	23	31853768	C	18	39	T	0.0004141
rs331318	23	32324466	C	56	31	T	0.0006352
rs3788900	23	32352246	T	25	46	C	0.0007575
rs721422	23	32352714	T	25	46	C	0.0007575
rs4829252	23	32356321	C	27	49	T	0.0004874
rs926953	23	32364660	T	25	46	C	0.0007575
rs6527189	23	32374317	T	26	50	A	0.0001475
rs145839936	23	32374759	GGTTA	30	51	G	0.0009286
rs113982864	23	32375099	T	2	18	C	0.00007463
rs67626720	23	32375433	T	2	18	C	0.00006478
rs5927972	23	32376945	G	29	50	A	0.0009027
rs57445410	23	32377166	A	2	19	T	0.00003587
rs41303181	23	32408311	C	0	10	T	0.0005917
rs7882841	23	32416812	C	3	17	T	0.0006426
rs60017465	23	32418245	C	3	17	T	0.0006426
rs7891076	23	32418611	T	3	17	C	0.0006426
rs60364627	23	32419451	TA	3	17	T	0.0006426
rs962012	23	32420096	G	3	17	C	0.0006426
rs962013	23	32420335	T	3	17	C	0.0006426
rs56152215	23	32421209	C	3	17	G	0.0006426
rs55811023	23	32421430	C	3	17	T	0.0006426
rs56132370	23	32421613	G	3	17	A	0.0006426
rs16990306	23	32422265	A	3	17	G	0.0006426
rs56395123	23	32422846	A	3	17	G	0.0006426
rs66506532	23	32423285	G	3	17	A	0.0006426
rs16990311	23	32423481	T	3	17	C	0.0006426
rs10598359	23	32423605	A	3	17	AAG	0.0006426
rs7882139	23	32424108	C	3	17	G	0.0006426
rs187302049	23	32424276	T	3	17	C	0.0006426
rs2207798	23	32424896	G	3	17	A	0.0006426
rs2224126	23	32424901	C	3	17	T	0.0006426
rs142815666	23	32425158	A	3	17	T	0.0006426
rs149976886	23	32425179	T	3	17	C	0.0006426
rs144342404	23	32425218	A	3	17	C	0.0006426
rs113225073	23	32426218	C	3	17	T	0.0006426
rs73619075	23	32426260	A	3	17	G	0.0006426
rs73619076	23	32428012	T	3	17	C	0.0006426
rs55768447	23	32428228	G	3	17	A	0.0006426
rs10521994	23	32428596	A	3	17	G	0.0006426

rs56094071	23	32430503	T	3	17	A	0.0006426
rs10521995	23	32430767	T	3	17	C	0.0006426
rs55717900	23	32431030	C	3	17	T	0.0006426
rs113058119	23	32431084	T	3	17	C	0.0006426
rs58067019	23	32431237	T	3	17	C	0.0006426
rs3761606	23	32432718	G	4	20	A	0.0003114
rs10521996	23	32432855	G	4	20	C	0.0003114
rs12392232	23	32434506	C	3	17	A	0.0006426
rs16990333	23	32434554	G	3	17	A	0.0006426
rs10521997	23	32435412	T	3	20	C	0.0000828
rs16998301	23	32436410	A	3	17	G	0.0006426
rs10521998	23	32437853	C	3	20	A	0.0000828
rs3827460	23	32437885	C	4	20	T	0.0003114
rs56266543	23	32437990	A	4	20	AAGCATTG	0.0003114
rs12387844	23	32438873	G	4	20	A	0.0003114
rs60403469	23	32439300	A	3	17	G	0.0006426
rs145233065	23	32439554	GA	3	20	G	0.0000828
rs16990354	23	32439579	T	3	20	C	0.0000828
rs3788905	23	32439812	C	5	20	T	0.0009659
rs7064842	23	32439889	G	3	20	C	0.0000828
rs7064732	23	32439928	G	3	20	A	0.0000828
rs7064854	23	32439932	A	3	20	C	0.0000828
rs7065395	23	32440087	A	3	20	G	0.0000828
rs7066496	23	32440258	A	3	20	T	0.0000828
rs7065493	23	32440404	T	3	20	A	0.0000828
rs7051685	23	32440587	A	5	20	G	0.0009659
rs10521999	23	32440977	G	5	20	A	0.0009659
rs7053451	23	32441181	C	5	20	T	0.0009659
rs16998308	23	32442346	C	3	20	T	0.0000828
rs58831952	23	32442538	T	3	20	A	0.0000828
rs55718503	23	32443913	T	3	20	A	0.0000828
rs56234042	23	32444222	G	3	17	C	0.0006426
rs12007963	23	32444463	A	5	20	C	0.0009659
rs12012720	23	32444870	C	3	20	T	0.0000828
rs73619077	23	32444911	A	3	17	G	0.0006426
rs7050950	23	32446098	T	3	20	G	0.0000828
rs7051000	23	32446301	T	5	20	C	0.0009659
rs60545338	23	32446316	G	5	20	A	0.0009659
rs56787928	23	32446701	A	5	20	G	0.0009659
rs73619079	23	32447015	T	3	20	C	0.0000828

rs73619080	23	32447177	A	3	20	G	0.0000828
rs10522001	23	32447855	G	5	20	A	0.0009659
rs57582722	23	32448139	G	3	20	T	0.0000828
rs73619083	23	32449948	A	3	20	G	0.0000828
rs73619084	23	32450026	A	5	20	G	0.0009659
rs16990375	23	32450742	C	3	20	T	0.0000828
rs6631580	23	32453737	T	3	17	C	0.0006426
rs12559442	23	32461630	A	1	13	G	0.0005059
X:32624859	23	32624859	T	3	18	TAC	0.0003291
rs199511492	23	33509576	T	2	15	TAAATA	0.0006243
rs12156989	23	33540856	T	2	15	C	0.0006243
rs5928322	23	33547621	C	2	15	T	0.0006243
rs202044715	23	33551598	GA	2	15	G	0.0005586
rs5928325	23	33554170	C	5	20	G	0.0009659
X:33556441	23	33556441	CA	2	15	C	0.0006243
rs5928328	23	33557903	C	2	15	A	0.0006243
rs142445332	23	33567684	C	2	15	T	0.0006243
rs34213377	23	34039627	T	0	11	G	0.0002715
rs79656436	23	34066593	G	0	11	A	0.0002355
rs4827015	23	39360766	A	21	41	G	0.0009669
rs35162982	23	39361891	AT	21	41	A	0.0009669
rs4827159	23	39363899	G	21	41	A	0.0009669
rs5917842	23	39367677	T	21	41	C	0.0009669
rs4827016	23	39369141	T	21	41	C	0.0009669
rs5917310	23	39369598	C	21	42	T	0.0005665
rs902256	23	39371271	C	21	42	T	0.0005665
rs5963667	23	39372355	C	21	42	T	0.0005665
rs5963668	23	39372465	A	21	43	G	0.0003256
rs140937319	23	41261816	A	4	19	C	0.0005969
X:41868970	23	41868970	A	0	10	G	0.0005917
rs73624973	23	41975911	G	16	2	A	0.0007845
rs17214625	23	41978484	G	16	2	A	0.0007845
rs17280548	23	41981146	G	16	2	A	0.0007845
rs76657601	23	42085019	A	21	4	G	0.0008791
rs1207265	23	46095451	A	20	42	T	0.0002952
rs34139282	23	46096980	TATAG	20	42	T	0.0001632
rs1207263	23	46098697	A	20	42	T	0.0002952
rs1207270	23	46112988	T	20	41	C	0.0005156
rs5906176	23	46189488	G	54	29	C	0.0006016
rs5906178	23	46192269	T	54	29	C	0.0006016

rs6521095	23	46194662	G	55	30	A	0.0006201
rs5906179	23	46194689	C	55	30	A	0.0006201
rs2355334	23	46199816	C	54	29	G	0.0006016
rs5952855	23	46202327	G	54	29	A	0.0006016
rs4239960	23	46203667	C	54	29	A	0.0006016
rs5906180	23	46203987	C	54	29	T	0.0006016
rs5952864	23	46234850	C	52	29	T	0.0007301
rs5905532	23	46235267	T	52	27	C	0.0004991
rs73630223	23	46247540	T	55	29	G	0.0005668
rs57903655	23	47386025	CT	5	21	C	0.0005044
rs13328514	23	47393711	G	13	33	C	0.0005413
rs13328515	23	47393752	G	13	33	C	0.0005413
rs5906422	23	47398816	A	11	31	G	0.0003471
rs5906423	23	47398889	A	11	31	T	0.0003471
rs5953051	23	47401302	C	10	31	T	0.0001457
rs199965927	23	47401422	AAAAC	10	31	A	0.0001457
rs73206116	23	47402046	G	6	22	A	0.0007921
rs5952437	23	47402470	C	9	29	T	0.0001986
rs11450456	23	47403970	CA	10	28	C	0.000853
rs5906427	23	47407427	G	10	29	T	0.0004811
rs5953057	23	47410025	T	10	29	C	0.0004811
rs67848181	23	47411823	T	8	28	C	0.000142
rs4824621	23	47450276	C	9	27	T	0.000654
rs4824622	23	47450580	C	9	27	T	0.000654
rs62592125	23	47450949	A	9	27	G	0.0003604
rs5953061	23	47451476	C	9	27	T	0.000654
rs5953062	23	47451559	A	9	27	G	0.000654
rs73206134	23	47453025	C	5	21	T	0.0005184
rs5953063	23	47454598	A	9	27	G	0.000654
rs5906437	23	47455346	G	9	27	A	0.000654
rs9887335	23	47459125	G	9	27	A	0.000654
rs5953066	23	47461549	T	9	27	C	0.000654
rs4824424	23	47462630	T	9	27	C	0.000654
rs4824623	23	47462631	G	9	27	T	0.000654
rs149060491	23	48970697	A	13	0	G	0.00016
rs142430804	23	48985571	A	12	0	G	0.0003422
rs202078647	23	49057656	C	12	0	CAG	0.0003422
rs73209799	23	49160980	A	12	0	G	0.0003422
rs148107284	23	49399422	A	12	0	G	0.0003422
rs2028329	23	51574091	C	2	15	G	0.0006243

rs145718598	23	51843635	T	2	15	A	0.0006243
rs143928923	23	51906541	A	2	15	C	0.0006243
rs4986582	23	53044825	A	6	23	T	0.000429
rs5991831	23	53045870	T	6	23	C	0.000429
rs5991832	23	53045948	T	6	23	C	0.000429
rs5951137	23	53049271	T	6	23	C	0.000429
rs5951152	23	53051705	T	6	23	C	0.0004533
rs5951153	23	53051782	T	6	23	G	0.0003888
rs5951139	23	53052155	C	6	24	T	0.0002208
rs5951140	23	53052302	C	7	24	T	0.0006277
rs6643517	23	53053254	C	6	23	T	0.000429
rs6643602	23	53056259	G	6	23	A	0.000429
rs2495795	23	54095232	C	2	16	T	0.0003115
rs150018032	23	70094743	A	20	2	G	0.00005017
rs201334832	23	70105023	TG	17	2	T	0.0004014
rs4524964	23	70144801	T	14	33	C	0.0007122
rs11291509	23	70149333	A	14	34	AC	0.0003416
rs5936607	23	70149404	C	22	42	T	0.0006025
rs62606971	23	71023406	A	14	34	G	0.0004075
rs376880157	23	71307811	T	12	0	TTGGGAGGCCGAGGCGGGCTG	0.0003422
rs202226560	23	71309707	C	12	0	CA	0.0003422
rs140325071	23	71325372	A	12	0	G	0.0003422
rs12006827	23	71337452	A	12	0	G	0.0003422
rs12393772	23	71341981	T	12	0	C	0.0003422
rs34677932	23	71342420	A	12	0	AT	0.0003422
rs12388804	23	71343433	T	12	0	C	0.0003422
rs73565816	23	71343535	A	12	0	G	0.0003422
rs73565818	23	71344760	A	12	0	G	0.0003422
rs112253440	23	71344767	G	12	0	T	0.0003422
rs56290856	23	78037890	AT	52	27	A	0.0005555
rs10909066	23	78041561	A	52	27	G	0.0005555
rs12559776	23	78794135	A	47	23	C	0.0007443
rs34858870	23	78934852	T	28	49	TA	0.0008723
rs6523980	23	79788147	T	28	9	G	0.0008484
rs77230460	23	79847201	A	28	9	G	0.0007186
rs147276218	23	80077905	G	33	11	A	0.0002645
rs143517032	23	80080225	A	33	11	G	0.0002645
rs199777134	23	80080622	CA	33	11	C	0.0002645
rs6524023	23	80084448	G	33	11	A	0.0002645
rs5959043	23	80084885	A	33	11	T	0.0002645

rs12689552	23	80086115	G	33	11	T	0.0002645
rs6524024	23	80087539	T	33	11	A	0.0002645
rs5959044	23	80092224	T	33	11	G	0.0002645
rs6622374	23	80094000	C	33	11	A	0.0002645
rs151000551	23	80167991	T	18	3	A	0.0008121
rs182706350	23	80717633	C	0	10	T	0.0005917
rs149477376	23	80730143	T	0	10	C	0.0005917
rs73237376	23	81231297	A	1	14	G	0.0002433
rs114545707	23	81312969	C	1	14	A	0.0002433
rs142224359	23	81466433	A	0	10	G	0.0005917
rs141990906	23	81476209	A	0	10	G	0.0005917
rs200676876	23	81657539	A	0	10	ATC	0.0005917
rs139759794	23	81810102	G	0	10	C	0.0005917
rs143781480	23	81811216	G	0	10	A	0.0005917
rs144543301	23	81811869	C	0	10	G	0.0005917
rs143387505	23	83620472	T	22	4	C	0.0002148
rs140538401	23	83620619	T	22	4	C	0.0002148
rs147071035	23	83621520	T	22	4	C	0.0002148
rs143856395	23	83621627	A	22	4	T	0.0002148
rs146273249	23	83621636	T	22	4	A	0.0002148
rs138146847	23	83622161	GA	22	4	G	0.0002148
rs145974824	23	83622873	G	22	4	A	0.0002148
rs151153278	23	83623294	A	22	4	G	0.0002148
rs3788956	23	83625961	C	22	4	T	0.0002148
rs148411370	23	83626440	A	22	4	G	0.0002148
rs2334214	23	83628018	C	22	4	T	0.0002148
rs192217168	23	83628924	G	22	4	A	0.0002148
rs183591666	23	83629498	C	22	4	T	0.0002148
rs186945596	23	83629499	A	22	4	G	0.0002148
rs143850825	23	83630607	A	22	4	G	0.0002148
rs2092545	23	83631474	G	22	4	A	0.0002148
rs141703939	23	83633235	A	22	4	G	0.0002148
rs138684265	23	83633447	T	22	4	C	0.0002148
rs147330459	23	83634030	T	22	4	C	0.0002148
rs149405319	23	83634197	C	22	4	T	0.0002148
rs4828256	23	83636936	A	22	4	C	0.0002148
rs187422740	23	83641693	A	22	4	T	0.0002148
rs191711942	23	83641694	T	22	4	C	0.0002148
rs56997878	23	83642440	T	22	4	G	0.0002148
rs55719627	23	83643190	T	20	4	G	0.000711

rs17219420	23	83658710	C	22	4	T	0.0002148
rs57819529	23	83661110	T	22	4	C	0.0002148
rs138342358	23	83662421	G	22	4	A	0.0002148
rs4828257	23	83663001	G	22	4	C	0.0002148
rs4828258	23	83663036	T	22	4	C	0.0002148
rs4828259	23	83663206	A	22	4	G	0.0002148
rs4828261	23	83663795	A	22	4	T	0.0002148
rs145493356	23	83664038	A	22	4	G	0.0002148
rs199688599	23	83664225	G	22	4	T	0.0002148
rs187724536	23	83664363	T	22	4	C	0.0002148
rs140410819	23	83664833	G	22	4	A	0.0001968
rs201145449	23	83664893	T	20	3	TA	0.0002129
rs7472132	23	83665880	A	22	4	T	0.0002392
rs201940237	23	83665966	GTGT	22	4	G	0.0002148
rs4828262	23	83666425	A	22	4	G	0.0002148
rs145723094	23	83667629	G	22	4	C	0.0002148
rs141008163	23	83668371	T	22	4	G	0.0002148
rs56804468	23	83669036	A	22	4	T	0.0002148
rs60588322	23	83669563	A	22	4	G	0.0002148
rs10521367	23	83669700	G	22	4	A	0.0002148
rs146522984	23	83670042	T	22	4	A	0.0002148
rs150161974	23	83670753	T	22	4	C	0.0002148
rs144733150	23	83672110	C	22	4	T	0.0002148
rs140023556	23	83673499	T	22	4	C	0.0001968
rs140301368	23	83674126	C	22	4	T	0.0002148
rs145847877	23	83674275	A	22	4	G	0.0002148
rs182866637	23	83674562	G	22	4	C	0.0002148
rs4828266	23	83674724	G	22	4	A	0.0002148
rs4828267	23	83674832	C	22	4	G	0.0002148
rs148364124	23	83675747	A	22	4	C	0.0002148
rs55943150	23	83676556	C	22	4	CAA	0.0002148
rs56251925	23	83676699	A	22	4	G	0.0002102
rs55940001	23	83677967	G	22	4	T	0.0002148
rs56168822	23	83678021	T	20	4	A	0.0003367
rs59392857	23	83678649	G	22	4	A	0.0002148
rs61469178	23	83678786	T	22	4	C	0.0002148
rs61710472	23	83678935	C	22	4	T	0.0002148
rs141423363	23	83679079	T	22	4	G	0.0002148
rs139241696	23	83679323	A	22	4	G	0.0002148
rs147216147	23	83679799	G	22	4	A	0.0002148

rs2858677	23	86221003	G	57	30	C	0.0005516
rs5969294	23	86983288	C	2	16	G	0.0003115
rs6521963	23	86983765	G	2	16	A	0.0003115
rs5924097	23	86984262	C	2	16	A	0.0003115
rs5924099	23	86985145	C	2	16	T	0.0003115
rs6521964	23	86987258	T	2	16	C	0.0003115
rs5969296	23	86987339	G	2	16	A	0.0003115
rs2988819	23	90200597	G	24	46	C	0.0006257
rs6522514	23	91548348	G	14	1	A	0.0006103
rs6522516	23	91548566	G	15	1	A	0.000299
rs5984170	23	91557924	G	15	1	A	0.0003131
rs5949292	23	94634026	C	16	2	T	0.0007845
rs12844369	23	96255401	T	0	10	C	0.0005917
rs7050867	23	100686677	A	32	12	G	0.0008741
rs7052093	23	100686721	T	32	12	C	0.0008741
rs5951263	23	100704131	C	28	8	T	0.0006347
rs139562096	23	101097636	C	18	3	G	0.0008121
rs77412011	23	102522169	A	49	25	AG	0.00085
rs2983089	23	102535469	C	48	24	T	0.0007984
rs2983090	23	102537062	A	50	25	T	0.000499
rs60128102	23	102537096	A	50	26	AT	0.0008986
rs2858109	23	102537485	T	49	25	C	0.00085
rs2858108	23	102537799	T	49	25	C	0.00085
rs3015329	23	102538313	A	49	25	C	0.00085
rs146346206	23	102539527	G	49	25	C	0.00085
rs4907884	23	102540254	A	49	25	G	0.00085
rs139345449	23	102541151	A	18	2	G	0.000203
rs2474244	23	102543595	G	49	25	A	0.0004802
rs2756052	23	102544296	A	49	25	G	0.00085
rs2756054	23	102546270	A	50	25	G	0.000499
rs2858106	23	102550213	C	49	25	T	0.00085
rs34815154	23	102552032	A	49	25	AT	0.00085
rs5945971	23	102583405	T	52	28	C	0.0009847
rs6621664	23	102678067	C	48	23	T	0.0004351
rs210434	23	104949928	G	4	19	A	0.0005969
rs210439	23	104959059	G	4	19	A	0.0005969
rs3038940	23	104963739	TAA	2	15	T	0.000613
rs73636261	23	104964027	C	4	19	T	0.0005969
rs7891511	23	104973602	G	4	19	T	0.0005969
rs5962575	23	104981504	C	4	19	T	0.0005969

rs200458290	23	104983337	GATTT	4	19	G	0.0005969
rs10126447	23	104988706	G	4	19	A	0.0005969
rs10218388	23	104988949	C	4	19	T	0.0005969
rs3764765	23	104992963	C	4	19	T	0.0005969
rs5962298	23	104993513	G	4	19	A	0.0005969
rs5962579	23	104994182	T	4	19	A	0.0005969
rs201435006	23	105050984	GT	1	14	G	0.0005996
rs78553510	23	105268812	C	1	14	CT	0.0002759
rs5916754	23	105455775	T	24	6	C	0.0005592
rs150724424	23	109752418	A	11	0	G	0.0007274
rs141911700	23	109770433	G	12	0	A	0.0003422
X:109846096	23	109846096	T	12	0	C	0.0003422
rs149294490	23	109887848	G	12	0	A	0.0003422
rs141590782	23	110212107	A	11	0	G	0.0007274
rs16986748	23	111286093	G	1	12	A	0.0009301
rs7054642	23	111289482	T	1	12	C	0.0009301
rs7878779	23	111292662	T	1	14	C	0.0002433
rs60744336	23	111311333	G	0	10	A	0.0005917
rs16986963	23	111647953	A	11	0	G	0.0007274
rs5946287	23	114396657	A	2	15	T	0.0006243
rs59067332	23	114723192	C	37	15	T	0.0009053
rs73577794	23	114724262	T	37	11	G	0.00007658
rs73577800	23	114725164	A	37	15	G	0.0009053
rs2895154	23	114725960	G	36	13	T	0.0003885
rs988456	23	115659164	A	56	29	G	0.0001959
rs139721366	23	115661289	T	56	29	G	0.0001959
rs1013220	23	115662445	T	56	29	C	0.0001959
rs5905338	23	115663534	T	56	29	C	0.0001959
rs5905342	23	115664640	G	56	29	A	0.0001959
rs6651577	23	115665691	G	56	29	A	0.0001959
rs5905343	23	115666144	C	56	29	T	0.0001959
rs5905211	23	115666246	T	56	29	C	0.0001959
rs5905346	23	115667761	T	56	29	C	0.0001959
rs6608698	23	115668322	C	56	29	T	0.0001959
rs6608699	23	115669288	G	56	29	A	0.0001959
rs4145420	23	115670754	G	56	29	A	0.0001959
rs6608527	23	115670779	C	56	29	G	0.0001959
rs5905347	23	115671758	T	56	28	C	0.0001045
X:115672688	23	115672688	T	56	29	TTC	0.0001959
X:115672692	23	115672692	CAAATTAGAG	56	29	C	0.0001959

X:115672695	23	115672695	TGACAAATTGACA	56	29	T	0.0001959
rs2187881	23	115673376	T	56	29	A	0.0001959
rs2157427	23	115673454	G	55	27	A	0.0001689
rs5905348	23	115675146	T	56	29	C	0.0001959
rs11260159	23	115734350	T	56	29	C	0.0001959
rs9329406	23	115740985	A	29	52	G	0.0002991
rs9780253	23	115741598	C	56	27	A	0.00005419
rs12390117	23	115743813	G	56	27	A	0.00005419
rs11260106	23	115744144	C	56	27	T	0.00005419
rs11260118	23	115744897	C	56	27	T	0.00005419
rs11260121	23	115745642	C	56	27	T	0.00005419
rs6655546	23	115747311	T	56	27	C	0.00005419
rs11260135	23	115747885	G	56	27	A	0.00005419
rs112534898	23	115747924	G	56	27	GAT	0.00005419
rs12834189	23	115748233	A	56	27	G	0.00005419
rs12837027	23	115748364	C	56	27	T	0.00005419
rs200565252	23	115749377	G	56	27	GA	0.00005419
rs11260148	23	115749542	C	56	27	A	0.00005419
rs11260149	23	115749593	A	56	27	C	0.00005419
rs11260150	23	115749818	G	56	27	A	0.00005419
rs11260151	23	115749920	A	56	27	G	0.00005419
rs12398681	23	115750080	C	56	27	T	0.00005419
rs12398711	23	115750294	G	56	27	T	0.00005419
rs28441538	23	115750497	A	32	57	G	0.00009501
rs66834529	23	115751442	C	56	27	T	0.00005419
rs73226865	23	115751478	A	56	27	T	0.00005419
rs11260156	23	115752193	A	56	27	T	0.00005419
rs12398615	23	115752926	T	56	27	C	0.00005419
rs10907033	23	115753053	G	56	27	A	0.00005419
rs10907034	23	115753283	G	56	27	C	0.00005419
rs10907035	23	115753330	A	56	27	G	0.00005419
rs10796347	23	115753621	C	56	27	T	0.00005419
rs11260172	23	115755324	C	58	28	T	0.00005205
rs12400026	23	115755656	A	34	56	T	0.0005669
rs12557454	23	115756177	T	34	56	C	0.0005669
rs74574712	23	115756248	TA	34	56	T	0.0005669
rs73213286	23	115756372	C	34	56	A	0.0005669
rs12398361	23	115757430	G	23	44	A	0.0006657
rs6603394	23	115757476	G	45	21	A	0.0006315
rs12400045	23	115758626	A	23	44	G	0.0006657

rs7059336	23	115762843	T	56	27	C	0.00005419
rs7049926	23	115762854	A	56	27	G	0.00005419
rs12835373	23	115764107	C	23	44	T	0.0006657
rs12853107	23	115764937	C	58	28	T	0.00002988
rs7473283	23	115766722	A	16	35	T	0.000956
rs10658209	23	115769642	G	56	27	GAT	0.00005419
rs7473627	23	115774190	T	15	35	C	0.0004795
rs7391753	23	115774601	T	58	28	C	0.00002988
rs12398148	23	115778720	A	15	35	C	0.0004795
rs12847918	23	115780825	A	15	35	G	0.0004795
rs12853855	23	115780844	T	15	35	C	0.0004795
rs12559419	23	115781639	C	26	51	T	0.00008049
rs12395694	23	115781926	C	27	51	T	0.0001548
rs199692781	23	115782112	T	28	52	TAATA	0.0001612
rs62605741	23	115782964	C	27	52	T	0.00008446
rs12398468	23	115784630	T	26	51	C	0.00008049
rs11260345	23	115786229	G	28	51	T	0.0002892
rs12395202	23	115786761	T	56	27	C	0.00005419
rs5910167	23	115787601	T	27	51	G	0.0001548
rs5910166	23	115787761	A	26	51	G	0.00008049
rs5912057	23	115788011	G	26	51	C	0.00008049
rs34307386	23	115788942	A	27	51	G	0.0001548
rs5912030	23	115792853	A	58	28	G	0.00002988
rs5912025	23	115794587	A	45	21	G	0.0006315
rs7057172	23	115797756	A	60	28	G	0.000007795
rs1506960	23	115798129	T	60	28	C	0.000007795
rs5956740	23	115799907	C	60	28	G	0.000007795
rs1506958	23	115803356	C	22	44	T	0.0003557
rs5903481	23	115803731	T	47	21	TTATC	0.000209
rs113858028	23	115806047	A	47	21	ATGT	0.000209
rs5911731	23	115807323	T	60	28	C	0.000007795
rs1395554	23	115808095	T	22	44	G	0.0003557
rs11798535	23	115808182	C	22	44	G	0.0003557
rs1354799	23	115808840	T	22	44	G	0.0003557
rs12395259	23	115810023	G	22	44	A	0.0003557
rs7473168	23	115810825	C	22	44	T	0.0003557
rs7473986	23	115811177	C	22	44	T	0.0003557
rs28581957	23	115811962	G	22	44	C	0.0003557
rs2335018	23	115812234	C	22	44	T	0.0003557
rs3965555	23	115812261	C	22	44	A	0.0003557

rs369242479	23	115813360	T	1	13	C	0.0005677
rs34339026	23	115816699	C	28	56	CT	0.000007955
rs5909877	23	115825960	G	49	23	A	0.0002495
rs202023623	23	115826705	GT	4	20	G	0.0003114
rs1876296	23	115827591	C	49	23	G	0.0002495
rs1876297	23	115827794	A	3	19	G	0.0001662
rs147251870	23	115984992	C	14	1	T	0.0006677
rs137995500	23	116090158	C	14	1	T	0.0006347
X:116415994	23	116415994	C	21	4	T	0.000448
rs1230835	23	117318970	A	10	30	G	0.0002669
rs57114393	23	117319283	A	10	28	AGTGGGGAAAAAAAGAAAAT	0.0008979
rs2682987	23	117322365	G	10	28	A	0.000853
rs2683008	23	117324955	C	10	28	A	0.000853
rs139144471	23	117829694	G	17	2	T	0.0004014
rs4825655	23	118721419	G	24	45	A	0.0007128
rs139282281	23	118758234	A	11	0	G	0.0007086
rs6603602	23	119338274	C	42	19	G	0.0008809
rs5909724	23	119619770	C	18	3	G	0.0008121
rs11314157	23	119917765	T	43	20	TA	0.0009741
rs5909763	23	119918392	G	43	20	A	0.0009741
rs5957482	23	119918735	G	43	20	T	0.0009741
rs5909764	23	119919126	A	43	20	C	0.0009741
rs5956304	23	120465431	A	20	4	G	0.0007606
rs10482505	23	120466128	C	20	4	A	0.0007606
rs6648591	23	120469471	C	20	4	T	0.0007606
rs6649201	23	120471336	G	22	4	T	0.0002148
rs6648601	23	120476218	A	20	4	G	0.0007606
rs5957660	23	120477020	A	20	4	G	0.0007606
rs10429756	23	120478777	G	20	4	A	0.0007606
rs5956305	23	120479248	C	20	4	G	0.0007606
rs6648605	23	120482339	G	20	4	C	0.0007606
rs6648606	23	120482340	T	20	4	G	0.0007606
rs73631065	23	120486850	A	20	4	G	0.0007606
rs6648611	23	120489819	C	20	4	T	0.000828
rs73631088	23	120492945	G	20	4	C	0.0007606
rs12687916	23	120493218	T	20	4	G	0.000828
rs5957689	23	120494229	G	20	4	A	0.0007606
rs5956318	23	120497263	C	20	4	G	0.0007606
rs12396010	23	120502315	T	20	4	C	0.0007606
rs1351454	23	121521935	A	20	40	G	0.0008835

rs7884179	23	121522338	T	20	40	C	0.0008835
rs5911308	23	121523310	C	20	40	A	0.0008835
rs5958012	23	121524744	A	18	40	G	0.0003958
rs58439862	23	121526326	G	20	40	GA	0.0008835
rs1585954	23	121528162	A	20	40	G	0.0008835
rs202026185	23	121529094	C	20	40	T	0.000791
rs1988741	23	121532885	A	20	40	G	0.0008835
rs62594887	23	121533258	G	20	40	T	0.0008835
rs11796035	23	121534107	A	20	40	C	0.0008835
rs1481179	23	121536593	A	20	40	G	0.0008835
rs5911314	23	121537051	C	20	40	T	0.0008835
rs13440620	23	121538717	T	20	40	C	0.0008835
rs5911315	23	121539373	T	20	40	A	0.0008835
rs1351453	23	121540546	T	20	40	G	0.0008835
rs2199957	23	121546704	C	20	41	T	0.0005156
rs11260371	23	121546991	C	20	41	T	0.0005156
rs2840663	23	121562938	G	15	40	A	0.00004144
rs4825452	23	121565186	G	18	41	T	0.0001308
rs3761557	23	122315469	T	15	35	C	0.0004795
rs34786936	23	122573644	C	3	17	CT	0.0006979
rs199426	23	123131239	G	19	3	A	0.0004265
rs112913630	23	123192419	T	20	3	A	0.0002211
rs35941521	23	123208682	C	22	3	T	0.00005724
rs1279794	23	123324050	T	6	22	C	0.0007921
rs58030679	23	123325170	CACACACAT	6	22	C	0.0007921
rs76652059	23	123325634	C	6	22	CT	0.0007921
rs1279796	23	123326007	C	6	22	G	0.0007921
rs72155652	23	123326867	G	6	22	GCC	0.0007921
rs72059681	23	123326870	T	6	22	TCCGCA	0.0007921
rs1279799	23	123327156	T	6	22	G	0.0007921
rs1279800	23	123327486	T	6	22	C	0.0007921
rs1279801	23	123327934	G	6	22	A	0.0007921
rs1279802	23	123327963	C	6	22	T	0.0007921
rs1279803	23	123328193	T	6	22	C	0.0007921
rs988930	23	123328406	A	6	22	G	0.0007921
rs79001586	23	123329157	CT	6	22	C	0.0007921
rs1279807	23	123329635	T	6	22	C	0.0007921
rs1279808	23	123329909	G	6	22	A	0.0007921
rs1279810	23	123332593	C	6	22	A	0.0007921
rs3126134	23	124547784	C	17	37	T	0.0006349

rs35336134	23	124549116	AT	18	38	A	0.0007166
rs3126133	23	124552884	C	18	38	A	0.0007166
rs3101167	23	124556706	G	18	38	A	0.0007166
rs3126129	23	124561259	A	17	38	G	0.0003648
rs3101137	23	124572254	A	13	32	G	0.0009401
rs3101139	23	124573867	A	13	32	G	0.0009401
rs3126296	23	124574101	G	13	32	A	0.0009401
rs3126297	23	124574107	G	13	32	A	0.0009401
rs1903090	23	124574330	G	13	32	A	0.0009401
rs1903091	23	124574487	T	13	32	C	0.0009401
rs1903092	23	124574488	G	13	32	A	0.0009401
rs3126302	23	124577099	C	13	32	T	0.0009401
rs35532723	23	124577621	TAC	13	32	T	0.0009401
rs5930020	23	124577901	C	11	32	T	0.000192
rs61536326	23	127751810	T	21	41	C	0.0009669
rs2027843	23	127758031	T	21	45	C	0.0001015
rs648	23	127764381	T	21	45	C	0.0001015
rs5932468	23	127765609	C	21	45	T	0.0001015
rs5930270	23	127776026	A	21	45	G	0.0001015
rs5930271	23	127777221	A	21	45	G	0.0001015
rs10528606	23	127778933	A	26	49	AAAACAAAC	0.0004462
rs148044293	23	132414633	A	43	15	G	0.00002703
rs2073051	23	132473645	A	12	31	G	0.0007705
rs139859148	23	133515780	T	24	5	C	0.0001934
rs2807261	23	135661799	C	24	5	T	0.0001934
rs2518882	23	135662054	A	24	5	G	0.0001934
rs2807263	23	135663181	C	24	5	G	0.0001934
rs2807264	23	135665778	C	24	5	A	0.0001934
rs2807266	23	135666415	T	24	5	G	0.0001934
rs2807267	23	135666453	T	24	5	G	0.0001934
rs34891204	23	135666576	TC	24	5	T	0.0001748
rs2807257	23	135666848	A	24	6	G	0.0005592
rs2518883	23	135666923	T	24	5	C	0.0001934
rs2807258	23	135668903	A	24	5	G	0.0001934
rs2518885	23	135668988	A	24	5	C	0.0001934
rs2518886	23	135670593	T	24	5	C	0.0001934
rs12852548	23	135670824	A	24	5	T	0.0001934
rs2807259	23	135671221	A	24	5	G	0.0001934
rs2807260	23	135674044	A	25	5	C	0.00009108
rs2518888	23	135674388	C	25	5	T	0.000102

rs2518889	23	135674543	A	25	5	T	0.0001169
rs2518891	23	135675755	C	25	5	T	0.000102
rs144807293	23	135676651	CACTT	25	5	C	0.000102
rs2518892	23	135676896	T	25	5	C	0.000102
rs2518893	23	135677559	T	25	5	A	0.000102
rs2518894	23	135677710	C	25	5	T	0.000102
rs2518895	23	135678840	A	25	5	G	0.000102
rs2518896	23	135679053	A	25	5	G	0.0001169
rs2518897	23	135679059	A	25	5	G	0.0001169
rs2518899	23	135679631	A	25	5	G	0.000102
rs2518900	23	135679960	G	25	5	C	0.000102
rs2518901	23	135680078	T	25	5	G	0.000102
rs2518902	23	135680774	G	25	5	C	0.000102
rs12556398	23	135680970	A	25	5	G	0.0001169
rs2518903	23	135681110	C	25	5	T	0.000102
rs2518904	23	135681823	A	23	5	G	0.0003539
rs12007112	23	135682887	C	26	7	T	0.0004518
rs12012220	23	135683355	T	25	5	C	0.000102
rs12012314	23	135683500	A	25	5	G	0.000102
rs73228703	23	135683508	G	25	5	T	0.000102
rs12848318	23	135684162	A	25	5	C	0.000102
rs12559890	23	135685169	A	25	5	C	0.000102
rs12014670	23	135685563	A	25	5	C	0.000102
rs12556784	23	135687009	G	26	7	A	0.0004518
rs12559116	23	135687540	C	26	7	T	0.0004518
rs3043322	23	135689151	A	26	7	ATTTTG	0.0004518
rs518667	23	138077510	C	0	15	T	0.0000112
rs78974273	23	138079591	A	0	15	AC	0.0000112
rs579260	23	138086137	T	0	15	C	0.0000112
rs471635	23	138086434	A	0	15	C	0.00001334
rs500096	23	138087155	G	0	15	C	0.0000112
rs565127	23	138088701	C	0	15	T	0.0000112
rs5931554	23	138091869	C	2	15	T	0.0006952
rs472273	23	138097159	G	0	15	C	0.0000112
rs2179589	23	138097310	G	0	15	A	0.0000112
rs566187	23	138098674	A	0	15	T	0.0000112
rs496732	23	138099648	T	0	15	C	0.000009157
rs490787	23	138100179	G	0	15	A	0.0000112
rs512809	23	138101612	C	0	15	A	0.0000112
rs471600	23	138101814	G	0	15	A	0.0000112

rs482328	23	138102649	C	0	15	T	0.0000112
rs483419	23	138105978	C	0	15	T	0.0000112
rs497635	23	138106122	T	0	15	G	0.0000112
rs578264	23	138107101	C	0	15	T	0.0000112
rs515444	23	138107138	G	0	15	A	0.0000112
rs519841	23	138108803	C	0	15	T	0.0000112
rs149173046	23	138110078	C	0	15	CAAGA	0.0000112
rs693310	23	138110176	C	0	15	T	0.0000112
rs5931560	23	138110307	A	0	15	G	0.0000112
rs526093	23	138111126	G	0	15	A	0.0000112
rs477821	23	138111932	A	0	15	G	0.0000112
rs6635762	23	138113107	A	0	15	G	0.0000112
rs67703366	23	138113374	CA	0	15	C	0.0000112
rs79440397	23	138113382	A	0	15	T	0.0000112
rs6634041	23	138113909	C	0	13	A	0.00004715
rs17001950	23	138114091	G	0	15	C	0.0000112
rs60643249	23	138114217	A	0	15	G	0.0000112
rs62602197	23	138114331	A	0	15	G	0.0000112
rs61245857	23	138114383	G	0	15	A	0.0000112
rs6634042	23	138114720	C	0	15	A	0.0000112
rs7884030	23	138115512	C	0	15	T	0.0000112
rs7883424	23	138115569	A	0	15	G	0.0000112
rs17001957	23	138116229	C	0	15	A	0.0000112
rs62602201	23	138116536	A	0	15	G	0.0000112
rs62602202	23	138117015	C	0	15	T	0.0000112
rs16993529	23	138117256	T	0	15	C	0.0000112
rs10521792	23	138117409	G	0	15	A	0.0000112
rs17001960	23	138119216	A	0	15	T	0.0000112
rs62602203	23	138119644	C	0	15	A	0.0000112
rs62602204	23	138119702	A	0	15	T	0.0000112
rs112015472	23	138119748	A	0	15	T	0.0000112
rs7889036	23	138119874	T	0	15	A	0.0000112
rs62602205	23	138120458	A	0	15	G	0.0000112
rs12558011	23	138120702	T	0	15	A	0.0000112
rs12556537	23	138120938	C	0	15	T	0.0000112
rs17001963	23	138122394	T	0	15	G	0.0000112
rs62602208	23	138126601	C	0	15	A	0.0000112
rs62602209	23	138127036	G	0	15	A	0.0000112
rs2103610	23	138127481	A	0	15	G	0.0000112
rs112592286	23	138128310	C	0	15	CCTCAGGAAG	0.0000112

rs62602236	23	138128358	A	0	15	G	0.0000112
rs12560172	23	138130418	C	0	15	T	0.0000112
rs62602238	23	138130921	C	0	15	T	0.0000112
rs62602239	23	138133918	G	0	15	T	0.0000112
rs62602240	23	138134536	G	0	15	A	0.0000112
rs62602241	23	138134795	T	0	15	C	0.0000112
rs1080166	23	138137774	C	0	15	T	0.0000112
rs62602242	23	138140150	C	0	15	T	0.0000112
rs7877464	23	138140408	G	0	15	A	0.0000112
rs12556549	23	138140736	T	0	15	G	0.0000112
rs62602243	23	138142110	A	0	15	G	0.0000112
rs374366794	23	138143770	A	0	15	AT	0.0000112
rs113373639	23	138144111	C	0	15	CTTA	0.0000112
rs143453100	23	138147525	C	0	12	T	0.0001058
rs62602246	23	138148857	A	0	14	G	0.00002513
rs62602247	23	138150491	C	0	10	T	0.0004558
rs138951453	23	138152160	C	0	15	T	0.0000112
rs141403534	23	138152804	A	0	14	G	0.00002513
rs147006243	23	138152877	T	0	14	C	0.00002513
rs112600886	23	138154239	A	0	15	G	0.0000112
rs112886945	23	138155295	T	0	14	TC	0.00002513
rs201924524	23	138155299	G	0	14	C	0.00002513
rs10521793	23	138156505	T	0	14	C	0.00002513
rs17284663	23	138160456	T	0	14	A	0.00002513
rs17331408	23	138160576	C	0	14	T	0.00002513
rs112249167	23	138161557	A	0	14	AC	0.00002513
rs62603179	23	138166155	G	0	15	A	0.0000112
rs62603180	23	138177022	T	0	15	C	0.0000112
rs7890667	23	138178531	A	0	14	G	0.00002513
rs62603183	23	138179958	G	0	14	C	0.00002513
rs111506647	23	138181040	C	0	12	T	0.0001058
rs7884308	23	138182426	A	0	14	G	0.00002513
rs7888266	23	138182784	T	0	14	G	0.00002513
rs62603185	23	138183067	G	0	15	A	0.00001334
rs2206125	23	138185215	T	0	14	C	0.00002513
rs111519164	23	138185531	A	0	14	G	0.00002513
rs17001986	23	138186360	C	0	12	G	0.0001058
rs62603187	23	138186858	T	0	14	C	0.00002513
rs199795206	23	138187174	GT	0	11	G	0.0002032
rs17331422	23	138187934	G	0	15	A	0.0000112

rs979128	23	138188114	C	0	15	A	0.0000112
rs41300307	23	138188496	A	0	14	G	0.00002513
rs201138057	23	138189171	C	0	11	CT	0.0002715
rs200196785	23	138189174	A	0	11	T	0.0002715
rs17284670	23	138198382	C	0	12	T	0.0001237
rs5976277	23	138201343	G	0	11	A	0.0002715
rs5976278	23	138201602	G	0	12	A	0.0001237
rs62603211	23	138203325	G	0	10	C	0.0005917
rs7884407	23	138203555	G	0	10	T	0.0005917
rs62603212	23	138203581	T	0	10	A	0.0005917
rs2227059	23	138236904	C	26	47	T	0.0007993
rs74657155	23	139874134	TC	0	11	T	0.0002715
rs78879113	23	139875692	TTCTC	0	11	T	0.0002715
rs55877737	23	139876177	CT	0	11	C	0.0002715
rs1006861	23	141304473	T	12	33	G	0.0002456
rs1883397	23	141308325	C	12	35	T	0.00007325
rs62600825	23	141963178	T	30	10	C	0.0006347
rs7878290	23	142421475	C	9	27	T	0.000654
rs7881609	23	142422021	A	9	27	G	0.000654
rs57173964	23	142426750	G	9	27	A	0.000654
rs200143228	23	142427183	T	9	27	TTCC	0.000654
rs7889524	23	142429074	T	9	27	C	0.000654
rs67474692	23	142430116	A	9	27	G	0.000654
rs199822404	23	142430337	A	9	27	AT	0.000654
rs67893966	23	142430401	G	9	27	A	0.000654
rs74769907	23	143200302	C	43	17	T	0.00006761
rs77429583	23	143200330	G	43	17	T	0.000129
rs77311884	23	144119425	C	12	0	T	0.0003422
rs62598640	23	144120361	G	12	0	A	0.0003422
rs143406498	23	144120430	C	12	0	A	0.0003422
rs62598643	23	144121141	G	12	0	C	0.0003422
rs142573764	23	144121641	G	12	0	T	0.0003422
rs148321100	23	144122981	T	12	0	C	0.0003422
rs62598644	23	144123298	T	12	0	C	0.0003422
rs201153231	23	144124658	A	12	0	AAGAGAG	0.0003422
rs149379606	23	144125365	T	12	0	C	0.0003422
X:144125922	23	144125922	C	12	0	G	0.0003422
X:144126802	23	144126802	G	12	0	A	0.0003422
rs62600167	23	144127770	T	12	0	C	0.0003422
rs62600169	23	144129067	G	12	0	A	0.0003422

rs7064720	23	144129334	T	12	0	G	0.0003422
rs62600170	23	144130622	G	12	0	A	0.0003422
rs62600171	23	144133012	C	12	0	T	0.0003422
rs58284834	23	144133144	A	12	0	G	0.0003422
rs202110064	23	144136355	G	12	0	GTGTA	0.0003315
rs202055875	23	144144189	C	12	0	CAT	0.0003422
rs200055114	23	144145183	A	12	0	AAAAT	0.0003422
rs62600181	23	144145918	C	12	0	T	0.0003422
rs62600183	23	144146627	A	12	0	G	0.0003422
rs62600218	23	144171602	A	11	0	T	0.0007274
rs5920184	23	145591751	C	38	15	T	0.0005267
rs5920186	23	145592503	T	38	14	C	0.0002538
rs5920190	23	145599038	C	38	15	T	0.0005267
rs12690080	23	145605081	A	50	24	G	0.0002688
rs6626130	23	145606916	A	45	20	G	0.000332
rs12013368	23	145607302	C	47	22	T	0.0004013
rs5920197	23	145607800	G	45	20	A	0.000332
rs4827722	23	145608214	G	47	21	A	0.000209
rs4827723	23	145608311	C	47	21	T	0.000209
rs11439097	23	145608414	G	47	21	GA	0.000209
rs6626622	23	145608730	T	45	20	C	0.000332
rs139717812	23	145611501	GT	51	24	G	0.0001515
rs7064347	23	145611502	G	51	24	T	0.0001515
rs62607009	23	145611503	T	51	24	G	0.0001515
rs12847976	23	145613433	T	50	22	G	0.00002954
rs6525699	23	145621372	C	45	19	G	0.0001682
rs5920210	23	145623452	A	45	19	G	0.0001682
rs5920215	23	145627146	G	46	21	A	0.0003668
rs12556994	23	145627150	G	47	22	T	0.0004013
rs5920216	23	145627182	G	45	21	A	0.0006315
rs5966251	23	145627597	G	47	22	A	0.0004013
rs2207939	23	145628200	A	47	22	G	0.0004013
rs2207941	23	145628442	G	51	22	A	0.00003821
rs4827730	23	145628722	A	47	22	T	0.0004013
rs1543431	23	145629374	G	47	22	A	0.0004013
rs2207942	23	145630047	C	27	7	T	0.0002486
rs10521858	23	145630262	A	27	7	C	0.0002486
X:145630523	23	145630523	C	26	7	CCCACTT	0.0004518
X:145630534	23	145630534	G	26	7	C	0.0004518
X:145630535	23	145630535	T	26	7	C	0.0004518

X:145630536	23	145630536	G	26	7	C	0.0004518
X:145630539	23	145630539	T	26	7	A	0.0004518
rs202182192	23	145630542	T	26	7	TAG	0.0004518
rs12689781	23	145630720	T	27	7	C	0.0002486
rs5920217	23	145630797	C	54	27	G	0.000181
rs2385657	23	145631205	A	27	7	G	0.0002486
rs5920218	23	145631282	A	32	53	G	0.0009656
rs5920219	23	145631330	G	27	7	C	0.0002486
rs2224194	23	145633069	T	27	7	C	0.0002486
rs5966255	23	145634017	T	27	6	C	0.0000877
rs5920220	23	145637758	T	26	6	G	0.0001649
rs6626138	23	145641261	A	50	24	G	0.0002688
rs5966261	23	145641460	G	27	7	A	0.0002486
rs5965765	23	145641513	A	51	24	G	0.0001515
rs5920221	23	145642309	G	51	24	A	0.0001515
rs5920223	23	145643622	C	51	24	T	0.0001515
rs5920224	23	145643688	C	51	24	T	0.0001515
rs5920225	23	145643826	C	51	24	G	0.0001515
rs5920226	23	145643916	C	51	24	T	0.0001515
rs5920227	23	145644014	G	27	7	A	0.0002486
rs5965766	23	145644397	C	51	24	A	0.0001515
rs1998037	23	145645011	C	51	24	A	0.0001515
rs12688276	23	145645486	T	26	6	G	0.0001649
rs5920228	23	145646314	G	27	6	A	0.0000877
rs1157066	23	145646510	G	51	24	A	0.0001515
rs5920229	23	145646619	G	51	24	T	0.0001515
rs4827731	23	145646863	C	51	24	A	0.0001515
rs4827732	23	145646889	G	51	24	A	0.0001515
rs4827733	23	145646998	C	51	24	T	0.0001515
rs4827617	23	145647131	G	51	24	A	0.0001515
rs4827618	23	145647421	G	50	24	A	0.0002688
rs6626142	23	145647815	G	26	7	T	0.0004518
rs6626143	23	145647916	G	51	24	A	0.0001515
rs6626652	23	145648192	C	51	24	A	0.0001515
rs7061576	23	145649065	T	27	7	A	0.0002486
rs1033646	23	145649246	C	27	7	T	0.0002486
rs1033647	23	145649326	A	27	7	G	0.0002486
rs1033648	23	145649595	G	27	7	T	0.0002486
rs5919714	23	145649944	G	27	7	A	0.0002486
rs1033649	23	145650329	C	27	7	T	0.0002486

rs1033650	23	145650468	A	51	24	G	0.0001515
rs1033651	23	145650554	C	27	7	A	0.0002486
rs1033652	23	145650719	A	51	22	G	0.00003821
rs57898538	23	145650780	CTG	51	24	C	0.0001515
rs5920231	23	145651231	G	26	6	A	0.0001649
rs5966265	23	145653988	A	24	6	G	0.0005592
rs62607028	23	145658228	A	52	28	C	0.0009847
rs5966266	23	145658240	T	26	6	A	0.0001649
rs1116419	23	145659829	C	27	6	T	0.0000877
rs1118070	23	145661708	A	27	6	C	0.0000877
rs369026644	23	145661896	G	26	6	GT	0.0001649
rs6525706	23	145664570	A	51	22	T	0.00003821
rs73240086	23	145666370	A	28	6	T	0.00004599
rs368541187	23	145668384	T	27	6	TTATTGA	0.0000877
rs79144050	23	145668395	G	27	6	A	0.0000877
rs34264217	23	145670465	G	27	6	T	0.0000877
rs200026091	23	145670540	A	49	20	AAC	0.00003084
rs12853269	23	145672354	C	38	8	T	0.0000009115
rs2207930	23	145672472	T	55	30	C	0.0006201
rs12855563	23	145672954	G	39	8	T	0.0000002313
rs12855872	23	145672989	C	39	8	T	0.0000002313
rs61548656	23	145673230	C	50	19	G	0.000007343
rs5920237	23	145674023	A	28	5	G	0.0000138
rs60071470	23	145674985	A	28	6	ATG	0.00009139
rs6626660	23	145680046	A	28	5	G	0.00001183
rs5919717	23	145681309	A	27	5	C	0.00002218
rs5920245	23	145690788	C	46	19	T	0.00009334
rs11094365	23	145691236	G	46	19	A	0.00009334
rs5965773	23	145692932	C	45	19	T	0.0001682
rs6626148	23	145694582	G	25	5	C	0.000102
rs959605	23	145698003	C	25	5	T	0.000102
rs4304470	23	145702711	G	46	19	T	0.00009334
rs4250358	23	145702983	A	45	19	G	0.0001682
rs4319233	23	145703297	C	25	5	G	0.00009913
rs2891884	23	145703928	G	25	5	A	0.00009108
rs7887109	23	145722579	T	33	55	C	0.0005652
rs381365	23	145748908	T	23	4	G	0.0001119
rs407585	23	145767522	T	24	4	A	0.00005751
rs445978	23	145788647	T	23	5	A	0.0003539
rs1620816	23	145796549	A	23	5	G	0.0003619

rs192213056	23	146496473	G	1	14	T	0.0002433
rs5905048	23	146496477	G	32	55	T	0.0003175
rs7889692	23	147086039	G	13	0	A	0.00016
rs199528103	23	147091621	AT	13	0	A	0.00016
rs73249440	23	147846601	A	3	18	G	0.0003291
rs1882727	23	148354706	G	50	25	A	0.000499
rs10709019	23	148354772	TA	50	25	T	0.000499
rs2056833	23	148355171	G	50	25	A	0.000499
rs741733	23	148355251	C	50	25	T	0.000499
rs760051	23	148355553	C	50	25	A	0.000499
rs5936253	23	148358734	C	50	25	T	0.000499
rs5936254	23	148358760	C	50	25	T	0.000499
rs5936255	23	148360525	A	50	25	G	0.000499
rs1007084	23	148361284	A	50	25	G	0.000499
rs373447133	23	148361579	AT	50	25	A	0.000499
rs7051515	23	148362146	T	50	25	C	0.000499
rs5936256	23	148362574	T	50	25	C	0.000499
rs1980403	23	148363982	T	42	18	C	0.0004599
rs5904262	23	148364087	AG	50	25	A	0.000499
rs2205026	23	148366190	G	42	18	C	0.0004599
rs4844009	23	148369123	G	50	24	A	0.0002688
rs4844010	23	148369188	C	50	25	T	0.000499
rs5936258	23	148369607	C	50	25	T	0.000499
rs4581208	23	148370174	G	50	23	A	0.000237
rs113034212	23	148370310	CAGAGAG	38	14	C	0.0002545
rs1882728	23	148370989	A	50	25	C	0.000499
rs6641287	23	148372456	C	53	25	T	0.0000896
rs1882731	23	148406285	C	42	16	T	0.0001106
rs5936259	23	148414197	G	42	16	T	0.0001106
rs5936182	23	148470066	C	40	16	A	0.0003516
rs4133910	23	148484659	C	40	16	A	0.0003516
rs2142021	23	148487605	A	40	16	C	0.0003516
rs1576343	23	148494332	C	40	16	T	0.0003516
rs199861875	23	148518096	T	38	15	C	0.0005267
rs150553377	23	148518856	G	40	16	T	0.0003516
rs2341057	23	148525397	C	40	16	A	0.0003516
rs2142024	23	148530880	G	40	15	T	0.0001692
rs985081	23	148538099	C	40	13	T	0.0000337
rs5980471	23	148553479	A	25	7	G	0.0008092
rs5936284	23	148554165	G	29	8	A	0.0001956

rs5936285	23	148554167	T	29	8	G	0.0001956
rs5936286	23	148555007	A	29	8	G	0.0001956
rs5936186	23	148555199	G	29	8	A	0.0001956
rs1329433	23	148556303	T	29	8	C	0.0001956
rs1329432	23	148556337	A	29	8	G	0.0001956
rs5936287	23	148557940	G	29	8	C	0.0001956
rs5936288	23	148558579	A	29	8	C	0.0001956
rs5936289	23	148558580	G	29	8	T	0.0001956
rs5936290	23	148559165	T	28	8	C	0.0003544
rs5936291	23	148560611	A	27	8	G	0.0005667
rs5936188	23	148560766	C	27	8	T	0.0006326
rs11969	23	148561274	C	29	8	T	0.0001956
rs1064463	23	148562599	A	27	8	G	0.0006326
rs685678	23	148565758	C	29	8	A	0.0001956
rs10218066	23	150343248	T	26	7	G	0.0004518
X:150712685	23	150712685	G	14	1	GA	0.0006677
rs60273654	23	150838270	C	29	8	T	0.0001956
rs61186528	23	150838373	A	27	8	G	0.0006326
rs144794829	23	150849687	T	21	2	C	0.00002453
X:151164833	23	151164833	A	1	20	T	0.000002918
rs4898495	23	153659164	T	11	0	G	0.0007086
rs8425	23	153664332	G	11	0	A	0.0007274
rs146618130	23	153688369	T	11	0	C	0.0007274
X:155255914	23	155255914	C	11	0	G	0.0007274
X:155256863	23	155256863	G	11	0	C	0.0007529

Supplementary Table 2. All nominally significant (p≤0.001) genetic variants (SNVs/INDELs) for leukopenia as determined by Fisher's exact test.

rsID	Chromosome	Position	Minor allele	Minor allele frequency among cases	Minor allele frequency among controls	Major allele	p-value
rs780587	1	4505966	T	7	0	C	0.0008679
rs4990051	1	4582998	A	17	3	C	0.000003925
1:4583000	1	4583000	A	13	3	C	0.0002093
rs17030882	1	7505217	T	7	0	C	0.0008679
rs5772364	1	9351017	C	12	47	CT	0.0004795
rs9434757	1	9351785	C	11	47	G	0.0001343
rs4080311	1	9355278	A	2	23	T	0.0005167
rs563317	1	9357948	G	37	33	C	0.0006234
rs622662	1	9358085	C	37	33	G	0.0006234
rs621846	1	9358243	C	37	33	T	0.0006234
rs74052097	1	11028623	T	7	0	C	0.0008679
rs74052098	1	11028631	T	7	0	G	0.0008679
rs74052099	1	11029552	T	7	0	C	0.0008679
rs56102775	1	11029942	A	7	0	G	0.0008679
rs78234331	1	11030861	T	7	0	C	0.0008679
rs637299	1	12219861	G	38	35	C	0.0006427
rs816060	1	12219910	G	38	35	A	0.0006427
rs622839	1	12220797	C	38	34	G	0.000347
rs150885757	1	12221069	CA	31	30	C	0.0003475
rs534146	1	12221606	G	38	35	A	0.0006427
rs11122006	1	12917017	G	22	13	T	0.0006749
rs2260539	1	12922908	G	24	14	A	0.0003806
rs2743657	1	12923108	T	18	9	C	0.0009279
rs72472702	1	12923209	A	24	14	T	0.0003806
rs141354376	1	12923779	C	24	14	T	0.0003806
rs116769915	1	12925918	A	23	12	C	0.0002434
rs11811058	1	12927186	A	25	15	G	0.0002543
rs199989365	1	12931538	G	18	7	A	0.0001556
rs3895132	1	12940822	T	23	13	A	0.0003278
rs12120528	1	12941125	C	23	12	T	0.0002434
rs138280829	1	12941381	A	14	4	G	0.0003939
rs4993091	1	12941437	A	23	13	G	0.0003278
rs3121081	1	12943038	C	23	11	A	0.00008787
1:12962181	1	12962181	C	14	4	CTCTCCCTCTCCCTCTCCCTC[illegible]	0.00049
rs374538704	1	12997870	C	13	4	T	0.000883
rs2744690	1	14137801	A	13	4	C	0.000883
rs2744689	1	14137908	T	13	4	C	0.000883
rs2744688	1	14138493	G	13	4	A	0.000883
rs2744687	1	14138813	T	13	4	G	0.000883

rs34646499	1	14138827	CG	13	4	C	0.000883
rs2245213	1	14139683	A	15	4	C	0.000175
rs2245218	1	14139826	C	15	4	T	0.000175
rs2235516	1	14140457	A	14	3	G	0.00009441
rs2235514	1	14146935	G	15	4	A	0.000175
rs2697964	1	14150709	C	15	4	T	0.000175
rs2744680	1	14150714	G	15	4	A	0.000175
rs2697963	1	14150740	G	15	4	T	0.000175
rs58504586	1	14152152	T	15	4	TC	0.000175
rs2744678	1	14152217	G	15	4	A	0.000175
rs2697961	1	14153333	C	15	4	T	0.000175
rs2697960	1	14153837	A	15	4	G	0.000175
rs2744677	1	14154723	G	18	8	T	0.0005897
rs149580705	1	14154806	A	18	8	AGTAAACTTACCTGTTTCTG	0.0005897
rs2697983	1	14157577	A	18	8	G	0.0005897
rs3978481	1	16369761	A	34	30	G	0.0009121
rs34400771	1	17493815	T	8	0	C	0.0003016
rs114352248	1	23737013	T	7	0	C	0.0008679
rs76230830	1	23784293	G	7	0	A	0.0008679
rs7512844	1	23826473	T	7	0	G	0.0008679
1:23864717	1	23864717	AT	7	0	A	0.0008062
rs195738	1	24806560	C	28	19	T	0.0005259
rs200943188	1	35068893	G	9	1	GAGAGAC	0.0007194
rs6658272	1	38472841	T	20	10	C	0.0005561
rs1037976	1	38501187	T	3	26	C	0.0005074
rs7553786	1	38514453	T	21	12	C	0.0009318
rs34371200	1	38516178	T	3	26	TA	0.0005074
rs4970582	1	38516567	C	3	26	T	0.0005074
rs11205722	1	38517981	G	3	26	A	0.0005074
rs6688460	1	38522618	C	1	19	T	0.0008417
rs34125937	1	38524773	G	1	19	A	0.0008417
rs12723955	1	38527331	G	1	19	A	0.0008417
rs7537906	1	38527502	G	1	19	A	0.0008417
rs2247886	1	38931754	A	13	49	C	0.0003246
1:40190955	1	40190955	A	13	4	T	0.0007457
rs232872	1	59118959	A	9	42	G	0.0003081
rs232869	1	59119135	C	9	42	A	0.0003081
rs232861	1	59120399	T	9	42	G	0.0003081
rs232851	1	59122871	C	9	43	T	0.0001644
rs232775	1	59124546	A	9	42	G	0.0003081
rs232776	1	59124803	C	9	42	T	0.0003081

rs232781	1	59127637	T	9	42	G	0.0003081
rs232791	1	59131481	C	9	42	T	0.0003081
rs506077	1	59136429	A	9	42	C	0.0003081
rs506078	1	59136431	T	9	42	C	0.0003081
rs12082097	1	59137111	T	9	42	C	0.0003081
rs12076717	1	59137120	G	9	42	A	0.0003081
rs10489539	1	59137210	C	9	42	T	0.0003081
rs539141	1	59137783	A	9	42	G	0.0003081
rs11207286	1	59137987	G	9	42	A	0.0003081
rs2064370	1	59139223	C	9	42	G	0.0003081
rs11207287	1	59139537	G	9	42	A	0.0003081
rs2029648	1	59139725	G	9	42	A	0.0003081
rs11207288	1	59140552	T	9	43	C	0.0002957
rs11207289	1	59140553	G	9	43	A	0.0002957
rs2406931	1	59141818	C	9	42	T	0.0003081
rs554318	1	59142248	T	9	42	C	0.0003081
rs579110	1	59142713	T	9	42	C	0.0003081
rs10789070	1	59144465	C	9	42	T	0.0003081
rs527445	1	59144479	T	9	42	C	0.0003081
rs584284	1	59144787	A	9	42	G	0.0003081
rs4506484	1	59145452	A	9	42	C	0.0003081
rs4912370	1	59146186	T	10	42	C	0.0006808
rs11207292	1	59147420	G	9	41	A	0.0003404
rs12139511	1	59147926	T	9	41	C	0.0003404
rs6658186	1	59148618	C	9	41	T	0.0003404
rs6587836	1	59149561	A	10	42	C	0.0006808
rs6661709	1	59149612	C	10	42	G	0.0006808
rs6686043	1	59149664	A	9	41	T	0.0003404
rs2206765	1	59151273	G	10	42	C	0.0006808
rs2064371	1	59152015	C	9	41	T	0.0003404
rs2064372	1	59152134	T	9	41	G	0.0003404
rs12092121	1	59153010	G	9	41	A	0.0003404
rs12097517	1	59153067	T	9	41	C	0.0003404
rs12737850	1	59153177	A	9	41	T	0.0003404
rs2223698	1	59155683	T	10	42	C	0.0006808
rs12076049	1	59156155	C	9	41	T	0.0003404
rs12755615	1	59156825	G	10	43	C	0.0003854
rs2746081	1	59158161	A	9	41	G	0.0003404
rs12097333	1	59158632	T	9	41	C	0.0003404
rs12741968	1	59159062	G	9	41	T	0.0003404
rs2746082	1	59160283	G	9	41	C	0.0005204

rs11207293	1	59160380	G	9	40	C	0.0005923
rs66629118	1	59160724	TA	8	43	T	0.00005831
rs2473746	1	59160735	G	8	41	A	0.0002447
rs2064374	1	59160885	G	9	41	A	0.0003404
rs5774423	1	59160982	A	9	41	ATACT	0.0003404
rs2811893	1	59162148	C	9	41	T	0.0003404
rs4912373	1	59162493	C	9	41	A	0.0003404
rs6701772	1	59162675	A	10	42	G	0.0006808
rs6677560	1	59162793	G	9	41	A	0.0003404
rs112756138	1	59162923	GAGGC	9	41	G	0.0003404
rs6656037	1	59163286	A	8	40	C	0.0004251
rs6680626	1	59163441	G	9	40	A	0.000591
rs6680750	1	59163553	G	10	42	A	0.0006808
rs11803186	1	59164140	G	9	41	A	0.0003404
rs11368794	1	59164220	AG	9	41	A	0.0003404
rs10691262	1	59164244	AAAAATT	10	42	A	0.0006808
rs10889110	1	59164512	T	9	41	C	0.0003404
rs11207298	1	59167316	A	10	42	C	0.0006808
rs10889111	1	59169839	C	10	45	A	0.0001954
rs10889112	1	59169895	C	11	46	T	0.0002401
rs10889113	1	59173187	G	10	42	A	0.0003844
rs10159308	1	59174129	G	10	45	A	0.0001954
rs5774424	1	59175216	A	10	45	AAG	0.0001954
rs10158502	1	59175979	A	10	45	C	0.0001954
rs6668605	1	59179331	G	11	45	A	0.0004315
rs1323280	1	59201807	C	9	42	T	0.0003081
rs201586451	1	61446080	A	9	1	AG	0.0007194
rs1260836	1	64488856	A	6	34	G	0.0005004
rs2991270	1	65239444	C	33	28	A	0.0009909
rs310239	1	65277910	T	33	28	A	0.0009909
rs1333739	1	65293449	T	33	28	A	0.0009909
rs113399410	1	65857369	G	7	0	GA	0.0008679
rs11208644	1	65858151	T	7	0	C	0.0008679
rs59610328	1	65859783	AGC	7	0	A	0.0008679
rs7532967	1	65859869	C	7	0	A	0.0008679
rs7555604	1	65859910	G	7	0	C	0.0008679
rs12077111	1	65860660	C	7	0	T	0.0008679
rs3930551	1	65860874	G	7	0	T	0.0008679
rs7556443	1	65861340	C	7	0	T	0.0008679
rs10218548	1	65861555	C	7	0	A	0.0008679
rs9326067	1	65861660	C	7	0	T	0.0008679

rs4915689	1	65862079	G	7	0	C	0.0008679
rs4916048	1	65862508	G	7	0	C	0.0008679
rs4916049	1	65862747	A	7	0	G	0.0008679
rs4916050	1	65862844	A	7	0	C	0.0008679
rs4916051	1	65862897	A	7	0	C	0.0008679
1:65862956	1	65862956	ATTG	7	0	A	0.0008679
1:65862957	1	65862957	CCCACCTGCAA	7	0	C	0.0008679
rs12087420	1	65864009	G	7	0	A	0.0008679
rs10889547	1	65864198	G	7	0	A	0.0008679
rs10889548	1	65864220	T	7	0	G	0.0008679
rs7415248	1	65865662	G	7	0	A	0.0008679
rs4915690	1	65866667	G	7	0	T	0.0008679
rs4915692	1	65867715	T	7	0	C	0.0008679
rs143451102	1	68769719	G	7	0	A	0.0008679
rs148941019	1	68796152	T	7	0	A	0.0008679
rs145790006	1	68832288	A	8	0	G	0.0003016
rs71095930	1	71590995	T	21	11	TC	0.0003893
rs74365843	1	71652676	T	29	21	C	0.0005775
rs1417709	1	71656481	A	29	22	G	0.0007063
rs2639329	1	71660120	G	29	22	A	0.0007063
rs2796201	1	71660796	C	29	22	T	0.0007063
rs1954285	1	71662977	T	29	21	A	0.0005775
rs952337	1	71663414	T	29	21	A	0.0005775
rs2796203	1	71665671	T	29	21	C	0.0005775
rs2066371	1	71667881	G	29	22	A	0.0007063
rs1573061	1	71668906	C	29	21	T	0.0005775
rs2639338	1	71672398	A	29	21	G	0.0005775
rs2639339	1	71672677	G	29	21	C	0.0005775
rs12406316	1	71673738	T	29	21	C	0.0005775
rs2639335	1	71676127	A	29	21	G	0.0005775
rs2639334	1	71677428	G	29	22	A	0.0007063
rs2249759	1	71681377	C	29	22	T	0.0007063
rs370402759	1	71683102	G	29	22	C	0.0007063
rs12759919	1	71685633	T	29	21	G	0.0005775
rs9726375	1	71686237	C	29	21	A	0.0005775
rs2796190	1	71698274	A	29	21	G	0.0005775
rs2639331	1	71698461	T	29	21	C	0.0005775
rs2796192	1	71698946	C	29	22	G	0.0007063
rs2639330	1	71699013	T	29	21	C	0.0005775
rs2790674	1	71719363	C	27	19	T	0.0008555
rs35251740	1	71746844	CT	15	5	C	0.0002217

rs368186205	1	85615108	G	18	8	A	0.0002786
rs28663573	1	88656453	G	35	28	C	0.0001616
rs1336584	1	88656688	T	35	28	C	0.0001616
rs12755168	1	88657007	T	37	28	G	0.00003443
rs34293383	1	88658666	G	37	29	GA	0.00007345
rs12145817	1	88662490	G	39	30	A	0.00001738
rs12117110	1	88663269	G	39	30	A	0.00001738
rs10923040	1	88664008	C	36	29	T	0.0001509
rs11577821	1	88676956	C	16	7	G	0.0009539
rs10923044	1	88681580	C	16	7	T	0.0009539
rs2039626	1	88684866	G	14	5	A	0.0008508
rs4656023	1	88688065	G	14	5	A	0.0008508
rs59267365	1	88698986	T	17	7	C	0.0004382
rs12407424	1	88832596	G	7	0	A	0.0008679
rs12123312	1	90305865	T	25	16	A	0.0005585
rs79573214	1	98669432	T	14	5	G	0.0008508
rs72719817	1	98671145	G	14	5	C	0.0008508
rs371116664	1	98671431	A	14	5	AAAGG	0.0008508
rs143827852	1	98673434	GA	14	5	G	0.0008508
rs116196581	1	98673909	C	14	5	G	0.0008508
rs78249251	1	98675141	G	14	5	C	0.0008508
rs11342720	1	98675992	C	14	5	CT	0.0008508
rs138511666	1	98679233	C	14	5	A	0.0008508
rs72719839	1	98695915	C	14	5	T	0.0008508
rs148603931	1	98697195	A	14	5	AC	0.0008508
rs10648324	1	98698522	GTGGA	14	5	G	0.0008508
rs6672621	1	98698557	T	14	5	C	0.0008508
rs12041603	1	98700623	T	14	5	C	0.0008508
rs2391926	1	98701571	G	14	5	A	0.0008508
rs12028859	1	98703049	C	14	5	T	0.0008508
rs11165972	1	98712294	G	14	5	T	0.0008508
rs12030857	1	98712337	T	14	5	G	0.0008508
rs12030892	1	98712356	A	14	5	G	0.0008508
rs12751197	1	98727780	C	28	20	A	0.0005398
rs730023	1	112515115	C	36	32	G	0.0006397
rs3008541	1	112516919	A	36	32	G	0.0006397
rs1373286	1	112517914	A	36	32	G	0.0006397
rs3000756	1	112518120	C	36	32	T	0.0006397
1:142538413	1	142538413	G	7	0	T	0.0008679
1:142538419	1	142538419	C	7	0	G	0.0008679
1:142538775	1	142538775	T	7	0	A	0.0008679

1:142577113	1	142577113	G	13	4	A	0.000883
1:143352871	1	143352871	C	7	0	T	0.0008679
1:148200111	1	148200111	T	7	0	C	0.0008679
rs77287809	1	152125335	A	7	0	C	0.0008679
rs12093512	1	152158481	T	7	0	C	0.0008679
rs186123580	1	152174073	T	7	0	C	0.0008679
rs11581057	1	156991688	C	17	6	T	0.0002592
rs11584902	1	157001597	C	17	6	G	0.0002592
rs34314027	1	157003864	C	17	6	T	0.0002592
1:157016023	1	157016023	GCCGCCA	16	5	G	0.0001588
rs150800619	1	157025409	CT	17	6	C	0.0002592
rs11577418	1	157026971	A	17	6	G	0.0002592
rs11576099	1	157027002	C	17	6	T	0.0002592
rs11576100	1	157027028	C	17	6	T	0.0002592
rs71593876	1	157027179	C	17	6	CT	0.0002592
rs35631733	1	157027288	T	17	6	TAA	0.0002592
rs11581893	1	157027520	T	17	6	C	0.0002592
rs35756545	1	157027944	T	17	6	C	0.0002592
rs12745721	1	157028212	T	17	6	G	0.0002592
rs34160895	1	157028313	A	17	5	AG	0.00006668
rs11582411	1	157028335	T	17	6	C	0.0002592
rs11577087	1	157028388	C	18	6	T	0.0000668
rs11578403	1	157028389	A	18	6	G	0.0000668
rs11578421	1	157028531	A	17	6	G	0.0002592
rs11577133	1	157028744	G	16	6	T	0.0005436
rs11582484	1	157028809	A	17	6	C	0.0002592
rs866605	1	157028990	T	17	6	C	0.0002592
rs61326781	1	157029134	C	17	6	T	0.0002592
rs11577688	1	157029857	C	17	6	T	0.0002592
rs61329932	1	157030077	A	17	6	T	0.0002592
rs58824481	1	157030082	T	17	6	C	0.0002592
rs59571768	1	157030099	A	17	6	G	0.0002592
rs1706	1	157030280	C	17	6	T	0.0002592
rs11590493	1	157030627	C	16	6	T	0.0005436
rs35256778	1	157031010	A	17	6	G	0.0002592
rs34994593	1	157031017	G	17	6	A	0.0002592
rs35045427	1	157031055	G	17	6	T	0.0002592
rs34230711	1	157031435	A	17	6	G	0.0002592
rs12735183	1	157031591	T	17	6	C	0.0002592
rs12759104	1	157031622	C	17	6	T	0.0002592
rs12734005	1	157031623	C	17	6	G	0.0002592

rs12759254	1	157031678	C	17	6	T	0.0002592
rs35209965	1	157031969	C	17	6	CT	0.0002592
rs12759732	1	157032001	C	17	6	T	0.0002592
rs35418037	1	157032593	A	17	6	G	0.0002592
rs59149386	1	157032706	T	17	6	C	0.0002592
rs34609359	1	157032832	C	17	6	T	0.0002592
rs34971986	1	157032883	G	17	6	A	0.0002592
rs11581819	1	157032995	T	17	6	C	0.0002592
rs11577808	1	157033027	A	17	6	G	0.0002592
rs11576914	1	157033294	A	17	6	T	0.0002592
rs11576533	1	157033505	G	17	6	A	0.0002592
rs35275017	1	157033610	C	17	7	CT	0.0004382
rs12731416	1	157033664	C	17	6	T	0.0002592
rs67062038	1	157033901	G	17	6	A	0.0002592
rs67278038	1	157033919	A	17	6	G	0.0002592
rs35025242	1	157033971	AT	16	6	A	0.0005436
rs34177345	1	157034213	A	17	6	G	0.0002592
rs34065145	1	157034246	C	17	6	T	0.0002592
rs34438489	1	157034325	C	17	6	T	0.0002592
rs11582800	1	157034471	T	17	6	C	0.0002592
rs11577472	1	157034503	A	17	6	T	0.0002592
rs35043156	1	157034715	G	17	6	A	0.0002592
rs138311748	1	157035303	T	17	6	C	0.0002592
rs115482314	1	157035522	G	17	6	C	0.0002592
rs146206625	1	157035548	A	17	6	C	0.0002592
rs144424473	1	157035772	C	17	6	T	0.0002592
rs12743010	1	157036195	G	17	6	A	0.0002592
rs34047481	1	157036233	CA	17	6	C	0.0002592
rs12760516	1	157036266	T	17	6	G	0.0002592
rs12747244	1	157036322	C	17	6	T	0.0002592
rs12747418	1	157036424	A	17	6	T	0.0002592
rs71593878	1	157036573	C	17	6	CT	0.0002592
rs66524118	1	157036657	T	17	6	C	0.0002592
rs67992922	1	157036686	C	17	6	T	0.0002592
rs67871969	1	157036870	G	17	6	A	0.0002592
rs35310943	1	157036956	C	17	6	G	0.0002592
rs34330037	1	157036971	T	17	6	C	0.0002592
rs34951264	1	157037242	C	17	6	G	0.0002592
rs35958339	1	157037270	A	17	6	AT	0.0002592
rs60784227	1	157037779	G	17	6	T	0.0002592
rs57543447	1	157037957	G	17	6	A	0.0002592

rs61407874	1	157038008	G	17	6	A	0.0002592
rs12728942	1	157038163	C	17	6	G	0.0002592
rs12753932	1	157038227	G	17	6	A	0.0002592
rs12758039	1	157038250	G	17	6	T	0.0002592
rs36051715	1	157038624	C	17	6	G	0.0002592
rs12759047	1	157038865	G	17	6	T	0.0002592
rs12759364	1	157038997	C	17	6	T	0.0002592
rs12759394	1	157039061	C	17	6	T	0.0002592
rs12755356	1	157039073	G	17	6	A	0.0002592
rs12725372	1	157039400	C	17	6	T	0.0002592
rs12725382	1	157039437	C	17	6	T	0.0002592
rs12739927	1	157039462	A	17	6	C	0.0002592
rs12739240	1	157039741	A	17	6	G	0.0002592
rs35904515	1	157040203	A	17	6	G	0.0002592
rs34909260	1	157040296	G	17	6	A	0.0002592
rs12755131	1	157040527	G	17	6	C	0.0002592
rs12740642	1	157040552	C	17	6	T	0.0002592
rs12741140	1	157040837	C	17	6	T	0.0002592
rs12755959	1	157041010	T	17	6	C	0.0002592
rs12737408	1	157041078	G	17	6	A	0.0002592
rs12756141	1	157041107	T	17	6	C	0.0002592
rs12760052	1	157041181	G	17	6	C	0.0002592
rs12760229	1	157041302	T	17	6	C	0.0002592
rs56801406	1	157044993	G	18	7	C	0.0001975
rs59462822	1	157045213	A	18	7	G	0.0001975
rs35188764	1	157046198	G	17	5	A	0.00006668
rs36032506	1	157046248	T	17	6	C	0.0002592
rs34074614	1	157047366	C	17	6	T	0.0002592
rs11587380	1	157048220	G	18	7	C	0.0001975
rs11587404	1	157048354	T	17	6	C	0.0002592
rs34961273	1	157050194	G	17	6	A	0.0002592
rs12752535	1	157050368	T	17	6	G	0.0002592
rs11578226	1	157051348	C	18	7	T	0.0001975
rs34328576	1	157051761	CCCT	17	6	C	0.0002592
rs149171885	1	157052071	TCAGTTTGGCATT	18	7	T	0.0001975
rs34414479	1	157052224	A	18	7	G	0.0001975
rs34369222	1	157052352	G	17	6	A	0.0002592
rs35090422	1	157052784	G	17	6	GA	0.0002592
rs34542108	1	157052794	T	17	6	C	0.0002592
rs11580245	1	157052987	A	17	6	G	0.0002592
rs35043649	1	157053226	ATT	17	6	A	0.0002592

rs1176561	1	157053342	T	17	5	C	0.00006668
rs12080191	1	157053415	T	17	6	C	0.0002592
rs11580501	1	157055760	C	17	6	T	0.0002592
rs67980707	1	157060544	G	17	6	A	0.0002592
rs67947469	1	157061643	T	17	6	C	0.0002592
rs12136960	1	157062739	G	22	10	C	0.00007379
rs72700786	1	157077970	T	13	4	C	0.000883
rs35947264	1	157079790	G	13	4	A	0.000883
rs2275703	1	160165095	C	38	33	A	0.0001874
rs35652278	1	160241692	AT	16	54	A	0.0003907
rs114513978	1	160241695	T	16	54	A	0.0003907
rs1886417	1	160313733	A	38	35	C	0.0006427
rs12143237	1	164491903	A	24	13	G	0.0001567
rs12402920	1	164492154	T	23	13	C	0.0003278
rs10918046	1	164526190	G	11	1	A	0.00009775
rs531650	1	170657994	C	8	40	T	0.0002504
1:172569838	1	172569838	A	13	3	G	0.0002409
rs570762	1	177890153	T	12	45	C	0.0009266
rs6424946	1	184290191	T	20	11	C	0.0008202
rs138042176	1	186630884	GT	5	31	G	0.0006549
rs5277	1	186648197	G	3	25	C	0.0009258
rs4549996	1	186707043	C	2	29	G	0.000017
rs200743165	1	188832851	T	7	0	G	0.000891
rs72060837	1	194371558	A	4	28	ATAATG	0.0008078
rs3010141	1	195854742	T	3	25	C	0.0009258
rs2923886	1	195855359	G	3	26	C	0.0005074
rs68003289	1	195858334	GGAAGATGAATA	3	26	G	0.0005074
rs3010143	1	195859677	C	3	26	T	0.0005074
rs11324284	1	195860011	TA	3	26	T	0.0005074
rs1451913	1	195863634	A	3	26	G	0.0005074
rs3008578	1	195864604	A	3	26	T	0.0005074
rs2923881	1	195867105	A	3	26	G	0.0005074
rs3010144	1	195867138	G	3	26	T	0.0005074
rs2923880	1	195868109	A	3	26	G	0.0005074
rs3008576	1	195869123	T	3	26	C	0.0005074
rs3008575	1	195869526	T	3	26	A	0.0005074
rs3010146	1	195870879	G	3	26	A	0.0005074
rs3010147	1	195871839	C	6	33	T	0.0009098
rs3010149	1	195872456	C	3	25	T	0.0009258
rs3010150	1	195873063	C	3	26	T	0.0005074
rs3008573	1	195874388	A	3	25	C	0.0009258

rs1451920	1	195874790	T	6	33	A	0.0009098
rs2123080	1	195874856	C	3	26	T	0.0005074
rs2167518	1	195874907	A	3	26	G	0.0005074
rs5779820	1	195875053	AT	3	26	A	0.0005074
rs3010151	1	195876205	T	3	26	C	0.0005074
rs3010152	1	195876254	T	6	33	A	0.0009098
rs3008572	1	195876292	C	3	26	T	0.0005074
rs3008571	1	195876392	C	3	25	T	0.0009258
rs3008570	1	195876537	A	6	33	T	0.0009098
rs3008569	1	195876542	C	3	26	T	0.0005074
rs2923904	1	195877044	A	3	26	G	0.0005074
rs5779821	1	195877194	AT	6	33	A	0.0009098
rs5779822	1	195877296	T	3	26	TAA	0.0005074
rs3008549	1	195880550	A	3	26	T	0.0005074
rs3008550	1	195880622	G	3	26	C	0.0005074
rs3008551	1	195880830	C	3	26	T	0.0005074
rs3008552	1	195881459	G	3	26	A	0.0005074
1:196477349	1	196477349	A	0	16	G	0.0005214
1:197957887	1	197957887	TA	0	15	T	0.0005628
1:198804284	1	198804284	A	6	34	AG	0.0004884
1:198804286	1	198804286	A	6	35	G	0.0002765
1:198804287	1	198804287	A	6	34	AAGGAAGGAAGGAAGGAA	0.0004884
rs78607681	1	200668908	G	9	0	A	0.0001034
rs113906106	1	200670819	T	10	0	C	0.00003497
rs113122215	1	200712919	G	8	0	A	0.0003016
rs200517518	1	201100129	A	26	18	AT	0.0007809
rs200108640	1	201100133	A	26	18	T	0.0007809
rs61136828	1	202167795	G	16	5	GGTTTTTTTTTTT	0.0001864
rs199940288	1	203750337	A	9	0	AAAG	0.0001214
rs71568011	1	204013671	AAG	16	7	A	0.0009539
rs12046258	1	204039160	A	18	8	G	0.0005897
rs77321629	1	204042433	G	18	8	T	0.0005897
rs17404301	1	204047520	A	18	8	G	0.0005897
rs71568016	1	204049014	G	18	8	GCCT	0.0005897
rs12117788	1	204049200	A	18	8	G	0.0005897
rs12727635	1	204052988	G	18	8	C	0.0005897
rs34618698	1	204056948	A	13	2	G	0.00006438
rs59065201	1	204067376	T	20	11	C	0.0008202
rs61825739	1	204067474	A	14	5	G	0.0008508
rs12034981	1	204069237	A	15	5	C	0.0003715
rs7539399	1	204590044	G	6	32	A	0.00098

rs7551222	1	204599295	G	6	34	A	0.0005004
rs7553683	1	204599461	G	6	34	A	0.0005004
rs4951084	1	204606974	C	6	33	G	0.0009098
rs12024827	1	204607874	T	12	47	C	0.0004795
rs4951085	1	204608284	C	12	47	G	0.0004795
rs4951086	1	204608643	T	8	42	C	0.0000745
rs4951087	1	204609194	A	12	47	G	0.0004795
rs4950954	1	204610475	G	12	47	A	0.0004795
rs4951088	1	204610780	T	12	47	C	0.0004795
rs4951089	1	204610898	T	12	47	C	0.0004795
rs138881554	1	204611658	C	12	47	CAAGGGAGGG	0.0004795
rs11240234	1	204612138	T	12	47	C	0.0004795
rs11240235	1	204612200	T	12	47	C	0.0004795
rs11240236	1	204612463	T	12	47	G	0.0004795
rs11240237	1	204612518	A	12	47	G	0.0004795
rs11240238	1	204612537	T	12	47	C	0.0004795
rs11240239	1	204612804	A	12	47	G	0.0004795
rs4279904	1	204613764	A	9	45	G	0.0000477
rs10900418	1	204614166	T	9	45	C	0.0000477
rs2772227	1	204615400	T	8	41	A	0.0001361
rs1329631	1	204616390	G	12	45	A	0.0009266
rs12127178	1	204620354	A	12	47	G	0.0004795
rs2772244	1	204621178	G	9	44	A	0.0000882
rs2492985	1	204621247	A	9	44	G	0.0000882
rs880497	1	204622214	C	12	45	G	0.0009266
rs2480467	1	204624957	A	10	43	G	0.0003854
rs1120599	1	209670782	T	8	39	C	0.0004628
rs1011398	1	209705895	T	6	34	C	0.0005004
rs11119358	1	210042456	C	2	26	T	0.0001452
rs2064163	1	210048819	T	3	27	G	0.0002808
rs2143545	1	210062667	C	15	51	T	0.0006974
rs12078439	1	210075260	G	37	34	T	0.0006983
rs34704551	1	210107045	C	25	15	A	0.0002543
rs66736737	1	210109844	G	24	15	GAGTCTCCTGGACAAATA	0.0005235
rs6657016	1	210114848	A	24	15	T	0.0005235
rs34931444	1	210125122	G	25	15	A	0.0002543
rs10779516	1	210128127	T	37	34	C	0.0006983
rs56131913	1	210146047	C	25	15	T	0.0002543
rs75456071	1	210159801	T	23	14	A	0.0007414
rs11590161	1	210165928	C	23	14	G	0.0007414
rs61820396	1	210167102	G	23	14	C	0.0007414

rs7543650	1	210170975	T	23	14	A	0.0007414
rs12750599	1	210173234	C	23	14	T	0.0007414
rs34076794	1	210174598	A	23	14	AT	0.0007414
rs12401646	1	210182369	G	23	14	A	0.0007414
rs61820397	1	210189249	A	23	14	G	0.0007414
rs71571925	1	210197923	C	23	14	CA	0.0007414
rs17318612	1	210206179	T	23	14	A	0.0007414
rs61826848	1	210220847	G	23	14	A	0.0007414
rs17318744	1	210244470	G	23	14	A	0.0007414
rs2484034	1	210254787	A	23	14	G	0.0007414
rs2451696	1	210282346	T	25	14	G	0.0001947
rs2451697	1	210284025	G	25	14	A	0.0001947
rs2484033	1	210287837	A	25	14	G	0.0001947
rs2484030	1	210296910	G	25	14	T	0.0001947
rs2484031	1	210303614	C	25	14	A	0.0001947
rs35028401	1	210317184	C	14	5	T	0.0008508
rs6660244	1	210379156	A	11	2	G	0.0004434
rs17757020	1	210396557	T	11	2	C	0.0004434
rs12026739	1	210431517	T	11	2	C	0.0004434
rs12044365	1	210431761	G	11	2	A	0.0004434
rs34285465	1	210439000	T	11	2	G	0.0004434
rs55673030	1	210449901	T	11	2	C	0.0004434
rs12069491	1	210460899	G	2	22	A	0.0009583
rs12070628	1	210460909	C	2	22	T	0.0009583
rs79578370	1	210462978	G	2	21	GTGTAATTCC	0.0009745
rs590380	1	210463071	A	2	23	G	0.0005167
rs689248	1	210463122	T	2	23	C	0.0005167
rs11453134	1	210463173	CT	2	23	C	0.0005167
rs588197	1	210463515	T	2	23	A	0.0005167
rs583148	1	210463553	C	2	23	A	0.0005167
rs587203	1	210463786	A	2	23	G	0.0005167
rs61621397	1	210463870	G	2	23	GAA	0.0005167
rs608791	1	210464638	T	2	23	C	0.0005167
rs652256	1	210465017	C	2	23	T	0.0005167
rs652241	1	210465029	T	2	23	A	0.0005167
rs650854	1	210465315	C	2	23	G	0.0005167
rs5780569	1	210465434	ATATAAT	2	24	A	0.0002818
rs1338290	1	210465467	A	2	24	G	0.0002818
rs635084	1	210466625	A	20	10	G	0.0005561
rs639095	1	210466818	A	20	10	T	0.0005561
rs12030512	1	210466886	G	2	25	A	0.0001556

rs7516171	1	210466932	T	20	10	C	0.0005561
rs7416272	1	210467425	A	20	9	G	0.0002046
rs12030856	1	210471463	G	2	25	T	0.0001556
rs58366398	1	210471924	A	2	25	G	0.0001556
rs12040684	1	210472053	T	2	25	C	0.0001556
rs12074229	1	210474602	G	2	25	A	0.0001556
rs12034871	1	210475701	C	2	25	T	0.0001556
rs2057044	1	210477579	T	20	10	A	0.0005561
rs61848993	1	211095666	T	27	20	C	0.0008778
rs10494947	1	212089141	A	7	0	C	0.0008679
rs562410	1	215139180	C	16	55	T	0.0003306
rs476557	1	215140251	C	14	52	A	0.0001845
rs507072	1	215140520	C	14	53	T	0.0001565
rs11120456	1	215142401	C	10	50	A	0.00001223
rs487208	1	215143278	A	13	53	G	0.00004294
rs1890007	1	215146411	T	13	52	A	0.0000799
rs864736	1	215150260	A	16	54	C	0.0003907
rs543029	1	215151063	A	16	54	G	0.0003907
rs495296	1	215153348	C	16	55	T	0.0003306
rs1452632	1	215154120	A	13	52	C	0.0000799
rs6681013	1	215154797	G	13	52	C	0.0000799
rs566980	1	218295397	A	9	1	G	0.0007194
rs12403383	1	218296752	A	12	2	C	0.0001711
rs75332983	1	218302164	A	12	2	G	0.0001711
rs12033297	1	218302651	T	12	2	C	0.0001711
rs12045142	1	218305929	A	11	2	G	0.0004434
rs74226968	1	218311278	A	12	2	T	0.0001711
rs2069094	1	218313036	A	11	2	G	0.0004434
rs146197820	1	220565631	TC	31	24	T	0.0004543
rs72760323	1	224767537	A	0	16	G	0.0005294
rs6672413	1	224817129	C	3	25	T	0.0009258
rs6672806	1	224817506	A	3	25	T	0.0009258
1:226417566	1	226417566	GGT	8	0	G	0.0003016
1:228614740	1	228614740	C	31	25	CT	0.0003995
1:228614744	1	228614744	C	31	25	CTCCTTCCCTCCCTTCCTTTC	0.0003995
rs6656666	1	233635440	T	12	45	A	0.0009266
rs597474	1	234450288	C	8	38	G	0.0005059
rs149002825	1	234518776	AGAGAAG	8	42	A	0.0002193
rs10797542	1	234562268	A	9	1	G	0.0007194
rs10797543	1	234562287	G	9	1	A	0.0007194
rs10910434	1	234563556	A	9	1	T	0.0007194

rs10797546	1	234564011	G	9	1	A	0.0007194
rs10797547	1	234564203	T	9	1	C	0.0007194
rs10797548	1	234564205	C	9	1	G	0.0007194
rs71701823	1	234564864	G	9	1	GAA	0.0007194
rs915184	1	234565787	A	9	1	G	0.0007194
rs2236587	1	234566085	G	9	1	A	0.0007194
rs72632290	1	234566681	T	9	1	C	0.0007194
rs10910435	1	234566943	C	9	1	T	0.0007194
rs10910436	1	234567301	G	9	1	C	0.0007194
rs10797549	1	234567581	A	9	1	G	0.0007194
rs12024737	1	234568101	T	9	1	C	0.0007194
rs12045698	1	234568114	A	9	1	G	0.0007194
rs12045759	1	234568264	A	9	1	G	0.0007194
rs74308430	1	234568685	TC	9	1	T	0.0007194
rs112979975	1	234569032	AC	9	1	A	0.0007194
rs3754311	1	234569192	C	9	1	T	0.0007194
rs12026726	1	234569772	A	9	1	C	0.0007194
rs12048608	1	234570889	A	9	1	G	0.0007194
rs112010867	1	234571123	CGG	9	1	C	0.0007194
rs12047138	1	234571854	A	9	1	T	0.0007194
rs12028777	1	234571886	T	9	1	C	0.0007194
rs12049594	1	234571888	A	9	1	G	0.0007194
rs12046432	1	234571928	G	9	1	A	0.0007194
rs12064512	1	234574937	G	9	1	C	0.0007194
rs13374387	1	234576405	A	9	1	C	0.0007194
1:238178375	1	238178375	G	11	2	T	0.0004434
rs113242973	1	238957843	G	7	0	A	0.0008062
rs60728123	1	240279926	T	16	7	C	0.0009539
rs6656703	1	242539132	G	18	9	A	0.0009279
rs6429351	1	242541697	C	18	9	T	0.0009279
rs60251166	1	242543844	G	18	9	A	0.0009279
rs11485478	1	248502769	T	2	22	C	0.0009583
2:582907	2	582907	A	7	0	AAAGAAGCTCCACGCAGCG	0.0008679
rs12714409	2	596532	T	13	4	C	0.000883
rs12999227	2	597382	T	13	4	C	0.000883
2:722604	2	722604	TTATG	1	21	T	0.0004716
rs7576110	2	758088	G	18	9	C	0.0009279
rs2674225	2	2591136	C	24	14	G	0.0003806
rs202066201	2	3528553	C	23	14	CTT	0.0007414
rs7583559	2	15866020	G	22	11	C	0.0001874
rs2380671	2	15867707	G	23	12	A	0.0002434

rs4668964	2	15867762	T	20	11	C	0.0008202
rs4668965	2	15868003	C	19	9	T	0.0004395
rs11096512	2	18295234	T	23	13	C	0.0003278
rs1653773	2	23735843	G	37	34	A	0.0006983
rs10203512	2	23736637	G	37	34	A	0.0006983
rs1653775	2	23737302	G	37	34	C	0.0006983
rs12712975	2	23741410	A	27	19	G	0.0008555
rs1275984	2	26911509	A	32	26	C	0.0008799
rs1275986	2	26912079	C	33	27	A	0.0005258
rs736699	2	26913930	A	32	26	G	0.0008799
rs935168	2	26914787	G	32	23	A	0.0001132
rs34004783	2	26916846	C	31	23	G	0.0003729
rs1275977	2	26922855	G	31	23	A	0.0003729
rs13394970	2	26929282	T	33	27	G	0.0005258
rs1731243	2	26930411	C	33	28	T	0.0009909
rs2586886	2	26932031	C	33	28	T	0.0009909
rs1275923	2	26932796	C	33	28	T	0.0009909
rs71446010	2	31859714	C	1	19	T	0.0008417
rs35890136	2	32861027	G	5	33	T	0.0002023
rs72864592	2	35747234	A	20	11	G	0.0008202
rs4670667	2	37392402	C	8	37	A	0.0008686
rs28428996	2	37393941	C	8	38	T	0.0005059
rs151238316	2	37678662	T	16	7	TTTG	0.0009539
rs150795296	2	38311413	G	15	52	A	0.0006042
rs162332	2	38322361	A	15	52	G	0.0006042
rs62141389	2	41019721	T	13	47	C	0.0009845
rs56100439	2	41021497	A	13	47	C	0.0009845
rs2280220	2	45391901	G	21	12	A	0.0009318
rs1922043	2	47794960	C	2	22	T	0.0009583
rs145653565	2	52269404	C	11	43	A	0.0008678
rs140336606	2	52271010	A	8	37	G	0.0008686
rs372996983	2	52271700	T	7	38	C	0.0001991
rs184082143	2	52271831	A	11	43	C	0.0008678
rs36138953	2	52272508	T	10	42	C	0.0006808
rs34393418	2	52274004	C	9	40	G	0.000591
rs13015554	2	52274513	T	11	43	C	0.0008678
rs13429833	2	52276727	T	10	42	C	0.0006808
rs10164955	2	52277943	T	10	42	C	0.0006808
rs4504033	2	52280819	G	9	40	C	0.000591
rs12472125	2	52281116	A	11	43	T	0.0008678
rs7559419	2	52284070	G	11	43	A	0.0008678

rs10177709	2	52284768	G	11	43	A	0.0008678
rs12612665	2	54908210	A	31	25	G	0.0008555
rs72618666	2	54919369	G	31	24	T	0.0004543
rs72618668	2	54919594	G	31	24	A	0.0004543
rs6749785	2	56049242	G	16	53	T	0.0006806
rs7426380	2	56095010	G	16	53	A	0.0006806
rs2192515	2	60050857	G	12	45	A	0.0009266
2:60676657	2	60676657	C	2	25	T	0.0002841
rs10165560	2	62059398	C	4	28	A	0.0008901
rs115595309	2	67226030	A	11	2	C	0.0004434
rs112889343	2	74647099	A	11	2	G	0.0004434
rs61311422	2	75386176	CT	13	47	C	0.0009845
rs3771843	2	75386776	C	11	46	A	0.0002401
rs3821317	2	75386811	G	13	47	C	0.0009845
rs17640500	2	75386860	G	12	46	A	0.0005428
rs10194954	2	75386871	C	12	46	T	0.0005428
rs10207441	2	75386881	T	12	46	C	0.0005428
rs3771844	2	75386925	T	12	46	C	0.0005428
rs3821318	2	75387311	T	12	45	C	0.0009266
rs10198644	2	75388053	G	12	45	C	0.0009266
rs10177213	2	75388125	A	12	45	G	0.0009266
rs12713834	2	75391100	A	11	45	G	0.0004315
rs2058859	2	75391574	G	12	45	A	0.0009266
rs2216307	2	75392831	T	5	34	C	0.0001138
rs11886595	2	75681052	C	16	6	T	0.0005436
2:76593575	2	76593575	C	9	0	T	0.0001173
rs954491	2	76964356	G	1	19	T	0.0008417
2:81108696	2	81108696	C	7	0	CAT	0.0008679
rs10175174	2	85287948	T	28	20	C	0.0005398
rs4019832	2	85296388	A	32	24	G	0.0002243
rs113625787	2	85296601	A	31	23	G	0.0003995
rs55869261	2	85298955	C	32	24	T	0.0002243
rs62162805	2	85300366	A	32	24	C	0.0002243
rs1975130	2	85301540	A	32	24	T	0.0002243
rs59640363	2	85304912	C	32	25	T	0.0004454
rs4832133	2	85305903	C	32	24	G	0.0002243
rs11890007	2	85306122	G	32	24	A	0.0002243
rs146964623	2	85307222	T	32	24	TC	0.0002243
rs113625990	2	85307290	G	32	24	C	0.0002243
rs62162807	2	85307445	A	31	23	G	0.0003729
rs13430659	2	85308534	T	32	24	C	0.0002243

rs13393644	2	85308872	C	32	24	T	0.0002243
rs12714136	2	85310080	G	32	26	A	0.0008799
2:85571001	2	85571001	G	7	0	A	0.0008679
rs6724470	2	85571005	T	7	0	A	0.0008679
2:91737945	2	91737945	ATTTCATCTCATCA	14	4	A	0.0003939
rs75746990	2	92166963	A	13	4	T	0.000883
rs201649520	2	92316009	A	13	4	G	0.000981
2:100271679	2	100271679	G	28	18	C	0.0001825
2:127729242	2	127729242	AGACCAGCCCTGT	13	4	A	0.000883
rs13416459	2	129864010	G	18	8	A	0.0005897
rs56009990	2	129864416	A	18	8	G	0.0005897
2:130676946	2	130676946	A	13	4	G	0.000883
rs62165681	2	134908823	T	11	2	G	0.0004434
rs76856516	2	136739259	T	8	0	G	0.0003372
rs7597859	2	141237995	G	9	1	T	0.0007194
rs6753651	2	141239345	G	9	1	A	0.0007194
rs76407510	2	146348200	T	1	19	C	0.0008417
rs75823384	2	146353540	C	1	19	T	0.0008417
rs12615318	2	146354167	A	1	19	C	0.0008417
rs75180379	2	146356926	T	1	19	G	0.0008417
rs78270796	2	146364506	G	1	19	A	0.0008417
rs77283667	2	146368972	A	1	19	T	0.0008417
rs115248214	2	146373817	G	1	19	A	0.0008417
rs74769191	2	146377138	G	1	19	A	0.0008417
rs6707541	2	146378480	A	1	19	G	0.0008417
rs2381750	2	146382236	T	1	19	G	0.0008417
rs79180145	2	146388853	A	1	19	C	0.0008417
rs12995143	2	148965908	T	28	20	C	0.0005398
rs4972338	2	148974872	T	30	21	A	0.0001793
rs11685302	2	148980230	T	30	21	C	0.0001793
rs10432369	2	148982641	A	30	19	C	0.00006044
rs368079176	2	148982873	T	30	19	TAAAC	0.00006044
rs2167127	2	148983394	T	30	21	C	0.0001793
rs12691780	2	148983760	C	30	21	T	0.0001793
rs4399670	2	148985774	C	30	19	G	0.00006044
rs142457231	2	148985860	C	30	21	A	0.0001793
rs12691781	2	148987013	A	30	21	T	0.0001793
rs10928388	2	148987453	A	30	21	G	0.0001793
rs7570104	2	148988832	G	30	21	A	0.0001793
rs34002489	2	148989115	CT	30	21	C	0.0001793
rs2044760	2	148989731	T	30	21	C	0.0001793

rs2044761	2	148989742	A	30	21	G	0.0001793
rs987626	2	148990144	T	30	19	G	0.00006044
rs987625	2	148990155	C	30	19	G	0.00006044
rs1449960	2	148990179	G	30	21	A	0.0001793
rs7572948	2	148992803	C	30	19	A	0.00006044
rs13022005	2	148993822	A	30	19	T	0.00006044
rs2839755	2	148993952	C	30	19	T	0.00006044
rs13001946	2	148994598	G	30	19	A	0.00006044
rs374750204	2	148996565	CAT	31	22	C	0.0001772
rs7575333	2	148996571	C	31	20	T	0.00004024
rs6721794	2	148999403	C	30	21	T	0.0001793
rs10598345	2	149000901	A	30	19	ATT	0.00006044
rs17274896	2	149001029	C	30	19	A	0.00006044
rs10928389	2	149002181	A	30	21	T	0.0001793
rs13027785	2	149003522	A	30	21	C	0.0001793
rs70992200	2	149005231	A	30	21	ACT	0.0001793
rs11692246	2	149005881	C	30	19	T	0.00006044
rs11676308	2	149006021	G	30	19	A	0.00006044
rs10210377	2	149007885	T	30	21	C	0.0001793
rs13425594	2	149009833	G	30	21	A	0.0001793
rs1453318	2	149010800	G	30	19	A	0.00006044
rs6746932	2	149012777	C	30	19	T	0.00006044
rs2084954	2	149014250	T	31	22	C	0.0001772
rs35042809	2	149015017	C	31	22	T	0.0001772
rs2890917	2	149015704	T	31	24	A	0.0004543
rs13032996	2	149016812	C	31	22	A	0.0001772
rs1120671	2	149018605	G	31	24	A	0.0004543
rs6430305	2	149019608	T	31	24	A	0.0004543
rs11452364	2	149023500	G	30	21	GA	0.0001793
rs961207	2	149024933	A	30	21	G	0.0001793
rs12053104	2	149036219	T	30	20	C	0.0001367
rs4972277	2	149039379	T	30	20	C	0.0001367
rs147698344	2	149040112	A	30	20	AT	0.0001367
rs11675979	2	149041386	C	30	20	A	0.0001367
rs1869341	2	149042682	C	30	20	A	0.0001367
rs4972347	2	149046472	A	30	20	T	0.0001367
rs4972278	2	149046830	A	30	20	C	0.0001367
rs11683219	2	149050998	G	30	20	A	0.0001367
rs11678688	2	149055476	T	30	20	C	0.0001367
rs949679	2	149061500	A	30	20	G	0.0001367
rs4972355	2	149062824	A	30	21	G	0.0001793

rs12470061	2	149063513	T	30	20	C	0.0001367
rs2656324	2	149070920	T	30	20	C	0.0001367
rs2656320	2	149073443	A	30	20	G	0.0001367
rs2656319	2	149073507	A	30	20	G	0.0001367
rs2602358	2	149077501	G	30	20	A	0.0001367
rs2602357	2	149079445	T	30	20	C	0.0001367
rs2656317	2	149079447	T	30	19	A	0.00006044
rs28831467	2	149085230	G	30	20	C	0.0001367
rs2167880	2	149086619	T	30	20	C	0.0001367
rs17276501	2	149087536	T	30	20	C	0.0001367
rs1453317	2	149090114	A	30	20	C	0.0001367
rs2656325	2	149097252	G	30	20	A	0.0001367
rs35177340	2	149100102	T	28	19	A	0.0002695
rs2602352	2	149100152	C	30	20	T	0.0001367
rs2938705	2	149102437	A	30	20	G	0.0001367
rs62183965	2	149102712	A	28	19	G	0.0002695
rs12618358	2	149105710	C	30	20	G	0.0001367
rs869595	2	149108684	A	30	20	G	0.0001367
rs141834862	2	149109148	C	30	20	CA	0.0001367
rs4972358	2	149110717	A	30	20	G	0.0001367
rs10196090	2	149112792	G	30	20	T	0.0001367
rs6734448	2	149113561	C	30	20	T	0.0001367
rs7592019	2	149115939	C	30	19	T	0.00006044
rs10208839	2	149116334	G	30	19	T	0.00006044
rs2382226	2	149118062	A	30	19	G	0.00006044
rs12478120	2	149128717	A	30	19	T	0.00006044
rs141222971	2	149138948	T	30	17	C	0.00001954
rs146839713	2	149140476	T	30	17	C	0.00001954
rs141039307	2	149141219	CA	30	17	C	0.00001954
rs201766774	2	149142262	T	30	17	G	0.00001954
rs6730847	2	149142703	G	30	17	A	0.00001954
rs6730848	2	149142707	G	30	17	C	0.00001954
rs6730640	2	149142724	C	30	17	T	0.00001954
rs6745554	2	149142725	T	30	17	C	0.00001954
rs6731266	2	149143093	G	30	17	A	0.00001954
rs11899163	2	149143496	G	30	18	A	0.00002692
rs12464361	2	149153361	G	26	17	C	0.0003852
rs12469720	2	149154514	T	26	17	G	0.0003852
rs1404187	2	149157710	A	26	17	G	0.0003852
rs34263784	2	150554783	G	7	0	T	0.0008679
rs35950612	2	150595052	G	7	0	A	0.0008679

rs35287908	2	151316181	CA	20	11	C	0.0008202
rs16830686	2	152954035	T	7	0	C	0.0008679
rs10203804	2	155603154	A	11	43	G	0.0008678
rs13029130	2	158698252	T	3	25	C	0.0009258
rs114545093	2	167790469	A	9	1	G	0.0007194
rs116531315	2	167853218	C	10	1	T	0.0002681
rs7591667	2	168538458	A	9	1	G	0.0007194
rs114644345	2	170706904	A	7	0	G	0.0008679
rs77763348	2	170778065	A	7	0	G	0.0008679
rs115502145	2	170781206	C	7	0	T	0.0008679
rs116794266	2	170871344	A	7	0	G	0.0008679
rs144291934	2	170894864	T	7	0	TACC	0.0008679
rs34509009	2	171768835	AT	6	37	A	0.00008623
rs11902360	2	171768908	G	7	39	A	0.0001101
rs11902369	2	171768951	G	6	38	C	0.00003819
rs2195116	2	171785428	C	6	37	T	0.00008623
rs731693	2	171797234	A	9	40	G	0.000591
rs4025984	2	176194225	T	4	31	C	0.0002188
rs13032587	2	177683404	T	6	32	C	0.00098
rs12469347	2	177688749	T	6	32	G	0.00098
rs1517709	2	180333881	C	6	32	G	0.00098
2:183127685	2	183127685	TGTGTGTGTGG	0	18	T	0.0001312
rs1997131	2	185043362	A	0	15	G	0.0005628
rs1526197	2	185050101	A	0	15	G	0.0005628
rs6434076	2	185052186	A	0	15	C	0.0005628
rs62176371	2	185151931	T	0	15	C	0.0005628
rs10177272	2	185189820	A	0	14	G	0.0009501
rs376234709	2	185196329	G	0	15	A	0.0005628
rs2219224	2	185211739	A	40	37	G	0.0003306
rs1378675	2	185213344	G	40	38	A	0.0003907
rs10209231	2	185221959	T	7	35	G	0.0007198
rs1443021	2	185226984	T	7	35	A	0.0007198
rs4233783	2	185227769	A	7	36	T	0.0006639
rs1596269	2	185239448	G	7	35	T	0.0007198
rs1596270	2	185251767	T	7	35	C	0.0007198
rs1596271	2	185251841	C	7	35	T	0.0007198
rs1579762	2	185252515	G	7	35	A	0.0007198
rs981091	2	185257415	G	7	35	A	0.0007198
rs2369337	2	185264965	C	7	36	T	0.0006639
rs2034059	2	185275344	T	7	36	A	0.0006639
rs2034058	2	185276674	C	7	36	A	0.0006639

rs1443033	2	185277576	T	7	36	C	0.0006639
rs10204602	2	185279087	G	7	37	A	0.0003628
2:196431908	2	196431908	T	8	0	TCCCGTCTCCC	0.000247
2:203185279	2	203185279	C	12	49	T	0.0001424
rs17248627	2	206042059	G	7	0	A	0.0008679
rs62172730	2	206046970	T	7	0	C	0.0008679
rs905883	2	213188470	C	0	15	T	0.0005628
rs7565150	2	213585035	T	0	18	C	0.0001312
rs7369041	2	213585180	G	0	15	T	0.0005628
rs6435724	2	213585190	C	0	18	T	0.0001312
rs11678818	2	213627986	T	0	17	C	0.0002606
rs4143570	2	214653497	C	35	29	G	0.0003069
rs55658550	2	214690983	T	32	25	G	0.0004454
rs72941141	2	214723852	C	34	27	G	0.000994
2:214725204	2	214725204	TAG	29	21	T	0.0006145
rs7597948	2	214826315	G	37	34	T	0.0009371
rs4674090	2	217400187	G	8	0	C	0.0003016
rs1179733	2	217725474	G	5	34	C	0.0001138
rs1179722	2	217733731	T	6	34	C	0.0005004
rs35422889	2	219809760	T	3	29	C	0.0001425
2:224139541	2	224139541	TA	7	0	T	0.0008679
rs10599484	2	225257525	C	20	11	CTA	0.0008202
rs35573107	2	225834004	T	1	24	C	0.00006045
rs59406413	2	225835800	A	1	24	C	0.00006045
rs4674956	2	225837435	A	1	24	C	0.00006045
rs57462780	2	225837562	C	1	24	A	0.00006045
rs10167041	2	228123661	G	37	30	A	0.00008804
rs4332918	2	228124702	G	36	30	A	0.000303
rs57569440	2	228125276	GA	37	30	G	0.00008804
rs6436670	2	228126494	C	13	48	T	0.0005647
rs13421399	2	229022674	A	26	17	G	0.0003852
rs75339037	2	229022874	G	17	7	A	0.0004382
rs76762410	2	229022909	A	17	7	G	0.0004382
rs55851318	2	230234141	G	11	2	A	0.0004571
2:237235440	2	237235440	T	18	8	TG	0.0005897
rs11884821	2	238169567	G	7	0	T	0.0008679
rs12328617	2	238173270	A	8	0	G	0.0003016
rs11688753	2	239109823	G	5	33	T	0.0002023
rs149600429	2	239768441	T	12	2	C	0.0001711
rs139338240	2	239768515	T	12	2	C	0.0001711
rs149017432	2	239769748	C	12	2	T	0.0001711

rs7582778	2	240350457	A	1	19	G	0.0008417
rs2019849	2	241048221	C	33	25	T	0.0002474
2:242437342	2	242437342	C	9	1	CTGTGACTCCCACCCTCTCC	0.0007194
rs12495985	3	13142638	C	16	7	T	0.0009539
rs3821605	3	13625471	C	3	26	T	0.0005074
rs35220159	3	13631296	G	2	26	A	0.0001452
rs17305345	3	14285488	T	1	23	C	0.0001244
3:15506747	3	15506747	ATTTATTTATTTAT	8	0	A	0.0003016
rs6794881	3	17901388	C	32	25	T	0.0004454
3:19098681	3	19098681	G	6	45	T	0.000001067
rs6777278	3	27663953	T	30	23	G	0.0007082
rs3846009	3	27668464	T	29	22	C	0.0007063
rs9861189	3	27673788	A	31	25	G	0.0008555
rs2642910	3	27676816	G	31	25	A	0.0008555
rs34839218	3	27676911	T	30	22	TA	0.0006925
rs2642911	3	27677178	G	31	25	T	0.0008555
rs2724544	3	27702766	C	29	22	T	0.0007063
rs7650346	3	27888690	T	11	2	C	0.0005111
3:28983432	3	28983432	G	29	20	T	0.000273
3:28983435	3	28983435	G	29	21	T	0.0005775
3:29970248	3	29970248	C	15	5	CCATAACATATTTGAACCAC	0.0003715
rs4420912	3	31571327	T	11	44	A	0.0007768
rs73824170	3	31575172	C	11	43	G	0.0008678
rs7610458	3	31582238	T	11	43	G	0.0008678
rs6763173	3	31584438	A	11	43	T	0.0008678
rs4356850	3	31590065	T	11	43	A	0.0008678
rs10222653	3	31606923	T	11	43	G	0.0008678
rs9833491	3	31612235	T	11	43	C	0.0008678
rs35838645	3	31612854	A	11	44	C	0.0007768
rs10662234	3	31613426	T	11	43	TATTA	0.0008678
rs9868487	3	31638797	G	11	43	A	0.0008678
rs9835139	3	31639335	A	11	47	T	0.0001991
rs11713598	3	31642037	A	11	43	T	0.0008678
rs4569693	3	31644397	G	11	43	T	0.0008678
rs7433833	3	31650288	C	11	43	T	0.0008678
rs4443213	3	31650500	A	11	43	T	0.0008678
rs7349468	3	31651335	G	11	43	A	0.0008678
rs9854442	3	31654038	A	11	43	G	0.0008678
rs13083410	3	31659754	T	11	43	C	0.0008678
rs12629516	3	31682527	T	11	43	C	0.0008678
rs9875317	3	31685398	T	11	44	C	0.0007768

rs12172958	3	31691100	G	11	44	A	0.0007768
rs12630628	3	31694718	C	11	44	G	0.0007768
rs79723395	3	31954275	T	0	15	C	0.0005628
rs76226662	3	36884315	T	10	1	C	0.0002681
rs62245253	3	36898036	A	10	1	G	0.0002681
rs62245258	3	36932602	A	10	1	G	0.0002681
rs113568999	3	40943865	AT	7	0	A	0.0008679
rs60041886	3	40944219	T	7	0	C	0.0008679
3:40946542	3	40946542	G	7	0	A	0.0008679
rs143825656	3	40947151	T	7	0	C	0.0008679
3:40948039	3	40948039	T	7	0	C	0.0008679
rs6793742	3	40949548	C	7	0	T	0.0008679
rs6599126	3	40954063	G	7	0	A	0.0008679
rs72859818	3	40957804	A	7	0	G	0.0008679
rs11929001	3	40977639	G	7	0	A	0.0008679
rs10865897	3	41017070	G	7	0	A	0.0008679
rs342539	3	42510528	A	1	23	G	0.0001158
rs140056071	3	45359850	C	10	1	G	0.0002681
rs61007059	3	45361210	CG	10	1	C	0.0002681
rs150045682	3	45361953	C	10	1	CTTT	0.0002681
rs2581792	3	53035044	C	26	17	T	0.0007398
rs73077837	3	53042074	T	12	2	C	0.0001279
3:53138402	3	53138402	CT	10	1	C	0.0002681
rs56125165	3	53317098	T	17	7	C	0.0004382
rs17062350	3	60128846	T	6	32	C	0.00098
rs35022605	3	60130882	A	6	33	AT	0.0009098
rs62240253	3	60132040	T	6	33	C	0.0009098
rs12632865	3	60132390	T	6	33	C	0.0009098
rs11130756	3	60132658	A	6	32	G	0.00098
rs4679639	3	60134540	C	6	32	A	0.00098
rs11709255	3	60137556	T	6	32	A	0.00098
3:65023533	3	65023533	CTGTGTGTGTGTG	9	0	C	0.0001034
rs4688537	3	65044903	A	13	3	G	0.0002409
rs17072462	3	65151691	C	19	9	G	0.0004395
rs17072464	3	65151812	G	19	9	T	0.0004395
rs9876289	3	66931371	G	9	1	A	0.0007194
rs9857208	3	66964812	A	9	1	C	0.0007194
rs13065059	3	67031250	A	9	1	G	0.0007194
rs4855329	3	68353494	T	2	27	C	0.000073
rs4345072	3	68353956	C	2	27	A	0.000073
rs6549115	3	68355872	A	2	27	T	0.000073

rs9822608	3	68357852	T	2	27	G	0.000073
rs57935782	3	68360456	T	2	27	TGTCATATA	0.000073
rs59158231	3	68360460	A	2	27	ACGTT	0.000073
rs2131042	3	68361179	G	2	27	A	0.000073
rs4378971	3	68363844	A	2	27	T	0.000073
rs13097366	3	68367425	A	11	44	T	0.0007768
rs10155009	3	68368649	G	4	31	A	0.0001398
rs1491757	3	68369111	G	4	31	T	0.0001398
rs1491756	3	68369288	G	4	31	A	0.0001398
rs1491755	3	68369391	G	4	31	A	0.0001398
rs1388506	3	68372549	G	9	44	A	0.0000882
rs11274211	3	68373386	TGCCTTGTTGATA	9	44	T	0.0000882
rs6549116	3	68373756	T	4	29	C	0.0004463
rs6770136	3	68374110	A	4	29	G	0.0004463
rs34315106	3	68374822	C	9	44	T	0.0000882
rs1491743	3	68374909	C	4	29	T	0.0004463
rs1491745	3	68379169	A	9	42	G	0.0003081
rs1491746	3	68379240	G	7	37	C	0.0003628
rs2313283	3	68380055	C	7	37	A	0.0003628
rs5849825	3	68380059	G	7	37	GT	0.0003628
rs3033741	3	68380202	C	7	37	CTGA	0.0003628
rs3033745	3	68380268	A	7	37	AAGTACT	0.0003628
rs6763500	3	68381076	T	6	34	C	0.0004884
rs6763516	3	68381106	T	6	34	C	0.0005004
rs1552710	3	68386395	A	12	46	G	0.0005428
rs1552711	3	68386397	G	12	46	C	0.0005428
rs1388500	3	68388365	G	15	52	C	0.0006042
rs262250	3	68403593	C	15	51	G	0.0006974
rs262248	3	68405259	T	16	53	C	0.0006806
rs171892	3	68430620	C	16	54	T	0.0003907
rs17831048	3	68475353	C	29	22	T	0.0007063
rs113071699	3	70386328	T	27	17	TTAA	0.000186
rs12491962	3	70389633	C	26	18	A	0.0007809
rs11715008	3	72674056	A	24	15	G	0.0005235
rs516826	3	73862619	A	19	7	C	0.00008788
rs35869217	3	73862785	AG	19	7	A	0.00008788
rs291532	3	73864474	A	19	7	T	0.00008788
rs291530	3	73866394	G	19	7	C	0.00008788
rs17028298	3	73867865	T	5	31	C	0.0006549
rs548611	3	73868089	T	19	7	C	0.00008788
rs633118	3	73868891	G	19	7	A	0.00008788

rs634465	3	73869231	T	19	7	C	0.00008788
rs514711	3	73869503	T	18	6	C	0.0001281
rs492878	3	73869545	T	19	7	C	0.00008788
rs9848344	3	73869793	G	33	27	A	0.0005258
rs291509	3	73870186	T	19	7	C	0.00008788
rs291508	3	73870544	A	19	7	G	0.00008788
rs2323170	3	73871068	G	33	27	C	0.0005258
rs291507	3	73871691	G	18	7	C	0.0001975
rs167097	3	73872340	C	19	7	G	0.00008788
rs7649822	3	73872496	G	20	11	A	0.0008202
rs291505	3	73873620	T	19	7	C	0.00008788
rs167096	3	73874699	T	19	7	G	0.00008788
rs291503	3	73874777	C	19	7	T	0.00008788
rs1107768	3	73876725	T	19	7	C	0.00008788
rs9784332	3	77106847	G	3	26	A	0.0005074
rs13060873	3	79768662	A	30	23	G	0.0007082
rs34322553	3	80115330	G	24	15	T	0.0005235
rs34957999	3	80115332	T	24	15	G	0.0005235
3:81054639	3	81054639	T	14	2	TCTC	0.00002365
rs11712051	3	81289857	A	7	35	G	0.0007198
rs10568741	3	84322697	CAT	16	7	C	0.0009539
rs79693379	3	87006367	G	7	0	A	0.0008679
rs764684	3	100530856	C	11	44	T	0.0007768
rs7612334	3	100532269	A	11	43	G	0.0008678
rs9879088	3	100537896	A	9	40	G	0.000591
rs7634500	3	100539676	C	9	40	G	0.000591
3:100562246	3	100562246	G	20	10	GT	0.0005977
3:100562247	3	100562247	G	20	10	T	0.0005977
rs1445076	3	106191942	C	13	48	T	0.0005647
3:106522026	3	106522026	A	11	43	T	0.0008678
rs11921731	3	115389796	A	24	15	G	0.0005235
rs3732624	3	115394383	T	24	15	C	0.0005235
rs9631453	3	115394563	C	24	12	G	0.0001247
rs3772920	3	115397299	G	18	9	A	0.0009279
rs12634436	3	115402685	T	18	9	A	0.0009279
rs3772926	3	115405505	C	18	9	T	0.0009279
rs111879237	3	115406999	A	18	9	G	0.0009279
rs6777348	3	117137202	T	18	9	C	0.0009279
rs4632551	3	117148573	C	18	9	G	0.0009279
3:124386900	3	124386900	A	0	19	C	0.00006755
3:128495792	3	128495792	G	36	31	C	0.0001448

rs201011313	3	130973927	G	30	24	A	0.0007114
rs201645765	3	130973931	G	25	14	A	0.0002032
3:130973932	3	130973932	A	16	7	G	0.0009539
rs275688	3	148347114	T	7	0	A	0.0008679
rs275687	3	148347582	C	7	0	A	0.0008679
rs275686	3	148347714	C	7	0	G	0.0008679
rs275684	3	148347952	T	7	0	C	0.0008679
rs275683	3	148347959	G	7	0	A	0.0008679
rs649342	3	148350663	T	7	0	A	0.0008679
rs444752	3	148351096	A	7	0	C	0.0008679
rs413671	3	148351517	T	7	0	A	0.0008679
rs387951	3	148351790	A	7	0	G	0.0008679
rs375839	3	148351839	G	7	0	A	0.0008679
rs201943	3	148352362	C	7	0	T	0.0008679
rs275695	3	148352651	T	7	0	A	0.0008679
rs4058143	3	148352896	CAATT	7	0	C	0.0008679
rs275696	3	148353206	G	7	0	A	0.0008679
rs275697	3	148353740	C	7	0	T	0.0008679
rs275698	3	148353866	T	7	0	C	0.0008679
rs200840889	3	148543836	T	22	13	TA	0.0006749
rs200542983	3	148543842	T	22	13	TGAATG	0.0006749
rs189278664	3	150440484	A	8	0	T	0.0003372
rs201510360	3	151265219	T	18	8	C	0.0005897
rs62275102	3	152825408	C	12	2	T	0.0001711
rs11323788	3	152826132	G	12	2	GA	0.0001711
rs17468698	3	152831259	A	12	2	G	0.0001711
rs111530192	3	152831858	A	12	2	G	0.0001711
rs56195773	3	152833232	T	12	2	C	0.0001711
rs56107526	3	152833810	A	12	2	G	0.0001711
rs894802	3	152878719	C	13	4	A	0.000883
rs894801	3	152878838	A	13	4	C	0.000883
rs61252895	3	152882810	G	13	4	A	0.000883
rs6809490	3	152885818	A	13	4	G	0.000883
rs59172775	3	152902599	T	13	4	C	0.000883
rs73163278	3	152906063	A	13	4	G	0.000883
rs55748959	3	152912196	G	13	4	A	0.000883
rs822719	3	165070685	T	10	42	C	0.0006808
rs7648573	3	165080780	G	10	42	A	0.0006808
rs9839242	3	165116509	T	9	40	C	0.000591
rs137947271	3	165117360	C	0	20	CT	0.0000355
rs1404782	3	165118985	A	9	40	G	0.000591

rs6444805	3	165121123	A	9	40	G	0.000591
rs9878522	3	165125159	C	2	22	T	0.0009583
rs9859645	3	165125655	A	2	22	G	0.0009583
rs6776072	3	165126317	C	0	20	T	0.0000355
rs9864667	3	165126682	T	2	22	C	0.0009583
rs9869260	3	165127088	A	2	22	G	0.0009583
rs4858780	3	165127614	A	0	20	T	0.0000355
rs9874980	3	165128475	G	2	22	C	0.0009583
rs9879604	3	165129027	A	0	20	C	0.0000355
rs10936444	3	165129566	A	2	22	T	0.0009583
rs11923404	3	165131390	G	0	19	A	0.00006755
rs2312406	3	165132797	T	0	17	C	0.0002606
rs6764581	3	165133245	A	0	17	G	0.0002606
rs60566962	3	165134328	T	0	17	C	0.0002606
rs58212043	3	165135759	CA	0	17	C	0.0002606
rs111463002	3	165138293	C	0	17	T	0.0002606
rs9862194	3	165138422	G	0	17	A	0.0002606
rs7626548	3	165138597	G	0	17	A	0.0002606
rs1010211	3	171131640	T	13	4	C	0.000883
3:177943093	3	177943093	A	2	22	C	0.0009583
3:178546724	3	178546724	A	16	6	T	0.0002677
rs9290671	3	178568129	C	33	29	T	0.0009301
rs2119898	3	179439466	T	37	34	C	0.0006983
rs2165679	3	179439567	A	37	34	G	0.0006983
rs35593662	3	179440107	G	37	34	C	0.0006983
rs1966757	3	179442279	T	37	34	G	0.0006983
rs2139561	3	183653254	T	27	19	C	0.0008555
rs2139559	3	183653937	T	27	19	G	0.0008555
rs3749441	3	183660806	G	27	19	C	0.0008555
rs1464322	3	183666862	A	26	18	G	0.0007809
rs4148586	3	183669396	T	27	19	C	0.0008555
rs56343575	3	183670633	T	27	19	C	0.0008555
rs3817404	3	183670672	A	27	19	C	0.0008555
rs4912517	3	183671340	T	27	19	G	0.0008555
rs68153167	3	183672846	T	27	19	C	0.0008555
rs6443925	3	183679754	C	27	18	G	0.0004001
rs2271936	3	183682695	C	27	19	T	0.0008555
rs4148581	3	183685165	C	27	19	T	0.0008555
rs7637515	3	183694215	A	27	19	T	0.0008555
rs3792582	3	183694602	G	27	19	A	0.0008555
rs66605623	3	183697628	A	27	19	G	0.0008555

rs4912471	3	183698341	T	27	19	C	0.0008555
rs12185910	3	183698626	A	27	19	G	0.0008555
rs2872247	3	183699781	C	27	19	A	0.0008555
rs55714546	3	183711150	C	27	19	T	0.0008555
rs66850130	3	183711628	T	27	19	C	0.0008555
rs55905394	3	183722364	A	27	19	T	0.0008555
rs55982636	3	183722417	C	27	19	T	0.0008555
rs112396093	3	183724974	C	27	19	A	0.0008555
rs55846170	3	183737498	G	27	19	A	0.0008555
rs55913043	3	183737667	T	27	19	C	0.0008555
rs56148005	3	183738579	A	27	19	G	0.0008555
rs66765300	3	183743778	A	27	19	G	0.0008555
rs939334	3	183749731	C	27	18	T	0.0004001
rs2014046	3	183749937	A	27	18	G	0.0004001
rs2090720	3	183750005	C	27	18	T	0.0004001
rs55953689	3	183756897	G	27	17	A	0.000186
rs9647371	3	184284279	T	13	4	C	0.000883
rs7644377	3	184295316	T	16	5	C	0.0001588
rs6786200	3	184311889	G	9	1	T	0.0007194
rs17799372	3	187592723	G	7	0	C	0.0008679
rs377395085	3	187609123	A	7	0	C	0.0008679
3:187609127	3	187609127	G	7	0	C	0.0008679
rs9880944	3	197647970	A	0	15	C	0.0005628
rs113481869	3	197647973	T	0	15	C	0.0005628
rs111656082	3	197647976	G	0	15	A	0.0005628
rs2164049	4	4175554	T	33	26	C	0.0002784
rs12501349	4	4175868	C	9	1	T	0.0007194
rs12498912	4	4175887	T	9	1	C	0.0007194
rs4689202	4	4175894	G	25	11	A	0.00001926
rs34485304	4	4175938	A	9	39	G	0.000596
rs12501414	4	4176067	C	9	1	T	0.0007194
rs2083724	4	4190942	G	16	6	A	0.0005436
rs3097979	4	4201105	C	31	23	A	0.0003729
rs3097980	4	4201116	G	31	23	T	0.0003729
rs2980129	4	4201297	G	30	23	A	0.0007082
rs2916416	4	4201342	G	30	23	A	0.0007082
rs2980131	4	4202350	A	31	24	C	0.0004543
rs2916417	4	4202389	A	29	23	T	0.0006489
rs2916422	4	4206953	A	12	47	G	0.0004795
rs7666381	4	4210460	C	12	46	G	0.0005428
rs11725336	4	4218669	T	15	54	C	0.0001803

rs28633695	4	4220375	C	36	29	G	0.0001509
rs4689930	4	4756636	T	13	4	C	0.000883
rs739778	4	7703265	A	31	24	G	0.0004543
rs2865050	4	9546117	G	0	15	C	0.0005628
rs9715737	4	9546122	A	0	15	G	0.0005628
rs6843276	4	9714147	C	12	45	T	0.0009266
4:12151990	4	12151990	G	20	10	GT	0.0005561
4:12151991	4	12151991	G	20	10	T	0.0005346
rs12640981	4	14764548	T	14	5	C	0.0008508
rs35371493	4	14769427	A	14	5	G	0.0008508
rs55770230	4	14770760	C	14	5	A	0.0008508
rs56365123	4	14771921	T	14	5	C	0.0008508
rs35340808	4	14784441	C	14	5	T	0.0008508
rs59167658	4	14798590	G	14	5	A	0.0008508
rs4554067	4	14798707	T	16	7	C	0.0009539
4:19690823	4	19690823	A	8	38	T	0.0004405
rs58918138	4	20353473	T	1	20	G	0.0004403
rs77041696	4	20358927	G	1	20	C	0.0004403
rs4552454	4	20386044	A	1	20	C	0.0004403
rs10027333	4	20390198	G	1	20	A	0.0004403
rs7435615	4	20399168	A	1	20	G	0.0004403
rs57411082	4	20408564	C	1	20	CTG	0.0004403
rs10026931	4	20430582	G	1	19	C	0.0008417
rs11728803	4	20851589	T	1	19	C	0.0008417
rs71940315	4	20865321	C	1	19	CTT	0.0008417
rs759245	4	25058232	G	15	51	A	0.0006974
rs759244	4	25058323	A	15	51	T	0.0006974
rs6821216	4	25058776	G	15	51	C	0.0006974
rs6835786	4	25061194	A	15	51	C	0.0006974
rs17408391	4	25101584	C	4	29	G	0.0004463
rs80196582	4	25889810	C	7	0	A	0.0008679
rs17499919	4	38461337	C	11	47	T	0.0001343
4:40024963	4	40024963	CGAG	26	17	C	0.0003136
4:40024966	4	40024966	G	24	15	GGCA	0.0003499
4:47142112	4	47142112	A	13	50	C	0.0002818
4:49209131	4	49209131	C	17	7	T	0.0004382
rs7441960	4	57542238	A	31	25	G	0.0008555
rs11721544	4	57542299	T	30	23	C	0.0007082
rs12642850	4	57543677	G	30	23	A	0.0007082
rs11945101	4	57544353	C	30	23	T	0.0007082
rs72636326	4	58791168	G	8	0	A	0.0003016

rs72636328	4	58795455	T	8	0	C	0.0003016
4:58987446	4	58987446	G	8	0	T	0.0003776
rs145721254	4	60398515	T	12	50	A	0.00007817
rs2054799	4	60437170	T	7	0	G	0.0008679
rs79602894	4	61920822	G	1	19	T	0.0008417
rs76948623	4	61921038	T	1	19	G	0.0008417
rs74899240	4	61922855	G	1	19	C	0.0008417
rs79108432	4	61923606	G	1	18	T	0.0008528
rs2341739	4	61927052	T	1	19	A	0.0008417
4:66069285	4	66069285	AC	14	3	A	0.00009441
rs11933083	4	68114997	G	14	4	A	0.0003939
rs6534012	4	75547732	A	30	23	C	0.0007082
rs13124944	4	75547845	A	26	16	C	0.0002858
rs72653702	4	76999523	A	3	25	G	0.0009258
4:82703972	4	82703972	G	8	0	C	0.0003016
rs10016545	4	84578892	G	30	23	A	0.0007082
rs6821732	4	84580093	T	30	23	C	0.0007082
rs7694734	4	84580763	C	30	23	A	0.0007082
rs7671912	4	84581271	T	30	23	C	0.0007082
rs2868797	4	84582260	G	30	23	A	0.0007082
rs6822897	4	84582536	C	30	23	T	0.0007082
rs1563696	4	84583876	T	31	23	C	0.0003729
rs57980547	4	84600736	G	24	15	T	0.0005235
rs6535496	4	84601526	G	32	25	A	0.0004454
rs72659283	4	84907590	T	11	2	C	0.0004434
rs2135284	4	84908159	G	11	2	A	0.0004434
rs72659284	4	84910400	G	11	2	A	0.0004434
rs72659286	4	84916214	G	11	2	T	0.0004434
rs72659295	4	84934184	A	11	2	G	0.0004434
rs74476563	4	84935594	GT	11	2	G	0.0004434
rs72659296	4	84936325	T	11	2	G	0.0004434
rs72659297	4	84938960	C	11	2	G	0.0004434
rs4129091	4	84941953	A	11	2	G	0.0004434
rs113134562	4	84951643	T	11	2	C	0.0004434
rs17007881	4	84953207	G	13	2	A	0.00006438
rs1921883	4	84953383	C	13	2	T	0.00006438
rs72660815	4	84954205	G	13	2	T	0.00006438
rs72660816	4	84954602	C	13	2	A	0.00006438
rs72660817	4	84955535	G	13	2	A	0.00006438
rs72660819	4	84956002	C	13	2	A	0.00006438
rs1317020	4	84956277	T	13	2	C	0.00006438

rs56004187	4	84956736	C	13	2	T	0.00006438
rs1921880	4	84957198	T	13	2	G	0.00006438
rs1317519	4	84957652	T	13	2	C	0.00006438
rs72660826	4	84965875	G	13	3	A	0.0002409
rs74671679	4	84967393	G	13	4	GA	0.000883
rs56364031	4	84968393	T	13	4	A	0.000883
rs72660828	4	84969826	T	13	4	C	0.000883
rs72660829	4	84970036	G	13	4	A	0.000883
rs72660830	4	84972404	A	13	4	G	0.000883
rs72660831	4	84972436	T	13	4	C	0.000883
rs11097378	4	94548777	T	12	45	G	0.0009266
rs3864152	4	94553780	A	12	46	G	0.0005428
rs11932367	4	94557555	A	12	46	G	0.0005428
rs56210124	4	96672725	G	16	5	C	0.0001588
rs146016883	4	96673449	A	16	5	G	0.0001588
rs2043658	4	96677620	C	16	5	G	0.0001588
rs144043778	4	96721581	CT	16	5	C	0.0001588
rs1160489	4	99773191	T	10	1	G	0.0002681
rs7665186	4	99775406	G	9	1	A	0.0007194
rs961757	4	99779875	C	9	1	G	0.0007194
rs6532769	4	99780391	C	9	1	T	0.0007194
rs7677046	4	99781025	T	9	1	C	0.0007194
4:105051472	4	105051472	C	0	19	T	0.0001516
4:109041155	4	109041155	T	6	35	G	0.0002764
rs35828847	4	111211729	CT	24	16	C	0.0009443
rs12510061	4	111214726	G	30	22	A	0.0003565
rs7682430	4	111219351	G	30	22	T	0.0003565
rs11279781	4	111235243	G	22	13	GTTATTATA	0.0006749
rs62336634	4	111236872	A	22	13	G	0.0006749
rs11098151	4	113255531	A	27	20	G	0.0008358
rs11936170	4	119464069	G	22	13	A	0.0006749
rs111866828	4	120124537	A	13	4	G	0.000883
rs34350503	4	123827911	A	22	13	G	0.0006749
rs12513181	4	123835656	C	22	13	A	0.0006749
rs12648093	4	123838758	A	22	13	G	0.0006749
rs62325775	4	123947268	A	20	10	G	0.0005561
rs1506161	4	126766315	T	31	25	C	0.0008555
rs13119203	4	130194362	A	33	27	G	0.0005258
4:139684634	4	139684634	T	7	0	TAGATAGATAGATAGATAG	0.0008679
rs13108250	4	145234989	T	3	27	G	0.0002808
rs2719341	4	145263286	T	7	36	G	0.0006639

rs2719335	4	145289459	C	7	36	T	0.0006639
rs2657795	4	145350264	A	6	36	C	0.0001537
rs112587461	4	145379968	T	4	27	G	0.000811
4:153056425	4	153056425	A	0	16	G	0.0002757
rs10002011	4	154094675	A	17	56	G	0.0003692
rs10001881	4	154094682	T	17	56	C	0.0003692
rs142792673	4	154096164	GTTTTGT	35	33	G	0.0004561
rs3804100	4	154625409	C	9	1	T	0.0007194
rs111905892	4	154628613	A	9	1	G	0.0007194
rs2606322	4	154643222	G	7	0	T	0.0008679
rs1869947	4	154643503	G	7	0	A	0.0008679
rs4145338	4	154654323	C	7	0	T	0.0008679
rs10434139	4	154654665	A	7	0	G	0.0008679
rs56256921	4	154655929	A	7	0	G	0.0008679
rs11931479	4	154664523	G	7	0	A	0.0008679
rs17030417	4	154670813	G	7	0	A	0.0008679
rs11946635	4	154674080	A	7	0	G	0.0008679
rs11946638	4	154674095	T	7	0	G	0.0008679
rs57666128	4	154674842	A	7	0	T	0.0008679
4:155246471	4	155246471	A	0	18	G	0.0001312
4:155246473	4	155246473	A	0	21	G	0.00003544
4:161072770	4	161072770	G	4	28	A	0.0008078
rs374300171	4	161074637	C	2	22	T	0.0009583
4:163515735	4	163515735	A	1	20	AAATAAATAAATAAAT	0.0004403
rs9995462	4	163515736	T	2	23	A	0.0005167
rs200385614	4	164804411	A	7	35	G	0.0007198
rs200908737	4	164804412	T	7	35	A	0.0007198
4:164896322	4	164896322	T	35	30	G	0.0006538
rs1246927	4	166543852	C	28	17	T	0.0000884
rs78218057	4	172125931	A	7	0	G	0.0008679
rs901236	4	172740734	T	12	47	G	0.0004795
rs4396959	4	172743234	G	19	58	A	0.0006983
rs2716789	4	180503663	T	31	24	C	0.0004543
rs7696678	4	189015024	T	10	42	C	0.0006808
rs72725404	4	189965620	A	0	17	G	0.0002606
rs62340474	4	189975229	T	1	21	C	0.0002339
rs34465723	4	190008501	A	33	26	G	0.0002784
rs2101911	4	190009975	T	33	26	C	0.0002784
rs62340525	4	190010558	T	32	26	C	0.0008799
rs6848118	4	190014350	C	32	26	T	0.0008799
rs6848314	4	190014460	C	32	26	T	0.0008799

rs34069301	4	190015739	AG	32	26	A	0.0008799
rs36108077	4	190017202	T	31	25	C	0.0008555
rs6829594	4	190029232	T	0	15	C	0.0005628
rs116695447	4	190128855	C	9	1	G	0.0007194
rs35269155	5	2077306	G	14	52	GC	0.0001845
rs11741532	5	2077318	G	14	52	A	0.0001845
rs35028140	5	2077343	G	13	50	GAA	0.0002818
rs11133927	5	2077356	T	13	50	G	0.0002818
rs10866530	5	2077580	T	13	50	C	0.0002818
rs10866531	5	2077739	T	13	50	G	0.0002818
rs4246755	5	2078006	T	15	53	G	0.0003298
rs10062706	5	2091502	C	13	48	G	0.0005647
rs6891887	5	2092044	G	13	48	T	0.0005647
rs6893206	5	2092045	C	13	48	T	0.0005647
rs460672	5	2355032	G	16	4	C	0.00007821
rs462906	5	2355389	T	15	4	C	0.000175
rs458006	5	2355587	A	14	4	C	0.0003939
rs113602711	5	2355658	T	15	4	TATGCTAATGTCAGAAAGA	0.000175
rs58937979	5	2355682	T	15	4	C	0.000175
rs2641215	5	2355878	T	15	4	G	0.000175
rs456732	5	2358645	G	15	4	A	0.000175
rs465527	5	2359450	A	13	3	G	0.0002409
rs2628160	5	2361180	T	13	3	G	0.0002409
5:2781841	5	2781841	T	6	36	TTC	0.0001537
rs200556114	5	2781844	A	6	36	ATCCAG	0.0001537
rs34866103	5	2782407	GA	6	36	G	0.0001537
rs11952868	5	2783714	C	6	36	T	0.0001537
rs11743697	5	2784489	A	6	36	T	0.0001537
rs1118651	5	2786321	T	6	37	A	0.00008623
rs78185201	5	2786669	T	9	1	C	0.0007194
rs34196947	5	2786964	T	6	37	C	0.00008623
rs34195323	5	2787379	C	6	37	CT	0.00008623
rs35813681	5	2787631	A	6	36	T	0.0001537
rs11133961	5	2788119	T	6	36	G	0.0001537
rs1106547	5	2788832	A	6	36	G	0.0001537
rs7702905	5	2788906	A	6	36	G	0.0001537
rs35681818	5	2791777	T	6	36	C	0.0001537
rs10069464	5	2792395	G	6	36	T	0.0001537
rs7729432	5	2793841	T	6	36	G	0.0001537
rs13180135	5	2798572	A	5	37	G	0.00005136
rs12374492	5	2798741	T	6	36	C	0.0001537

rs148802928	5	2800384	C	9	1	CCT	0.0007194
rs3822432	5	6609112	G	40	37	A	0.0003306
rs3756429	5	6613146	A	34	29	G	0.0006139
rs3776436	5	6614963	G	34	29	A	0.0006139
rs3776435	5	6614969	A	34	29	G	0.0006139
rs2288443	5	6619733	C	32	26	T	0.0008799
rs2288446	5	6620459	A	32	26	G	0.0008799
5:8922720	5	8922720	T	12	47	C	0.0004795
rs10547411	5	9384785	G	9	1	GAT	0.000839
rs4425512	5	18954896	A	27	17	G	0.000186
rs4447989	5	18959731	A	27	17	T	0.000186
rs10040022	5	18964842	G	27	17	A	0.000186
rs75322590	5	18970904	T	27	17	TG	0.000186
rs35800895	5	18970905	T	27	17	TATGTA	0.000186
5:18970906	5	18970906	C	27	17	CAACAAATGTACAA	0.000186
rs4597970	5	18971937	C	27	17	T	0.000186
rs10056516	5	18973089	C	27	18	A	0.0004001
rs10071196	5	18973115	G	27	18	A	0.0004001
rs142645628	5	18973588	T	28	17	TA	0.0000884
rs13155140	5	18973594	T	28	17	A	0.0000884
rs111273782	5	18974181	A	9	1	T	0.0007194
5:18974183	5	18974183	A	27	17	T	0.000186
5:18974185	5	18974185	A	27	18	T	0.0004001
5:18974187	5	18974187	A	27	18	T	0.0004001
rs4866133	5	18974981	T	27	17	G	0.000186
rs77430313	5	18976454	G	28	19	A	0.0002695
rs70950014	5	18976473	T	26	17	TAC	0.0002863
rs141348783	5	18976539	CAT	27	18	C	0.0004001
rs12109900	5	18976856	T	27	17	C	0.000186
rs12109901	5	18976857	T	27	17	C	0.000186
rs10037119	5	18985701	G	27	17	C	0.000186
rs11747928	5	18986545	C	27	17	T	0.000186
rs10036758	5	18987784	T	27	17	A	0.000186
rs35854198	5	18988665	GC	27	17	G	0.000186
rs13159552	5	18989020	C	27	17	T	0.000186
rs35204104	5	18989038	A	27	17	AT	0.000186
rs34575433	5	18989059	AGC	27	17	A	0.000186
rs7713168	5	18990117	C	27	17	A	0.000186
rs10940990	5	18990844	A	27	17	G	0.000186
rs4263496	5	18994108	G	27	17	T	0.000186
rs4510591	5	18994392	A	27	17	G	0.000186

rs7730213	5	18994462	C	27	17	T	0.000186
rs10064363	5	18997081	C	27	17	T	0.000186
rs33915140	5	18997166	G	27	18	GA	0.0004001
rs9292456	5	19001097	C	27	17	T	0.000186
rs5866354	5	19004633	A	27	17	AT	0.000186
rs4537064	5	19011415	A	27	17	C	0.000186
rs7731960	5	19011714	T	27	17	C	0.000186
rs111988657	5	19100793	A	16	6	T	0.0005436
rs77644860	5	19102912	T	16	6	TC	0.0005436
rs78836818	5	19105611	T	16	6	C	0.0005436
rs11441553	5	19106020	GT	16	6	G	0.0005436
rs200889621	5	19109088	CT	15	4	C	0.0001909
rs79882907	5	19109585	G	16	6	A	0.0005436
rs76054915	5	19110676	TG	16	6	T	0.0005436
rs78954403	5	19111751	T	16	6	TA	0.0005436
rs7442872	5	19112897	T	16	6	C	0.0005436
rs73759603	5	19114103	T	16	6	A	0.0005436
rs73759606	5	19115514	G	16	6	T	0.0005436
rs59439678	5	19118412	A	16	6	G	0.0005436
rs74624881	5	19118807	G	16	6	A	0.0005436
rs76198877	5	19120349	G	16	6	T	0.0005436
rs11956374	5	19120681	T	16	6	C	0.0005436
rs11960467	5	19120884	C	16	6	T	0.0005436
rs36114787	5	19121081	C	16	6	CG	0.0005436
rs73759608	5	19121365	G	16	6	A	0.0005436
rs76591709	5	19121718	T	16	6	C	0.0005436
rs59487235	5	19123299	G	16	6	A	0.0005436
rs57533008	5	19125248	G	16	6	A	0.0005436
rs79052914	5	19127280	T	16	6	C	0.0005436
rs11950664	5	19148922	T	16	6	C	0.0005436
rs202053412	5	19162349	G	16	6	GA	0.0005436
rs200432192	5	19163455	T	15	5	C	0.0003715
rs192918603	5	19166903	G	20	11	T	0.0008202
rs10941087	5	19171347	A	20	11	G	0.0008202
rs4546383	5	19174117	T	20	11	G	0.0008202
rs56127401	5	19175816	G	20	11	A	0.0008202
rs11954805	5	19179319	C	20	11	T	0.0008202
rs67595693	5	19181373	C	20	11	T	0.0008202
rs56010419	5	19181660	C	20	11	T	0.0008202
rs28753870	5	19183204	G	20	11	T	0.0008202
rs10461927	5	19183422	C	20	11	T	0.0008202

rs67749574	5	19184078	C	20	11	T	0.0008202
rs4492113	5	19185179	C	20	11	A	0.0008202
rs10461707	5	19185427	T	20	11	A	0.0008202
rs10055960	5	19185900	A	20	11	G	0.0008202
rs10056045	5	19186087	C	20	11	G	0.0008202
rs35630947	5	19186390	G	20	11	GA	0.0008202
rs4409093	5	19186620	T	20	11	C	0.0008202
rs59242851	5	19186881	TA	16	6	T	0.0005436
rs10073850	5	19187322	T	20	11	C	0.0008202
rs143565175	5	19187627	G	20	11	C	0.0008202
rs1512548	5	19399602	T	16	5	C	0.0001588
rs2137386	5	19413622	C	17	6	T	0.0002592
rs3032401	5	19413874	T	17	6	TAA	0.0002592
rs2137389	5	19417376	A	17	6	G	0.0002592
rs76966134	5	24866179	A	7	0	G	0.0008679
rs77157809	5	24866294	T	7	0	C	0.0008679
rs74730385	5	24867181	A	7	0	G	0.0008679
rs12513781	5	25620280	A	8	37	G	0.0008686
rs66820692	5	28635368	T	31	24	A	0.0004543
rs6450656	5	28648576	A	17	59	C	0.0000866
rs7722988	5	28648707	T	17	59	C	0.0000866
rs7736782	5	28656324	A	15	51	G	0.0006974
rs11347966	5	37158892	A	8	0	AT	0.0003372
rs76348379	5	37901402	T	8	0	C	0.0003016
rs76564102	5	37903568	A	7	0	C	0.0008679
rs3836796	5	39289543	GT	11	42	G	0.0007112
rs34525658	5	39427198	A	21	9	C	0.00009415
rs35950587	5	39429785	A	20	10	C	0.0005561
rs34209642	5	39430941	A	20	10	G	0.0005561
rs6877573	5	39432245	C	20	10	G	0.0005561
rs7703879	5	39434349	A	20	10	G	0.0005561
rs76402156	5	42917370	A	16	7	G	0.0009539
rs10066777	5	59174758	G	9	1	C	0.000839
rs346406	5	62490220	G	16	5	A	0.0001588
rs346407	5	62491973	T	14	4	C	0.0003939
rs346408	5	62493050	C	14	4	T	0.0003939
rs346403	5	62493840	T	14	4	C	0.0003939
rs186968	5	62498557	C	16	5	T	0.0001588
rs455779	5	62500313	A	14	4	G	0.0003939
rs346409	5	62508981	C	14	4	G	0.0003939
rs164103	5	62512669	G	14	4	A	0.0003939

rs346405	5	62513738	C	14	4	A	0.0003939
rs347669	5	62523431	G	14	4	A	0.0003939
rs347670	5	62526755	C	14	4	T	0.0003939
rs164105	5	62528445	A	14	4	G	0.0003939
rs346420	5	62529212	G	14	4	T	0.0003939
rs346421	5	62529983	T	14	4	G	0.0003939
rs346423	5	62532892	G	14	4	A	0.0003939
5:63924273	5	63924273	GA	10	2	G	0.0009067
rs62360705	5	71028274	A	7	0	G	0.0008679
rs60286869	5	71028824	T	7	0	C	0.0008679
rs62360707	5	71029427	T	7	0	C	0.0008679
rs4057259	5	71029730	C	7	0	G	0.0008679
rs4057258	5	71029743	C	7	0	T	0.0008679
rs3857386	5	71029835	T	7	0	TCTC	0.0008679
rs78111196	5	71030187	G	7	0	A	0.0008679
rs201230349	5	71033734	AT	7	0	A	0.0008679
rs77510257	5	73708375	A	7	0	T	0.0008679
rs59857064	5	73735191	T	9	1	A	0.0007194
rs76518990	5	73736642	G	8	0	A	0.0003016
rs7736553	5	73737072	C	8	0	A	0.0003016
rs17736697	5	73738367	C	8	0	T	0.0003016
rs74968908	5	73741967	G	8	0	A	0.0003016
rs78116060	5	73745351	A	8	0	G	0.0003016
rs79114589	5	73746799	T	8	0	G	0.0003016
rs76655003	5	73747050	G	8	0	A	0.0003016
rs77379635	5	73748608	T	8	0	C	0.0003016
rs75776784	5	73749080	C	7	0	G	0.0008679
rs1873365	5	73749892	G	8	0	A	0.0003016
rs1873364	5	73750044	C	8	0	T	0.0003016
rs7729784	5	73750685	A	8	0	G	0.0003016
rs144616208	5	73760442	AGAAAAGGTCGT	7	0	A	0.0008679
rs13156710	5	73760792	A	9	0	T	0.0001034
rs78691613	5	73760961	C	0	18	T	0.0001312
rs4621539	5	73767751	T	7	36	C	0.0006165
rs73113802	5	75515494	C	11	2	G	0.0004434
rs74616192	5	75515504	T	11	2	C	0.0004434
rs75055749	5	75515958	G	11	2	A	0.0004434
rs77096726	5	75517297	A	11	2	G	0.0004434
rs79502197	5	75519156	G	11	2	A	0.0004434
rs6884651	5	75538661	C	11	2	T	0.0004434
rs80286446	5	75538689	C	11	2	A	0.0004434

rs61497857	5	75538893	A	11	2	G	0.0004434
rs11740431	5	75540890	A	36	31	G	0.0003502
rs56264728	5	75541183	T	36	31	G	0.0003502
rs28587768	5	75541811	A	36	31	G	0.0003502
rs145224048	5	75544482	T	10	1	C	0.00021
rs1847060	5	75544538	G	36	32	A	0.0006397
rs6879020	5	75545035	G	36	32	A	0.0006397
rs11748793	5	75548176	T	36	31	C	0.0003502
rs10055247	5	75549475	G	36	29	A	0.0002973
rs10045181	5	75549745	A	36	30	C	0.000303
rs10040087	5	75550007	A	37	32	G	0.0003248
rs6862529	5	75551209	T	36	30	C	0.000303
rs55973414	5	75551315	G	39	32	A	0.00004458
rs10035663	5	75551739	G	11	2	C	0.0004434
rs6868455	5	76398623	T	4	28	C	0.0008078
rs13358038	5	76404051	A	4	28	G	0.0008078
rs4703726	5	76404238	A	4	28	G	0.0008078
rs4703727	5	76407577	C	4	28	T	0.0008078
rs4437352	5	76412745	T	12	45	C	0.0009266
rs10037303	5	76413897	C	6	33	T	0.0009098
rs2055432	5	78988769	A	15	51	C	0.0006974
rs116556111	5	85770112	C	7	0	T	0.0008679
rs16902245	5	85770686	C	7	0	T	0.0008679
rs10065803	5	85771235	G	7	0	A	0.0008679
rs13358838	5	85775350	C	7	0	T	0.0008679
rs73142510	5	85778295	T	7	0	G	0.0008679
rs73142511	5	85778425	G	7	0	T	0.0008679
rs73142516	5	85780087	T	7	0	C	0.0008679
rs74601594	5	85781523	C	7	0	T	0.0008679
5:85784243	5	85784243	GT	7	0	G	0.0008679
rs77942162	5	85791830	C	7	0	T	0.0008679
rs74616050	5	85792031	C	7	0	T	0.0008679
rs73142527	5	85792215	T	7	0	G	0.0008679
rs75482133	5	85792829	A	7	0	G	0.0008679
rs10053075	5	85793391	G	7	0	T	0.0008679
rs10038807	5	85793781	C	7	0	A	0.0008679
rs10057196	5	85795217	C	7	0	T	0.0008679
rs141369087	5	85795562	T	8	0	TGATA	0.0003016
rs75017161	5	85795906	G	7	0	T	0.0008679
rs73142537	5	85796600	G	7	0	A	0.0008679
rs78018415	5	85796652	A	7	0	AT	0.0008679

rs73142540	5	85796915	C	7	0	T	0.0008679
rs73142543	5	85797158	C	7	0	T	0.0008679
rs4920666	5	85801789	G	7	0	T	0.0008679
rs55687655	5	86972361	C	23	14	T	0.0007414
rs12186390	5	86998366	T	23	14	C	0.0007414
5:94344766	5	94344766	A	24	14	G	0.0004114
5:94344769	5	94344769	G	25	14	A	0.0002032
5:94344774	5	94344774	A	24	14	G	0.0004114
rs145531067	5	94761820	A	14	5	ATATATATG	0.0006534
rs6556941	5	96218598	T	7	0	C	0.0008679
5:96366336	5	96366336	C	9	1	G	0.0007194
rs114487483	5	99678415	C	6	34	T	0.0002428
rs2400601	5	100976362	T	7	0	G	0.0008679
rs62357464	5	106619434	C	16	6	G	0.000589
5:115814627	5	115814627	G	20	11	T	0.0008202
rs199731665	5	116877468	T	8	0	TACATCTACAC	0.0003776
rs199590016	5	116877483	G	8	0	A	0.0003372
rs36138399	5	117187732	C	3	24	G	0.0009363
rs4012925	5	118273667	T	7	0	TTTG	0.0008679
rs6898332	5	122648541	G	7	35	C	0.0007198
rs10900782	5	125403809	G	29	21	A	0.0005775
rs1421870	5	125405191	C	28	20	A	0.0005398
rs35006655	5	125406364	AATGTTTGC	28	20	A	0.0005398
rs13167197	5	125408090	C	29	20	T	0.0003112
rs10213871	5	125409767	G	28	20	A	0.0005398
rs6895992	5	125411032	T	28	20	A	0.0005398
rs10064839	5	125412411	G	28	20	A	0.0005398
rs10051723	5	125413290	A	28	20	G	0.0005398
rs10076671	5	125414225	T	28	20	C	0.0005398
rs3909303	5	125421873	T	22	13	C	0.0006749
rs6878605	5	125424091	G	21	12	A	0.0009318
rs3849073	5	125434497	A	28	20	G	0.0005398
rs10478682	5	125437788	C	28	20	A	0.0005398
rs5007293	5	125439855	C	28	20	A	0.0005398
rs1383555	5	125440053	C	28	20	T	0.0005398
rs1383556	5	125440179	G	28	20	C	0.0005398
rs12520267	5	125441239	T	28	20	A	0.0005398
rs12520368	5	125441739	T	28	20	C	0.0005398
rs13188435	5	125442155	C	28	20	T	0.0005398
rs5871191	5	125445019	G	28	20	GT	0.0005398
rs6861867	5	125451685	A	28	20	G	0.0005398

rs11241867	5	125458387	C	16	7	T	0.0009539
rs7728215	5	125461790	A	16	7	G	0.0009539
rs10054533	5	125462485	C	16	7	T	0.0009539
rs3849075	5	125466176	G	16	7	A	0.0009539
rs11739456	5	125467005	G	29	21	C	0.0005775
rs10463817	5	125471402	G	29	22	T	0.0007063
rs75509873	5	125475752	AG	30	23	A	0.0007082
rs10477666	5	125478358	C	29	22	A	0.0007063
rs374455672	5	125482719	ATAAAG	29	22	A	0.0007063
rs140272168	5	125486927	G	29	22	A	0.0007063
rs140084416	5	125487289	CTCTG	29	22	C	0.0007063
rs6888912	5	125489390	C	29	22	T	0.0007063
rs6870502	5	125489767	T	29	22	C	0.0007063
rs3843819	5	125496145	C	22	12	T	0.000477
rs3898923	5	125498708	C	25	16	T	0.0005585
rs3909804	5	125507018	A	23	14	C	0.0007414
rs10061705	5	125507903	T	23	14	C	0.0007414
rs10900783	5	125512816	T	23	14	C	0.0007414
rs2081831	5	125530928	C	16	7	A	0.0009539
rs7718533	5	125533158	T	23	14	G	0.0007414
rs13359614	5	125550942	C	24	14	T	0.0003806
rs115455679	5	125554997	T	2	22	C	0.0009583
rs35450346	5	125618844	C	24	15	CA	0.0009958
rs6878654	5	131627401	A	16	54	T	0.0003907
rs199725847	5	142367103	T	18	9	TA	0.0009279
rs245723	5	142367558	T	18	9	A	0.0009279
rs151975	5	142369812	A	18	9	G	0.0009279
rs143838874	5	142373382	TTTTTC	16	7	T	0.0009539
rs151972	5	142375982	G	18	9	T	0.0009279
rs13162212	5	142377013	A	19	9	G	0.0004395
rs7721905	5	142379626	G	19	9	A	0.0004395
rs728465	5	142384160	C	19	9	T	0.0004395
rs1438724	5	142385141	T	19	9	G	0.0004395
rs245848	5	142386578	C	18	9	T	0.0009279
rs4912888	5	142387103	G	19	9	A	0.0004395
rs3822398	5	142387961	C	19	9	T	0.0004395
rs10875598	5	142391158	C	19	9	T	0.0004395
rs3822397	5	142393562	G	19	9	A	0.0004395
rs11313040	5	142394088	A	18	9	AG	0.0009279
rs245813	5	142397025	T	18	9	A	0.0009279
rs245816	5	142398445	C	18	9	T	0.0009279

rs245817	5	142400297	A	18	9	G	0.0009279
rs245818	5	142400947	G	18	9	A	0.0009279
rs188696155	5	144093281	A	3	25	G	0.0009258
rs7700566	5	145734375	A	0	15	G	0.0005628
rs57060750	5	145735566	T	0	15	TA	0.0005628
rs17653203	5	148295775	G	14	5	C	0.0008508
rs353244	5	148826915	A	15	52	C	0.0006042
rs2748247	5	149794412	A	16	53	G	0.0006806
rs60404047	5	149850518	G	15	4	A	0.000175
rs73272621	5	149854482	G	14	4	A	0.0003939
rs79664277	5	149859889	A	16	4	G	0.00007821
rs73272643	5	149860190	G	16	4	A	0.00007821
rs75528239	5	149862325	G	13	4	A	0.000883
rs76358617	5	149862396	T	13	4	C	0.000883
rs73272649	5	149862885	T	16	4	C	0.00007821
rs148685493	5	149864258	T	13	4	C	0.000883
rs12520805	5	149864390	A	13	4	G	0.000883
rs150900613	5	149866587	A	13	4	AC	0.000883
rs12519213	5	149867885	A	13	4	C	0.000883
rs12519820	5	149868277	A	13	4	G	0.000883
rs12519827	5	149868326	A	16	4	G	0.00007821
rs79152136	5	149868870	C	16	4	T	0.00007821
rs7736222	5	149869381	C	15	4	T	0.000175
rs111288376	5	149869528	A	13	4	T	0.000883
rs7716964	5	149870057	G	16	4	A	0.00007821
rs3846704	5	149871216	C	16	4	T	0.00007821
rs3846705	5	149871333	C	16	4	T	0.00007821
rs17726511	5	149871484	T	16	4	G	0.00007821
rs17799481	5	149871771	C	16	4	T	0.00007821
rs139250076	5	149872188	ATTC	16	4	A	0.00007821
rs78946830	5	149872650	G	13	4	C	0.000883
rs74942585	5	149872666	C	16	4	T	0.00007821
rs368595259	5	149873741	C	13	3	CA	0.0001069
rs17726541	5	149873850	A	15	4	G	0.000175
rs112209415	5	149873904	CT	13	2	C	0.00006438
rs138831508	5	149874235	C	15	5	CTCTTTTCTTT	0.0003715
rs112171844	5	149874411	C	17	4	T	0.0000182
rs3846706	5	149875731	G	16	4	A	0.00007821
rs3846707	5	149875858	C	16	4	T	0.00007821
rs11167503	5	149876876	C	16	4	T	0.00007821
rs11167504	5	149876885	C	16	4	T	0.00007821

rs28372763	5	149877270	T	13	4	C	0.000883
rs57772388	5	149877773	T	16	4	A	0.00007821
rs2343814	5	149878801	G	16	4	A	0.00007821
rs2343813	5	149878840	T	16	4	C	0.00007821
rs4428396	5	149878988	C	16	4	T	0.00007821
rs74877579	5	149879246	T	13	4	C	0.000883
rs76873130	5	149879332	A	13	4	G	0.000883
rs35224765	5	149879853	AG	16	4	A	0.00007821
rs3889713	5	149882183	C	16	4	T	0.00007821
rs17726577	5	149884424	A	15	4	G	0.000175
rs113681861	5	149885169	C	13	4	G	0.000883
rs973319	5	153303618	C	16	53	T	0.0006806
rs1461238	5	153304416	C	16	53	T	0.0006806
rs35257267	5	153304538	T	16	53	C	0.0006806
rs970751	5	153305796	T	16	53	C	0.0006806
rs386248	5	153308317	T	16	53	G	0.0006806
rs35910531	5	153310336	TAAAA	16	54	T	0.0003907
rs406708	5	153311032	A	16	53	G	0.0006806
rs432891	5	153311464	C	16	53	T	0.0006806
rs394446	5	153311841	G	16	53	A	0.0006806
rs381582	5	153312282	A	16	53	G	0.0006806
rs454317	5	153312771	A	16	53	G	0.0006806
rs201644188	5	153313518	G	16	53	GAGACA	0.0006806
rs448234	5	153315300	A	16	53	G	0.0006806
rs1438586	5	153315743	C	16	53	A	0.0006806
rs11167651	5	153322296	A	16	53	C	0.0006806
rs4349768	5	153323126	A	16	53	C	0.0006806
rs690091	5	153323948	G	16	53	A	0.0006806
rs557697	5	153324256	C	16	53	A	0.0006806
rs689836	5	153324406	G	16	53	A	0.0006806
rs2560068	5	153330453	T	16	53	C	0.0006806
rs5872353	5	153336184	CT	16	53	C	0.0006806
rs11355335	5	153337255	TA	16	53	T	0.0006806
rs286958	5	153338048	T	16	53	C	0.0006806
rs286959	5	153338516	C	16	53	T	0.0006806
rs286961	5	153341404	C	16	53	G	0.0006806
rs2560050	5	153346190	G	16	53	C	0.0006806
rs4958688	5	153351519	G	16	53	A	0.0006806
rs4958689	5	153351720	T	16	53	C	0.0006806
rs10053993	5	153355854	A	16	53	G	0.0006806
rs12153343	5	158964827	T	14	51	A	0.000328

rs10708390	5	158972532	C	12	46	CT	0.0005428
rs10051643	5	159636258	C	28	20	T	0.0005398
rs10080214	5	159639563	T	28	20	C	0.0005398
rs10605034	5	159641155	A	24	16	T	0.0005508
rs113426544	5	159642534	G	28	20	A	0.0005398
rs11957752	5	159648920	A	27	19	G	0.0008555
rs77519178	5	159682737	G	13	3	A	0.0002409
rs4921273	5	159699709	C	4	30	A	0.0002486
rs17057596	5	159704258	A	4	30	G	0.0002486
rs1363151	5	162756297	A	9	1	T	0.0007194
rs1421840	5	162773548	G	14	5	A	0.0008508
rs62394934	5	162979499	T	9	1	A	0.0007194
5:166406201	5	166406201	A	2	26	AG	0.0001452
5:166406205	5	166406205	GCAGCAGCAGCA(	2	23	G	0.0005167
rs6555877	5	169331938	A	10	44	G	0.000219
rs6871299	5	169334914	T	10	44	C	0.000219
rs11740615	5	169340765	T	10	45	G	0.0001954
rs11741522	5	169341951	A	11	44	C	0.0007768
rs11741667	5	169342351	C	11	45	G	0.0004315
rs11742527	5	169343217	A	11	45	G	0.0004315
rs11749769	5	169343672	G	11	45	A	0.0004315
rs6869528	5	169346855	A	11	44	G	0.0007768
rs17560633	5	169347372	T	11	44	C	0.0007768
rs978913	5	169348792	G	11	45	T	0.0004315
rs978914	5	169348810	A	11	45	G	0.0004315
5:169452780	5	169452780	CTCCT	7	0	C	0.0008679
rs3822730	5	171174254	G	17	55	C	0.000662
rs3797456	5	172754364	G	18	9	A	0.0009279
rs1421922	5	175476709	A	35	30	C	0.0006538
rs200801611	5	175481459	A	35	30	G	0.0006538
rs35312700	5	177525307	G	13	47	C	0.0009845
rs146888227	5	177525995	A	13	47	ATTTTAT	0.0009845
rs142280981	5	177536822	CTTTA	32	26	C	0.0008799
rs3812082	5	177547336	A	8	38	G	0.0005059
rs67080522	5	177549802	G	17	7	C	0.0004382
rs9392528	6	3839792	A	1	21	G	0.0002339
rs9405266	6	5426228	A	13	47	G	0.0009845
rs6906233	6	6125283	G	1	19	A	0.0008293
rs9505065	6	7188487	C	11	44	T	0.0007768
rs77563770	6	9702092	A	18	8	G	0.0005897
6:22830530	6	22830530	A	0	16	G	0.0002449

rs12198996	6	25166010	C	9	1	T	0.0007194
rs10946818	6	26371006	A	3	25	G	0.0009258
rs78512949	6	28328293	A	8	0	G	0.0003016
rs76043707	6	28338473	C	8	0	T	0.0003016
rs78620132	6	28339253	C	8	0	T	0.0003016
rs111669150	6	28362987	T	8	0	C	0.0003016
rs17393066	6	28364793	G	8	0	A	0.0003016
rs2232419	6	28367768	A	8	0	G	0.0003016
rs10484541	6	28371346	C	8	0	G	0.0003016
rs143000906	6	28374338	TA	8	0	T	0.0003016
rs56241479	6	28389174	T	9	1	G	0.0007194
rs200981348	6	28390484	TA	8	0	T	0.0003776
rs61653240	6	28403541	TA	9	0	T	0.0001333
rs2531807	6	28420013	G	8	0	A	0.0003016
rs113145976	6	28438650	C	9	1	T	0.0007194
rs200314214	6	28685339	T	34	31	C	0.0009632
rs4483030	6	30767129	T	16	7	C	0.0009539
rs4711234	6	30768583	G	16	7	A	0.0009539
rs9404967	6	30770375	G	27	20	A	0.0008358
rs9394015	6	30773640	T	29	21	C	0.0005775
rs9394016	6	30773899	C	29	21	T	0.0005775
rs12526945	6	30774522	C	29	21	T	0.0005775
rs28894972	6	30774687	A	29	21	G	0.0005775
rs35583979	6	30774724	C	17	6	CA	0.00009264
rs12664556	6	30775017	G	29	21	A	0.0005775
rs12661690	6	30775033	C	29	21	G	0.0005775
rs9378110	6	30775733	C	29	21	G	0.0005775
rs9391635	6	30775805	G	29	21	C	0.0005775
rs28986800	6	30775910	CCTGT	29	21	C	0.0005775
rs9404970	6	30776174	T	17	6	C	0.0002592
rs9404971	6	30776195	C	17	6	G	0.0002592
rs9404972	6	30776229	C	29	21	T	0.0005775
rs9391693	6	30776393	A	17	6	G	0.0002592
rs9295921	6	30776802	T	29	21	C	0.0005775
rs9295922	6	30776858	G	29	21	A	0.0005775
rs9368641	6	30777237	C	29	21	A	0.0005775
rs9380194	6	30777258	C	17	6	T	0.0002592
rs9394017	6	30777604	G	29	21	A	0.0005775
rs9380196	6	30777648	C	29	21	A	0.0005775
rs9380197	6	30778203	A	29	21	G	0.0005775
rs9391695	6	30778685	C	17	6	G	0.0002592

rs9391696	6	30778786	G	17	6	A	0.0002592
rs9378224	6	30781748	C	17	6	A	0.0002592
rs4286818	6	30781923	C	29	21	T	0.0005775
rs2894046	6	30782105	C	29	21	T	0.0005775
rs2394412	6	30782235	A	29	21	G	0.0005775
rs2394413	6	30782303	T	29	21	C	0.0005775
rs2894047	6	30782407	G	29	21	A	0.0005775
rs139856443	6	30782619	ATCTTTATT	29	21	A	0.0005775
rs9368642	6	30782659	G	29	21	T	0.0005775
rs9394019	6	30782672	G	29	21	C	0.0005775
rs374749028	6	30782789	A	31	23	AT	0.0003729
rs4959052	6	30782883	C	29	21	T	0.0005775
rs12524350	6	30782979	C	29	16	T	0.00004527
rs13197101	6	30783099	C	24	13	T	0.0001334
rs370961073	6	30783159	C	10	1	CT	0.0003065
rs28744071	6	30783167	G	8	0	A	0.0003372
rs71541425	6	30783274	C	29	21	A	0.0005775
rs115857087	6	30783300	G	29	21	A	0.0005775
rs71541427	6	30783311	C	29	21	T	0.0005775
rs28744112	6	30783334	G	17	6	T	0.0002592
rs28744185	6	30783530	A	17	6	C	0.0002592
rs10807072	6	30783896	C	29	21	T	0.0005775
rs10947100	6	30783977	A	17	6	G	0.0002592
rs9405012	6	30784109	C	29	21	G	0.0005775
rs9391810	6	30784170	T	17	6	G	0.0002592
rs9405013	6	30784258	G	29	21	C	0.0005775
rs9405014	6	30784259	T	17	6	C	0.0002592
rs9391811	6	30784296	T	17	6	C	0.0002592
rs9391812	6	30784353	C	17	6	G	0.0002592
rs35401082	6	30784439	TC	29	21	T	0.0005775
rs9295925	6	30784553	G	29	21	A	0.0005775
rs9295926	6	30784700	C	29	21	G	0.0005775
rs9348843	6	30786676	A	29	21	G	0.0005775
rs11314302	6	30786832	C	29	21	CT	0.0005775
rs4713381	6	30786927	T	17	6	C	0.0002592
rs28381566	6	30787309	CT	17	6	C	0.0002592
rs9394020	6	30792698	C	29	21	T	0.0005775
rs3888582	6	30794004	T	29	21	A	0.0005775
rs4248151	6	30794346	C	29	21	T	0.0005775
rs9262252	6	30828433	A	4	28	G	0.0008078
rs2894059	6	30828434	A	4	28	G	0.0008078

rs3130797	6	30846088	T	4	28	C	0.0008078
rs3094108	6	30850395	A	4	28	G	0.0008078
rs3130780	6	30874308	T	4	28	G	0.0008078
rs885905	6	30890431	A	6	34	G	0.0005004
rs2257347	6	30892054	A	6	32	G	0.00098
rs2532941	6	30892521	G	6	35	A	0.0002764
rs1043483	6	30893728	C	6	34	T	0.0005004
rs2532925	6	30899163	G	6	35	A	0.0002764
rs2253588	6	30899377	C	6	35	G	0.0002764
rs9368662	6	31156148	T	22	12	G	0.0004525
rs9368663	6	31156155	T	22	13	C	0.0006663
rs6929268	6	40704959	C	6	36	A	0.0001537
rs9471531	6	41242200	G	14	50	A	0.0005849
rs11754211	6	43106226	T	14	4	C	0.0003939
rs78991495	6	47730859	A	16	5	G	0.0004508
6:55717970	6	55717970	T	2	25	TAAAATAAAATAAAATAAA/	0.0001556
rs681089	6	71036220	A	35	29	G	0.0003069
rs9354914	6	71036721	A	16	53	G	0.0006806
rs12524645	6	71036888	A	16	53	G	0.0006806
rs13216351	6	71037050	G	16	53	A	0.0006806
rs13199908	6	71037205	A	16	53	G	0.0006806
rs34123608	6	71037266	G	16	53	GA	0.0006806
rs11757241	6	71037412	T	16	53	C	0.0006806
rs2203664	6	71037500	A	16	53	C	0.0006806
rs696988	6	71038944	A	34	29	G	0.0006139
rs718352	6	71039156	T	16	53	C	0.0006806
rs6921980	6	71040195	A	34	29	C	0.0006139
rs4299788	6	71041772	T	16	53	C	0.0006806
rs4707778	6	71042128	T	34	29	C	0.0006139
rs3106871	6	71042160	C	34	29	T	0.0006139
rs479783	6	71042358	T	34	29	C	0.0006139
rs58595644	6	71044616	T	34	29	TG	0.0006139
rs1711715	6	71044871	T	34	29	C	0.0006139
rs1739676	6	71044875	C	34	29	T	0.0006139
rs1739675	6	71044892	A	34	29	T	0.0006139
rs1829198	6	71045723	A	16	53	G	0.0006806
rs1614788	6	71046269	T	34	29	C	0.0006139
rs34203299	6	71046377	G	14	47	GA	0.0009636
rs1321066	6	71047476	T	34	29	C	0.0006139
rs1621078	6	71048428	A	34	29	G	0.0006139
rs34308048	6	71048604	G	34	29	GT	0.0006139

rs1711724	6	71048651	G	34	29	A	0.0006139
rs1589045	6	71048860	C	34	29	A	0.0006139
rs1739668	6	71048886	T	34	29	G	0.0006139
rs1711725	6	71048944	T	34	29	C	0.0006139
rs9364081	6	71049401	T	16	53	A	0.0006806
rs10155708	6	71050009	G	34	29	A	0.0006139
rs10155683	6	71050033	T	34	29	C	0.0006139
rs9364082	6	71050050	C	34	29	T	0.0006139
rs9354917	6	71050154	A	34	29	G	0.0006139
rs6907779	6	71050202	A	34	29	G	0.0006139
rs1830499	6	71050871	T	16	53	C	0.0006806
rs1590166	6	71051093	T	34	29	C	0.0006139
rs70990303	6	71051438	CT	34	29	C	0.0006139
rs1739665	6	71051475	C	34	29	T	0.0006139
rs9346380	6	71052169	T	34	29	C	0.0006139
rs9455058	6	71052661	G	16	53	A	0.0006806
rs1092508	6	71052975	C	34	29	G	0.0006139
rs1092259	6	71053500	A	34	29	G	0.0006139
rs9342786	6	71053744	A	16	53	G	0.0006806
rs4707782	6	71053876	T	16	53	C	0.0006806
rs9455063	6	71055346	A	16	53	T	0.0006806
rs781115	6	71055814	C	19	59	T	0.0006234
rs80303828	6	72509127	C	15	5	T	0.0004343
rs9341425	6	74175192	A	7	38	T	0.0001991
rs9359036	6	74241438	T	8	38	C	0.0005059
rs9343053	6	74246392	A	8	38	G	0.0005059
rs9343054	6	74246847	A	8	38	G	0.0005059
rs112254029	6	74249532	T	8	38	TC	0.0005059
rs62438402	6	74249533	T	8	38	C	0.0005059
rs656724	6	74252706	C	8	38	T	0.0005059
rs616610	6	74258360	A	8	38	T	0.0005059
rs36067667	6	74260951	GA	8	39	G	0.0004628
rs61332147	6	74266085	A	7	38	AAAAAAACAAAAAAC	0.0003582
rs2643198	6	74269691	T	8	38	C	0.0005059
rs11299381	6	74271069	GT	8	38	G	0.0005059
rs534562	6	74283946	G	8	39	C	0.0004628
rs9343056	6	74366061	A	6	33	C	0.0009098
rs4145195	6	74379180	A	6	32	G	0.00098
rs6453688	6	74379809	T	6	32	A	0.00098
rs6921096	6	74381301	G	6	33	A	0.0009098
rs375668817	6	74383626	TTG	6	33	T	0.0009098

rs199867892	6	74383629	AACCCAGGAGGA	6	33	A	0.0009098
rs9446978	6	74385013	T	21	9	A	0.00008232
rs9446979	6	74387591	A	6	32	C	0.00098
rs1876700	6	74388742	T	6	32	A	0.00098
rs4544874	6	74390391	C	6	32	G	0.00098
rs6901285	6	74390527	G	6	32	A	0.00098
rs2351307	6	74392921	A	6	32	G	0.00098
rs9446981	6	74394785	A	6	33	G	0.0009098
rs3907648	6	74395505	G	6	32	A	0.00098
rs3050889	6	74397916	C	6	32	CTGTGA	0.00098
6:79106259	6	79106259	T	14	4	TG	0.0004351
6:79106260	6	79106260	A	14	4	G	0.0003939
6:88836867	6	88836867	AATG	28	16	A	0.00003871
rs11757976	6	88836870	G	29	14	A	0.000004298
rs976832	6	92526806	C	16	6	T	0.0005436
rs1498249	6	92529713	G	16	6	T	0.0005436
rs2454253	6	92536582	T	16	6	C	0.0005436
rs2454251	6	92536639	C	16	6	G	0.0005436
rs2454250	6	92536643	T	16	6	C	0.0005436
rs4457133	6	92537385	A	16	6	C	0.0005436
rs4265011	6	92537625	A	16	6	G	0.0005436
rs1498261	6	92539077	T	16	6	C	0.0005436
rs1353700	6	92539213	A	16	6	G	0.0005436
rs2501653	6	92548874	G	16	6	T	0.0005436
rs2454273	6	92550258	C	16	6	A	0.0005436
rs1979793	6	92553419	C	16	6	T	0.0005436
rs35977654	6	92554141	C	16	6	T	0.0005436
rs2255287	6	92559843	T	16	6	A	0.0005436
rs2454269	6	92560297	C	16	6	A	0.0005436
rs56164195	6	92582923	A	16	6	AC	0.0005436
rs4707701	6	92584877	T	16	6	C	0.0005436
rs946120	6	97544329	A	39	25	G	0.0000005193
rs11361496	6	97545509	CT	39	32	C	0.00004458
rs1206153	6	97546036	T	39	32	C	0.00004458
rs1206159	6	97558136	A	38	30	G	0.00003943
rs1737131	6	97558907	C	15	58	G	0.00002069
rs1737132	6	97558912	T	15	58	G	0.00002069
rs1206160	6	97561059	C	14	57	T	0.00001769
rs1206161	6	97561088	C	14	57	T	0.00001769
rs1206146	6	97563222	T	14	57	C	0.00001769
rs25637	6	97563868	CTGTT	14	58	C	0.000008395

rs1206147	6	97565173	G	14	58	A	0.000008395
rs1206148	6	97566464	T	14	56	C	0.00001636
rs1206149	6	97566938	A	14	51	G	0.000328
rs1206150	6	97568482	G	15	57	A	0.00004089
rs1206151	6	97568613	T	15	57	C	0.00004089
rs1206152	6	97568963	C	15	57	A	0.00004089
rs9285408	6	97571877	T	15	51	C	0.0006974
rs1206166	6	97572581	C	15	57	T	0.00004089
rs1206167	6	97572903	G	15	57	A	0.00004089
rs1206168	6	97573678	C	15	57	A	0.00004089
rs1206169	6	97573953	T	15	57	C	0.00004089
rs2206094	6	97575196	T	15	57	C	0.00004089
rs1612775	6	97575423	C	15	57	G	0.00004089
rs1766531	6	97576033	C	15	57	T	0.00004089
rs1737135	6	97577031	G	15	57	A	0.00004089
rs6568746	6	97578265	C	15	56	T	0.0000815
rs1206145	6	97585786	G	15	56	C	0.0000815
rs1206176	6	97587527	A	15	56	T	0.0000815
rs1206177	6	97590623	T	15	56	C	0.0000815
rs1206178	6	97591680	A	15	56	G	0.0000815
rs1206134	6	97601582	T	15	56	A	0.0000815
rs140706	6	97603195	A	15	56	AATTGTT	0.0000815
rs1206137	6	97605268	G	15	56	A	0.0000815
rs1206139	6	97606816	C	15	56	G	0.0000815
rs1766513	6	97607265	G	15	56	T	0.0000815
rs1737140	6	97607266	A	15	56	C	0.0000815
rs1206141	6	97608206	T	15	56	G	0.0000815
rs3061513	6	97612420	CAAAAT	15	56	C	0.0000815
rs1206124	6	97612708	G	15	56	T	0.0000815
rs1209290	6	97613163	C	15	56	T	0.0000815
rs1206125	6	97616717	G	15	56	A	0.0000815
rs11317728	6	97618338	G	17	56	GT	0.0003692
rs1206126	6	97618476	G	16	56	C	0.0001751
rs1623061	6	97621318	T	15	56	A	0.0000815
rs1623056	6	97621320	T	15	56	C	0.0000815
rs3061515	6	97621860	G	15	56	GCTC	0.0000815
rs1206129	6	97622500	A	14	56	G	0.00002211
rs6926003	6	97624248	C	15	56	T	0.0000815
rs1766470	6	97625034	C	15	56	T	0.0000815
rs1014976	6	97628442	T	15	56	G	0.0000815
rs6913262	6	97629155	C	15	56	T	0.0000815

rs1206170	6	97629381	G	15	56	C	0.0000815
rs1206171	6	97629689	A	15	56	C	0.0000815
rs717020	6	97630619	T	15	56	C	0.0000815
rs1206172	6	97631143	C	15	56	T	0.0000815
rs1209293	6	97632347	T	15	56	C	0.0000815
rs1206174	6	97637114	T	14	54	C	0.00003985
rs1206175	6	97639120	C	15	56	T	0.0000815
rs1206163	6	97642227	T	15	56	C	0.0000815
rs6931754	6	97644798	T	15	56	C	0.0000815
rs6932480	6	97645222	G	15	56	C	0.0000815
rs199760978	6	100330942	A	13	2	ATATATATATAT	0.00007644
6:100330946	6	100330946	A	7	0	ATATATAT	0.0009566
rs1998736	6	105886985	A	17	7	G	0.0004382
rs7765899	6	105896843	T	17	7	C	0.0004382
rs17797252	6	125681602	A	3	25	T	0.0009258
rs17797323	6	125683552	A	3	25	G	0.0009258
rs17740938	6	125683815	C	3	25	T	0.0009258
rs17797377	6	125684251	G	3	25	A	0.0009258
rs117239907	6	129086098	T	7	0	C	0.0008679
rs139839707	6	129529703	A	0	16	AAGAAAG	0.0005294
rs149221317	6	129529821	A	0	16	AGAAG	0.0005294
rs9398914	6	129986453	G	29	22	C	0.0007063
rs372889112	6	132830595	AC	16	7	A	0.0007388
6:151367629	6	151367629	G	0	15	A	0.0004006
6:151367631	6	151367631	A	0	15	ATAT	0.0004006
rs851977	6	152029608	C	6	32	T	0.00098
rs851976	6	152030008	C	6	32	T	0.00098
rs34053404	6	152036607	TC	6	32	T	0.00098
rs11756568	6	152042413	T	6	32	A	0.00098
rs7772579	6	152042502	C	6	32	A	0.00098
rs3020340	6	152043290	G	6	32	A	0.00098
rs3020341	6	152047557	C	6	32	G	0.00098
rs3020342	6	152047753	G	6	32	A	0.00098
rs6923710	6	152876122	G	21	12	C	0.0009318
rs2623960	6	152893441	T	20	10	C	0.0005561
rs12213593	6	152896250	A	21	12	T	0.0009318
rs1936729	6	153013061	T	18	8	C	0.0005897
rs13211784	6	153066369	G	18	8	T	0.0005897
rs12193114	6	155179754	G	21	12	A	0.0009318
rs764420	6	155182564	G	19	8	A	0.0002919
rs17085762	6	155194880	G	20	10	T	0.0005561

rs11756339	6	155203746	G	19	9	C	0.0004395
rs12200590	6	155213791	C	20	11	A	0.0008202
rs11759770	6	155216072	C	20	11	T	0.0008202
rs12215496	6	155219791	T	20	11	C	0.0008202
rs4523098	6	156109429	A	8	0	T	0.0003016
rs4642490	6	156109467	G	8	0	A	0.0003016
rs4631304	6	156114139	T	8	0	C	0.0003016
rs13203482	6	158865022	C	9	1	T	0.0007194
rs34871904	6	164419870	T	7	0	C	0.0008679
rs206731	6	164503657	A	9	1	C	0.0007194
rs34883865	6	164519315	T	7	0	G	0.0008679
rs13214170	6	164533595	A	7	0	G	0.0008679
rs77742773	7	178778	T	7	0	C	0.0008679
rs36156024	7	299642	A	13	48	G	0.0005647
rs36173075	7	299976	A	12	48	G	0.0002609
rs36179424	7	300864	T	11	48	G	0.0001177
rs36132515	7	301269	A	11	48	G	0.0001177
rs137936691	7	301592	C	11	48	G	0.0001177
rs145903549	7	301646	T	11	47	C	0.0001343
rs146049438	7	302713	T	11	46	C	0.0002401
rs148712790	7	302742	T	11	45	C	0.0004315
rs141483653	7	302781	G	11	45	C	0.0004315
rs36181705	7	304301	G	26	18	T	0.0007809
rs36127047	7	304633	G	26	18	A	0.0007809
rs142523453	7	305124	A	11	46	G	0.0002401
rs36144408	7	305366	A	26	18	G	0.0007809
rs149104177	7	305433	A	11	45	G	0.0004315
rs36175942	7	307087	G	26	18	A	0.0007809
rs36188152	7	307113	G	26	18	A	0.0007809
rs142817657	7	308803	G	10	45	A	0.0001954
rs147412934	7	308829	T	10	44	C	0.000219
rs6943256	7	3294678	C	1	21	T	0.0002339
rs6967704	7	3295384	A	11	43	T	0.0008678
rs11982357	7	3295697	G	11	43	A	0.0008678
rs6948159	7	3302628	C	11	43	G	0.0008678
rs13230939	7	3303735	A	8	38	G	0.0005059
rs10654490	7	3304547	A	8	38	AAAC	0.0005059
rs10232995	7	5129342	C	3	25	T	0.0009258
rs1859512	7	8535486	T	9	1	C	0.0007194
rs62448063	7	8537239	T	9	1	C	0.0007194
rs78646483	7	8537781	G	9	1	GTTA	0.0007194

rs17149768	7	8538351	A	9	1	T	0.0007194
rs17404592	7	8538451	C	9	1	G	0.0007194
rs12702770	7	8749807	A	13	47	G	0.0009845
rs75876133	7	17262196	G	0	16	T	0.0005294
rs143469691	7	22059039	C	13	4	T	0.000883
rs6462091	7	28514580	C	9	41	T	0.0003404
rs6462092	7	28514625	G	2	22	C	0.0009583
rs12674383	7	28515071	T	2	22	A	0.0009583
rs34388783	7	28515097	AAAG	10	42	A	0.0006808
rs9791765	7	28515392	A	2	22	C	0.0009583
rs13242388	7	28518903	A	2	22	C	0.0009583
rs34323713	7	28519179	G	2	22	C	0.0009583
rs35894653	7	28519197	G	2	22	A	0.0009583
rs114066291	7	28519652	G	2	23	C	0.0005167
rs4722804	7	28532464	T	2	26	G	0.0001452
rs4722806	7	28534115	A	2	24	G	0.0002818
rs13224567	7	28539195	G	2	24	C	0.0002818
rs12671325	7	28539563	T	2	24	A	0.0002818
rs7807102	7	28540019	T	2	24	C	0.0002818
rs7802212	7	28546366	T	2	24	C	0.0002818
rs71876335	7	28550825	T	2	24	TGGTCAGGCA	0.0002818
rs12700889	7	28551585	A	2	22	G	0.0009583
rs71555746	7	28553169	C	2	22	CTTATA	0.0009583
rs10046532	7	30971322	T	1	19	A	0.0008417
rs28808367	7	30980546	C	2	22	T	0.0009583
rs6462239	7	30981435	G	2	22	A	0.0009583
rs80161983	7	30982439	G	2	22	A	0.0009583
rs57439212	7	30983904	G	2	22	T	0.0009583
rs4723028	7	30985232	G	2	22	C	0.0009583
rs10265514	7	30985769	A	2	22	G	0.0009583
rs4000207	7	30988163	G	2	23	A	0.0005167
rs76778316	7	30990363	A	0	17	G	0.0002606
rs4723034	7	30994911	G	2	22	T	0.0009583
rs73305796	7	30997063	A	2	22	G	0.0009583
rs1894850	7	30997245	A	2	22	G	0.0009583
rs10271796	7	30998184	T	2	22	C	0.0009583
rs112395084	7	30998810	A	2	22	G	0.0009583
rs4723036	7	30999116	T	2	22	C	0.0009583
rs75702393	7	37401837	AT	21	12	A	0.0009318
7:37401838	7	37401838	ATAT	21	12	A	0.0009318
7:38430592	7	38430592	G	1	23	GA	0.0001244

rs2329396	7	39534540	A	33	28	G	0.0009909
rs7797586	7	39539525	A	36	32	T	0.0006397
rs7778850	7	39539715	G	36	32	C	0.0006397
rs55813588	7	39540119	A	36	32	AG	0.0006397
rs2278365	7	39540611	T	36	32	C	0.0006397
rs139669364	7	42438916	GA	9	0	G	0.0001034
rs76889858	7	46919281	T	7	0	C	0.0008679
rs148068890	7	46956216	A	7	0	T	0.0008679
rs117127634	7	46965364	T	8	0	C	0.0003016
rs71545975	7	47144505	A	10	2	G	0.0008882
rs73118262	7	52667755	G	11	44	A	0.0007768
rs35286387	7	52757969	T	10	42	A	0.0006808
rs34904869	7	52760201	T	10	42	G	0.0006808
rs1442236	7	52761379	C	10	42	T	0.0006808
rs845552	7	55245507	G	16	54	A	0.0003907
rs845555	7	55247334	T	16	53	C	0.0006806
rs12672565	7	55656562	G	32	26	A	0.0008799
rs111322735	7	61786170	A	5	34	T	0.0001138
rs73125943	7	62070077	A	7	0	G	0.0008679
7:64580618	7	64580618	G	11	2	A	0.0003416
rs10551417	7	71563744	G	15	6	GGC	0.000384
rs200136831	7	75372323	T	13	47	TA	0.0005538
rs200981755	7	79359478	T	9	44	TAAATATTTAAAATATTGAT	0.0000882
rs62468686	7	81063239	T	13	4	C	0.000883
rs2079888	7	84877586	A	29	22	T	0.0007063
rs13247006	7	84883983	G	29	20	T	0.0003112
rs12674373	7	84884322	C	32	23	T	0.000199
rs1569114	7	85003763	A	4	31	C	0.0001398
rs374778279	7	85006798	A	6	35	AAT	0.0002556
rs2435272	7	85007066	T	11	50	G	0.00003137
rs2463468	7	85009349	T	11	50	C	0.00003137
rs2030624	7	85013958	C	11	50	T	0.00003137
rs2463475	7	85016000	T	11	50	C	0.00003137
rs2462058	7	85019297	T	11	50	A	0.00003137
rs1403785	7	85021953	A	11	50	G	0.00003137
rs2462057	7	85024037	T	11	50	C	0.00003137
rs1960550	7	85024643	T	11	50	C	0.00003137
rs2077770	7	85026097	T	11	50	A	0.00003137
rs2463478	7	85030592	G	11	48	T	0.0001177
rs1583081	7	85034227	T	11	50	G	0.00003137
rs2463480	7	85036389	T	11	50	C	0.00003137

rs2435264	7	85036405	T	11	50	A	0.00003137
rs2463481	7	85037374	G	10	44	A	0.000219
rs148525782	7	85045118	A	11	50	AT	0.00003137
rs112461735	7	85045121	C	11	50	A	0.00003137
rs7794512	7	85045881	A	10	44	G	0.000219
rs34140074	7	85054102	A	19	58	AT	0.0006983
rs1829902	7	85057001	C	19	58	G	0.0006983
rs35288706	7	85058425	GA	19	58	G	0.0006983
rs34970164	7	85058967	GA	19	58	G	0.0006983
rs12704128	7	85059181	A	19	58	G	0.0006983
rs13237273	7	85063736	C	19	58	T	0.0006983
rs10952847	7	85064804	C	19	58	T	0.0006983
rs17159934	7	85070736	A	18	58	C	0.000347
rs17159935	7	85070949	A	18	58	C	0.000347
rs1560315	7	85075150	G	18	58	C	0.000347
rs10499888	7	85078623	A	19	58	G	0.0006983
rs73193990	7	85079015	A	19	58	G	0.0006983
rs35523949	7	85085202	G	19	58	A	0.0006983
rs143204611	7	85086268	TTTAATTATGGTC	18	58	T	0.000347
rs35295520	7	85091841	G	18	58	GA	0.000347
rs2462049	7	85099898	G	15	52	T	0.0006042
rs78388085	7	86482580	C	0	15	T	0.0005628
rs10226491	7	86483501	C	0	15	T	0.0005628
rs10269943	7	86483503	A	0	15	G	0.0005628
rs58496134	7	86483847	TAATAAA	0	15	T	0.0005628
rs60438843	7	86484166	C	0	15	G	0.0005628
rs7792069	7	86485557	C	0	15	G	0.0005628
rs10263372	7	86486846	G	0	15	C	0.0005628
rs73382349	7	86489205	A	0	15	G	0.0005628
rs7808623	7	86490154	G	0	15	T	0.0005628
rs17161067	7	86490264	C	0	15	T	0.0005628
rs28479466	7	86491021	T	0	15	A	0.0005628
rs28755802	7	86491029	A	0	15	G	0.0005628
rs7781782	7	86506594	G	0	15	A	0.0005628
rs6972489	7	86523164	A	0	15	G	0.0005628
rs28405414	7	86525772	A	0	15	G	0.0005628
rs17161098	7	86531065	A	0	15	G	0.0005628
rs148521890	7	86531485	GTTGT	0	15	G	0.0005628
rs35895874	7	86542455	T	0	15	C	0.0005628
rs1029367	7	86544248	T	0	15	C	0.0005628
rs28410495	7	86552503	A	0	15	G	0.0005628

rs139183478	7	86554071	T	0	16	TG	0.0005294
rs145574697	7	86562259	A	0	15	ATTT	0.0005628
rs10216150	7	86562755	G	0	15	A	0.0005628
rs10215436	7	86562756	A	0	15	C	0.0005628
rs12111888	7	86581391	G	0	15	A	0.0005628
rs28362761	7	86583044	T	0	15	A	0.0005628
rs17161118	7	86586561	T	0	15	C	0.0005628
rs10282116	7	94566679	T	14	50	C	0.0005849
rs10246268	7	94567965	A	14	51	T	0.000328
rs6949844	7	94583279	T	15	51	A	0.0006974
rs854717	7	94593140	A	14	51	G	0.000328
rs11981513	7	94648239	A	37	31	G	0.0001688
rs7783267	7	101330957	T	2	28	A	0.00003728
rs17368789	7	106210885	G	6	33	T	0.0009098
rs13231035	7	113053088	G	13	54	A	0.0000357
rs425956	7	114719858	C	8	38	T	0.0005059
rs73217748	7	122738762	G	22	13	A	0.0006749
rs17149636	7	126018952	G	3	25	A	0.0009258
rs10248006	7	126034791	G	26	16	A	0.0002858
rs887025	7	126040117	C	26	15	T	0.0001216
rs887026	7	126040259	A	26	15	T	0.0001216
rs4731312	7	126041074	C	16	5	A	0.0001588
rs12113410	7	126044778	A	16	5	G	0.0001588
rs756969	7	126051716	G	3	25	T	0.0009258
rs12531943	7	126055409	C	34	26	T	0.0001338
rs2028058	7	126059071	A	34	25	G	0.00006745
rs2106197	7	126060738	C	33	26	T	0.0002784
rs1016973	7	126063696	G	26	17	A	0.0003852
rs34056637	7	126064156	GTATC	26	17	G	0.0003852
rs56171722	7	126064759	CT	26	17	C	0.0003852
rs7796433	7	136074061	A	28	20	G	0.0005398
rs7796512	7	136074356	C	28	20	A	0.0005398
rs10264196	7	136076820	G	28	20	A	0.0005398
rs12707301	7	136078014	T	28	20	C	0.0005398
rs12707302	7	136086103	G	28	20	A	0.0005398
rs368726252	7	136099934	T	28	18	A	0.000203
rs35567576	7	136107895	C	28	20	CACT	0.0005398
rs2349736	7	136108549	A	28	20	T	0.0005398
rs17168667	7	136111845	G	9	1	T	0.0007194
7:136115344	7	136115344	C	7	0	CAT	0.0008679
7:136195265	7	136195265	T	7	0	TAAG	0.0008679

rs188902097	7	136300945	T	7	0	A	0.0008679
rs7801517	7	136464049	A	26	17	G	0.0005997
rs28393971	7	137992022	A	13	3	T	0.0002093
rs10261424	7	138556831	T	11	44	A	0.0007768
rs10261559	7	138556954	C	11	44	A	0.0007768
rs10954634	7	138557068	T	7	42	C	0.00002668
rs34717241	7	138560840	C	11	44	T	0.0007768
rs10249247	7	138561175	G	11	44	C	0.0007768
rs10278730	7	138561260	G	11	44	A	0.0007768
rs10252467	7	138561417	A	11	44	C	0.0007768
rs12114019	7	138565405	T	10	44	G	0.000219
rs143461168	7	138588606	CGT	4	28	C	0.0007865
rs10278421	7	138588610	T	4	27	C	0.000811
rs117919318	7	140083539	T	14	4	C	0.0003939
rs62483146	7	144225556	A	14	5	T	0.0008508
rs17798068	7	144877432	T	23	12	C	0.0002434
rs13234300	7	144882368	A	22	6	G	0.00000161
rs12386575	7	144882592	G	26	13	T	0.00003493
rs10228815	7	144882889	G	21	6	T	0.000004184
rs35064577	7	144883441	A	25	13	AC	0.00007409
rs66810244	7	144884238	G	25	12	C	0.00002059
rs71528967	7	144884334	A	21	6	G	0.000004184
rs34397230	7	144884596	C	25	12	T	0.00002834
rs6956030	7	144884884	G	25	12	A	0.00002834
rs2371838	7	144885248	C	25	12	G	0.00002834
rs2888169	7	144885398	A	25	12	G	0.00002834
rs2371839	7	144885447	A	25	12	G	0.00002834
rs4272286	7	144885533	C	25	12	G	0.00002834
rs4484599	7	144885638	A	25	12	C	0.00002834
rs56046712	7	144885654	T	25	12	C	0.00002834
rs13240871	7	144885939	G	25	12	A	0.00002834
rs10232491	7	144885995	T	25	12	G	0.00002834
rs10262936	7	144886172	T	25	12	C	0.00002834
rs10263180	7	144886365	T	25	12	C	0.00002834
rs10263307	7	144886410	T	25	11	C	0.00001926
rs10233496	7	144886823	A	25	12	G	0.00002834
rs35551476	7	144887688	CA	25	12	C	0.00002834
rs10237587	7	144887701	A	25	12	G	0.00002834
rs10237736	7	144887821	A	25	12	G	0.00002834
rs10230667	7	144887987	C	25	12	T	0.00002834
rs10228165	7	144888530	G	25	12	A	0.00002834

rs7799776	7	144888698	T	21	6	C	0.000004184
rs7782759	7	144888944	G	25	12	A	0.00002834
rs7786860	7	144889158	A	25	12	T	0.00002834
rs7786717	7	144889286	T	25	12	A	0.00002834
rs12703655	7	144889357	C	25	12	T	0.00002834
rs12703656	7	144889407	T	25	12	C	0.00002834
rs12703657	7	144889435	C	25	12	T	0.00002834
rs12703658	7	144889457	A	25	12	G	0.00002834
rs12703659	7	144889468	T	25	12	C	0.00002834
rs12703660	7	144889611	G	25	12	C	0.00002834
rs12703661	7	144889622	C	25	12	G	0.00002834
rs13228764	7	144889646	G	25	12	A	0.00006375
rs13228765	7	144889652	G	25	12	A	0.00006375
rs12532810	7	144890664	G	25	12	A	0.00002834
rs12535162	7	144890750	C	25	12	G	0.00002834
rs12533418	7	144890960	C	25	12	T	0.00002834
rs12533931	7	144890997	A	25	12	T	0.00002834
rs12533395	7	144891006	C	25	12	A	0.00002834
rs12535710	7	144891096	A	25	12	G	0.00002834
rs28465582	7	144891444	A	25	12	G	0.00002834
rs28718081	7	144891453	C	25	12	T	0.00002834
rs13221722	7	144891618	T	25	12	G	0.00002834
rs12534658	7	144891996	A	25	12	T	0.00002834
rs12531488	7	144892086	T	25	12	C	0.00002834
rs34987711	7	144892400	G	25	12	A	0.00002834
rs7788595	7	144893242	A	21	6	C	0.000004184
rs34694182	7	144893767	A	22	9	AT	0.00004313
rs2371843	7	144895554	A	19	5	G	0.00001146
rs2371844	7	144895575	A	19	5	G	0.00001146
rs12703663	7	144895979	G	19	5	A	0.00001146
rs13247774	7	144896721	A	19	5	G	0.00001146
rs71528968	7	144901501	T	21	8	C	0.00003188
rs150195176	7	144911246	T	19	5	C	0.00001146
rs12703667	7	144915712	T	19	6	C	0.00002708
rs139336131	7	144935515	A	13	2	G	0.00006438
rs12703668	7	144938998	C	18	5	T	0.00002769
rs66706284	7	144946452	A	19	5	G	0.00001146
rs73170827	7	144947604	T	16	3	A	0.0000134
rs34223395	7	144950375	T	19	5	C	0.00001146
rs2888208	7	144953486	G	19	8	T	0.0002919
rs12703672	7	144965537	C	19	6	A	0.00002708

7:151092596	7	151092596	CGG	9	0	C	0.0001034
rs143970329	7	153517490	G	17	6	T	0.0002592
rs11451890	7	153520700	G	23	14	GT	0.0007414
rs4725514	7	153520711	C	23	14	A	0.0007414
rs2622260	7	153521183	A	24	14	G	0.0003806
rs6464368	7	153521224	G	24	14	A	0.0003806
rs7795045	7	153521738	T	24	14	C	0.0003806
rs150245240	7	153994161	T	7	0	TG	0.0006731
rs13223976	7	154976046	C	18	57	T	0.0006427
rs7794634	7	154980152	T	18	57	C	0.0006427
rs10279840	7	154980616	A	18	57	C	0.0006427
7:155817937	7	155817937	TGTGTGTGTGGGT	11	2	T	0.0005111
rs55935121	7	156687241	G	1	20	A	0.0004403
rs10234398	7	156699525	A	2	22	G	0.0009583
rs10949613	7	156715941	A	2	22	G	0.0009583
rs11772112	7	156727939	C	1	21	A	0.0002339
rs12697999	7	156737675	A	14	50	G	0.0005849
rs2365991	7	156740311	T	16	54	C	0.0003907
rs10273308	7	156749283	G	14	50	A	0.0005849
rs7458640	7	156750236	G	14	50	A	0.0005849
rs887614	7	156750695	G	14	50	A	0.0005849
rs887613	7	156750843	G	14	50	A	0.0005849
rs887612	7	156750945	C	14	50	T	0.0005849
rs887611	7	156751230	G	14	50	A	0.0005849
rs2365992	7	156751365	T	14	50	A	0.0005849
rs887610	7	156751377	T	14	50	C	0.0005849
rs10234725	7	156751997	C	14	50	T	0.0005849
rs10263247	7	156752018	G	14	50	A	0.0005849
rs10253363	7	156752231	T	14	50	C	0.0005849
rs6943879	7	156760485	A	14	50	G	0.0005849
rs10272415	7	156761137	C	14	50	T	0.0005849
rs9969350	7	156761751	C	14	51	T	0.000328
rs2302445	7	156761818	G	14	50	A	0.0005849
rs2302443	7	156762224	G	14	50	C	0.0005849
rs12919	7	156762248	G	14	50	A	0.0005849
rs3087905	7	156762446	G	14	50	T	0.0005849
rs12698009	7	156763188	T	14	50	C	0.0005849
rs12698010	7	156763248	C	14	50	T	0.0005849
rs4434512	7	156763550	A	14	50	G	0.0005849
rs4326276	7	156763588	C	14	50	T	0.0005849
rs4434513	7	156763589	A	14	50	T	0.0005849

rs5888698	7	156763602	C	14	50	CT	0.0005849
rs10260387	7	156764039	T	14	50	C	0.0005849
rs10260499	7	156764072	T	14	50	C	0.0005849
rs35889324	7	156764269	T	14	50	TG	0.0005849
rs34351910	7	156764279	A	14	50	G	0.0005849
rs6952436	7	156764431	T	14	50	G	0.0005849
rs3800868	7	156764766	A	14	50	C	0.0005849
rs3800867	7	156764887	G	14	50	A	0.0005849
rs3823623	7	156764972	G	14	50	A	0.0005849
rs7803794	7	156765043	C	14	50	A	0.0005849
rs11321727	7	156765156	GT	14	50	G	0.0005849
rs7786691	7	156765243	A	14	50	G	0.0005849
rs13242102	7	156765455	C	14	50	T	0.0005849
rs10672171	7	156765797	A	14	50	ATC	0.0005849
rs7808719	7	156765920	C	14	50	T	0.0005849
rs9647658	7	156766227	T	14	50	C	0.0005581
rs9647673	7	156766493	T	14	50	G	0.0005849
rs10266217	7	156766511	A	14	50	G	0.0005849
rs10251364	7	156766726	C	14	50	G	0.0005849
rs7796704	7	156767075	A	14	50	C	0.0005849
rs7777199	7	156767147	G	14	50	A	0.0005849
rs7777336	7	156767197	G	14	50	A	0.0005849
rs7777497	7	156767337	G	14	50	C	0.0005849
rs12538789	7	156767585	A	14	50	G	0.0005849
rs12530723	7	156767612	G	14	50	A	0.0005849
rs34559388	7	156768398	C	14	50	CTG	0.0005849
rs7806063	7	156768424	T	14	50	C	0.0005849
rs7786986	7	156768943	G	14	50	A	0.0005849
rs71189970	7	156769252	TCGTGATCCACCC	15	51	T	0.0006974
rs62492706	7	156769314	G	15	51	A	0.0006974
rs34141534	7	156769534	TA	16	53	T	0.0005124
rs9770440	7	156770033	C	14	50	T	0.0005849
rs10237164	7	156770281	G	14	52	A	0.0001845
rs10223955	7	156770396	A	14	50	G	0.0005849
rs10227139	7	156770436	T	14	50	C	0.0005849
rs7785038	7	156771174	T	14	50	C	0.0005849
rs7797591	7	156771205	G	14	50	C	0.0005849
rs11509859	7	156773156	G	14	50	A	0.0005849
rs374002060	7	156775625	CTG	14	50	C	0.0005849
rs66473256	7	156776519	TATA	13	50	T	0.0004864
rs71189975	7	156776573	CTG	14	50	C	0.0009959

rs72384323	7	158466979	A	20	12 AATT	0.0005792
rs11769858	7	158501492	T	25	17 C	0.0006387
rs113802881	7	158505363	A	24	16 G	0.0009443
rs113348912	8	2573525	TTCTTG	5	34 T	0.0001138
rs5889044	8	4377442	AGT	26	18 A	0.0007809
rs1983474	8	4403586	A	20	11 C	0.0008202
rs2725032	8	4441569	T	33	26 C	0.0002784
rs2617076	8	4445174	A	33	28 C	0.0009909
rs2724991	8	4445298	C	33	26 G	0.0002784
rs2724988	8	4445638	G	33	28 T	0.0009909
rs2617080	8	4446598	A	32	25 G	0.0004454
rs11785293	8	6435943	T	8	0 C	0.0003016
rs2945254	8	8093240	T	1	19 G	0.0008417
8:9113931	8	9113931	C	1	21 T	0.000538
rs17601783	8	14756101	C	16	7 G	0.0009539
rs10100097	8	14761792	G	16	7 A	0.0009539
rs1351413	8	14766679	G	18	9 A	0.0009279
rs150787829	8	15271944	G	9	1 A	0.0007194
rs62504242	8	16738575	G	1	20 A	0.0004403
rs59765184	8	18042251	A	3	25 T	0.0009258
rs62499972	8	20129828	A	1	20 G	0.0004403
rs62499973	8	20131277	G	1	19 C	0.0008417
rs62499974	8	20132142	T	1	20 C	0.0004403
rs11985274	8	20132201	A	1	20 T	0.0004403
rs11995187	8	20132391	T	1	20 C	0.0004403
rs62499975	8	20132529	T	1	20 C	0.0004403
rs62499976	8	20132828	G	0	20 A	0.0000355
rs111519245	8	20132873	A	1	20 G	0.0004893
rs57092179	8	20133227	T	1	20 C	0.0004403
rs60828034	8	20133304	G	1	20 T	0.0004403
rs60124846	8	20133306	A	1	20 C	0.0004403
rs62499980	8	20133714	T	1	20 C	0.0004403
rs62499981	8	20133780	C	0	19 G	0.0000703
rs62499982	8	20134176	A	1	20 G	0.0004403
rs763573	8	22803050	A	38	35 C	0.0006427
rs17334323	8	26688826	A	34	27 G	0.0002666
rs495495	8	26698168	T	10	44 A	0.000219
rs499042	8	26698515	C	10	44 A	0.000219
rs556793	8	26699067	C	16	53 T	0.0006806
rs3102087	8	26699937	A	16	53 C	0.0006806
rs2036107	8	26701444	G	10	44 A	0.000219

rs498194	8	26703221	C	9	41	T	0.0003404
rs523816	8	26703750	G	10	44	T	0.000219
rs476631	8	26706459	C	9	41	T	0.0003404
rs539144	8	26706905	A	9	40	G	0.000591
rs520180	8	26709922	G	9	41	A	0.0003404
rs512131	8	26710792	A	9	41	T	0.0003404
rs522315	8	26710975	C	9	41	A	0.0003404
rs497883	8	26712895	C	8	41	T	0.0002447
rs7008686	8	28098720	G	13	47	A	0.0009845
rs10088613	8	38808612	A	7	35	G	0.0007198
rs76242682	8	38820921	T	1	19	A	0.0008417
rs62504408	8	38821130	A	1	19	G	0.0008417
rs77574620	8	38822034	A	1	19	G	0.0008417
rs59814883	8	38908019	A	32	28	G	0.0008859
rs4617119	8	38937114	A	30	21	T	0.0002823
rs11419069	8	38953596	TA	30	23	T	0.0007875
rs61709546	8	42712987	C	12	1	G	0.00003491
rs75524513	8	42823912	A	7	0	G	0.0008679
rs72647289	8	42964349	T	12	1	C	0.00003491
rs59037494	8	42997744	T	12	1	C	0.00003491
rs190317160	8	43274690	T	10	2	C	0.0008882
rs148996977	8	55420141	A	32	31	ACTTTCTTTCTTG	0.0007289
rs117786334	8	55930236	T	7	0	C	0.0008679
rs74848436	8	55983773	T	7	0	C	0.0008679
rs11783005	8	56116482	T	24	14	C	0.0003806
rs112209050	8	56116719	G	24	14	GCCAACATAC	0.0003806
rs56081101	8	56407009	T	9	1	C	0.0007194
rs62516880	8	56789408	T	10	1	C	0.0002681
rs11996440	8	56789956	T	10	1	C	0.0002681
rs74464209	8	56792994	G	10	1	C	0.0002681
rs62507588	8	62807013	T	7	0	C	0.0008679
rs10957390	8	68457397	G	20	11	A	0.0008202
rs4737239	8	68460852	A	21	11	G	0.0003953
rs2380415	8	68462201	T	20	11	C	0.0008202
rs2380416	8	68462304	A	20	11	G	0.0008202
rs2380417	8	68462501	G	20	11	C	0.0008202
rs11785565	8	68462679	T	21	11	C	0.0003953
rs924742	8	68464647	A	22	12	G	0.000477
rs28416049	8	68975590	C	11	2	T	0.0004434
rs5009543	8	69883776	T	28	19	A	0.0002695
rs6993007	8	72705499	C	16	7	T	0.0009539

rs62518663	8	72723754	C	23	13	T	0.0003278
rs10108922	8	72724089	C	23	13	T	0.0003278
rs28460598	8	72729815	T	23	13	C	0.0003278
rs61670134	8	72739815	C	23	13	G	0.0003278
rs62518682	8	72739855	T	23	13	C	0.0003278
rs139658753	8	72740975	TTCTC	23	13	T	0.0003278
rs56262713	8	85281410	G	20	11	C	0.0008202
rs783783	8	85403656	T	28	19	G	0.0002695
rs201122439	8	85819551	T	34	25	A	0.00006745
rs4400401	8	88328968	A	13	4	G	0.000883
8:88337898	8	88337898	G	13	4	A	0.000981
rs77155700	8	89853307	C	2	22	T	0.0009583
rs79954560	8	89859652	T	2	22	C	0.0009583
rs140507702	8	89861766	C	2	23	T	0.0005167
rs6994018	8	89863807	C	2	23	T	0.0005167
rs370170108	8	89868860	T	2	22	C	0.0009583
8:89869753	8	89869753	C	2	22	T	0.0009583
rs7841185	8	89871813	A	2	22	G	0.0009583
rs113304118	8	89879992	C	1	20	G	0.0004202
rs1391629	8	89891698	G	2	22	A	0.0009583
rs79888923	8	89896133	A	2	22	T	0.0009583
rs142214846	8	89896412	GA	2	22	G	0.0009583
rs79450712	8	89897429	C	2	22	T	0.0009583
rs79342344	8	89897987	C	2	22	A	0.0009583
rs12545589	8	89898996	G	2	22	A	0.0009583
rs34462248	8	90480215	T	1	19	C	0.0008417
rs185504961	8	94163993	C	21	11	A	0.0003953
rs59851916	8	94190441	C	20	10	G	0.0005561
rs16915713	8	94190752	A	20	11	G	0.0008202
rs72662246	8	94191231	A	20	11	T	0.0008202
rs2197673	8	94192043	A	20	11	T	0.0008202
rs1374631	8	94192133	C	20	11	A	0.0008202
rs1374630	8	94192137	A	20	11	G	0.0008202
rs1374629	8	94192318	T	20	11	C	0.0008202
rs148531481	8	94192592	C	20	11	CT	0.0008202
rs16915718	8	94192856	C	20	11	T	0.0008202
rs66461754	8	94193026	A	20	11	G	0.0008202
rs10504927	8	94193643	T	20	11	A	0.0008202
rs61578878	8	94196408	G	20	11	T	0.0008202
8:95877242	8	95877242	AT	1	19	A	0.0008417
rs7828927	8	99123614	C	15	5	T	0.0003715

rs4543513	8	99148285	C	16	6	A	0.0005436
rs1264865	8	102857833	G	6	32	A	0.00098
rs1264968	8	102907502	C	2	22	G	0.0009525
rs150629554	8	103218466	TA	16	7	T	0.0009539
rs28758972	8	108777198	A	7	0	C	0.0009566
rs138007099	8	115585034	CTTTACTTCCT	9	1	C	0.0007194
rs138795202	8	115585723	A	9	1	ACAC	0.0007194
rs56358453	8	115585748	G	9	1	C	0.0007194
rs12682243	8	115589058	A	9	1	G	0.0007194
rs4278177	8	115589089	A	9	1	C	0.0007194
rs1390802	8	115589367	C	9	1	T	0.0007194
rs11987607	8	115589433	C	9	1	T	0.0007194
rs112972565	8	115592838	T	9	1	C	0.0007194
rs188331229	8	115593078	G	9	1	T	0.0007194
8:115595036	8	115595036	CT	7	0	C	0.0008679
8:115595287	8	115595287	G	9	1	C	0.0007194
rs144558766	8	115595780	T	9	1	C	0.0007194
rs368858621	8	115595883	T	9	0	C	0.0001034
8:115596736	8	115596736	A	9	1	G	0.0007194
rs4360323	8	115597453	G	9	1	T	0.0007194
rs189798615	8	115597550	A	9	1	G	0.0007194
rs4375035	8	115597614	T	9	1	G	0.0007194
rs113058397	8	115598479	T	9	1	C	0.0007194
rs113913892	8	115598520	A	9	1	T	0.0007194
rs72688152	8	115598609	C	9	1	T	0.0007194
rs140621	8	115601414	T	9	1	A	0.0007194
rs140623	8	115601454	T	9	1	C	0.0007194
rs140624	8	115601470	C	9	1	G	0.0007194
rs72688155	8	115603416	A	9	1	G	0.0007194
rs72688157	8	115605160	C	9	1	A	0.0007194
rs72688158	8	115606114	C	9	1	G	0.0007194
rs72688160	8	115606270	G	9	1	A	0.0007194
rs72688161	8	115607364	T	9	1	C	0.0007194
rs72688163	8	115607738	T	9	1	A	0.0007194
rs72688165	8	115609100	C	9	1	T	0.0007194
rs61119861	8	115609854	T	9	1	C	0.0007194
rs61642028	8	115609982	C	9	1	T	0.0007194
rs111607481	8	115610153	G	9	1	GATTTT	0.0007194
rs11436316	8	115611394	AC	9	1	A	0.0007194
rs149860162	8	115611534	A	9	1	AAATT	0.0007194
rs11436317	8	115611558	CA	9	1	C	0.0007194

rs1010296	8	115612916	G	9	1	A	0.0007194
rs59874151	8	115613474	G	9	1	A	0.0007194
rs141271051	8	115614789	G	9	1	GA	0.0007194
rs1390805	8	115615140	C	9	1	A	0.0007194
rs10505224	8	115615472	G	9	1	C	0.0008107
rs72688171	8	115617154	A	9	1	G	0.0007194
rs6993330	8	115618369	G	9	1	A	0.0007194
rs990064	8	115620846	A	9	1	G	0.0007194
rs72688174	8	115621273	T	9	1	G	0.0007194
rs67259516	8	115622714	A	9	1	G	0.0007194
rs11987094	8	115623858	G	9	1	T	0.0007194
rs113317677	8	115624633	GA	9	1	G	0.0007194
rs56744618	8	115625115	G	9	1	A	0.0007194
rs10568390	8	115625262	A	9	1	ATG	0.0007194
rs716319	8	115626526	G	9	1	A	0.0007194
rs72688177	8	115628267	A	9	1	G	0.0007194
rs11989617	8	115630616	A	9	1	G	0.0007194
rs12675391	8	115632043	T	9	1	C	0.0007194
rs9632820	8	115633517	G	21	11	T	0.0003953
rs5894209	8	115634776	ATTTC	27	18	A	0.0004513
rs1390806	8	115635282	A	9	1	G	0.0008107
rs28460811	8	115635497	C	24	13	T	0.0001822
rs4027275	8	115636252	C	23	13	A	0.0006366
rs148024664	8	115637496	G	10	1	GC	0.0003065
rs72688180	8	115638469	C	10	1	G	0.0003065
rs1156022	8	115641238	C	24	13	T	0.0001822
rs7846669	8	115641841	C	24	13	T	0.0001822
rs72688182	8	115641954	T	9	1	C	0.0008107
rs6469535	8	115642408	G	27	18	T	0.0004513
rs10087123	8	115643325	T	24	13	C	0.0001822
rs201102203	8	115644084	A	9	1	C	0.0008107
rs140178196	8	115644994	T	9	1	C	0.0008107
8:115645784	8	115645784	T	11	1	G	0.00006485
rs374695779	8	115645976	G	9	1	A	0.0008107
rs200975191	8	115646810	A	21	11	G	0.0003953
rs150137951	8	115648635	T	9	1	C	0.0007194
rs142867399	8	115648840	T	9	1	G	0.0007194
rs9297529	8	115649127	A	21	11	G	0.0003953
rs75777265	8	115649722	C	9	1	T	0.0007194
rs72688185	8	115650891	G	9	1	A	0.0007194
rs182353197	8	115652354	A	9	1	T	0.0007194

rs75910793	8	115652678	G	9	1	A	0.0007194
rs11996788	8	115653663	G	9	1	C	0.0007194
rs931614	8	115653998	A	9	1	G	0.0007194
rs1496752	8	115654884	T	9	1	C	0.0007194
rs72688196	8	115663941	G	9	1	C	0.0007194
rs200567012	8	115666287	T	9	1	G	0.0007194
8:115667919	8	115667919	A	9	1	ATTTTTT	0.0005223
rs10088421	8	115668528	G	21	11	A	0.0003953
rs72688199	8	115670389	T	9	1	G	0.0007194
rs5894213	8	115672710	A	21	11	AT	0.0003953
rs56335213	8	115675219	A	9	1	C	0.0007194
rs6985846	8	115683736	G	20	11	T	0.0008202
rs4364661	8	115686399	A	20	11	G	0.0008202
rs4610784	8	115688964	C	20	11	T	0.0008202
rs12681320	8	115692959	G	20	11	C	0.0008202
rs28605498	8	115693802	G	20	11	C	0.0008202
rs28445023	8	115701338	T	13	3	C	0.0002409
rs28505858	8	115707801	T	13	3	C	0.0002409
rs7827613	8	115712241	G	13	3	A	0.0002409
rs10086649	8	115713721	C	9	1	G	0.0007406
rs201598000	8	115713723	C	13	3	G	0.0001768
8:115713725	8	115713725	C	9	1	G	0.0007194
8:115713727	8	115713727	C	9	1	G	0.0007194
8:115714930	8	115714930	T	9	1	TA	0.0007194
rs10109368	8	115715757	C	13	3	T	0.0002409
rs71886645	8	115717208	T	13	3	TAATA	0.0002409
rs10100267	8	115721881	T	13	3	C	0.0002409
rs12677228	8	115723817	G	21	11	A	0.0003953
rs10955729	8	115724259	T	21	11	C	0.0003953
rs7462721	8	115725090	G	21	11	A	0.0003953
rs6988312	8	115726314	G	21	11	T	0.0003953
rs7006212	8	115726321	A	21	11	C	0.0003953
rs2198629	8	115727285	G	21	11	A	0.0003953
rs6994458	8	115727826	C	21	11	T	0.0003953
rs6989479	8	115730294	T	21	11	A	0.0003953
rs3062473	8	115731072	TTAG	13	3	T	0.0002409
rs35150135	8	115731550	T	13	4	C	0.000883
rs73324878	8	122791268	T	22	13	C	0.0006749
rs80348309	8	122799461	A	22	13	T	0.0006749
rs16896129	8	122809618	A	20	11	G	0.0008202
rs57882380	8	122810517	T	20	11	TAATC	0.0008202

rs73708584	8	122811694	A	20	11	C	0.0008202
rs371445185	8	122818223	C	20	11	CT	0.0008202
rs35421863	8	122818227	C	20	11	T	0.0008202
rs113486044	8	122829554	GGT	20	11	G	0.0008202
rs6984548	8	122829941	G	20	11	T	0.0008202
rs73326834	8	122830447	A	20	11	G	0.0008202
rs73326837	8	122831066	A	20	11	G	0.0008202
rs77556695	8	122831393	A	20	11	G	0.0008202
rs73326841	8	122833648	G	20	11	T	0.0008202
rs6999162	8	122838008	C	20	11	T	0.0008202
rs66461063	8	122839750	T	20	11	TGTTG	0.0008202
rs10101648	8	122839754	G	23	14	A	0.0007414
rs73326848	8	122839863	T	20	11	C	0.0008202
rs57936719	8	122840333	G	20	11	C	0.0008202
rs72609838	8	122842632	C	1	21	T	0.0002339
rs4474034	8	122842642	C	25	15	T	0.0002543
rs4132903	8	122843830	T	25	15	C	0.0002543
rs6983575	8	122844454	C	25	15	T	0.0002543
rs6983605	8	122844564	A	25	15	C	0.0002543
rs12681688	8	122845858	T	22	12	C	0.000477
rs111416674	8	122846406	T	22	12	C	0.000477
rs72609839	8	122849590	A	1	21	C	0.0002339
rs60304095	8	122852238	C	1	21	A	0.0002339
rs36006614	8	122860595	A	1	21	AT	0.0002339
rs10956060	8	122860693	G	1	21	A	0.0002339
rs10956061	8	122860731	C	1	21	A	0.0002339
rs16896253	8	122869617	G	1	21	T	0.0002339
rs72609840	8	122885169	C	1	21	T	0.0002339
rs16896361	8	122890522	C	1	19	A	0.0008417
rs66516233	8	122892653	A	1	19	G	0.0008417
8:124856940	8	124856940	T	15	5	G	0.0003715
rs2385682	8	127681857	G	0	14	C	0.0009049
8:130622402	8	130622402	G	27	17	A	0.000186
rs7840584	8	130622403	T	27	18	C	0.0004001
8:130622405	8	130622405	T	22	10	C	0.00007379
rs77848529	8	131834788	G	32	22	C	0.00008721
8:131834790	8	131834790	A	25	13	ATCC	0.0000842
8:131834792	8	131834792	G	23	13	C	0.0003278
8:131834793	8	131834793	A	23	13	C	0.0003278
9:14695	9	14695	A	8	0	G	0.0003016
9:30098	9	30098	T	9	0	C	0.0001034

rs7042540	9	343859	C	8	41	T	0.0001361
rs13295058	9	858735	A	12	45	G	0.0009266
rs7021646	9	860004	C	11	45	T	0.0004315
rs10969013	9	2929424	G	7	35	A	0.0007198
rs7864285	9	2930820	G	5	31	C	0.0006549
rs71493307	9	2930908	G	5	31	GT	0.0006549
rs10121692	9	2931315	T	5	31	C	0.0006549
rs13292810	9	2931341	A	5	31	G	0.0006549
rs59601268	9	2931638	AG	5	33	A	0.0002023
rs34078121	9	2932355	C	6	33	A	0.0009098
rs12348338	9	2932407	A	5	33	C	0.0002023
rs7858374	9	2932813	G	5	34	A	0.0001138
rs4740708	9	2993975	T	7	35	C	0.0007198
rs7849097	9	2997408	A	7	35	C	0.0007198
rs10757922	9	2997536	C	7	35	T	0.0007198
rs1537324	9	3004105	T	6	33	C	0.0009098
rs7033052	9	5042981	G	37	34	C	0.0006983
rs372117298	9	12946036	G	30	22	A	0.0001613
rs62532547	9	14204068	T	0	17	C	0.0002606
rs62532548	9	14205054	C	0	17	T	0.0002606
rs62532553	9	14218106	T	0	15	C	0.0005628
rs35402224	9	14224117	A	0	15	C	0.0005628
rs200702730	9	14382344	TGAGA	23	13	T	0.0003278
rs7848552	9	14500538	C	16	6	G	0.0005436
rs10961546	9	14510183	T	8	39	C	0.0004628
rs9886866	9	14510925	C	31	20	T	0.00004024
rs10738363	9	14511236	G	31	20	A	0.00004024
rs11793755	9	14511245	A	17	7	G	0.0004382
rs10738364	9	14511315	C	31	20	T	0.00004024
rs112966547	9	14511523	ATGAGT	31	20	A	0.00004024
rs7023433	9	14511645	A	31	20	C	0.00004024
rs7040054	9	14511793	T	31	20	A	0.00004024
rs10738365	9	14512767	T	33	28	A	0.0009909
rs4740579	9	14514325	T	25	14	C	0.0001947
rs4741388	9	14514366	C	25	14	T	0.0001947
rs10756564	9	14514783	C	30	19	T	0.00006044
rs963659	9	14515598	C	16	6	G	0.0005436
rs4741390	9	14524784	G	18	7	A	0.0001975
rs10961553	9	14527083	G	33	25	T	0.0002294
rs76268233	9	19683470	G	8	0	A	0.0003016
rs10964259	9	19740463	C	7	0	T	0.0008679

rs10757098	9	19845883	A	13	4	G	0.000883
rs12344856	9	20031365	C	4	28	A	0.0008078
rs12552907	9	20202697	A	7	0	G	0.0008679
rs35789870	9	20203975	T	7	0	TCA	0.0008679
rs67815395	9	20204573	A	7	0	G	0.0008679
rs67851366	9	20207782	T	7	0	C	0.0008679
rs13301831	9	20210027	C	7	0	G	0.0008679
rs1333775	9	20217025	A	7	0	G	0.0008679
rs7852474	9	20218285	C	7	0	G	0.0008679
rs7867959	9	20218463	A	7	0	T	0.0008679
rs10465049	9	20219031	A	7	0	G	0.0008679
rs10964480	9	20225817	T	7	0	C	0.0008679
rs4977395	9	20226358	G	7	0	A	0.0008679
rs10811332	9	20227187	G	7	0	A	0.0008679
rs11560492	9	20231812	A	7	0	G	0.0008679
rs149905185	9	20232312	G	7	0	C	0.0008679
rs200377511	9	20232602	T	7	0	C	0.0008679
rs138189734	9	20234370	T	7	0	C	0.0008679
rs4562423	9	20235378	T	7	0	C	0.0008679
rs7040926	9	20241351	C	9	1	T	0.0007194
rs4977398	9	20243207	T	7	0	A	0.0008679
rs1379217	9	20244758	G	10	1	A	0.0002681
rs10964495	9	20245283	C	10	1	T	0.0002681
rs2034876	9	20245896	G	10	1	A	0.0002681
rs765668	9	20248289	C	10	1	G	0.0002681
rs35141027	9	20250667	A	7	0	AT	0.0008679
rs10757127	9	20260958	T	7	0	A	0.0008679
rs28391312	9	20261281	A	7	0	T	0.0008679
rs16923521	9	20261635	C	7	0	T	0.0008679
rs12553948	9	20263186	G	10	1	A	0.0002681
rs4977406	9	20267556	A	7	0	G	0.0008679
rs7034958	9	20268487	C	7	0	G	0.0008679
rs1551535	9	20270444	A	7	0	C	0.0008679
rs57859418	9	20274356	C	10	1	A	0.0002681
rs4146635	9	20274698	G	10	1	A	0.0002681
rs7041951	9	20275354	G	7	0	C	0.0008679
rs10964511	9	20276363	C	10	1	T	0.0002681
rs4977251	9	20279793	G	10	1	A	0.0002681
rs4977409	9	20281811	G	7	0	T	0.0008679
rs4977412	9	20282412	G	7	0	T	0.0008679
rs60615195	9	20284302	T	7	0	G	0.0008679

rs12337967	9	26546715	G	1	20	C	0.0004893
9:32578420	9	32578420	T	7	0	C	0.0008679
9:32578423	9	32578423	T	7	0	A	0.0008679
rs117400746	9	32908910	T	1	21	C	0.000241
rs1530308	9	32911524	C	3	27	T	0.0002808
rs200201160	9	40323997	A	0	16	G	0.0005294
rs4961872	9	43396853	A	14	5	G	0.0008508
rs10907621	9	43510463	T	10	2	A	0.000793
rs143890219	9	43698587	T	10	2	C	0.0009067
rs28572375	9	43702852	A	2	22	C	0.0009525
rs10796812	9	44793373	T	14	4	C	0.0003939
rs201420555	9	65595104	A	2	25	G	0.0003162
rs373640745	9	65595491	T	2	25	C	0.0002841
rs73648380	9	65600248	T	2	22	C	0.0009583
rs199513089	9	65607945	A	2	22	G	0.0009583
rs147043737	9	65611113	A	2	25	C	0.0003162
rs200225501	9	65613073	C	2	25	G	0.0003162
rs4878855	9	65620187	T	2	25	G	0.0002833
rs147400228	9	65623432	T	2	25	A	0.0003162
rs143687106	9	65630711	T	2	25	C	0.0003162
rs200699052	9	66258887	C	14	5	A	0.0008508
rs71484888	9	66280230	G	14	5	C	0.0008508
rs115510119	9	66303655	A	14	5	G	0.0008508
rs188552917	9	66339289	A	14	5	T	0.0006534
rs201820068	9	67328193	T	8	0	C	0.0003016
rs199578430	9	67328446	A	9	1	G	0.0007194
9:67343530	9	67343530	A	7	0	G	0.0008679
9:68416460	9	68416460	G	0	15	A	0.0005628
rs76489623	9	68474714	T	12	2	G	0.0001711
rs202140006	9	69004570	A	9	1	T	0.0007194
rs374991588	9	69792207	G	11	1	C	0.00009775
rs76125007	9	69957710	C	11	2	G	0.0004434
9:70174858	9	70174858	G	7	0	T	0.0008679
rs200861709	9	75711139	A	11	43	ATTT	0.0008678
rs140164764	9	75711154	A	10	42	T	0.0006808
rs13299218	9	75714793	A	11	43	T	0.0008678
rs7041453	9	75715270	T	3	29	C	0.0001425
rs367712245	9	75715272	T	11	43	C	0.0008678
rs57593530	9	76959262	G	14	52	GT	0.0001327
rs10869673	9	78580233	T	15	52	C	0.0006042
rs11432519	9	78587997	TA	34	25	T	0.00006745

rs2377637	9	78975569	A	16	7	G	0.0009539
rs11144853	9	78976290	C	16	7	A	0.0009539
rs11144855	9	78977568	A	16	7	G	0.0009539
rs34662513	9	78980960	CTG	27	20	C	0.0008358
rs11454957	9	78981576	TA	16	7	T	0.0009539
rs143198459	9	79101832	AAAAC	12	45	A	0.0009266
rs590666	9	79103366	T	16	53	C	0.0006806
rs503222	9	79104807	T	14	51	C	0.000328
rs608697	9	79105108	A	15	51	G	0.0006974
rs620295	9	79105317	T	15	51	C	0.0006974
rs2476940	9	79105850	T	15	51	C	0.0006974
rs2476941	9	79107595	T	15	51	C	0.0006974
rs1085341	9	79107749	T	15	51	C	0.0006974
rs664013	9	79108199	G	15	51	T	0.0006974
rs587111	9	79109110	A	15	51	G	0.0006974
rs587171	9	79109158	G	15	51	C	0.0006974
rs619429	9	79111675	A	15	51	C	0.0006974
rs634064	9	79112688	A	14	51	C	0.000328
rs566470	9	79113087	G	15	51	A	0.0006974
rs785927	9	79113497	A	15	51	T	0.0006974
rs785907	9	79121085	G	6	33	T	0.0009098
rs7026142	9	79125260	A	16	53	C	0.0006806
rs7026268	9	79125340	A	16	53	C	0.0006806
rs688268	9	79139590	T	6	35	G	0.0002764
rs582011	9	79251600	T	15	51	A	0.0006974
rs605523	9	79254447	T	15	55	G	0.00009899
rs11432533	9	79255482	A	15	55	AT	0.00009899
rs478699	9	79258395	T	14	55	C	0.00004213
rs478792	9	79258419	G	14	56	C	0.00002211
rs511040	9	79259592	C	15	58	T	0.00002069
rs512951	9	79259850	A	14	55	G	0.00004213
rs526347	9	79264147	T	15	56	C	0.0000815
rs370347624	9	79264272	TATC	16	58	T	0.00008167
rs488165	9	79312409	G	15	52	A	0.0006042
rs489069	9	79312491	C	15	52	A	0.0006042
rs684639	9	79312579	A	15	52	T	0.0006042
rs685011	9	79312627	C	15	52	G	0.0006042
rs2487263	9	79313293	A	15	52	G	0.0006042
rs519983	9	79313525	A	15	52	G	0.0006042
rs551262	9	79314660	T	15	52	A	0.0006042
rs620985	9	79318921	A	15	52	G	0.0006042

rs683866	9	79322068	G	15	52	T	0.0006042
rs2307907	9	79324135	A	15	52	ATGACTGTTGCAG	0.0006042
rs530978	9	79324179	G	15	52	A	0.0006042
rs557352	9	79324741	T	15	52	C	0.0006042
rs600859	9	79325558	G	15	52	C	0.0006042
rs626837	9	79326770	A	15	52	G	0.0006042
rs630423	9	79327585	G	15	52	A	0.0006042
rs641088	9	79327670	A	15	52	G	0.0006042
rs537903	9	79327732	T	15	52	C	0.0006042
rs571960	9	79329205	G	15	52	T	0.0006042
rs658548	9	79329257	T	15	52	C	0.0006042
rs659003	9	79329337	G	15	52	T	0.0006042
rs35230302	9	79329462	CA	15	52	C	0.0006042
rs491798	9	79330169	T	15	52	A	0.0006042
rs1854439	9	79335763	T	32	30	C	0.0009098
rs11141862	9	90139441	T	6	34	C	0.0005004
9:94981017	9	94981017	G	8	0	A	0.0003016
9:94981023	9	94981023	G	8	0	A	0.0003016
rs10987898	9	101595443	G	32	25	T	0.0004454
rs10760544	9	101596861	C	32	24	A	0.0002243
rs2416537	9	101598071	A	34	29	G	0.0006139
rs3216734	9	101599211	A	26	18	AT	0.0007809
rs12377568	9	101632069	C	16	53	G	0.0006806
rs2698999	9	102735865	A	37	34	C	0.0006983
rs2791336	9	102739446	C	37	34	G	0.0006983
rs2791337	9	102741443	C	37	32	T	0.0002611
rs7862460	9	102748888	G	37	34	C	0.0006983
rs12685650	9	102749175	G	37	34	C	0.0006983
rs10819714	9	102751188	T	37	34	C	0.0006983
rs7874449	9	102752182	A	37	34	G	0.0006983
rs10988934	9	102752686	T	37	34	C	0.0006983
rs10819715	9	102769559	C	37	34	G	0.0006983
rs2209082	9	102770884	C	37	34	T	0.0006983
rs10988945	9	102776091	T	37	34	A	0.0006983
rs7870585	9	102779098	A	37	34	G	0.0006983
rs4742779	9	102781837	C	37	33	A	0.0006234
rs1578646	9	102798031	G	37	34	A	0.0006983
rs2093696	9	102799758	A	37	34	G	0.0006983
rs35493819	9	102803899	A	37	34	G	0.0006983
rs4743379	9	102807181	A	37	34	C	0.0006983
rs4743380	9	102807279	G	37	34	A	0.0006983

rs3052013	9	102812595	C	37	34	CAT	0.0006983
rs41346545	9	102816751	A	37	34	C	0.0006983
rs4743381	9	102817835	C	37	34	T	0.0006983
rs4743382	9	102817841	T	37	34	C	0.0006983
rs942163	9	102820002	C	37	34	T	0.0006983
rs7042194	9	102821369	A	37	34	G	0.0006983
rs7027536	9	102829705	G	37	34	C	0.0006983
rs10988961	9	102829806	A	37	34	G	0.0006983
rs2900234	9	102830208	T	37	33	C	0.0006234
rs7036518	9	102839699	C	37	34	T	0.0006983
rs7047092	9	102839709	A	37	34	G	0.0006983
rs7018981	9	102842941	C	37	34	T	0.0006983
rs1342147	9	102844842	A	37	34	C	0.0006983
rs1529191	9	102845908	A	37	34	G	0.0006983
rs4517187	9	102850723	A	37	34	C	0.0006983
rs10988969	9	102852444	G	37	34	A	0.0006983
rs72548315	9	102857088	TA	37	31	T	0.000342
rs2181579	9	102863821	A	37	34	C	0.0006983
rs12552646	9	102868475	T	37	33	C	0.0006234
rs10988973	9	102869846	A	37	34	G	0.0006983
rs1539297	9	102871586	G	37	34	A	0.0006983
rs10988974	9	102871835	T	37	34	C	0.0006983
rs4743386	9	102872241	A	37	34	G	0.0006983
rs11333350	9	102911331	C	35	30	CA	0.0002569
rs1014651	9	102976644	G	37	33	A	0.0006234
rs12349610	9	109404422	A	1	19	G	0.0008417
rs12350586	9	109404541	A	1	19	G	0.0008417
rs10978580	9	109410929	T	2	22	C	0.0009583
rs10816411	9	109411613	T	2	22	C	0.0009583
rs7859930	9	109412031	T	2	22	A	0.0009583
rs10816412	9	109412764	T	2	22	G	0.0009583
rs2493636	9	109520821	A	15	51	G	0.0006974
rs80067155	9	112068534	C	7	0	G	0.0008679
rs10739293	9	113058357	G	7	36	A	0.0006639
rs17237455	9	117483259	C	3	25	A	0.0009258
rs144053768	9	117495940	CA	2	25	C	0.0001556
rs7854152	9	117497728	C	2	25	T	0.0001556
rs117204803	9	117503443	A	2	24	G	0.0002818
rs139806730	9	117522739	C	2	23	CAT	0.0005167
rs78069215	9	117524379	C	2	23	T	0.0005167
rs4978609	9	117530319	G	2	23	A	0.0005167

rs12348862	9	117531308	T	2	23	C	0.0005167
rs12335468	9	117531374	C	2	23	T	0.0005167
rs6478126	9	117770400	A	20	9	G	0.0004716
rs34952709	9	118082431	A	22	10	AT	0.00007379
rs62577508	9	118167915	C	14	5	T	0.0008508
rs72750117	9	118169815	C	14	5	A	0.0008508
rs62577526	9	118170841	T	14	5	C	0.0008508
rs62577528	9	118172545	A	14	5	G	0.0008508
rs959486	9	118178057	G	16	6	C	0.0005436
rs62577529	9	118183867	A	16	6	G	0.0005436
rs62577532	9	118195772	A	14	5	C	0.0008508
rs62576480	9	118204211	C	16	6	A	0.0005436
rs62576482	9	118213639	A	14	5	G	0.0008508
rs1335142	9	118233090	A	14	5	G	0.0008508
rs62576499	9	118233968	C	16	6	A	0.0005436
rs62576501	9	118240336	T	16	6	C	0.0005436
rs149182817	9	118240608	G	16	6	A	0.0005436
rs62576506	9	118244944	T	16	6	A	0.0005436
rs62576533	9	118264110	T	13	4	C	0.000883
rs113047360	9	118347866	T	15	5	G	0.0008419
rs10982832	9	118348955	C	18	9	T	0.0009279
rs237624	9	125396192	G	16	54	C	0.0003907
9:126708698	9	126708698	CACACACACACAC	10	0	C	0.00003497
9:126708699	9	126708699	C	10	0	A	0.00003497
9:126708703	9	126708703	ACC	8	0	A	0.0003016
rs9299438	9	136986481	A	9	1	G	0.0007194
rs73559035	9	137156676	A	19	9	G	0.0004395
rs34540368	9	138324367	T	0	16	C	0.0005294
rs3812565	9	139272502	C	12	45	T	0.0009266
rs3812573	9	139276680	T	12	46	C	0.0005428
rs10781510	9	139279173	A	12	46	G	0.0005428
rs34826348	9	139281570	C	12	46	A	0.0005428
rs10870113	9	139284790	G	12	46	A	0.0005428
10:1981876	10	1981876	ACACCTTGGTGTT	0	15	A	0.0005628
rs10903603	10	1981965	G	0	15	T	0.0005628
rs10903604	10	1981983	A	0	15	G	0.0005628
rs7096341	10	1982328	G	0	15	A	0.0005628
rs80100416	10	1985903	T	0	15	C	0.0005628
rs11250945	10	1986322	T	0	15	G	0.0005628
rs11250953	10	1991144	C	0	15	G	0.0005628
rs17157353	10	1992873	T	0	15	C	0.0005628

rs11250954	10	1993586	T	0	15	G	0.0005628
10:4454848	10	4454848	C	6	32	T	0.00098
rs2397988	10	4846866	G	12	47	T	0.0004795
rs4881348	10	4848287	A	13	47	G	0.0009845
rs4881349	10	4848290	C	13	47	A	0.0009845
rs12769532	10	4858328	C	8	38	T	0.0005059
rs2123351	10	4862582	G	8	40	A	0.0002504
rs10904350	10	4866433	T	12	46	C	0.0005428
rs2277225	10	7244675	A	7	37	G	0.0003628
rs7920088	10	7250148	T	7	37	C	0.0003628
rs61835084	10	7250882	T	7	37	C	0.0003628
rs61835085	10	7250885	T	7	37	C	0.0003628
rs955921	10	9425906	G	0	15	A	0.0005628
rs78762327	10	10750115	A	7	0	G	0.0008679
rs2479895	10	17530850	C	14	5	T	0.0008508
rs2479894	10	17531246	C	14	5	A	0.0008508
rs2226249	10	17531632	A	21	11	C	0.0003953
rs2479893	10	17532277	C	21	11	T	0.0003953
rs2039976	10	29192769	C	20	11	T	0.0008202
rs2039977	10	29192854	C	20	11	T	0.0008202
rs7084821	10	29200793	G	20	11	A	0.0008202
rs2047242	10	29201860	A	20	11	G	0.0008202
rs1756742	10	30247590	A	9	1	G	0.0007194
rs367811925	10	30962162	C	13	4	T	0.000883
rs112011604	10	39022249	AG	21	12	A	0.0009318
10:42711053	10	42711053	A	18	4	T	0.00000678
rs12570505	10	43276878	G	0	16	T	0.0005294
rs369849154	10	43327009	T	0	15	TTTTTG	0.0005628
rs72792266	10	49518130	A	10	1	G	0.0002681
rs4007215	10	56337604	A	27	11	AAATAAATG	0.000001741
rs72051960	10	56337612	G	22	7	GAATA	0.000001897
10:60153654	10	60153654	CA	10	1	C	0.0003065
rs150289992	10	65905314	T	8	0	G	0.0003016
rs201038704	10	66264592	C	7	0	CAG	0.0008679
rs192062864	10	66268556	G	7	0	A	0.0008679
rs185216213	10	66273096	A	7	0	G	0.0008679
rs74142010	10	71572026	C	7	0	T	0.0008679
rs59999447	10	71572437	T	7	0	C	0.0008679
rs57843783	10	71572490	A	7	0	G	0.0008679
rs10999537	10	72536849	T	16	7	C	0.0009539
rs827347	10	72655206	G	34	27	A	0.0002666

rs827349	10	72655450	G	12	50	A	0.00007817
rs10823635	10	72660331	T	24	12	C	0.00006466
rs146991153	10	72665149	C	11	2	CT	0.0004434
rs59844598	10	72665926	T	20	9	C	0.0002046
rs78785419	10	72666056	A	20	9	AG	0.0002046
rs827344	10	72667037	G	28	17	C	0.0000884
rs61851192	10	72668090	G	20	9	A	0.0002046
rs75488623	10	72681874	A	8	0	G	0.0003016
rs139966141	10	72692502	A	7	0	G	0.0008679
rs201841370	10	73513906	C	8	0	CT	0.0003016
rs61853694	10	73604847	T	8	0	C	0.0003016
rs61853695	10	73610125	G	8	0	T	0.0003016
rs11593281	10	73636103	T	9	1	G	0.0007194
rs116994293	10	73637359	T	9	1	C	0.0007194
rs61852188	10	73667877	A	7	0	G	0.0008679
rs61852192	10	73681619	T	7	0	C	0.0008679
10:78930285	10	78930285	C	8	0	CA	0.0003016
10:78930287	10	78930287	G	8	0	GAGGTGAGGCTCCATCCCT	0.0003016
10:78931156	10	78931156	T	10	0	TGC	0.00003497
10:82833117	10	82833117	CTTCCTTCT	1	19	C	0.0008417
10:83017314	10	83017314	C	5	31	T	0.0003749
rs11458432	10	83888508	G	23	14	GT	0.0008216
10:85688069	10	85688069	A	36	31	T	0.0005991
10:92049644	10	92049644	T	0	18	C	0.0001312
rs143771247	10	95295738	G	0	17	A	0.0002606
rs34828676	10	95815073	AT	22	11	A	0.000217
rs6583926	10	95844938	A	23	14	C	0.0007414
rs72811038	10	98008283	T	10	1	A	0.0002681
rs72811041	10	98012127	T	10	1	C	0.0002681
rs12570043	10	98052555	T	10	1	C	0.0002681
rs2253224	10	98933044	G	18	9	T	0.0009279
rs12572567	10	100991972	G	0	16	A	0.0005465
rs6584416	10	102918800	G	29	22	A	0.0007063
rs12771487	10	102924567	C	29	22	T	0.0007063
rs7082980	10	102934796	C	29	22	T	0.0007063
rs9325502	10	102951547	T	28	20	C	0.0005398
rs701839	10	102955294	A	28	20	G	0.0005398
rs7922128	10	108933871	C	0	16	T	0.0005294
rs7906243	10	108934196	A	0	16	G	0.0005294
rs12360219	10	108937557	T	0	16	C	0.0005294
rs11598667	10	108941739	T	0	16	C	0.0005294

rs12767990	10	108944867	G	0	16	A	0.0005294
rs11193241	10	108948346	C	0	16	T	0.0005294
rs17212285	10	108950581	G	0	16	A	0.0005294
rs71484305	10	108952297	C	0	16	CCT	0.0005294
rs10884432	10	108954666	T	0	16	C	0.0005294
rs11193247	10	108960595	C	0	16	G	0.0005294
rs11193248	10	108964544	T	0	16	C	0.0005294
rs10884434	10	108965240	T	0	16	C	0.0005294
rs10787015	10	108965600	C	0	16	G	0.0005294
rs10884435	10	108965649	A	0	16	G	0.0005294
rs10787016	10	108968432	A	0	16	C	0.0005294
rs12358576	10	108969037	T	0	16	G	0.0005294
rs11193253	10	108969714	T	0	16	C	0.0005294
rs11193256	10	108973730	A	0	16	G	0.0005294
rs10884438	10	108974359	A	0	16	G	0.0005294
rs11193258	10	108975552	G	0	16	A	0.0005294
rs12354523	10	108979345	T	0	16	C	0.0005294
rs11193263	10	108985888	C	0	15	T	0.0005628
rs34775254	10	108990471	T	0	15	C	0.0005628
rs35107623	10	108990498	A	0	15	G	0.0005628
rs12785180	10	108992940	T	0	15	C	0.0005628
rs12255601	10	108993226	C	0	15	T	0.0005628
rs12763192	10	108993310	A	0	15	T	0.0005628
rs12359447	10	109001176	T	0	15	C	0.0005628
rs11193273	10	109002111	G	0	15	C	0.0005628
rs35241930	10	109002677	G	0	15	A	0.0005628
rs11592478	10	109011053	C	0	15	G	0.0005628
rs11193280	10	109011463	A	0	15	G	0.0005628
rs12355630	10	109016725	T	0	15	C	0.0005628
rs74856127	10	109028018	T	0	15	A	0.0005628
rs12761509	10	109072456	T	0	16	G	0.0005294
rs111719998	10	114941343	GGCTGAGCA	8	37	G	0.0008686
rs151592	10	115716300	A	24	15	G	0.0005235
rs9421378	10	122201098	T	9	1	G	0.0006548
rs9421379	10	122201104	C	9	1	A	0.0006548
rs10829622	10	131519512	C	6	33	T	0.0009098
rs34636064	10	131519968	C	6	33	T	0.0009098
rs77183314	10	131520086	A	6	33	AGCATATTAG	0.0009098
rs10829623	10	131520689	C	6	33	T	0.0009098
rs6482752	10	131524721	G	4	30	C	0.0002486
rs13377140	10	131526249	T	4	30	A	0.0002486

rs11592158	10	131526661	A	4	30	G	0.0002486
rs7898934	10	131529320	T	4	30	C	0.0002486
10:131532754	10	131532754	G	4	30	A	0.0002486
rs11595020	10	131536734	A	4	30	G	0.0002486
rs11595599	10	131537580	A	4	30	G	0.0002486
rs34523787	10	131802518	C	4	28	T	0.0008078
rs7910987	10	131802999	T	4	28	C	0.0008078
rs7087362	10	131803211	T	4	28	C	0.0008078
rs35691464	10	131805107	T	4	28	C	0.0008078
rs2388528	10	131805763	A	4	28	G	0.0008078
11:148028	11	148028	C	7	0	T	0.0009566
rs11601758	11	200801	T	7	0	G	0.0008679
rs11604736	11	200805	C	7	0	T	0.0008679
rs370095577	11	927257	T	20	10	G	0.0005561
rs76282688	11	4587440	G	0	16	A	0.0005294
rs16933455	11	7910939	C	7	0	T	0.0008679
rs17227935	11	8262750	C	25	15	T	0.0002543
rs34494077	11	8270544	A	26	15	T	0.0001216
rs1598165	11	8274248	A	26	18	G	0.0007809
rs11041827	11	8274699	T	15	53	C	0.0003298
rs4256972	11	8275004	T	15	53	C	0.0003298
rs11041828	11	8275294	T	15	53	C	0.0003298
rs4237770	11	8275835	C	15	53	T	0.0003298
rs369776490	11	8276156	T	15	53	TATGGTCCCTCCCCACC	0.0003298
rs373931277	11	8276174	A	15	53	AGGCTGGC	0.0003298
rs149239827	11	8276184	T	15	53	TGG	0.0003298
rs1454439	11	8276219	C	15	53	A	0.0003298
rs4758055	11	8277435	C	18	57	G	0.0003261
rs12802521	11	8277575	G	18	58	T	0.000347
rs12797411	11	8277594	G	18	59	A	0.0001874
rs71463719	11	8277624	G	38	33	A	0.0001874
rs11041830	11	8277807	A	38	33	C	0.0001874
rs3838787	11	8280679	CG	27	19	C	0.0008555
rs72852579	11	8282711	C	31	25	A	0.0008555
rs10833089	11	19310745	A	8	39	C	0.0004628
rs4423178	11	20626318	A	6	32	G	0.00098
rs2665704	11	22408655	G	11	44	A	0.0007768
rs2665705	11	22408803	T	11	44	C	0.0007768
rs721840	11	22411865	C	11	44	T	0.0007768
rs721841	11	22411969	C	11	44	G	0.0007768
rs2593649	11	22413033	T	11	44	C	0.0007768

rs2665712	11	22414861	C	11	44	G	0.0007768
rs2665713	11	22417107	A	11	44	G	0.0007768
rs1447504	11	22417712	A	11	44	T	0.0007768
rs726858	11	22418220	T	11	44	G	0.0007768
rs726859	11	22418570	A	11	44	G	0.0007768
rs2593651	11	22420611	G	11	44	A	0.0007768
rs2593652	11	22421015	T	11	44	C	0.0007768
rs2593653	11	22422503	G	10	44	C	0.000219
rs111974052	11	22423006	A	11	44	AATGACTATTTG	0.0007768
rs2593657	11	22423166	T	11	44	C	0.0007768
rs35078276	11	22423689	C	11	44	T	0.0007768
rs34754498	11	22423691	G	11	44	T	0.0007768
rs34550272	11	22423693	C	11	44	T	0.0007768
rs4923000	11	22423752	A	11	44	C	0.0007768
rs2593660	11	22424782	C	11	45	A	0.0004315
rs5790261	11	22425340	TG	10	44	T	0.000219
rs2593663	11	22428384	C	11	44	T	0.0007768
rs2665718	11	22428882	G	11	44	A	0.0007768
rs3106185	11	22435111	C	11	44	A	0.0007768
rs380241	11	22696692	T	14	5	C	0.0008508
rs380563	11	22701195	G	14	5	A	0.0008508
rs2928340	11	22703955	T	14	5	A	0.0008508
rs200967939	11	26518574	G	8	37	C	0.0008686
rs5020951	11	47610273	A	31	24	T	0.0008755
rs7936042	11	48344136	T	17	7	C	0.0004382
rs4450174	11	48345535	T	20	11	C	0.0008202
rs7395561	11	48359930	A	20	11	G	0.0008202
rs7396789	11	48359978	T	20	11	G	0.0008202
rs111349704	11	48361184	C	20	11	T	0.0008202
rs75036683	11	48361774	A	20	11	T	0.0008202
11:49263077	11	49263077	A	17	7	ATATATGTATATATATGTAT	0.0005099
rs34701823	11	49263199	G	17	7	A	0.0003336
rs10839247	11	49284326	C	24	15	G	0.0005235
rs10839270	11	49387337	C	18	9	A	0.0009279
rs7113355	11	49394560	T	18	9	C	0.0009279
rs4297465	11	49397079	C	18	9	G	0.0009279
rs10839271	11	49398826	A	18	9	G	0.0009279
rs58025312	11	49400534	T	18	9	A	0.0009279
rs113728976	11	49405469	T	18	9	A	0.0009279
rs74462122	11	49405607	G	18	9	A	0.0009279
rs2091206	11	49407508	A	18	9	G	0.0009279

rs10769576	11	49410203	A	18	9	G	0.0009279
rs4109834	11	49413046	A	18	9	G	0.0009279
rs12294396	11	49418158	G	18	9	T	0.0009279
rs60691122	11	49424706	G	18	9	A	0.0009279
rs56006436	11	49424902	T	18	9	G	0.0009279
rs11040359	11	49437005	T	18	9	G	0.0009279
rs7926211	11	49438332	T	19	9	C	0.0004395
rs113869127	11	49443122	GA	18	9	G	0.0009279
rs35797587	11	49445135	GA	18	9	G	0.0009279
rs7118273	11	49446564	C	18	9	A	0.0009279
rs11040364	11	49447342	T	18	9	C	0.0009279
rs7358352	11	49447850	C	18	9	T	0.0009279
rs7924702	11	49450356	C	18	9	A	0.0009279
rs61278589	11	49450916	GT	18	9	G	0.0009279
rs10839279	11	49451370	C	18	9	G	0.0009279
rs7108400	11	49456896	G	18	9	A	0.0009279
rs7104119	11	49463162	G	18	9	A	0.0009279
rs74237315	11	49464951	T	18	8	C	0.0006346
rs11040375	11	49470089	A	18	9	T	0.0009279
rs10839280	11	49481319	G	18	9	A	0.0009279
rs78275063	11	49491771	C	18	9	T	0.0009279
rs11040392	11	49505175	T	18	9	C	0.0009279
rs10839288	11	49506590	G	18	9	C	0.0009279
rs3862347	11	49517160	T	18	9	C	0.0009279
rs28627422	11	49517955	T	18	9	C	0.0009279
rs5791912	11	49519012	A	18	9	AT	0.0009279
rs7931159	11	49521758	T	18	9	G	0.0009279
rs7113589	11	49523244	C	18	9	G	0.0009279
rs10839293	11	49525436	A	18	9	G	0.0009279
rs148363829	11	49533795	C	18	9	CAT	0.0009279
rs7123475	11	49534164	G	18	9	T	0.0009279
rs11040409	11	49535138	T	18	9	C	0.0009279
rs75971566	11	49550581	A	18	9	G	0.0009279
rs1827012	11	49555761	G	18	9	T	0.0009279
rs10839298	11	49569382	A	19	9	G	0.0004395
rs11040424	11	49573704	T	18	9	C	0.0009279
rs10839300	11	49577519	T	18	9	A	0.0009279
rs7108779	11	49582085	T	18	9	G	0.0009279
rs10839303	11	49582462	T	18	9	G	0.0009279
rs10769589	11	49582504	T	18	9	C	0.0009279
rs10769590	11	49585158	C	18	9	A	0.0009279

rs11420458	11	49606837	TA	18	9	T	0.0009279
rs28498099	11	49607490	C	18	9	T	0.0009279
rs3974531	11	49610094	T	18	9	G	0.0009279
rs10839308	11	49614443	A	18	9	G	0.0009279
rs7925397	11	49615516	C	18	9	A	0.0009279
rs7926115	11	49616011	G	18	9	C	0.0009279
rs10769594	11	49616766	T	18	9	G	0.0009279
rs7123089	11	49618468	T	18	9	C	0.0009279
rs7117720	11	49629919	G	18	9	A	0.0009279
rs28478434	11	49637858	C	18	9	T	0.0009279
rs56397807	11	49650748	C	18	9	T	0.0009279
rs11040454	11	49667123	C	18	9	T	0.0009279
rs1508692	11	49670255	C	18	9	A	0.0009279
rs12223469	11	49675012	T	18	9	C	0.0009279
rs145099871	11	49684587	A	18	9	AAAGAGTGTG	0.0009279
rs10769602	11	49688172	T	18	9	A	0.0009279
rs7107310	11	49690812	C	18	9	T	0.0009279
rs6486003	11	49693773	A	18	9	T	0.0009279
rs12224222	11	49699461	G	23	12	A	0.0002434
rs75789447	11	49706844	A	23	12	G	0.0002434
rs11040475	11	49709472	A	23	12	G	0.0002434
rs111343904	11	49719799	AT	25	12	A	0.00003495
rs10769606	11	49721909	T	25	13	C	0.0000842
rs75884431	11	49723814	A	25	12	G	0.00003495
rs6486007	11	49725128	A	25	12	G	0.00003495
rs7934809	11	49729155	A	25	12	G	0.00003495
rs200365936	11	49732793	A	25	12	T	0.00003495
rs201030732	11	49732796	C	25	12	G	0.00003495
rs202076125	11	49732797	C	25	12	CTTGTGGA	0.00003495
rs202013769	11	49732805	C	25	12	A	0.00003495
rs11040486	11	49734673	A	25	12	C	0.00003495
rs200584985	11	49738321	T	25	12	TG	0.00003495
11:49738324	11	49738324	A	25	12	AT	0.00003495
11:49738328	11	49738328	C	25	12	CT	0.00003495
11:49738330	11	49738330	CTAGAA	25	12	C	0.00003495
11:49738332	11	49738332	A	25	12	C	0.00003495
rs10839344	11	49745879	T	25	12	C	0.00003495
rs7478971	11	49747444	C	25	12	T	0.00003495
rs201448975	11	49750010	G	25	12	A	0.00003495
rs11040495	11	49751202	G	25	12	A	0.00003495
rs9735717	11	49752014	T	20	10	G	0.0005977

rs11040502	11	49754372	G	25	12	A	0.00003495
rs10839350	11	49755815	C	25	12	T	0.00003495
rs11040503	11	49760350	A	25	13	C	0.0001716
rs10839355	11	49764142	A	23	12	G	0.0002434
rs7929466	11	49764501	T	23	12	C	0.0002434
rs7930031	11	49764744	A	23	12	G	0.0002434
rs7940219	11	49769264	C	23	12	T	0.0002434
rs10839357	11	49769993	A	23	12	G	0.0002434
rs7126006	11	49770456	G	23	12	T	0.0002434
rs7342231	11	49772761	G	22	11	A	0.0001874
rs11040511	11	49775247	T	23	12	A	0.0002434
rs76075584	11	49776343	C	23	12	CT	0.0002434
rs9633834	11	49780784	G	23	12	T	0.0002434
rs7938664	11	49784497	G	23	12	A	0.0002434
rs7943827	11	49786202	A	23	12	G	0.0002434
rs7928365	11	49790540	A	23	12	T	0.0002434
rs7926022	11	49793884	A	23	12	G	0.0002434
rs11040520	11	49797641	A	22	10	G	0.00005982
rs200455225	11	49798676	T	23	12	C	0.0002434
rs200690573	11	49799511	A	20	10	G	0.0005561
rs10839373	11	49802697	G	23	12	C	0.0002434
rs10839374	11	49802707	C	23	12	T	0.0002434
rs10839376	11	49802835	A	23	12	T	0.0002434
rs12225361	11	49811416	C	23	12	T	0.0002434
rs368958306	11	49819744	T	24	12	G	0.00006466
rs11040544	11	49821636	T	23	12	G	0.0002434
rs7937581	11	49824449	G	23	12	A	0.0002434
rs7115159	11	49829781	A	23	12	G	0.0002434
rs3974371	11	49837553	A	22	12	G	0.000477
rs3974437	11	49838800	C	23	12	T	0.0002434
rs3974438	11	49838802	C	23	12	G	0.0002434
rs7927743	11	49839800	A	23	12	G	0.0002434
rs11828963	11	49840036	C	23	12	G	0.0002434
rs7113158	11	49841717	T	23	12	C	0.0002434
rs7121847	11	49843560	A	23	12	G	0.0002434
rs7122262	11	49844121	T	23	13	C	0.0003278
rs10839393	11	49844505	T	21	12	G	0.0009318
rs7130289	11	49845685	T	23	12	C	0.0002434
rs7120100	11	49848506	C	23	12	A	0.0002434
rs7929167	11	49857885	C	23	12	G	0.0002434
rs10839398	11	49860044	G	23	12	A	0.0002434

rs11040588	11	49860762	A	23	12	G	0.0002434
rs11040591	11	49864459	A	23	12	G	0.0002434
rs7932861	11	49865897	A	23	12	G	0.0002434
rs10160231	11	49866908	C	23	12	T	0.0002434
rs10160234	11	49867055	C	23	12	T	0.0002434
rs7925690	11	49872449	G	23	12	T	0.0002434
rs28580349	11	49873632	G	23	12	C	0.0002434
rs12222826	11	49873758	A	23	11	G	0.00008787
rs10839400	11	49874567	A	23	12	G	0.0002434
rs7130010	11	49875570	G	23	12	C	0.0002434
rs7130624	11	49875845	A	23	12	G	0.0002434
rs7395782	11	49878353	C	23	12	G	0.0002434
rs7925632	11	49878419	A	23	12	T	0.0002434
rs7926023	11	49881879	T	23	12	C	0.0002434
rs10769629	11	49882682	A	23	12	G	0.0002539
rs73466972	11	49884317	T	23	12	G	0.0002434
rs10839410	11	49884692	A	23	12	G	0.0002434
rs10839411	11	49886623	A	23	12	G	0.0002434
rs10839412	11	49886667	G	23	12	A	0.0002434
rs200523127	11	49896280	A	23	12	G	0.0002434
rs7947702	11	49897846	T	23	12	C	0.0002434
rs11040614	11	49898849	T	23	12	C	0.0002434
rs12270672	11	49900067	T	23	12	C	0.0002434
rs10839421	11	49900489	C	23	12	G	0.0002434
rs10839423	11	49901708	G	23	12	T	0.0002434
rs73467002	11	49905020	G	23	12	A	0.0002434
rs10839427	11	49905764	G	23	12	C	0.0002434
rs145109916	11	49908519	T	23	12	G	0.0002434
rs12284792	11	49909416	G	23	12	T	0.0002434
rs60299751	11	49911267	G	23	12	A	0.0002434
rs56258327	11	49911401	T	23	12	A	0.0002434
rs7121372	11	49914210	C	23	12	G	0.0002434
rs7937552	11	49916242	C	23	12	T	0.0002434
rs10734604	11	49923034	T	22	11	C	0.0001529
rs2003143	11	49923509	T	23	12	C	0.0002434
rs2003144	11	49923744	T	23	12	C	0.0002434
rs10742956	11	49925909	A	23	12	G	0.0002434
rs57549473	11	49930116	T	23	12	TA	0.0002434
rs11040645	11	49933282	A	23	12	C	0.0002434
rs10839447	11	49934353	C	23	12	T	0.0002434
rs9705134	11	49934901	A	23	12	G	0.0002434

rs10734605	11	49936323	A	23	12	T	0.0002434
rs4474434	11	49936666	G	23	12	A	0.0002434
rs4628673	11	49937579	C	23	12	A	0.0002434
rs7125437	11	49937971	C	23	12	T	0.0002434
rs6486038	11	49946299	T	23	12	C	0.0002434
rs4109780	11	49948091	T	23	12	C	0.0002434
rs10734607	11	49950098	C	23	12	A	0.0002434
rs10742959	11	49956951	A	23	12	T	0.0002434
rs10742960	11	49956953	G	23	12	C	0.0002434
rs6486041	11	49962843	A	23	12	G	0.0002434
rs7129763	11	49969219	C	23	12	T	0.0002434
rs7102734	11	49969469	C	23	12	A	0.0002434
rs7102618	11	49969473	A	23	12	G	0.0002434
rs11040675	11	49969615	T	23	12	C	0.0002434
rs11601773	11	49978958	C	23	12	A	0.0001164
rs10839466	11	49981669	A	23	12	G	0.0002434
rs1818888	11	49987537	T	23	12	C	0.0002434
rs4378400	11	49993317	C	23	13	A	0.0003278
rs7483015	11	49998023	A	22	12	G	0.000477
rs10769647	11	50002275	T	23	12	C	0.0002434
rs66513186	11	50006407	T	23	12	TA	0.0002434
rs10742963	11	50010007	G	23	12	T	0.0002434
rs4242812	11	50010908	C	23	12	T	0.0002434
rs7483824	11	50024530	T	23	12	C	0.0002434
rs4264152	11	50025257	T	23	12	C	0.0002434
rs10769649	11	50028341	T	23	12	A	0.0002434
rs7125112	11	50032877	A	23	12	G	0.0002434
rs4509737	11	50033896	T	23	12	A	0.0002434
rs28862975	11	50044480	A	23	12	G	0.0002434
rs28616719	11	50048663	T	24	12	G	0.00006466
rs28491498	11	50052422	A	23	12	T	0.0002434
rs28452864	11	50055245	G	23	12	A	0.0002434
rs7106938	11	50062925	A	23	12	T	0.0002434
rs200652008	11	50064702	CA	22	11	C	0.0001874
rs7939285	11	50065753	A	23	12	G	0.0002434
rs12224327	11	50068838	T	23	12	C	0.0002434
rs1390677	11	50070332	T	23	12	G	0.0002434
rs202088682	11	50093897	A	21	10	G	0.000281
rs7932015	11	50094879	T	23	12	C	0.0002434
rs2201636	11	50095742	T	23	12	G	0.0002434
rs28651975	11	50096293	T	23	12	C	0.0002434

rs12273546	11	50101348	T	23	12	G	0.0002434
rs4082585	11	50104726	T	23	12	G	0.0002434
rs12282428	11	50105543	A	23	12	G	0.0002434
rs10901984	11	50105783	G	23	12	A	0.0002434
rs7104310	11	50106409	T	23	12	C	0.0002434
rs7101532	11	50106487	G	23	12	A	0.0002434
rs6597938	11	50108289	G	23	12	C	0.0002434
rs7944772	11	50111674	T	23	12	A	0.0002434
rs4551759	11	50112158	T	23	12	C	0.0002434
rs11245791	11	50114708	G	23	12	A	0.0002434
rs6597940	11	50116278	C	23	12	T	0.0002434
rs1845858	11	50116583	A	23	12	G	0.0002434
rs1496467	11	50117620	C	23	12	T	0.0002434
rs7119707	11	50118197	C	23	12	T	0.0002434
rs12223378	11	50119077	C	23	12	T	0.0002434
rs7949346	11	50122862	C	23	12	G	0.0002434
rs7129932	11	50125428	C	23	12	A	0.0002434
rs7938918	11	50128413	C	23	12	A	0.0002434
rs7104653	11	50129598	A	23	12	G	0.0002434
rs74603331	11	50130690	A	23	12	ATATAT	0.0002434
rs7939306	11	50132363	T	23	12	C	0.0002434
rs10902066	11	50134037	G	23	12	C	0.0002434
rs11245902	11	50137461	A	23	12	G	0.0002434
rs7119765	11	50141419	T	23	12	A	0.0002434
rs7115169	11	50143774	T	23	12	C	0.0002434
rs112916529	11	50145989	TGCAC	23	12	T	0.0002434
rs2006896	11	50147183	A	23	12	G	0.0002434
rs7935868	11	50148729	T	23	12	C	0.0002434
rs10794275	11	50154408	A	23	12	G	0.0002434
rs10902071	11	50154754	G	23	12	A	0.0002434
rs11245915	11	50155636	C	23	12	G	0.0002434
rs10901885	11	50165376	A	23	12	G	0.0002434
rs6597888	11	50166161	A	23	11	G	0.00009985
rs10794212	11	50171877	C	23	12	T	0.0002434
rs7951692	11	50175386	T	23	12	C	0.0002434
rs12271034	11	50179838	G	23	12	A	0.0002434
rs139099319	11	50180779	CTTAT	23	12	C	0.0002434
rs36104516	11	50187374	C	23	12	CA	0.0002434
rs7945981	11	50189879	G	24	11	A	0.00002041
rs12281164	11	50192243	T	23	12	C	0.0002434
rs78067132	11	50200440	A	23	12	G	0.0002434

rs7117400	11	50204466	A	23	12	G	0.0002434
rs6597891	11	50205579	G	23	12	A	0.0002434
rs6597892	11	50206408	A	23	12	G	0.0002434
rs7129988	11	50208668	T	23	12	A	0.0002434
rs12270832	11	50209577	T	23	12	A	0.0002434
rs61317280	11	50210996	G	23	12	C	0.0002434
rs12274911	11	50212299	G	24	12	A	0.00006466
rs12276637	11	50212313	A	24	12	T	0.00006466
rs10794216	11	50216041	T	23	12	G	0.0002434
rs7941169	11	50217658	C	23	12	T	0.0002434
rs1826833	11	50219823	T	23	12	C	0.0002434
rs7952693	11	50225493	A	23	12	G	0.0002434
rs7926715	11	50228759	T	23	12	C	0.0002434
rs10901906	11	50229266	C	23	12	T	0.0002434
rs6597897	11	50229835	A	23	12	T	0.0002434
rs7126773	11	50229982	A	23	12	G	0.0002434
rs12292034	11	50231436	C	23	12	A	0.0002434
rs76509416	11	50232059	A	23	12	G	0.0002434
rs143260220	11	50233427	AC	23	12	A	0.0002434
rs7101625	11	50248534	A	23	12	G	0.0002434
rs7102961	11	50252272	G	23	12	A	0.0002434
rs80243738	11	50253401	A	23	14	ATTC	0.0007414
rs3829060	11	50257732	C	23	12	T	0.0002434
rs12222099	11	50260871	A	23	12	G	0.0002434
rs6597899	11	50262926	G	23	12	A	0.0002434
rs12224914	11	50270503	T	23	12	C	0.0002434
rs148166088	11	50272145	T	18	9	C	0.0009279
rs11245630	11	50279053	A	23	12	G	0.0002434
rs11245631	11	50279561	T	24	14	C	0.0003806
rs10901911	11	50282774	T	23	12	C	0.0002434
rs7101411	11	50289225	C	23	12	G	0.0002434
rs7943523	11	50295566	C	23	12	T	0.0002434
rs4111483	11	50298232	G	23	12	A	0.0002434
rs7104183	11	50302343	A	23	12	G	0.0002434
rs199523190	11	50304508	GT	21	11	G	0.0003953
rs11502001	11	50324829	A	23	12	G	0.0002434
rs3904258	11	50327748	A	23	12	T	0.0002434
rs7938813	11	50328033	A	23	12	G	0.0002434
rs3904274	11	50329862	A	23	12	G	0.0002434
rs56258046	11	50331203	A	23	12	T	0.0002434
rs7942986	11	50331809	C	23	12	A	0.0002434

rs11502003	11	50341483	A	23	12	G	0.0002434
rs11502004	11	50341671	A	23	12	G	0.0002434
rs61319414	11	50345819	A	23	12	C	0.0002434
rs77639753	11	50347898	A	23	12	T	0.0002434
rs11530897	11	50348489	G	23	12	A	0.0002434
rs12273168	11	50350886	T	23	12	G	0.0002434
rs7117501	11	50352564	A	23	12	G	0.0002434
rs7480651	11	50355829	G	23	13	C	0.0003278
rs4097537	11	50359470	A	23	13	G	0.0003278
rs7130223	11	50361056	C	23	13	T	0.0003278
rs7120506	11	50361085	T	23	13	C	0.0003278
rs12274688	11	50362077	T	23	13	G	0.0003278
rs3912710	11	50364862	T	18	9	C	0.0009279
rs7483219	11	50365404	A	23	13	G	0.0003278
rs7483817	11	50365440	A	23	13	G	0.0003278
rs11245655	11	50371570	T	23	12	C	0.0002434
rs3866801	11	50374138	G	23	12	A	0.0002434
rs11245661	11	50386489	T	23	12	A	0.0002434
rs11245662	11	50388103	G	23	12	A	0.0002434
rs7293950	11	50392698	T	23	12	A	0.0002434
rs8189401	11	50395952	A	23	12	G	0.0002434
rs79147867	11	50400993	GT	23	12	G	0.0002434
rs8189134	11	50407583	T	23	12	C	0.0002434
rs12288415	11	50409948	A	23	12	G	0.0002434
rs12293696	11	50412498	A	23	12	G	0.0002434
rs113829766	11	50418356	A	23	12	T	0.0002434
rs7938137	11	50424591	C	23	12	T	0.0002434
rs7933409	11	50434166	A	24	12	G	0.00006466
rs11537283	11	50437542	G	23	12	C	0.0002434
rs2313609	11	50439113	A	21	12	C	0.0009318
rs113944228	11	50441095	A	21	12	ATTTC	0.0009318
rs6597926	11	50444220	C	23	12	T	0.0002434
rs11245689	11	50451116	A	21	12	G	0.0009318
rs11245692	11	50451337	T	21	12	G	0.0009318
rs10901952	11	50452283	C	23	12	A	0.0002434
rs77340754	11	50453047	A	21	12	C	0.0009318
rs73470296	11	50455067	T	21	12	A	0.0009318
rs12422197	11	50456487	T	21	12	C	0.0009318
rs113623252	11	50457940	T	21	12	A	0.0009318
rs11533317	11	50458825	C	23	12	T	0.0002434
rs10901955	11	50459288	C	23	12	T	0.0002434

rs7293940	11	50459908	C	21	12	G	0.0009318
rs11245700	11	50460517	T	23	12	C	0.0002539
rs11245702	11	50462967	C	21	12	T	0.0009318
rs7294136	11	50464201	C	23	12	G	0.0002434
rs8189003	11	50465834	A	21	12	C	0.0009318
rs75618991	11	50468157	A	21	12	AC	0.0009318
rs1589629	11	50474459	T	21	12	C	0.0009318
rs7294276	11	50475856	T	21	12	C	0.0009318
rs11532134	11	50483601	A	21	12	G	0.0009318
rs10901963	11	50488853	T	21	12	C	0.0009318
rs1608272	11	50490256	A	21	12	T	0.0009318
rs10901965	11	50496188	T	21	12	C	0.0009318
rs7947077	11	50497585	T	21	12	C	0.0009318
rs12282218	11	50498647	T	23	12	C	0.0002434
rs1608276	11	50503949	A	21	12	G	0.0009318
rs11245732	11	50507774	A	21	12	C	0.0009318
rs11825447	11	50508744	A	21	12	G	0.0009318
rs4631910	11	50510243	T	21	12	C	0.0009318
rs2204185	11	50510403	G	21	12	T	0.0009318
rs11245735	11	50513030	G	21	12	T	0.0009318
rs2313661	11	50513441	C	21	12	G	0.0009318
rs7294023	11	50513544	T	21	12	C	0.0009318
rs12275650	11	50517487	C	21	12	T	0.0009318
rs10794249	11	50519296	C	21	12	A	0.0009318
rs10901970	11	50520259	C	21	12	A	0.0009318
rs8189234	11	50526569	T	21	12	C	0.0009318
rs11245749	11	50540697	G	21	12	A	0.0009318
rs8189030	11	50543925	T	21	12	C	0.0009318
rs8186351	11	50544072	A	21	12	G	0.0009318
rs4472965	11	50547599	C	21	12	G	0.0009318
rs11245754	11	50548882	C	21	12	T	0.0009318
rs11535886	11	50558437	G	22	12	A	0.000477
rs7293930	11	50562142	C	21	12	T	0.0009318
rs8189213	11	50566719	T	21	12	C	0.0009318
rs11537269	11	50577906	G	21	12	T	0.0009318
rs8186556	11	50579584	A	23	12	C	0.0002539
rs59058603	11	50581369	C	21	12	CT	0.0009318
rs11245773	11	50582317	G	21	12	C	0.0009318
rs60441611	11	50582908	A	21	12	C	0.0009318
rs1592602	11	50602567	C	22	13	T	0.0006749
rs1833272	11	50602940	C	22	13	A	0.0006749

rs12272441	11	50605469 T	21	12 C	0.0009318
rs3919555	11	50620415 T	22	12 G	0.000477
rs111766585	11	50620511 C	21	12 CA	0.0009318
rs1118132	11	50621548 T	21	12 C	0.0009318
rs1833261	11	50629395 G	21	12 A	0.0009318
rs12287190	11	50631738 T	21	12 C	0.0009318
rs11245790	11	50638617 T	21	12 G	0.0009318
rs4980483	11	50649709 T	21	12 C	0.0009318
rs4980456	11	50650534 T	21	12 C	0.0009318
rs2872642	11	50652890 G	21	12 A	0.0009318
rs11245798	11	50653895 A	21	12 G	0.0009318
rs7294275	11	50653980 T	21	12 C	0.0009318
rs11245799	11	50654134 T	21	12 C	0.0009318
rs10902019	11	50654339 A	21	12 G	0.0009318
rs11561033	11	50657803 C	21	12 G	0.0009318
rs144300519	11	50658518 A	21	12 AT	0.0009318
rs10431185	11	50659828 C	21	12 T	0.0009318
rs11245809	11	50663560 T	21	12 C	0.0009318
rs2313933	11	50667351 T	21	12 C	0.0009318
rs11823709	11	50668546 T	21	12 C	0.0009318
rs111910431	11	50670939 C	21	12 CAA	0.0009318
rs7294196	11	50670949 A	21	12 G	0.0009318
rs142814862	11	50682185 A	21	12 ATTG	0.0009318
rs2216276	11	50685730 A	21	12 G	0.0009318
rs7294008	11	50687904 G	21	12 A	0.0009318
rs8188960	11	50688586 T	21	12 C	0.0009318
rs8188971	11	50696175 A	23	12 C	0.0002539
rs8189359	11	50699030 A	23	12 C	0.0002539
rs73468069	11	50708031 A	21	12 C	0.0009318
rs12288231	11	50708208 T	21	12 C	0.0009318
rs111348362	11	50708654 C	21	12 CTG	0.0009318
rs7294286	11	50710459 T	21	12 A	0.0009318
rs7925215	11	50727606 A	21	12 T	0.0009318
rs11501991	11	50732728 A	21	12 T	0.0009318
rs7944041	11	50734530 G	21	12 C	0.0009318
rs34813929	11	50738430 A	21	12 AT	0.0009318
rs11245860	11	50740226 T	21	12 C	0.0008432
rs11245864	11	50748677 A	21	12 G	0.0009318
rs10902053	11	50751880 C	22	12 T	0.000477
rs11245872	11	50752748 G	22	12 T	0.000477
rs11245875	11	50754477 A	22	12 G	0.000477

rs11245876	11	50755041	T	22	12	C	0.000477
rs11245878	11	50756950	C	22	12	T	0.000477
rs7293994	11	50757564	C	22	12	A	0.000477
rs9634039	11	50761263	T	21	12	C	0.0009318
rs61657582	11	50762942	CT	21	12	C	0.0009318
rs11245880	11	50764078	A	21	12	G	0.0009318
rs10902061	11	50766866	T	21	12	A	0.0009318
rs8189341	11	50769125	A	21	12	C	0.0009318
rs11533307	11	50773906	T	21	12	C	0.0009318
rs11561040	11	50774090	C	21	12	G	0.0009318
rs11245891	11	50774654	T	21	12	G	0.0009318
rs193019049	11	50777681	G	21	12	A	0.0009318
rs12293802	11	50779877	T	21	12	A	0.0009318
rs10902323	11	51313521	A	21	12	C	0.0009318
rs1603755	11	51314373	G	21	12	C	0.0009318
rs10902325	11	51323008	C	21	12	A	0.0009318
rs10902328	11	51327845	C	21	12	G	0.0009318
rs7294263	11	51339825	G	21	12	A	0.0009318
rs1783974	11	51376313	A	21	12	G	0.0009318
rs544743	11	51384161	A	21	12	G	0.0009318
rs568295	11	51388790	G	21	12	T	0.0009318
rs503846	11	51393313	T	21	12	A	0.0009318
rs1620679	11	51407874	T	21	12	C	0.0009318
rs480014	11	51417899	T	22	12	G	0.000477
rs508912	11	51418789	T	21	12	A	0.0009318
rs527464	11	51440610	C	21	12	T	0.0009318
rs505040	11	51443639	A	22	12	G	0.000477
rs505039	11	51443640	A	22	12	G	0.000477
rs479106	11	51444130	A	21	12	G	0.0009318
rs496572	11	51456554	C	21	12	T	0.0009318
rs510517	11	51460443	T	21	12	C	0.0009318
rs530655	11	51469977	T	21	12	C	0.0009318
rs10794391	11	51472652	C	21	12	T	0.0009318
rs36122694	11	51478967	A	21	12	AC	0.0009318
rs5791938	11	51486517	AG	21	12	A	0.0009318
rs4881829	11	51492274	T	21	12	C	0.0009318
rs7478797	11	51494224	A	21	12	T	0.0009318
rs7951522	11	51495019	C	21	12	G	0.0009318
rs7480344	11	51495569	T	21	12	A	0.0009318
rs6598116	11	51502234	T	21	12	A	0.0009318
rs7127896	11	51519416	C	21	12	T	0.0009318

rs7107202	11	51519430	T	21	12 A	0.0009318
rs10794398	11	51538073	G	21	12 A	0.0009318
rs7480941	11	51539692	A	20	12 C	0.0008157
rs4881864	11	51554380	G	21	12 A	0.0009318
rs4881865	11	51554552	A	21	12 T	0.0009318
rs10902378	11	51555757	A	21	12 C	0.0009318
rs77665545	11	51556448	T	21	12 A	0.0009318
rs10902385	11	51575951	A	21	12 G	0.0009318
rs4881874	11	51579888	G	23	13 A	0.0002775
rs9795278	11	54890653	A	23	15 G	0.0007412
rs10896808	11	58421179	C	33	28 T	0.0009909
rs12420131	11	61848276	C	7	0 A	0.0008679
rs10897275	11	62203865	A	31	21 G	0.00008418
rs9645690	11	62206288	T	31	21 C	0.00008418
rs59665939	11	62207685	G	31	25 C	0.0008555
rs56299751	11	62208039	A	30	23 G	0.0007082
rs11231093	11	62210822	A	29	21 G	0.0006145
rs10897278	11	62213068	A	30	23 G	0.0007082
rs12805216	11	62214363	A	33	26 G	0.0002784
rs12802176	11	64806701	A	35	33 T	0.0005003
rs7932004	11	66689915	A	7	39 C	0.0001101
rs7110302	11	66690454	T	7	39 C	0.0001101
11:71910931	11	71910931	T	26	20 TGTG	0.000739
rs10898940	11	73455292	C	16	53 A	0.0006806
rs12222811	11	81703344	C	7	0 T	0.0008679
11:83019686	11	83019686	G	26	10 A	0.000002426
11:83019706	11	83019706	T	22	8 A	0.00001321
rs57719262	11	87207380	A	8	0 G	0.0003016
rs79744783	11	87211218	T	7	0 C	0.0008679
11:87337925	11	87337925	C	7	0 A	0.0008679
rs117152419	11	87362825	G	7	0 A	0.0008679
rs308788	11	88285408	C	0	19 T	0.00006755
rs308787	11	88285704	C	0	19 T	0.00006755
rs308786	11	88285887	G	0	19 A	0.00006755
rs308785	11	88286031	G	0	19 T	0.00006755
rs308763	11	88289360	A	0	18 G	0.0001312
rs16914244	11	88295675	G	0	16 T	0.0005294
rs308872	11	88295873	G	0	18 C	0.0001312
rs308874	11	88297066	T	0	18 C	0.0001312
rs7949902	11	88297748	C	0	16 T	0.0005294
rs60953957	11	88308583	A	0	16 ATTTAT	0.0005294

rs10831094	11	88311037	T	0	17	G	0.0002606
rs11821168	11	88312675	C	0	17	G	0.0002606
rs11020513	11	88313217	T	0	17	C	0.0002606
rs3913664	11	88313677	T	0	16	C	0.0005294
rs7944148	11	88315106	G	0	17	A	0.0002606
rs11826037	11	88316083	G	0	17	A	0.0002606
11:92106942	11	92106942	G	7	0	A	0.0008679
11:95398394	11	95398394	C	8	0	T	0.0003016
rs117615763	11	96175310	C	9	1	G	0.0007194
rs75830241	11	96177533	G	9	1	A	0.0007194
rs79010680	11	96190756	A	9	1	C	0.0007194
rs140040266	11	96223824	T	9	1	A	0.0007194
rs11217075	11	96678998	A	13	47	G	0.0009845
rs35274850	11	96703332	A	18	8	T	0.0005897
rs12807861	11	96706992	C	18	8	G	0.0005897
rs1452974	11	96709499	T	18	8	A	0.0005897
rs1452976	11	96709583	G	18	8	C	0.0005897
rs35541412	11	96710432	C	18	8	G	0.0005897
rs34043458	11	96712892	T	18	8	C	0.0005897
rs72959017	11	96713783	A	18	8	C	0.0005897
rs55996850	11	96714195	G	18	8	T	0.0005897
rs66512518	11	96715396	C	18	8	T	0.0005897
rs55926807	11	96728216	T	18	8	G	0.0005897
rs12795826	11	96729813	C	18	8	G	0.0005897
rs2218990	11	96732368	T	18	8	C	0.0005897
rs35440310	11	96734651	T	18	8	A	0.0005897
rs11219720	11	96736172	C	20	11	G	0.0008202
rs1159532	11	96738190	A	18	8	G	0.0005897
rs67365311	11	96742344	A	18	8	G	0.0005897
rs35547878	11	96742763	T	18	8	A	0.0005897
rs1869267	11	96745534	A	18	8	T	0.0005897
rs1869268	11	96745574	A	18	8	G	0.0005897
rs6589013	11	97618077	T	30	23	C	0.0007082
rs4754310	11	97625249	A	30	23	G	0.0007082
rs34502141	11	97625570	C	29	22	G	0.0007063
rs187247072	11	98475858	T	0	15	C	0.0005628
rs116881838	11	101317751	C	9	1	T	0.0007194
11:108781518	11	108781518	G	10	1	C	0.0002681
rs35232735	11	109983724	CT	26	18	C	0.0007809
rs7482993	11	109996139	A	27	18	G	0.0007941
rs12272465	11	109996164	G	27	18	A	0.0007941

rs10891059	11	109996248	G	26	16	A	0.000295
rs729611	11	109996258	A	26	16	G	0.000295
rs587802	11	109998415	A	26	18	T	0.0007809
rs11213900	11	111310395	A	16	7	C	0.0009539
rs55945818	11	111512871	A	11	43	G	0.0008678
rs67420946	11	111583916	A	7	36	AAGTT	0.0006639
rs10891290	11	111605215	C	7	38	T	0.0001991
rs736650	11	111607051	A	7	35	G	0.0007198
rs72523156	11	111621399	CA	8	38	C	0.0005059
rs10891291	11	111627502	A	7	38	G	0.0001991
rs3829258	11	111642119	A	7	37	G	0.0003628
rs10502148	11	111647998	T	7	38	C	0.0001991
rs1529330	11	111652323	C	7	35	T	0.0007198
rs142679311	11	111659743	T	5	34	TC	0.00009587
rs3867465	11	111662109	T	6	35	C	0.0002764
rs635148	11	111678524	T	7	35	A	0.0007198
rs12284944	11	111787383	G	7	35	A	0.0007198
rs57377042	11	111791075	T	6	34	C	0.0005004
rs7124433	11	111795148	G	7	35	C	0.0007198
rs11384304	11	111796137	TG	7	35	T	0.0007198
rs1320664	11	111800849	G	7	35	A	0.0007198
rs35759638	11	111808876	G	6	36	C	0.0001537
rs10891301	11	111809489	T	6	36	C	0.0001537
rs12225443	11	111814931	C	6	36	A	0.0001537
rs11214049	11	111816143	G	6	36	A	0.0001537
rs7945387	11	111817682	T	6	37	C	0.00008623
rs35440570	11	111817914	C	6	36	T	0.0001537
rs491535	11	111820482	C	6	37	G	0.00008623
rs2105619	11	111825253	T	6	36	G	0.0001537
rs12795123	11	111826511	G	6	36	T	0.0001537
rs510274	11	111833209	A	6	31	G	0.0007822
rs3809033	11	111845933	A	6	36	C	0.0001537
rs1939528	11	111846715	A	6	36	G	0.0001537
rs10891303	11	111848531	T	6	36	C	0.0001537
rs10789854	11	111853865	C	6	36	G	0.0001537
rs479658	11	111856412	C	6	37	A	0.00008623
rs2851187	11	111857217	T	7	36	C	0.0006639
rs4935890	11	111861156	T	6	37	C	0.00008623
rs489417	11	111861190	G	6	37	A	0.00008623
rs10789855	11	111866015	G	6	35	C	0.0002764
rs3839945	11	111866442	G	6	35	GAC	0.0002764

rs10891306	11	111869550	C	6	35	G	0.0002764
rs35823624	11	111873931	A	6	35	G	0.0002764
rs35345409	11	111874414	T	6	35	C	0.0002764
rs10789856	11	111875773	T	6	35	C	0.0002764
rs2081508	11	111876817	T	6	35	C	0.0002764
rs149534010	11	111877844	G	6	36	A	0.0001537
rs201198738	11	111878217	G	6	35	GAC	0.0002764
rs202204694	11	111880807	CCTT	6	37	C	0.00008623
rs650461	11	111889635	G	6	36	C	0.0001537
rs10891310	11	111892059	C	6	36	A	0.0001537
rs9971485	11	111892246	C	6	36	G	0.0001537
rs606721	11	111892515	A	6	36	G	0.0001537
rs1130025	11	111892527	T	6	36	C	0.0001537
rs7940353	11	111893843	C	6	36	G	0.0001537
rs2276391	11	111895253	T	6	35	A	0.0002764
rs2276390	11	111895254	T	6	35	G	0.0002764
rs2303436	11	111896324	T	6	35	C	0.0002764
rs59151988	11	111897658	G	6	35	A	0.0002764
rs4456288	11	111897741	T	5	34	C	0.00009587
rs142747216	11	111897941	T	6	33	C	0.0009098
rs9704549	11	111898203	T	6	32	C	0.00098
rs370830942	11	111898224	T	6	32	C	0.00098
rs373140686	11	111898225	G	6	32	A	0.00098
rs201205994	11	111898617	AGAGAGG	6	34	A	0.0005004
rs11390768	11	111901115	CA	6	37	C	0.00008623
rs10789858	11	111902202	G	6	36	A	0.0001537
rs478941	11	111903995	C	6	37	T	0.00008623
rs694747	11	111904526	C	6	37	T	0.00008623
rs6589259	11	111905468	G	6	34	A	0.0002824
rs71060207	11	111906820	CAT	6	37	C	0.00008623
rs627441	11	111908162	C	6	37	T	0.00008623
rs6589260	11	111909819	A	6	36	C	0.0001537
rs10891313	11	111912083	G	6	36	A	0.0001537
rs7949136	11	111913364	A	6	36	G	0.0001537
rs559170	11	111914566	G	6	37	A	0.00008623
rs646469	11	111916342	G	6	37	A	0.00008623
rs10891314	11	111916647	A	6	36	G	0.0001537
rs676922	11	111920741	G	7	37	A	0.0003628
rs514615	11	111921108	T	7	37	C	0.0003628
rs11214068	11	111926072	C	6	36	G	0.0001537
rs12576402	11	111926528	C	6	36	T	0.0001537

rs34169957	11	111927363	A	6	36	AT	0.0001537
rs3882890	11	111930576	T	6	36	C	0.0001537
rs10891315	11	111933088	G	7	36	A	0.0006639
rs9371	11	111933678	G	7	37	A	0.0003628
rs9822	11	111934507	C	6	36	G	0.0001537
rs11214075	11	111942256	C	6	36	T	0.0001537
rs2303437	11	111944901	T	6	34	C	0.0005004
rs12417851	11	111950136	A	6	34	G	0.0005004
rs3839946	11	111953922	TA	7	35	T	0.0007198
rs10812	11	111955502	T	6	33	C	0.000495
rs35404297	11	111960377	T	7	35	TA	0.0007198
rs10789859	11	111960533	C	7	35	T	0.0007198
rs559147	11	111969827	C	7	35	G	0.0007198
rs498547	11	111972656	T	7	35	C	0.0007198
rs7944155	11	111973603	T	7	35	G	0.0007198
rs12272763	11	111988926	T	7	35	C	0.0007198
rs10431070	11	112779711	T	4	28	C	0.0008078
11:113378629	11	113378629	G	8	40	T	0.00005012
rs73010348	11	117422690	A	9	0	G	0.0001034
rs10892138	11	117437699	G	13	0	T	0.000001239
rs59765231	11	117438189	C	13	0	T	0.000001239
rs648740	11	117447373	A	13	0	G	0.000001239
rs12799825	11	117448013	A	14	0	C	0.0000003945
rs681046	11	117452212	C	13	0	T	0.000001239
rs490914	11	117475052	T	11	1	A	0.00009775
rs680292	11	117475341	T	11	1	G	0.00009775
rs512017	11	117511787	G	28	20	A	0.0005398
rs480801	11	117512831	C	28	19	T	0.0002695
rs537592	11	117517424	C	27	19	T	0.0008555
rs626571	11	117518038	C	29	20	T	0.000273
rs673028	11	117518200	G	29	20	A	0.000273
rs678327	11	117521603	A	32	25	G	0.0004454
rs5795209	11	119882854	G	12	45	GT	0.0009266
rs61897906	11	119887951	A	12	45	G	0.0009266
11:119889757	11	119889757	CCG	12	45	C	0.0009266
rs113601370	11	119897502	G	33	27	GGC	0.0002335
11:119897506	11	119897506	T	34	27	G	0.0001123
rs1784014	11	119897514	T	33	27	G	0.0002335
rs1787023	11	119903704	T	13	51	C	0.0001497
rs1530919	11	119905908	A	11	45	G	0.0004315
rs12420530	11	119907605	T	11	43	G	0.0007455

rs11217652	11	119909306	C	11	45	G	0.0004315
rs5795211	11	119910438	T	11	45	TC	0.0004315
rs2084893	11	119914182	G	11	45	C	0.0004315
rs1597866	11	119914915	G	11	45	A	0.0004315
rs2100357	11	119915395	T	11	45	C	0.0004315
rs2084892	11	119915651	A	11	45	G	0.0004315
rs2887378	11	119916226	A	11	45	G	0.0004315
rs11217656	11	119918703	A	11	45	G	0.0004315
rs11217660	11	119920139	A	11	45	G	0.0004315
rs10892522	11	119921730	G	23	14	A	0.0008216
rs12421541	11	119922558	T	11	45	A	0.0004315
rs666640	11	119923259	T	11	45	A	0.0004315
rs1001973	11	119923643	A	11	45	G	0.0004315
rs664845	11	119923652	A	11	45	G	0.0004315
rs628861	11	119925575	A	11	45	C	0.0004315
rs4938762	11	119927043	T	11	44	C	0.0007768
rs11217663	11	119927491	C	11	44	T	0.0007768
rs1545509	11	119927888	A	11	44	G	0.0007768
rs10444336	11	119928883	A	11	44	C	0.0007768
rs10444337	11	119929276	A	11	44	G	0.0007768
rs10444338	11	119929445	C	11	44	T	0.0007768
rs56937356	11	119932050	T	10	43	C	0.0003854
rs11439053	11	122917024	GA	13	3	G	0.0005464
rs77115993	11	123083510	T	1	19	G	0.0008417
rs76330420	11	123083904	G	1	19	A	0.0008417
rs35040533	11	123083911	G	1	19	A	0.0008417
rs7927152	11	123087671	T	2	23	C	0.0005167
rs113857229	11	123087733	G	2	23	T	0.0005167
rs7927804	11	123088216	T	2	23	C	0.0005167
rs60653352	11	123088698	GCT	2	22	G	0.0009583
rs7931911	11	123089206	C	2	23	G	0.0005167
rs10892982	11	123090555	A	2	23	G	0.0005167
rs10892983	11	123092243	T	2	23	C	0.0005167
rs11600907	11	123092831	A	2	23	G	0.0005167
rs12272976	11	123093198	T	3	27	C	0.0002808
rs10892984	11	123094150	T	3	27	A	0.0002808
rs10790975	11	128756183	C	17	7	A	0.0004382
rs10736569	11	128756564	T	16	7	C	0.0009539
rs1940457	11	128757286	G	16	7	T	0.0009539
rs199564754	11	129907486	GGC	1	19	G	0.0008417
rs2537761	12	10483698	G	34	28	T	0.00053

rs73062703	12	10484833	G	20	11	A	0.0008202
rs145800306	12	10484969	GA	20	11	G	0.0008202
rs12370603	12	10485242	G	36	31	C	0.0003502
rs2110598	12	12900512	G	30	23	A	0.0007082
rs73240463	12	20863306	C	9	1	T	0.0007194
rs1566331	12	24276792	C	20	11	G	0.0008202
rs1039228	12	24285197	C	2	22	G	0.0009583
rs371781671	12	25689751	G	7	0	A	0.0008679
12:26865583	12	26865583	T	20	10	C	0.0005561
rs754296	12	30270589	A	18	9	G	0.0009279
rs1402360	12	43276559	T	3	26	G	0.0005074
12:49151441	12	49151441	A	17	7	AT	0.0005099
rs638515	12	50303927	C	40	38	T	0.0003907
rs61926091	12	50309023	A	40	39	G	0.0006806
rs661776	12	50314771	T	34	29	C	0.0006139
rs836568	12	50315401	G	34	29	A	0.0006139
rs836567	12	50315463	C	34	29	T	0.0006139
rs659674	12	50315797	C	34	29	T	0.0006139
rs584385	12	50316901	T	34	29	C	0.0006139
rs297942	12	50318528	T	34	27	C	0.0002666
rs7311832	12	51618856	G	20	11	A	0.0008202
rs7312011	12	51619155	C	20	11	T	0.0008202
rs58891036	12	51624973	T	20	10	TAAA	0.0005561
rs7397479	12	51630614	T	20	11	C	0.0008202
rs10735833	12	51631016	C	20	11	A	0.0008202
rs1049467	12	51636259	C	20	11	T	0.0008202
rs11331542	12	51641035	CA	20	11	C	0.0008202
rs11613388	12	54342563	T	18	9	C	0.0009279
rs3832829	12	54383924	GCC	6	33	G	0.0009098
rs1109391	12	54387377	T	37	31	G	0.0001688
rs4759316	12	54388473	A	37	31	G	0.0001688
rs71953465	12	54437750	G	20	11	GTT	0.0007056
12:54904482	12	54904482	T	7	0	C	0.0009566
rs144106147	12	57220184	A	7	0	G	0.0008679
rs117634863	12	57234927	G	7	0	C	0.0008679
rs117120119	12	57237463	G	9	0	C	0.0001034
rs35663032	12	57247188	T	9	1	G	0.0007194
rs28678573	12	57262741	A	9	1	G	0.0007194
rs11610564	12	57296274	C	9	1	T	0.0007194
rs7300263	12	60899873	C	2	22	T	0.0009583
rs11173501	12	60903883	C	2	22	T	0.0009583

rs141586045	12	60906183	A	2	22	AAAG	0.0009583
rs7312092	12	60916935	A	2	22	G	0.0009583
rs12307323	12	60917014	A	2	22	G	0.0009583
rs73129004	12	60918843	A	2	22	C	0.0009583
rs35819407	12	60932005	AT	2	22	A	0.0009583
rs11173512	12	60933305	T	2	22	A	0.0009583
rs12313466	12	60937861	G	2	22	A	0.0009583
rs12313099	12	60950166	T	2	22	G	0.0009583
rs9919797	12	60952404	A	2	22	G	0.0009583
rs147477900	12	60952934	GTGATGTGCTGTG	3	25	G	0.0009258
rs11173522	12	60953472	A	3	25	C	0.0009258
rs73130285	12	61759110	G	9	1	A	0.0007194
rs11173917	12	61765951	C	9	1	T	0.0007194
rs11173919	12	61768732	T	9	1	C	0.0007194
rs73130300	12	61770505	C	9	1	T	0.0007194
rs11173953	12	61788334	T	9	1	C	0.0007194
rs73115534	12	61798909	T	9	1	C	0.0007194
rs1516031	12	61802226	G	9	1	A	0.0007194
rs12301969	12	61808150	C	9	1	T	0.0007194
rs139572285	12	61808905	G	9	1	C	0.0007194
rs11173988	12	61809215	C	9	1	T	0.0007194
rs11173990	12	61809325	T	9	1	C	0.0007194
rs148131026	12	61810255	G	9	1	T	0.0007194
rs12227992	12	61817650	G	9	1	C	0.0007194
rs11174010	12	61818194	C	9	1	T	0.0007194
rs8181615	12	61839142	T	9	1	C	0.0007194
rs7294839	12	69510098	G	11	44	C	0.0004199
rs73332112	12	71236244	G	13	4	T	0.000883
rs11358197	12	71250514	A	13	4	AT	0.000883
rs73332150	12	71252402	A	13	4	C	0.000883
rs12302875	12	71252916	T	13	4	C	0.000883
rs73332175	12	71263378	T	13	4	C	0.000883
rs12306153	12	71264782	G	13	4	C	0.000883
rs7975082	12	71265577	G	13	4	A	0.000883
rs7313997	12	71265958	C	13	4	A	0.000883
rs12305560	12	71269754	T	13	4	C	0.000883
rs113621739	12	73788190	G	40	34	A	0.00008167
rs200626615	12	73788192	A	16	56	G	0.0001751
rs11105147	12	77808154	A	27	18	G	0.0004001
rs200391075	12	81755796	G	0	15	C	0.0005628
rs113717657	12	82040845	A	0	14	G	0.0009501

rs7958440	12	85094289	C	11	46	A	0.0002401
rs12815101	12	85098417	A	13	49	T	0.0003246
rs7305535	12	85099563	A	11	46	G	0.0002401
rs66587911	12	85105512	G	11	46	GT	0.0002401
rs4417331	12	85126040	G	11	47	T	0.0001343
rs12812186	12	85130681	T	11	46	G	0.0002401
rs12812197	12	85130694	A	11	46	G	0.0002401
rs71076106	12	85130890	TA	11	46	T	0.0002133
rs7959249	12	85131338	A	11	46	C	0.0002401
rs10862925	12	85132607	G	11	46	T	0.0002401
rs11608665	12	85132866	C	11	46	T	0.0002401
rs11116567	12	85133829	G	11	46	A	0.0002401
rs7975168	12	85135376	T	11	46	C	0.0002401
rs7974535	12	85135521	A	11	46	G	0.0002401
rs12811799	12	85136452	G	11	46	T	0.0002401
rs11116569	12	85139350	G	11	46	A	0.0002401
rs11116570	12	85140141	A	11	46	G	0.0002401
rs11116571	12	85140787	C	11	46	T	0.0002401
rs4424713	12	85142898	A	11	46	G	0.0002401
rs4761090	12	85144089	G	11	46	A	0.0002401
rs10746332	12	85145427	G	11	46	A	0.0002401
rs3051102	12	85146708	C	11	46	CAT	0.0002401
rs7297078	12	85147102	T	11	46	C	0.0002401
rs10779124	12	85147452	T	11	46	C	0.0002401
rs11116584	12	85147715	A	11	46	G	0.0002401
rs10779125	12	85148325	T	11	46	C	0.0002401
rs10779126	12	85148575	C	11	46	T	0.0002401
rs5799843	12	88313577	TA	4	28	T	0.0008078
rs825963	12	90726160	T	40	36	G	0.0001751
rs34969611	12	90726275	G	40	36	GCA	0.0001751
rs1148691	12	90734509	T	5	31	C	0.0006549
rs35236110	12	90736251	A	5	31	AC	0.0006549
rs704094	12	90739448	T	5	31	C	0.0006549
rs704096	12	90740480	C	5	31	T	0.0006549
rs864851	12	90742720	T	5	31	G	0.0006549
rs4842725	12	90743103	G	5	31	A	0.0006549
rs60587792	12	90743126	TATGCAAGTGTCA	3	27	T	0.0002409
rs1499802	12	90743557	A	5	31	G	0.0006549
rs1527024	12	90744231	A	5	31	C	0.0006549
rs704097	12	90745631	G	5	31	T	0.0006549
rs704098	12	90746687	C	5	31	G	0.0006549

rs704099	12	90746821	A	5	31	T	0.0006549
rs704100	12	90747312	C	5	31	T	0.0006549
rs704101	12	90748127	A	5	31	G	0.0006549
rs1653580	12	90749277	G	5	31	A	0.0006549
rs826214	12	90749899	T	5	31	C	0.0006549
rs1148690	12	90751162	C	8	37	G	0.0008686
rs1148689	12	90751181	C	5	31	A	0.0006549
rs17017853	12	90756180	G	5	31	A	0.0006549
rs7135829	12	90757533	G	5	31	A	0.0006549
rs851648	12	90758129	A	8	37	T	0.0008686
rs34737918	12	90759341	A	5	31	AT	0.0006549
rs35014129	12	90760647	A	5	31	AC	0.0006549
rs704103	12	90762487	C	5	31	G	0.0006549
rs67355962	12	90762875	C	5	31	CATGAAT	0.0006549
rs1148685	12	90763408	T	5	31	C	0.0006549
rs1222658	12	90764376	C	5	31	T	0.0006549
rs860459	12	100614498	T	16	6	C	0.0005436
rs150482549	12	100622249	TGGGCA	14	5	T	0.0006534
rs11836972	12	101996918	C	14	4	T	0.0003939
rs11834115	12	101998203	C	14	4	T	0.0003939
rs11110885	12	102003816	T	14	5	C	0.0008508
rs72301569	12	102004290	CT	14	5	C	0.0008508
rs79769390	12	102004539	T	14	5	C	0.0008508
rs7308665	12	102006501	A	23	14	G	0.0007414
rs10507132	12	102006992	T	14	5	C	0.0008508
rs11830848	12	102007433	C	14	4	T	0.0003939
rs7963476	12	102008823	A	14	5	G	0.0008508
rs7963722	12	102009069	C	14	5	G	0.0008508
rs7967311	12	102009916	T	14	5	C	0.0008508
12:103098033	12	103098033	T	6	38	TCCTTCC	0.00008484
rs10860906	12	103098034	T	6	38	C	0.00008484
12:105526569	12	105526569	A	33	24	T	0.000109
rs4529927	12	108749359	G	10	44	T	0.000219
rs10850306	12	114724414	G	33	28	A	0.0009909
rs11067035	12	114742173	G	13	2	A	0.0001085
rs59076525	12	115263658	G	8	0	A	0.0003016
rs780892	12	116224360	C	20	11	G	0.0008202
rs1715583	12	116225839	A	20	11	G	0.0008202
rs1647103	12	116227378	T	20	11	C	0.0008202
rs2681455	12	116261087	A	36	31	G	0.0005991
rs375510578	12	118604654	T	14	4	C	0.0003939

12:122052734	12	122052734	AAAAG	2	22	A	0.0005044
rs12229898	12	130888284	G	0	16	A	0.0005294
rs12309914	12	131442850	T	30	23	C	0.0007875
rs376761445	13	19679763	T	13	4	G	0.000883
rs61946215	13	19691884	G	18	8	A	0.0005739
rs12865323	13	25373515	C	1	20	G	0.0004403
rs146464268	13	25375950	T	1	20	TTTC	0.0004403
rs2186217	13	25376995	A	1	20	G	0.0004403
rs202048843	13	25385229	A	1	20	C	0.0004403
rs373378943	13	25385318	C	1	20	T	0.0004403
rs200257871	13	25385942	A	1	20	G	0.0004403
rs71431704	13	25386966	A	1	20	C	0.0004403
rs35138618	13	25394623	G	1	20	GAGT	0.0004403
rs34876328	13	25395494	C	1	20	CA	0.0004403
rs71431706	13	25395774	G	1	20	A	0.0004403
rs12856222	13	25396027	A	1	20	T	0.0004403
rs17081239	13	25404364	G	1	20	A	0.0004403
rs2121252	13	25410290	C	1	20	G	0.0004403
rs7998291	13	25412619	G	1	20	A	0.0004403
rs147388360	13	25412959	G	1	20	GAGCAGTCAA	0.0004403
rs144125849	13	25412968	A	1	20	G	0.0004403
rs71431711	13	25414647	C	1	20	T	0.0004403
rs12876959	13	25433561	G	1	20	C	0.0004403
rs3839991	13	25451475	TAC	1	20	T	0.0004403
rs71431715	13	25454998	T	1	20	C	0.0004403
rs12864973	13	25459270	T	1	20	C	0.0004403
rs34990870	13	25462716	T	1	20	TA	0.0004403
rs71431716	13	25474085	A	1	19	G	0.0008293
rs12429192	13	25474443	C	1	20	T	0.0004403
rs67483456	13	25481769	A	1	20	AATACTT	0.0004403
rs9553470	13	25500327	T	1	19	C	0.0008417
rs12864497	13	25503448	T	1	19	C	0.0008417
rs12428615	13	25522015	A	1	19	C	0.0008417
rs7333197	13	25526357	T	1	19	C	0.0008417
rs9553479	13	25528542	T	1	19	C	0.0008417
rs143109001	13	25528927	C	3	27	T	0.0002808
rs71431724	13	25528984	A	3	27	G	0.0002808
rs4268601	13	29911974	A	3	25	G	0.000918
rs200445708	13	29946121	C	6	38	CCCCACA	0.00008484
rs200430206	13	29946123	C	15	5	CCA	0.0003243
rs112601805	13	34201773	C	7	0	CA	0.0009566

rs72621283	13	36704967	A	31	24	G	0.0004543
rs9565874	13	36707981	T	31	22	A	0.0001772
rs9565875	13	36708040	A	31	22	G	0.0001772
rs9575380	13	36708286	G	31	22	A	0.0001772
rs1328652	13	36709527	T	31	22	C	0.0001772
rs4480666	13	36713946	G	31	22	A	0.0001772
rs67889814	13	36716095	G	31	22	GA	0.0001772
rs9575407	13	36716901	C	31	22	T	0.0001772
rs55785621	13	36718445	A	31	22	G	0.0001772
rs11839407	13	36721457	A	31	22	G	0.0001772
rs7996350	13	36723145	C	36	27	G	0.00003771
rs2149422	13	36724392	A	31	22	C	0.0001772
rs76291553	13	36725064	A	31	22	AG	0.0001772
rs1928017	13	36726009	A	31	22	C	0.0001772
rs7333872	13	36726387	T	33	23	C	0.00005195
rs7329072	13	36728229	A	33	23	T	0.00005195
rs7322525	13	36728490	A	31	21	G	0.00003533
rs146483983	13	36728706	C	33	23	CAAACA	0.00005195
rs36120646	13	36729284	A	33	23	G	0.00005195
rs34780772	13	36730591	TGA	33	24	T	0.000109
rs17053576	13	36730932	C	33	23	T	0.00005195
rs11841245	13	36731120	A	33	23	G	0.00005195
rs9575434	13	36731197	T	33	23	C	0.00005195
rs9575437	13	36731208	G	33	23	A	0.00005195
rs9575445	13	36731586	A	34	23	G	0.00002346
rs7324116	13	36731747	T	33	23	G	0.00005195
rs7331192	13	36731772	C	33	23	T	0.00005195
rs6563367	13	36732032	G	33	23	A	0.00005195
rs6563368	13	36732113	T	33	23	C	0.00005195
rs6563369	13	36732378	G	33	23	A	0.00005195
rs1328646	13	36732654	A	39	32	G	0.00004458
rs11616924	13	36733944	G	27	19	A	0.000391
rs9575485	13	36735181	A	38	35	G	0.0006427
rs9575486	13	36735182	C	38	35	T	0.0006427
rs7338030	13	37943237	C	13	48	A	0.0005647
rs9576253	13	37943359	C	12	49	T	0.0001424
rs9566214	13	37943776	T	11	49	C	0.00006054
rs9566215	13	37943825	T	11	49	C	0.00006054
rs9576254	13	37944123	C	11	49	G	0.00006054
rs1324023	13	37944928	G	12	49	A	0.0001424
rs4943495	13	37945343	T	33	28	C	0.0009909

rs4943496	13	37945499	C	33	28	T	0.0009909
rs1072736	13	37946430	G	35	29	T	0.0005465
rs57441523	13	37946930	TG	33	28	T	0.0009909
rs7991989	13	37947132	C	33	28	T	0.0009909
rs71198442	13	37947257	T	33	28	TTCTC	0.0009909
rs4420394	13	37948260	C	33	28	T	0.0009909
rs1359209	13	37949872	G	33	28	A	0.0009909
rs970446	13	37951903	C	33	28	A	0.0009909
rs1359211	13	37952685	T	33	28	C	0.0009909
rs9603175	13	37960839	T	13	51	C	0.0001497
rs9566217	13	37963168	G	13	50	A	0.0002818
rs9566218	13	37964230	A	13	50	G	0.0002818
rs2323476	13	37966084	A	13	50	G	0.0002818
rs9576262	13	37967705	T	12	49	G	0.0001424
rs7992271	13	40363878	C	9	1	A	0.0007194
rs17445717	13	40369200	G	9	1	A	0.0007194
rs2985974	13	46086763	T	6	33	C	0.0009098
rs1998789	13	46091363	T	5	31	C	0.0006549
rs9535559	13	51613977	G	40	39	C	0.0006806
rs9568615	13	52080884	A	11	43	C	0.0008678
13:52085316	13	52085316	AGGATTGACTTG	7	36	A	0.0003589
rs148731067	13	52086145	C	12	46	T	0.0005428
rs12861627	13	52090275	A	12	46	G	0.0005428
rs12875314	13	52096145	G	12	46	A	0.0005428
rs9526776	13	52097905	T	12	46	A	0.0005428
rs1913223	13	65370523	A	8	0	T	0.0003016
rs9592365	13	65381616	A	8	0	G	0.0003016
rs9592366	13	65382906	A	8	0	G	0.0003016
rs117707305	13	65383451	T	8	0	C	0.0003016
rs143420078	13	67787159	T	7	0	C	0.0008679
rs45527937	13	67801532	G	7	0	A	0.0008679
rs117179968	13	68160758	A	9	1	G	0.0007194
rs6562505	13	68202087	T	14	5	A	0.0008508
rs1977681	13	68206479	C	14	5	T	0.0008508
rs78853777	13	68213008	G	14	5	GC	0.0008508
rs11839142	13	68216276	G	14	5	A	0.0008508
rs56840064	13	68217594	A	14	5	G	0.0008508
rs11842763	13	68218114	T	14	5	G	0.0008508
rs55773544	13	68219611	T	14	5	C	0.0008508
rs73509529	13	68224831	C	14	5	T	0.0008508
rs56906756	13	68234875	A	14	5	T	0.0008508

rs76113651	13	68236187	C	14	5 T	0.0008508
rs78508177	13	68301306	A	9	1 G	0.0007194
rs79934772	13	70825234	CA	9	1 C	0.0007194
rs181389845	13	70861807	G	7	0 A	0.0008679
rs201127657	13	79012524	ACG	0	20 A	0.0000355
13:79012528	13	79012528	G	0	18 A	0.0001234
rs9530835	13	79497589	A	5	34 G	0.0001138
rs1361540	13	79497895	G	5	32 A	0.0003626
rs4090350	13	79498089	G	5	33 C	0.0002023
rs12560651	13	79499225	A	5	33 C	0.0002023
rs1341133	13	79499934	A	5	34 T	0.0001138
rs1341134	13	79499999	C	6	34 T	0.0005004
rs1341135	13	79500382	T	5	33 C	0.0002023
rs1341136	13	79500420	C	5	33 T	0.0002023
rs8000648	13	79500718	T	6	34 C	0.0005004
rs1934947	13	79501374	C	5	34 T	0.0001138
rs9601138	13	79502310	A	5	33 G	0.0002023
rs142985247	13	79502512	C	5	33 CA	0.0007114
rs9544925	13	79502760	T	6	34 C	0.0005004
rs4511422	13	79504472	C	6	34 T	0.0005004
rs1112425	13	79504570	C	6	34 A	0.0005004
rs1417671	13	79504575	C	5	33 T	0.0002023
rs1417673	13	79504811	T	5	33 C	0.0002023
rs9544926	13	79505489	A	5	32 G	0.0003626
rs9544927	13	79505864	G	5	33 A	0.0002023
rs7325470	13	79508437	C	6	34 A	0.0005004
rs2027425	13	79509217	C	5	32 T	0.0003626
rs7320273	13	79510572	C	5	32 T	0.0003626
rs1539008	13	79512338	T	5	33 C	0.0002023
rs7322004	13	79519992	A	6	32 G	0.00098
rs1304128	13	79520477	A	5	32 G	0.0003626
rs4884117	13	79522117	T	5	33 C	0.0002023
rs7997078	13	82312698	A	13	49 G	0.0003246
rs9561492	13	87689497	A	30	23 T	0.0007082
rs55848046	13	87720203	TATC	30	22 T	0.0001613
rs9590304	13	87842918	G	5	31 A	0.0006549
rs60786638	13	87848266	G	5	31 GT	0.0006549
rs4238212	13	88107344	C	16	54 T	0.0003907
rs9517402	13	88151735	C	13	48 T	0.0005647
rs9517403	13	88151738	C	13	48 T	0.0005647
rs1340830	13	88151753	T	13	48 A	0.0005647

rs1340834	13	88155298	G	13	48	A	0.0005647
rs7993966	13	88161748	G	13	48	T	0.0005647
rs2774188	13	89815375	T	3	27	C	0.0002808
rs59445483	13	89817214	T	4	28	TTA	0.0008078
rs1373729	13	89854996	T	4	28	C	0.0008078
rs397192	13	89894463	T	3	26	A	0.0005074
rs364362	13	89894648	T	3	25	C	0.0009258
rs2781301	13	89895783	C	4	28	G	0.0008078
rs2781302	13	89896514	A	3	26	G	0.0005074
rs143797518	13	89911252	G	3	25	GT	0.0009258
rs191140	13	89915036	T	3	25	C	0.0009258
rs182607	13	89915169	T	3	25	G	0.0009258
rs167546	13	89915230	A	3	25	G	0.0009258
rs221316	13	89915578	G	3	25	A	0.0009258
rs314520	13	89916502	G	3	25	A	0.0009258
rs314522	13	89917083	A	3	25	G	0.0009258
rs78603082	13	89934258	T	3	26	A	0.0005074
rs185327044	13	89935312	T	3	26	C	0.0005074
rs77269664	13	89937844	A	3	26	T	0.0005074
rs78808880	13	89938279	G	3	26	A	0.0005074
rs75321300	13	89938776	T	3	26	C	0.0005074
rs78304741	13	89938954	A	3	26	G	0.0005074
rs74356458	13	89948951	T	3	26	C	0.0005074
rs77576439	13	89951091	C	3	26	G	0.0005074
rs75925780	13	89951263	C	3	26	T	0.0005074
rs75399994	13	89951946	G	3	26	A	0.0005074
rs77540614	13	89952335	C	3	26	A	0.0005074
rs75868821	13	89952354	C	3	26	T	0.0005074
rs79703267	13	89952637	T	2	24	A	0.0002818
rs76579177	13	89954677	C	3	26	T	0.0005074
rs78421246	13	89956345	A	3	26	G	0.0005074
rs74871833	13	89959457	A	3	26	G	0.0005074
rs75263180	13	89963336	T	3	26	C	0.0005074
rs117514867	13	89970443	T	3	26	C	0.0005074
rs7993692	13	90642252	G	8	38	T	0.0005059
13:95469735	13	95469735	A	0	17	ATC	0.0002606
13:95469737	13	95469737	A	0	17	C	0.0002606
rs9557686	13	102184287	G	23	14	A	0.0007414
rs72659103	13	102221421	T	22	12	A	0.000477
rs11450872	13	102229315	AG	19	9	A	0.0009219
rs9518452	13	102229814	T	23	14	C	0.0007414

rs7982227	13	102230866	A	23	14	G	0.0007414
rs139563621	13	102233361	AT	23	14	A	0.0007414
rs9513922	13	102236446	C	23	14	G	0.0007414
rs4771404	13	102236713	A	23	14	G	0.0007414
rs4771405	13	102236957	A	23	14	G	0.0007414
rs4329783	13	102243357	G	23	14	A	0.0007414
rs9557710	13	102246839	C	31	25	T	0.0008555
rs9554809	13	102247971	G	31	25	A	0.0008555
rs1583526	13	102249625	G	31	25	T	0.0008555
rs9585734	13	102252461	A	31	25	C	0.0008555
13:102257790	13	102257790	GCGTATATATACA	23	17	G	0.0008033
rs114819883	13	102257824	G	31	19	C	0.00002421
rs4144376	13	102262631	A	31	24	C	0.0004543
rs7999749	13	102263382	A	27	16	G	0.0001457
rs9557712	13	102264251	G	31	25	A	0.0008555
rs7139608	13	102267424	C	32	25	A	0.0004454
rs4772402	13	102268562	G	24	15	T	0.0005235
rs4772403	13	102268639	T	32	25	C	0.0004454
rs58393947	13	102269161	G	32	25	C	0.0004454
rs7324511	13	102270623	A	32	25	G	0.0004454
rs9557713	13	102270675	A	32	26	G	0.0008799
rs9557714	13	102270794	A	32	25	G	0.0004454
rs9582510	13	102271110	C	32	25	G	0.0004454
rs4238229	13	102271715	T	24	15	C	0.0005235
rs1982142	13	102272356	A	28	16	G	0.00003871
rs7338172	13	102272550	G	24	15	A	0.0005235
rs1125871	13	102272954	A	24	15	C	0.0005235
rs1125872	13	102273822	A	32	25	G	0.0004454
rs1925993	13	102275744	C	24	15	T	0.0005235
rs1836843	13	102276430	G	24	15	T	0.0005235
rs1436260	13	102276717	A	28	16	G	0.00003871
rs4451831	13	102276769	G	32	25	A	0.0004454
rs35177518	13	102277908	A	22	13	AT	0.0006749
rs7982014	13	102282005	T	27	18	A	0.0004001
rs7995030	13	102287484	C	31	25	T	0.0008555
13:102563194	13	102563194	T	19	8	G	0.0003856
rs9300718	13	102866294	G	9	1	T	0.0007194
rs58604229	13	102890429	C	9	1	T	0.0007194
rs57997300	13	102891220	G	9	1	T	0.0007194
rs9518684	13	102895729	C	9	1	T	0.0007194
rs12430941	13	102898489	T	9	1	C	0.0007194

rs9514002	13	102901347	T	9	1	A	0.0007194
rs9518685	13	102905268	C	9	1	T	0.0007194
rs72647461	13	102937184	C	9	1	T	0.0007194
rs72647467	13	102944229	T	9	1	C	0.0007194
rs12430169	13	102948379	C	9	1	G	0.0007194
rs72647476	13	102970018	C	9	1	G	0.0007194
rs72647477	13	102970128	G	9	1	C	0.0007194
13:107735084	13	107735084	GT	36	24	G	0.000005973
13:107735085	13	107735085	T	34	24	A	0.00001993
13:107735086	13	107735086	AGAGTAGAGTAG/	36	24	A	0.000005973
rs77555188	13	110553810	G	11	2	A	0.0004434
rs1889775	13	110595596	G	11	2	T	0.0004434
rs79715145	13	110598569	A	11	2	G	0.0004434
rs7337066	13	110630181	C	11	2	T	0.0004434
rs75270145	13	110630767	T	11	2	C	0.0004434
rs7320126	13	110632935	T	11	2	C	0.0004434
rs75979907	13	110635054	T	11	2	C	0.0004434
rs3742209	13	110638360	A	11	2	G	0.0004434
rs76903621	13	110638738	T	11	2	C	0.0004434
rs74441026	13	110640241	G	11	2	C	0.0004434
rs75343535	13	110642379	T	11	2	G	0.0004434
rs183711260	13	110644456	T	11	2	A	0.0004434
rs76312859	13	110645201	G	11	2	A	0.0004434
rs144036587	13	110645641	C	11	2	A	0.0004434
rs79751079	13	110647902	A	11	2	T	0.0004434
rs76743258	13	110648047	T	11	2	C	0.0004434
rs74995538	13	110648682	T	11	2	C	0.0004434
rs187349184	13	110648837	A	11	2	C	0.0004434
rs150709813	13	110649274	A	11	2	AT	0.0004434
rs77984166	13	110650041	G	11	2	A	0.0004434
rs143872776	13	110651735	CT	11	2	C	0.0004434
rs76847385	13	110652990	G	11	2	A	0.0004434
rs75424296	13	110653121	C	11	2	A	0.0004434
rs73617001	13	110662426	A	10	0	C	0.00003497
rs73618805	13	110664426	G	9	1	C	0.0007194
rs9550152	13	113233836	A	24	15	G	0.0009958
rs12876871	13	114201576	A	5	37	T	0.00002706
rs9549811	13	114205883	T	6	35	A	0.0002764
rs7989136	13	114827017	T	15	51	C	0.0009773
rs9805859	13	114887910	T	16	53	C	0.0006806
rs7982070	13	115064685	G	3	31	A	0.00003786

14:19072247	14	19072247	A	5	33	AT	0.0002023
rs12881322	14	22260754	C	37	34	T	0.0006983
rs11621382	14	22260788	C	37	34	T	0.0006983
rs11624799	14	22260795	A	37	34	C	0.0006983
rs12895584	14	22260810	A	37	34	G	0.0006983
rs11625436	14	22260893	A	37	34	G	0.0006983
rs2733763	14	22267491	T	39	36	C	0.0003692
rs7159790	14	22325185	T	32	26	A	0.000927
rs4982518	14	22333119	A	17	55	C	0.000662
rs909076	14	22337181	T	17	55	C	0.000662
rs12885199	14	22378708	A	37	31	G	0.0001688
rs61980339	14	33448386	C	9	1	A	0.0007194
rs72682330	14	34041274	G	11	2	A	0.0004434
rs28444441	14	34041296	G	11	2	A	0.0004434
rs28407670	14	34041345	G	11	2	A	0.0004434
rs61421949	14	34116500	C	17	6	A	0.0002592
rs797383	14	34538112	T	12	45	C	0.0009266
rs1088254	14	34611351	T	18	9	C	0.0009279
rs140502759	14	34619584	C	18	9	T	0.0009279
rs149154061	14	34620268	C	18	9	T	0.0009279
rs8006604	14	34621153	C	18	9	T	0.0009279
rs8005825	14	34621412	T	18	9	C	0.0009279
rs2915277	14	34621700	C	18	9	T	0.0009279
rs146533521	14	34621909	T	18	9	C	0.0009279
rs9672128	14	34624304	G	18	9	C	0.0009279
rs9671224	14	34624527	T	23	14	G	0.0007414
rs9671225	14	34624830	C	22	13	A	0.0006749
rs11845203	14	35157104	G	27	19	A	0.0008555
rs11627959	14	35160471	A	34	28	C	0.00053
rs8003414	14	35170800	C	34	28	T	0.00053
rs12883300	14	35171218	G	34	28	A	0.00053
rs61981195	14	35172966	G	30	23	A	0.0007082
rs10143298	14	35177986	G	29	22	A	0.0005519
rs712301	14	35180389	T	34	28	A	0.00053
rs61981198	14	35220605	G	7	38	A	0.0001991
rs112182670	14	35223413	A	7	36	G	0.0003589
rs8005659	14	35223956	G	10	43	A	0.0003854
rs8016548	14	35225483	T	10	42	C	0.0006808
rs61981202	14	35228090	A	8	38	G	0.0005059
rs59678582	14	35235160	A	10	43	G	0.0003854
rs10150083	14	35238045	C	10	42	G	0.0006808

rs12435859	14	35242391	T	10	43	C	0.0003854
rs7153289	14	35246534	T	10	43	C	0.0003854
rs35217207	14	35261589	CA	6	37	C	0.0001336
rs112562212	14	35271057	G	8	38	A	0.0008595
rs5807801	14	35343464	A	30	20	AC	0.0005523
rs8012618	14	47542996	A	7	0	G	0.0008679
rs35411710	14	50948012	C	0	15	A	0.0005628
rs4901835	14	58495312	T	19	58	C	0.0006983
rs7148795	14	58495849	C	19	58	T	0.0006983
14:59729372	14	59729372	T	18	8	G	0.0006346
rs200067221	14	63993887	CA	7	0	C	0.0008679
rs11623044	14	66373688	G	7	0	A	0.0008679
rs11621520	14	66382895	G	7	0	A	0.0008679
rs12891482	14	66396885	T	11	2	A	0.0004434
14:70345440	14	70345440	C	4	30	G	0.0002225
rs8008001	14	76583051	C	1	19	T	0.0008417
14:78492143	14	78492143	TGTGTGTGC	5	35	T	0.00009992
rs4903706	14	78556272	G	35	30	A	0.0005835
rs145898678	14	82301150	G	13	4	A	0.0006916
rs7144544	14	84969785	A	11	43	C	0.0008678
rs10143465	14	84971413	T	11	43	C	0.0008678
rs10646499	14	90129322	C	36	32	CTG	0.0006397
rs2632191	14	90132482	C	37	32	T	0.0003248
rs2018763	14	90132951	A	29	22	G	0.0007063
rs12587740	14	90135343	T	36	32	C	0.0006397
rs998564	14	90139311	C	36	31	T	0.0003502
rs2896113	14	90139991	C	36	31	T	0.0003502
rs1967774	14	90142951	A	16	59	G	0.0001347
rs732914	14	90148522	A	36	31	G	0.0003502
rs8006462	14	90878676	T	31	20	C	0.00004024
rs12881551	14	90878880	A	37	32	G	0.0003248
rs4904689	14	90879761	G	37	32	A	0.0003248
rs2896135	14	90880740	G	35	27	A	0.0001338
rs12891145	14	90883069	G	37	33	A	0.0006234
rs759061	14	90883818	C	35	28	A	0.0001616
rs2098254	14	90884018	C	31	21	A	0.00008418
rs2896136	14	90898875	G	30	21	T	0.0001793
rs4904691	14	90899020	G	35	29	A	0.0003069
rs6575136	14	90899380	C	31	22	G	0.0001772
14:91692455	14	91692455	T	7	0	C	0.0008679
rs2069963	14	95052627	T	6	34	C	0.0005004

rs61993541	14	101897039	C	27	17	T	0.000186
rs190885263	14	103632418	T	0	15	C	0.0005628
14:103632429	14	103632429	A	0	15	G	0.0005628
rs199969979	14	106170334	C	11	2	T	0.0004571
15:20086576	15	20086576	T	7	0	TA	0.0008679
rs137957754	15	20291576	C	30	23	T	0.0007875
rs7164260	15	22798709	C	32	21	G	0.00003887
rs4438262	15	27674176	C	24	15	T	0.0005235
rs3922613	15	27682831	C	24	15	T	0.0005235
rs372698865	15	30982225	G	0	17	A	0.0002606
rs28862072	15	31995025	A	5	31	G	0.0006549
rs12595478	15	32001295	A	5	33	G	0.0002023
rs59487628	15	32064340	A	5	30	AG	0.0006526
rs7181402	15	33102157	T	12	45	G	0.0009266
rs1258801	15	33104041	T	13	48	G	0.0005647
rs1258799	15	33106027	A	12	49	C	0.0001424
rs1258795	15	33107305	A	12	49	G	0.0001424
rs34238593	15	34816950	G	10	40	A	0.0006687
15:41438169	15	41438169	G	8	0	A	0.0002766
rs2470149	15	51612240	T	11	2	A	0.0004434
rs2445763	15	51618307	G	11	2	A	0.0004434
rs2470146	15	51618369	G	11	2	A	0.0004434
rs2446404	15	51618943	C	11	2	T	0.0004434
rs2470145	15	51619138	C	11	2	T	0.0004434
rs17602385	15	51624730	C	11	2	T	0.0004434
rs2470169	15	51625116	G	11	2	T	0.0004434
rs2470165	15	51627798	T	11	2	A	0.0004434
rs2446409	15	51628101	A	11	2	G	0.0004434
rs2445764	15	51633517	G	11	2	A	0.0004434
rs2445769	15	51635715	C	11	2	T	0.0004434
rs2445770	15	51637158	A	11	2	G	0.0004434
rs2446425	15	51639727	T	11	2	C	0.0004434
rs2446426	15	51641156	T	11	2	C	0.0004434
rs2470162	15	51643567	G	11	2	C	0.0004434
rs2470161	15	51644669	T	11	2	C	0.0004434
rs145619752	15	51648156	G	9	1	A	0.0007194
rs117429898	15	54562605	A	7	0	G	0.0008679
15:57300052	15	57300052	C	8	41	CT	0.0001347
15:57300054	15	57300054	T	8	39	TC	0.0002434
15:57300056	15	57300056	T	8	40	C	0.0002406
15:57300066	15	57300066	C	8	38	T	0.0007829

rs28367289	15	57868701	A	0	19	G	0.00006755
rs35120591	15	58814992	C	16	5	T	0.0001588
rs1968687	15	58822697	G	15	5	T	0.0003715
rs4775073	15	58826632	T	15	5	C	0.0003715
rs3803448	15	59427200	C	2	22	T	0.0009583
rs11286231	15	59441673	A	8	37	AT	0.0008686
rs7178642	15	65513562	C	35	28	T	0.0001616
rs36013682	15	65515981	G	35	27	A	0.0001338
rs12901822	15	65518320	T	35	28	C	0.0001616
rs7170648	15	65518971	C	35	28	T	0.0001616
rs6494512	15	65519389	G	35	28	T	0.0001616
rs12903067	15	65520087	A	35	28	G	0.0001616
rs4776261	15	65522320	T	35	28	C	0.0001616
rs2083726	15	65525146	A	35	28	C	0.0001616
rs2120014	15	65533541	C	36	32	T	0.0006397
rs12148373	15	65534289	G	36	32	A	0.0006397
rs12593488	15	65535341	T	36	32	C	0.0006397
rs4776676	15	65536316	A	36	32	T	0.0006397
rs1561890	15	65539407	T	36	31	C	0.0003502
rs8029480	15	65540299	T	36	31	C	0.0003502
rs34711258	15	65543186	T	36	31	C	0.0003502
rs11270085	15	65543940	G	36	31	GGATCCCCAGCAAAACTTA	0.0003502
rs4776262	15	65544293	A	36	31	G	0.0003502
rs12906430	15	65548870	T	36	31	C	0.0003502
rs2414879	15	65549457	T	36	31	C	0.0003502
rs1961279	15	65554740	A	36	30	G	0.000303
rs894494	15	65556442	C	36	30	T	0.000303
rs10467935	15	65561077	T	36	29	C	0.0001509
rs10467936	15	65561155	T	35	30	C	0.0005835
rs8023288	15	65561829	A	36	30	C	0.000303
rs7167901	15	65562811	G	36	30	A	0.000303
rs1982257	15	65563649	C	36	30	T	0.000303
rs8032837	15	65567436	A	36	29	C	0.000174
rs10083573	15	65569083	C	36	30	T	0.000303
rs62012559	15	65569302	C	36	28	A	0.0001393
rs62014960	15	65569312	A	35	28	C	0.000282
rs4776679	15	65569933	G	36	30	C	0.000303
rs4776680	15	65571977	G	36	30	A	0.000303
rs2899707	15	65575585	C	36	30	G	0.000303
rs34440719	15	65577548	T	36	30	TCAC	0.000303
rs1057254	15	65579033	A	35	28	G	0.000282

rs7171823	15	65580983	C	36	30	T	0.000303
rs7162046	15	65618532	A	36	32	C	0.0006397
rs2013609	15	67253339	A	38	30	C	0.00003943
rs444141	15	67253847	G	38	30	C	0.00003943
rs871505	15	69429674	G	15	51	A	0.0006974
rs55747964	15	73031102	C	10	1	A	0.0002681
rs35062240	15	80979987	G	8	0	A	0.0003016
rs199976048	15	86780174	G	11	46	GGGGGGGGGC	0.0003773
rs77122263	15	90069444	G	11	2	A	0.0004434
15:91179398	15	91179398	T	13	3	G	0.0002409
rs11629627	15	96608104	T	6	34	C	0.0005004
rs11629694	15	96608137	A	6	37	G	0.00008623
rs12901938	15	96621035	T	23	14	C	0.0007414
rs4998382	15	99331289	C	18	6	G	0.0000668
rs7165207	15	99619610	A	19	9	G	0.0004395
rs8038925	15	99621043	A	20	9	T	0.0002046
rs1509157	15	99623607	T	20	9	C	0.0002046
rs62023819	15	99625603	G	20	10	A	0.0005561
rs10459737	15	99626170	T	20	9	C	0.0002046
rs1551244	15	99629260	A	19	8	G	0.0002919
rs79274204	15	100459748	C	7	0	A	0.0008679
rs112312792	15	102132563	A	7	0	G	0.0009566
rs2294444	16	1724693	G	3	26	T	0.0005074
rs11248888	16	1779399	G	0	16	C	0.0005294
rs34520001	16	1779444	AG	0	17	A	0.0002606
rs12920634	16	1779695	T	0	16	C	0.0005294
rs146273776	16	1788588	C	0	14	T	0.0008416
rs35545084	16	1790297	A	0	14	G	0.0009501
rs2294613	16	1797050	T	0	16	C	0.0005294
rs747254	16	1802518	G	0	16	A	0.0005294
rs4786801	16	6064447	C	15	5	G	0.0003715
rs12924078	16	6281970	T	9	1	C	0.0007194
rs9940011	16	6282896	T	24	15	G	0.0005235
rs9927038	16	6283311	G	25	15	C	0.0002543
rs12930199	16	6283321	G	25	16	A	0.0005585
rs143294556	16	7149149	A	7	0	AAGTT	0.0008679
16:7155779	16	7155779	A	8	0	ATG	0.000247
16:7155788	16	7155788	GAT	8	0	G	0.000247
rs201756757	16	13267014	G	7	0	A	0.0008679
rs57819322	16	16212817	G	7	0	C	0.0008679
16:20886534	16	20886534	G	26	12	A	0.00001225

rs7184277	16	23955242	C	0	16	T	0.0005294
rs142864473	16	24757122	CG	7	0	C	0.0008679
rs4787795	16	26021604	A	37	32	G	0.0003248
rs7189364	16	26021731	T	36	32	C	0.0006397
rs3925102	16	26032650	A	35	29	G	0.0003069
16:32600808	16	32600808	A	2	23	T	0.0004813
rs13332397	16	34175440	C	9	0	G	0.0001034
rs202037976	16	56161190	G	4	48	A	0.000000005601
rs34927755	16	56211091	GT	13	48	G	0.0009655
rs35869729	16	56254662	T	4	31	G	0.0001398
rs36048827	16	56316281	AT	8	37	A	0.0008686
rs899239	16	56321119	A	9	40	G	0.000591
16:62122974	16	62122974	AG	36	28	A	0.00002207
rs4783644	16	66383359	C	12	2	T	0.0001711
rs4788477	16	72754901	C	11	44	T	0.0007768
16:73093273	16	73093273	G	13	2	A	0.00006438
rs200278292	16	76628424	AT	10	0	A	0.00003497
rs118048110	16	76679885	T	9	0	A	0.0001034
rs145368075	16	76814522	G	10	1	A	0.0002681
rs55815014	16	77567903	G	25	16	A	0.0005585
rs881083	16	80652783	C	0	15	A	0.0005628
rs12598873	16	81755511	C	7	36	T	0.0006639
rs4380051	16	81765109	T	15	52	C	0.0006042
rs254334	16	83797708	C	27	19	T	0.0008555
rs462157	16	83821060	C	28	20	T	0.0005398
rs1645850	16	83822305	G	28	20	C	0.0005398
rs556974	16	83874760	C	25	16	T	0.0005585
rs529223	16	83874823	A	23	12	C	0.0002434
rs66658588	16	83875572	T	22	13	TA	0.0004884
rs200190540	16	83875574	G	22	13	A	0.0004884
rs7191122	16	83875688	G	25	16	C	0.000624
rs366495	16	83876171	T	25	16	C	0.000624
rs824544	16	83876991	T	25	16	G	0.0005585
rs365942	16	83880187	A	20	11	C	0.0006096
rs9888805	16	83907379	T	16	53	G	0.0006806
rs247820	16	84445437	C	36	32	T	0.0006397
rs71151211	16	84446090	A	18	8	ATGTG	0.0005897
rs17741422	16	84448418	C	19	8	T	0.0002919
rs12149164	16	84451091	A	22	9	G	0.00004313
rs202114647	16	84481319	T	16	7	TATA	0.0009539
rs8062339	16	84481589	A	16	7	G	0.0009539

rs200078985	16	84766065	T	7	0	C	0.0006731
rs28499270	16	85567332	A	22	11	G	0.0001874
rs8054346	16	86287812	T	14	54	C	0.0001045
rs71388046	16	86487803	GA	9	1	G	0.0007194
rs12922134	16	86489401	A	10	1	G	0.0002681
rs71153865	16	86490325	C	9	1	CTCTT	0.0007194
rs34771315	16	86492690	C	10	1	G	0.0002681
rs4843553	16	87233185	A	32	27	C	0.0004214
rs4024019	17	1216227	C	5	33	A	0.0006717
rs62087949	17	1283446	T	7	0	C	0.0008679
rs7503462	17	3074176	A	8	39	T	0.0004628
rs4564609	17	3078700	A	8	39	C	0.0004628
rs35331788	17	3078740	A	8	39	AAG	0.0004628
rs4542692	17	3078940	A	8	39	C	0.0004628
rs142517458	17	3081461	T	9	40	G	0.000591
rs201492019	17	3082412	C	9	41	A	0.0005847
rs199689703	17	3082992	C	13	47	A	0.0009845
rs12150595	17	3083944	G	9	40	C	0.000591
rs4377191	17	3087015	G	9	41	A	0.0003404
rs4581733	17	3087019	G	9	41	C	0.0003404
rs4294846	17	3087577	A	9	41	C	0.0003404
rs61416144	17	3089784	C	9	41	T	0.0003404
rs60763033	17	3090136	CA	9	41	C	0.0003404
rs4283256	17	3096508	A	9	41	T	0.0003404
rs2241093	17	3100827	C	9	41	T	0.0003404
rs2241091	17	3101578	T	9	41	G	0.0003404
rs12150427	17	3101691	T	9	41	G	0.0003404
rs9652829	17	3106087	C	9	41	T	0.0003404
rs11652109	17	3106962	A	9	41	T	0.0003404
rs11078439	17	3108225	T	9	41	A	0.0003404
rs17174920	17	3119871	C	15	51	T	0.0006974
rs2469803	17	3160405	C	11	43	A	0.0008678
rs2459530	17	3160700	G	11	43	A	0.0008678
rs2469802	17	3161257	G	11	43	C	0.0008678
17:3224740	17	3224740	T	7	0	C	0.0008679
rs142097988	17	3381486	T	8	0	A	0.000247
rs7218276	17	4670631	C	26	17	T	0.0007398
17:6333365	17	6333365	G	24	15	GT	0.0003949
17:6333367	17	6333367	G	24	15	GTGTGT	0.0008364
rs7212120	17	8922842	C	6	35	A	0.0002764
rs57785525	17	9130804	G	0	15	A	0.0005628

rs7211590	17	13139032	G	5	33	A	0.0002023
rs369619837	17	19571912	G	19	9	A	0.0004395
17:19571913	17	19571913	G	19	9	GAAA	0.0004395
17:19571918	17	19571918	G	19	8	GCTTTCCCTAT	0.0002919
rs76119305	17	19605569	C	0	16	T	0.0005294
17:21200214	17	21200214	CTGT	0	15	C	0.0005628
rs116691607	17	28604586	T	7	0	C	0.0008679
rs12944686	17	29930942	G	9	1	A	0.0007194
rs12944364	17	29975200	C	9	1	T	0.0007194
rs7222687	17	31770471	A	0	14	G	0.0009786
rs17784068	17	32497214	C	9	1	T	0.0007194
rs3138032	17	32689042	A	10	1	G	0.0002681
17:39301380	17	39301380	T	20	10	C	0.0005561
17:43122073	17	43122073	C	7	0	A	0.0008679
rs370484228	17	55127061	C	24	15	T	0.0005235
rs4794723	17	55435563	G	21	12	T	0.0009318
rs8068233	17	55450074	A	21	12	G	0.0009318
rs9916654	17	55452531	A	23	13	G	0.0003278
rs11653165	17	55687346	A	1	19	G	0.0008417
rs11079317	17	55708795	A	1	19	G	0.0008417
rs34227181	17	59312249	C	2	27	CTTAT	0.000073
rs12947302	17	59312394	G	1	25	A	0.00002992
rs4793394	17	68509868	A	15	52	G	0.0006042
rs4793395	17	68509948	C	15	52	T	0.0006042
rs9894256	17	68511145	A	15	52	G	0.0006042
rs415298	17	68569973	G	34	29	A	0.0006139
rs72631364	17	71069438	T	14	4	C	0.0003939
rs138367737	17	71498221	C	14	4	CAT	0.0003939
17:73169571	17	73169571	CG	8	0	C	0.000252
rs346802	17	74383601	T	8	0	C	0.0003016
rs346801	17	74383678	A	8	0	G	0.0003016
rs346800	17	74384072	G	8	0	A	0.0003016
rs346799	17	74384123	A	8	0	G	0.0003016
rs76754708	17	74384593	T	8	0	C	0.0003016
rs346816	17	74390938	G	8	0	A	0.0003016
rs346817	17	74396486	A	8	0	G	0.0003016
rs453116	17	74401804	A	8	0	C	0.0003016
rs346810	17	74405114	A	8	0	G	0.0003016
rs346808	17	74405286	A	8	0	G	0.0003016
rs346807	17	74408046	C	8	0	T	0.0003016
rs381774	17	74411788	A	8	0	G	0.0003016

rs377253	17	74413155	C	8	0	G	0.0003016
rs443112	17	74413899	A	8	0	G	0.0003016
rs369654	17	74416147	T	8	0	C	0.0003016
rs560924	17	74416431	A	8	0	G	0.0003016
rs144685854	17	74425173	A	8	0	G	0.0003016
rs146627981	17	74429265	C	8	0	CACA	0.0003016
rs12165049	17	74431465	C	8	0	T	0.0003016
rs495055	17	74440890	G	8	0	A	0.0003016
rs493035	17	74441157	T	8	0	C	0.0003016
rs201664546	17	74442551	C	9	0	A	0.0001034
rs619907	17	74445776	A	8	0	G	0.0003016
rs28709924	17	74448395	G	8	0	C	0.0003016
rs28615986	17	74451270	C	8	0	G	0.0003016
rs73996395	17	74455248	C	8	0	T	0.0003016
rs9912178	17	77445148	C	19	58	T	0.0006983
rs9912221	17	77445186	C	18	57	T	0.0006427
rs8068701	17	81039239	C	15	53	A	0.0003298
rs12957723	18	325884	A	0	16	G	0.0005294
rs8086678	18	905887	T	8	0	C	0.0003016
rs7239044	18	3965251	G	9	1	A	0.0007194
rs7243307	18	3965318	A	9	1	T	0.0007194
rs190474398	18	5499832	G	18	7	A	0.0001719
rs4797378	18	9440382	C	13	47	G	0.0009845
rs12604351	18	13593728	G	7	0	A	0.0008679
rs35363319	18	13607469	GT	7	0	G	0.0008679
rs12605886	18	13611533	T	18	9	G	0.0009279
rs148457493	18	15207993	C	3	27	CCCTTG	0.0002808
rs35585139	18	22958165	A	10	1	G	0.0002681
rs929704	18	24028353	A	14	57	T	0.00001769
rs11433917	18	24029563	G	11	49	GC	0.00006054
rs202138803	18	27865841	AT	11	44	A	0.0007768
rs111414298	18	34880257	G	7	0	A	0.0009566
rs1787086	18	35309697	G	22	9	T	0.00004081
18:35309698	18	35309698	G	17	7	A	0.0004382
18:35309700	18	35309700	CGATCTGTCTAT	23	12	C	0.0002434
rs10853454	18	36005986	G	11	43	A	0.0007141
rs7241992	18	36020096	T	14	52	C	0.0001845
rs472591	18	40483622	A	16	7	G	0.0009539
rs367904418	18	48853566	A	18	7	T	0.0001975
rs5824918	18	49707139	A	20	6	AT	0.00001339
rs17815367	18	49710900	G	20	6	A	0.00001074

rs4940169	18	49713609	T	20	6	C	0.00001074
rs4617958	18	49716105	G	20	6	A	0.00001074
rs117258597	18	49718931	T	14	5	C	0.0008508
rs1822853	18	49722688	G	14	5	C	0.0008508
rs7232159	18	49727080	G	14	5	A	0.0008508
rs56247919	18	49727301	T	14	5	C	0.0008508
rs9967108	18	49731470	A	14	5	G	0.0008508
18:53253409	18	53253409	A	13	47	AG	0.0009845
18:53253411	18	53253411	C	13	47	CAGCAGCAGCAGCAGCAG(	0.0009845
rs10163859	18	56274075	T	14	51	C	0.000328
rs7245005	18	56277218	A	14	51	C	0.000328
rs141675242	18	56915946	AGAAG	38	28	A	0.00006287
18:61815642	18	61815642	C	3	25	T	0.0009258
18:72564123	18	72564123	G	2	26	A	0.0001452
rs60749094	18	73824642	G	37	32	C	0.0003248
rs76470163	18	73824644	G	37	33	C	0.0006234
rs73483896	18	75682645	T	14	4	C	0.0003939
rs113847412	18	75684998	C	14	4	G	0.0003939
rs73485705	18	75688882	T	14	4	C	0.0003939
rs4624302	18	75692920	C	14	4	T	0.0003939
rs113437666	18	75694247	G	14	4	T	0.0003939
rs201116607	19	7312727	T	2	30	C	0.00001022
19:7316684	19	7316684	GTA	2	29	G	0.00001936
rs3093029	19	10399551	G	12	2	C	0.0001711
rs73510898	19	10416444	A	13	4	G	0.000883
rs75231016	19	10420982	C	13	4	G	0.000883
rs12720356	19	10469975	C	14	4	A	0.0003939
rs2304257	19	10491005	G	13	4	C	0.000883
rs34285220	19	11184260	T	7	0	C	0.0008679
rs7249753	19	11192226	C	7	0	T	0.0008679
rs12981050	19	11200412	T	7	0	C	0.0008679
rs2569543	19	11236121	C	9	0	G	0.00008233
rs2569538	19	11238548	A	9	0	G	0.0001034
rs4804567	19	11245472	T	8	0	C	0.0003016
rs7256793	19	11247081	T	8	0	C	0.0003016
rs1054486	19	12774208	C	30	21	G	0.0001793
rs1859007	19	19162186	C	0	16	G	0.0005399
19:36438503	19	36438503	C	10	42	T	0.0003844
19:48492369	19	48492369	T	2	22	TA	0.0009583
rs146010481	19	49222905	GC	0	16	G	0.0005294
rs62132778	19	49234624	C	2	23	G	0.0005167

rs60068692	19	49236465	A	0	15	G	0.0005628
rs35650232	19	49259973	G	6	32	T	0.0008713
rs34860785	19	49554411	G	26	16	GC	0.0002858
rs382838	19	52997170	A	7	0	G	0.0008679
rs36067315	19	54070074	A	30	23	AT	0.0007082
rs10417487	19	54076784	C	28	19	T	0.0002695
rs11879243	19	54093450	A	7	36	G	0.0006279
rs201362768	19	56038849	TCG	3	25	T	0.0009258
rs4384691	19	58567696	G	12	45	A	0.0009266
rs62125248	19	58568181	C	12	45	T	0.0009266
rs974183	19	58569118	C	15	52	G	0.0006042
rs6054736	20	762555	T	27	17	C	0.000186
rs6054737	20	762696	T	27	17	G	0.000186
rs6054739	20	762778	C	27	17	T	0.000186
rs6054740	20	762977	G	27	17	T	0.000186
rs6085872	20	763195	A	27	17	G	0.000186
rs6085874	20	763391	T	21	12	C	0.0009318
rs6085876	20	763541	G	27	17	A	0.000186
rs6085877	20	763651	G	27	17	A	0.000186
rs6085878	20	763831	G	28	17	A	0.0000884
rs6085880	20	763838	A	21	12	G	0.0009318
rs6085881	20	763968	C	27	17	A	0.000186
rs6085882	20	764067	T	27	17	C	0.000186
rs6085884	20	764163	T	27	17	C	0.000186
rs6085887	20	764382	A	27	17	G	0.000186
rs6085889	20	764477	G	27	17	A	0.000186
rs6085890	20	764580	G	27	17	A	0.000186
rs6085892	20	764832	A	27	17	G	0.000186
rs6038717	20	764887	G	27	17	A	0.000186
rs6054751	20	764888	C	27	17	A	0.000186
rs6038718	20	764980	T	27	17	C	0.000186
rs6117641	20	764990	A	27	17	G	0.000186
rs4815973	20	765338	A	27	17	T	0.000186
rs4815974	20	765464	A	27	17	G	0.000186
rs4815975	20	765937	C	27	17	A	0.000186
rs6104589	20	1089123	T	14	51	C	0.000328
rs56113269	20	1655186	T	13	2	C	0.00006438
rs60733085	20	1655481	T	13	2	C	0.00006438
rs4473456	20	1659992	A	13	2	C	0.00006438
rs6043722	20	1661245	A	13	2	G	0.00006438
rs60824122	20	1663080	A	13	2	G	0.00006438

rs12626154	20	1664079	T	13	2	C	0.00006438
rs72484092	20	1665756	G	13	2	T	0.00006438
rs72484093	20	1666842	G	13	2	T	0.00006438
rs2273329	20	1678188	T	13	2	C	0.00006438
rs75624807	20	3427167	A	7	0	C	0.0008679
rs75132954	20	3446195	G	8	0	T	0.0003016
rs16988566	20	3447224	G	8	0	A	0.0003016
rs77240657	20	3447716	G	8	0	A	0.0003016
rs74638763	20	3449559	A	8	0	G	0.0003016
rs6107293	20	3452512	A	8	0	G	0.0003016
rs79115083	20	3455395	G	8	0	T	0.0003016
rs111488652	20	3455575	G	7	0	A	0.0008679
rs73893004	20	3455944	C	8	0	G	0.0003016
rs238724	20	3469332	G	8	0	C	0.0003016
20:3473524	20	3473524	G	8	0	A	0.000247
rs145053037	20	3479380	G	8	0	A	0.0003016
rs116211755	20	3481508	T	7	0	A	0.0008679
rs75203092	20	3485028	C	8	0	A	0.0003016
rs57391116	20	3495212	A	8	0	G	0.0003016
rs112749688	20	3511035	T	7	0	C	0.0008679
rs76354602	20	3512458	G	8	0	A	0.0003016
rs79234455	20	3519375	T	8	0	A	0.0003016
rs11697740	20	9094630	T	18	8	G	0.0005897
rs8122136	20	9096762	A	18	8	G	0.0005897
rs8114968	20	9099219	T	18	8	C	0.0005897
rs9679853	20	9101418	C	18	8	T	0.0005897
rs35494972	20	9102024	T	18	8	C	0.0005897
rs11697355	20	9102114	G	18	8	A	0.0005897
rs7271703	20	9102845	A	18	8	G	0.0005897
rs7266736	20	9103343	G	18	8	A	0.0005897
rs6516437	20	9108165	A	18	8	G	0.0005897
rs12624848	20	9109267	G	18	8	A	0.0005897
rs7264121	20	9114588	T	18	8	A	0.0005897
rs13044386	20	9116155	A	18	8	G	0.0005897
rs986995	20	9120479	G	18	8	C	0.0005897
rs13036264	20	9132796	C	17	7	G	0.0004382
rs13041550	20	9133660	A	16	7	G	0.0009539
rs113907634	20	11224146	AT	28	19	A	0.00047
rs6134480	20	12226630	T	37	34	C	0.0006983
rs6134481	20	12226754	A	37	34	G	0.0006983
rs8117853	20	12226945	C	37	34	T	0.0006983

rs6134807	20	13182235	G	0	15	A	0.0005628
rs6131449	20	13185372	A	0	15	C	0.0005628
rs144086464	20	13186217	C	0	15	CT	0.0005628
rs6137853	20	23147846	C	9	1	T	0.0007194
rs34854625	20	32501744	CT	13	4	C	0.0006614
rs6579129	20	32533524	T	7	35	A	0.0007198
rs73904764	20	32648075	C	2	24	G	0.0002818
rs79078695	20	32654817	G	0	21	T	0.00003544
rs79027916	20	32654818	A	0	21	C	0.00003544
rs77042903	20	32668624	T	2	24	C	0.0002818
20:33909063	20	33909063	G	4	29	GT	0.0004463
rs34874881	20	35284381	G	9	1	A	0.0007194
rs3843762	20	42839990	C	7	0	T	0.0008679
rs16988852	20	42841629	A	7	0	G	0.0008679
rs73106287	20	42842363	A	7	0	T	0.0008679
rs73106290	20	42843271	A	7	0	G	0.0008679
rs73106293	20	42843929	G	7	0	A	0.0008679
rs78619615	20	42844249	C	7	0	CAAT	0.0008679
rs12624924	20	42845269	C	7	0	T	0.0008679
rs75704496	20	42845345	TC	7	0	T	0.0008679
rs45472797	20	42846065	T	7	0	C	0.0008679
rs55691720	20	42847766	G	7	0	A	0.0008679
rs41280254	20	42852509	T	7	0	C	0.0008679
rs523004	20	48205025	T	26	17	C	0.0006727
rs473069	20	48205055	A	27	18	G	0.0004001
rs6063430	20	48404077	C	10	42	T	0.0006808
rs117259369	20	51890546	T	9	1	G	0.0007194
rs204765	20	52250478	T	4	28	C	0.0008078
rs204766	20	52250781	G	4	28	A	0.0008078
rs204767	20	52250876	C	4	28	T	0.0008078
rs204768	20	52251613	G	4	28	C	0.0008078
rs204769	20	52253579	T	4	28	C	0.0008078
rs204771	20	52254420	C	12	49	T	0.0001424
rs71323974	20	52389640	C	13	4	A	0.000981
rs74520236	20	55090657	G	10	2	C	0.0008882
20:55090659	20	55090659	G	9	1	C	0.0005799
rs6025163	20	55364708	T	7	35	C	0.0007198
rs2426686	20	55364766	C	8	41	T	0.0001361
rs167847	20	56521853	A	19	9	T	0.0004395
rs2426761	20	56526972	G	25	14	A	0.0001947
rs370087	20	56527490	T	25	14	C	0.0001947

rs62206222	20	56528595	T	19	9	C	0.0004395
rs7273047	20	57987661	G	17	7	C	0.0004382
rs1041272	20	57987864	A	17	7	G	0.0004382
rs369319469	21	9420676	A	7	0	G	0.0008679
rs375128030	21	9584280	C	8	0	T	0.0003016
21:9994132	21	9994132	G	15	4	T	0.000175
rs11912006	21	16506531	T	7	0	C	0.0008679
rs71330164	21	22886804	C	9	41	A	0.0009153
rs35603376	21	24937476	C	17	57	CT	0.0005003
rs7280791	21	25853593	C	38	35	T	0.0006427
rs9975542	21	29285000	G	17	7	A	0.0004382
rs2091928	21	30617086	G	2	23	A	0.0005167
rs2244978	21	30618226	C	2	23	A	0.0005167
rs1236461	21	30619989	A	2	23	G	0.0005167
rs1236462	21	30622725	A	0	15	G	0.0005628
rs4817280	21	30646046	C	2	23	T	0.0005167
rs78907469	21	30651727	A	0	15	C	0.0005628
rs61547678	21	30658435	T	2	23	C	0.0005167
rs12482988	21	30660917	A	0	15	G	0.0005628
rs4621239	21	30669459	A	2	23	C	0.0005167
rs2300301	21	30676316	G	2	23	A	0.0005167
rs2248043	21	30682347	T	2	22	C	0.0009583
rs2832281	21	30685563	T	2	22	C	0.0009583
rs77638319	21	30702229	TA	2	22	T	0.0009583
rs5843379	21	30717616	A	2	22	AT	0.0009583
rs2832286	21	30720971	T	2	22	C	0.0009583
rs2832287	21	30721932	C	2	22	T	0.0009583
rs2832288	21	30722560	C	2	22	T	0.0009583
rs17744645	21	30726922	A	1	21	G	0.0002339
rs4817283	21	30727894	C	2	22	T	0.0009583
rs11910694	21	30728257	C	2	22	A	0.0009583
rs74313696	21	30731498	G	2	22	A	0.0009583
rs147525188	21	30738295	GT	2	22	G	0.0009583
rs9647054	21	30748728	A	5	31	T	0.0006549
rs9647133	21	30748729	A	5	31	C	0.0006549
rs1977905	21	32668006	T	14	4	C	0.0003939
rs116478170	21	34544260	A	9	1	G	0.0007194
rs73205065	21	34545442	A	9	1	T	0.0007194
rs715116	21	34547667	A	9	1	G	0.0007194
rs73205071	21	34552644	T	9	1	C	0.0007194
rs73205072	21	34552723	C	9	1	T	0.0007194

rs73205073	21	34553102	C	9	1	T	0.0007194
21:38052040	21	38052040	A	0	17	G	0.0002606
rs9975976	21	41422527	G	29	22	A	0.0007063
rs9305690	21	41430388	A	29	22	G	0.0007063
rs13048349	21	43194459	T	13	47	G	0.0009845
rs13051036	21	43194464	G	13	47	A	0.0009845
rs13050469	21	43194647	C	13	48	T	0.0005647
rs13051449	21	43194680	A	13	48	G	0.0005647
rs13048431	21	43194705	A	13	48	C	0.0005647
rs13051575	21	43194795	A	13	48	G	0.0005647
rs13050610	21	43194798	C	13	48	T	0.0005647
rs13047126	21	43194856	G	13	48	A	0.0005647
rs13047384	21	43195062	G	13	48	A	0.0005647
rs13048903	21	43195074	T	13	48	C	0.0005647
rs13047351	21	43195679	C	15	51	T	0.0006974
rs12627623	21	43197859	T	12	46	A	0.0005428
rs60027869	21	43364813	T	18	8	C	0.0005897
rs4920032	21	43368105	A	18	8	C	0.0005897
rs9978540	21	43369599	T	20	9	C	0.0002046
rs183516	21	46278923	T	11	47	C	0.0001343
rs188419227	21	46763272	T	8	0	C	0.0003016
rs147041751	21	46796202	A	8	0	G	0.0003016
rs4818845	21	47966016	G	15	5	C	0.0003715
rs5749041	22	17825248	T	37	33	C	0.0006234
22:21709667	22	21709667	T	6	32	C	0.0008219
rs140433	22	22977269	A	13	3	T	0.0001799
22:23103428	22	23103428	A	20	11	G	0.0008202
rs6003306	22	23136811	G	7	0	A	0.0008679
rs6003308	22	23139043	C	7	0	T	0.0008679
rs12157440	22	23139081	T	7	0	C	0.0008679
rs112199621	22	23140068	T	7	0	TA	0.0008679
rs5996387	22	23143636	G	7	0	A	0.0008679
rs9612496	22	24221405	C	13	47	G	0.0009845
rs9612497	22	24221445	T	13	47	C	0.0009845
rs5763646	22	30359410	C	8	42	T	0.0002398
22:31019704	22	31019704	CACACATATATAT	9	0	C	0.0001173
rs933224	22	36766003	T	30	23	C	0.0007082
rs4821767	22	38614129	C	13	47	A	0.0009845
rs12172253	22	38781997	A	18	9	G	0.0008077
rs139764778	22	41665995	A	7	0	G	0.0008679
rs142865703	22	44594739	TATCC	2	23	T	0.0005167

rs13058073	22	45225247	G	14	5	T	0.0008508
rs4823388	22	45226985	A	19	9	G	0.0004395
rs4823389	22	45226990	G	19	9	A	0.0004395
rs12483795	22	45227123	C	19	9	T	0.0004395
rs4823390	22	45227167	C	19	9	T	0.0004395
rs4823391	22	45227172	T	19	9	C	0.0004395
rs4823392	22	45227273	T	19	9	C	0.0004395
rs5766073	22	45227318	G	19	9	A	0.0004395
rs36042057	22	45227402	AC	19	9	A	0.0004395
rs35282580	22	45227579	CA	14	5	C	0.0008508
rs5766074	22	45227827	G	14	5	C	0.0008508
rs5766075	22	45228629	C	14	5	T	0.0008508
rs150439071	22	45229224	GTGGT	13	2	G	0.00006438
rs5766076	22	45229798	G	14	5	A	0.0008508
rs5766077	22	45230436	T	14	5	C	0.0008508
rs9917547	22	45231099	C	14	5	T	0.0008508
rs5845675	22	45231795	T	14	5	TG	0.0008508
rs5765043	22	45231883	C	14	5	T	0.0008508
rs2269536	22	45232965	G	14	5	A	0.0008508
X:562214	23	562214	A	12	45	G	0.0009266
X:563872	23	563872	A	10	42	C	0.0006808
X:743185	23	743185	C	28	19	T	0.00047
X:944463	23	944463	T	8	37	C	0.0008686
X:951162	23	951162	T	36	33	G	0.0009752
X:952260	23	952260	G	38	34	C	0.000347
X:952262	23	952262	G	38	34	C	0.000347
X:952300	23	952300	C	37	32	G	0.0003684
X:952447	23	952447	G	38	35	GTCTC	0.0006427
X:952501	23	952501	C	38	35	G	0.0006427
rs5939341	23	2734930	G	6	35	C	0.0002764
rs113131499	23	2734948	AC	6	33	A	0.000495
rs62582293	23	2747703	T	1	18	C	0.0008528
rs57068689	23	2748051	T	3	26	C	0.0005131
rs12398173	23	3454292	G	22	11	C	0.0001874
rs5915463	23	3650763	T	20	10	C	0.0005977
rs67132522	23	3665013	C	17	7	T	0.0005099
rs5916697	23	4628916	T	17	55	C	0.000662
rs11798666	23	4905356	C	9	43	T	0.0001644
rs58367116	23	9904364	C	18	9	T	0.0009279
rs5934765	23	10107241	G	7	40	C	0.00008907
rs5934766	23	10108960	A	11	43	G	0.0008678

rs5934767	23	10109014	G	11	43	A	0.0008678
rs5933977	23	12280747	C	30	23	T	0.0007082
rs62578874	23	15495759	C	5	31	T	0.0006549
rs62578875	23	15495810	A	5	31	G	0.0006549
rs6632666	23	15496733	C	5	31	T	0.0006549
rs6629103	23	15499047	C	5	31	A	0.0006549
rs5934245	23	15499746	G	5	31	A	0.0006549
rs5934246	23	15505264	C	5	31	T	0.0006549
rs6632668	23	15506733	G	5	31	A	0.0006549
rs5978772	23	16467111	A	33	25	G	0.0002294
rs4825330	23	17552650	A	3	25	G	0.0009258
rs5904523	23	22285006	T	26	18	A	0.0007809
rs62584154	23	23111867	A	2	23	G	0.0005167
rs73198955	23	23133898	G	9	1	A	0.0007194
rs2695491	23	23134026	T	5	33	A	0.0002023
rs2465828	23	23140838	G	9	42	A	0.0003081
rs5018824	23	23154667	T	12	45	G	0.0009266
rs5925724	23	23154938	C	5	34	G	0.0001138
rs5925725	23	23155133	C	5	32	T	0.0003626
rs79897219	23	23156347	C	7	0	T	0.0008679
rs5926337	23	23465760	C	13	2	T	0.00006438
rs6629690	23	23598811	G	36	29	A	0.0001509
rs4828873	23	23616474	G	33	28	A	0.0009909
rs2885473	23	23616686	C	33	28	T	0.0009909
rs2885474	23	23616752	C	33	28	T	0.0009909
rs5970757	23	23620422	T	32	25	C	0.0004454
rs59623092	23	23622264	A	32	25	AAGAATGCCACTGCCT	0.0004454
rs189169676	23	24248972	T	8	0	G	0.0003016
rs113912339	23	25084085	C	7	0	T	0.0008679
rs112566028	23	25086290	C	7	0	G	0.0008679
rs112466980	23	25088391	C	7	0	G	0.0008679
rs4898305	23	25088920	G	7	0	A	0.0008679
X:25097947	23	25097947	CAT	7	0	C	0.0009566
X:25097957	23	25097957	T	7	0	C	0.0009566
rs113491897	23	25098985	C	7	0	T	0.0008679
rs200103439	23	25104644	TC	7	0	T	0.0008679
rs112483250	23	25112646	C	7	0	T	0.0008679
rs111350748	23	25112690	T	7	0	C	0.0008679
rs113853004	23	25116140	T	7	0	C	0.0008679
rs368084863	23	25120155	T	7	0	C	0.0008679
rs199741820	23	25128561	TAA	7	0	T	0.0008679

rs113637250	23	25132172	G	7	0	A	0.0008679
rs192430333	23	26150490	G	12	2	C	0.0002346
rs143450119	23	26153200	T	12	2	C	0.0001711
rs141122379	23	26222568	A	12	2	G	0.0001711
rs146979379	23	26265498	C	12	2	G	0.0001711
rs145026071	23	26293486	G	12	2	A	0.0001711
rs140699821	23	26341000	T	12	2	G	0.0001711
rs72340275	23	27386423	TTA	31	25	T	0.0008555
rs5926577	23	28323036	T	19	7	A	0.00008788
rs12391023	23	28323501	A	19	7	C	0.00008788
rs12856271	23	28323519	G	19	7	T	0.00008788
rs35197417	23	28326346	T	19	7	C	0.00008788
rs12387440	23	28770293	G	19	9	A	0.0004395
rs10521946	23	28770685	A	19	9	G	0.0004395
rs12846361	23	28776628	G	19	9	A	0.0004395
rs1842879	23	28851539	A	14	5	C	0.0008508
rs5927029	23	31689066	C	11	2	T	0.0004434
rs5927038	23	31768311	A	9	1	G	0.0007194
rs936400	23	31849330	A	6	36	G	0.0001537
rs2405153	23	31850711	C	39	37	G	0.000662
rs1965262	23	31852562	T	6	32	G	0.00098
rs2199049	23	31862612	T	6	33	C	0.0009629
rs4829235	23	31900869	T	3	27	C	0.0002808
X:31915397	23	31915397	C	3	24	G	0.0009363
rs331318	23	32324466	C	40	34	T	0.00008167
rs12010323	23	32327384	A	0	15	T	0.0005628
rs11095228	23	32327486	G	0	15	T	0.0005628
rs28588634	23	32327527	T	0	15	C	0.0005628
rs114124505	23	32329083	T	0	15	C	0.0005628
rs145001082	23	32329186	G	0	15	A	0.0005628
rs41369746	23	32329269	G	0	15	A	0.0005628
rs115862380	23	32330062	A	0	15	G	0.0005628
rs5927113	23	32952019	G	0	18	A	0.0001312
rs139327021	23	33349947	T	13	4	C	0.000883
rs149576274	23	33350633	A	13	4	G	0.000883
rs202231501	23	33351443	A	13	4	AG	0.000883
rs139569192	23	33353400	A	13	4	C	0.000883
rs149704165	23	33353435	A	13	4	G	0.000883
rs73623950	23	33353963	A	13	4	C	0.000883
rs66718538	23	33357839	G	13	4	T	0.000883
rs145704272	23	33361712	T	13	4	C	0.000883

rs138261017	23	33363323	G	13	4	C	0.000883
rs149302050	23	33364294	T	13	4	C	0.000981
rs73623953	23	33365232	C	13	4	G	0.000883
rs139948839	23	33365720	C	13	4	T	0.000883
rs73623955	23	33367217	T	13	4	A	0.000883
rs148926468	23	33369764	T	13	4	C	0.000883
rs143620890	23	33369836	T	13	4	C	0.000883
rs146108307	23	33373323	A	13	4	G	0.000883
rs5928245	23	33374322	T	31	21	C	0.00008418
rs143514056	23	33375050	A	13	4	G	0.000883
rs148524859	23	33378151	G	13	4	A	0.000883
rs142803377	23	33378182	A	13	4	G	0.000883
rs147654804	23	33378479	T	13	4	C	0.000883
rs150341416	23	33379556	A	13	4	C	0.000883
rs7055924	23	33388392	G	13	4	A	0.000883
rs7061656	23	33389014	C	13	4	T	0.000883
rs113761632	23	33392266	A	16	4	G	0.00007821
rs9887051	23	33393169	C	18	4	T	0.00000678
rs150218268	23	33394045	T	18	4	C	0.00000678
rs62594431	23	33394466	A	18	4	T	0.00000678
rs370407198	23	37897073	A	20	10	AG	0.0005561
rs374693453	23	37897075	A	20	10	ATAG	0.0005561
rs4827139	23	39185636	T	7	36	G	0.0006639
rs953524	23	39227207	A	8	37	G	0.0008686
rs12387471	23	39228662	T	8	37	C	0.0008686
rs1490013	23	39231086	T	8	37	G	0.0008686
rs12689301	23	39232009	T	8	37	G	0.0008686
rs67075788	23	39240429	A	8	37	AGAGGTAAAGGATGGCCAT	0.0008686
rs12688992	23	39246849	C	8	37	A	0.0008686
rs729841	23	39251422	G	8	37	A	0.0008686
rs3034995	23	39251561	CGTAGG	8	37	C	0.0008686
rs1472039	23	39252744	C	8	40	T	0.0002504
rs1472040	23	39252819	A	8	40	G	0.0002504
rs5963154	23	39918362	C	38	35	A	0.0006427
rs149891625	23	40121247	T	7	0	C	0.0007305
rs186199538	23	43700851	G	7	0	A	0.0008679
rs5902373	23	46224482	CA	36	32	C	0.0009221
rs5906188	23	46234266	A	36	32	C	0.0006397
rs5905532	23	46235267	T	36	32	C	0.0005072
rs62590007	23	46246592	C	8	43	T	0.00007402
rs73630223	23	46247540	T	37	32	G	0.0003684

rs5952920	23	46539595	A	2	22	C	0.0009379
rs5905555	23	46540220	C	2	22	A	0.0009583
rs5905556	23	46542500	T	2	22	C	0.0009379
rs28445915	23	46544025	G	2	22	A	0.0009583
rs5952923	23	46549671	A	2	22	C	0.0009583
rs66832551	23	46550608	C	2	22	CTG	0.0009583
rs67496984	23	46552310	C	2	22	T	0.0009583
rs5906256	23	46557821	T	2	22	C	0.0009583
rs5906257	23	46558015	T	2	22	G	0.0009583
rs5952926	23	46562584	T	2	22	C	0.0009583
rs375309230	23	46566078	G	2	22	GTGT	0.0009583
rs4607750	23	46577646	T	2	23	C	0.0005167
rs35968392	23	46578551	T	2	23	TA	0.0005167
rs12394488	23	46579194	T	2	23	C	0.0005167
rs5906263	23	46582501	G	2	23	A	0.0005167
rs12397070	23	46586486	G	2	23	C	0.0005167
rs5906264	23	46587103	A	2	23	G	0.0005167
rs4258122	23	46592678	A	2	23	G	0.0005855
rs57634661	23	46593119	T	2	23	TAGATTG	0.0005855
rs5906265	23	46593817	A	2	23	G	0.0005167
rs4824410	23	46598739	T	2	23	G	0.0005167
rs5906266	23	46598854	T	2	23	A	0.0005167
rs4824589	23	46606140	A	2	23	G	0.0005167
rs5906269	23	46607246	A	2	23	C	0.0005167
rs5906272	23	46608657	T	2	23	C	0.0005167
rs5906273	23	46615592	T	2	23	C	0.0005167
rs7061720	23	46616771	A	2	23	G	0.0005167
rs56185835	23	46937076	T	30	22	A	0.0003565
rs2854420	23	47423185	T	9	42	A	0.0003081
rs2854422	23	47425058	A	8	42	G	0.0000745
rs2854426	23	47427594	C	8	42	G	0.0000745
rs2858769	23	47430165	T	8	41	C	0.0001361
rs2854412	23	47430457	T	8	41	A	0.0001361
X:47430614	23	47430614	G	6	36	GT	0.0001301
rs62592099	23	47439278	A	7	36	G	0.0003046
rs111947105	23	47440161	C	7	41	G	0.00002223
rs55696512	23	47443111	A	7	38	G	0.0001991
rs6609534	23	47446653	A	7	38	G	0.0001991
rs5906434	23	47447159	G	7	38	C	0.0001991
rs6520278	23	47447924	T	7	38	C	0.0001991
rs12556415	23	47448275	A	7	38	G	0.0001991

rs5905614	23	47448276 A	7	38 C	0.0001991
rs5906435	23	47448410 T	7	38 C	0.0001991
rs4824621	23	47450276 C	2	32 T	0.00000437
rs4824622	23	47450580 C	2	32 T	0.00000437
rs62592125	23	47450949 A	2	32 G	0.000002242
rs5953061	23	47451476 C	2	32 T	0.00000437
rs5953062	23	47451559 A	2	32 G	0.00000437
rs73206134	23	47453025 C	1	20 T	0.0004403
rs5953063	23	47454598 A	2	32 G	0.00000437
rs5906437	23	47455346 G	2	32 A	0.00000437
rs9887335	23	47459125 G	2	32 A	0.00000437
rs5953066	23	47461549 T	2	32 C	0.00000437
rs4824424	23	47462630 T	2	32 C	0.00000437
rs4824623	23	47462631 G	2	32 T	0.00000437
rs140770289	23	51015458 A	7	0 G	0.0008679
rs143698215	23	51049831 C	7	0 T	0.0008679
X:51056474	23	51056474 A	7	0 C	0.0008679
rs144827840	23	51135633 T	7	0 A	0.0008679
rs141738881	23	51175298 T	7	0 C	0.0008679
X:61803232	23	61803232 A	7	0 G	0.0008679
rs143110708	23	69838040 T	13	4 C	0.000981
rs142361225	23	69935729 G	12	45 A	0.0009266
rs11795627	23	69957441 C	15	52 T	0.0006042
rs5936979	23	69976939 G	8	42 A	0.0000745
rs5936980	23	69982713 A	5	32 G	0.0003626
rs6625672	23	69984497 C	8	42 T	0.0000745
rs4844261	23	69994878 T	5	31 G	0.0006549
rs5936983	23	70001025 T	9	41 C	0.0003404
rs147402025	23	70004851 A	9	41 T	0.0003404
rs6625678	23	70006176 G	9	41 A	0.0003404
rs12164387	23	70006783 G	9	41 A	0.0002923
rs5936984	23	70010619 T	9	41 A	0.0003404
rs5936985	23	70011773 G	9	41 A	0.0009997
rs5936986	23	70013404 G	9	41 A	0.0003404
rs5936987	23	70016629 C	7	41 T	0.00005212
rs140007081	23	70018751 T	7	41 TG	0.00009932
rs6625686	23	70029125 A	7	41 G	0.00005212
rs5936993	23	70034741 T	7	41 A	0.00005212
rs6625691	23	70043958 G	7	41 C	0.00005212
rs5937000	23	70047788 T	9	46 C	0.00004283
rs5937005	23	70077955 G	7	42 A	0.00002668

rs6625698	23	70080295	G	7	42	A	0.00002668
rs5937008	23	70093038	C	7	42	T	0.00002668
rs150018032	23	70094743	A	14	5	G	0.0008508
rs5936597	23	70095359	C	7	42	T	0.00005048
rs200957850	23	70100415	AAAAC	7	42	A	0.00002668
rs201334832	23	70105023	TG	13	4	T	0.000883
rs4617319	23	70108247	C	2	32	A	0.00000437
rs13441058	23	70108862	C	3	32	G	0.00001994
rs13441059	23	70108889	A	3	32	G	0.00001994
rs7888597	23	70110790	G	7	43	A	0.00001382
rs4300150	23	70111711	C	7	43	T	0.00001382
rs5981026	23	70114332	T	8	41	C	0.0001237
rs186347618	23	70117073	T	2	33	A	0.0000008804
rs10672857	23	70117076	TAGA	2	33	T	0.000001897
rs4560953	23	70122195	T	8	43	G	0.0000683
rs62608084	23	70125103	A	2	32	G	0.000004243
rs140342782	23	70127143	C	9	1	T	0.0004616
rs5937022	23	70129087	G	7	44	A	0.000007255
rs143608528	23	70132640	TA	2	31	T	0.00000434
rs72628881	23	70137042	C	7	42	A	0.00001401
rs5937024	23	70141643	G	10	44	T	0.0001738
rs5937025	23	70142030	C	8	42	T	0.00006336
rs4524964	23	70144801	T	2	34	C	0.000001049
rs4360450	23	70146398	A	3	34	G	0.000009143
rs7059898	23	70149078	A	3	32	C	0.00003815
rs11291509	23	70149333	A	5	35	AC	0.0000517
rs5936607	23	70149404	C	7	44	T	0.000005685
rs17216883	23	70151978	G	6	32	A	0.00098
rs5937026	23	70153056	A	8	44	G	0.00003425
rs6625717	23	70153585	C	8	44	T	0.00001695
rs5936610	23	70156788	T	10	44	C	0.000219
rs6625721	23	70157130	C	10	44	G	0.0001848
X:70162038	23	70162038	TA	9	43	T	0.0001644
rs189326428	23	70162078	A	9	43	G	0.0001644
rs144301759	23	70162340	T	9	43	C	0.0001644
X:70162627	23	70162627	G	9	1	T	0.0007194
rs199714075	23	70162636	C	9	42	T	0.0003081
rs4403555	23	70163272	A	9	43	T	0.0001644
rs4402040	23	70163543	G	10	44	A	0.000219
rs4484853	23	70163548	G	9	43	A	0.0001644
rs6624521	23	70163620	G	10	44	A	0.000219

rs4463589	23	70163621	G	9	43	A	0.0001644
rs4370694	23	70163780	A	7	39	G	0.0001964
rs5937029	23	70164217	T	10	44	C	0.000219
rs12556778	23	70164825	G	10	44	A	0.000219
rs4320701	23	70167445	T	8	37	A	0.0007955
rs6625725	23	70168589	T	10	44	C	0.000219
rs5937033	23	70171574	G	11	44	A	0.0007768
rs5981041	23	70173331	C	10	44	A	0.000219
rs5936612	23	70173688	C	10	44	T	0.000219
rs5936613	23	70173743	C	10	44	A	0.000219
rs11094188	23	70173910	G	10	44	A	0.000219
rs11094189	23	70173972	C	10	44	T	0.000219
rs5937035	23	70175558	T	10	44	C	0.000219
rs4273986	23	70178305	A	10	42	G	0.0006601
rs4636364	23	70178323	C	10	42	T	0.0006601
rs6625728	23	70179024	T	10	44	A	0.0001848
rs111415036	23	70179591	G	10	44	A	0.000219
rs5937036	23	70180081	T	11	44	C	0.0007768
rs73214833	23	70181107	A	11	44	G	0.0007768
rs5936615	23	70182429	T	11	44	G	0.0007768
rs5937038	23	70184285	A	10	44	G	0.000219
rs5937039	23	70184830	C	10	44	T	0.000219
rs5981049	23	70185965	G	10	44	A	0.000219
rs11094191	23	70195929	C	11	43	T	0.0008678
rs199639085	23	70196617	ATTTTATTTTATT	11	43	A	0.0008678
rs6625736	23	70197878	C	10	43	T	0.0006662
rs4592186	23	70201767	G	9	41	A	0.0003404
rs201401367	23	70202595	GT	9	41	G	0.0003404
rs6625747	23	70214437	C	9	41	T	0.0003404
rs5936617	23	70215816	T	9	41	G	0.0003404
rs6624525	23	70217433	G	9	41	A	0.0003404
rs5937052	23	70218306	T	9	39	C	0.000596
rs374777112	23	70222085	GAAGA	9	40	G	0.000591
rs5937058	23	70234170	T	10	41	C	0.0006679
rs139693845	23	76898316	C	0	15	T	0.0005628
rs138056129	23	76934081	T	0	15	C	0.0005628
X:77133371	23	77133371	TAC	37	26	T	0.00001019
rs73227653	23	77924400	G	3	27	T	0.0002808
rs2411753	23	78792528	A	33	27	G	0.0005258
rs12559776	23	78794135	A	31	24	C	0.0004543
rs5959402	23	78900345	A	15	51	T	0.0006974

rs5959403	23	78900346	T	15	51	G	0.0006974
rs5959404	23	78901952	G	15	51	A	0.0006974
rs200171362	23	78906807	TGGGAGTGTAAA(	15	51	T	0.0006974
rs145845316	23	78908071	A	15	51	G	0.0006974
rs2411749	23	78910664	A	16	53	G	0.0006806
rs78463595	23	78911465	C	15	51	G	0.0006974
rs73237417	23	78912775	A	15	51	G	0.0006974
rs199862808	23	78913083	T	15	51	TTGATTTTTCTCGATTTTC	0.0006974
rs5958910	23	78914016	C	15	51	G	0.0006974
rs5959408	23	78914271	G	15	51	A	0.0006974
rs147252698	23	78926429	C	15	51	CAGA	0.0006974
rs5958914	23	78928752	G	15	51	C	0.0006974
rs5958915	23	78928840	C	15	51	A	0.0006974
rs5958917	23	78931358	G	15	51	C	0.0006974
rs4826118	23	78932289	A	15	51	G	0.0006974
rs5959421	23	78933591	G	15	51	A	0.0006974
rs34858870	23	78934852	T	14	51	TA	0.000328
rs5959422	23	78935812	T	15	51	G	0.0006974
rs12690356	23	78936316	C	15	51	T	0.0006974
rs12687170	23	78936571	C	15	51	T	0.0006974
rs4826119	23	78939433	A	15	51	G	0.0006974
rs4826121	23	78941684	T	15	51	C	0.0006974
rs4131988	23	80977847	G	16	6	C	0.0005436
rs141979277	23	81185978	T	16	6	C	0.0005436
rs142819227	23	81191091	C	16	6	T	0.0005436
rs188005309	23	81306385	G	16	7	A	0.0007388
rs9698501	23	82020743	A	20	11	G	0.0008202
rs143205082	23	82170027	A	20	11	ATCTCT	0.0008202
rs7061408	23	82187693	A	26	16	G	0.0002858
rs143878757	23	82201623	C	20	11	T	0.0008202
rs139453630	23	82207817	A	20	11	G	0.0008202
rs62617489	23	82236836	G	26	18	A	0.0007809
rs201394251	23	83406022	T	11	2	TAGAG	0.0006828
rs145128705	23	83955996	C	13	48	G	0.0005647
X:86227739	23	86227739	T	2	21	C	0.0007866
rs5969294	23	86983288	C	0	18	G	0.0001312
rs6521963	23	86983765	G	0	18	A	0.0001312
rs5924097	23	86984262	C	0	18	A	0.0001312
rs5924099	23	86985145	C	0	18	T	0.0001312
rs6521964	23	86987258	T	0	18	C	0.0001312
rs5969296	23	86987339	G	0	18	A	0.0001312

rs140914166	23	87630602	A	18	9	G	0.0009279
rs11093096	23	87724429	A	28	19	G	0.0002139
rs188619341	23	88227718	A	0	14	C	0.0009501
rs377141189	23	88462122	G	7	0	A	0.0008679
X:88462136	23	88462136	T	8	0	C	0.0003016
rs138389217	23	91790925	G	5	31	A	0.0006549
rs34341230	23	91792732	TA	5	30	T	0.0006526
rs369536606	23	91798108	G	5	31	A	0.0006549
rs3752336	23	92925839	C	9	1	A	0.0007194
rs115854148	23	92997006	A	7	0	G	0.0008679
rs147086984	23	93033488	A	7	0	G	0.0008679
rs191091193	23	93046551	C	7	0	A	0.0008679
rs143586699	23	94571371	C	11	2	CT	0.0004434
rs6620228	23	96416258	C	33	27	G	0.0009278
rs7051269	23	96424293	G	36	32	A	0.0006397
rs2148100	23	96425266	C	35	29	T	0.0003069
rs6620231	23	96426226	C	35	29	T	0.0003069
rs5967084	23	96426401	G	35	29	T	0.0003069
rs12014439	23	96436052	C	37	32	T	0.0003248
rs5920815	23	96436380	G	37	32	A	0.0003248
rs4007952	23	102446102	G	11	2	GATGTAT	0.0004434
rs5987702	23	102449852	G	11	2	A	0.0004434
rs6621664	23	102678067	C	31	23	T	0.0003729
rs12839772	23	102701896	T	8	0	C	0.0003016
X:102714882	23	102714882	G	13	4	C	0.000883
rs5942626	23	109358807	G	22	12	T	0.000477
rs5942890	23	109362969	G	19	9	A	0.0004395
rs5985446	23	109365260	A	19	9	T	0.0004395
rs4893449	23	109365958	T	19	9	C	0.0004395
rs2185758	23	109366381	A	19	9	T	0.0004395
rs5942893	23	109367316	G	19	9	A	0.0004395
rs5942896	23	109368576	C	19	9	T	0.0004395
rs5942897	23	109368709	G	19	9	A	0.0004395
rs111527219	23	109369426	G	19	9	GT	0.0004395
rs5942628	23	109369578	C	19	9	A	0.0004395
rs4421522	23	109369916	T	19	9	C	0.0004395
rs143509714	23	109372340	C	19	9	CT	0.0004395
rs4893450	23	109374464	G	19	9	C	0.0004395
rs5942629	23	109375110	A	19	9	G	0.0005118
rs141911700	23	109770433	G	9	1	A	0.0007194
X:109846096	23	109846096	T	9	1	C	0.0007194

rs149294490	23	109887848	G	9	1	A	0.0007194
rs5943072	23	110110885	A	3	29	G	0.0001425
rs57105424	23	110116493	GT	3	29	G	0.0001425
rs1556803	23	110186221	T	3	28	C	0.0001569
rs5985312	23	110194225	A	3	28	G	0.0001569
rs141590782	23	110212107	A	7	0	G	0.0008679
rs73215431	23	112845399	G	9	1	T	0.0007194
rs80131172	23	113643765	G	13	2	C	0.00007644
rs148692510	23	113702367	T	13	4	C	0.000883
rs150903498	23	114569770	T	16	6	C	0.0005436
rs7884307	23	114574640	A	16	7	C	0.0009539
rs73636279	23	114576213	C	16	7	A	0.0009539
rs78902560	23	114578966	C	18	7	A	0.0001975
rs73636280	23	114579062	T	18	7	C	0.0001975
rs201116393	23	114579152	C	17	7	CCACACGGTA	0.0004382
rs148225485	23	114579323	A	17	7	C	0.0004382
rs73580006	23	114579496	T	18	7	C	0.0001975
rs7891269	23	114580249	C	18	7	T	0.0001975
rs7886209	23	114580270	A	18	7	G	0.0001975
rs7891410	23	114580274	C	18	7	T	0.0001975
rs1884396	23	114580445	C	17	7	T	0.0004382
rs375467466	23	114582884	T	16	7	G	0.0006018
rs73580011	23	114583333	A	18	7	G	0.0001975
rs73580014	23	114583399	C	17	7	A	0.0004382
rs73580016	23	114583776	G	17	7	A	0.0004382
rs73580018	23	114584158	C	18	7	T	0.0001975
rs147457731	23	114584186	C	18	7	T	0.0001975
rs73580019	23	114584656	C	18	7	T	0.0001975
rs10521696	23	114584805	G	18	7	A	0.0001975
rs73580022	23	114587083	A	18	7	T	0.0001975
rs73580023	23	114587527	T	17	7	C	0.00034
rs5987877	23	114587714	C	17	7	T	0.0004382
rs5987878	23	114587792	C	17	7	T	0.0005099
rs17095493	23	114588167	T	18	7	A	0.0001975
rs112945252	23	114588241	A	17	7	G	0.0004382
rs4911795	23	114589262	G	18	7	T	0.0001975
rs59969921	23	114589858	G	18	7	A	0.0001975
rs4911814	23	114590155	C	17	7	T	0.0004382
rs4911816	23	114590336	A	18	7	G	0.0001975
rs1321125	23	114591500	G	17	7	C	0.0004382
rs2231	23	114591894	C	18	7	T	0.0002294

rs5987726	23	114591955	C	18	7	T	0.0001975
rs73580032	23	114592176	A	17	7	G	0.0004382
rs73580034	23	114592197	A	17	7	G	0.0004382
rs1853000	23	114593037	G	17	7	A	0.0004382
rs1853001	23	114593073	C	18	7	T	0.0001975
rs59121764	23	114594049	C	18	7	T	0.0001975
rs35036780	23	114594708	G	18	7	A	0.0001975
rs113910546	23	114595258	A	18	7	G	0.0001975
rs112652023	23	114596923	A	18	7	G	0.0001975
rs725102	23	114601290	G	18	7	A	0.0001975
rs112071098	23	114604239	A	18	7	C	0.0001975
rs7892307	23	114605041	G	18	7	T	0.0001975
rs1016237	23	114608863	T	18	7	C	0.0001975
rs12014903	23	114611273	A	18	7	C	0.0001975
rs139564840	23	114611882	C	18	7	T	0.0001975
rs73580071	23	114612927	T	18	7	C	0.0001975
rs141250214	23	114616333	C	18	7	G	0.0001975
rs73580080	23	114620385	A	18	9	G	0.0009279
rs73580081	23	114620414	A	18	9	G	0.0009279
rs73580092	23	114625847	T	18	9	C	0.0009279
rs28380553	23	114634849	T	18	9	G	0.0009279
rs111670357	23	114637227	C	18	9	T	0.0009279
rs17260677	23	114637278	A	18	9	G	0.0009279
rs113966797	23	114640655	C	18	9	T	0.0009279
rs4911820	23	114641418	T	19	9	C	0.0004395
rs17326548	23	114644730	C	18	9	G	0.0009279
rs4911821	23	114645027	T	19	9	C	0.0004395
rs59067332	23	114723192	C	23	14	T	0.0007414
rs73577794	23	114724262	T	21	12	G	0.0008432
rs73577800	23	114725164	A	23	14	G	0.0007414
rs2895154	23	114725960	G	22	12	T	0.0005137
rs72605210	23	115120693	T	31	25	C	0.0008555
rs7057172	23	115797756	A	37	32	G	0.0003248
rs1506960	23	115798129	T	37	32	C	0.0003248
rs5956740	23	115799907	C	37	32	G	0.0003248
rs5911731	23	115807323	T	37	32	C	0.0003248
rs34339026	23	115816699	C	17	56	CT	0.0002611
rs150495951	23	116493773	C	7	0	A	0.0008679
rs200125552	23	116596157	C	12	2	A	0.0001711
rs438070	23	118625275	T	0	17	C	0.0002538
rs5909632	23	118664198	A	0	16	G	0.0005294

rs139282281	23	118758234	A	10	0	G	0.00003497
rs147315460	23	118867070	T	0	15	C	0.0005301
rs194291	23	119055950	A	14	50	G	0.0005849
rs194292	23	119055971	C	14	50	A	0.0005849
rs194293	23	119057291	C	14	50	T	0.0005849
rs194296	23	119060622	C	14	50	T	0.0005849
rs7883407	23	119060900	C	12	51	T	0.0001199
rs194297	23	119061588	A	14	50	G	0.0005849
rs194302	23	119065893	G	14	50	A	0.0005849
rs194303	23	119066586	G	14	50	C	0.0005849
rs194307	23	119070411	T	14	50	A	0.0005849
rs194308	23	119070567	A	14	50	T	0.0005849
rs194317	23	119075883	T	14	50	C	0.0005849
rs194318	23	119076675	T	14	50	C	0.0003177
rs194321	23	119080626	T	14	50	C	0.0005849
rs5910713	23	119082081	T	14	50	G	0.0005849
rs72062566	23	119083223	T	14	50	TTAAC	0.0005849
rs5909671	23	119090750	G	14	50	T	0.0005849
rs138142471	23	119100107	G	14	50	A	0.0003177
rs62612907	23	119100673	T	14	50	G	0.0005849
rs2190332	23	119124658	C	16	52	G	0.0006549
rs1468423	23	119128195	A	16	53	G	0.0006806
rs62601344	23	121230701	T	7	0	C	0.0008679
rs62603761	23	121311457	A	9	1	G	0.0007194
rs5909899	23	121806218	A	22	13	G	0.0006749
rs4542067	23	121820256	A	23	12	C	0.0002434
rs35232625	23	121821449	A	23	12	AT	0.0002434
rs5909901	23	121825773	G	22	12	A	0.000477
rs4289947	23	121831400	C	33	28	A	0.0005106
rs5911385	23	121833540	T	23	12	G	0.0002434
rs4436257	23	121841990	G	23	12	A	0.0002434
rs66569928	23	121850214	T	21	12	TA	0.0009012
rs5911397	23	121850402	T	24	14	C	0.0003806
rs6608007	23	121850699	T	24	14	A	0.0003806
rs526716	23	122546969	T	25	14	C	0.0001947
rs35941521	23	123208682	C	14	4	T	0.0003939
rs34543054	23	123602533	C	38	35	T	0.0006427
rs35783045	23	123602802	C	36	31	CCT	0.0003502
rs201775061	23	123647006	AG	4	28	A	0.0008078
rs7051106	23	123809595	T	0	14	A	0.0008416
rs7051135	23	123809651	G	0	14	A	0.0008879

rs5956678	23	123829661	G	0	14	T	0.0009501
rs5932467	23	127763077	C	0	15	G	0.0005628
rs7892367	23	127763657	T	0	15	C	0.0005628
rs5932470	23	127767395	G	0	15	A	0.0005628
rs73223309	23	127770888	T	0	15	C	0.0005628
rs72435158	23	127773725	A	0	15	ATATC	0.0005628
rs5976838	23	127778738	T	0	15	C	0.0005628
rs5976839	23	127780694	C	0	15	T	0.0005628
rs10218225	23	127785423	A	0	15	G	0.0005628
rs2071706	23	128724525	A	6	32	G	0.00098
rs148044293	23	132414633	A	24	15	G	0.0005235
rs2073051	23	132473645	A	6	35	G	0.0002764
rs5933394	23	133383423	G	16	5	T	0.0001054
rs139859148	23	133515780	T	14	5	C	0.0008508
rs518667	23	138077510	C	0	15	T	0.0005628
rs78974273	23	138079591	A	0	15	AC	0.0005628
rs579260	23	138086137	T	0	15	C	0.0005628
rs500096	23	138087155	G	0	15	C	0.0005628
rs565127	23	138088701	C	0	15	T	0.0005628
rs472273	23	138097159	G	0	15	C	0.0005628
rs2179589	23	138097310	G	0	15	A	0.0005628
rs566187	23	138098674	A	0	15	T	0.0005628
rs496732	23	138099648	T	0	15	C	0.0005082
rs490787	23	138100179	G	0	15	A	0.0005628
rs512809	23	138101612	C	0	15	A	0.0005082
rs471600	23	138101814	G	0	15	A	0.0005628
rs482328	23	138102649	C	0	15	T	0.0005628
rs483419	23	138105978	C	0	15	T	0.0005628
rs497635	23	138106122	T	0	15	G	0.0005628
rs578264	23	138107101	C	0	15	T	0.0005628
rs515444	23	138107138	G	0	15	A	0.0005628
rs519841	23	138108803	C	0	15	T	0.0005628
rs149173046	23	138110078	C	0	15	CAAGA	0.0005628
rs693310	23	138110176	C	0	15	T	0.0005628
rs5931560	23	138110307	A	0	15	G	0.0005628
rs526093	23	138111126	G	0	15	A	0.0005628
rs477821	23	138111932	A	0	15	G	0.0005628
rs6635762	23	138113107	A	0	15	G	0.0005628
rs67703366	23	138113374	CA	0	15	C	0.0005628
rs79440397	23	138113382	A	0	15	T	0.0005628
rs17001950	23	138114091	G	0	15	C	0.0005628

rs60643249	23	138114217	A	0	15	G	0.0005628
rs62602197	23	138114331	A	0	15	G	0.0005628
rs61245857	23	138114383	G	0	15	A	0.0005628
rs6634042	23	138114720	C	0	15	A	0.0005628
rs7884030	23	138115512	C	0	15	T	0.0005628
rs7883424	23	138115569	A	0	15	G	0.0005628
rs17001957	23	138116229	C	0	15	A	0.0005628
rs62602201	23	138116536	A	0	15	G	0.0005628
rs62602202	23	138117015	C	0	15	T	0.0005628
rs16993529	23	138117256	T	0	15	C	0.0005628
rs10521792	23	138117409	G	0	15	A	0.0005628
rs17001960	23	138119216	A	0	15	T	0.0005628
rs62602203	23	138119644	C	0	15	A	0.0005628
rs62602204	23	138119702	A	0	15	T	0.0005628
rs112015472	23	138119748	A	0	15	T	0.0005628
rs7889036	23	138119874	T	0	15	A	0.0005628
rs62602205	23	138120458	A	0	15	G	0.0005082
rs12558011	23	138120702	T	0	15	A	0.0005628
rs12556537	23	138120938	C	0	15	T	0.0005628
rs17001963	23	138122394	T	0	15	G	0.0005628
rs62602208	23	138126601	C	0	15	A	0.0005628
rs62602209	23	138127036	G	0	15	A	0.0005628
rs2103610	23	138127481	A	0	15	G	0.0005628
rs112592286	23	138128310	C	0	15	CCTCAGGAAG	0.0005628
rs62602236	23	138128358	A	0	15	G	0.0005628
rs12560172	23	138130418	C	0	15	T	0.0005628
rs62602238	23	138130921	C	0	15	T	0.0005628
rs62602239	23	138133918	G	0	15	T	0.0005628
rs62602240	23	138134536	G	0	15	A	0.0005628
rs62602241	23	138134795	T	0	15	C	0.0005628
rs1080166	23	138137774	C	0	15	T	0.0005628
rs62602242	23	138140150	C	0	15	T	0.0005628
rs7877464	23	138140408	G	0	15	A	0.0005628
rs12556549	23	138140736	T	0	15	G	0.0005628
rs62602243	23	138142110	A	0	15	G	0.0005628
rs374366794	23	138143770	A	0	15	AT	0.0005628
rs113373639	23	138144111	C	0	15	CTTA	0.0005628
rs138951453	23	138152160	C	0	15	T	0.0005628
rs112600886	23	138154239	A	0	15	G	0.0005628
rs5931567	23	138160113	T	33	26	A	0.0002784
rs62603179	23	138166155	G	0	15	A	0.0005628

rs62603180	23	138177022	T	0	15	C	0.0005628
rs62603185	23	138183067	G	0	15	A	0.0005628
rs17331422	23	138187934	G	0	15	A	0.0005628
rs979128	23	138188114	C	0	15	A	0.0005628
rs5907727	23	140002404	T	8	37	C	0.0008686
rs62600825	23	141963178	T	20	11	C	0.0008202
rs2223452	23	141964914	A	23	14	G	0.0007414
rs5904083	23	141964932	C	23	14	CT	0.0008216
rs12839812	23	141965807	A	23	14	G	0.0007414
rs34057690	23	141982184	CTTGA	27	19	C	0.0008555
rs12557040	23	141982219	T	26	18	C	0.0007809
rs2865824	23	142709722	G	6	38	A	0.00007454
rs993478	23	142711939	C	6	38	T	0.00007454
rs35807313	23	142712351	GT	6	38	G	0.00007454
rs5908730	23	142713590	G	6	35	A	0.0002764
rs5907447	23	142719576	A	6	38	T	0.00007454
rs5907448	23	142719596	C	6	38	T	0.00007454
rs5907449	23	142719599	A	6	38	T	0.00007454
rs2038319	23	142727441	C	4	28	T	0.0008078
rs5907452	23	142730813	G	4	30	A	0.0002486
rs2903195	23	142740479	A	4	30	G	0.0002486
rs74769907	23	143200302	C	30	18	T	0.00002692
rs77429583	23	143200330	G	30	18	T	0.00002692
rs147021978	23	143344123	A	8	0	G	0.000247
rs5919645	23	143879419	C	0	20	A	0.0000355
rs1585276	23	144251790	A	24	15	G	0.0005235
rs145921180	23	144431263	T	7	0	A	0.0008679
rs12557016	23	144731102	A	8	0	G	0.0003016
rs17319419	23	144732953	T	8	0	C	0.0003016
rs3852999	23	144735602	C	8	0	T	0.0003016
rs3865789	23	144735612	G	8	0	A	0.0003016
rs6626048	23	144741204	C	8	0	G	0.0003016
rs72608976	23	144746769	A	8	0	G	0.0003016
rs6525626	23	144752754	C	7	0	T	0.0008679
rs17319426	23	144757656	A	8	0	G	0.0003016
rs12847976	23	145613433	T	30	23	G	0.0007082
rs6525699	23	145621372	C	26	18	G	0.0007809
rs5920210	23	145623452	A	26	18	G	0.0007809
rs2207942	23	145630047	C	20	7	T	0.00003885
rs10521858	23	145630262	A	20	7	C	0.00003885
X:145630523	23	145630523	C	19	7	CCCACTT	0.00008788

X:145630534	23	145630534	G	19	7	C	0.00008788
X:145630535	23	145630535	T	19	7	C	0.00008788
X:145630536	23	145630536	G	19	7	C	0.00008788
X:145630539	23	145630539	T	19	7	A	0.00008788
rs202182192	23	145630542	T	19	7	TAG	0.00008788
rs12689781	23	145630720	T	20	7	C	0.00003885
rs2385657	23	145631205	A	20	7	G	0.00003885
rs5920219	23	145631330	G	20	7	C	0.00003885
rs2224194	23	145633069	T	20	7	C	0.00003885
rs5966255	23	145634017	T	20	6	C	0.00001074
rs5920220	23	145637758	T	19	6	G	0.00002708
rs199692010	23	145638047	T	20	8	TTTTA	0.00007555
rs5966261	23	145641460	G	20	7	A	0.00003885
rs5920227	23	145644014	G	20	7	A	0.00003885
rs12688276	23	145645486	T	19	6	G	0.00002708
rs5920228	23	145646314	G	20	6	A	0.00001074
rs6626142	23	145647815	G	19	7	T	0.00008788
rs7061576	23	145649065	T	20	7	A	0.00003885
rs1033646	23	145649246	C	20	7	T	0.00003885
rs1033647	23	145649326	A	20	7	G	0.00003885
rs1033648	23	145649595	G	20	7	T	0.00003885
rs5919714	23	145649944	G	20	7	A	0.00003885
rs1033649	23	145650329	C	20	7	T	0.00003885
rs1033651	23	145650554	C	20	7	A	0.00003885
rs5920231	23	145651231	G	19	6	A	0.00002708
rs5966265	23	145653988	A	17	6	G	0.0002592
rs5966266	23	145658240	T	19	6	A	0.00002708
rs1116419	23	145659829	C	20	6	T	0.00001074
rs1118070	23	145661708	A	20	6	C	0.00001074
rs369026644	23	145661896	G	19	6	GT	0.00002708
rs73240086	23	145666370	A	20	6	T	0.00001074
rs368541187	23	145668384	T	19	6	TTATTGA	0.00002708
rs79144050	23	145668395	G	19	6	A	0.00002708
rs34264217	23	145670465	G	19	6	T	0.00002708
rs12853269	23	145672354	C	23	9	T	0.00002126
rs12855563	23	145672954	G	24	9	T	0.000004665
rs12855872	23	145672989	C	24	9	T	0.000004665
rs61548656	23	145673230	C	28	20	G	0.0005398
rs5920237	23	145674023	A	20	5	G	0.000004766
rs60071470	23	145674985	A	20	6	ATG	0.00001339
rs6626660	23	145680046	A	20	5	G	0.000004766

rs5919717	23	145681309	A	18	5	C	0.000008779
rs6626148	23	145694582	G	18	5	C	0.00002769
rs959605	23	145698003	C	18	5	T	0.00002769
rs4319233	23	145703297	C	18	5	G	0.00003231
rs2891884	23	145703928	G	18	5	A	0.00002769
rs381365	23	145748908	T	18	4	G	0.00000678
rs407585	23	145767522	T	18	4	A	0.00000678
rs445978	23	145788647	T	18	5	A	0.00002392
rs1620816	23	145796549	A	18	5	G	0.00002769
rs5951923	23	146156542	A	39	34	G	0.0001692
rs2442040	23	146351375	G	29	21	T	0.0005775
rs5905048	23	146496477	G	15	56	T	0.0000815
rs2064336	23	149544089	A	7	40	G	0.0001027
rs180495	23	150326325	G	5	33	A	0.0002023
rs180496	23	150330311	A	6	33	G	0.0009098
rs9698833	23	150836609	T	6	34	A	0.0005004
X:151164833	23	151164833	A	1	20	T	0.0004893
rs77731008	23	151203666	G	8	37	A	0.0008686
rs4495590	23	151205238	T	1	21	C	0.0002339
rs4433304	23	151206198	G	1	21	C	0.0002339
rs4828697	23	151666727	C	21	12	T	0.0009318
rs112671205	23	151692719	C	16	7	CTG	0.0007333
rs200866541	23	151717749	A	17	7	C	0.0009525
rs5987101	23	152679963	A	13	47	G	0.0009845
X:153565589	23	153565589	CAA	8	0	C	0.0003016
rs6642243	23	154404057	A	19	8	C	0.0003064
rs6567793	23	154408041	A	32	18	G	0.000005019
rs199802338	23	154412526	T	25	16	C	0.0005585
rs6642392	23	154416077	G	31	17	T	0.000004945
rs3105274	23	154416937	A	31	17	G	0.000004945
rs149642500	23	154418896	T	31	17	A	0.000004945
rs581225	23	154419122	T	37	23	A	0.000001043
rs495251	23	154422423	A	31	17	G	0.000004945
rs6567813	23	154423133	T	26	16	C	0.0002858
rs488312	23	154423899	G	32	18	C	0.000005019
rs150862266	23	154426668	C	31	17	G	0.000004945
rs139630104	23	154428021	A	32	18	T	0.000005019
rs4893075	23	154429214	G	32	18	A	0.000005019
rs149859098	23	154430778	A	26	16	G	0.0002858
rs5983645	23	154431612	C	37	24	T	0.000005162
rs11156599	23	154431803	T	32	18	C	0.000005019

rs12395768	23	154431805 T	26	16 C	0.0002858
rs5983644	23	154434658 T	26	16 C	0.0001243
rs73641118	23	154437045 G	26	16 A	0.0002858
rs73641119	23	154437056 C	26	16 T	0.0002858
rs573189	23	154437617 C	37	24 T	0.000002425
rs573158	23	154437629 G	32	18 A	0.000005019
rs11156600	23	154440161 C	30	16 T	0.00000736
rs499669	23	154442443 C	32	18 G	0.000005019
rs480210	23	154446014 C	33	23 T	0.00005195
rs3105280	23	154451785 C	31	18 T	0.00001169
rs150847435	23	154452126 T	27	18 C	0.0001465
rs35296872	23	154454087 G	25	16 A	0.0005585
rs73569697	23	154458814 A	25	16 G	0.0005585
rs11887	23	154467457 T	31	18 C	0.00001169
rs35943578	23	154468825 T	25	16 C	0.0005585
rs73571105	23	154478027 T	25	16 C	0.000624
rs547447	23	154479421 T	25	16 C	0.0005585
rs475025	23	154484937 G	25	16 A	0.0005585
rs3105275	23	154488432 A	26	16 G	0.0002858
rs543054	23	154493106 G	26	16 A	0.0002858
rs573572	23	154494178 T	26	16 C	0.0002858
rs3105276	23	154495049 A	26	16 G	0.0002858
rs3105277	23	154495135 G	23	13 T	0.0002775
rs200023018	23	154495668 GT	24	16 G	0.0005508
rs3105278	23	154495927 A	26	16 G	0.0002858
rs523614	23	154496166 C	26	16 T	0.0002858
rs485957	23	154497242 G	26	16 T	0.0002858
rs3105279	23	154497666 C	32	18 A	0.000005019
rs578365	23	154498348 C	26	16 T	0.0002858
rs498535	23	154498524 G	26	16 A	0.0002858
rs492622	23	154498668 T	26	16 C	0.0003104
rs503202	23	154499056 T	26	16 C	0.0002858
rs496583	23	154499139 T	26	16 G	0.0002858
rs524668	23	154499876 A	26	16 C	0.0002858
rs527473	23	154500175 T	26	16 C	0.0002858
rs536181	23	154500374 C	26	16 T	0.0002858
rs557984	23	154500421 A	26	16 G	0.0002858
rs5983694	23	154503045 T	26	16 G	0.0002858
rs7891581	23	154503266 T	26	16 A	0.0002858
rs525161	23	154505745 G	26	16 A	0.0002858
rs559165	23	154507173 G	26	16 T	0.0002858

rs515165	23	154508027 G	26	16 C	0.0002858
rs544582	23	154508954 G	26	16 A	0.0002858
rs3120732	23	154509719 C	26	16 T	0.0002858
rs3120731	23	154509738 G	26	16 T	0.0002858
rs812476	23	154512854 C	26	15 T	0.0001216
rs497502	23	154513813 G	26	16 C	0.0002858
rs499428	23	154514047 G	26	16 A	0.0002858
rs504984	23	154514666 C	26	16 T	0.0002858
rs507127	23	154514919 G	26	16 A	0.0002858
rs513441	23	154515619 G	32	18 A	0.000005019
rs535869	23	154515705 T	26	16 C	0.0002858
rs572988	23	154517488 C	26	16 T	0.0002858
rs516396	23	154518759 C	26	16 T	0.0002858
rs519641	23	154519176 A	26	16 C	0.0002858
rs542899	23	154519309 T	26	16 A	0.0002858
rs4893113	23	154520877 T	32	18 C	0.000005019
rs782769	23	154521614 A	26	16 G	0.0002858
rs61166524	23	154521831 G	26	16 A	0.0002858
rs139939230	23	154521832 T	26	16 C	0.0002858
rs55700970	23	154521837 A	26	16 G	0.0002858
rs533001	23	154524691 T	26	16 C	0.0002858
rs201126787	23	154524753 GA	26	16 G	0.0002858
rs3120729	23	154526428 A	26	16 T	0.0002858
rs1032821	23	154527474 G	26	16 C	0.0002858
rs139866562	23	154529407 T	26	16 C	0.0002858
rs201379772	23	154530885 A	26	16 C	0.0001243
rs199886570	23	154531631 A	25	16 G	0.0005585
rs200812781	23	154531843 C	26	16 G	0.0003104
rs201526230	23	154532369 A	24	16 G	0.0005508
rs200983321	23	154533450 G	24	14 T	0.0002009
rs5983838	23	154534628 T	26	16 C	0.0002858
rs1393999	23	154536274 T	26	16 G	0.0002858
rs12013168	23	154536836 T	26	16 C	0.0002858
rs513227	23	154539215 A	24	16 T	0.0005508
rs200876850	23	154539980 AG	32	16 A	0.000001499
rs568396	23	154541603 G	26	16 C	0.0002858
rs506457	23	154542927 A	26	16 T	0.0002858
rs547724	23	154543125 G	32	18 T	0.000005019
rs529965	23	154546292 A	26	16 G	0.0002858
rs534490	23	154546764 A	26	16 G	0.0002858
rs481097	23	154550003 C	26	16 A	0.0002858

rs537992	23	154550004	A	26	16	T	0.0002858
rs4320706	23	154550730	T	26	16	A	0.0002858
rs202190443	23	154553620	A	24	16	AAAAC	0.0005508
rs7052359	23	154553751	C	32	18	T	0.000005019
rs541080	23	154554695	A	26	16	G	0.0002858
rs524609	23	154555595	G	26	16	T	0.0002858
rs495736	23	154557053	A	26	16	T	0.0002858
rs496740	23	154557160	G	32	18	A	0.000005019
rs5983764	23	154558908	C	26	16	T	0.0002858
rs200473316	23	154561955	TTAA	26	16	T	0.0002858
rs520145	23	154564940	C	26	16	G	0.0002858
rs28773194	23	154617179	C	32	18	T	0.000005019
rs28767831	23	154619181	A	26	16	G	0.0003104
rs4131734	23	154622229	C	32	18	T	0.000005019
rs59863310	23	154622257	AT	26	16	A	0.0001017
rs57535347	23	154622912	A	26	16	AAACAAAACTTCAGGTG	0.0003104
rs28868976	23	154623878	A	26	16	G	0.0002858
rs28850951	23	154624809	T	26	16	C	0.0002858
rs369595430	23	154626197	TAAGATCAGGAA[illegible]	26	16	T	0.0002858
rs28867409	23	154628006	A	26	16	G	0.0002858
rs28852197	23	154628007	C	26	16	T	0.0002858
rs28769303	23	154634487	G	26	16	A	0.0002858
rs28676325	23	154634827	C	26	16	T	0.0003104
rs28855519	23	154635311	T	26	16	C	0.0002858
rs28788787	23	154635575	A	26	14	C	0.000109
rs28435674	23	154640027	T	26	16	A	0.0002858
rs28785918	23	154641200	T	26	16	G	0.0002858
rs28782601	23	154642141	C	26	16	T	0.0002858
rs28870179	23	154642144	A	26	16	C	0.0002858
rs28793417	23	154643832	A	26	16	G	0.0002858
rs28576503	23	154644493	T	26	16	C	0.0002858
rs28503175	23	154647114	A	32	18	C	0.000005019
rs28719866	23	154649082	A	26	16	C	0.0002858
rs28476526	23	154650337	A	33	18	G	0.000002153
rs143835791	23	154650909	A	26	16	AC	0.0002858
rs28376822	23	154651459	A	26	16	C	0.0002858
rs28461854	23	154651980	A	26	16	C	0.0002858
rs139968580	23	154652484	G	26	16	A	0.0002858
rs143869954	23	154652773	G	26	16	A	0.0002858
rs146843809	23	154653100	G	26	16	A	0.0002858
rs150609667	23	154653639	T	26	16	A	0.0002858

rs139678244	23	154654004	C	26	16	T	0.0003638
rs146628589	23	154654218	T	26	16	C	0.0003638
rs141107998	23	154654719	T	27	18	G	0.0004001
rs77705284	23	154655375	C	26	17	A	0.0003852
rs74858147	23	154655663	G	26	16	A	0.0002858
rs145051005	23	154657022	A	26	16	C	0.0002858
rs113233836	23	154657311	C	26	16	A	0.0002858
rs61036919	23	154657762	T	26	16	TATA	0.0003104
rs28444648	23	154657902	A	26	16	C	0.0002858
rs28599172	23	154658213	T	26	16	C	0.0002858
rs28448813	23	154658332	C	26	16	G	0.0002858
rs28655495	23	154658415	A	26	16	G	0.0002858
rs28656912	23	154659587	C	26	17	T	0.0003852
rs28860618	23	154660311	G	26	16	A	0.0002858
rs146704646	23	154660744	G	26	16	A	0.0002858
rs141788318	23	154661259	C	26	16	T	0.0002858
rs150613854	23	154661565	G	26	16	A	0.0002858
rs140276121	23	154661793	A	26	16	C	0.0002858
rs79923209	23	154662136	C	26	16	T	0.0002858
rs28864804	23	154665095	G	32	18	A	0.000005019
rs28848797	23	154665337	C	32	18	T	0.000005019
rs28791527	23	154665890	G	26	16	A	0.0002858
rs28842206	23	154665968	G	32	18	A	0.000005019
rs28857192	23	154666533	A	26	16	T	0.0002858
rs28862095	23	154667475	C	26	16	T	0.0002858
rs28480480	23	154668066	T	32	18	A	0.000005019
rs28810503	23	154670873	A	32	18	C	0.000005019
rs111951856	23	154671407	T	26	16	TACTC	0.0002858
rs28790957	23	154671432	T	26	16	A	0.0002858
rs28526133	23	154674697	G	26	16	A	0.0002858
rs111291840	23	154674731	G	26	16	GA	0.0002858
rs28880039	23	154678628	G	32	19	C	0.00001307
rs28873383	23	154678938	C	26	16	T	0.0002858
rs28811917	23	154679547	A	26	17	T	0.0003852
rs28805403	23	154680583	G	26	16	A	0.0002858
rs28769041	23	154680828	C	26	16	T	0.0002858
rs5940390	23	154681629	T	26	16	A	0.0003104
rs5940511	23	154682147	C	26	16	A	0.0002858
rs5940510	23	154683663	C	28	19	T	0.0002695
rs72616497	23	154696888	C	26	16	T	0.0002858
rs72616498	23	154696905	A	26	16	G	0.0002858

rs12557310	23	154735698	T	32	18	C	0.000005019
rs5940458	23	154736968	T	26	16	G	0.0002858
rs5940372	23	154737376	G	26	16	A	0.0002858
rs989711	23	154739500	A	26	16	T	0.0002858
rs79920288	23	154740179	T	26	16	C	0.0002858
rs76536573	23	154740180	G	33	18	A	0.000002153
rs5940460	23	154740429	A	26	18	G	0.0007809
rs6642272	23	154741935	A	26	16	C	0.0002858
rs5940463	23	154745067	T	26	16	A	0.0002858
rs5940464	23	154745451	G	26	16	T	0.0002858
rs1598123	23	154753286	C	26	16	G	0.0002858
rs5940465	23	154753903	A	32	18	G	0.000005019
rs56387516	23	154754455	T	32	18	C	0.000005019
rs2123894	23	154755253	T	26	16	A	0.0002858
rs57421488	23	154757994	ATTTTT	26	16	A	0.0002858
rs1840291	23	154758172	G	26	16	A	0.0002858
rs1454268	23	154758477	T	26	16	C	0.0002858
rs11270561	23	154760090	A	26	16	AGGGAGGAAATGGGAGAT	0.0002858
rs5940466	23	154763036	A	26	16	G	0.0002858
rs60233546	23	154763040	G	26	15	GCA	0.0001216
rs5940467	23	154763368	G	26	16	A	0.0002858
rs5940468	23	154766596	A	26	16	C	0.0002858
rs5940469	23	154766772	C	26	16	G	0.0002858
rs1598126	23	154767486	A	26	16	C	0.0002858
rs1563178	23	154770290	A	26	16	T	0.0002858
rs4514170	23	154771641	C	26	16	T	0.0003104
rs2305517	23	154774663	G	32	18	A	0.000005019
rs2305518	23	154774707	C	26	16	T	0.0002858
rs5940478	23	154776197	G	26	16	A	0.0002858
rs5940376	23	154776510	G	26	16	A	0.0002858
rs5940479	23	154776644	T	26	16	C	0.0002858
rs5940480	23	154776650	A	32	18	G	0.000005019
rs5940484	23	154777564	C	26	16	A	0.0002858
rs5940485	23	154777919	A	26	16	G	0.0002858
rs5940486	23	154778123	A	26	16	G	0.0002858
rs5940487	23	154778278	C	24	16	T	0.0009443
rs5940381	23	154778352	T	31	18	C	0.0000103
rs6642286	23	154779570	C	26	16	G	0.0002858
rs12688786	23	154779966	T	32	17	A	0.000001976
rs6642287	23	154780283	A	32	17	C	0.000001976
rs5940488	23	154780734	A	26	16	T	0.0002858

rs5940489	23	154780920	A	26	16	G	0.0002858
rs6567762	23	154781700	G	26	16	C	0.0002858
rs5940490	23	154782661	C	26	16	T	0.0002858
rs5940491	23	154782766	C	26	16	T	0.0002858
rs2085080	23	154784641	T	26	16	C	0.0003104
rs2379272	23	154784846	C	26	16	T	0.0002858
rs72278264	23	154784848	TC	26	16	T	0.0002858
rs5940392	23	154784873	A	26	16	C	0.0002858
rs2085081	23	154784876	G	26	16	T	0.0002858
rs2085082	23	154784971	C	26	16	G	0.0003104
rs6567763	23	154785091	G	26	16	C	0.0003104
rs6567764	23	154785167	T	26	16	C	0.0003104
rs5940519	23	154785672	A	27	18	G	0.0004513
rs5940521	23	154786106	G	32	18	A	0.000005019
rs6642226	23	154786213	C	26	16	G	0.0002858
rs6567765	23	154786302	G	26	16	C	0.0002858
rs6642292	23	154786702	C	32	18	T	0.000005019
rs6642293	23	154787056	C	26	16	T	0.0002858
rs5940512	23	154789183	G	32	18	A	0.000005019
rs5940513	23	154789496	A	26	16	G	0.0002858
rs5940526	23	154803793	T	32	18	A	0.000005019
rs5940397	23	154804301	G	26	16	A	0.0002858
rs12834147	23	154805102	G	26	16	C	0.0002858
rs2890132	23	154805911	G	26	16	A	0.0002858
rs371249305	23	154808856	TA	26	16	T	0.0002858
rs1598125	23	154809205	T	26	16	C	0.0002858
rs139295015	23	154810792	T	32	18	C	0.000005019
rs140600574	23	154811177	C	26	16	T	0.0002858
rs142781423	23	154811232	T	26	16	C	0.0003104
rs1084453	23	154812237	C	26	16	T	0.0002858
rs1084452	23	154814147	C	26	14	T	0.00009952
rs1084451	23	154816439	A	26	16	C	0.0002858
rs1091848	23	154818314	C	26	16	T	0.0003104
rs782311	23	154819003	C	26	16	T	0.0002858
rs782310	23	154819368	T	26	16	C	0.0002858
rs1084450	23	154822874	A	26	16	T	0.0002858
rs782308	23	154825665	T	26	16	A	0.0002858
rs782307	23	154826161	G	32	18	C	0.000005019
rs782306	23	154826774	G	26	16	A	0.0001017
rs809138	23	154827160	T	26	16	C	0.0002858
rs5983745	23	154827280	T	26	16	C	0.0002858

rs5983658	23	154830377	C	26	16	T	0.0002858
rs5940539	23	154830650	A	32	18	C	0.000005019
rs10782079	23	154830744	A	26	16	G	0.0002858
rs522805	23	154839087	T	26	16	C	0.0002858
rs89278	23	154842666	C	26	16	T	0.0002858
rs479901	23	154844287	A	26	16	T	0.0002858
rs476928	23	154844660	C	26	16	G	0.0002858
rs561928	23	154845104	G	26	16	T	0.0002858
rs498000	23	154846104	G	26	16	T	0.0002858
rs580468	23	154846835	A	32	18	G	0.000005019
rs574386	23	154847489	C	26	16	T	0.0002858
rs146884178	23	154848759	T	26	16	TCTTAGAGCC	0.0002858
rs1834433	23	154849030	G	26	16	T	0.00005482
rs672434	23	154850125	T	26	16	A	0.0002858
rs569049	23	154850801	T	26	16	C	0.0002858
rs561841	23	154851523	G	26	16	A	0.0002858
rs75097820	23	154855606	T	26	16	TTA	0.0002858
rs654274	23	154856281	A	26	16	G	0.0002858
rs666267	23	154856709	C	26	16	G	0.0002858
rs666344	23	154856764	A	26	16	C	0.0002858
rs573685	23	154856774	T	26	16	A	0.0002858
rs514136	23	154858649	C	24	16	T	0.0005508
rs513234	23	154858725	T	26	16	C	0.0002858
rs622211	23	154860283	A	26	16	G	0.0002858
rs487307	23	154865855	A	26	16	G	0.0002858
rs601290	23	154865915	G	26	16	A	0.0002858
rs473772	23	154870197	T	26	16	C	0.0002858
rs528286	23	154871743	C	27	16	T	0.0001457
rs612412	23	154872707	G	26	16	A	0.0002858
rs613783	23	154873044	C	32	18	A	0.000005019
rs642362	23	154874837	A	26	16	G	0.0002858
rs372034665	23	154875192	GTACTC	26	16	G	0.0002858
rs150712976	23	154879115	A	26	16	G	0.0002858
rs491661	23	154880669	T	24	16	A	0.0005508
rs494282	23	154880917	C	26	16	T	0.0002858
rs520792	23	154881521	G	32	18	A	0.000005019
rs563690	23	154885405	G	26	16	A	0.0002858
rs608695	23	154885878	A	26	16	G	0.0002858
rs610828	23	154886333	A	26	16	G	0.0002858
rs512918	23	154887308	G	32	18	A	0.000005019
rs636742	23	154887583	C	26	16	T	0.0002858

rs638156	23	154887924	T	26	16	A	0.0002858
rs547437	23	154888722	G	26	16	A	0.0002858
rs671588	23	154889423	C	26	16	T	0.0002858
rs526172	23	154891554	G	26	16	T	0.0002858
rs553678	23	154892230	A	26	16	G	0.0003104
rs513900	23	154893980	C	26	16	T	0.0002858
rs637652	23	154894353	A	26	16	C	0.0002858
rs34440537	23	154897693	TA	26	16	T	0.0002858
rs586843	23	154897868	G	26	16	A	0.0002858
rs549327	23	154898403	C	32	18	A	0.000005019
rs473491	23	154899846	A	32	18	G	0.000005019
rs628761	23	154900717	C	26	16	A	0.0002858
rs627797	23	154900976	C	32	18	G	0.000005019
rs697727	23	154901936	A	26	16	G	0.0002858
rs696316	23	154902105	G	26	16	T	0.0002858
rs697725	23	154902889	A	26	16	T	0.0003104
rs479770	23	154903118	G	26	16	A	0.0002858
rs480725	23	154903224	A	26	16	T	0.0002858
rs674707	23	154903937	G	26	16	A	0.0002858
rs488777	23	154906905	T	26	16	C	0.0002858
rs601837	23	154908749	C	26	16	G	0.0002858
rs473529	23	154909055	G	32	21	C	0.00003887
rs586454	23	154909877	T	25	13	G	0.0000842
rs622581	23	154913799	C	26	16	T	0.0003104
rs35543589	23	154915433	C	26	14	CT	0.00009952
rs571421	23	154915619	A	24	16	G	0.0005508
rs672932	23	154915975	A	26	16	G	0.0002858
rs669237	23	154916845	T	26	16	G	0.0002858
rs642043	23	154918266	T	32	21	C	0.00003887
rs509981	23	154925045	T	26	16	C	0.0002858
rs538470	23	154925895	T	26	16	C	0.0002858
rs645904	23	154927199	T	26	16	C	0.0002858
rs644138	23	154927581	A	32	21	G	0.00003887
rs557132	23	154929412	T	26	16	C	0.0002858
rs4012982	23	154929952	C	25	16	CAA	0.0005585
rs781880	23	154930230	G	22	12	A	0.0002081
X:154981605	23	154981605	A	15	4	T	0.0001557
X:155255914	23	155255914	C	9	1	G	0.0007194

Supplementary Table 3. All nominally significant (p≤0.001) genetic variants (SNVs/INDELs) for thrombocytopenia as determined by Fisher's exact test.

rsID	Chromosome	Position	Minor allele	Minor allele frequency among cases	Minor allele frequency among controls	Major allele	p-value
rs146451123	1	4075519	C	29	7	CT	0.0001081
rs7514522	1	4752088	G	3	17	C	0.0005045
rs35923204	1	4766739	T	19	3	C	0.0005082
rs4654593	1	4769825	T	21	4	G	0.0008104
rs12760917	1	4770065	C	43	18	T	0.0003905
rs1061968	1	4772053	C	44	18	T	0.0002208
rs17345600	1	4999073	A	26	7	C	0.0007902
rs10915295	1	5001488	G	29	9	T	0.0008145
rs79342656	1	7035983	T	0	9	C	0.0009641
rs75077858	1	7037163	A	0	9	G	0.0009641
rs201655284	1	7044684	T	0	9	TACTC	0.0009641
rs11589210	1	7072945	T	0	9	C	0.0009641
rs142745042	1	11438910	C	0	10	T	0.0004292
rs637299	1	12219861	G	52	26	C	0.0005884
rs816060	1	12219910	G	52	26	A	0.0005884
rs1212890	1	12220369	A	30	49	G	0.0005383
rs622839	1	12220797	C	52	26	G	0.0005884
rs621938	1	12221026	T	52	26	C	0.0005884
rs150885757	1	12221069	CA	46	22	C	0.0002256
rs534146	1	12221606	G	52	26	A	0.0005884
rs3830763	1	14142002	AC	40	14	A	0.0001172
rs1810474	1	14145914	A	23	5	G	0.0006807
rs2744681	1	14149396	G	23	5	A	0.0006807
rs2697970	1	14149505	C	23	5	A	0.0006807
rs2697969	1	14149601	C	23	5	T	0.0006807
rs2697968	1	14149622	T	23	5	C	0.0006807
rs1046333	1	14149844	T	23	5	A	0.0006807
rs2697966	1	14150116	C	23	5	T	0.0006807
rs2697965	1	14150505	T	23	5	C	0.0006807
rs2744677	1	14154723	G	27	6	T	0.0001606
rs149580705	1	14154806	A	26	6	AGTAAACTTACCT	0.0003002
rs2697983	1	14157577	A	26	6	G	0.0003002
rs932369	1	14161921	T	23	5	A	0.0006807
rs1203690	1	14162023	T	23	5	G	0.0006807
rs1203686	1	14163428	A	21	4	G	0.0008104
rs68184904	1	14165191	G	25	6	C	0.000538
rs849609	1	16924589	A	35	12	G	0.0004754
rs2935931	1	17500227	T	45	20	A	0.0007326
rs2977255	1	17501651	C	45	20	T	0.0007326

rs3003452	1	17501720	C	45	20	G	0.0007326
rs35354876	1	17501814	CA	45	20	C	0.0007326
rs2977258	1	17501898	A	45	20	G	0.0007326
rs2935934	1	17502941	G	45	20	A	0.0007326
rs2935935	1	17502942	G	45	20	A	0.0007326
rs7529982	1	17503117	A	45	20	T	0.0007326
rs2977265	1	17503210	A	45	20	G	0.0007326
rs35497865	1	17504802	AT	45	20	A	0.0007326
rs2977276	1	17504846	A	45	20	T	0.0007326
rs2935921	1	17511243	A	45	20	G	0.0007326
rs2977247	1	17515804	T	45	20	C	0.0007326
rs2935942	1	17517255	C	45	20	T	0.0007326
rs3104441	1	17517831	C	45	20	A	0.0007326
rs3123530	1	17517833	A	45	20	T	0.0007326
rs370567292	1	22547086	A	0	10	G	0.0004292
rs72662681	1	26112371	A	0	9	G	0.0009641
1:33682038	1	33682038	C	0	9	T	0.0009641
rs17465854	1	33687320	C	0	9	T	0.0009641
rs72656286	1	33719199	G	0	9	T	0.0009641
rs143567912	1	34140788	G	2	14	T	0.0009649
rs1203148	1	35968899	G	9	30	A	0.00003688
rs1203147	1	35969652	C	9	25	A	0.0008322
rs6668196	1	36069204	G	1	12	A	0.0007887
rs6658272	1	38472841	T	26	7	C	0.0007902
1:40648426	1	40648426	C	0	10	T	0.0003596
rs61768443	1	44279579	G	54	28	A	0.0009655
rs147285662	1	53476248	A	0	10	AAT	0.0004292
rs200971546	1	53601827	C	4	23	A	0.00001525
rs143756558	1	54954041	CG	0	9	C	0.0009641
rs35361720	1	57526515	A	28	8	AT	0.0006092
rs17114917	1	57529790	A	29	9	G	0.0008145
rs35512026	1	57530093	T	29	9	C	0.0008145
rs7529536	1	57531021	A	29	9	G	0.0008145
rs11308788	1	57531622	C	29	9	CT	0.0008145
rs79534351	1	58501725	G	2	15	A	0.0004755
rs12122973	1	62642252	G	35	13	C	0.000996
rs4468199	1	65893960	G	1	12	A	0.0007887
rs75465244	1	65894940	G	1	12	A	0.0007887
rs1887285	1	65897747	G	1	12	A	0.0007887
rs77223187	1	65909606	G	1	12	A	0.0007887
rs74501836	1	76625210	G	2	14	A	0.0009649

rs76783328	1	76773440	G	0	11	T	0.0001895
rs12117719	1	79111194	T	25	6	C	0.0005533
1:83950538	1	83950538	C	26	7	G	0.0007902
rs4907075	1	84926639	G	4	19	A	0.0004818
rs9432839	1	84927162	T	4	19	C	0.0004818
rs7546947	1	85047535	T	4	18	G	0.0009242
rs72950380	1	85048148	A	4	18	C	0.0009242
rs12144566	1	87698441	A	32	51	C	0.0003437
rs138048727	1	87827916	G	4	20	GA	0.0001364
rs17129984	1	87828033	A	5	20	G	0.0004383
rs12138109	1	87839828	A	1	12	G	0.0007887
rs115448241	1	87842327	G	1	12	A	0.0007887
rs113509141	1	87844556	T	1	12	C	0.0007887
rs10518386	1	87847161	A	1	12	G	0.0007887
rs79251904	1	87848315	G	1	12	A	0.0007887
rs753231	1	87849684	C	2	14	G	0.0009649
rs76933147	1	87851606	C	1	12	A	0.0007887
rs75287360	1	87852689	A	1	12	G	0.0007887
rs76301958	1	87852723	G	1	12	T	0.0007887
rs75830465	1	87853862	T	1	12	C	0.0007887
rs12026304	1	87854121	A	2	14	T	0.0009649
rs11161998	1	87854436	G	2	14	A	0.0009649
rs113364398	1	87855351	T	1	12	C	0.0007887
rs12127125	1	87856069	G	1	12	A	0.0007887
rs11161999	1	87856133	A	2	14	G	0.0009649
rs12127255	1	87856501	G	1	12	A	0.0007887
rs79250690	1	87858551	A	1	12	T	0.0007887
rs370234031	1	87859619	A	1	12	AT	0.0007887
rs4500347	1	107083242	T	4	19	C	0.0004818
rs11184922	1	107106193	C	4	19	T	0.0004818
rs76529689	1	107109479	C	16	2	T	0.0008956
rs4573547	1	107140440	A	4	19	T	0.0004818
rs10430081	1	107179756	G	3	16	A	0.0009912
rs200827562	1	107184638	A	3	16	G	0.0005264
rs7527883	1	107200907	T	5	19	A	0.0008415
rs12065128	1	107201593	T	5	19	G	0.0008415
rs7515817	1	107204738	A	5	19	G	0.0008415
rs12060904	1	107206300	G	5	19	T	0.0008415
rs55885362	1	107206882	A	5	19	C	0.0008415
rs72982130	1	107207111	T	5	19	C	0.0008415
rs12066188	1	107207603	A	5	19	G	0.0008415

rs12077984	1	107208268	G	5	19	C	0.0008415
rs12062790	1	107208406	G	5	19	A	0.0008415
rs113588885	1	107208439	C	5	19	T	0.0008415
rs12063902	1	107208466	C	5	19	T	0.0008415
rs147299481	1	107208818	GA	5	19	G	0.0008415
rs12079433	1	107209364	T	5	19	C	0.0008415
rs72982138	1	107211035	A	5	19	T	0.0008415
rs112539959	1	107211704	T	5	19	C	0.0008415
rs80211416	1	107212319	T	5	19	G	0.0008415
rs12074685	1	107213259	T	5	19	G	0.0008415
rs72982144	1	107213759	A	5	19	G	0.0008415
rs12066688	1	107216073	G	5	19	A	0.0008415
rs72982148	1	107216419	C	5	19	A	0.0008415
rs11184960	1	107216719	C	5	19	A	0.0008415
rs56377258	1	107217276	A	5	19	G	0.0008415
rs72982150	1	107218105	G	5	19	A	0.0008415
rs6692251	1	107221859	G	5	19	A	0.0008415
rs12409401	1	107222522	T	5	19	C	0.0008415
rs41346254	1	107223280	C	5	19	T	0.0008415
rs72982166	1	107223588	A	5	19	C	0.0008415
rs4323743	1	107224678	C	5	19	T	0.0008415
rs4311915	1	107225176	C	5	19	T	0.0008415
rs72982173	1	107225472	T	5	19	C	0.0008415
rs4338391	1	107226805	A	5	19	T	0.0008415
rs17017407	1	107229999	C	5	19	A	0.0008415
rs59494924	1	107230598	A	5	19	G	0.0008415
rs72982182	1	107237310	T	5	19	G	0.0008415
rs141209417	1	107238006	T	5	19	TC	0.0008415
rs201979451	1	107238009	T	5	19	A	0.0008415
rs72982185	1	107240633	G	5	19	A	0.0008415
rs78413118	1	107241996	T	5	19	C	0.0008415
rs72982195	1	107250118	A	5	19	G	0.0008415
rs72984005	1	107252855	A	5	19	G	0.0008415
rs4614293	1	107256357	T	5	19	C	0.0008415
rs4424539	1	107256600	A	5	19	G	0.0008415
rs17017475	1	107259785	A	5	19	G	0.0008415
rs12124701	1	107358226	A	7	22	G	0.0009169
rs17482072	1	115912328	A	48	23	G	0.0008881
rs17541389	1	115916854	C	48	23	G	0.0008881
rs17541465	1	115918184	C	48	23	A	0.0008881
1:142909136	1	142909136	T	0	11	C	0.0001895

rs79597374	1	143275511	T	0	9	A	0.0009641
rs182686243	1	149034849	G	4	22	T	0.00003217
1:149458249	1	149458249	T	0	9	C	0.000946
rs116753436	1	151258314	C	2	16	T	0.0002312
rs11581057	1	156991688	C	23	4	T	0.0002248
rs11584902	1	157001597	C	23	4	G	0.0002248
rs34314027	1	157003864	C	23	4	T	0.0002248
1:157016023	1	157016023	GCCGCCA	22	3	G	0.0001254
rs150800619	1	157025409	CT	23	4	C	0.0002248
rs11577418	1	157026971	A	23	4	G	0.0002248
rs11576099	1	157027002	C	23	4	T	0.0002248
rs11576100	1	157027028	C	23	4	T	0.0002248
rs71593876	1	157027179	C	23	4	CT	0.0002248
rs35631733	1	157027288	T	23	4	TAA	0.0002248
rs11581893	1	157027520	T	23	4	C	0.0002248
rs35756545	1	157027944	T	23	4	C	0.0002248
rs12745721	1	157028212	T	23	4	G	0.0002248
rs34160895	1	157028313	A	21	4	AG	0.0004214
rs11582411	1	157028335	T	23	4	C	0.0002248
rs11577087	1	157028388	C	24	4	T	0.0001159
rs11578403	1	157028389	A	24	4	G	0.0001159
rs11578421	1	157028531	A	23	4	G	0.0002248
rs11577133	1	157028744	G	22	4	T	0.00043
rs11582484	1	157028809	A	23	4	C	0.0002248
rs866605	1	157028990	T	23	4	C	0.0002248
rs61326781	1	157029134	C	23	4	T	0.0002248
rs11577688	1	157029857	C	23	4	T	0.0002248
rs61329932	1	157030077	A	23	4	T	0.0002248
rs58824481	1	157030082	T	23	4	C	0.0002248
rs59571768	1	157030099	A	23	4	G	0.0002248
rs1706	1	157030280	C	23	4	T	0.0002248
rs11590493	1	157030627	C	22	4	T	0.00043
rs35256778	1	157031010	A	23	4	G	0.0002248
rs34994593	1	157031017	G	23	4	A	0.0002248
rs35045427	1	157031055	G	23	4	T	0.0002248
rs34230711	1	157031435	A	23	4	G	0.0002248
rs12735183	1	157031591	T	23	4	C	0.0002248
rs12759104	1	157031622	C	23	4	T	0.0002248
rs12734005	1	157031623	C	23	4	G	0.0002248
rs12759254	1	157031678	C	23	4	T	0.0002248
rs35209965	1	157031969	C	23	4	CT	0.0002248

rs12759732	1	157032001	C	23	4	T	0.0002248
rs35418037	1	157032593	A	23	4	G	0.0002248
rs59149386	1	157032706	T	23	4	C	0.0002248
rs34609359	1	157032832	C	23	4	T	0.0002248
rs34971986	1	157032883	G	23	4	A	0.0002248
rs11581819	1	157032995	T	23	4	C	0.0002248
rs11577808	1	157033027	A	23	4	G	0.0002248
rs11576914	1	157033294	A	23	4	T	0.0002248
rs11576533	1	157033505	G	23	4	A	0.0002248
rs35275017	1	157033610	C	24	4	CT	0.0001159
rs12731416	1	157033664	C	23	4	T	0.0002248
rs67062038	1	157033901	G	23	4	A	0.0002248
rs67278038	1	157033919	A	23	4	G	0.0002248
rs35025242	1	157033971	AT	22	4	A	0.00043
rs34177345	1	157034213	A	23	4	G	0.0002248
rs34065145	1	157034246	C	23	4	T	0.0002248
rs34438489	1	157034325	C	23	4	T	0.0002248
rs11582800	1	157034471	T	23	4	C	0.0002248
rs11577472	1	157034503	A	23	4	T	0.0002248
rs35043156	1	157034715	G	23	4	A	0.0002248
rs138311748	1	157035303	T	23	4	C	0.0002248
rs115482314	1	157035522	G	23	4	C	0.0002248
rs146206625	1	157035548	A	23	4	C	0.0002248
rs144424473	1	157035772	C	23	4	T	0.0002248
rs12743010	1	157036195	G	23	4	A	0.0002248
rs34047481	1	157036233	CA	23	4	C	0.0002248
rs12760516	1	157036266	T	23	4	G	0.0002248
rs12747244	1	157036322	C	23	4	T	0.0002248
rs12747418	1	157036424	A	23	4	T	0.0002248
rs71593878	1	157036573	C	23	4	CT	0.0002248
rs66524118	1	157036657	T	23	4	C	0.0002248
rs67992922	1	157036686	C	23	4	T	0.0002248
rs67871969	1	157036870	G	23	4	A	0.0002248
rs35310943	1	157036956	C	23	4	G	0.0002248
rs34330037	1	157036971	T	23	4	C	0.0002248
rs34951264	1	157037242	C	23	4	G	0.0002248
rs35958339	1	157037270	A	23	4	AT	0.0002248
rs60784227	1	157037779	G	23	4	T	0.0002248
rs57543447	1	157037957	G	23	4	A	0.0002248
rs61407874	1	157038008	G	23	4	A	0.0002248
rs12728942	1	157038163	C	23	4	G	0.0002248

rs12753932	1	157038227	G	23	4	A	0.0002248
rs12758039	1	157038250	G	23	4	T	0.0002248
rs36051715	1	157038624	C	23	4	G	0.0002248
rs12759047	1	157038865	G	23	4	T	0.0002248
rs12759364	1	157038997	C	23	4	T	0.0002248
rs12759394	1	157039061	C	23	4	T	0.0002248
rs12755356	1	157039073	G	23	4	A	0.0002248
rs12725372	1	157039400	C	23	4	T	0.0002248
rs12725382	1	157039437	C	23	4	T	0.0002248
rs12739927	1	157039462	A	23	4	C	0.0002248
rs12739240	1	157039741	A	23	4	G	0.0002248
rs35904515	1	157040203	A	23	4	G	0.0002248
rs34909260	1	157040296	G	23	4	A	0.0002248
rs12755131	1	157040527	G	23	4	C	0.0002248
rs12740642	1	157040552	C	23	4	T	0.0002248
rs12741140	1	157040837	C	23	4	T	0.0002248
rs12755959	1	157041010	T	23	4	C	0.0002248
rs12737408	1	157041078	G	23	4	A	0.0002248
rs12756141	1	157041107	T	23	4	C	0.0002248
rs12760052	1	157041181	G	23	4	C	0.0002248
rs12760229	1	157041302	T	23	4	C	0.0002248
rs56801406	1	157044993	G	26	5	C	0.0001012
rs59462822	1	157045213	A	26	5	G	0.0001012
rs35188764	1	157046198	G	23	3	A	0.00006268
rs36032506	1	157046248	T	23	4	C	0.0002248
rs34074614	1	157047366	C	23	4	T	0.0002248
rs11587380	1	157048220	G	26	5	C	0.0001012
rs11587404	1	157048354	T	23	4	C	0.0002248
rs34961273	1	157050194	G	23	4	A	0.0002248
rs12752535	1	157050368	T	23	4	G	0.0002248
rs11578226	1	157051348	C	26	5	T	0.0001012
rs34328576	1	157051761	CCCT	23	4	C	0.0002248
rs149171885	1	157052071	TCAGTTTGGCATT	26	5	T	0.0001012
rs34414479	1	157052224	A	26	5	G	0.0001012
rs34369222	1	157052352	G	23	4	A	0.0002248
rs35090422	1	157052784	G	23	4	GA	0.0002248
rs34542108	1	157052794	T	23	4	C	0.0002248
rs11580245	1	157052987	A	23	4	G	0.0002248
rs35043649	1	157053226	ATT	23	4	A	0.0002248
rs1176561	1	157053342	T	23	3	C	0.00006268
rs12080191	1	157053415	T	23	4	C	0.0002248

rs11580501	1	157055760	C	23	4	T	0.0002248
rs67980707	1	157060544	G	23	4	A	0.0002248
rs67947469	1	157061643	T	23	4	C	0.0002248
rs12136960	1	157062739	G	30	7	C	0.0000683
rs12749198	1	157074813	A	18	3	G	0.0009486
rs11585088	1	160975049	G	5	20	A	0.0004383
rs67414804	1	160988485	T	6	23	G	0.0001763
rs72714913	1	161117902	G	3	16	A	0.0009912
rs10918046	1	164526190	G	14	1	A	0.0007094
rs11800702	1	170335130	A	31	9	G	0.0002479
rs28782297	1	170924418	A	5	19	G	0.0008415
rs146426777	1	170924776	T	5	19	C	0.0008415
rs76289818	1	170939874	T	5	19	A	0.0007163
rs9427208	1	170940240	T	5	19	C	0.0008415
rs143694065	1	170969416	GT	2	15	G	0.0004755
rs11488713	1	170972335	A	2	15	T	0.0004755
rs9426924	1	170973094	T	2	15	C	0.0004755
rs149697643	1	170973559	G	2	15	GC	0.0004755
rs11486182	1	170975587	A	2	15	G	0.0004755
rs61816860	1	170979240	A	2	15	G	0.0004755
rs9426925	1	170982001	G	2	15	A	0.0004755
rs41310903	1	170983976	T	2	15	C	0.0004755
rs72710570	1	170985995	C	2	15	A	0.0004755
rs72710571	1	170986343	T	2	15	C	0.0004755
rs56053729	1	170986415	T	2	15	G	0.0004755
rs55847906	1	170986416	T	2	15	C	0.0004755
rs72710573	1	170987674	G	2	15	A	0.0004755
rs72710575	1	170988418	A	2	15	G	0.0004755
rs61336965	1	170989327	C	2	15	CAA	0.0004755
rs114283104	1	170989942	A	2	15	G	0.0004755
rs116494685	1	170990527	G	2	15	A	0.0004755
rs56225411	1	170990995	T	2	14	C	0.0009649
rs16863948	1	170992100	A	2	15	G	0.0004755
rs16863949	1	170992602	C	2	15	A	0.0004755
rs61816864	1	170992716	A	2	15	T	0.0004755
rs72710577	1	170993313	A	2	15	G	0.0004755
1:170993440	1	170993440	ACGG	2	15	A	0.0004755
rs16863950	1	170994761	A	2	15	G	0.0004755
rs151073251	1	170995015	TC	2	15	T	0.0004755
rs146990598	1	170995639	TC	2	15	T	0.0004755
rs72710580	1	170995732	A	2	15	G	0.0004755

rs114765529	1	170997401	T	2	15	G	0.0004755
rs72710581	1	170999936	G	2	15	A	0.0004755
rs56346740	1	171002346	C	2	15	G	0.0004755
rs61816868	1	171004783	T	2	15	C	0.0004755
rs72710583	1	171005464	G	2	15	A	0.0004755
rs61816869	1	171005760	T	2	15	C	0.0004755
rs78664997	1	175165977	T	0	11	C	0.0001895
rs640496	1	183157036	C	24	43	T	0.000471
rs495418	1	183159664	A	24	43	G	0.000471
1:183160328	1	183160328	TA	24	43	T	0.0007782
rs594026	1	183160477	T	24	43	G	0.000471
rs687988	1	183161310	T	24	43	G	0.000471
rs481023	1	183161869	T	24	42	G	0.0008352
rs672525	1	183162450	A	24	43	G	0.000471
rs509826	1	183162705	A	24	43	G	0.000471
rs657869	1	183163430	T	24	43	G	0.000471
rs2477439	1	183164905	C	24	43	A	0.000471
1:183164928	1	183164928	GTTTAATGTGATG	24	42	G	0.0008352
1:183164934	1	183164934	T	24	42	G	0.0008352
1:183164935	1	183164935	G	24	42	GC	0.0008352
rs61479242	1	183164937	A	24	42	C	0.0008352
rs572977	1	183164991	G	24	43	C	0.000471
rs10911276	1	183165358	C	23	43	T	0.0002487
rs1760628	1	183165505	C	24	43	G	0.000471
rs1760629	1	183165518	T	24	43	G	0.000471
rs627064	1	183165716	T	24	43	C	0.000471
rs35890410	1	183165895	GA	24	43	G	0.000471
rs519350	1	183166573	A	24	43	G	0.000471
rs596263	1	183166582	A	24	43	G	0.000471
rs3118182	1	183167175	G	24	43	A	0.000471
rs473789	1	183169032	T	25	43	A	0.0008649
rs12724111	1	183169122	G	21	40	A	0.0006238
rs1760632	1	183169386	G	25	43	A	0.0008649
rs661451	1	183169690	C	26	44	T	0.0008911
rs648341	1	183170353	C	25	43	T	0.0008649
rs570415	1	183171316	A	23	43	G	0.0002487
rs4549996	1	186707043	C	8	25	G	0.0006969
rs76858875	1	188667613	T	16	2	G	0.0008912
rs71519083	1	189717270	A	24	48	T	0.0000187
rs71519084	1	189717272	A	24	48	T	0.0000187
rs71637213	1	189717274	A	24	48	T	0.0000187

1:192580429	1	192580429	C	45	20	A	0.0007326
rs56262835	1	198237275	CT	16	2	C	0.0008956
1:203595404	1	203595404	TGG	25	6	T	0.0005533
rs77861062	1	208286360	C	0	9	A	0.0009641
rs4844851	1	209717109	T	24	42	G	0.0008352
rs11119358	1	210042456	C	9	25	T	0.0008942
rs7522894	1	211082050	A	1	13	G	0.0003716
rs12028941	1	211088474	T	0	13	C	0.00003603
rs12021704	1	211088631	C	1	13	G	0.0003716
rs59806283	1	211088915	G	1	15	GC	0.00007965
rs12030905	1	211090360	T	1	15	C	0.00007965
rs61848992	1	211091849	T	1	15	C	0.00007965
rs2358210	1	211094834	C	2	15	T	0.0004755
rs57655345	1	211108933	C	2	15	T	0.0004755
rs12036999	1	211112257	T	0	10	C	0.0004292
rs6540636	1	211120598	A	2	14	G	0.0009649
rs6675213	1	211121045	A	1	12	C	0.0007887
rs61849000	1	211121274	T	1	12	C	0.0007887
rs6678568	1	211122469	C	2	14	T	0.0009649
rs7554723	1	211129493	C	1	12	T	0.0007887
rs117202850	1	211129598	T	0	10	C	0.0004292
rs6666287	1	211130853	T	1	12	G	0.0007887
rs12039302	1	211133122	A	0	10	G	0.0004292
rs7551412	1	211134593	T	1	13	A	0.0003716
rs7553997	1	211134663	C	1	13	T	0.0003716
rs7518841	1	211138059	G	1	12	T	0.0007887
rs6540638	1	211139765	C	1	12	A	0.0007887
rs56999636	1	211141038	A	0	10	T	0.0004292
rs12406562	1	211142718	C	1	13	T	0.0003716
rs74422102	1	211149108	C	0	10	A	0.0004292
rs7523929	1	211149360	C	1	12	T	0.0007887
rs61849024	1	211152436	T	1	12	A	0.0007887
rs1501544	1	211153252	A	1	12	T	0.0007887
rs1501545	1	211153638	A	1	12	G	0.0007887
rs7515316	1	211156440	T	1	17	C	0.00001632
rs7529625	1	211156698	C	1	17	T	0.00001632
rs7537597	1	211156775	C	1	17	G	0.00001632
rs6663672	1	211157628	T	1	17	C	0.00001632
rs148749633	1	211158461	T	0	10	C	0.0004292
rs75986786	1	211158739	T	1	17	C	0.00001632
rs77208679	1	211158768	G	1	16	A	0.00003625

rs10863872	1	211159950	G	1	17	A	0.00001632
rs10863873	1	211159985	C	1	17	T	0.00001632
rs150093062	1	211160642	G	1	17	A	0.00001632
rs6671367	1	211161351	C	1	17	A	0.00001632
rs12043937	1	211161502	G	1	17	A	0.00001632
rs12043952	1	211161510	C	1	17	A	0.00001632
rs6659020	1	211161526	T	1	17	C	0.00001632
rs6659135	1	211161618	T	1	16	C	0.00003625
rs6680997	1	211163369	G	1	16	T	0.00003625
rs12070402	1	211165752	T	1	17	C	0.00001632
rs1532682	1	211166426	G	1	17	T	0.00001632
rs142791118	1	211167308	GA	1	16	G	0.00003625
rs76610265	1	211167315	G	1	16	T	0.00003625
rs12022715	1	211167954	C	1	17	T	0.00001632
rs12022739	1	211168085	C	1	17	T	0.00001632
rs1501548	1	211168634	C	1	17	T	0.00001632
rs1501549	1	211168669	T	1	17	G	0.00001632
rs1501550	1	211168759	A	1	17	G	0.00001632
rs11119654	1	211168993	G	1	17	A	0.00001632
rs6687069	1	211169973	G	1	17	C	0.00001632
rs6689715	1	211170209	T	1	17	C	0.00001632
rs145184581	1	211173970	C	1	17	A	0.00001632
rs35683927	1	211175219	A	1	17	G	0.00001632
rs34279520	1	211175222	G	1	17	A	0.00001632
1:211175223	1	211175223	GA	1	17	G	0.00001632
rs12403500	1	211175373	A	1	17	G	0.00001632
rs12026311	1	211176612	A	1	16	T	0.00003625
rs12025525	1	211176618	G	1	16	A	0.00003625
rs12026374	1	211176816	A	1	16	T	0.00003625
rs12027535	1	211178147	G	1	17	T	0.00001632
rs12030715	1	211178374	A	1	16	G	0.00003625
rs11806852	1	211178828	A	1	16	C	0.00003625
rs78743738	1	211180810	C	1	16	T	0.00003625
rs61850228	1	211183952	A	1	17	T	0.00001632
rs61848562	1	211187679	T	1	16	C	0.00003625
rs139001420	1	211190264	AAAAAT	1	12	A	0.0007119
rs7514606	1	211191730	G	1	16	C	0.00003625
rs57179129	1	211194404	T	1	16	C	0.00003625
rs201353335	1	211196494	A	0	10	AAATAAATAAATT	0.0004869
rs11119666	1	211201592	A	6	22	G	0.000342
rs1604371	1	211203732	G	6	22	T	0.000342

rs4575146	1	211204122	T	1	16	A	0.00003625
rs4570464	1	211204187	C	1	16	A	0.00003625
rs6688920	1	211205507	A	1	16	C	0.00003625
rs75983525	1	211205657	C	1	16	T	0.00003625
rs183507111	1	211205883	G	1	17	A	0.00001632
rs75886883	1	211208652	A	0	9	G	0.0009641
rs61848568	1	211208841	T	1	16	C	0.00003625
rs6666342	1	211209023	G	1	17	T	0.00001632
rs57045219	1	211209354	T	3	18	C	0.0002532
rs58255417	1	211209457	T	1	17	C	0.00001632
rs11119668	1	211209776	G	1	17	A	0.00001632
rs6540648	1	211210280	A	1	17	C	0.00001632
rs114901383	1	216622783	A	11	0	G	0.0007855
1:221854004	1	221854004	A	5	21	ACAC	0.0002135
1:223624884	1	223624884	G	17	2	A	0.0004076
rs5050	1	230849886	G	3	16	T	0.0009912
rs6683321	1	230851628	A	3	16	G	0.0009912
rs12568273	1	234506616	G	14	36	A	0.00003962
rs45577939	1	234509865	G	21	43	T	0.0001081
rs4300255	1	234510262	G	21	42	A	0.0001202
rs7554965	1	234511541	A	14	38	G	0.000009956
rs11285466	1	234512010	T	14	38	TC	0.000009956
rs6671513	1	234512169	G	14	38	A	0.000009956
rs10429821	1	234512471	C	25	43	T	0.0008649
rs10429823	1	234512583	C	14	38	T	0.000009956
rs6681436	1	234514025	C	14	38	T	0.000009956
rs6664462	1	234514485	T	14	38	G	0.000009956
rs10797537	1	234514613	T	14	36	G	0.00003962
rs10797538	1	234514672	A	13	35	G	0.00001184
rs12024011	1	234514725	A	11	36	G	0.000003952
rs55884696	1	234515265	T	14	38	C	0.000009956
rs6684927	1	234516617	A	14	38	G	0.000009956
rs4477318	1	234517227	T	14	38	A	0.000009956
rs12401853	1	234517474	C	15	38	A	0.00002412
rs149002825	1	234518776	AGAGAAG	11	34	A	0.00001988
rs10797539	1	234519385	C	14	38	T	0.000009956
rs60111174	1	234520784	ACTT	14	38	A	0.000009956
rs10910424	1	234521024	T	14	38	C	0.000009956
rs10910425	1	234521118	C	14	38	T	0.000009956
rs10752785	1	234521718	T	22	41	G	0.0004309
rs7527165	1	234524045	T	14	38	C	0.000009956

rs12023971	1	234525333	A	14	38	G	0.000009956
rs6669190	1	234525940	C	29	48	T	0.0005332
rs12402835	1	234526732	C	14	38	G	0.000009956
rs3862040	1	234527575	T	24	46	C	0.0000732
rs7523423	1	234528373	T	24	46	C	0.0000732
rs2175594	1	234529552	T	23	47	C	0.00001794
rs2175593	1	234529570	A	23	46	G	0.00003593
rs10910426	1	234530325	G	24	46	A	0.0000732
rs10797540	1	234530370	A	24	46	G	0.0000732
rs12031728	1	234537992	A	4	18	G	0.0009242
rs10910427	1	234539157	T	24	46	C	0.0000732
rs3768288	1	234540547	T	50	25	C	0.0009811
rs1141264	1	234546245	T	19	43	C	0.00002243
rs12035712	1	234555310	C	12	30	T	0.0005652
rs10797541	1	234555447	A	12	30	G	0.0005652
rs6661926	1	234556630	T	13	30	C	0.0007578
rs6669819	1	234556646	A	13	30	T	0.0007578
rs6666922	1	234556774	T	12	30	A	0.0005652
rs6703763	1	234556984	A	12	30	G	0.0005652
rs6703898	1	234557171	C	12	30	G	0.0005652
rs6665103	1	234557360	T	12	30	C	0.0005652
rs750813	1	234557918	A	12	30	C	0.0005652
rs750814	1	234558430	G	12	30	A	0.0005652
rs6676736	1	234558771	C	12	30	T	0.0005652
rs6676309	1	234558966	T	12	30	A	0.0005652
rs6676815	1	234559397	G	12	30	A	0.0005652
rs3820603	1	234559804	T	12	30	A	0.0005652
rs10910430	1	234560927	G	12	30	C	0.0005652
rs6429207	1	235798334	A	44	19	C	0.0006784
rs4660139	1	236155817	C	16	35	T	0.000362
rs12074023	1	240631508	A	1	12	G	0.0008669
rs7529566	1	246130744	C	8	27	A	0.00009961
rs2066028	1	246130872	T	8	27	C	0.00009961
rs61839381	1	246133416	A	8	27	G	0.00009961
rs146192520	1	246134737	TA	8	27	T	0.00009339
rs61839384	1	246134977	G	8	27	C	0.00009961
rs11576666	1	246136553	G	8	27	A	0.00009961
rs11811545	1	246136870	A	12	31	C	0.0001903
rs10802305	1	246142250	T	8	26	C	0.0001919
rs10754491	1	246142279	C	14	33	T	0.0002703
rs199605639	1	246171117	CA	1	14	C	0.0002194

rs12993335	2	2424210	A	45	20	G	0.0007326
rs62119788	2	3057257	A	12	0	T	0.0003752
rs202066201	2	3528553	C	32	10	CTT	0.0003432
rs1622254	2	7130521	G	1	17	A	0.00001859
2:7130525	2	7130525	G	1	15	A	0.0001027
2:7130527	2	7130527	G	1	13	A	0.0003716
2:7130528	2	7130528	C	0	13	CACACACACACAC	0.00003603
rs138874463	2	11151955	A	9	27	ACAAGAACTTACG	0.0002633
rs114088839	2	23828157	A	0	9	G	0.0009641
rs6546911	2	26545067	C	52	28	T	0.0008402
rs35337085	2	29581969	T	14	31	TA	0.0006984
rs207423	2	31547084	G	40	16	A	0.0005221
rs35733574	2	31547119	C	40	16	CTTTT	0.0005221
rs207424	2	31547238	A	40	16	G	0.0005221
rs635581	2	31549213	T	39	16	G	0.0009032
rs13385162	2	31724929	T	15	35	C	0.0001708
rs11684698	2	31727524	A	15	35	T	0.0001708
rs4952218	2	31731120	A	15	35	T	0.0001708
rs2365778	2	31738219	T	28	46	C	0.0009321
rs6543630	2	31740034	A	14	33	G	0.0002703
rs56175176	2	31741566	T	14	32	C	0.0004925
rs1475054	2	31741965	G	14	32	C	0.0004925
rs6760199	2	31742278	A	14	32	C	0.0004925
rs2224310	2	31742467	C	14	32	T	0.0004925
rs2208158	2	31742862	T	14	32	A	0.0004925
rs4952197	2	31767131	A	14	31	G	0.0008795
rs725631	2	31781026	A	17	35	C	0.0007327
rs58811103	2	31801180	G	17	35	C	0.0007327
rs2754530	2	31803403	T	17	36	C	0.0004114
rs2268799	2	31803495	T	17	36	C	0.0004114
rs806645	2	31807940	T	17	36	C	0.0004114
rs11403363	2	31807998	A	17	35	AT	0.0007327
rs502139	2	31811268	T	17	35	C	0.0007327
rs633376	2	31815191	T	15	34	G	0.0003148
rs809364	2	31815453	A	15	34	G	0.0003148
rs646674	2	31815844	A	15	33	G	0.0005689
rs1090846	2	31816341	T	15	33	C	0.0005689
rs75688847	2	31816734	C	17	36	T	0.0004114
rs600015	2	31820300	G	17	37	C	0.0002264
rs607294	2	31832807	T	19	37	C	0.0009069
rs623419	2	31834078	A	19	37	G	0.0009069

rs655548	2	31836658	A	18	36	C	0.0008188
rs477517	2	31848637	C	17	35	T	0.0007327
rs614173	2	31853202	C	18	36	T	0.0008188
rs546935	2	31853674	A	17	37	G	0.0002264
rs35826411	2	31859226	C	16	34	CA	0.0006492
rs71446010	2	31859714	C	2	17	T	0.000111
rs481344	2	31859919	A	16	34	G	0.0006492
rs681482	2	31871256	A	17	36	G	0.0004114
rs589427	2	31872513	A	17	35	T	0.0007327
rs553464	2	31873476	G	21	40	A	0.0006238
rs68057272	2	31875044	AGATT	20	39	A	0.0005694
rs12479192	2	31923042	T	16	34	C	0.0006492
rs1580329	2	32016677	G	17	36	A	0.0004114
rs223675	2	32026249	A	13	32	G	0.0002287
rs34450929	2	32034082	A	2	15	G	0.0004755
rs114467817	2	32083640	C	3	16	T	0.0009912
rs10779914	2	32106040	A	15	33	C	0.0005689
rs11124272	2	32142520	A	16	34	C	0.0006492
rs61144237	2	32150343	A	9	25	G	0.0008942
rs79379878	2	32152861	C	3	17	A	0.0005045
rs2082185	2	32155175	G	13	30	A	0.0007578
rs75440961	2	32219573	C	15	33	T	0.0005689
rs12995113	2	32221763	C	3	17	T	0.0005045
rs71446036	2	32226986	A	15	33	G	0.0005689
rs12478134	2	32240783	G	14	31	C	0.0008795
rs34702054	2	32256999	C	13	30	CA	0.0007578
rs34965238	2	32285441	G	15	33	GA	0.0005689
rs2280968	2	32290040	T	14	33	G	0.0002703
rs13017027	2	32291903	T	11	30	C	0.0002453
rs7556942	2	32295694	A	14	33	G	0.0002703
rs17011801	2	32298072	T	14	33	A	0.0002703
rs35112079	2	32302079	A	14	33	G	0.0002703
rs11695080	2	32316886	C	10	27	G	0.0006386
rs7557034	2	32316910	A	12	30	T	0.0005652
rs3769605	2	32322456	G	14	33	A	0.0002703
rs200363219	2	32327754	A	14	34	G	0.0001454
rs6543648	2	32330314	A	14	33	G	0.0002703
rs11676456	2	32331511	G	11	30	A	0.0002453
2:32336317	2	32336317	A	0	9	ATATAGTGTGTAT	0.0008243
rs6756085	2	32336522	G	14	33	A	0.0002703
rs13010405	2	32338204	T	13	32	A	0.0003918

rs11691939	2	32339561	T	14	33	C	0.0002703
rs7574000	2	32342888	C	14	33	A	0.0002703
rs56322034	2	32353013	C	15	33	T	0.0005689
rs2294178	2	32353324	C	15	33	A	0.0005689
rs13006495	2	32355490	T	15	33	C	0.0005689
rs13006880	2	32355539	A	11	30	G	0.0002453
rs12996943	2	32373645	C	15	32	G	0.000877
rs34197800	2	32389646	A	15	33	C	0.0005689
rs13013695	2	32391231	A	12	30	G	0.0005652
rs35978916	2	32400635	C	14	31	T	0.0008795
rs212742	2	32408629	T	15	33	C	0.0005689
rs375050	2	32413765	C	15	33	T	0.0005689
rs212751	2	32418808	T	15	33	A	0.0005689
rs212754	2	32421628	A	15	33	G	0.0005689
rs212761	2	32429288	T	16	35	C	0.000362
rs186135	2	32432578	C	15	33	T	0.0005689
rs212682	2	32432864	A	15	33	G	0.0004934
rs212683	2	32432934	A	14	33	T	0.0002703
rs212684	2	32433058	G	15	33	C	0.0005689
rs212685	2	32434701	A	15	33	G	0.0005689
rs177082	2	32438350	A	15	32	T	0.000877
rs212702	2	32444250	A	12	30	G	0.0005652
rs61393580	2	32461937	A	15	33	AAAAG	0.0005689
rs212736	2	32473126	T	12	30	C	0.0005652
rs410469	2	32476992	T	14	33	G	0.0002703
rs7562653	2	32478629	T	14	33	C	0.0002703
rs385076	2	32489851	T	16	36	C	0.0001978
rs5830226	2	32493527	C	15	33	CAT	0.0005689
rs1035025	2	37949160	C	53	27	T	0.0006109
rs6544102	2	37951123	G	30	49	A	0.0005383
rs6544103	2	37951249	G	30	49	A	0.0005383
rs201262385	2	37952621	A	43	19	G	0.000761
rs149254951	2	37953287	G	30	49	A	0.0005383
rs143024314	2	37954040	A	30	49	G	0.0005383
rs148517021	2	37954483	A	43	19	C	0.000761
rs6544104	2	37954827	C	30	49	T	0.0005383
rs7587427	2	37955010	A	30	49	T	0.0005383
rs4450584	2	37955267	C	30	49	T	0.0005383
rs11891125	2	37955417	G	30	49	C	0.0005383
rs11692897	2	37956018	A	30	49	C	0.0005383
rs232549	2	38346736	A	3	16	G	0.0009912

rs2432663	2	38351642	T	3	16	C	0.0009912
rs2447750	2	38352037	T	3	16	A	0.0009912
rs2432662	2	38352174	T	3	16	C	0.0009912
rs232623	2	38357292	T	3	16	C	0.0009912
rs170818	2	38374567	T	4	18	C	0.0009242
rs232598	2	38376966	A	4	18	T	0.0009242
rs10177042	2	38563527	G	37	14	C	0.0006739
2:43290255	2	43290255	G	15	1	C	0.0004612
rs7558342	2	46015851	T	32	50	G	0.0009655
rs75996100	2	46045223	T	1	12	C	0.0007887
rs13431476	2	46045551	T	1	12	C	0.0007887
rs72808733	2	46322160	A	8	25	G	0.0003633
rs67290491	2	46322166	G	8	25	C	0.0003633
rs66628040	2	46322232	A	8	25	C	0.0003633
rs67425308	2	46323103	A	8	25	G	0.0003633
rs17799476	2	46352326	G	4	18	C	0.0009242
rs34606309	2	46364687	T	7	24	C	0.0002977
rs61087417	2	47813986	A	0	9	T	0.0009641
rs17039159	2	49883594	C	2	15	T	0.0004755
rs10495981	2	49883809	C	2	15	T	0.0004755
rs75020896	2	49886012	G	2	15	T	0.0004755
rs76523299	2	49892510	C	2	14	G	0.0009649
rs55642256	2	49893646	C	2	14	T	0.0009649
rs4271798	2	49893851	G	2	14	T	0.0009649
rs76612430	2	49893866	C	2	14	T	0.0009649
rs2350700	2	49894534	C	2	14	T	0.0009649
rs76943415	2	49895770	C	2	14	T	0.0009649
rs116359123	2	49896274	G	2	15	A	0.0004755
rs56096801	2	49897226	C	2	14	T	0.0009649
rs55650884	2	49897367	T	2	14	C	0.0009649
rs76197520	2	49901119	T	2	15	C	0.0004755
rs55754993	2	49939073	G	2	15	A	0.0004755
rs17039201	2	49947538	T	2	14	C	0.0009649
rs72880459	2	49953640	A	2	15	T	0.0004755
rs1401325	2	49966481	C	2	14	G	0.0009649
rs6710424	2	49970721	C	2	14	T	0.0009649
rs72880477	2	49970967	C	2	14	T	0.0009649
rs12328055	2	49971239	A	2	14	G	0.0009649
rs59645701	2	49978243	G	2	14	A	0.0009649
rs10596176	2	50302053	G	0	9	GTT	0.0009641
rs17838395	2	50329142	G	0	9	A	0.0009641

2:50608662	2	50608662	T	7	22	C	0.0009169
rs1468986	2	53861263	T	50	25	A	0.0009811
rs35437504	2	54616263	G	1	13	A	0.0003716
rs201701440	2	59148035	CT	18	2	C	0.0002408
rs10490101	2	59149939	G	18	3	C	0.0009486
rs76040012	2	59150981	C	19	3	T	0.0005082
2:63777368	2	63777368	C	6	26	CACACA	0.00002181
2:63777371	2	63777371	C	6	23	A	0.0001763
rs1867848	2	63777373	C	6	21	A	0.0006518
2:65887032	2	65887032	GGCTAAAAACGG	0	9	G	0.0009641
rs6546916	2	74872278	A	0	9	C	0.0009641
rs34588873	2	76238523	T	7	26	G	0.00006834
rs7603333	2	79357595	A	29	47	G	0.0009467
rs77470225	2	84478792	A	0	9	G	0.0009641
rs112124898	2	84581600	A	0	12	G	0.00008299
rs77159567	2	84614874	A	0	12	T	0.00008299
rs11126962	2	84638116	C	0	12	A	0.00008299
2:84647251	2	84647251	T	0	10	TA	0.0004292
rs142296229	2	84672087	T	0	12	C	0.00008299
rs76990652	2	84676268	C	0	11	T	0.0001895
rs111878765	2	84714899	A	0	12	G	0.00008299
rs11687501	2	85009989	A	0	11	G	0.0001895
rs183611857	2	85056725	A	0	10	G	0.0004292
rs11686064	2	85063529	A	0	10	G	0.0004292
rs192817406	2	85232419	T	1	12	A	0.0007119
rs75358661	2	85610056	G	0	10	A	0.0003694
rs57603945	2	88835972	G	21	4	C	0.0008104
2:89076570	2	89076570	C	11	0	G	0.0007855
2:91634301	2	91634301	T	2	14	G	0.0009649
2:92295219	2	92295219	G	0	9	A	0.0009641
rs79926110	2	96628770	G	1	12	A	0.0007887
2:104263114	2	104263114	A	4	18	G	0.0009446
rs75852181	2	105363121	C	0	9	T	0.0009641
rs143223356	2	108973424	GT	11	28	G	0.0008103
rs6755756	2	109028656	G	11	28	A	0.0008103
rs59581093	2	109029003	CA	0	10	C	0.0004292
rs28413002	2	109031732	C	2	14	T	0.0009649
rs62151377	2	109289585	C	2	16	A	0.0002312
rs111522677	2	112047086	A	46	19	G	0.0002121
rs3891956	2	112047880	A	37	10	G	0.00002452
rs201218945	2	112047920	A	37	10	G	0.00002452

rs143703637	2	112048156	C	36	9	G	0.000008319
rs146039164	2	112048665	G	38	10	A	0.00001243
2:112054683	2	112054683	A	29	8	AAGCACTCAACA/	0.0003391
rs58475388	2	112070553	C	52	24	A	0.0001653
rs62162862	2	112070850	C	52	24	A	0.0001653
rs2139360	2	112071399	C	52	24	T	0.0001653
rs62162863	2	112071459	T	52	24	G	0.0001653
2:112071914	2	112071914	G	49	23	A	0.0002681
rs140607302	2	112071996	A	52	24	G	0.0001653
rs3814029	2	112073184	C	49	20	T	0.00006674
rs200296231	2	112141350	T	44	17	C	0.000106
rs78236988	2	112141772	C	44	17	T	0.000106
rs202225640	2	112145399	A	44	17	G	0.000106
rs116317168	2	112150554	C	44	17	T	0.000106
rs4849389	2	116003178	A	19	3	G	0.0005082
rs142458858	2	116116096	C	11	0	T	0.0007855
rs13022237	2	116416128	G	10	29	A	0.0001875
rs13027654	2	116416368	G	10	29	A	0.0001875
rs13022167	2	116419585	C	10	29	G	0.0001875
2:117350749	2	117350749	T	7	24	C	0.0002593
2:117350751	2	117350751	T	7	24	C	0.0002593
rs661500	2	119449361	A	5	19	C	0.0008415
rs512681	2	119450163	A	5	19	C	0.0008415
rs162752	2	119473589	T	7	22	A	0.0009169
rs12622890	2	142456472	A	34	12	G	0.000839
rs6721469	2	142591851	T	21	40	G	0.0006238
rs6721397	2	142591936	A	21	40	C	0.0006238
rs10191067	2	142592379	C	21	40	G	0.0006238
rs10175758	2	146703546	T	11	0	C	0.0007837
rs12476278	2	148123250	A	4	18	G	0.0009242
rs41420149	2	148138416	A	4	18	G	0.0009242
rs77388016	2	151733840	T	1	14	C	0.000173
rs12151724	2	151734715	A	1	14	T	0.000173
rs79308412	2	151737087	A	1	14	G	0.000173
rs75482237	2	151738987	T	2	15	C	0.0004755
rs77834577	2	151740507	G	2	15	A	0.0004755
rs2190381	2	151743024	G	2	15	T	0.0004755
rs2214898	2	151743382	A	2	15	T	0.0004755
rs78904109	2	151744806	T	2	15	C	0.0004755
2:151745438	2	151745438	C	2	15	CAGAG	0.0002125
2:151745446	2	151745446	CAGAG	2	15	C	0.0004755

rs77142918	2	151749662	T	2	15	C	0.0004755
rs78009712	2	151753877	G	2	15	A	0.0004755
rs75229806	2	151755090	A	2	15	C	0.0004755
rs79953851	2	151755522	C	2	15	A	0.0004755
rs74568159	2	151756126	C	2	15	T	0.0004755
rs76518694	2	151760617	T	0	13	C	0.00003603
rs78507847	2	151761128	A	1	13	G	0.0003716
rs78591232	2	151761540	T	3	18	C	0.0002532
rs75158222	2	151762639	G	1	13	A	0.0003716
rs60076148	2	151767049	G	52	26	T	0.0005884
rs1405934	2	155294658	C	16	2	T	0.0008956
rs1527862	2	155294795	T	16	2	C	0.0008956
rs1527861	2	155294870	A	16	2	G	0.0008956
rs10203804	2	155603154	A	19	37	G	0.0009069
rs4991002	2	155993849	G	15	33	A	0.0005689
rs77029648	2	161541408	C	0	9	T	0.0009641
rs77706588	2	161541956	G	0	9	A	0.0009641
rs77893985	2	161541961	G	0	9	A	0.0009641
rs80008796	2	161543287	C	0	9	T	0.0009641
rs77213830	2	161543637	A	0	9	C	0.0009641
rs5835831	2	161545040	C	0	9	CATT	0.0009641
rs4072752	2	161545135	C	0	9	T	0.0009641
rs12612157	2	161545838	G	0	9	A	0.0009641
rs139886426	2	161547808	GGT	0	10	G	0.0004292
rs78974879	2	161548402	T	0	9	C	0.0009641
rs12622288	2	161549843	T	0	9	C	0.0009641
rs7420949	2	161549993	C	0	9	T	0.0009641
rs12618371	2	161550899	A	0	9	C	0.0009641
rs143582228	2	161551338	G	0	9	GT	0.0009641
rs75879039	2	161552986	A	0	9	G	0.0009641
rs75620633	2	161553675	G	0	9	A	0.0009641
rs74741712	2	161554360	C	0	9	A	0.0009641
rs4286290	2	161554761	G	0	9	A	0.0009641
rs4467285	2	161554836	G	0	9	T	0.0009641
rs4312509	2	161555014	T	0	9	C	0.0009641
rs77567548	2	161555226	T	0	9	C	0.0009641
rs12613593	2	161556205	A	0	9	T	0.0009641
rs75365785	2	161557815	G	0	9	A	0.0009641
rs76391215	2	161558215	T	0	9	C	0.0009641
rs75313956	2	161558976	G	0	9	T	0.0009641
rs79226712	2	161559188	C	0	9	T	0.0009641

rs74268459	2	161559640	A	0	9	G	0.0009641
rs75870328	2	161559814	AT	0	9	A	0.0009641
rs78550197	2	161561105	T	0	9	C	0.0009641
rs74268460	2	161562594	T	0	9	A	0.0009641
rs77082406	2	161564616	G	0	9	A	0.0009641
rs112580488	2	161565442	G	0	9	A	0.0009641
rs111444533	2	161565445	G	0	9	A	0.0009641
rs79013319	2	161565611	A	0	9	T	0.0009641
rs62187573	2	162378064	A	1	12	G	0.0007887
rs73971596	2	169143262	A	2	14	C	0.0009649
rs377300030	2	169146356	T	2	14	TTTCAATTATTA	0.0009649
rs312922	2	172566634	T	14	31	C	0.0008795
2:195965687	2	195965687	C	15	35	G	0.0003321
2:200492176	2	200492176	G	24	4	GT	0.0001159
2:202876876	2	202876876	A	11	0	G	0.0007855
2:202876896	2	202876896	G	11	0	GAA	0.000738
2:203185279	2	203185279	C	23	41	T	0.0008021
rs2269079	2	207975211	C	16	2	T	0.0008956
rs1263628	2	207977132	C	16	2	G	0.0008956
2:220525299	2	220525299	G	35	12	GA	0.0004754
rs34083066	2	228958445	G	26	46	GTGCC	0.000277
rs141601528	2	231706695	G	0	9	A	0.0009641
rs201762374	2	233200229	G	24	5	C	0.0003659
rs6727222	2	234908786	T	40	16	G	0.0005221
rs6431650	2	234909387	G	40	16	A	0.0005221
rs4664000	2	234910002	C	40	16	G	0.0005221
rs6721761	2	234912661	T	41	17	G	0.0005984
rs6734618	2	234916831	A	40	16	G	0.0005221
rs6431651	2	234918215	C	40	15	T	0.0002535
rs6431652	2	234918391	G	38	15	C	0.0007851
rs4664001	2	234923881	T	39	15	C	0.0004501
rs11563200	2	234924809	G	20	41	A	0.0001748
rs2190951	2	234925075	T	39	15	C	0.0004501
rs2052029	2	234927688	C	39	15	G	0.0004501
rs10929331	2	234928493	G	20	40	A	0.0003188
rs1118259	2	234928612	A	39	15	G	0.0004501
rs35907217	2	234928973	T	39	15	TG	0.0004501
rs28948678	2	234928977	A	39	15	G	0.0004501
rs601129	2	234931053	T	39	15	C	0.0004501
rs604082	2	234931325	A	39	15	T	0.0004501
rs599294	2	234931434	A	20	39	G	0.0005694

rs56119740	2	234931598	C	20	39	CCT	0.0005694
rs597104	2	234931934	G	23	41	A	0.0008021
rs589763	2	234932165	T	20	39	C	0.0005694
rs585284	2	234932263	A	39	15	G	0.0004501
rs582082	2	234932954	T	49	24	G	0.000936
rs679207	2	234933090	T	20	39	C	0.0005694
rs663924	2	234934186	G	19	39	A	0.0002871
rs11679031	2	234934461	G	18	38	A	0.0002562
rs2176351	2	236394614	T	25	43	C	0.0008649
rs12471071	2	240995216	T	55	24	C	0.00002382
rs12471078	2	240995265	G	55	24	C	0.00002382
rs7565889	2	240997521	G	43	18	A	0.0003905
rs6437268	2	241000862	C	43	19	T	0.000761
rs10933567	2	241001540	G	43	18	C	0.0003905
rs7561353	2	241001882	T	43	18	A	0.0003905
rs57680690	2	241004813	A	43	18	G	0.0003905
rs12373696	2	241005516	T	43	18	C	0.0003905
rs56858681	2	241008514	T	43	18	TTA	0.0003905
rs1440011	2	241011768	A	43	18	G	0.0003905
rs35163917	2	241011913	T	43	18	TTAA	0.0003905
rs12477805	2	241016702	T	42	18	C	0.0006782
rs13026182	2	241016847	T	43	18	C	0.0003905
rs4555372	2	241020364	A	42	16	G	0.0002826
rs4854038	2	241023692	G	43	18	T	0.0003905
rs4854034	2	241028467	T	43	18	G	0.0003905
rs6725900	2	241029152	A	43	18	T	0.0003905
rs34204713	2	241033563	T	42	18	C	0.0006782
rs6756440	2	241040844	A	43	17	C	0.0001922
rs34427943	2	241041745	T	43	18	C	0.0003905
rs2369057	2	241043413	T	43	18	A	0.0003905
rs2019849	2	241048221	C	51	22	T	0.0000673
rs13422126	2	241048629	A	26	44	G	0.0008911
rs60296014	2	241048730	TC	26	44	T	0.0008911
rs4854030	2	241049147	A	26	45	G	0.0005028
rs4854029	2	241049148	A	26	45	G	0.0005028
rs4854028	2	241049442	A	26	44	C	0.0008911
rs6740952	2	241049626	A	26	45	G	0.0005028
rs10200500	2	241049977	C	26	45	T	0.0005028
rs10191379	2	241050051	G	26	45	T	0.0005028
2:241050226	2	241050226	TCCTCCTCCCCTCC	26	44	T	0.0004718
rs13414100	2	241050480	G	26	45	A	0.0005028

rs13424282	2	241050637	C	26	45 T	0.0005028
rs7572067	2	241051245	C	26	45 T	0.0005028
rs6721817	2	241051464	C	26	45 T	0.0005028
rs6731182	2	241051504	T	27	45 C	0.0009136
rs6722074	2	241051632	C	27	45 T	0.0009136
rs2083407	2	241054069	T	48	23 C	0.0008881
rs12467703	2	241054685	C	48	23 T	0.0008881
rs13411615	2	241075991	C	55	27 A	0.0002959
rs164462	3	8748648	A	45	20 G	0.0007326
rs164466	3	8749171	C	43	17 T	0.0001922
rs293786	3	9829226	A	34	11 C	0.0003893
rs2933304	3	11096859	G	30	49 T	0.0005383
rs55733119	3	11247589	T	13	30 G	0.0007578
rs11915050	3	11265233	G	11	28 A	0.0008103
rs11128544	3	11269122	A	11	28 G	0.0004277
rs6442240	3	11270027	A	11	29 G	0.0004498
rs6442241	3	11272005	G	10	27 A	0.0006386
rs73812409	3	11289265	G	10	27 A	0.0006386
rs34523650	3	11384407	A	9	25 AT	0.0008535
rs2442772	3	11493926	A	12	30 G	0.0005652
rs2454505	3	11497325	C	12	30 T	0.0005652
rs9820368	3	14247401	A	10	28 G	0.0006908
rs11709154	3	14582923	G	47	22 A	0.0008379
3:15506747	3	15506747	ATTTATTTATTTAT	12	0 A	0.0003752
3:20974098	3	20974098	A	21	40 C	0.0006238
rs12715025	3	22173653	C	39	14 T	0.0002137
rs2360615	3	24105183	G	5	24 T	0.00006606
rs111851541	3	24157398	A	12	28 AT	0.000945
rs150826285	3	26036390	A	0	10 C	0.0004292
rs140189968	3	26036393	G	0	10 C	0.0004292
rs76666691	3	26048549	T	0	10 C	0.0004292
rs75969402	3	26119136	A	0	10 G	0.0004292
rs138451079	3	26166331	T	0	9 C	0.0009641
rs13081103	3	29167341	A	0	9 G	0.0009641
3:30076122	3	30076122	A	11	0 AG	0.0008465
rs60554515	3	30076125	A	11	0 AAGGAAGGAAGG	0.0008465
rs202156754	3	33803658	TTTTC	5	22 T	0.0002305
3:34205883	3	34205883	C	0	9 T	0.0009641
rs200740771	3	38734879	C	0	11 A	0.0001895
rs35669757	3	38735351	GCC	0	11 G	0.0001895
rs7372391	3	38737643	C	1	13 T	0.0003716

rs62243860	3	38738157	C	0	11	T	0.0001895
rs62243861	3	38740297	A	1	12	G	0.0007887
rs7429946	3	38747522	G	0	11	A	0.0001895
rs6599244	3	38747735	G	0	11	A	0.0001895
rs6599246	3	38747810	C	1	13	G	0.0003716
rs11715513	3	38750383	A	0	11	G	0.0001895
rs35364139	3	40936831	T	16	2	C	0.0008956
rs17075284	3	43227494	C	3	16	T	0.0009912
rs71325010	3	43927399	G	20	40	C	0.0003188
rs979220	3	53556223	C	34	9	T	0.00004073
rs35555534	3	53562519	C	26	4	T	0.00002957
rs35306382	3	53579944	A	27	5	C	0.00005212
rs67495732	3	53587939	G	27	6	A	0.0001606
rs13100574	3	53596184	G	27	5	A	0.00005212
rs71074927	3	53596681	TC	27	5	T	0.0002419
rs2304577	3	53778621	A	5	19	G	0.0008415
rs11917063	3	59290258	C	24	42	A	0.0008352
rs6774839	3	60689647	C	39	16	T	0.0009032
rs67459165	3	61575592	T	41	17	C	0.0005984
rs73096794	3	61609045	A	41	17	G	0.0005984
rs4688645	3	61620896	T	42	17	C	0.0003422
rs4072198	3	62212067	A	29	48	G	0.0005332
rs875423	3	62246422	A	27	46	G	0.0005154
rs7651544	3	62265999	C	30	48	A	0.0009571
rs11312272	3	62304525	G	55	27	GT	0.0002959
rs1881269	3	62309701	A	55	27	G	0.0002959
rs10559149	3	63510527	G	35	13	GCA	0.000996
rs2202494	3	63521556	G	36	13	A	0.0005704
rs6445356	3	63523444	G	36	13	T	0.0005704
rs6786839	3	63523865	C	35	13	A	0.000996
rs7653698	3	63530406	T	36	13	A	0.0005704
rs34910545	3	65299195	C	15	1	T	0.0003561
3:75878600	3	75878600	G	14	1	GTA	0.0007094
3:82897861	3	82897861	T	9	26	TTCTTTC	0.0004896
rs34040734	3	102934988	T	0	9	A	0.0009641
rs35080343	3	102966610	A	0	11	G	0.0001895
rs75918042	3	102971010	A	1	12	C	0.0007887
3:102973408	3	102973408	G	0	10	A	0.0004292
rs75322311	3	102977885	C	0	9	T	0.0009641
rs34990881	3	102991912	A	1	12	C	0.0007887
3:103008027	3	103008027	A	0	11	G	0.0001895

rs186150184	3	103008029	A	0	11	G	0.0001895
3:103008030	3	103008030	TA	0	11	T	0.0001895
rs189281747	3	103008032	A	0	11	G	0.0001895
3:103008035	3	103008035	TTATATTTAATATT	0	11	T	0.0001895
3:103008036	3	103008036	T	0	11	G	0.0001895
3:103008038	3	103008038	A	0	11	G	0.0001895
3:103008042	3	103008042	T	0	11	C	0.0001895
3:103008045	3	103008045	ATATTTTATATTTT	0	11	A	0.0001895
rs78237109	3	103008122	G	0	9	A	0.0009641
rs74902008	3	103375964	G	0	9	C	0.0009641
rs201763621	3	109425840	T	34	12	A	0.000839
rs6804591	3	115401996	G	4	19	A	0.000262
rs6804592	3	115401998	G	3	19	A	0.0001307
rs78119965	3	126631298	T	0	9	C	0.0009641
3:135028478	3	135028478	TTAG	17	1	T	0.00007617
rs9852843	3	136938812	C	10	27	T	0.0006386
rs13324219	3	141037030	G	43	18	A	0.0003905
rs13065569	3	141077615	T	41	17	C	0.0005984
rs9857275	3	141078188	A	41	17	C	0.0005984
rs13100711	3	141082990	T	40	17	C	0.0009115
rs13098180	3	141091595	A	47	19	T	0.0001149
rs4683603	3	141092050	T	48	23	G	0.0008881
3:146472411	3	146472411	TTCTCTC	3	15	T	0.0008941
rs7615559	3	148544932	T	39	16	C	0.0009032
rs56745771	3	148545301	T	41	16	C	0.0002964
rs60122003	3	150675457	T	2	14	G	0.0009649
rs996838	3	150693325	C	30	48	G	0.0009571
rs34793251	3	150703180	TA	55	29	T	0.0009634
3:153129894	3	153129894	GTA	0	12	G	0.00008299
rs1907860	3	163546423	A	0	11	G	0.0001895
rs79902688	3	163549268	G	0	11	T	0.0001895
rs62293633	3	163557077	G	0	11	C	0.0001895
rs36022510	3	163557357	G	0	11	A	0.0001895
rs71156808	3	163562172	GT	0	11	G	0.0001895
rs62293636	3	163563579	C	0	11	T	0.0001895
rs12491031	3	163564559	C	0	11	T	0.0001895
rs13059483	3	163567068	A	0	11	C	0.0001895
rs13060209	3	163567299	C	0	11	G	0.0001895
3:163568079	3	163568079	AAT	0	11	A	0.0001895
rs12489971	3	163572245	A	0	11	C	0.0001895
rs2365589	3	163572825	G	0	11	T	0.0001895

rs141466002	3	163573679	A	0	11	G	0.0001895
rs34247696	3	163574006	T	0	11	G	0.0001895
rs71630481	3	163574522	C	0	11	CA	0.0001895
rs66817151	3	163574558	A	0	11	C	0.0001895
rs147941663	3	163575270	TTG	0	11	T	0.0001895
rs9839357	3	167595780	G	14	32	A	0.0004925
rs35997477	3	167595928	CT	13	31	C	0.0007049
3:173545424	3	173545424	TGTGTGTGTGTGT	0	20	T	0.00000008134
rs111562947	3	173824811	C	0	9	G	0.0009641
rs73173113	3	177081452	C	36	9	T	0.00001822
rs9861140	3	177085723	T	49	23	G	0.0005083
rs9816839	3	178288166	G	31	9	A	0.0002572
rs1477771	3	178289120	C	30	9	A	0.0004612
rs7652236	3	178291198	G	32	9	A	0.0001413
rs1382041	3	178291630	G	31	9	A	0.0002572
rs6443547	3	178291847	T	30	9	G	0.0004612
rs2863153	3	178294190	G	30	9	C	0.0004612
rs4857779	3	178298623	C	30	9	G	0.0004612
rs1477772	3	178300725	T	30	9	C	0.0004612
rs4857651	3	178302776	G	30	9	C	0.0004612
rs6443550	3	178304435	T	30	9	C	0.0004612
rs6809859	3	178316530	G	29	9	A	0.0008145
rs899077	3	178319839	C	29	9	T	0.0008145
rs1903811	3	178320664	A	42	17	G	0.0003422
rs931038	3	178320738	C	29	9	G	0.0008145
rs931039	3	178320739	G	42	17	A	0.0003422
rs1903812	3	178321446	T	42	17	C	0.0003422
rs1477774	3	178321949	G	42	17	A	0.0003422
rs1870979	3	178323513	T	42	17	G	0.0003422
rs3930234	3	184216130	T	19	3	C	0.0005082
rs11374679	3	184216166	T	22	4	TC	0.00043
rs4912570	3	184216323	G	27	5	C	0.00005212
rs11307534	3	184216381	CT	23	4	C	0.0002248
rs4912571	3	184216615	T	23	4	C	0.0002248
3:185821419	3	185821419	A	7	23	C	0.0004919
rs13060765	3	186701750	G	2	14	A	0.0009649
rs13060784	3	186701791	G	2	14	A	0.0009649
rs35846970	3	187170810	A	27	46	G	0.0005154
rs7627192	3	187171467	A	26	45	G	0.0005028
rs11721135	3	187172570	G	32	53	A	0.00009342
rs2378240	3	187173607	G	27	46	A	0.0005154

rs9815313	3	187175707	T	31	52	C	0.00009769
rs9832985	3	187175819	A	31	52	G	0.00009769
rs6444231	3	187177911	C	31	52	T	0.00009769
rs6444232	3	187177932	C	31	52	T	0.00009769
rs9820758	3	187180014	C	27	46	G	0.0005154
rs9863882	3	187181082	A	31	52	C	0.00009769
rs9826615	3	187181103	G	31	52	A	0.00009769
rs9837025	3	187183233	C	31	52	G	0.00009769
rs9874793	3	187183283	A	31	52	G	0.00009769
rs7637660	3	187183328	T	31	52	G	0.00009769
rs36000570	3	187184628	TA	31	52	T	0.00009769
rs11711933	3	187184904	T	31	52	G	0.00009769
rs11707872	3	187184976	C	31	52	A	0.00009769
rs11712026	3	187185161	T	32	54	C	0.00004651
rs11707979	3	187185176	G	32	54	C	0.00004651
rs11707964	3	187185301	C	31	52	T	0.00009769
rs145406405	3	187185380	T	50	25	G	0.0009811
rs149858946	3	187185465	C	30	52	A	0.00008196
rs13071669	3	187185696	G	49	22	C	0.0002673
rs13071540	3	187185714	G	47	21	A	0.000234
rs13071675	3	187185745	G	45	20	A	0.0003895
rs13071695	3	187185791	G	47	21	A	0.0001195
rs13076367	3	187186031	A	31	53	G	0.000049
rs13077076	3	187186132	G	31	52	A	0.00009769
rs13076909	3	187186148	C	31	52	T	0.00009769
rs13077380	3	187186244	A	49	24	G	0.000936
rs13076957	3	187186246	G	49	24	A	0.000936
3:187277304	3	187277304	T	32	10	C	0.0005752
rs35503915	3	188507075	TTTG	7	22	T	0.0009169
rs34785844	3	188507474	A	7	22	T	0.0009169
rs1606789	3	189045487	T	1	17	C	0.00001632
rs73889196	3	189050749	G	1	17	T	0.00001632
rs113495037	3	189051167	A	1	13	C	0.0003716
rs1399614	3	189067066	G	3	18	C	0.0002532
rs62289698	3	189067220	G	3	18	A	0.0002532
rs146602352	3	189068374	TCA	3	17	T	0.0002579
rs200458285	3	189690826	AC	10	27	A	0.0006898
rs710578	3	189741453	C	11	28	G	0.0008103
rs1403416	3	189767199	T	13	35	C	0.00003266
rs1403417	3	189767472	A	13	35	G	0.00003266
3:190280484	3	190280484	CAT	22	4	C	0.0004184

rs200759056	3	190280486	T	22	3	C	0.0001254
rs10804421	3	193721250	A	43	18	G	0.0003905
rs62288213	3	193723842	C	40	15	T	0.0002535
rs12487949	3	193726610	A	46	20	G	0.0004155
rs12487254	3	193727193	G	47	20	C	0.0002308
rs62288215	3	193727513	C	47	20	A	0.0002308
rs34144732	3	193727709	T	47	20	C	0.0002308
rs11715098	3	193727726	T	47	20	A	0.0002308
rs77882215	3	193727947	C	49	24	CAGG	0.000936
rs11706230	3	193728852	A	46	20	T	0.0004155
rs4687496	3	193729225	T	51	24	C	0.0003012
rs62288220	3	193729836	C	46	20	T	0.0004155
rs62288221	3	193730075	A	46	20	G	0.0004155
rs9870522	3	193730758	G	49	21	A	0.0001359
rs9875031	3	193731258	G	49	21	A	0.0001359
rs9875036	3	193731262	G	49	21	A	0.0001359
rs56182048	3	193731569	A	49	21	C	0.0001359
rs56060318	3	193731844	T	48	21	C	0.0002492
rs56223959	3	193731858	T	48	21	C	0.0002492
rs56079000	3	193732010	A	48	21	G	0.0002492
rs1078811	3	193732779	C	46	20	T	0.0004155
rs1078813	3	193732819	C	49	21	A	0.0001359
rs1078814	3	193733031	C	49	21	T	0.0001359
rs2279511	3	193733343	C	46	20	G	0.0004155
rs2279512	3	193733393	T	46	20	G	0.0004155
rs56150153	3	193733404	C	46	20	T	0.0004155
rs4607052	3	193733890	T	46	20	C	0.0004155
rs4610173	3	193734054	G	46	20	A	0.0004155
rs4607053	3	193734072	T	46	20	C	0.0004155
rs4607054	3	193734094	G	46	20	C	0.0004155
rs4632490	3	193734206	G	46	20	A	0.0004155
rs4600776	3	193734280	G	46	20	C	0.0004155
rs62288224	3	193734681	G	46	20	A	0.0004155
rs6773898	3	197067697	C	0	9	A	0.0009641
rs6776479	3	197067712	C	0	9	T	0.0009641
rs73212319	3	197068925	C	0	9	T	0.0009641
rs10428227	3	197566540	A	48	23	G	0.0008881
rs2306439	3	197574936	C	47	22	G	0.0008379
rs9325410	3	197580938	C	53	27	T	0.0006109
rs12718052	3	197594521	A	49	22	G	0.0002673
rs13144661	4	3945627	T	10	27	C	0.0006386

rs2980177	4	4171615	C	16	36	T	0.0001978
rs2980178	4	4171622	T	20	38	G	0.0009963
rs2916386	4	4171624	G	20	38	A	0.0009963
rs2980210	4	4174141	T	32	51	G	0.0003437
rs4689202	4	4175894	G	33	6	A	0.000002794
rs34485304	4	4175938	A	19	37	G	0.0004873
rs3097979	4	4201105	C	39	16	A	0.0009032
rs3097980	4	4201116	G	39	16	T	0.0009032
rs2916417	4	4202389	A	39	15	T	0.000769
rs2916422	4	4206953	A	23	44	G	0.0001337
rs7666381	4	4210460	C	22	44	G	0.00006674
rs34634401	4	4216238	C	17	35	T	0.0007327
rs34096660	4	4216258	C	17	36	T	0.0004114
rs13111344	4	4216578	T	17	36	C	0.0004114
rs13112949	4	4217277	T	17	36	C	0.0004114
rs13130968	4	4218502	C	18	36	A	0.0008188
rs13150671	4	5206154	T	37	14	G	0.0006739
rs62297683	4	5804878	G	8	26	A	0.0001919
rs62297684	4	5806712	T	7	22	G	0.0009169
rs62297707	4	5813512	C	6	22	T	0.0007374
rs10461053	4	6031632	G	15	33	T	0.0005689
rs17765384	4	6032235	A	11	29	G	0.0004498
rs7438838	4	6058732	T	11	29	C	0.0004498
rs17766335	4	6068267	T	13	30	C	0.0007578
rs11731133	4	6068495	T	13	32	C	0.0002287
rs28496717	4	8638873	G	17	35	A	0.0007327
rs28440053	4	8638924	C	17	35	T	0.0007327
4:9033194	4	9033194	ACG	34	5	A	0.0000002723
4:9033196	4	9033196	G	33	6	A	0.000002794
rs887925	4	13833161	C	0	9	T	0.0009641
rs142176733	4	16248336	G	22	4	T	0.00043
rs6448409	4	25846377	C	6	21	A	0.0006518
rs35758792	4	38961597	C	19	41	CT	0.00008368
4:40273934	4	40273934	T	0	9	TA	0.0009641
4:40273936	4	40273936	C	0	9	A	0.0009641
rs13104844	4	42500835	G	51	25	T	0.0005636
rs13148916	4	42507023	A	52	24	T	0.0001653
rs13139149	4	42510813	C	52	26	T	0.0005884
rs12650104	4	42511212	G	52	26	A	0.0005884
rs12651087	4	42512619	G	52	26	A	0.0005884
rs13353762	4	42519515	A	51	25	C	0.0005636

rs13143829	4	42523909	C	52	26	T	0.0005884
rs12649944	4	42527333	C	52	25	G	0.0003167
rs71648734	4	42528232	C	52	25	CCTT	0.0003167
rs5024243	4	42528369	C	52	25	A	0.0003167
rs35722201	4	42530697	A	52	25	G	0.0003167
rs4861033	4	42539281	C	52	25	T	0.0003167
rs62302266	4	42542341	A	52	25	G	0.0003167
rs10461060	4	42547450	G	52	25	T	0.0003167
rs12645297	4	42554363	C	50	25	T	0.0009811
rs4328946	4	42554984	T	50	25	G	0.0009811
rs10805104	4	42556540	T	50	25	C	0.0009811
rs13133445	4	42556776	T	50	25	C	0.0009811
rs13108456	4	42556881	G	50	25	A	0.0009811
rs13119526	4	42557045	C	50	25	T	0.0009811
rs7666279	4	42557555	C	50	25	A	0.0009811
rs7671271	4	42557627	G	50	25	T	0.0009811
rs7666734	4	42557817	G	50	25	A	0.0009811
rs114935572	4	43378946	C	0	9	A	0.0009641
rs76788362	4	49185593	A	12	0	C	0.0003752
4:49316327	4	49316327	G	11	0	C	0.0007855
4:49630742	4	49630742	T	14	1	C	0.0007094
rs2616405	4	54589232	T	52	26	A	0.0005884
rs2590827	4	54589337	G	52	26	A	0.0005884
rs2590829	4	54590029	G	32	50	C	0.0009655
rs9759545	4	54606083	C	52	26	A	0.0005884
rs2590763	4	54613453	C	50	23	T	0.0002847
rs2616434	4	54616649	G	52	22	A	0.00004088
rs2616433	4	54617199	A	50	22	C	0.0001461
rs5858236	4	54617655	C	49	21	CA	0.0001359
rs2616431	4	54618315	C	51	22	G	0.00007816
rs2412448	4	54620401	A	45	20	C	0.0007326
rs2590807	4	54621567	A	52	22	T	0.00004088
rs6826915	4	54622017	A	52	22	G	0.00004088
rs6815433	4	54622219	C	40	16	T	0.0005221
rs59481386	4	54625401	G	45	20	T	0.0007326
rs6842780	4	54626228	C	45	20	T	0.0007326
rs7655896	4	54626897	A	45	20	G	0.0007326
rs6828755	4	54637552	G	50	22	A	0.0001461
rs4292386	4	54642592	T	49	23	C	0.0005083
rs5858241	4	54644872	AT	50	22	A	0.0001461
rs7694601	4	54645684	C	50	22	T	0.0001461

rs4864488	4	54646237	C	50	22	A	0.0001461
rs4864820	4	54646492	T	50	22	C	0.0001461
rs1498830	4	54647761	G	50	22	A	0.0001461
rs904414	4	54648902	G	52	26	C	0.0005884
rs7378471	4	54652137	G	48	21	A	0.0002492
rs2088582	4	54653803	C	48	21	T	0.0002492
rs1718824	4	57903747	A	11	32	C	0.00006919
rs1277313	4	57903948	T	11	32	G	0.00006919
rs6839014	4	57911929	G	14	32	A	0.0004925
rs12642538	4	57914318	T	14	32	C	0.0004925
rs13145672	4	57917126	C	14	31	G	0.0008795
4:68012780	4	68012780	C	34	10	CAG	0.0001587
rs1220858	4	70805831	T	42	18	A	0.0006782
4:82709303	4	82709303	G	13	0	GTATATATACA	0.0001973
4:84610390	4	84610390	T	37	14	TG	0.0006739
rs34736493	4	89746367	A	50	23	AT	0.000426
rs1513815	4	90099668	C	16	2	A	0.0008956
rs34952537	4	90112103	G	17	2	A	0.000467
rs71609558	4	90267765	A	0	10	G	0.0004292
rs4441725	4	94082392	C	43	19	A	0.000761
rs1393804	4	94084099	C	43	19	A	0.000761
rs144710773	4	94084638	G	43	19	C	0.0006329
rs1371358	4	94585894	C	15	33	A	0.0005689
4:99749755	4	99749755	G	4	19	GA	0.0004818
rs3804357	4	102002123	A	16	34	C	0.0006492
rs17030740	4	102006277	C	16	34	T	0.0006492
rs62329749	4	105811657	T	16	2	C	0.0008956
rs62329754	4	105827355	A	16	2	G	0.0008956
4:109041155	4	109041155	T	10	32	G	0.00002619
rs1126483	4	111409705	T	22	46	C	0.00001707
rs6833731	4	111410482	A	12	31	G	0.0001903
rs12503640	4	111412833	T	22	45	A	0.00003418
rs2348427	4	111414399	T	22	45	C	0.00003418
rs2881913	4	111416282	A	22	45	C	0.00003418
rs2348428	4	111418528	A	22	45	T	0.00003418
rs2348429	4	111418774	G	22	45	A	0.00003418
rs17551888	4	111419056	T	22	45	C	0.00003418
rs5861009	4	111419509	C	22	45	CA	0.00003418
rs6842486	4	111420283	T	22	46	A	0.00001707
rs11938659	4	111423314	A	12	31	G	0.0001903
rs2348430	4	111424096	G	22	45	A	0.00003418

rs6821205	4	111424198	C	12	31 T	0.0001903
rs9992936	4	111424307	A	22	45 G	0.00003418
rs10015807	4	111424391	G	22	45 C	0.00003418
rs12506732	4	111424431	G	22	46 A	0.00001707
rs6844746	4	111424598	G	12	31 A	0.0001903
rs2348431	4	111424767	C	22	45 A	0.00003418
rs11731078	4	111426341	C	12	31 T	0.0001903
rs9998275	4	111431182	G	12	31 A	0.0001903
rs6813802	4	111432041	C	22	45 T	0.00003418
rs10024396	4	111432708	G	12	31 C	0.0001903
rs10024670	4	111432841	A	12	31 G	0.0001903
rs10024679	4	111432854	A	12	31 G	0.0001903
rs10027031	4	111433076	T	12	31 C	0.0001903
rs13134674	4	111433217	A	12	31 G	0.0001903
rs3796889	4	111435409	T	22	45 A	0.00003418
rs3796888	4	111435434	T	22	45 C	0.00003418
rs11406861	4	111435856	T	22	45 TG	0.00003418
rs2348433	4	111437127	A	22	45 C	0.00003418
rs1996604	4	111438359	G	22	45 A	0.00003418
rs11735606	4	111439018	G	12	31 C	0.0001903
rs7669705	4	111439486	T	12	31 G	0.0001903
rs1448808	4	111445587	A	23	48 G	0.000008737
rs9993086	4	111447259	G	14	32 C	0.0004925
rs639194	4	111450323	C	14	33 T	0.0002703
rs522426	4	111450537	C	14	33 T	0.0002703
rs6823156	4	111451028	A	14	33 G	0.0002703
rs35782762	4	111451075	C	15	33 CA	0.0005689
rs55704506	4	111451237	G	14	33 GA	0.0004679
rs1375298	4	111453751	A	24	48 G	0.0000187
rs28447570	4	111456923	A	13	31 G	0.0004205
rs12331296	4	113255293	G	16	35 A	0.000362
rs13144272	4	113309794	C	23	44 A	0.0006722
4:114222536	4	114222536	T	5	24 C	0.00002717
rs58515333	4	114242449	C	1	13 CAT	0.0003716
rs77771853	4	118223667	C	16	2 T	0.0008956
rs115597384	4	118224725	G	16	2 T	0.0008956
rs72914164	4	118238440	G	16	2 A	0.0008956
rs143741879	4	118241384	AC	16	2 A	0.0008956
rs72914172	4	118244899	A	16	2 G	0.0008956
rs17861758	4	118254457	A	16	2 G	0.0008956
rs80129590	4	118255352	T	16	2 C	0.0008956

rs144775364	4	118259719	CT	16	2	C	0.0008956
rs6820085	4	118259780	A	16	2	G	0.0008956
rs6824651	4	118259951	G	16	2	C	0.0008956
rs148569473	4	118264325	C	16	2	CA	0.0008956
rs72344381	4	118287512	T	16	2	TTCTC	0.0008956
rs17861809	4	118292890	T	16	2	C	0.0008956
rs17861813	4	118294536	C	16	2	G	0.0008956
rs17861840	4	118310450	T	16	2	G	0.0008956
rs62313510	4	118313326	A	16	2	G	0.0008956
rs13132223	4	118316992	C	16	2	G	0.0008956
rs17861874	4	118328706	G	16	2	A	0.0008956
rs150600712	4	118332190	AT	16	2	A	0.0008956
rs13114404	4	118334362	G	16	2	A	0.0008956
rs17861904	4	118354621	C	16	2	T	0.0008956
4:118438369	4	118438369	C	12	0	T	0.0004093
rs58125305	4	120045373	GA	10	27	G	0.0006386
rs7695544	4	120741242	A	30	9	G	0.0004612
rs75842627	4	120745179	T	30	9	TG	0.0004612
rs35367572	4	120747061	A	29	9	G	0.0008145
rs34269004	4	120747293	C	29	9	G	0.0008145
rs12509043	4	120747574	T	29	9	C	0.0008145
rs11098543	4	120748542	G	32	9	A	0.0001413
rs11737790	4	120751664	T	32	9	C	0.0001413
rs4834817	4	120752566	C	33	9	T	0.00007642
rs6534157	4	120753056	G	32	9	C	0.0001413
rs6858155	4	120754216	T	32	9	G	0.0001413
rs72682879	4	120754392	G	32	9	T	0.0001413
rs2389919	4	120756095	G	32	9	A	0.0001413
rs17363983	4	120756608	G	32	9	T	0.0001413
rs6856904	4	120759579	A	29	9	G	0.0008145
rs6822154	4	120761791	A	30	9	C	0.0004612
rs6822164	4	120761806	G	30	9	C	0.0004612
rs62319692	4	120763615	T	29	8	G	0.0003391
rs12374391	4	120766947	G	29	9	A	0.0008145
rs1587736	4	120772766	T	30	9	G	0.0004612
rs1910423	4	120777023	C	32	9	T	0.0001413
rs34463615	4	120784702	G	32	9	C	0.0001413
rs34021877	4	120784708	C	32	9	T	0.0001413
rs35229169	4	120787593	C	32	9	A	0.0001413
rs1818167	4	120790884	C	29	9	T	0.0008145
rs4254800	4	122378732	G	7	23	A	0.0004919

rs376830774	4	124487236	A	40	16	ATCTG	0.0005221
rs1872296	4	143231657	A	2	14	T	0.0009649
rs35157240	4	143233592	A	2	14	G	0.0009649
rs35298298	4	143234244	T	2	14	G	0.0009649
rs10013303	4	143234542	G	2	14	C	0.0009649
rs10034842	4	143234609	C	2	14	G	0.0009649
rs10024947	4	143234663	G	2	14	A	0.0009649
rs10025044	4	143234754	G	2	14	A	0.0009649
rs16998530	4	143235258	A	2	14	G	0.0009649
rs3756121	4	143235344	A	2	14	G	0.0009649
rs11945996	4	143235721	C	2	15	T	0.0004755
rs6811427	4	143236750	T	2	14	C	0.0009649
rs6858661	4	143236897	A	2	14	G	0.0009649
rs6812034	4	143237043	T	2	14	C	0.0009649
rs6816219	4	143237530	C	2	14	G	0.0009649
rs6817826	4	143237539	T	2	14	C	0.0009649
rs6847957	4	143237568	G	2	14	T	0.0009649
rs62328283	4	143237903	A	2	14	G	0.0009649
rs6850050	4	143238764	G	2	15	A	0.0004755
rs9654253	4	143239277	T	2	15	A	0.0004755
rs17015920	4	143240651	C	2	15	T	0.0004755
rs62328284	4	143241936	G	2	15	A	0.0004755
rs62328285	4	143242074	C	2	15	T	0.0004755
rs10519642	4	143243150	G	2	14	A	0.0009649
rs200309497	4	147248072	AGT	12	33	A	0.00005204
rs147593266	4	153449012	TTA	5	21	T	0.0002243
rs6535901	4	154093408	C	31	50	T	0.0005409
rs6820726	4	154093464	T	31	51	C	0.0002959
rs10023596	4	154094001	G	32	51	A	0.0003437
rs11099874	4	154094107	C	31	51	G	0.0002959
rs11099875	4	154094109	C	31	51	G	0.0002959
rs11099876	4	154094235	G	31	51	C	0.0002959
rs10002011	4	154094675	A	30	51	G	0.0001576
rs10001881	4	154094682	T	30	51	C	0.0001576
rs7671132	4	154094793	T	31	51	C	0.0002959
rs7696186	4	154095073	C	31	51	T	0.0002959
rs142792673	4	154096164	GTTTTGT	52	25	G	0.00007927
rs75572337	4	156985219	G	1	12	A	0.0007887
rs75082465	4	162686966	T	0	10	G	0.0004197
rs200385614	4	164804411	A	9	31	G	0.00001851
rs200908737	4	164804412	T	9	31	A	0.00001851

4:164896322	4	164896322	T	49	21	G	0.000236
rs1246928	4	166543688	G	19	3	T	0.0005082
rs1246927	4	166543852	C	39	12	T	0.00004106
rs56327210	4	166716974	T	47	22	C	0.0008379
rs3046194	4	166721228	ATAATAT	47	22	A	0.0008379
rs7683920	4	166750173	C	40	16	T	0.0005221
rs7654317	4	166750528	A	40	16	T	0.0005221
rs1460065	4	166750991	A	40	16	G	0.0005221
rs78076428	4	169546661	G	20	3	T	0.0005154
rs57853587	4	170291277	CT	23	5	C	0.0007245
rs112092862	4	175405570	A	0	9	G	0.0009641
rs112529026	4	175405838	C	0	9	CAGG	0.0009641
rs13145311	4	187022823	C	52	25	T	0.0003167
rs10564187	4	187022827	T	6	21	TA	0.0006518
4:187284255	4	187284255	A	0	9	C	0.0009641
rs72629660	4	187687317	A	1	12	G	0.0007887
rs79858754	4	189972171	A	1	12	G	0.0007119
rs55809797	4	189995776	A	6	21	G	0.0006518
rs74799204	4	190024436	A	1	15	C	0.00007965
rs1566102	4	190025016	G	17	35	T	0.0007327
4:190913846	4	190913846	T	11	0	C	0.0007855
rs35028140	5	2077343	G	25	43	GAA	0.0008649
rs11133927	5	2077356	T	25	43	G	0.0008649
rs10866530	5	2077580	T	25	43	C	0.0008649
rs10866531	5	2077739	T	25	43	G	0.0008649
rs465950	5	2354762	C	18	3	T	0.0009486
rs460672	5	2355032	G	17	2	C	0.000467
rs462906	5	2355389	T	16	2	C	0.0008956
rs113602711	5	2355658	T	16	2	TATGCTAATGTCA	0.0008956
rs58937979	5	2355682	T	16	2	C	0.0008956
rs2641215	5	2355878	T	16	2	G	0.0008956
rs456732	5	2358645	G	16	2	A	0.0008956
rs7356684	5	2661836	A	26	5	C	0.0001012
rs6896765	5	3424724	T	6	21	C	0.0006518
rs13186531	5	3660262	A	19	1	G	0.00001507
rs13187667	5	3660269	T	16	1	C	0.0001772
rs67486607	5	3895075	G	0	9	A	0.0009641
rs12653733	5	5433611	T	24	43	C	0.000471
rs78139414	5	18375453	A	2	14	G	0.0009649
rs200432192	5	19163455	T	21	4	C	0.0008104
rs192918603	5	19166903	G	29	9	T	0.0008145

rs10941087	5	19171347	A	29	9	G	0.0008145
rs4546383	5	19174117	T	29	9	G	0.0008145
rs56127401	5	19175816	G	29	9	A	0.0008145
rs11954805	5	19179319	C	29	9	T	0.0008145
rs67595693	5	19181373	C	29	9	T	0.0008145
rs56010419	5	19181660	C	29	9	T	0.0008145
rs28753870	5	19183204	G	29	9	T	0.0008145
rs10461927	5	19183422	C	29	9	T	0.0008145
rs67749574	5	19184078	C	29	9	T	0.0008145
rs4492113	5	19185179	C	29	9	A	0.0008145
rs10461707	5	19185427	T	29	9	A	0.0008145
rs10055960	5	19185900	A	29	9	G	0.0008145
rs10056045	5	19186087	C	29	9	G	0.0008145
rs35630947	5	19186390	G	29	9	GA	0.0008145
rs4409093	5	19186620	T	29	9	C	0.0008145
rs10073850	5	19187322	T	29	9	C	0.0008145
rs143565175	5	19187627	G	30	9	C	0.0004612
rs1512548	5	19399602	T	21	4	C	0.0008104
rs5866397	5	19742516	TAC	27	45	T	0.0009136
rs10068995	5	19800575	C	28	46	T	0.0009321
rs5024079	5	19808438	A	24	43	C	0.000471
rs34820291	5	19837916	CA	29	47	C	0.0009467
rs6451918	5	20909303	G	27	46	T	0.0005154
rs184303633	5	21357168	C	11	0	G	0.000738
rs2174436	5	31920019	A	3	17	G	0.0005045
5:35548420	5	35548420	C	20	41	T	0.0001748
rs10941321	5	37213350	C	31	10	A	0.0006062
rs76348379	5	37901402	T	13	0	C	0.0001783
rs76564102	5	37903568	A	12	0	C	0.0003752
rs2973080	5	37906865	G	39	15	A	0.0004501
rs6874732	5	38105437	C	1	12	T	0.0007887
rs7715558	5	38105838	C	1	12	T	0.0007887
rs11346399	5	49624292	G	29	9	GA	0.0008145
rs7702276	5	51805742	T	17	35	C	0.0007327
rs75510999	5	52940660	A	0	9	G	0.0009641
rs116611286	5	53040709	G	0	9	A	0.0009641
rs6878995	5	54976056	T	0	10	G	0.0004292
5:57402182	5	57402182	A	10	32	G	0.00002619
rs140681291	5	58464504	A	1	12	AAGAG	0.0007887
5:58496527	5	58496527	A	0	9	ATAGATACACGTA	0.000946
rs147324646	5	59812601	AT	22	4	A	0.00043

rs35856431	5	59815431	GA	21	4	G	0.0008104
rs10075757	5	59815534	C	21	4	T	0.0008104
rs10065640	5	59821598	G	21	4	A	0.0008104
rs7727343	5	59823198	C	21	4	T	0.0008104
rs7712117	5	59823870	C	21	4	A	0.0008104
rs112450883	5	59826635	C	21	4	T	0.0008104
rs3857235	5	59830539	G	21	4	A	0.0008104
rs6891919	5	59832376	C	21	4	A	0.0008104
rs6898521	5	59833943	C	21	4	A	0.0008104
rs6449475	5	59834261	T	21	4	C	0.0008104
rs7724442	5	59834603	A	21	4	T	0.0008104
rs9291692	5	59835549	G	21	4	A	0.0008104
rs6870200	5	61405556	C	29	9	T	0.0008145
rs6449583	5	61408033	C	27	6	A	0.0001606
rs10471534	5	61414874	A	27	6	G	0.0001606
rs35311635	5	61415810	A	27	6	C	0.0001606
rs7721209	5	61416428	A	27	6	G	0.0001606
rs3899770	5	61416926	A	27	6	G	0.0001606
rs9291748	5	61418667	T	20	1	C	0.000009945
rs7719350	5	61419458	G	27	6	T	0.0001606
rs13356167	5	61420029	C	27	6	T	0.0001606
rs7704609	5	61420501	G	27	6	A	0.0001606
rs1363785	5	61428613	C	27	6	T	0.0001606
rs7722122	5	61439524	T	27	5	G	0.00005212
rs71631929	5	61446349	T	20	1	C	0.000009945
rs201535035	5	61451674	G	18	1	GA	0.00004271
rs376091358	5	61451676	T	18	1	G	0.00004271
rs1833800	5	61453396	A	28	6	G	0.00008463
rs6876952	5	61459698	C	27	6	T	0.0001606
rs6878813	5	61463949	T	20	1	C	0.000009945
rs401016	5	67316364	C	11	28	A	0.0008103
rs62357186	5	73432081	T	23	5	G	0.0006807
rs12187319	5	73433727	G	24	5	A	0.0003659
rs11744466	5	73438529	A	24	5	G	0.0003659
rs11738090	5	73438540	T	24	5	C	0.0003659
rs9293620	5	73441891	G	33	11	C	0.0006951
rs12188714	5	73450440	G	24	4	A	0.0001159
rs62357188	5	73454385	C	17	1	T	0.00008737
rs138284024	5	73454807	AT	17	1	A	0.00008737
5:73455588	5	73455588	G	16	1	GAGAGAGAGAGA	0.0001772
rs62357189	5	73455804	A	17	1	G	0.00008737

rs72776364	5	73456113	C	24	4	G	0.0001159
rs62357204	5	73457335	A	17	1	G	0.00008737
rs62357205	5	73458233	T	17	1	C	0.00008737
rs62357206	5	73458276	T	17	1	A	0.00008737
rs62357207	5	73458728	C	17	1	T	0.00008737
rs975949	5	73460035	T	17	1	C	0.00008737
rs76802708	5	73460555	G	17	1	A	0.00008737
rs76299023	5	73460579	T	17	1	A	0.00008737
rs77089651	5	73460581	T	17	1	C	0.00008737
rs17637176	5	73460633	A	17	1	T	0.00008737
rs62357208	5	73464335	T	24	4	C	0.0001159
rs72776367	5	73467836	T	24	4	C	0.0001159
5:75248077	5	75248077	TTCCG	0	12	T	0.00006904
rs569523	5	78024366	T	17	36	C	0.0004114
rs2055432	5	78988769	A	27	47	C	0.0002837
rs4704574	5	78989327	C	28	47	T	0.0005255
rs4704575	5	78989365	G	28	47	A	0.0005255
rs4704576	5	78989486	C	28	46	T	0.0009321
rs7702610	5	78990914	C	27	46	A	0.0005154
rs12514427	5	78992606	G	28	46	A	0.0009321
rs4704577	5	79006389	T	25	43	C	0.0008649
rs201096991	5	83266476	G	0	10	A	0.0004292
rs10063186	5	85052886	T	24	42	C	0.0008352
rs10067814	5	85055484	T	24	42	C	0.0008352
rs3909944	5	85055983	G	24	42	T	0.0008352
rs62363128	5	85057573	C	5	19	T	0.0008415
5:85063367	5	85063367	CTGGAGTGCAG	5	19	C	0.0008415
rs62364079	5	85097891	A	24	44	T	0.0002594
rs10078974	5	85101296	T	24	42	G	0.0008352
rs6860643	5	85102038	T	23	42	G	0.0004519
rs6879925	5	85102220	A	23	42	G	0.0004519
5:85102370	5	85102370	C	23	42	CT	0.0004519
rs1039324	5	85102498	G	23	42	A	0.0004519
rs4304102	5	85108834	T	23	42	A	0.0004519
rs10942404	5	85109492	C	22	42	T	0.0002369
rs6895196	5	85111641	A	7	22	T	0.0009169
rs2173679	5	85111915	A	22	42	G	0.0002369
rs6877523	5	85115583	C	7	22	T	0.0009169
rs6888878	5	85117627	T	22	42	C	0.0002369
rs6868211	5	85117836	G	22	42	A	0.0002369
rs12652897	5	85123006	C	22	42	G	0.0002369

rs57485661	5	85124421	A	7	22	G	0.0009169
rs10071957	5	85124453	A	22	42	G	0.0002369
rs10942407	5	85126036	A	22	42	C	0.0002369
rs10069169	5	85126181	T	22	42	C	0.0002369
rs10474196	5	85127397	A	22	42	G	0.0002369
rs71607777	5	85129576	T	22	43	TACAAAA	0.0001272
rs6881850	5	85130765	A	7	22	G	0.0009169
rs11742380	5	85131478	A	7	22	C	0.0009169
rs2133745	5	85136248	G	23	42	T	0.0004519
rs34068512	5	85140278	GT	23	42	G	0.0004519
rs144463603	5	85173367	A	0	10	C	0.0004292
rs72781968	5	85186743	T	0	10	C	0.0004292
rs149527180	5	85205233	C	0	10	T	0.0004292
rs72783908	5	85232631	G	0	10	T	0.0004292
rs147518381	5	86338865	G	5	19	A	0.0008415
rs10514311	5	89027113	T	6	21	C	0.0006518
rs6893436	5	89040596	A	6	21	T	0.0006518
rs10074028	5	89041960	A	5	20	G	0.0004383
rs6868869	5	89042883	A	5	20	T	0.0004383
rs13182917	5	89052566	A	5	20	T	0.0004383
rs6452945	5	90499159	A	19	3	T	0.0005082
rs2941885	5	90505517	C	28	8	A	0.0006092
5:91200681	5	91200681	A	45	16	T	0.00002569
5:91200683	5	91200683	A	45	16	T	0.00002569
rs116698885	5	102950733	G	14	1	A	0.0007094
rs113569962	5	105957055	G	34	11	T	0.0003893
rs10477869	5	105959382	C	40	14	A	0.0001172
rs202030174	5	105960773	C	40	13	A	0.0000514
rs74957701	5	105960906	C	40	13	G	0.0000514
rs140479842	5	105961778	G	35	12	A	0.0004754
rs200882810	5	105962269	C	35	11	G	0.0002142
rs190734124	5	105962622	A	40	13	C	0.0000514
rs183331089	5	105962624	G	40	13	C	0.0000514
rs6873840	5	105965170	G	40	13	A	0.0000514
rs9327968	5	105968183	C	35	12	A	0.0004754
rs10059626	5	105969636	A	35	12	T	0.0005157
rs138302217	5	105974033	T	39	13	C	0.00009633
rs202038596	5	105974512	G	35	12	A	0.0004754
rs58151039	5	105975178	C	40	13	A	0.0000514
rs34925549	5	105975284	G	35	12	C	0.0004754
rs5870212	5	105976556	G	40	13	GA	0.0000514

rs4616879	5	105982997	C	40	13	T	0.0000514
rs9327970	5	105985135	G	40	13	A	0.0000514
rs1158614	5	105985695	C	39	13	T	0.00009633
rs966225	5	105986040	T	40	13	A	0.0000514
rs7721522	5	105989095	G	35	12	A	0.0004754
rs1370474	5	105990288	C	39	13	T	0.00009633
rs11242599	5	105992483	A	34	12	C	0.000839
rs6891375	5	105996058	C	33	11	A	0.0006951
rs1370475	5	105996725	G	33	11	A	0.0006951
rs10079437	5	106000391	G	33	11	A	0.0006951
rs10053634	5	106002360	G	33	11	A	0.0006951
rs1437636	5	106016950	C	33	11	T	0.0006951
rs143398868	5	106018150	C	33	11	CAT	0.0006951
rs770165	5	107046018	T	24	5	G	0.0003951
rs78207211	5	113161697	A	11	0	G	0.0007855
rs12656434	5	113218006	A	50	25	C	0.0009811
rs140935191	5	113218057	G	2	17	T	0.00012
5:113218065	5	113218065	G	2	14	T	0.0009649
rs12186566	5	113218242	G	50	25	A	0.0009811
rs4895328	5	120967641	A	7	23	G	0.0004919
rs5870967	5	120968708	CT	9	25	C	0.0008942
rs6869549	5	120969061	C	9	25	T	0.0008942
rs9327207	5	120969316	C	9	25	A	0.0008942
rs7711526	5	120969689	G	9	25	A	0.0008942
rs7731440	5	120969849	T	9	25	A	0.0008942
rs4370308	5	120983112	C	9	25	T	0.0008942
rs337092	5	122558368	C	38	15	T	0.0007851
rs79167211	5	122560124	T	16	1	C	0.0001772
rs17405383	5	122560170	G	16	1	A	0.0001772
rs150027894	5	122566007	T	16	1	G	0.0001772
rs13172417	5	122566448	A	16	1	G	0.0001772
rs34280645	5	122568587	C	16	1	A	0.0001772
rs74414053	5	122569810	C	16	1	A	0.0001772
rs76138637	5	122569848	G	16	1	C	0.0001772
5:122573470	5	122573470	C	15	1	T	0.0003561
5:122573576	5	122573576	T	16	1	G	0.0001772
5:122573578	5	122573578	C	16	1	T	0.0001772
5:122573586	5	122573586	G	16	1	C	0.0001772
5:122573736	5	122573736	C	16	1	T	0.0001772
5:122573742	5	122573742	A	16	1	G	0.0001772
5:122573780	5	122573780	T	15	1	C	0.0003561

5:122575121	5	122575121	C	16	1	G	0.0001772
5:122576433	5	122576433	A	16	1	G	0.0001772
rs79781130	5	122577509	G	16	1	T	0.0001772
rs71594339	5	122579587	G	16	1	A	0.0001772
rs35608317	5	122581688	A	16	1	G	0.0001772
rs17405867	5	122581941	G	16	1	A	0.0001772
rs13164755	5	122584429	A	16	1	G	0.0001772
rs145981334	5	122587801	C	16	1	G	0.0001772
5:122589049	5	122589049	A	16	1	G	0.0001772
5:122589050	5	122589050	A	16	1	G	0.0001772
rs6884047	5	122597982	G	14	1	A	0.0006479
rs79445288	5	122600576	T	16	1	A	0.0001772
rs111502771	5	122601754	C	16	1	A	0.0001772
rs79398678	5	122601871	G	16	1	T	0.0001772
rs6881525	5	122606476	C	12	0	T	0.0003752
5:124925463	5	124925463	T	16	2	TAG	0.0008956
rs2162702	5	125525211	C	34	12	A	0.000839
rs7718533	5	125533158	T	31	10	G	0.0006062
rs13359614	5	125550942	C	31	10	T	0.0006062
rs10069560	5	132499786	C	6	24	G	0.00008935
rs1428142	5	132500250	A	6	21	G	0.0005124
rs1346488	5	132500707	G	6	24	C	0.00008935
rs10479029	5	132501489	A	6	23	G	0.0001763
rs71221401	5	132501948	GT	6	23	G	0.0001763
rs6596105	5	132502510	T	6	24	C	0.00008935
rs10075650	5	132502643	T	6	24	G	0.00008935
rs6596106	5	132502706	C	6	24	T	0.00008935
rs34716434	5	132503050	A	6	24	AT	0.00008935
rs2864674	5	132503211	G	6	24	A	0.00008935
rs2864675	5	132503305	G	6	24	T	0.00008935
rs139044359	5	132503863	AT	6	24	A	0.00008935
rs6876153	5	132503945	G	8	25	C	0.0003633
rs148563958	5	132504805	C	5	24	G	0.00002717
rs10078326	5	132505281	C	6	24	T	0.00008935
rs4958098	5	132505795	G	6	24	A	0.00008935
rs2081876	5	132506338	C	6	24	G	0.00008935
rs2864676	5	132506685	C	6	24	T	0.00008935
rs2864677	5	132506913	T	6	24	C	0.00008935
rs2864678	5	132507175	C	6	24	A	0.00008935
rs1428149	5	132510176	G	6	24	A	0.00008935
rs2195308	5	132510855	T	6	24	C	0.00008935

rs1896634	5	132511553	C	6	24	T	0.00008935
rs6895827	5	132512049	G	6	23	C	0.0001763
rs10065529	5	132513293	G	6	23	T	0.0001763
rs141838651	5	132516619	C	6	23	T	0.0001763
rs187907350	5	132516659	G	6	23	A	0.0001763
rs7445282	5	132517003	G	6	23	T	0.0001763
rs4605841	5	132517114	A	6	23	G	0.0001763
rs10479033	5	132517892	T	6	23	C	0.0001763
rs10479034	5	132518301	G	6	23	C	0.0001763
rs199797087	5	132519701	C	6	23	A	0.0001763
rs200466766	5	132520095	T	6	23	TC	0.0001763
rs28721275	5	132521908	C	6	23	T	0.0001763
rs28711097	5	132522108	A	6	22	C	0.000342
rs28615392	5	132522121	C	6	22	G	0.000342
rs10068084	5	132523892	T	6	23	G	0.0001763
rs11744199	5	132527098	A	6	23	G	0.0001763
rs201464745	5	132528130	C	6	23	CCAT	0.0001763
rs67782869	5	132529906	A	6	23	G	0.0001763
rs61168871	5	132531840	T	6	24	A	0.00008935
rs57352224	5	132532002	A	6	24	G	0.00008935
rs61671272	5	132532128	G	6	24	GA	0.00008935
rs3749820	5	132533063	T	6	24	A	0.00008935
rs13183672	5	132533517	C	6	24	A	0.00008935
rs3798103	5	132534059	G	6	24	T	0.00008935
rs3749817	5	132535046	A	6	24	G	0.00008935
rs3749816	5	132535659	G	16	34	A	0.0006492
rs71581359	5	132538068	G	7	24	GAAGT	0.0002593
rs4533941	5	132539483	C	7	24	T	0.0002593
rs4441915	5	132539522	T	7	24	G	0.0002593
rs67266593	5	132540436	A	7	24	ATTGT	0.0002593
rs9327646	5	132540707	A	7	24	G	0.0002593
rs9327647	5	132541007	A	7	24	G	0.0002593
rs9327648	5	132541121	T	7	24	C	0.0002593
rs141063508	5	132541475	T	7	24	TAATC	0.0002593
rs5871476	5	132541874	CA	7	24	C	0.0002593
rs67490161	5	132542052	G	7	24	GTTAT	0.0002593
rs4958168	5	132543001	G	9	25	A	0.0008942
rs13175863	5	134579640	T	0	9	C	0.0009641
rs429946	5	141183920	T	1	12	C	0.0007887
rs420150	5	141184008	A	1	12	C	0.0007887
rs404942	5	141184120	T	1	12	C	0.0007887

rs430963	5	141184633	T	1	12	C	0.0007887
rs457518	5	141185061	A	1	12	G	0.0007887
rs456653	5	141185473	A	1	12	C	0.0007887
rs2974707	5	141187233	G	1	12	A	0.0007887
rs248470	5	141188537	G	1	12	C	0.0007887
rs447869	5	141188750	A	1	12	C	0.0007887
rs248472	5	141189541	T	1	12	C	0.0007887
rs173570	5	141198031	G	0	10	A	0.0004292
rs171584	5	141201531	C	0	10	G	0.0004292
rs248517	5	141201893	A	0	10	C	0.0004292
rs116557	5	141203359	G	0	10	T	0.0004292
rs123146	5	141204679	G	0	10	C	0.0004292
rs248508	5	141208344	A	0	10	G	0.0004292
rs248506	5	141208854	A	0	10	G	0.0004292
rs2974703	5	141225318	G	1	13	C	0.0003716
rs2974704	5	141227042	T	1	13	C	0.0003716
rs75718464	5	141231970	T	1	13	C	0.0003716
rs10054359	5	143357845	A	11	0	G	0.0007855
rs10477222	5	143359111	T	11	0	C	0.0007855
rs12717907	5	143364448	T	11	0	C	0.0007855
rs10056911	5	143365966	A	11	0	C	0.0007855
rs6870501	5	143366425	G	11	0	A	0.0007855
rs67611103	5	143368861	A	11	0	G	0.0007855
rs35152581	5	143370499	T	11	0	TC	0.0007855
rs7727120	5	143371748	A	11	0	G	0.0007855
rs13360967	5	143372189	T	11	0	G	0.0007855
rs10044882	5	143373540	A	11	0	G	0.0007855
rs10044910	5	143373611	A	11	0	G	0.0007855
rs10068921	5	143376594	T	11	0	C	0.0007855
rs9324935	5	143377050	T	11	0	C	0.0007855
rs67244803	5	143377715	A	11	0	G	0.0007855
rs10076085	5	143378264	A	11	0	G	0.0007855
rs10069477	5	143378291	G	11	0	A	0.0007855
rs13181480	5	143378452	G	11	0	C	0.0007855
rs10078432	5	143379748	A	11	0	G	0.0007855
rs10066351	5	143380917	T	11	0	C	0.0007855
rs1497746	5	143381414	T	11	0	A	0.0007855
rs34660587	5	143382771	T	11	0	TA	0.0007855
rs13175610	5	143384428	A	11	0	C	0.0007855
rs13174792	5	143384615	A	11	0	G	0.0007855
rs12717908	5	143387284	A	11	0	T	0.0007855

rs111437042	5	143390644	TG	11	0	T	0.0007855
rs56166697	5	143390645	G	11	0	C	0.0007855
rs28498587	5	143390662	C	11	0	T	0.0007855
rs28521517	5	143390745	G	11	0	A	0.0007855
rs6877207	5	143390802	G	11	0	A	0.0007855
rs28449742	5	143390816	T	11	0	C	0.0007855
rs7700905	5	143391092	A	11	0	G	0.0007855
rs7701240	5	143391310	T	11	0	G	0.0007855
rs7726003	5	143391442	C	11	0	T	0.0007855
rs7702656	5	143391500	A	11	0	C	0.0007855
rs28855539	5	143391967	T	11	0	A	0.0007855
rs28826677	5	143392041	T	11	0	C	0.0007855
rs28768503	5	143392065	C	11	0	T	0.0007855
rs34681142	5	143392128	C	11	0	T	0.0007855
rs72797441	5	143392278	T	11	0	C	0.0007855
rs72797443	5	143392286	T	11	0	C	0.0007855
rs35522635	5	143392424	T	11	0	C	0.0007855
rs34213275	5	143392484	A	11	0	G	0.0007855
rs28693642	5	143392766	A	11	0	G	0.0007855
rs3913201	5	143392946	G	11	0	A	0.0007855
rs3913200	5	143393115	A	11	0	G	0.0007855
rs3913199	5	143393155	T	11	0	C	0.0007855
rs13354382	5	143393378	G	11	0	C	0.0007855
rs13354385	5	143393402	T	11	0	C	0.0007855
rs13358741	5	143393599	T	11	0	G	0.0007855
rs34037977	5	143393853	T	11	0	C	0.0007855
rs35720433	5	143394238	G	11	0	C	0.0007855
rs17101368	5	143394562	G	11	0	A	0.0007855
rs13163496	5	143394734	G	11	0	C	0.0007855
rs112207493	5	143394765	GA	11	0	G	0.0007855
rs17101389	5	143397593	T	12	0	C	0.0003752
rs34656558	5	143650193	TAA	5	21	T	0.0004449
rs13187326	5	143738995	A	18	3	G	0.0009486
rs17387610	5	144091703	A	30	48	G	0.0009571
rs11749768	5	153877234	T	2	15	C	0.0004755
rs60663840	5	153879520	G	2	16	A	0.0002312
rs6580084	5	153882460	C	2	16	G	0.0002312
rs80164565	5	153884201	C	2	16	T	0.0002312
rs72803027	5	156154347	G	0	9	T	0.0009641
rs17053865	5	156154478	G	0	9	A	0.0009641
rs72803029	5	156155368	G	0	9	C	0.0009641

rs10515742	5	156155832	G	0	9	A	0.0009641
rs17053873	5	156164188	T	0	9	G	0.0009641
rs10515744	5	156166759	T	0	9	C	0.0009641
rs11955986	5	156168617	A	0	9	G	0.0009641
rs10515745	5	156170837	A	0	9	G	0.0009641
rs66744889	5	156174457	T	0	9	C	0.0009641
rs56297325	5	156175259	G	0	9	A	0.0009641
rs59428757	5	156179592	T	0	9	C	0.0009641
rs11135381	5	156182200	G	0	9	A	0.0009641
rs11135382	5	156182450	A	0	9	C	0.0009641
rs57452961	5	156182818	C	0	9	T	0.0009641
rs3215118	5	156184457	GT	0	9	G	0.0009641
rs3913020	5	156185734	G	0	9	A	0.0009641
rs3857412	5	156187182	C	0	9	G	0.0009641
rs1389818	5	156188019	C	0	9	T	0.0009641
rs56389540	5	156190493	G	0	9	A	0.0009641
rs10041876	5	156190796	T	0	9	A	0.0009641
rs1845479	5	156191214	C	0	9	T	0.0009641
rs3913482	5	156191515	T	0	9	C	0.0009641
rs11953631	5	156192264	T	0	9	C	0.0009641
rs2029776	5	156196045	G	0	9	A	0.0009641
rs56920379	5	156196226	T	0	9	A	0.0009641
rs56094322	5	156201303	G	0	9	A	0.0009641
rs3909322	5	156254003	A	0	9	G	0.0009641
rs1978426	5	156261279	T	0	9	C	0.0009641
rs9313318	5	156261737	G	0	9	A	0.0009641
rs10475787	5	156262172	C	0	9	G	0.0009641
rs7725502	5	156263206	A	0	9	C	0.0009641
rs10475501	5	156264032	A	0	9	C	0.0009641
rs9885363	5	156267197	A	0	9	G	0.0009641
rs7704664	5	156268656	C	0	9	T	0.0009641
rs55711014	5	156270765	C	0	9	A	0.0009641
rs7707893	5	156272432	T	0	9	C	0.0009641
rs6555703	5	156272490	T	0	9	C	0.0009641
rs12153343	5	158964827	T	27	45	A	0.0009136
rs35978513	5	160736639	A	3	16	ATT	0.0009912
rs6556667	5	162746270	C	5	19	T	0.0008415
rs1421840	5	162773548	G	19	3	A	0.0005082
rs2216559	5	162778631	G	21	3	A	0.0002475
rs34432869	5	162782745	A	20	3	G	0.000269
rs35836896	5	162789959	A	19	3	T	0.0005082

rs13159369	5	162801076	A	19	3	G	0.0005082
rs34472788	5	167637617	C	4	20	CT	0.0002424
rs11480110	5	167648116	CA	3	18	C	0.0002532
rs55982071	5	168253296	A	1	12	G	0.0007887
rs59344702	5	168254496	C	1	12	T	0.0007887
rs7726584	5	168254897	G	1	12	T	0.0007887
rs7707369	5	168255217	A	1	12	G	0.0007887
rs3797456	5	172754364	G	23	5	A	0.0006807
rs4867706	5	172781934	G	13	33	A	0.000122
rs2034235	5	172783518	T	12	32	C	0.0001005
rs1862428	5	172784614	C	12	32	T	0.0001005
rs10476071	5	172785705	A	21	41	G	0.0003509
rs202028398	5	177277758	G	27	8	T	0.0009854
rs6601226	5	177624390	G	48	21	T	0.0002492
rs2242213	5	180048316	G	1	12	C	0.0007887
rs369415913	5	180718703	C	17	2	T	0.000467
rs2816239	6	1448243	C	16	2	T	0.0008956
rs9405141	6	1580838	T	5	19	C	0.0008415
rs9503157	6	2369670	G	53	27	A	0.0006109
rs4296927	6	2369733	G	53	27	C	0.0006109
rs9503159	6	2371304	C	53	27	T	0.0006109
rs11752965	6	2373843	T	51	25	C	0.0005636
rs11242762	6	2379182	G	53	27	A	0.0006109
rs11752051	6	2379497	A	53	27	G	0.0006109
rs11756861	6	2379640	T	52	26	C	0.0003091
rs11242763	6	2380251	A	53	27	C	0.0006109
rs2503809	6	5388938	G	9	25	T	0.0008942
rs35967844	6	5389386	TA	7	23	T	0.0004919
rs2503814	6	5390860	A	7	24	G	0.0002593
rs17140417	6	5396100	G	7	22	A	0.0009169
rs13215393	6	5397426	A	7	22	G	0.0009169
rs35394307	6	5398032	T	7	22	TCTTC	0.0009169
rs2326606	6	5398329	A	7	22	G	0.0009169
rs62385371	6	5405445	C	7	22	T	0.0009169
rs2432774	6	5406604	G	25	43	C	0.0008649
rs35835664	6	5406899	C	7	22	T	0.0009169
rs12528042	6	5408762	C	7	22	T	0.0009169
rs2032986	6	5409181	G	25	43	A	0.0008649
rs2326607	6	5412968	A	7	22	G	0.0009169
rs1014427	6	5418852	G	7	22	C	0.0009169
rs1549363	6	5419594	T	7	22	G	0.0009169

rs5873980	6	5419708	C	7	22	CTTTACCGTTT	0.0009169
rs34269790	6	5420060	A	7	22	G	0.0009169
rs35719003	6	5420105	T	7	22	C	0.0009169
rs12524064	6	5422832	T	7	22	G	0.0009169
rs34499406	6	5423324	G	7	22	GC	0.0009169
rs11757581	6	5424511	T	7	22	C	0.0009169
rs11752854	6	5424762	A	7	22	G	0.0009169
rs9405266	6	5426228	A	22	41	G	0.0004309
rs11752504	6	5426450	C	7	22	T	0.0009169
rs4960090	6	5427879	T	7	22	C	0.0009169
rs35420040	6	5429820	A	7	22	C	0.0009169
rs13210571	6	5429888	G	7	22	A	0.0009169
rs11753202	6	5430181	A	5	22	G	0.0002207
rs11243011	6	5431340	G	7	22	A	0.0009169
rs5873981	6	5434330	TA	7	22	T	0.0009169
rs1424736	6	5434447	T	7	22	C	0.0009169
rs200190	6	5483696	T	24	43	G	0.000471
rs4476877	6	6988545	C	14	1	T	0.0007094
rs9379896	6	10886949	C	20	2	T	0.00006204
rs9358956	6	10887253	G	18	1	C	0.00004271
rs9348724	6	10887276	C	18	1	G	0.00004271
6:17167167	6	17167167	G	28	8	A	0.0006092
rs35544048	6	17758844	C	5	21	G	0.0002243
rs11963363	6	22914924	C	11	28	A	0.0008103
rs12526188	6	22919962	C	0	9	T	0.0009641
rs13201703	6	22921471	A	0	9	C	0.0009641
rs59885141	6	22926116	C	0	9	T	0.0009641
rs34947088	6	22929340	T	0	9	A	0.0009641
rs11967825	6	22930117	A	0	9	T	0.0009641
rs13212187	6	22941703	A	0	9	G	0.0009641
rs13211061	6	22944597	C	0	9	A	0.0009641
rs12527826	6	22947966	T	0	9	G	0.0009641
rs1569701	6	22966154	A	0	9	G	0.0009641
rs11965774	6	22971200	G	0	9	C	0.0009641
rs35588537	6	22972197	T	0	9	G	0.0009641
rs13211265	6	22974647	T	0	9	G	0.0009641
rs12525343	6	23207471	G	1	13	T	0.0003716
6:23291477	6	23291477	A	32	9	ACATACATTATAC	0.00005996
6:23406822	6	23406822	G	46	21	T	0.0007859
rs72839070	6	25532885	G	0	10	T	0.0004292
rs9356992	6	25925400	A	0	10	G	0.0004292

rs9295676	6	25928376	T	0	10	G	0.0004292
rs62394491	6	25934106	T	0	9	C	0.0009641
rs10456346	6	26966846	G	0	9	A	0.0009641
rs10456347	6	26966853	G	0	9	A	0.0009641
rs10456348	6	26969033	A	0	9	T	0.0009641
rs146539976	6	26977290	G	0	9	T	0.0009641
rs3864294	6	28641957	G	22	41	A	0.0004309
rs10456364	6	28681444	C	26	44	T	0.0002393
rs9391676	6	28683743	T	28	47	C	0.0005255
rs200314214	6	28685339	T	50	24	C	0.0008877
rs1033569	6	29257772	C	46	21	T	0.0007859
rs1033568	6	29257967	G	47	21	A	0.0004473
6:29264095	6	29264095	A	46	21	AT	0.0007859
rs4713207	6	29264655	A	46	21	G	0.0007859
rs9257687	6	29267175	A	47	21	G	0.0004473
rs4713208	6	29283579	T	45	15	C	0.00001097
rs720831	6	29284518	A	45	15	G	0.00001097
rs10447393	6	29316852	T	46	16	C	0.00001332
rs7754402	6	29328834	C	39	15	T	0.0004501
6:29338562	6	29338562	T	52	26	TAAA	0.0005884
rs28749522	6	29338563	G	52	26	C	0.0005884
rs4713213	6	29338962	G	52	26	A	0.0005884
6:29339965	6	29339965	CA	52	26	C	0.0005884
rs10807053	6	29340585	G	52	26	C	0.0005884
rs3094561	6	29404157	T	28	47	G	0.0005255
rs3094574	6	29422092	A	29	47	G	0.0009467
rs3117194	6	29426008	T	28	47	C	0.0005255
rs3128854	6	29431698	A	28	47	G	0.0005255
rs2107191	6	29434295	C	28	47	T	0.0005255
rs3117196	6	29439779	C	28	46	T	0.0009321
rs3094573	6	29446632	G	28	46	T	0.0009321
rs2189371	6	29449840	C	28	46	T	0.0009321
rs3128844	6	29459260	G	28	46	C	0.0009321
rs3131854	6	29611885	T	10	28	C	0.0003491
rs3095292	6	29628620	A	9	27	G	0.0001402
rs2071653	6	29635928	T	9	27	C	0.0002633
rs3095291	6	29637710	C	9	27	G	0.0002633
rs3117290	6	29649944	T	9	26	G	0.0004896
rs1610726	6	29738665	G	38	15	C	0.0007851
6:29738688	6	29738688	C	38	15	T	0.0007851
rs1610722	6	29761467	C	42	18	A	0.0006782

rs3094170	6	29829265	T	7	22	C	0.0009169
rs3128904	6	29831466	A	5	20	G	0.0004383
rs3128900	6	29833057	G	7	22	T	0.0009169
rs3115612	6	29851443	A	7	23	G	0.0004919
rs9259141	6	29852203	G	7	23	C	0.0004919
rs3094646	6	29852855	C	7	23	T	0.0004919
rs3094645	6	29853936	C	7	23	T	0.0004919
rs9259191	6	29854280	T	7	23	C	0.0004919
rs9259192	6	29854392	C	7	23	T	0.0004919
rs3132697	6	29854539	A	7	23	G	0.0004919
rs1054176	6	29854703	A	7	23	G	0.0004919
rs3132696	6	29855331	A	7	23	G	0.0004919
rs59958576	6	29855580	GCCCTGA	10	28	G	0.0003491
rs3128876	6	29856178	C	7	23	T	0.0004919
rs3115604	6	29857730	G	7	23	C	0.0004919
rs9259265	6	29857852	A	7	23	G	0.0004919
rs3115603	6	29858213	G	7	23	C	0.0004919
rs9259329	6	29861134	T	9	25	G	0.0008942
rs3115595	6	29861795	A	7	23	T	0.0004919
6:29862476	6	29862476	T	7	23	C	0.0004919
rs77646284	6	29862699	C	7	23	T	0.0004919
rs3094185	6	29867395	T	7	23	A	0.0004919
rs3094167	6	29872869	A	8	24	G	0.0006758
rs9259585	6	29873562	G	7	23	A	0.0004919
rs9259588	6	29873671	T	7	23	G	0.0004919
rs420306	6	29874165	A	7	23	T	0.0004919
rs9259640	6	29875891	C	7	23	T	0.0004919
rs9259641	6	29875965	G	7	23	A	0.0004919
rs9259645	6	29876102	G	7	23	T	0.0004919
rs2253517	6	29876774	A	7	23	C	0.0004919
rs2253518	6	29876775	G	7	23	A	0.0004919
rs1632923	6	29877000	A	7	23	G	0.0004919
rs2517805	6	29877341	A	7	23	G	0.0004919
rs3094143	6	29877483	C	45	19	T	0.0003833
rs3128992	6	29877572	G	45	19	A	0.0003833
rs1632922	6	29877590	A	7	23	G	0.0004919
rs415216	6	29877976	A	10	28	G	0.0003491
rs433478	6	29878055	C	54	27	T	0.0003437
rs2517800	6	29878138	T	10	28	C	0.0003299
6:29878143	6	29878143	GA	10	28	G	0.0001505
rs2734930	6	29878262	G	7	23	A	0.0004919

rs2571406	6	29878308	A	10	28	C	0.0003491
rs2905782	6	29878432	A	10	28	G	0.0003491
rs2975022	6	29878433	C	10	28	A	0.0003491
rs2524031	6	29878456	A	7	23	C	0.0004919
rs2269853	6	29878461	T	54	27	C	0.0003437
rs3128990	6	29878483	T	54	27	C	0.0003437
rs390703	6	29878657	A	10	29	C	0.0001875
rs2975019	6	29878686	T	10	28	C	0.0003491
rs2524029	6	29878757	C	7	23	T	0.0004919
rs403306	6	29878779	A	10	28	C	0.0003491
rs419316	6	29878824	C	10	28	T	0.0003491
rs2517799	6	29878825	A	8	27	G	0.00009961
rs441627	6	29878892	T	10	28	A	0.0003491
rs2524028	6	29878897	T	8	27	G	0.00009961
rs381379	6	29879084	A	10	28	G	0.0003491
rs2524027	6	29879105	T	7	23	C	0.0004919
rs391705	6	29879173	T	10	28	C	0.0003491
rs406344	6	29879252	A	10	28	T	0.0003491
rs9259694	6	29879427	G	10	28	C	0.0003491
rs9259697	6	29879472	A	10	28	G	0.0003491
rs9259698	6	29879509	G	10	28	A	0.0003491
rs2524024	6	29879529	C	7	23	T	0.0004919
rs9259699	6	29879536	A	10	28	G	0.0003491
rs9259700	6	29879563	C	10	28	T	0.0003491
rs9259701	6	29879584	C	10	28	T	0.0003491
rs2734929	6	29879655	T	10	28	C	0.0003491
rs2734928	6	29879804	G	7	23	A	0.0004919
rs2524023	6	29879911	C	8	27	T	0.00009961
rs412982	6	29880050	T	10	28	C	0.0003491
rs446822	6	29880181	A	10	28	G	0.0003491
6:29880395	6	29880395	AG	10	28	A	0.0003491
rs2893996	6	29880397	C	10	28	T	0.0003491
rs368685	6	29880406	A	10	28	G	0.0003491
rs2254042	6	29880424	C	10	28	A	0.0003491
rs2571409	6	29880428	C	10	28	T	0.0003491
rs1611440	6	29880429	A	7	23	G	0.0004919
rs1611442	6	29880825	T	8	27	C	0.00009961
rs1611443	6	29880904	C	7	23	T	0.0004919
rs1611444	6	29881093	G	7	23	A	0.0004919
rs2734926	6	29881244	G	10	28	A	0.0003491
rs1610754	6	29881268	A	8	27	G	0.00009961

rs1611447	6	29881754	T	7	23	G	0.0004919
rs1611452	6	29882005	A	7	23	G	0.0004919
rs1611457	6	29882147	A	8	27	T	0.00009961
rs2734924	6	29882174	C	8	27	G	0.00009961
rs1611458	6	29882297	T	7	23	G	0.0004919
rs2734923	6	29882749	T	8	27	A	0.00009961
rs1611462	6	29882790	A	8	27	G	0.00009961
rs1610755	6	29882985	A	8	27	G	0.0001129
rs1610756	6	29883728	T	7	23	C	0.0004919
rs1611474	6	29884075	A	8	27	G	0.00009961
rs1611478	6	29884533	T	7	23	C	0.0004919
rs1611479	6	29884541	A	8	27	G	0.00009961
rs1611481	6	29885169	G	7	23	C	0.0004919
rs1611484	6	29885307	G	7	23	T	0.0004919
rs1610757	6	29886191	C	7	23	A	0.0004919
rs1610619	6	29886615	A	7	23	G	0.0004919
rs1611504	6	29887053	C	7	23	T	0.0004919
rs67378810	6	29887205	GTC	7	23	G	0.0004919
rs1632913	6	29887277	T	7	23	C	0.0004919
rs1632912	6	29887497	T	8	27	G	0.00009961
rs1655949	6	29887639	A	7	23	T	0.0004919
rs1625356	6	29887885	A	8	27	G	0.00009961
rs2508042	6	29888317	A	8	27	G	0.00009961
rs1655944	6	29888396	T	7	23	C	0.0004919
rs1632911	6	29889368	T	7	23	C	0.0004919
6:29890146	6	29890146	G	7	23	T	0.0004919
rs2517789	6	29890224	A	7	23	T	0.0004919
rs1611394	6	29890563	G	7	23	A	0.0004919
rs1611401	6	29890950	A	7	23	G	0.0004919
rs1611408	6	29891423	T	7	23	C	0.0004919
rs1611413	6	29891625	T	7	23	G	0.0004919
rs1610751	6	29893040	C	7	23	T	0.0004919
rs1611430	6	29893461	G	7	23	A	0.0005609
rs1632907	6	29894794	A	7	23	G	0.0004144
rs1655936	6	29895649	G	7	25	A	0.0001175
6:29895667	6	29895667	C	8	26	A	0.0001738
rs3863209	6	29895928	G	7	25	A	0.0001343
rs9259842	6	29895973	C	7	23	G	0.0004919
rs2517752	6	29897018	A	7	23	G	0.0004337
rs1655932	6	29897102	G	7	25	A	0.0001343
rs389725	6	29897129	C	9	26	T	0.0004896

rs1632902	6	29897412	C	39	16	G	0.0009032
rs1632901	6	29897736	T	7	25	C	0.0001343
6:29897759	6	29897759	G	7	23	A	0.0004919
rs2471864	6	29897770	A	39	16	G	0.0009032
rs6926454	6	29897872	G	7	25	A	0.0001343
rs9259870	6	29897944	A	7	25	G	0.0001343
rs9259876	6	29898117	A	7	25	G	0.0001343
rs9259890	6	29898413	A	7	25	C	0.0001175
rs2517742	6	29898751	T	39	16	C	0.0009032
rs2517741	6	29898806	G	7	25	T	0.0001343
rs2517740	6	29898821	G	7	25	C	0.0001343
rs2394705	6	29899207	C	39	16	T	0.0009032
rs1655928	6	29899461	G	39	16	A	0.0009032
rs2524004	6	29901899	C	7	23	G	0.0004919
rs1611304	6	29901919	T	7	23	C	0.0004919
rs9259925	6	29902197	C	7	23	G	0.0004919
rs1611327	6	29902837	A	7	23	G	0.0005609
rs1610618	6	29903111	T	7	23	C	0.0004919
rs2571421	6	29904309	C	7	23	T	0.0004919
rs1632896	6	29904808	A	7	23	G	0.0004919
rs1632895	6	29904838	T	7	25	G	0.0001343
rs1655925	6	29904840	C	7	23	T	0.0004919
rs1632894	6	29905158	A	7	23	G	0.0004919
rs1632893	6	29905193	G	7	23	A	0.0004919
rs1655920	6	29908667	A	7	23	G	0.0004919
rs1632889	6	29909181	A	7	23	C	0.0004919
rs2916801	6	29910286	C	7	23	T	0.0004919
rs9260142	6	29910820	G	7	23	C	0.0004919
rs67054159	6	29911457	G	7	22	GT	0.0009169
rs2571381	6	29912830	T	7	23	C	0.0004919
rs2916802	6	29912935	C	6	21	T	0.0006518
rs9260205	6	29913074	A	7	23	G	0.0004919
rs41546815	6	29913078	A	7	23	G	0.0004919
rs13488	6	29913638	G	7	23	C	0.0004919
rs2248006	6	29913672	G	7	23	A	0.0004919
rs2571382	6	29913727	T	7	23	C	0.0004919
rs1655902	6	29916855	G	7	24	A	0.0002593
rs1632880	6	29917610	C	7	23	G	0.0004919
rs1632879	6	29917703	G	7	23	A	0.0004919
rs1655906	6	29918254	A	7	23	T	0.0004919
rs1655907	6	29918587	T	7	23	C	0.0004919

6:29920465	6	29920465	A	7	23	C	0.0004919
rs885905	6	30890431	A	13	30	G	0.0007578
rs2257347	6	30892054	A	11	30	G	0.0002453
rs2532941	6	30892521	G	13	31	A	0.0004205
rs1043483	6	30893728	C	13	30	T	0.0007578
rs2532932	6	30895444	A	10	27	G	0.0006386
rs2532925	6	30899163	G	13	31	A	0.0004205
rs2253588	6	30899377	C	13	31	G	0.0004205
rs3095148	6	30933967	T	38	15	C	0.0007851
rs9281099	6	30978979	GT	47	22	G	0.0008379
rs2517503	6	31032407	A	3	16	C	0.0009912
rs2517438	6	31064100	T	3	16	C	0.0009912
rs368354370	6	31121874	CAA	31	49	C	0.0009634
rs130066	6	31122315	C	51	23	G	0.000156
rs3130453	6	31124849	T	54	24	C	0.00004651
rs3095239	6	31126790	A	31	49	G	0.0009634
rs3094187	6	31126944	T	55	27	C	0.0002959
rs6905862	6	31127602	A	51	23	G	0.000156
rs1419881	6	31130593	A	52	26	G	0.0005884
rs13409	6	31132140	A	51	23	G	0.000156
rs145919884	6	31133577	A	24	43	AAAGCCC	0.000471
rs3132526	6	31134175	T	55	27	C	0.0002959
rs1419880	6	31135197	C	55	26	T	0.00009769
rs2021375	6	31135387	G	54	27	A	0.0003437
6:31135730	6	31135730	TC	55	26	T	0.00009769
rs3094195	6	31135966	T	55	27	C	0.0002959
rs3094193	6	31137774	G	28	46	T	0.0009321
rs2077010	6	31138377	T	49	23	C	0.0002681
rs885952	6	31139490	T	51	23	C	0.000156
rs3132520	6	31140008	C	32	50	T	0.0009655
rs885951	6	31140091	A	51	23	C	0.000156
rs885950	6	31140152	C	51	25	A	0.0005636
rs885948	6	31140532	G	55	27	A	0.0002959
rs887464	6	31145920	T	51	23	C	0.000156
6:31204204	6	31204204	TTCTTTCTA	45	20	T	0.0003895
rs142259499	6	31341771	A	39	15	G	0.0004501
rs2844508	6	31436499	G	23	41	C	0.0008021
rs2523669	6	31437872	C	23	41	T	0.0008021
rs2596450	6	31438243	G	23	41	A	0.0008021
rs2523663	6	31439740	G	24	42	T	0.0008352
rs2516423	6	31449216	T	54	26	A	0.00008952

rs16881066	6	35772722	G	13	0	A	0.0001783
rs2237232	6	35812379	C	13	0	T	0.0001783
rs3804456	6	35818395	C	13	0	T	0.0001783
rs2301509	6	35854936	T	13	0	C	0.0001783
rs74896584	6	35878713	T	13	0	C	0.0001783
rs76985948	6	35895153	T	13	0	C	0.0001783
rs9368895	6	35913096	A	13	0	C	0.0001783
rs851044	6	35972766	A	27	45	G	0.0009136
rs4711445	6	36205532	G	14	1	C	0.0007094
rs16886912	6	36207973	A	14	1	G	0.0007094
rs4713939	6	36209623	A	20	3	G	0.000269
rs150201683	6	41493770	A	0	9	G	0.0009641
rs79102660	6	45466010	C	18	3	T	0.0009486
rs520409	6	47802504	C	20	38	T	0.0009963
rs693770	6	47805729	C	20	38	A	0.0009963
rs576826	6	47806706	G	20	38	A	0.0009963
rs578532	6	47806895	A	27	45	G	0.0009136
rs559028	6	47809908	T	20	38	C	0.0009963
rs559768	6	47809949	T	20	38	C	0.0009963
rs563587	6	47810381	C	27	45	T	0.0009136
rs528229	6	47810445	G	20	38	A	0.0009963
rs482434	6	47811272	C	20	38	T	0.0009963
rs517025	6	47819141	G	20	38	A	0.0009963
rs579329	6	47822681	T	27	45	C	0.0009136
rs540010	6	47822907	A	20	38	T	0.0009963
rs514974	6	47825345	C	20	38	T	0.0009963
rs9463962	6	53564752	A	16	2	G	0.0008956
rs6912139	6	53565153	A	19	2	G	0.0001229
rs6937819	6	53569785	C	18	2	T	0.0002408
rs9464133	6	54625502	T	43	19	A	0.000761
rs11754220	6	54629589	G	44	19	C	0.0006784
rs35741450	6	55137395	CT	26	8	C	0.000972
rs13199480	6	55878415	A	16	36	G	0.0001978
rs9370477	6	55880322	T	12	31	C	0.0001515
rs9349800	6	55885348	T	10	27	C	0.0006386
rs2206679	6	55886109	T	21	40	C	0.0006238
rs2223660	6	55886898	T	10	27	C	0.0006386
rs6933744	6	55887025	A	10	27	C	0.0006386
rs4712111	6	55887274	A	10	27	G	0.0006386
rs11970687	6	56041487	G	20	38	C	0.0009963
6:64251929	6	64251929	G	16	2	T	0.0008956

rs72875947	6	65698962	T	0	9	C	0.0009641
rs2273428	6	70643018	G	25	48	A	0.00003878
rs9360410	6	70652566	A	29	48	G	0.0005332
rs3805963	6	70654658	A	29	48	G	0.0005332
rs960816	6	71008490	T	21	4	C	0.0008104
rs9354912	6	71008745	G	21	4	A	0.0008104
rs9446281	6	71331307	G	0	10	A	0.0004197
6:74067800	6	74067800	TTATC	4	17	T	0.0008872
6:74067804	6	74067804	C	4	17	T	0.0008872
rs9447974	6	77836688	A	32	10	G	0.000288
6:83237475	6	83237475	C	12	0	CT	0.0003752
6:83546524	6	83546524	TG	14	1	T	0.0007094
6:83546527	6	83546527	TG	15	1	T	0.0003561
rs1040544	6	83574391	C	51	24	T	0.0003012
rs220398	6	86826836	G	23	5	C	0.0006807
rs220400	6	86839936	A	23	5	G	0.0006807
rs11757976	6	88836870	G	35	12	A	0.0004754
6:91579114	6	91579114	T	30	8	TC	0.0003626
rs12203938	6	93829029	G	0	9	T	0.0009641
rs35031934	6	94141464	AT	40	16	A	0.0005221
rs591356	6	94162298	T	26	44	C	0.0008911
rs590881	6	94162429	A	26	44	T	0.0008911
rs684081	6	94163373	G	30	48	T	0.0009571
rs630517	6	94163617	G	30	48	A	0.0009571
rs655710	6	94165129	G	26	44	A	0.0008911
rs11463424	6	94165165	AT	30	48	A	0.0009571
rs674517	6	94165491	G	30	48	A	0.0009571
rs35984410	6	94165894	CA	30	48	C	0.0009571
rs643976	6	94167745	A	30	48	C	0.0009571
rs595153	6	94169456	C	30	48	T	0.0009571
rs491112	6	94169647	A	30	48	C	0.0009571
rs628135	6	94172250	T	26	44	G	0.0008911
rs662410	6	94172491	A	30	48	G	0.0009571
rs151250987	6	94172802	TCAGA	30	48	T	0.0009571
rs644400	6	94174210	A	26	44	G	0.0008911
rs631463	6	94174854	T	26	44	C	0.0008911
rs619087	6	94175279	G	26	44	A	0.0008911
rs902160	6	94214998	A	15	33	G	0.0005689
rs9445122	6	94221770	A	16	34	G	0.0006492
rs9363086	6	94227308	G	16	34	T	0.0006492
rs7746862	6	94246104	C	16	34	T	0.0006492

rs2325505	6	94249481	C	16	34	G	0.0006492
rs9345366	6	94249559	G	16	34	A	0.0006492
rs9445132	6	94256087	A	16	34	G	0.0006492
rs7758621	6	94262332	T	16	34	C	0.0006492
rs1489818	6	94262344	T	16	34	C	0.0006492
rs976765	6	94268297	G	16	34	A	0.0006492
rs382257	6	95004088	C	9	26	T	0.0004896
rs434310	6	95004561	A	9	26	G	0.0004896
rs426370	6	95009400	A	9	27	G	0.0002633
rs10944744	6	95190344	A	27	48	G	0.0001523
rs9342418	6	95197387	C	25	46	T	0.0001445
rs1022787	6	95202197	G	25	46	A	0.0001445
rs222550	6	95211989	C	25	46	T	0.0001445
rs222554	6	95214209	T	23	41	C	0.0008021
rs222555	6	95215610	C	23	41	G	0.0008021
rs222558	6	95216589	A	23	41	G	0.0008021
rs1741377	6	95220828	A	23	41	C	0.0008021
rs6929516	6	95220915	C	17	35	G	0.0007327
rs2819971	6	95278063	G	51	24	T	0.0003012
rs2750030	6	95278533	T	51	24	A	0.0003012
rs6905809	6	95278921	C	11	31	A	0.0000622
rs2224317	6	95285511	C	51	24	T	0.0003012
rs2208170	6	95285661	A	51	24	T	0.0003012
rs2750024	6	95303185	C	50	25	T	0.0009811
rs2750023	6	95304525	A	50	25	C	0.0009811
rs79731152	6	97223220	A	14	1	G	0.0007094
rs946120	6	97544329	A	49	23	G	0.0005083
rs34070466	6	97823031	AT	8	24	A	0.0006758
rs35795392	6	98054182	C	6	22	T	0.000342
rs9398069	6	106601165	C	24	44	T	0.0002594
rs9372118	6	106601355	C	24	44	A	0.0002594
rs10717926	6	106604781	A	26	44	AT	0.0008911
rs11755599	6	106847758	G	5	20	T	0.0004383
rs11153721	6	118424187	T	30	9	C	0.0004612
rs12209804	6	118424296	T	30	9	A	0.0004612
rs9387558	6	118426567	T	30	9	A	0.0004612
rs9387559	6	118426951	G	30	9	A	0.0004612
rs3924525	6	118427216	G	45	19	C	0.0003833
rs12194270	6	118430123	G	30	9	A	0.0004612
rs9320641	6	118434086	A	30	9	G	0.0004612
rs12204646	6	118434647	G	30	9	T	0.0004612

rs34666479	6	118435702	ATT	30	9	A	0.0004612
rs9387560	6	118435846	C	30	9	T	0.0004612
rs12203615	6	118438261	T	30	9	C	0.0004612
rs28817378	6	118448386	A	31	9	C	0.0002572
rs11153723	6	118448635	C	31	9	G	0.0002572
rs427474	6	118450944	T	31	9	C	0.0002572
rs141635890	6	118452942	A	31	9	AATTAT	0.0002572
rs205975	6	118455142	G	32	9	T	0.0001413
rs11334484	6	118459873	CT	32	9	C	0.0001413
rs205972	6	118460362	A	32	9	G	0.0001413
rs6910376	6	118460555	G	46	19	A	0.0002121
rs2152076	6	118461619	C	46	19	T	0.0002121
rs140772634	6	118461760	T	46	21	TG	0.0004215
rs55845306	6	118461761	A	33	10	T	0.0001422
rs56388357	6	118462096	G	46	19	T	0.0002121
rs205971	6	118462913	G	32	9	A	0.0001413
rs74384532	6	118463728	AT	46	19	A	0.0002121
rs11756834	6	118465674	T	46	19	C	0.0002121
rs11754433	6	118465941	G	46	19	A	0.0002121
rs62423695	6	118466219	C	46	19	T	0.0002121
rs12662262	6	118466410	C	46	19	T	0.0002121
rs2051615	6	118467905	A	33	9	C	0.00007642
rs6940272	6	118471856	T	46	19	C	0.0002121
rs205968	6	118473563	C	32	9	T	0.0001413
rs5005997	6	118474562	T	46	19	C	0.0002121
rs205966	6	118475798	G	33	10	A	0.0003125
rs12530139	6	129775084	A	4	18	T	0.0009446
rs9402159	6	129982039	T	42	17	A	0.0003422
rs9388722	6	129982285	G	42	15	A	0.0001339
rs4482995	6	129984362	A	43	17	T	0.0001922
rs13203844	6	129985147	C	43	17	T	0.0001922
rs12193673	6	129985921	A	43	17	G	0.0001922
rs9398914	6	129986453	G	39	16	C	0.0009032
rs6899523	6	129986588	G	43	17	A	0.0001922
rs6922711	6	129986792	C	43	17	T	0.0001922
rs9402160	6	129987987	G	43	17	A	0.0001922
rs11450417	6	129988030	GC	43	17	G	0.0001922
rs6906744	6	129988135	T	43	17	C	0.0001922
rs78917176	6	129988199	G	42	17	T	0.0003422
rs79069340	6	129988200	A	42	17	T	0.0003422
rs76543028	6	129988201	A	42	17	C	0.0003422

rs9398915	6	129988553	A	43	17 T	0.0001922
rs9402162	6	129988892	C	43	17 G	0.0001922
rs7750456	6	129989333	T	43	17 C	0.0001922
rs4379317	6	129989626	G	43	17 A	0.0001922
rs9375720	6	130835901	A	7	22 G	0.0009169
rs4521612	6	139052870	A	9	25 C	0.0008942
rs4285339	6	139053155	A	9	25 G	0.0008942
rs4896395	6	139054359	G	9	25 C	0.0008942
rs6570275	6	139059783	T	16	35 C	0.000362
rs76184559	6	142561359	G	11	0 T	0.0007855
rs7765677	6	143259563	G	14	0 A	0.00008428
rs78806794	6	143305594	A	16	2 C	0.0008956
rs142413193	6	143320209	A	16	2 G	0.0008956
rs10484586	6	143322428	T	16	2 C	0.0008956
rs7739046	6	148098356	A	11	0 G	0.0007855
rs7775245	6	148890218	T	22	41 C	0.0004309
rs73005910	6	150429880	A	16	1 C	0.0001772
rs7758196	6	150445836	G	16	2 C	0.0008956
rs13211170	6	152610972	G	28	8 A	0.0005481
rs9383984	6	152626689	G	30	48 A	0.0009571
rs11453636	6	152734821	AG	6	21 A	0.0006518
rs742784	6	152736737	C	8	24 A	0.0006758
rs761408	6	152736812	T	8	24 C	0.0006758
rs2092602	6	152737092	C	8	24 T	0.0006758
rs7738528	6	152738335	C	8	24 A	0.0006758
rs7738706	6	152738409	G	8	24 A	0.0006758
rs7756247	6	152738424	A	8	24 G	0.0006758
rs62426960	6	152738622	G	8	24 A	0.0006758
rs34651734	6	152740681	TA	9	25 T	0.0008942
rs6557219	6	152743448	A	9	25 G	0.0008942
rs6913500	6	152744007	T	9	25 C	0.0008942
rs9479313	6	152744750	C	6	21 T	0.0006518
rs2295043	6	152764485	T	5	19 C	0.0008415
rs11753918	6	153504188	C	16	2 T	0.0008956
rs9397270	6	156163798	C	51	25 T	0.0005636
rs4455684	6	156191577	G	44	19 T	0.0006784
rs6927902	6	156191899	C	44	19 G	0.0006784
rs9397272	6	156192287	A	44	19 G	0.0006784
rs9397859	6	156214337	G	29	47 C	0.0009467
rs13210479	6	159805224	C	35	13 T	0.000996
rs783176	6	161170835	G	17	2 A	0.000467

rs1084653	6	161182115	A	16	2	C	0.0008956
rs35692234	6	161182859	CA	16	2	C	0.0008956
rs2465835	6	161186537	G	16	2	A	0.0008956
rs2465836	6	161187044	A	16	2	G	0.0008956
rs1271391	6	161190851	G	17	2	T	0.000467
rs62436699	6	161190945	C	15	1	G	0.0003561
rs1652498	6	161191645	G	17	2	T	0.000467
rs36032205	6	161191858	A	17	2	AG	0.000467
rs1740447	6	161193779	A	17	2	G	0.000467
rs1652501	6	161193959	G	17	2	C	0.000467
rs1782602	6	161199887	A	18	3	G	0.0009486
rs1740438	6	161199900	C	18	3	T	0.0009486
rs1652489	6	161200191	A	17	2	G	0.000467
rs1652492	6	161201024	A	17	2	G	0.000467
rs1367216	6	161203454	C	17	2	T	0.000467
rs1740430	6	161211997	A	17	2	G	0.000467
rs1652456	6	161215051	T	17	2	A	0.000467
rs1247572	6	161222490	G	17	2	A	0.000467
rs1740466	6	161225836	T	17	2	C	0.000467
rs145349920	6	161225900	TA	17	2	T	0.000467
rs34026567	6	161225901	G	17	2	T	0.000467
rs74196484	6	161268783	A	18	3	G	0.0009486
rs2489929	6	161270781	T	18	3	C	0.0009486
rs9347601	6	162636810	C	10	30	G	0.00009899
rs4235934	6	162645907	G	25	43	T	0.0008649
rs7771463	6	162659272	C	6	21	G	0.0006518
rs12203066	6	162660066	A	6	21	G	0.0006518
rs12175150	6	162661445	C	6	21	G	0.0006518
rs7750058	6	162684854	T	1	12	C	0.0007887
rs79711914	6	162687612	T	1	12	C	0.0007887
rs9347607	6	162689104	A	1	12	T	0.0007887
rs7744581	6	162691033	T	1	12	C	0.0007887
rs11969329	6	162693329	A	1	12	C	0.0007887
rs9355996	6	162696005	T	1	12	C	0.0007887
rs9347610	6	162702832	T	1	12	C	0.0007887
rs9355394	6	162703082	T	1	12	C	0.0007887
rs9364645	6	162705879	C	1	12	T	0.0007887
rs7740578	6	162708551	C	1	12	G	0.0007887
rs74352937	6	162709548	T	1	12	C	0.0007887
rs77424716	6	162712953	G	0	9	A	0.0009641
rs77743401	6	162714571	T	0	9	C	0.0009641

rs9347613	6	162714980	T	0	9	G	0.0009641
rs9355999	6	162716154	A	0	9	G	0.0009641
rs79581497	6	169081533	G	0	9	A	0.0009641
rs76675635	6	169902302	A	5	19	G	0.0008415
rs140215843	6	169929414	C	0	10	G	0.0004292
rs71010609	6	169990056	C	0	9	CAACAAGTCCTGA	0.0009641
rs34574309	7	7782647	CTA	47	22	C	0.0008379
rs4725022	7	7782661	A	47	22	G	0.0008379
rs2024372	7	7782919	A	54	26	G	0.0001819
rs2024373	7	7782982	A	54	26	G	0.0001819
rs138239053	7	7783317	G	54	26	GTATATGTATATG	0.0001819
rs2110556	7	7783587	A	54	26	G	0.0001819
rs12702642	7	7783919	T	54	26	C	0.0001819
rs35562533	7	7784322	A	55	26	G	0.00009769
rs2189906	7	8464974	G	28	6	A	0.00008463
rs2214790	7	10075603	G	0	9	A	0.0009641
rs4029461	7	11393850	C	26	6	G	0.000291
7:13658741	7	13658741	C	0	10	T	0.0004292
rs12699545	7	13796604	T	26	7	A	0.0007902
rs17732862	7	13815500	A	28	8	C	0.0006092
rs35329533	7	13821882	A	29	8	G	0.0003391
rs17167515	7	13822225	T	28	8	C	0.0006092
rs11980595	7	13822381	C	28	8	T	0.0006092
rs11980617	7	13822453	C	28	8	T	0.0006092
rs11980650	7	13822542	C	28	8	T	0.0006092
rs73062249	7	13825205	C	26	7	T	0.0007902
rs6949431	7	13825628	A	26	7	G	0.0007902
rs35336443	7	13826075	CA	26	7	C	0.0007902
rs35166573	7	13826104	A	26	7	AAT	0.0007902
rs186715394	7	18079316	C	0	9	T	0.0009641
rs2704280	7	18640746	T	31	49	C	0.0009634
rs4719620	7	20637759	T	27	47	G	0.0002837
7:20720623	7	20720623	T	12	0	TTC	0.000374
rs9691059	7	21429939	C	5	19	T	0.0008415
7:26066689	7	26066689	G	16	2	A	0.0008956
rs12333975	7	29549336	C	14	31	T	0.0008795
rs2302617	7	29605935	A	21	4	G	0.0008104
rs28362687	7	30949949	T	1	12	A	0.0007887
rs55867525	7	37271619	A	19	3	G	0.0005082
rs4723843	7	39337424	A	21	4	G	0.0008104
rs11310175	7	39351788	GA	21	4	G	0.0008104

rs2329396	7	39534540	A	47	21 G	0.0004473
rs370564210	7	47045993	A	2	14 T	0.0009649
rs34778134	7	47099416	G	14	1 A	0.0007094
rs56017904	7	47100078	T	14	1 C	0.0007094
rs55710472	7	47122464	T	19	2 C	0.0001229
rs71545970	7	47122753	C	19	2 G	0.0001229
rs71545971	7	47124934	C	19	2 T	0.0001229
rs13228000	7	47126710	G	19	2 A	0.0001229
rs12155121	7	47127677	A	19	2 G	0.0001229
rs34740359	7	47128115	C	19	2 T	0.0001229
rs17446835	7	47128635	C	19	2 T	0.0001229
rs12155471	7	47129422	G	19	2 A	0.0001229
rs35849184	7	47130395	CG	19	2 C	0.0001229
rs34436300	7	47134660	A	19	2 G	0.0001229
rs34644289	7	47134989	G	19	2 C	0.0001229
rs369251775	7	47136324	G	16	2 GA	0.0007358
rs11524610	7	47136472	G	18	2 T	0.0002408
rs10499651	7	47137770	A	18	2 G	0.0002408
rs35173793	7	47141298	A	18	2 G	0.0002408
rs35305609	7	47143515	G	18	2 A	0.0002408
rs71545975	7	47144505	A	16	1 G	0.000157
rs75843596	7	47145560	A	17	2 G	0.000467
rs76840621	7	47146824	A	17	2 G	0.000467
rs35013724	7	47148436	G	17	2 T	0.000467
rs35341942	7	47151123	T	20	1 TA	0.000009827
rs4557608	7	47154807	C	22	3 T	0.0001254
rs11767755	7	47155356	A	22	3 G	0.0001254
rs35325414	7	47156676	C	17	2 T	0.000467
rs62447756	7	47790222	A	5	19 C	0.0008415
rs138331667	7	47792185	T	5	19 TG	0.0008415
rs146000028	7	47794674	G	5	19 GAC	0.0008415
7:48259483	7	48259483	C	9	27 A	0.0001402
rs11238176	7	52169286	T	43	19 C	0.000761
rs12532936	7	52169566	C	43	19 T	0.000761
rs10250775	7	52169733	G	43	19 A	0.000761
rs4409354	7	52170775	T	43	19 C	0.000761
rs4458824	7	52170783	T	43	19 C	0.000761
rs4431548	7	52170869	G	43	19 A	0.000761
rs145770713	7	52170905	G	43	19 C	0.000761
rs150374058	7	52171600	A	43	19 C	0.000761
rs7808254	7	52171697	G	43	19 A	0.000761

rs2465787	7	52172110	C	43	19	T	0.000761
rs2952746	7	52172843	G	43	19	A	0.000761
rs4593482	7	52173068	G	43	19	A	0.000761
rs148576852	7	52173272	A	43	19	G	0.000761
rs114982018	7	52173431	C	43	19	T	0.000761
rs4349936	7	52174104	G	44	19	A	0.0006784
rs200757191	7	52174329	TA	43	19	T	0.000761
rs62465899	7	52175671	A	42	18	G	0.0006782
rs56383811	7	52176496	C	42	18	G	0.0006782
rs10235638	7	66396940	T	2	14	C	0.0009649
rs35558090	7	68080571	A	4	18	G	0.0009242
rs57967069	7	68081476	C	11	28	T	0.0008103
rs34518399	7	68083072	A	11	28	G	0.0008103
rs71565192	7	68083585	T	11	28	C	0.0008103
rs35952740	7	68089016	CA	25	43	C	0.0008649
rs36119273	7	68090225	T	4	22	C	0.00003217
rs4717464	7	68090345	T	4	22	C	0.00003217
rs12672169	7	68090829	C	5	22	T	0.0005146
rs35900028	7	68816739	T	4	18	C	0.0009242
rs2271032	7	68824095	G	4	18	A	0.0009242
rs34786963	7	68838684	C	4	18	T	0.0009242
rs1981586	7	68926224	G	9	26	A	0.0004896
rs1981585	7	68927887	G	9	25	A	0.0008942
rs12155091	7	68928747	G	9	26	A	0.0004896
rs115881004	7	68928894	T	4	20	C	0.0001364
rs56854903	7	68931329	A	9	25	G	0.0008942
rs71529974	7	68932680	G	9	26	GT	0.0004896
rs55714924	7	68933489	C	9	26	G	0.0004896
rs10240224	7	68934595	A	9	26	G	0.0004896
rs34117578	7	68935787	G	9	26	A	0.0004896
rs35816055	7	68935965	A	9	26	G	0.0004896
rs35611343	7	68936196	G	9	26	A	0.0004896
rs2865169	7	68936682	G	9	26	A	0.0004896
rs6975413	7	68937195	A	9	26	T	0.0004896
rs7778427	7	68939463	G	9	26	A	0.0004896
rs6954006	7	68940427	C	9	26	T	0.0004896
rs6460521	7	68941883	A	9	26	G	0.0004896
rs7810187	7	68941907	T	9	26	C	0.0004896
rs6979168	7	68942454	G	9	26	C	0.0004896
rs6944163	7	68942783	A	9	26	G	0.0004896
rs13226730	7	68943771	G	9	26	C	0.0004896

rs13243683	7	68943772	C	9	26	T	0.0004896
rs6951565	7	68944272	T	9	26	C	0.0004896
rs55651041	7	68945496	T	9	26	A	0.0004896
rs56919232	7	68945647	C	9	26	T	0.0004896
rs3944102	7	68948035	C	4	21	T	0.00006677
rs7797579	7	68948713	G	9	26	A	0.0004896
rs10233616	7	68950148	T	9	26	C	0.0004896
rs6960026	7	68950843	T	9	26	C	0.0004896
rs6979837	7	68950969	G	4	20	A	0.0001364
rs10256483	7	68951431	C	9	26	T	0.0004896
rs6460522	7	68955922	A	9	25	G	0.0008322
7:68958082	7	68958082	ATTTTAT	4	20	A	0.0001364
rs28758447	7	68958234	C	9	26	T	0.0004896
rs76969251	7	68959199	C	4	20	T	0.0001364
rs73437648	7	68959257	G	9	26	A	0.0004896
rs10257731	7	68961448	T	9	26	C	0.0004896
rs11772497	7	68962023	T	9	26	A	0.0004896
rs2865170	7	68963429	G	9	26	T	0.0004896
rs13239400	7	68965320	A	9	26	G	0.0004896
rs10278143	7	68966004	G	9	26	C	0.0004896
rs35187398	7	68968364	T	4	18	C	0.0003832
rs17137493	7	68968622	T	9	26	C	0.0004896
rs62454904	7	68968729	T	45	20	C	0.0007326
rs4268006	7	68969459	A	9	26	G	0.0004896
rs35960270	7	68970424	G	4	20	A	0.0001364
rs10950190	7	68971141	G	9	26	A	0.0004896
rs7785268	7	68974859	A	9	25	T	0.0008942
rs2108046	7	68976811	G	10	27	T	0.0006523
rs35351867	7	68977045	T	4	20	C	0.0001364
rs34824391	7	68978353	A	4	20	G	0.0001364
rs12538348	7	68982336	A	9	26	T	0.0004896
rs57286442	7	68982375	C	45	20	T	0.0007326
rs1541427	7	68983295	G	9	26	A	0.0004896
rs35241473	7	68985852	C	4	20	T	0.0001364
rs34650051	7	68986840	A	4	20	G	0.0001364
rs34201872	7	68987407	T	4	20	C	0.0001364
rs10265122	7	68991223	A	9	26	C	0.0004896
rs12531644	7	68993388	T	6	24	A	0.00008411
rs11771876	7	68993473	A	9	26	C	0.0004896
rs34387293	7	68994072	A	4	20	G	0.0001364
rs17140651	7	68995564	A	4	20	G	0.0001364

rs12698803	7	68997721	C	9	26	T	0.0004896
rs41862	7	69001467	A	9	26	G	0.0004896
rs10487947	7	69004381	A	4	20	G	0.0001364
rs12698804	7	69004448	G	9	26	A	0.0004896
rs41857	7	69008652	T	4	20	C	0.0001364
rs41854	7	69011346	T	4	19	C	0.0004818
rs41853	7	69011542	C	9	27	A	0.0002633
rs41848	7	69016574	A	4	21	T	0.00006677
rs12670658	7	69033897	T	5	20	C	0.0003688
rs6966052	7	69035699	A	44	19	G	0.0006784
rs1029811	7	69037529	T	5	21	G	0.0002243
rs2851493	7	69048815	T	4	21	G	0.00006677
rs2851520	7	69054756	C	4	21	T	0.00006677
rs1003404	7	69054960	T	4	21	C	0.00006677
rs56000336	7	75628874	T	11	29	TA	0.00087
rs10954750	7	75664921	C	37	12	G	0.0001447
rs10255591	7	75746409	C	32	10	T	0.0003432
rs55671363	7	75793405	C	32	10	T	0.0003432
rs112442379	7	75837823	C	27	7	CA	0.000799
rs111831719	7	75845457	C	29	9	A	0.0008145
rs3960554	7	75845597	T	29	9	G	0.0008145
rs78663390	7	75845837	A	29	9	C	0.0008145
rs10952869	7	75846453	G	33	10	A	0.0003125
rs6465036	7	75859296	G	34	12	A	0.000839
rs3807880	7	75864208	C	35	12	T	0.0004754
rs4727116	7	75869398	T	34	12	C	0.000839
rs10464565	7	75869526	G	35	13	T	0.000996
rs7782880	7	75871871	G	35	13	A	0.000996
rs1894761	7	75876754	C	35	13	T	0.000996
rs7777955	7	75879792	T	35	13	C	0.000996
rs11973658	7	75882026	C	35	13	A	0.000996
rs67387111	7	75883092	C	34	12	T	0.0004329
rs6978164	7	75887815	G	35	13	A	0.000996
rs6465068	7	75894946	A	35	13	G	0.000996
rs17802047	7	79632465	T	4	18	C	0.0009242
rs55836816	7	79634512	G	4	18	A	0.0009242
rs17802148	7	79636682	C	4	18	T	0.0009242
rs146184091	7	79638491	A	4	18	AC	0.0009242
rs56057150	7	79638737	T	4	18	C	0.0009242
rs62461708	7	79644665	G	4	18	A	0.0009242
rs34568037	7	79649750	C	4	18	G	0.0009242

rs55947746	7	79656124	A	4	18	G	0.0009242
rs62461712	7	79657182	T	4	18	G	0.0009242
rs2435272	7	85007066	T	23	41	G	0.0008021
rs2463468	7	85009349	T	23	41	C	0.0008021
rs2030624	7	85013958	C	23	41	T	0.0008021
rs2463475	7	85016000	T	23	41	C	0.0008021
rs2462058	7	85019297	T	23	41	A	0.0008021
rs1403785	7	85021953	A	23	41	G	0.0008021
rs2462057	7	85024037	T	23	41	C	0.0008021
rs1960550	7	85024643	T	23	41	C	0.0008021
rs2077770	7	85026097	T	23	41	A	0.0008021
rs1583081	7	85034227	T	23	41	G	0.0008021
rs2463480	7	85036389	T	23	41	C	0.0008021
rs2435264	7	85036405	T	23	41	A	0.0008021
rs148525782	7	85045118	A	23	41	AT	0.0008021
rs112461735	7	85045121	C	23	41	A	0.0008021
rs2462049	7	85099898	G	24	43	T	0.000471
rs6944685	7	85356747	T	41	16	C	0.0002964
rs12532025	7	88782746	G	19	3	A	0.0005082
rs12670534	7	93403525	G	24	42	T	0.0008352
rs2724119	7	93405685	G	30	48	A	0.0009571
rs7803982	7	93405960	T	23	42	C	0.0007426
7:96459804	7	96459804	C	0	10	A	0.0004292
rs77317590	7	98729318	T	0	9	C	0.0009641
rs10226446	7	98832759	T	1	16	C	0.00003625
rs369645786	7	98832779	T	3	21	C	0.00002955
rs6947597	7	108795622	A	30	9	G	0.0004612
rs7782912	7	108892907	C	28	8	T	0.0006092
rs38756	7	109939445	T	24	5	G	0.0003659
rs4727743	7	110332746	A	55	27	G	0.0002959
rs7802317	7	110373310	T	21	40	A	0.0006238
rs7802964	7	110373541	A	22	41	G	0.0007034
rs10246851	7	112126180	G	3	16	T	0.0009912
rs62474609	7	112126553	T	3	16	C	0.0009912
rs62474610	7	112126570	A	3	16	G	0.0009912
rs1449955	7	112628422	T	32	50	C	0.0009655
rs36091121	7	112700129	C	53	27	CCT	0.0006109
rs2950815	7	121045761	A	7	23	G	0.0005345
rs10261193	7	121246641	A	0	9	G	0.0009641
rs10236326	7	121248173	G	0	9	A	0.0009641
rs149552905	7	121251931	A	0	9	AT	0.0009641

rs10716875	7	122549304	A	25	5	AT	0.0001938
rs17145186	7	122601667	A	24	5	G	0.0003659
rs60189483	7	122603051	CT	24	5	C	0.0003659
rs7784430	7	122606768	T	24	5	C	0.0003659
rs6466846	7	122607937	A	24	5	G	0.0003659
rs719605	7	122613859	C	24	5	T	0.0003659
rs56758994	7	122619777	C	24	5	T	0.0003659
rs17145243	7	122625340	G	24	5	A	0.0003659
rs67821149	7	122628392	A	24	5	G	0.0003659
rs7785882	7	122629498	G	24	5	A	0.0003659
rs11976579	7	122630605	A	24	5	G	0.0003659
rs66848408	7	122634291	T	24	5	G	0.0003659
rs57442727	7	122638866	G	23	5	A	0.0006807
rs66998911	7	122645102	G	24	5	A	0.0003659
rs75158299	7	122647543	G	24	5	GA	0.0003659
rs2222610	7	122647812	T	24	5	A	0.0003659
rs11983656	7	122648709	T	24	5	C	0.0003659
rs67390806	7	122650589	A	24	5	G	0.0003659
rs66972046	7	122651571	C	24	5	T	0.0003659
rs55772605	7	122668336	G	24	5	T	0.0003659
rs73217748	7	122738762	G	34	12	A	0.000839
rs10953972	7	122749779	T	30	9	C	0.0004612
rs112153839	7	122787591	CCT	38	15	C	0.0007851
rs7804180	7	122790130	C	38	15	T	0.0007851
rs6953295	7	122807917	T	29	8	C	0.0003391
rs11976455	7	122808528	G	30	8	A	0.0001859
rs12706504	7	122901493	A	35	12	T	0.0004754
7:123237974	7	123237974	GTGTGTGTGTA	0	11	G	0.0002178
rs17147421	7	124383948	A	11	28	G	0.000811
rs73227503	7	124721044	G	12	31	A	0.0003217
rs12056290	7	124757562	C	15	33	T	0.0005689
rs66787780	7	124791849	A	14	32	G	0.0004925
rs67454509	7	124792500	G	14	32	A	0.0004925
rs59635547	7	124796371	C	14	32	A	0.0004925
rs68148969	7	124796408	G	14	32	C	0.0004925
rs17387591	7	124799471	C	14	32	T	0.0004925
rs67673811	7	124802694	G	14	32	C	0.0004925
rs702427	7	124802831	T	17	35	A	0.0007327
rs650030	7	124803703	G	19	37	A	0.0009069
rs648619	7	124803868	A	28	47	G	0.0005255
rs651332	7	124804423	A	28	47	G	0.0005255

rs67313231	7	124804532	A	14	33	T	0.0002703
rs61068276	7	124804887	T	23	43	C	0.0002487
rs35642992	7	124806186	GA	28	47	G	0.0005255
rs1728929	7	124808930	A	28	47	G	0.0005255
rs1728930	7	124809310	A	28	47	G	0.0005255
rs2968865	7	124810399	A	28	47	G	0.0005255
rs1991409	7	124923489	C	11	28	G	0.0008103
rs4728020	7	124926770	T	11	28	C	0.0008103
rs28645171	7	124927862	T	17	35	C	0.0007327
rs9784927	7	124928978	A	17	36	C	0.0004114
rs34927113	7	133325624	TC	50	24	T	0.0007955
rs73152980	7	133464988	A	2	15	C	0.0005115
rs4236486	7	139992324	T	39	13	C	0.00009633
rs4236487	7	139992334	A	39	13	G	0.00009633
rs4726753	7	139992554	T	39	12	C	0.00004106
rs62491836	7	139994133	A	39	12	G	0.00004106
rs79337537	7	140818880	G	15	1	A	0.0003561
rs58091278	7	140820604	A	15	1	AT	0.0003561
7:142216423	7	142216423	T	17	2	TCCAGC	0.0009658
7:142216431	7	142216431	T	16	2	C	0.0008912
7:142216433	7	142216433	GTTA	16	2	G	0.0008912
rs17798068	7	144877432	T	28	8	C	0.0006092
rs13234300	7	144882368	A	25	4	G	0.00005893
rs10228815	7	144882889	G	23	4	T	0.0001164
rs71528967	7	144884334	A	24	4	G	0.0001159
rs7799776	7	144888698	T	24	4	C	0.0001159
rs7788595	7	144893242	A	24	4	C	0.0001159
rs34694182	7	144893767	A	25	6	AT	0.0005533
rs2371843	7	144895554	A	21	3	G	0.0002475
rs2371844	7	144895575	A	21	3	G	0.0002475
rs12703663	7	144895979	G	21	3	A	0.0002475
rs13247774	7	144896721	A	21	3	G	0.0002475
rs150195176	7	144911246	T	21	3	C	0.0002475
rs12703667	7	144915712	T	21	4	C	0.0008104
rs12703668	7	144938998	C	20	3	T	0.000269
rs66706284	7	144946452	A	21	3	G	0.0002475
rs73170827	7	144947604	T	16	2	A	0.0008956
rs34223395	7	144950375	T	21	3	C	0.0002475
rs12703672	7	144965537	C	22	3	A	0.0001254
rs77056733	7	150961048	C	15	0	T	0.00003963
rs75368642	7	150969808	T	15	0	C	0.00003963

rs7796815	7	152731524	G	5	19	T	0.0008415
rs11768300	7	152731623	G	5	19	C	0.0008415
rs11772024	7	152731636	C	5	19	T	0.0008415
rs10262292	7	152732059	T	5	19	C	0.0008415
rs6967853	7	152737574	C	4	18	T	0.0009242
rs6967855	7	152737578	C	4	18	T	0.0009242
rs954360	7	152737659	T	4	18	C	0.0009242
rs3898061	7	152737701	A	4	18	G	0.0009242
rs954359	7	152737733	C	4	18	T	0.0009242
rs11388703	7	152738308	T	4	18	TG	0.0009242
rs3111875	7	154975699	A	13	30	G	0.0007578
rs13223976	7	154976046	C	29	48	T	0.0005332
rs6597471	7	154978788	G	30	48	C	0.0009571
rs6597473	7	154978979	A	30	48	G	0.0009571
7:154979133	7	154979133	C	30	48	CTT	0.0009571
7:154979135	7	154979135	C	30	48	CTGCCTCATTGG	0.0009571
rs4960695	7	154979628	A	30	48	C	0.0009571
rs4960696	7	154979643	C	30	48	T	0.0009571
rs7794634	7	154980152	T	29	48	C	0.0005332
rs7804702	7	154980153	G	30	48	A	0.0009571
rs7790169	7	154980168	A	30	48	G	0.0009571
rs7790310	7	154980243	A	30	48	G	0.0009571
rs10279840	7	154980616	A	29	48	C	0.0005332
rs10274085	7	154980631	C	30	48	T	0.0009571
rs35191725	7	154980990	T	30	48	C	0.0009571
rs11975494	7	154981034	G	30	48	C	0.0009571
rs4273809	7	154981157	A	30	48	G	0.0009571
rs1465783	7	154981313	A	30	48	T	0.0009571
rs1465784	7	154981331	G	30	48	A	0.0009571
rs11243341	7	154981454	T	30	48	C	0.0009571
rs6597474	7	154981756	T	30	48	G	0.0009571
rs6597475	7	154981760	G	30	48	A	0.0009571
rs4620236	7	154986897	T	8	25	C	0.0003633
rs11762624	7	154990360	T	8	25	C	0.0003633
rs34684769	7	154991646	A	8	25	G	0.0003633
rs2873382	7	154994122	G	8	25	C	0.0003633
rs11773201	7	154994653	A	8	25	G	0.0003633
rs11773280	7	154994990	A	8	25	G	0.0003633
rs12719690	7	155009761	A	27	45	G	0.0009136
rs6943455	7	155011946	C	31	51	T	0.0002959
rs1124175	7	155015124	T	7	23	G	0.0004919

rs4134496	7	155019285	A	3	16	G	0.0009912
rs12666834	7	155019546	A	8	26	C	0.0001919
rs7007130	8	1762754	C	30	48	G	0.0009571
rs4308779	8	1763227	G	30	48	A	0.0009571
8:1764026	8	1764026	GAAAGAAAGA	0	10	G	0.0003694
rs113348912	8	2573525	TTCTTG	13	30	T	0.0007578
rs6993235	8	2587648	G	41	16	A	0.0002964
rs17755518	8	2596106	T	37	14	G	0.0006739
rs7831554	8	2597843	G	38	15	A	0.0007851
rs4876049	8	2693627	C	15	33	T	0.0005689
rs17078672	8	2697280	G	13	32	A	0.0002287
rs75062583	8	2708370	C	1	12	T	0.0007887
8:3040116	8	3040116	A	0	10	G	0.0003159
8:3040118	8	3040118	GTGTA	0	10	G	0.0003694
8:3040126	8	3040126	A	0	10	G	0.0003694
rs1154063	8	4301904	A	2	15	G	0.000564
rs77354697	8	4311101	A	5	19	T	0.0008415
rs113568212	8	4358101	G	2	14	A	0.0009649
rs11985944	8	6021491	T	26	5	G	0.0001012
rs2570665	8	6031822	G	28	8	A	0.0006092
rs113660596	8	6323448	G	6	24	C	0.00008935
8:6323452	8	6323452	C	6	24	CCATCCATCCATC	0.00008935
8:6323456	8	6323456	C	6	23	CCATCCATCCATG	0.0001763
8:6323462	8	6323462	C	6	23	A	0.0001763
rs12550299	8	6325442	C	3	16	G	0.0009912
rs12545613	8	6325499	T	3	16	C	0.0009912
rs2442497	8	6326748	C	3	16	T	0.0009912
rs7833481	8	6327065	G	3	16	A	0.0009912
rs113529100	8	6327892	C	3	16	T	0.0009912
rs2440397	8	6328393	C	3	16	G	0.0009912
rs6989935	8	6328408	T	3	16	C	0.0009912
rs57804372	8	6328848	C	3	16	T	0.0009912
rs56073811	8	6328853	C	3	16	A	0.0009912
rs2440396	8	6329014	C	3	16	T	0.0009912
rs2442493	8	6329304	C	3	16	T	0.0009912
rs2442491	8	6329579	G	3	16	A	0.0009912
rs11137029	8	6330335	A	3	16	G	0.0009912
rs12676907	8	6330447	A	3	16	G	0.0009912
rs12681195	8	6330510	A	3	16	C	0.0009912
rs2440395	8	6330513	T	3	16	A	0.0009912
rs12676922	8	6330580	A	3	16	G	0.0009912

rs12675930	8	6330773	C	3	16	T	0.0009912
rs112705156	8	6331079	C	3	16	G	0.0009912
rs17077056	8	6331178	C	3	16	T	0.0009912
rs60915478	8	6331612	A	3	16	G	0.0009912
rs73196802	8	6333446	C	3	16	G	0.0009912
rs17077067	8	6334053	G	3	16	A	0.0009912
rs59635757	8	6334174	G	3	16	A	0.0009912
rs11137033	8	6334619	C	3	16	T	0.0009912
rs13281793	8	6337792	T	3	17	C	0.0005045
rs13282116	8	6337938	G	3	17	C	0.0005045
rs13248420	8	6337999	G	3	17	A	0.0005045
rs12674488	8	6338306	A	3	17	C	0.0005045
rs12677501	8	6338664	G	3	20	T	0.00006125
rs17613024	8	6338953	A	3	17	G	0.0005045
rs17077109	8	6339343	C	3	20	T	0.00006125
rs13267816	8	6340205	G	2	20	C	0.00001141
rs12679619	8	6340475	A	2	17	G	0.000111
rs12680048	8	6340660	A	2	17	G	0.000111
rs34635086	8	6341293	G	3	17	A	0.0005045
rs373003367	8	6341378	C	3	17	CAAAGA	0.0005045
rs34009442	8	6341569	C	3	17	T	0.0005045
rs60127657	8	6341897	GA	3	17	G	0.0005045
rs35150903	8	6342671	T	2	17	C	0.000111
rs34754537	8	6343123	TC	3	17	T	0.0005045
rs13281967	8	6347007	C	3	17	G	0.0005045
rs13282022	8	6347058	C	3	17	G	0.0005045
rs13282577	8	6347219	A	3	17	G	0.0005045
rs12681009	8	6350873	G	3	17	A	0.0005045
rs2442606	8	6387923	A	6	21	G	0.0006518
rs201428357	8	11871062	T	0	9	C	0.0009641
rs17222409	8	13385822	T	37	14	G	0.0006739
8:13730686	8	13730686	T	30	7	TA	0.0000559
8:13730689	8	13730689	G	35	8	A	0.00001201
8:13730692	8	13730692	TAA	30	7	T	0.0000559
8:13730707	8	13730707	T	30	5	A	0.000005352
8:13730713	8	13730713	A	26	5	ATATATATATATA	0.00009054
8:13730715	8	13730715	A	26	4	ATATATATATATA	0.00001482
rs7823636	8	14004973	C	22	41	A	0.0004309
rs9325689	8	14023396	G	22	41	C	0.0004309
rs34959295	8	15361577	A	14	31	G	0.0008795
rs72207091	8	18681288	A	9	26	ACT	0.0004896

rs4294199	8	19757645	T	8	24	C	0.0006758
rs11779530	8	19758215	T	8	24	G	0.0006758
rs9650414	8	25954874	C	14	1	T	0.0007094
rs73231554	8	26659518	T	0	10	C	0.0004292
rs73231562	8	26671066	T	0	9	C	0.0009641
rs73231563	8	26671162	G	0	9	A	0.0009641
rs55648206	8	26675004	A	0	9	T	0.0009641
rs55858769	8	26675245	G	0	9	T	0.0009641
rs73231575	8	26679581	G	0	9	T	0.0009641
rs17334323	8	26688826	A	48	23	G	0.0008881
rs113277653	8	27767946	T	0	9	C	0.0009641
8:28135959	8	28135959	A	12	0	G	0.0004093
rs3031241	8	32280128	TAAACA	18	3	T	0.0009486
rs10503914	8	32280827	C	18	3	T	0.0009486
rs73234126	8	32399663	A	18	3	G	0.0009486
8:32405112	8	32405112	C	1	12	CA	0.0007887
8:32405114	8	32405114	C	1	12	CACACACACACAC	0.0007887
rs12542432	8	40993135	T	24	5	C	0.0003659
rs61709546	8	42712987	C	13	0	G	0.0001783
rs72647289	8	42964349	T	13	0	C	0.0001783
rs59037494	8	42997744	T	13	0	C	0.0001783
8:43835929	8	43835929	A	20	3	T	0.000269
8:46840754	8	46840754	C	0	9	G	0.0009641
rs7464406	8	55407591	A	18	36	G	0.0008188
rs1072737	8	55414324	G	21	40	T	0.0006238
rs5891536	8	55417984	TA	21	40	T	0.0006238
rs17400452	8	55421102	C	21	40	A	0.0006238
rs34231092	8	55421139	T	21	40	A	0.0006238
rs17400501	8	55421226	G	21	40	A	0.0006238
rs17309332	8	55421388	A	21	40	T	0.0006238
rs17400569	8	55421420	C	21	40	T	0.0006238
rs17309381	8	55421510	A	21	40	C	0.0006238
rs10641771	8	55422635	CAAA	21	40	C	0.0006238
rs7819146	8	55423384	A	21	40	G	0.0006238
rs6473939	8	55423932	C	21	40	A	0.0006238
rs6473940	8	55424053	C	21	40	T	0.0006238
rs11782075	8	55434007	C	21	40	T	0.0006238
rs13251118	8	55442498	A	21	40	G	0.0006238
rs13266258	8	55444202	G	21	40	A	0.0006238
rs71554168	8	55445687	G	25	43	GA	0.0008649
rs13281003	8	55450271	C	21	40	T	0.0006238

rs57780081	8	55453219	T	21	40	C	0.0006238
rs66772979	8	55453589	A	20	42	G	0.00009387
rs4738971	8	55453592	T	25	45	G	0.0004551
rs6997005	8	55454506	A	21	40	G	0.0006238
rs62516550	8	55467028	T	18	38	C	0.0002562
rs34155635	8	55467747	CTT	17	38	C	0.0002126
rs117033707	8	55603134	C	0	10	G	0.0004292
rs75736542	8	55670027	T	0	9	C	0.0009641
rs144337474	8	55716390	C	0	10	G	0.0004292
rs72645681	8	56363436	T	11	0	C	0.0007855
rs62517408	8	56397088	G	11	0	A	0.0007855
rs2439727	8	57709572	G	14	31	C	0.0008795
rs2439726	8	57709999	T	14	31	C	0.0008795
rs2439725	8	57717891	T	14	31	G	0.0008795
rs2911710	8	57718944	A	14	31	G	0.0008795
rs201700604	8	57721064	C	14	31	A	0.0008795
rs140326078	8	57722202	T	14	31	C	0.0008795
rs145468278	8	57723186	T	14	31	G	0.0008795
rs149384686	8	57724174	A	14	32	G	0.0004925
rs7817180	8	66206231	T	21	4	C	0.0008104
rs10957338	8	66212413	T	21	4	C	0.0008104
8:66217376	8	66217376	AC	30	8	A	0.0001859
rs10107515	8	66218313	A	21	4	G	0.0008104
rs11426900	8	66221027	GT	23	5	G	0.0006807
rs12546368	8	66237960	A	23	5	G	0.0006807
8:66332579	8	66332579	A	12	0	G	0.0003752
rs34207507	8	67552947	CG	29	51	C	0.00008171
rs36069899	8	68438324	A	13	0	G	0.0001783
rs10957390	8	68457397	G	29	8	A	0.0003391
rs10957391	8	68458267	C	28	8	T	0.0006092
rs4737239	8	68460852	A	29	8	G	0.0003391
rs2380415	8	68462201	T	28	8	C	0.0006092
rs2380416	8	68462304	A	28	8	G	0.0006092
rs2380417	8	68462501	G	29	8	C	0.0003391
rs11785565	8	68462679	T	29	8	C	0.0003391
rs10808755	8	68516681	T	47	19	G	0.0001149
rs11781284	8	68517124	T	47	19	C	0.0001149
rs7002461	8	68517827	A	43	18	T	0.0003235
rs143489719	8	68518322	A	36	13	AAAAC	0.0005704
rs7836691	8	68518472	C	44	18	T	0.0002208
rs7818863	8	68518795	T	47	19	C	0.0001149

rs2128105	8	68519685	C	39	14	T	0.0002137
rs12680940	8	68522138	G	35	13	T	0.000996
rs610372	8	68523186	A	36	13	G	0.0005704
rs11782071	8	68523405	A	23	41	C	0.0008021
rs408802	8	68523594	T	47	19	C	0.0001149
rs7814309	8	68525283	T	36	13	C	0.0005704
rs7832751	8	68525425	C	38	14	T	0.0003829
rs2086540	8	68525931	A	36	13	C	0.0005704
rs379025	8	68526546	T	47	19	G	0.0001149
rs421352	8	68526582	G	35	13	A	0.000996
rs7004943	8	68998892	A	29	9	G	0.0008145
rs12677746	8	70908932	T	25	6	G	0.0005533
rs59764656	8	70915304	G	25	6	C	0.0005533
rs61043439	8	70915364	A	25	6	AT	0.0005533
rs62533361	8	70917328	A	25	6	G	0.0005533
rs62533365	8	70926751	C	25	6	G	0.0005533
rs7017473	8	72000474	A	49	24	T	0.000936
rs66664867	8	75356443	A	11	0	ACGCGCG	0.0007855
rs72672726	8	78915051	T	19	36	C	0.0008669
rs147963364	8	78939322	AT	19	37	A	0.0009069
rs79112138	8	79833517	A	2	17	G	0.000111
rs34445833	8	80770915	C	49	23	G	0.0005083
rs13279712	8	80771340	A	40	16	G	0.0005221
rs10098536	8	80771378	C	25	48	T	0.00003878
rs11986142	8	80772791	C	45	20	T	0.0007326
rs79107621	8	80772962	C	29	51	A	0.00008171
rs77652490	8	80774159	C	46	20	T	0.0004155
rs1966859	8	80776483	A	28	46	G	0.0009321
rs10958009	8	82239312	C	18	3	G	0.0009486
rs116877427	8	91064745	A	0	9	G	0.0009641
8:98448334	8	98448334	A	2	14	ATTTTAT	0.0009649
rs7828927	8	99123614	C	18	3	T	0.0009486
rs141450399	8	101232421	T	5	20	TCAGTACTG	0.0004383
rs17336058	8	101242613	T	5	20	C	0.0004383
rs17421246	8	101262063	G	5	20	A	0.0004383
rs145170162	8	101269045	C	5	20	CA	0.0004383
rs17422137	8	101297726	G	5	20	A	0.0004383
rs62514974	8	101306401	A	5	20	T	0.0004383
rs17337916	8	101317666	A	5	20	G	0.0004383
rs56356271	8	101322458	T	5	20	C	0.0004383
rs59885259	8	101345308	G	5	19	GA	0.0008415

8:102664587	8	102664587	GA	1	12	G	0.0009476
8:103535019	8	103535019	C	41	15	T	0.0001402
rs405634	8	108648739	A	34	11	G	0.0003893
rs138007099	8	115585034	CTTTACTTCCT	14	1	C	0.0007094
rs138795202	8	115585723	A	14	1	ACAC	0.0007094
rs56358453	8	115585748	G	14	1	C	0.0007094
rs12682243	8	115589058	A	14	1	G	0.0007094
rs4278177	8	115589089	A	14	1	C	0.0007094
rs1390802	8	115589367	C	14	1	T	0.0007094
rs11987607	8	115589433	C	14	1	T	0.0007094
rs112972565	8	115592838	T	14	1	C	0.0007094
rs188331229	8	115593078	G	14	1	T	0.0007094
8:115595287	8	115595287	G	14	1	C	0.0007094
rs144558766	8	115595780	T	14	1	C	0.0007094
rs368858621	8	115595883	T	14	0	C	0.00008428
8:115596736	8	115596736	A	14	1	G	0.0007094
rs4360323	8	115597453	G	14	1	T	0.0007094
rs189798615	8	115597550	A	14	1	G	0.0007094
rs4375035	8	115597614	T	14	1	G	0.0007094
rs113058397	8	115598479	T	14	1	C	0.0007094
rs113913892	8	115598520	A	14	1	T	0.0007094
rs72688152	8	115598609	C	14	1	T	0.0007094
rs140621	8	115601414	T	14	1	A	0.0007094
rs140623	8	115601454	T	14	1	C	0.0007094
rs140624	8	115601470	C	14	1	G	0.0007094
rs72688155	8	115603416	A	14	1	G	0.0007094
rs72688157	8	115605160	C	14	1	A	0.0007094
rs72688158	8	115606114	C	14	1	G	0.0007094
rs72688160	8	115606270	G	14	1	A	0.0007094
rs72688161	8	115607364	T	14	1	C	0.0007094
rs72688163	8	115607738	T	14	1	A	0.0007094
rs72688165	8	115609100	C	14	1	T	0.0007094
rs61119861	8	115609854	T	14	1	C	0.0007094
rs61642028	8	115609982	C	14	1	T	0.0007094
rs111607481	8	115610153	G	14	1	GATTTT	0.0007094
rs11436316	8	115611394	AC	14	1	A	0.0007094
rs149860162	8	115611534	A	14	1	AAATT	0.0007094
rs11436317	8	115611558	CA	14	1	C	0.0007094
rs1010296	8	115612916	G	14	1	A	0.0007094
rs59874151	8	115613474	G	14	1	A	0.0007094
rs141271051	8	115614789	G	14	1	GA	0.0007094

rs1390805	8	115615140	C	14	1	A	0.0007094
rs10505224	8	115615472	G	14	1	C	0.0006479
rs72688171	8	115617154	A	14	1	G	0.0007094
rs6993330	8	115618369	G	14	1	A	0.0007094
rs990064	8	115620846	A	14	1	G	0.0007094
rs72688174	8	115621273	T	14	1	G	0.0007094
rs67259516	8	115622714	A	14	1	G	0.0007094
rs11987094	8	115623858	G	14	1	T	0.0007094
rs113317677	8	115624633	GA	14	1	G	0.0007094
rs56744618	8	115625115	G	14	1	A	0.0007094
rs10568390	8	115625262	A	14	1	ATG	0.0007094
rs716319	8	115626526	G	14	1	A	0.0007094
rs72688177	8	115628267	A	14	1	G	0.0007094
rs11989617	8	115630616	A	14	1	G	0.0007094
rs12675391	8	115632043	T	14	1	C	0.0007094
rs1390806	8	115635282	A	14	1	G	0.0006479
rs148024664	8	115637496	G	15	1	GC	0.0003205
rs72688180	8	115638469	C	15	1	G	0.0003205
rs72688182	8	115641954	T	14	1	C	0.0006479
rs201102203	8	115644084	A	14	1	C	0.0006479
rs140178196	8	115644994	T	14	1	C	0.0006479
8:115645784	8	115645784	T	16	1	G	0.0001286
rs150137951	8	115648635	T	14	1	C	0.0007094
rs142867399	8	115648840	T	14	1	G	0.0007094
rs75777265	8	115649722	C	14	1	T	0.0007094
rs72688185	8	115650891	G	14	1	A	0.0007094
rs182353197	8	115652354	A	14	1	T	0.0007094
rs75910793	8	115652678	G	14	1	A	0.0007094
rs11996788	8	115653663	G	14	1	C	0.0007094
rs931614	8	115653998	A	14	1	G	0.0007094
rs1496752	8	115654884	T	14	1	C	0.0007094
rs72688196	8	115663941	G	14	1	C	0.0007094
rs200567012	8	115666287	T	14	1	G	0.0007094
8:115667919	8	115667919	A	14	1	ATTTTTT	0.0008899
rs72688199	8	115670389	T	14	1	G	0.0007094
rs56335213	8	115675219	A	14	1	C	0.0007094
rs7845200	8	115698181	G	17	2	A	0.000467
rs28445023	8	115701338	T	18	2	C	0.0002408
rs28505858	8	115707801	T	18	2	C	0.0002408
rs7827613	8	115712241	G	18	2	A	0.0002408
rs201598000	8	115713723	C	17	2	G	0.000959

8:115713727	8	115713727	C	14	1	G	0.0007094
8:115714930	8	115714930	T	14	1	TA	0.0007094
rs10109368	8	115715757	C	18	2	T	0.0002408
rs71886645	8	115717208	T	18	2	TAATA	0.0002408
rs10100267	8	115721881	T	18	2	C	0.0002408
rs3062473	8	115731072	TTAG	18	2	T	0.0002408
rs35150135	8	115731550	T	18	3	C	0.0009486
rs7826937	8	129264171	C	17	37	T	0.0002264
rs4733850	8	129264260	A	17	37	G	0.0002264
rs4733851	8	129264420	A	23	42	G	0.0004519
rs2142199	8	129266826	T	26	44	C	0.0008911
rs4733601	8	129269466	A	26	44	G	0.0008911
rs1120501	8	129274183	A	26	45	T	0.0005028
rs2702973	8	134090891	A	27	45	G	0.0009136
rs2739166	8	134091284	T	27	45	C	0.0009136
rs2702974	8	134092210	C	27	45	T	0.0009136
rs2702979	8	134093965	C	27	45	T	0.0009136
rs2739171	8	134098665	A	27	45	G	0.0009136
rs13256826	8	134116227	T	23	43	C	0.0002487
rs4736434	8	134121121	T	23	43	C	0.0002487
rs73365346	8	135605357	A	3	17	G	0.0005045
rs2668112	8	138939408	C	31	49	T	0.0009634
rs6578129	8	141656890	G	21	40	A	0.0006238
rs938724	8	141657133	G	21	40	A	0.0006238
rs6997840	8	141658361	T	20	40	C	0.0003188
rs4507813	8	141796344	T	26	44	C	0.0008911
rs59704417	8	142574115	T	5	19	C	0.0008415
rs7042540	9	343859	C	17	37	T	0.0002264
rs7032705	9	343861	G	14	31	C	0.0008795
rs750586	9	362749	G	7	23	A	0.0004919
rs10814269	9	366911	A	9	26	G	0.0004896
rs74343447	9	730460	A	15	36	G	0.00009076
rs10758864	9	743353	T	19	38	C	0.0005156
rs7033398	9	2303184	A	48	23	C	0.0008881
9:4806593	9	4806593	T	1	12	C	0.0007887
rs7027864	9	6654713	G	3	16	T	0.0009912
rs71330108	9	6661368	GA	2	15	G	0.0004755
9:7646055	9	7646055	G	16	2	A	0.0008956
rs12555779	9	8798076	T	39	16	A	0.0009032
rs10283912	9	12285170	C	53	26	T	0.0003309
rs1389739	9	14809650	C	2	14	T	0.0009649

rs1389738	9	14809826	C	2	14	T	0.0009649
rs35954375	9	14827173	CAGA	2	15	C	0.0004755
rs2483055	9	14834542	A	2	14	G	0.0009649
rs34699275	9	14834902	G	2	14	GT	0.0009649
rs1855138	9	14835141	C	2	14	G	0.0009649
rs10810790	9	17458872	C	9	25	T	0.0008942
rs10811484	9	21158515	G	43	19	C	0.000761
rs4568676	9	21184475	G	42	18	C	0.0006782
9:27989449	9	27989449	C	25	5	G	0.0001938
9:27989455	9	27989455	C	22	3	G	0.0001254
9:30709794	9	30709794	A	16	2	AC	0.0008037
rs17313768	9	32844139	G	27	45	A	0.0009136
rs1831038	9	32849413	T	55	28	C	0.0005409
rs199710298	9	33345167	GAAAAGA	40	17	G	0.0009663
rs200778528	9	39068726	G	33	11	A	0.0003674
rs201996957	9	39071703	C	38	14	T	0.0003829
rs201890411	9	39074383	C	39	12	T	0.00004106
rs185321727	9	39074414	C	37	14	A	0.0006739
rs62568849	9	39074909	T	39	14	G	0.0002137
rs1758483	9	39077411	G	38	14	A	0.0003829
rs7046178	9	39078975	G	38	13	C	0.0001774
rs201581639	9	39079350	T	39	14	G	0.0002137
rs62569964	9	39085463	G	34	7	A	0.000004686
rs6476770	9	39087785	A	39	14	C	0.0002137
rs1758516	9	39096642	T	38	14	C	0.0003829
rs1855006	9	39097179	C	40	14	A	0.0001172
rs4961901	9	39098698	A	39	14	G	0.0002137
rs6476771	9	39100338	T	39	14	C	0.0002137
rs10814795	9	39105379	G	47	22	A	0.0008379
9:40030023	9	40030023	C	12	0	A	0.0004093
rs28572375	9	43702852	A	5	21	C	0.0002135
rs117392740	9	45375209	A	2	18	G	0.00005262
9:66460369	9	66460369	G	0	9	T	0.0009641
rs140864612	9	68387193	T	11	0	C	0.0007855
9:69045346	9	69045346	T	2	17	C	0.000111
rs200037816	9	69045348	G	6	21	A	0.0006518
rs200760575	9	69152028	A	5	21	G	0.0002243
rs200315439	9	69271352	T	2	15	C	0.0004755
rs4571790	9	75833403	A	12	0	C	0.0003752
rs35750820	9	75835437	CTT	15	1	C	0.0003561
rs4581122	9	75836057	A	15	1	C	0.0003561

rs9657735	9	75838758	T	15	1	C	0.0003561
rs9657736	9	75840034	G	15	1	A	0.0003561
rs28796899	9	75841710	T	17	2	A	0.0004125
rs28833833	9	75841713	A	15	1	T	0.0003205
rs4366143	9	75843433	T	15	1	C	0.0003561
rs6560325	9	75844545	T	15	1	C	0.0003561
rs7863612	9	75845098	T	15	1	G	0.0003561
rs4495499	9	75845497	T	15	1	C	0.0003561
rs4745226	9	75846009	A	15	1	T	0.0003561
rs4510925	9	75846595	G	15	1	T	0.0003561
rs5898293	9	75847204	G	15	1	GA	0.0003561
rs4400456	9	75857034	T	17	2	C	0.000467
rs67538661	9	75858583	T	17	2	TA	0.000467
rs4276752	9	75860197	C	17	2	T	0.000467
rs4282636	9	75906317	A	17	0	C	0.000008611
rs4474087	9	75906822	A	17	0	G	0.000008611
rs10869248	9	75907820	A	15	0	C	0.00003963
rs11143561	9	75926372	T	17	0	C	0.000008611
rs1414159	9	75932545	C	17	0	A	0.000008611
rs35789695	9	75943778	C	17	0	CTCTT	0.000008611
rs1335387	9	75974289	G	17	0	A	0.000008611
rs6560339	9	76001473	A	17	0	G	0.000008611
rs2013971	9	76010849	C	17	0	A	0.000008611
rs1414161	9	76025984	G	17	0	A	0.000008611
rs1034264	9	76027946	T	17	0	C	0.000008611
rs280564	9	76052211	G	17	0	T	0.000008611
rs280573	9	76074106	C	21	3	T	0.0002475
rs2484348	9	76078030	T	14	0	G	0.00008428
rs375914	9	76085223	G	14	0	T	0.00008428
rs415546	9	76085262	C	18	3	T	0.0009486
rs278747	9	76089602	A	18	3	G	0.0009486
rs278742	9	76094975	A	14	0	T	0.00008428
rs278741	9	76095696	T	18	3	G	0.0009486
rs278738	9	76098252	A	14	0	C	0.00008428
rs278733	9	76110042	T	14	0	C	0.00008428
rs383182	9	76115818	C	14	0	T	0.00008428
rs278763	9	76127267	A	14	0	G	0.00008428
rs166767	9	76137194	C	14	0	T	0.00008428
rs278753	9	76141288	G	14	0	A	0.00008428
rs2492826	9	76150936	A	14	0	T	0.00008428
rs278711	9	76154116	A	14	0	G	0.00008428

rs278731	9	76162393	T	14	0	C	0.00008428
rs382417	9	76164453	G	14	0	A	0.00008428
rs451914	9	76164728	G	14	0	A	0.00008428
rs275530	9	76174599	G	14	0	A	0.00008428
rs157119	9	76182983	G	14	0	C	0.00008428
rs4745472	9	78547238	A	0	9	G	0.0009641
rs79217522	9	78549195	T	0	9	C	0.0009641
rs77748422	9	78550364	A	0	9	G	0.0009641
rs75091696	9	78550427	A	0	9	G	0.0009641
rs111599823	9	78552206	C	0	9	T	0.0009641
rs55889015	9	78552938	C	0	9	T	0.0009641
rs80035155	9	78556053	A	0	9	G	0.0009641
rs56041801	9	78556371	G	0	9	A	0.0009641
rs55819351	9	78557643	C	0	9	T	0.0009641
rs112424082	9	78558168	T	0	9	C	0.0009641
rs75747130	9	78559673	T	0	9	A	0.0009641
rs12553234	9	78560721	A	0	9	G	0.0009641
rs112901856	9	78562429	A	0	9	C	0.0009641
rs79019815	9	78562632	G	0	9	A	0.0009641
rs4745476	9	78565973	T	0	9	C	0.0009641
rs4745477	9	78566447	C	0	9	T	0.0009641
rs373589527	9	78566687	A	0	9	AAAC	0.0009641
rs7875186	9	81253945	C	18	36	T	0.0008188
rs2106804	9	81254538	C	18	36	T	0.0008188
rs9657722	9	81267990	A	18	37	T	0.0004627
rs7864005	9	81268849	G	18	36	T	0.0008188
rs2225192	9	81932941	C	35	12	T	0.0004754
rs12685026	9	81939372	T	34	12	G	0.000839
rs971986	9	81952643	C	33	11	T	0.0003674
rs1329559	9	82811242	G	16	34	T	0.0006492
rs36054026	9	85810319	A	30	6	C	0.00002254
rs10739960	9	96622388	G	23	41	T	0.0008021
rs4428725	9	96623453	G	23	41	T	0.0008021
rs4744295	9	96623821	G	23	41	A	0.0008021
rs4075732	9	96624526	C	23	41	A	0.0008021
rs10821241	9	96626249	A	23	41	G	0.0008021
rs2066474	9	99064425	C	10	28	T	0.0003491
rs1889266	9	101724686	C	17	35	G	0.0007327
rs2151616	9	101729103	A	9	25	G	0.0008942
rs882728	9	101730236	G	8	24	A	0.0006758
rs882221	9	101731087	A	9	25	G	0.0008942

rs60355315	9	101731421	T	9	25	A	0.0008942
rs2791337	9	102741443	C	50	25	T	0.000784
rs72731390	9	102824872	C	1	12	T	0.0007887
rs11333350	9	102911331	C	50	25	CA	0.0005324
rs138812004	9	102984216	G	1	12	GA	0.0007887
rs2812301	9	108208066	A	16	34	G	0.0006492
rs59463718	9	108208647	C	16	34	CTTAT	0.0006492
rs62575093	9	108209438	G	15	34	C	0.0003148
rs2771038	9	108219091	A	35	13	G	0.000996
rs2771039	9	108221252	A	16	34	G	0.0006492
rs2812306	9	108221369	A	16	34	T	0.0006492
rs62575095	9	108224124	A	16	34	G	0.0006492
rs12686557	9	108225290	A	46	20	G	0.0004155
rs145768242	9	108228282	A	16	34	ACT	0.0006492
rs2771044	9	108242839	G	16	34	C	0.0006492
rs139942055	9	108253522	T	45	20	C	0.0007326
rs7865377	9	108256602	A	16	34	G	0.0006492
rs7865160	9	108256610	T	16	34	C	0.0006492
rs2225246	9	108263907	A	36	14	G	0.0006638
rs10820812	9	108274931	A	45	20	G	0.0007326
rs7357782	9	108281208	A	47	21	G	0.0004473
rs7357632	9	108282855	A	45	20	G	0.0007326
rs62575103	9	108284699	A	16	34	G	0.0006492
rs62575104	9	108290049	T	16	34	C	0.0006492
rs74442717	9	108291374	T	16	34	G	0.0006492
rs2768279	9	108293223	T	46	21	A	0.0007859
rs59803965	9	108294017	T	45	20	TAG	0.0007326
rs57432946	9	108305782	C	16	34	G	0.0006492
rs149664708	9	108309573	TGTGTTTG	16	34	T	0.0006492
rs16403	9	108314485	A	47	21	AAC	0.0004473
rs4742651	9	108315424	T	16	34	C	0.0006492
rs62575112	9	108328228	A	16	34	G	0.0006492
rs4742653	9	108328588	C	16	34	A	0.0006492
rs76375684	9	108329464	A	16	34	C	0.0006492
rs7024265	9	108331234	A	16	34	G	0.0006492
rs4742953	9	108335958	C	16	34	G	0.0006492
rs62575116	9	108339846	C	16	34	A	0.0006492
rs147408591	9	108341274	G	16	34	A	0.0006492
rs35238231	9	108344560	A	48	23	AC	0.0008881
rs17309137	9	108348138	A	16	34	G	0.0006492
rs17309319	9	108350328	A	16	34	T	0.0006492

rs7037972	9	108351816	T	16	34	A	0.0006492
rs59750891	9	108353179	A	16	34	AC	0.0006492
rs2518124	9	108354230	C	46	20	T	0.0004155
rs1960023	9	108355222	T	46	20	C	0.0004155
rs4742955	9	108377097	A	17	35	G	0.0007327
rs11399147	9	108394700	TA	20	38	T	0.0009963
rs10978580	9	109410929	T	5	19	C	0.0008415
rs10816411	9	109411613	T	5	19	C	0.0008415
rs7859930	9	109412031	T	5	19	A	0.0008415
rs10816412	9	109412764	T	5	19	G	0.0008415
rs10816413	9	109413318	A	5	21	G	0.0002243
rs74751740	9	112059930	A	11	0	G	0.0007855
rs10981732	9	116087110	A	27	7	G	0.0004381
9:116087112	9	116087112	A	29	9	G	0.0008145
rs3810928	9	116815777	T	53	25	C	0.000174
rs141372136	9	116852266	A	52	26	AGTG	0.0005884
rs145837769	9	117007494	GGT	2	16	G	0.0002494
rs2029522	9	117009953	A	6	21	G	0.0006518
rs4979360	9	117010272	G	6	21	A	0.0006518
rs10982123	9	117011093	G	3	17	A	0.0005045
9:120060987	9	120060987	GTA	5	22	G	0.0001129
rs80255914	9	122018796	G	0	9	C	0.0009641
rs116127727	9	128190759	T	1	15	A	0.00007965
rs12552483	9	128215861	A	1	13	G	0.0003716
9:134920806	9	134920806	A	5	19	C	0.0008415
rs11103648	9	137941317	A	11	0	G	0.0007855
rs11103650	9	137942656	A	11	0	G	0.0007855
rs35973213	9	137960753	G	13	0	A	0.0001783
rs75796057	9	137961094	A	14	0	G	0.00008428
rs943202	10	832519	A	32	50	G	0.0009655
10:2362186	10	2362186	C	0	18	CACACA	0.0000004865
rs71497852	10	3042289	A	0	10	AG	0.0004292
rs7082064	10	3226474	G	18	36	A	0.0008188
rs138359959	10	3318900	C	13	0	CTCAAACCCAAAT	0.0001973
rs56296494	10	5918344	A	0	9	G	0.0009641
rs2274026	10	5960227	G	0	9	A	0.0009641
rs76713178	10	5962261	T	0	9	C	0.0009641
rs17146333	10	5963692	T	0	9	C	0.0009641
rs2274028	10	5965423	A	0	9	G	0.0009641
rs7899635	10	5967177	A	0	9	G	0.0009641
10:5968058	10	5968058	ATTC	0	9	A	0.0009641

rs7904712	10	5971249	T	0	9	A	0.0009641
rs2031230	10	5972582	C	0	9	T	0.0009641
rs7913876	10	5972885	G	0	9	T	0.0009641
rs7918162	10	5974304	G	0	9	A	0.0009641
rs17146580	10	5977684	C	0	9	T	0.0009641
rs17146587	10	5978169	G	0	9	A	0.0009641
rs7893821	10	5978272	C	0	9	G	0.0009641
rs2274031	10	5978629	A	0	9	G	0.0009641
rs2274033	10	5979117	C	0	9	T	0.0009641
rs76091163	10	5980087	G	0	9	C	0.0009641
rs17146629	10	5980750	A	0	9	G	0.0009641
rs75554627	10	5981023	A	0	9	ACTGT	0.0009641
rs117897503	10	5981344	T	0	9	C	0.0009641
rs78846836	10	5981785	G	0	9	A	0.0009641
rs7905947	10	9964509	A	18	37	T	0.0004627
10:10105662	10	10105662	C	26	8	CA	0.000972
rs112216636	10	13264824	C	26	7	T	0.0007902
rs138997947	10	13265182	G	18	3	C	0.0009486
rs138069192	10	13266397	G	21	3	GA	0.0002475
rs78030696	10	13266871	A	20	3	G	0.000269
rs182805957	10	19404368	T	11	0	A	0.0007855
rs76522551	10	19411160	A	12	0	G	0.0003752
rs74477644	10	19416736	G	12	0	A	0.0003752
rs73591925	10	19417095	C	12	0	G	0.0003752
rs78076387	10	19417208	A	11	0	G	0.0007855
rs11013273	10	23335957	A	5	21	G	0.0002243
rs4748836	10	23337395	A	5	21	G	0.0002243
rs7099373	10	25964505	A	47	22	C	0.0008379
rs10629126	10	25977799	TTA	50	25	T	0.0009811
rs10828848	10	25978231	T	50	25	A	0.0009811
rs10764579	10	25978342	G	50	25	A	0.0009811
rs10828849	10	25978533	C	50	25	T	0.0009811
rs11014679	10	25978963	G	50	25	T	0.0009811
rs10764580	10	25986499	C	29	49	G	0.0002928
rs786871	10	26765474	G	33	10	T	0.0003125
rs12414757	10	26767000	T	0	9	A	0.0009641
rs12412826	10	26778606	A	1	13	G	0.0003716
rs112903628	10	26780794	A	1	13	G	0.0003716
rs72803274	10	26782556	A	1	13	G	0.0003716
10:28244637	10	28244637	A	6	22	T	0.0006607
rs11006790	10	28262335	G	20	38	T	0.0009963

rs11595811	10	28268852	G	20	38	A	0.0009963
rs10826379	10	28273575	G	20	38	A	0.0009963
rs4747609	10	28293576	G	21	40	T	0.0006238
rs12411775	10	28294586	T	21	40	G	0.0006238
rs112635025	10	29021693	A	2	15	ATACTTTTTTTTTT	0.0005115
rs561505	10	29477518	C	11	29	T	0.0004498
rs6481863	10	33911780	G	17	36	A	0.0004114
rs7915520	10	33918423	G	17	35	T	0.0007327
rs1902406	10	33919875	G	17	35	T	0.0007327
rs4550167	10	33921708	A	17	35	G	0.0007327
rs4007270	10	35545637	T	18	36	C	0.0008188
rs72305787	10	35548597	A	12	31	ATTGTTTTTTT	0.0003217
rs2176543	10	42393589	A	25	6	G	0.000538
rs7910103	10	45368956	C	16	2	T	0.0008956
rs12266568	10	45369144	C	16	2	T	0.0008956
rs1325170	10	45369361	G	16	2	A	0.0008956
rs10508890	10	45370476	C	16	2	G	0.0008956
rs17157164	10	45370689	T	16	2	C	0.0008956
rs1018702	10	45371418	T	16	2	C	0.0008956
rs1002321	10	45371777	C	16	2	A	0.0008956
10:49239772	10	49239772	T	18	3	TGGCCCC	0.0008352
rs142912173	10	49239797	A	18	3	G	0.0008352
rs148445928	10	49379964	T	26	44	C	0.0008911
rs199919944	10	49389016	T	26	44	C	0.0008911
rs769008	10	49390308	G	26	44	A	0.0008911
rs769009	10	49390449	G	26	44	T	0.0008911
rs2059957	10	49391118	A	26	44	G	0.0008911
rs931743	10	49392575	C	26	44	G	0.0008911
10:49862843	10	49862843	C	0	12	CT	0.00008299
rs117066139	10	49876324	T	0	9	C	0.0009641
rs142841563	10	54999353	A	32	10	ACACACACT	0.0003432
rs11598240	10	55012303	C	40	16	T	0.0005221
rs56261918	10	55021278	C	39	16	T	0.0009032
rs12415393	10	55036000	C	40	15	T	0.0002535
rs11003503	10	55036929	A	40	15	G	0.0002535
rs55985090	10	55037182	A	43	17	T	0.0001922
rs7896245	10	55037271	A	43	16	T	0.00009051
rs58023733	10	55038244	CT	43	16	C	0.00009051
rs12359540	10	55040055	C	41	14	G	0.00006312
rs112215165	10	55813123	C	0	9	A	0.0009641
rs75285476	10	55815450	T	0	9	C	0.0009641

rs4007215	10	56337604	A	33	11	AAATAAATG	0.0006951
rs72803974	10	59064431	C	0	9	T	0.0009641
rs72803979	10	59090003	A	0	9	G	0.0009641
10:61618959	10	61618959	A	0	10	T	0.0004869
10:66724249	10	66724249	G	18	3	A	0.0009486
rs36108765	10	70470256	A	1	14	AT	0.0002194
rs35349003	10	70470266	A	1	14	T	0.0001932
10:70876187	10	70876187	G	14	0	GTGTT	0.00007607
rs7075372	10	72900980	C	25	44	T	0.000488
rs10740366	10	72901958	A	25	44	G	0.000488
rs10733879	10	72902150	G	25	44	T	0.000488
rs10740367	10	72902249	G	25	44	A	0.000488
rs2153711	10	72902948	C	25	44	G	0.000488
rs1935152	10	72903780	C	25	43	T	0.0008649
rs10762427	10	72905452	T	25	44	C	0.000488
rs10823695	10	72905671	G	25	44	A	0.000488
rs7922229	10	72905832	C	25	44	T	0.000488
rs7922634	10	72906185	C	25	44	T	0.000488
rs7922758	10	72906326	A	25	44	T	0.000488
rs1417914	10	72906390	T	25	44	C	0.000488
rs1417916	10	72906547	T	25	44	C	0.000488
rs3058460	10	72906653	CCT	25	44	C	0.000488
rs10762428	10	72907298	T	25	44	C	0.000488
rs7911205	10	72907766	C	25	44	A	0.000488
rs7900002	10	72907951	G	25	44	T	0.000488
rs1417918	10	72909867	T	25	44	G	0.000488
rs1935155	10	72910752	C	25	44	T	0.000488
rs2069017	10	72913419	T	25	44	G	0.000488
rs7070114	10	72915944	T	25	44	C	0.000488
rs7075641	10	72917390	A	25	44	G	0.000488
rs11400244	10	72917529	AT	25	44	A	0.000488
10:72917531	10	72917531	AT	25	44	A	0.000488
rs10509331	10	72919002	G	25	44	A	0.000488
rs947363	10	72921265	T	25	44	C	0.000488
rs1417910	10	72925977	C	25	44	T	0.000488
rs10823699	10	72926445	A	25	44	T	0.000488
rs1539136	10	72928131	G	25	44	A	0.000488
rs1539135	10	72929567	T	24	44	C	0.0002594
rs34868680	10	72935262	T	25	44	TA	0.000488
rs10823700	10	72939292	C	23	42	T	0.0004519
rs1935137	10	72941577	A	25	44	G	0.000488

rs10740368	10	72942724	C	25	44	G	0.000488
rs10762429	10	72942725	A	25	44	G	0.000488
rs1124754	10	72956842	G	26	44	A	0.0008911
rs75086736	10	76287135	C	0	9	A	0.0009641
rs35735588	10	81095875	T	0	9	C	0.0009641
10:83017314	10	83017314	C	9	31	T	0.000008514
rs399496	10	90526585	C	16	34	T	0.0006492
rs367605	10	90530164	C	16	34	T	0.0006492
rs435742	10	90531169	T	16	34	A	0.0006492
rs814630	10	90532331	G	16	34	A	0.0006492
rs303467	10	90533235	G	16	34	A	0.0006492
rs303483	10	90537352	C	16	34	T	0.0006492
rs303488	10	90538984	A	16	34	T	0.0006492
rs303489	10	90539246	G	16	34	A	0.0006492
rs303474	10	90543359	G	16	34	T	0.0006492
rs303478	10	90545972	T	16	34	G	0.0006492
rs303479	10	90546007	A	16	34	G	0.0006492
rs303500	10	90553417	C	17	35	A	0.0007327
rs303502	10	90554113	T	16	35	C	0.000362
rs845134	10	90558717	T	16	35	C	0.000362
rs303541	10	90560417	T	16	35	G	0.000362
rs303539	10	90561586	A	16	35	T	0.000362
rs303537	10	90563593	T	16	35	C	0.000362
rs303532	10	90566243	A	16	35	T	0.000362
rs303531	10	90566474	C	16	35	G	0.000362
10:92193527	10	92193527	C	12	0	G	0.0003752
rs1107172	10	108622962	T	45	20	C	0.0007326
rs874888	10	108623933	A	45	20	T	0.0007326
rs11193079	10	108624572	G	45	20	A	0.0007326
rs1596035	10	109359014	A	9	25	C	0.0008942
rs7910132	10	109361036	G	9	25	C	0.0008942
rs10509839	10	109372314	A	9	26	G	0.0004896
rs10787031	10	109374851	G	12	32	A	0.0001005
rs2083597	10	109375673	A	12	32	G	0.0001005
rs2083596	10	109375778	C	11	32	G	0.00006919
rs11193655	10	109499145	A	8	24	G	0.0006758
rs11193656	10	109499230	T	8	24	A	0.0006758
rs11193659	10	109500575	C	7	22	T	0.0009169
rs12779296	10	109501113	T	7	22	C	0.0009169
rs10787217	10	111621103	A	11	28	T	0.0008103
rs149522291	10	111622391	A	10	28	AAAT	0.0003491

rs10787219	10	111622694	A	10	28	G	0.0003491
rs492943	10	111629030	T	10	28	A	0.0003491
rs11280729	10	111630048	ACTAAATAAAT	10	28	A	0.0003491
rs3740137	10	111630917	A	10	28	G	0.0003491
rs3740138	10	111630931	T	10	28	C	0.0003491
rs3818285	10	111635212	C	10	28	T	0.0003491
rs550697	10	111637286	A	23	41	G	0.0008021
rs4918469	10	111639314	G	10	28	T	0.0003491
rs35712139	10	111642934	C	10	28	T	0.0003491
rs35420248	10	111643074	T	10	28	C	0.0003491
rs112957942	10	111646907	A	10	28	ACAAAAGCTTTAT	0.0003491
rs180946	10	115709203	C	29	9	A	0.0008145
rs151547	10	115715046	A	29	9	G	0.0008145
rs151592	10	115716300	A	33	11	G	0.0006951
rs181507	10	119165059	T	19	3	C	0.0005082
rs4612716	10	119619382	T	41	16	C	0.0002964
rs7909866	10	119619858	G	42	15	A	0.00007618
rs7909868	10	119619859	G	43	16	A	0.00009051
rs6585465	10	119620559	C	43	16	T	0.00009051
rs4752125	10	119621333	A	42	16	G	0.0001653
rs6585466	10	119621697	A	43	16	G	0.00009051
rs6585467	10	119621825	G	43	16	A	0.00009051
rs7087992	10	119621839	C	43	16	T	0.00009051
rs7070315	10	119622054	G	43	16	A	0.00009051
rs28522668	10	125722454	G	2	14	C	0.0009649
rs112396487	10	127613117	G	4	18	A	0.0009242
rs1887097	10	129236512	G	20	40	A	0.0003188
rs2275052	10	129237580	T	15	33	C	0.0005689
rs11815834	10	129238058	G	15	33	A	0.0005689
rs200992214	10	129931769	C	1	14	T	0.0002194
rs551275	10	131036836	A	18	3	C	0.0009486
rs570306	10	131037021	A	18	3	G	0.0009486
rs526562	10	131037236	A	18	3	G	0.0009486
rs10644519	10	131037354	A	18	3	ACC	0.0009486
rs522855	10	131037638	T	18	3	C	0.0009486
rs493861	10	131038424	T	18	3	C	0.0009486
rs496556	10	131038682	C	18	3	G	0.0009486
rs496646	10	131038717	A	18	3	G	0.0009486
rs542661	10	131039216	G	18	3	T	0.0009486
rs524697	10	131039430	T	18	3	C	0.0009486
rs538186	10	131039698	C	18	3	T	0.0009486

rs514158	10	131040058	C	18	3	A	0.0009486
rs555260	10	131040521	A	18	3	G	0.0009486
rs507756	10	131040776	G	18	3	A	0.0009486
rs506910	10	131040856	T	18	3	C	0.0009486
rs481938	10	131041246	T	18	3	A	0.0009486
rs481105	10	131041310	T	18	3	C	0.0009486
rs473925	10	131041379	C	18	3	T	0.0009486
rs499933	10	131041897	C	18	3	T	0.0009486
rs500009	10	131041918	G	18	3	C	0.0009486
rs504586	10	131042419	T	18	3	C	0.0009486
rs531984	10	131043103	G	18	3	A	0.0009486
rs532836	10	131043152	G	18	3	C	0.0009486
rs532788	10	131043179	T	18	3	C	0.0009486
rs499815	10	131044482	C	18	3	G	0.0009486
rs482817	10	131045160	C	18	3	T	0.0009486
rs579888	10	131045400	C	18	3	A	0.0009486
rs578926	10	131045539	T	18	3	G	0.0009486
rs514976	10	131046363	G	18	3	C	0.0009486
rs11597527	10	131047724	A	18	3	G	0.0009486
rs514712	10	131050546	C	18	3	A	0.0009486
rs61859447	10	131051754	C	18	3	T	0.0009486
rs534451	10	131051873	T	18	3	C	0.0009486
rs1750482	10	131052204	A	18	3	G	0.0009486
rs61859448	10	131052387	A	18	3	C	0.0009486
rs6144152	10	131052977	CTCTCACTCTCTT	18	3	C	0.0009486
rs10829622	10	131519512	C	14	32	T	0.0004925
rs34636064	10	131519968	C	14	32	T	0.0004925
rs77183314	10	131520086	A	14	32	AGCATATTAG	0.0004925
rs10829623	10	131520689	C	14	32	T	0.0004925
rs7916932	10	131520975	A	15	33	G	0.0005689
rs6482752	10	131524721	G	10	30	C	0.00009899
rs13377140	10	131526249	T	10	30	A	0.00009899
rs11592158	10	131526661	A	10	30	G	0.00009899
rs7898934	10	131529320	T	10	30	C	0.00009899
rs200859248	10	131531538	C	14	31	A	0.0008795
10:131532499	10	131532499	C	11	31	T	0.0001275
10:131532754	10	131532754	G	10	30	A	0.00009899
rs11595020	10	131536734	A	10	30	G	0.00009899
rs11595599	10	131537580	A	10	30	G	0.00009899
rs10829627	10	131567549	T	16	2	C	0.0008956
rs61874499	10	131637659	G	18	3	A	0.0009486

rs9419235	10	133806576	G	55	27	A	0.0002959
rs9419402	10	133806735	A	55	27	G	0.0002959
rs10747071	10	133810029	G	53	24	A	0.00008869
rs9419406	10	133811201	A	49	24	C	0.000936
rs4880276	10	133815227	A	49	24	C	0.000936
10:134078594	10	134078594	G	17	2	GTGCCTC	0.0003725
10:134078601	10	134078601	A	18	2	AAGGGCGGTGAG	0.0002408
rs10857687	10	134865155	G	0	9	A	0.0009641
rs7130403	11	346997	A	10	27	G	0.0006386
rs10902081	11	1079809	C	30	48	T	0.0009571
rs59720353	11	1489136	T	16	34	C	0.0006492
rs76216459	11	1489424	T	0	9	C	0.0009641
rs7935956	11	3181629	T	43	18	G	0.0003905
rs3794015	11	8264560	T	44	19	C	0.0006784
rs202017636	11	9624708	CT	20	43	C	0.00009249
rs10770043	11	9627471	G	29	48	A	0.0005332
rs10831561	11	11287806	C	26	45	T	0.0005028
rs10741540	11	11310293	A	25	44	G	0.000488
rs10765829	11	11310310	G	25	45	T	0.0002688
rs2699446	11	13729996	G	16	2	A	0.000692
rs12292273	11	19041455	G	1	12	A	0.0007887
rs11025025	11	19140352	C	18	37	A	0.0004627
rs11025026	11	19140385	G	18	37	A	0.0004627
rs4756990	11	19141210	C	19	37	T	0.0009069
rs1503508	11	19141721	A	19	37	C	0.0009069
rs10833064	11	19142375	G	18	37	A	0.0004627
rs11606057	11	19143110	T	19	37	G	0.0009069
rs10500841	11	19143383	C	18	37	A	0.0004627
rs4757757	11	19144144	T	18	37	C	0.0004627
rs11604483	11	19144579	G	19	37	A	0.0009069
rs11604471	11	19144724	C	19	37	T	0.0009069
rs11025028	11	19145288	A	19	37	G	0.0009069
rs1503507	11	19145847	G	18	37	A	0.0004627
rs1503506	11	19145974	G	18	37	A	0.0004627
rs10741761	11	19146531	A	18	38	G	0.0002562
rs7129212	11	19147268	C	18	37	T	0.0004627
rs7129326	11	19147327	C	18	37	T	0.0004627
rs7950788	11	19147479	G	18	37	A	0.0004627
rs7950603	11	19147562	A	18	37	C	0.0004627
rs4757758	11	19155035	G	18	37	A	0.0004627
rs4757759	11	19155219	A	18	37	G	0.0004627

rs7111105	11	19155554	G	18	38	T	0.0002893
rs10766528	11	19156398	C	18	37	T	0.0004627
rs10833065	11	19156496	C	18	37	T	0.0004627
rs4515942	11	19156707	C	19	37	A	0.0009069
rs4326774	11	19156965	G	18	37	T	0.0004627
rs190149361	11	19161016	T	19	40	C	0.0001566
rs201841380	11	19161017	G	20	40	A	0.0003188
rs182791328	11	19161032	A	19	40	C	0.0001726
rs28891775	11	19161806	A	18	37	G	0.0004627
rs4757760	11	19166892	C	18	37	G	0.0004627
rs2271001	11	19167850	A	18	37	G	0.0004627
rs2134622	11	19169508	G	18	37	C	0.0004627
rs3802796	11	19171021	C	18	37	T	0.0004627
rs11607797	11	19178169	A	18	37	T	0.0004627
rs11025037	11	19178764	C	18	37	T	0.0004627
rs12270581	11	19179901	A	18	37	G	0.0004627
rs12801321	11	19181590	A	19	37	G	0.0009069
rs3802797	11	19182288	T	18	37	C	0.0004627
rs3802798	11	19182348	C	19	37	T	0.0009069
rs2042688	11	19186294	C	18	37	A	0.0004627
rs2042687	11	19186826	A	19	37	T	0.0009069
rs1077686	11	19208611	C	14	31	A	0.0008795
rs10833073	11	19218716	C	18	36	A	0.0008188
rs67058053	11	19795319	T	41	17	G	0.0005984
rs1443116	11	19797326	A	41	17	G	0.0005984
rs11025254	11	19797631	C	41	17	A	0.0005984
rs35222325	11	19797656	A	41	17	ACT	0.0005984
rs35383695	11	19798570	T	38	14	C	0.00067
rs7105344	11	19840807	T	48	23	C	0.0008881
rs11025272	11	19841807	C	50	25	A	0.0009811
rs4757852	11	19846915	T	42	18	C	0.0006782
rs6483616	11	19848022	T	42	17	C	0.0003422
rs74358636	11	23039193	GT	21	4	G	0.0008104
rs12279454	11	23042315	T	21	4	C	0.0008104
rs876092	11	23042621	G	21	4	T	0.0008104
rs28591594	11	23043884	T	21	4	C	0.0008104
rs11026910	11	23049858	A	21	4	G	0.0008104
rs10833907	11	23049880	G	21	4	A	0.0008104
rs10833913	11	23054483	C	21	4	T	0.0008104
rs11026934	11	23067120	T	21	4	C	0.0008104
11:23409741	11	23409741	T	28	43	TC	0.0003794

rs71445462	11	23567476	TA	7	24	T	0.0002593
rs12799392	11	26382674	A	5	19	G	0.0008415
rs12790164	11	26384802	C	5	19	T	0.0008415
rs34253346	11	26386955	T	5	19	TG	0.0008415
rs12795939	11	26387285	T	5	19	A	0.0008415
rs77538811	11	26388005	G	5	19	A	0.0008415
rs12805572	11	26388890	A	5	19	T	0.0008415
rs34248864	11	26389892	C	5	19	A	0.0008415
rs12806876	11	26398012	G	5	19	T	0.0008415
rs12791416	11	26403015	T	5	19	C	0.0008415
rs12804855	11	26405037	G	4	19	A	0.0004818
11:26405044	11	26405044	T	4	19	TAGATTGACTGTC	0.0004818
rs34034555	11	26406683	G	5	19	A	0.0008415
rs12785352	11	26410129	G	5	19	T	0.0008415
rs1461378	11	26410930	A	5	19	T	0.0008415
rs10501048	11	26412471	A	5	19	G	0.0008415
rs1461383	11	26414218	G	5	19	A	0.0008415
rs12796591	11	26415691	A	5	19	T	0.0008415
rs142670140	11	26415785	T	5	19	TACTC	0.0008415
rs12795514	11	26415999	C	5	19	A	0.0008415
rs35218098	11	26418820	T	5	19	C	0.0008415
rs113004698	11	26419558	G	5	19	GA	0.0008415
rs12791810	11	26420088	T	5	19	C	0.0008415
rs200695807	11	26987879	G	16	2	C	0.0008956
11:27070014	11	27070014	T	3	18	G	0.0002532
11:27070015	11	27070015	G	3	18	T	0.0002532
rs7123921	11	28428018	C	0	10	T	0.0004292
rs10767981	11	33043577	G	31	10	A	0.0006062
rs28406816	11	33048154	A	31	10	C	0.0006062
rs7121724	11	33048397	T	31	10	C	0.0006062
rs368572891	11	33056812	GCCTCTCCTT	32	10	G	0.0003432
rs60784034	11	33097455	TGGCC	31	10	T	0.0006062
rs73491103	11	33385334	T	20	2	G	0.00006204
rs1065138	11	34379280	A	0	11	G	0.0001895
rs150240560	11	34402655	T	0	11	TC	0.0001895
rs10160662	11	34405010	A	0	11	C	0.0001895
rs11032644	11	34410017	T	0	11	C	0.0001895
rs12276589	11	34414176	A	0	11	T	0.0001895
rs12271710	11	34415592	A	0	11	G	0.0001895
rs684748	11	35504650	G	16	1	C	0.0001772
rs649640	11	35508918	G	16	2	A	0.0008956

11:37161690	11	37161690	TTTAA	20	48	T	0.0000007287
11:38232883	11	38232883	AAGAAAAGAAAA(	0	11	A	0.0001895
11:43575363	11	43575363	C	9	26	T	0.0004896
11:43575367	11	43575367	A	7	24	T	0.0002593
rs9971430	11	43755780	T	29	9	C	0.0008145
rs10838159	11	43762210	G	29	9	A	0.0008145
rs7110115	11	43780193	A	29	9	G	0.0008145
rs12273608	11	43784636	A	29	9	G	0.0008145
rs11037609	11	43786479	T	29	9	C	0.0008145
rs11037610	11	43787521	A	29	9	G	0.0008145
rs11037614	11	43790322	A	29	9	G	0.0008145
rs12363638	11	43795832	T	29	9	C	0.0008145
rs146985020	11	43797034	A	29	9	AT	0.0008145
rs10838164	11	43797474	T	29	9	C	0.0008145
rs6485460	11	43800689	T	29	9	G	0.0008145
rs10400325	11	43807002	A	29	9	G	0.0008145
rs10400390	11	43807392	G	29	9	T	0.0008145
rs11037638	11	43809833	T	29	9	C	0.0008145
rs10838170	11	43815600	G	29	9	A	0.0008145
rs16937633	11	43815806	G	29	9	A	0.0008145
rs6485463	11	43818055	C	29	9	T	0.0008145
rs10838172	11	43820208	A	29	9	C	0.0008145
rs7126905	11	43824778	A	29	9	G	0.0008145
rs7112511	11	43828094	G	29	9	A	0.0008145
rs11037654	11	43828759	A	29	9	G	0.0008145
rs10400368	11	43834451	T	29	9	G	0.0008145
rs147623853	11	43835087	T	29	9	TTTTG	0.0008145
rs59845637	11	43835714	A	29	9	AT	0.0008145
rs2037296	11	43836071	A	29	9	G	0.0008145
rs7105125	11	43840602	A	29	9	T	0.0008145
rs144452302	11	43845598	G	29	9	GA	0.0008145
rs72005218	11	43848205	A	29	9	AAT	0.0008145
rs7942607	11	43849595	C	29	9	T	0.0008145
rs10838183	11	43855072	G	29	9	A	0.0008145
rs141462246	11	43870871	CA	29	9	C	0.0008145
rs835998	11	44931607	T	15	1	C	0.0003561
rs11828734	11	45227196	G	27	45	A	0.0009136
rs11038354	11	45227210	G	27	45	A	0.0009136
rs731102	11	45227974	A	27	45	G	0.0009136
rs7119605	11	45228340	G	25	44	A	0.000488
rs12226640	11	45230443	C	27	45	T	0.0009136

rs3758727	11	45233671	T	27	45	A	0.0009136
rs3758726	11	45241581	G	27	45	A	0.0009136
rs11038358	11	45242558	A	27	45	G	0.0009136
rs11038360	11	45247314	A	27	45	C	0.0009136
rs3758719	11	45250447	A	27	46	C	0.0005154
rs11038363	11	45252267	A	27	45	C	0.0009136
rs79867898	11	45329146	C	1	12	T	0.0007887
rs111479273	11	45331745	A	1	12	T	0.0007887
rs2666918	11	45708930	A	39	15	G	0.0004501
rs2593729	11	45718385	G	39	15	T	0.0004501
rs1348961	11	45719734	T	39	15	A	0.0004501
rs2666913	11	45720898	A	39	15	G	0.0004501
rs2593726	11	45728743	G	39	15	A	0.0004501
rs2011389	11	45730269	G	39	15	A	0.0004501
rs17104	11	45730559	A	39	14	C	0.0002137
rs17105	11	45730635	C	39	14	G	0.0002137
rs756379	11	45730783	G	39	14	A	0.0002137
rs11038620	11	45734981	C	39	14	T	0.0002137
rs2941745	11	45735172	C	39	14	A	0.0002137
rs2666901	11	45739162	C	39	14	T	0.0002137
rs2593733	11	45747590	G	35	13	T	0.000996
rs2593734	11	45748110	T	35	13	C	0.000996
rs2666897	11	45749546	G	26	7	T	0.0007902
rs753198	11	45754962	C	35	13	T	0.000996
rs2666900	11	45757401	T	35	13	C	0.000996
11:46052391	11	46052391	C	14	0	T	0.00008428
rs117529631	11	46159633	T	11	0	C	0.0007855
11:48890089	11	48890089	A	2	14	C	0.0009649
rs200541435	11	61702896	A	3	18	AAAAAAAAAAAA/	0.0002532
11:69081228	11	69081228	AT	23	4	A	0.0001164
rs139373254	11	69436938	C	2	14	A	0.0009649
11:71910931	11	71910931	T	36	12	TGTG	0.0001399
rs1793406	11	75149378	A	2	14	C	0.0009649
rs1871373	11	80097498	C	34	12	T	0.000839
rs17369284	11	81022917	G	0	10	A	0.0004292
rs56177670	11	81023418	A	0	10	G	0.0004292
rs78577737	11	81026664	T	0	10	C	0.0004292
rs55822564	11	81030553	G	0	10	A	0.0004292
rs55811617	11	81030577	T	0	10	C	0.0004292
rs17369881	11	81031398	C	0	10	A	0.0004292
rs79645796	11	81031525	G	0	10	A	0.0004292

rs74590293	11	81031537	G	0	10	T	0.0004292
rs17369902	11	81031588	T	0	10	G	0.0004292
rs142751767	11	81031639	C	0	10	CA	0.0004292
rs17435637	11	81032637	A	0	10	G	0.0004292
rs78294947	11	81033308	C	0	10	T	0.0004292
rs115120279	11	81033409	C	0	10	T	0.0004292
rs17369997	11	81034927	C	0	10	A	0.0004292
rs56334566	11	81035122	T	0	10	TC	0.0004292
rs17141960	11	81035167	T	0	10	C	0.0004292
rs77455791	11	81036467	A	0	10	T	0.0004292
rs78348012	11	81036606	T	0	10	C	0.0004292
rs76083398	11	81037599	T	0	10	G	0.0004292
rs17370219	11	81037858	C	0	10	T	0.0004292
rs116957049	11	81038128	G	0	10	A	0.0004292
rs200508407	11	81038215	CA	0	10	C	0.0004292
rs77489824	11	81042247	A	0	10	C	0.0004292
rs79001473	11	81043432	G	0	10	A	0.0004292
rs77224744	11	81044607	T	0	10	C	0.0004292
rs114629989	11	81044656	A	0	10	G	0.0004292
rs182228872	11	81053076	G	0	10	T	0.0004292
rs185331674	11	81053077	T	0	10	C	0.0004292
rs80328220	11	81053637	T	0	10	A	0.0004292
rs147003829	11	81054364	AT	0	10	A	0.0004292
rs78005260	11	81054839	A	0	10	G	0.0004292
rs76081894	11	81057977	C	0	10	T	0.0004292
rs17371832	11	81059371	T	0	10	C	0.0004292
rs151150014	11	81060030	CATTT	0	10	C	0.0004292
rs56240555	11	81062072	A	0	10	G	0.0004292
rs7114073	11	81063336	A	0	10	T	0.0004292
rs55908086	11	81064868	T	0	10	C	0.0004292
rs139935298	11	81069039	T	0	10	G	0.0004292
rs116471067	11	81069721	G	0	10	A	0.0004292
rs146868223	11	81071462	TAC	0	10	T	0.0004292
rs140721601	11	81071566	T	0	10	TAC	0.0004292
rs17372055	11	81073164	T	0	10	C	0.0004292
rs17437784	11	81074310	T	0	10	C	0.0004292
rs56314671	11	81074741	C	0	10	T	0.0004292
rs72117550	11	81074777	C	0	10	CTTG	0.0004292
rs7358497	11	81075865	A	0	10	G	0.0004292
rs150387842	11	81079159	A	0	10	G	0.0004292
rs55725034	11	81081220	C	0	9	CT	0.000946

rs56085595	11	81081353	T	0	10	C	0.0004292
rs200435150	11	81085102	T	0	10	TTC	0.0004292
rs56025716	11	81086580	G	0	10	T	0.0004292
rs56032706	11	81086849	T	0	10	A	0.0004292
rs80138882	11	81090085	A	0	10	C	0.0004292
rs74639108	11	81091305	G	0	10	A	0.0004292
rs56043695	11	81092528	A	0	10	G	0.0004292
rs78927185	11	81093572	T	0	10	A	0.0004292
rs57921519	11	81095156	T	0	10	G	0.0004292
rs12292395	11	81096278	G	0	10	A	0.0004292
rs12288180	11	81097453	C	0	10	T	0.0004292
rs78410493	11	81101455	G	0	10	C	0.0004292
rs75692693	11	81104851	A	0	10	C	0.0004292
rs74374738	11	81107512	A	0	10	G	0.0004292
rs17439276	11	81108596	C	0	10	T	0.0004292
rs77564017	11	81118376	C	0	10	T	0.0004292
rs74941333	11	81119042	T	0	10	G	0.0004292
rs17439996	11	81122694	C	0	10	T	0.0004292
rs56135807	11	81126313	T	0	10	C	0.0004292
rs74502333	11	81127188	T	0	10	C	0.0004292
11:81127561	11	81127561	C	0	10	CCTCTGTGTGTGT	0.0004292
rs17440143	11	81128565	C	0	10	T	0.0004292
rs79149814	11	81128808	G	0	9	C	0.0008435
rs139433786	11	81128988	TGATC	0	10	T	0.0004292
rs147579820	11	81129670	CTG	0	10	C	0.0004292
rs17440276	11	81130250	C	0	10	G	0.0004292
rs148887234	11	81130953	TGA	0	10	T	0.0004292
rs17440290	11	81131518	C	0	10	A	0.0004292
rs17440339	11	81132432	A	0	10	C	0.0004292
rs200466563	11	87416485	G	3	17	T	0.0005045
rs117847495	11	101532916	T	16	2	A	0.0008956
rs12274654	11	103796623	T	1	12	A	0.0007887
rs149519553	11	104888092	T	0	10	C	0.0004292
rs75888398	11	104907272	G	0	10	T	0.0004292
rs57086873	11	110826764	C	16	34	A	0.0006492
rs61906458	11	114519089	G	15	1	A	0.0003561
rs2459753	11	114525993	G	29	48	A	0.0005332
rs1712802	11	114526484	A	30	48	C	0.0009571
rs1712806	11	114530868	T	29	48	A	0.0005332
rs1607258	11	114532699	T	30	48	C	0.0009571
rs1607259	11	114532788	G	29	48	A	0.0005332

rs1712816	11	114545978	A	29	48	C	0.0005332
rs1792414	11	114546149	C	29	48	G	0.0005332
rs35941139	11	115689375	T	0	11	C	0.0001895
rs12420530	11	119907605	T	17	36	G	0.0007253
rs677247	11	125010299	A	23	41	G	0.0008021
rs10634896	11	125012005	G	19	37	GAGGA	0.0009069
rs599150	11	125012338	A	19	37	C	0.0009069
rs609548	11	125012945	C	24	42	A	0.0008352
rs609550	11	125012952	A	24	42	C	0.0008352
rs2919475	11	125015114	A	56	29	G	0.0005383
rs619552	11	126397820	A	18	39	G	0.000139
rs497753	11	127486960	C	43	19	G	0.000761
rs2512642	11	131277056	A	17	2	G	0.000467
rs12418991	11	131373390	A	2	16	G	0.0002312
rs143545352	11	131373569	ACT	2	15	A	0.0004755
rs444328	11	131374059	T	2	17	C	0.000111
rs116924147	12	239844	A	0	9	C	0.0009641
rs78004686	12	608032	A	0	10	G	0.0004292
rs7977619	12	2335293	G	41	16	A	0.0002575
12:2335297	12	2335297	G	36	11	A	0.00005864
rs10774119	12	3221631	T	5	20	C	0.0004383
rs143642253	12	3227399	TCCCACA	4	18	T	0.0004505
rs11062510	12	3227503	A	4	20	G	0.0002508
rs12308222	12	3227855	G	5	20	C	0.0004383
rs77115380	12	3266644	T	1	12	C	0.0007887
rs11609265	12	6023222	C	16	41	T	0.000007089
12:8235380	12	8235380	T	5	19	A	0.0007163
12:8235382	12	8235382	T	5	19	A	0.0007163
rs73062703	12	10484833	G	28	7	A	0.0002393
rs145800306	12	10484969	GA	28	7	G	0.0002393
rs12370603	12	10485242	G	50	25	C	0.0009811
rs10743917	12	10817402	C	42	18	T	0.0006782
rs10743918	12	10819922	C	40	16	A	0.0005221
rs10845199	12	10820318	C	40	16	T	0.0005221
rs7297205	12	10820840	T	41	17	C	0.0005984
rs7977694	12	10822695	T	41	17	C	0.0005984
rs10772348	12	10822914	G	41	17	A	0.0005984
12:14983473	12	14983473	A	11	28	G	0.0007702
rs645395	12	18796472	T	16	2	G	0.0008956
12:18920312	12	18920312	C	11	0	CA	0.0007855
rs143552182	12	18949309	G	11	0	C	0.0007855

rs117581727	12	19002330	T	11	0	C	0.0007855
rs200443741	12	19849037	G	29	48	GTT	0.0008916
rs2731615	12	19928758	A	34	12	C	0.000839
rs61925727	12	23969278	A	18	36	G	0.0008188
rs4424758	12	23975993	A	18	36	C	0.0008188
rs113537256	12	29752901	A	1	12	ATG	0.0008498
12:29752905	12	29752905	A	1	12	G	0.0008498
rs61924380	12	42871306	G	3	16	A	0.0005264
rs61924381	12	42871308	G	3	16	A	0.0005264
rs11388170	12	48232487	C	42	18	CG	0.0005763
rs11168266	12	48251533	C	43	19	T	0.000761
rs11170000	12	52491833	A	4	21	G	0.00006677
12:52492295	12	52492295	A	43	16	G	0.00006634
rs9738139	12	52535246	C	13	30	T	0.0007578
rs146809456	12	53640383	A	0	10	G	0.0004869
rs7312770	12	56467587	T	31	50	C	0.0005409
rs117120119	12	57237463	G	11	0	C	0.0007855
rs56064527	12	58862337	T	0	10	A	0.0004292
rs34214690	12	58942658	CAG	20	38	C	0.0009963
rs11172810	12	59294631	G	2	14	A	0.0009649
rs73118573	12	59303764	A	2	14	G	0.0009649
rs4758753	12	60880942	A	7	23	G	0.0004919
rs57389490	12	60881991	G	7	23	C	0.0004919
rs73127375	12	60883266	T	7	23	C	0.0004919
rs74094136	12	60883337	T	7	23	C	0.0004919
rs11173493	12	60884349	A	7	23	T	0.0004919
rs10219669	12	60890065	C	7	23	G	0.0004919
rs11173495	12	60890422	A	7	23	G	0.0004919
rs17123875	12	60892973	G	7	23	A	0.0004919
12:62007949	12	62007949	C	0	10	CA	0.0004292
12:62007951	12	62007951	C	0	10	CACACACACACAC	0.0004292
rs4763025	12	63061997	T	11	29	A	0.0004498
rs10710091	12	63062464	G	11	28	GT	0.0008103
rs12300897	12	69365845	C	14	1	T	0.0007094
rs10450797	12	69367815	A	14	1	G	0.0007094
rs12298772	12	69370438	C	14	1	T	0.0007094
rs199594330	12	69515046	A	7	22	AAAAG	0.0008783
rs73139273	12	72711299	T	0	10	C	0.0004292
rs73144999	12	72752908	G	0	10	A	0.0004292
rs201692960	12	72754780	G	1	12	GC	0.0007887
rs368610674	12	72767102	C	1	12	T	0.0007887

rs372909689	12	72767107	T	1	12	C	0.0007887
rs377070019	12	72767108	G	1	12	A	0.0007887
rs73146906	12	72783553	G	0	10	A	0.0004292
rs73146915	12	72798547	A	1	12	G	0.0007887
12:73570125	12	73570125	C	0	13	CACACACACACAC	0.00003603
rs35782366	12	73733999	G	1	12	A	0.0007887
rs145808635	12	73791877	G	0	11	A	0.0001604
rs201246630	12	73792138	CA	0	11	C	0.0001895
rs117155171	12	73825708	A	0	9	G	0.0009641
rs12815472	12	73998450	T	0	9	C	0.0009641
rs146922274	12	77467304	T	1	12	TTTTA	0.0007887
rs61932809	12	77468993	T	1	12	C	0.0007887
rs61930667	12	77472384	A	1	12	T	0.0007887
rs61930670	12	77475922	G	1	12	A	0.0007887
rs61930671	12	77478571	T	1	12	C	0.0007887
rs11610700	12	77482684	A	1	12	C	0.0007887
rs930645	12	77492338	T	10	31	A	0.00005135
rs7298978	12	77493397	T	10	31	C	0.00005135
rs76566838	12	77495670	A	1	12	AGCC	0.0007887
rs61930294	12	77496073	A	1	12	G	0.0007887
rs74633707	12	77499208	A	1	12	G	0.0007887
rs56149569	12	77500698	C	1	12	T	0.0007887
rs9971921	12	77501316	T	1	12	C	0.0007887
rs61930296	12	77502598	A	1	12	G	0.0007887
rs78152080	12	77503218	T	1	12	C	0.0007887
rs75366818	12	77503221	C	1	12	T	0.0007887
rs59223587	12	77503897	T	1	12	C	0.0007887
rs61930298	12	77504676	G	1	12	A	0.0007887
rs10506730	12	77508673	G	1	12	A	0.0007887
rs10506731	12	77509089	G	1	12	A	0.0007887
rs11610352	12	77510711	G	1	12	A	0.0007887
rs72615642	12	77511067	C	9	27	T	0.0002633
rs1868356	12	77515225	G	9	27	A	0.0002633
rs11117188	12	77515573	G	9	27	A	0.0002633
rs10858400	12	77519228	T	9	27	C	0.0002633
rs7487338	12	77520190	G	0	11	A	0.0001555
rs1874887	12	77520378	C	9	27	G	0.0002633
rs771617	12	77531634	A	11	30	G	0.0002453
rs771659	12	77540253	T	10	29	C	0.0001875
rs1112921	12	77714826	T	38	15	C	0.0007851
rs60172725	12	77719625	C	41	15	T	0.0001402

rs11614989	12	78746493	C	29	47	A	0.0009467
rs148372708	12	80431354	GTCTA	0	9	G	0.0009641
rs5009830	12	82069012	T	3	17	G	0.0005045
rs61929509	12	83042393	A	17	35	G	0.0007327
rs12370635	12	84468344	G	11	0	A	0.0007855
rs4275668	12	94258196	C	49	24	T	0.000936
rs11107224	12	94258226	C	49	24	G	0.000936
rs4258423	12	94258229	T	49	24	C	0.000936
rs2028204	12	94258282	G	24	48	A	0.0000187
rs1002121	12	94258726	C	49	24	T	0.000936
rs1493855	12	94258930	G	31	50	A	0.0005409
rs1493854	12	94259043	T	31	50	A	0.0005409
rs11378803	12	94259600	A	20	44	AT	0.00001503
rs4761747	12	94259772	T	24	47	C	0.00003747
rs55956322	12	94265605	G	49	24	T	0.000936
rs7134396	12	94266865	G	31	10	A	0.0006062
rs76846711	12	94267015	GA	49	24	G	0.000936
rs35784448	12	94267327	G	49	24	A	0.000936
rs34243602	12	94269027	GT	50	24	G	0.0005368
rs7306328	12	94269138	G	21	44	A	0.00003223
rs7136883	12	94269207	C	49	24	T	0.000936
rs4761531	12	94269467	A	49	24	C	0.000936
rs4761532	12	94269672	G	49	24	A	0.000936
rs61927337	12	94269974	T	49	24	C	0.000936
rs71443557	12	94270817	A	49	24	AGTAACACT	0.000936
rs1493853	12	94271275	T	31	50	C	0.0005409
rs1493852	12	94271348	T	49	24	C	0.000936
rs7969712	12	94272243	C	49	24	T	0.000936
rs7969978	12	94272451	C	49	24	T	0.000936
rs35613486	12	94272580	G	49	24	A	0.000936
rs11107231	12	94273453	A	31	10	T	0.0006062
rs11107232	12	94273459	G	31	10	A	0.0006062
rs7980263	12	94289089	T	18	3	C	0.0009486
12:94291604	12	94291604	C	14	0	CCG	0.00007607
rs12368075	12	94293569	A	19	3	G	0.0005082
rs12371409	12	94293757	G	19	3	A	0.0005082
rs11107251	12	94293988	T	18	3	C	0.0009486
rs17790566	12	94294235	A	18	3	G	0.0009486
12:94294689	12	94294689	A	18	3	C	0.0009486
rs11107257	12	94304153	A	40	17	G	0.0009663
rs74728968	12	94936045	A	0	10	C	0.0003596

rs12824690	12	94948451	G	2	16	C	0.0002312
rs35677689	12	94950003	T	1	13	G	0.0003716
rs17022724	12	94952716	T	1	13	G	0.0003716
rs78104341	12	94966993	C	31	49	CTCA	0.0009634
rs1274525	12	94968493	G	31	49	A	0.0009634
rs1274528	12	94969866	A	31	49	T	0.0009634
rs1274529	12	94969872	G	31	49	A	0.0009634
rs1274530	12	94970125	A	31	49	G	0.0009634
rs1274531	12	94970275	A	31	49	T	0.0009634
rs1274532	12	94970382	T	31	49	G	0.0009634
rs1274533	12	94970570	A	31	49	T	0.0009634
rs1296197	12	95024859	T	25	44	A	0.000488
rs1274521	12	95026753	G	25	45	A	0.0002688
rs1290907	12	95027785	G	30	48	A	0.0009571
rs71071799	12	95054585	C	5	20	CT	0.0007845
rs9668373	12	95179709	C	48	23	T	0.0008881
rs9667991	12	95179862	T	48	23	G	0.0008881
rs10745707	12	95284635	G	49	21	A	0.0001359
rs10745708	12	95284726	G	49	21	A	0.0001359
rs10859781	12	95284857	C	49	21	G	0.0001359
rs10859782	12	95284883	C	49	21	A	0.0001359
rs11107774	12	95285064	G	49	21	C	0.0001359
rs58035116	12	95285195	GA	47	21	G	0.000234
rs10745709	12	95288047	T	49	21	C	0.0001359
rs3858656	12	96880553	G	3	16	A	0.0009912
rs2052295	12	96985378	A	0	9	T	0.0009641
rs73147818	12	100704733	C	0	10	G	0.0004292
rs7969213	12	101434784	C	4	18	T	0.0009242
rs7952812	12	101439513	G	7	24	C	0.0002593
rs2723875	12	104368449	C	10	27	A	0.0006386
rs2629805	12	104392195	A	9	27	T	0.0002909
12:105526569	12	105526569	A	46	18	T	0.0001043
rs111759074	12	107644137	T	1	12	C	0.0009476
rs7133632	12	107671301	A	1	12	G	0.0007887
rs113627797	12	107672272	A	1	12	G	0.0007887
rs74394042	12	107672914	G	1	12	T	0.0007887
rs117090648	12	108193398	A	3	18	G	0.0002532
rs73191612	12	108194285	T	1	17	C	0.00001632
rs73191615	12	108199455	T	2	18	C	0.00005262
rs73191653	12	108238939	G	2	15	A	0.0004755
rs56786064	12	108263741	T	2	15	G	0.0004755

rs4766845	12	117787554	T	30	9	G	0.0004612
rs3782219	12	117788240	T	30	9	C	0.0004612
rs4767532	12	117790932	T	30	9	C	0.0004612
rs61938114	12	119913163	A	14	1	C	0.0007094
rs112287083	12	124281943	C	2	15	CT	0.0005115
rs4760576	12	129253864	A	18	36	G	0.0008188
rs113368175	13	19386067	G	15	1	A	0.0003561
rs376761445	13	19679763	T	18	3	G	0.0009486
13:20121862	13	20121862	TAC	14	1	T	0.0006426
rs11840315	13	23631746	C	34	12	T	0.000839
rs4770372	13	23637683	T	34	12	G	0.000839
rs10536822	13	25493182	G	31	10	GAGAT	0.0006062
13:28831824	13	28831824	TATAAATATATAT	1	13	T	0.0002855
rs7321093	13	29147841	C	29	48	T	0.0005332
rs7321403	13	29147852	A	36	13	G	0.0005704
rs7326455	13	29148277	C	29	48	T	0.0005332
rs3812868	13	29149341	C	29	47	G	0.0009467
rs3829387	13	29149693	C	31	10	A	0.0006062
rs7327608	13	29150120	A	29	47	G	0.0009467
rs2006761	13	29150147	C	29	47	A	0.0009467
rs7328082	13	29150190	G	29	47	A	0.0009467
rs7327963	13	29150273	C	29	47	G	0.0009467
rs6490311	13	29150759	A	29	47	G	0.0009467
rs327118	13	29506175	C	23	41	T	0.0008021
rs9508336	13	29863014	G	14	33	C	0.0002703
rs7319242	13	29863365	T	14	32	C	0.0004925
rs4769709	13	29865808	A	19	37	G	0.0009069
rs11365604	13	29865972	C	14	32	CA	0.0004925
rs118079285	13	32097993	A	0	9	G	0.0009641
rs377801	13	33556219	C	36	13	T	0.0005704
13:49911875	13	49911875	C	3	18	CACA	0.0002532
rs4942985	13	51833944	C	2	14	A	0.0009649
rs73194198	13	51834884	T	2	14	G	0.0009649
rs17837207	13	51839336	A	2	14	G	0.0009649
rs9592367	13	65386113	A	14	1	G	0.0007094
rs73200478	13	65387678	T	14	1	C	0.0007094
rs73200481	13	65390377	A	14	1	G	0.0007094
rs150865846	13	65400548	A	14	1	ATTT	0.0007094
rs9598840	13	65406636	G	14	1	C	0.0007094
rs12429888	13	65413569	T	14	1	C	0.0007094
rs9598841	13	65419872	T	14	1	A	0.0007094

13:65760777	13	65760777	A	25	6	T	0.0005219
rs7492037	13	69418044	A	38	13	G	0.0001774
rs74096538	13	75143340	C	0	10	T	0.0004292
rs74096539	13	75143364	A	0	9	C	0.0009641
rs74096540	13	75143442	G	0	10	A	0.0004292
rs74094341	13	75143661	C	0	10	T	0.0004292
13:77819880	13	77819880	A	23	44	ACAC	0.0001337
rs475743	13	80767182	G	7	22	A	0.0009169
rs9560773	13	87414522	C	30	48	T	0.0009571
rs9515910	13	87416030	T	30	48	C	0.0009571
rs35420831	13	87416276	G	30	48	A	0.0009571
rs12430623	13	87416571	C	30	48	A	0.0009571
rs9589198	13	87417865	G	30	48	C	0.0009571
rs9523309	13	87418563	C	30	48	A	0.0009571
rs9515916	13	87419768	A	30	48	G	0.0009571
rs4132042	13	87422551	C	30	48	A	0.0009571
rs9515922	13	87423865	C	30	48	T	0.0009571
rs9556082	13	87425067	G	30	48	C	0.0009571
rs9523333	13	87427762	A	30	48	G	0.0009571
rs9556088	13	87430634	T	30	48	C	0.0009571
rs9556089	13	87430914	C	47	22	T	0.0008379
rs6492549	13	87434336	C	30	48	T	0.0009571
rs4261395	13	87438236	G	30	48	C	0.0009571
rs9556102	13	87439318	T	30	48	C	0.0009571
rs9523362	13	87439915	C	30	49	A	0.0005383
rs7332982	13	87440616	G	30	48	A	0.0009571
rs7333973	13	87441079	G	30	48	C	0.0009571
rs7988039	13	87443524	C	30	48	T	0.0009571
rs9515957	13	87445503	A	29	48	G	0.0005332
rs9301741	13	87446900	A	29	48	C	0.0005332
rs9515961	13	87447243	C	29	48	T	0.0005332
rs9301746	13	87448003	G	29	48	T	0.0005332
rs9523390	13	87448481	C	29	48	T	0.0005332
rs3015542	13	87451410	C	29	48	G	0.0005332
rs2992717	13	87452111	A	29	48	G	0.0005332
rs7995257	13	87453249	A	29	48	G	0.0005332
rs2992707	13	87454536	A	29	48	G	0.0005332
rs2992708	13	87455045	A	30	48	G	0.0009571
rs2992711	13	87457101	T	29	48	A	0.0005332
rs9515965	13	87459754	C	29	48	G	0.0005332
rs2992713	13	87461696	G	29	48	A	0.0005332

rs2992714	13	87461949	G	29	48	A	0.0005332
rs9523416	13	87463100	G	29	48	A	0.0005332
rs12429287	13	87469360	A	48	22	G	0.0004783
rs9589359	13	87469749	A	29	48	G	0.0005332
rs3015543	13	87469947	A	29	48	G	0.0005332
rs3015512	13	87471178	C	29	48	A	0.0005332
rs4773640	13	87474932	T	29	48	G	0.0005332
rs4287439	13	87475513	A	29	48	G	0.0005332
rs9556132	13	87476060	C	29	48	T	0.0005332
rs7984450	13	87476225	T	29	48	G	0.0005332
rs9515993	13	87478483	G	29	48	C	0.0005332
rs9515995	13	87479611	T	29	48	C	0.0005332
rs7337025	13	87481267	T	29	48	C	0.0005332
rs9560893	13	87481783	G	29	48	T	0.0005332
rs9560911	13	87490783	C	28	49	T	0.000155
rs9523520	13	87499244	C	28	49	T	0.000155
rs4420385	13	87501789	A	28	49	T	0.000155
rs4238296	13	87506446	G	27	49	A	0.00007972
rs9523562	13	87511042	G	28	49	A	0.000155
rs9556166	13	87514739	G	28	49	T	0.000155
rs9556167	13	87514778	G	28	49	A	0.000155
rs9561016	13	87518870	G	28	49	T	0.000155
rs4238298	13	87527893	T	30	50	C	0.0002951
rs7982268	13	87532943	G	30	50	A	0.0002951
rs7330816	13	87534747	G	30	50	A	0.0002951
rs11330429	13	87540259	C	51	23	CT	0.000156
rs6492625	13	87547394	G	29	47	A	0.0009467
rs9516118	13	87552124	G	29	47	T	0.0009467
rs7981849	13	87560377	A	29	47	C	0.0009467
rs1718017	13	87596099	G	29	47	A	0.0009467
rs2685796	13	87603946	A	29	47	G	0.0009467
rs969501	13	87617112	G	51	24	A	0.0003012
rs2685792	13	87627796	A	29	47	C	0.0009467
rs2685793	13	87629552	G	29	47	A	0.0009467
rs8000914	13	87638878	T	29	47	C	0.0009467
rs9556308	13	87641649	G	29	47	T	0.0009467
rs2049200	13	87643970	A	29	47	G	0.0009467
rs7319660	13	87645657	A	29	47	G	0.0009467
rs1522014	13	87658986	A	29	47	G	0.0009467
rs9524240	13	87666355	G	29	47	C	0.0009467
rs9524241	13	87666356	A	29	47	T	0.0009467

rs1040286	13	87668544	T	29	47	C	0.0009467
rs926016	13	87668961	T	29	47	C	0.0009467
rs9301917	13	87674122	C	29	47	A	0.0009467
rs34520380	13	87678333	C	29	47	T	0.0009467
rs9561480	13	87679842	A	29	47	G	0.0009467
rs1543232	13	87682869	A	29	47	G	0.0009467
rs6492695	13	87695546	G	29	48	A	0.0005332
rs9524345	13	87700013	C	29	48	T	0.0005332
rs9524365	13	87702747	A	29	48	T	0.0005332
rs9524367	13	87703310	T	29	48	G	0.0005332
rs9516366	13	87705456	C	29	48	T	0.0005332
rs9556359	13	87706470	T	29	48	C	0.0005332
rs9524373	13	87706576	A	29	48	G	0.0005332
rs9516372	13	87709360	C	29	48	T	0.0005332
rs9516374	13	87709690	C	29	48	T	0.0005332
rs9524401	13	87712675	A	29	48	G	0.0005332
rs9561541	13	87719368	A	29	48	T	0.0005332
rs9561544	13	87720442	G	29	48	A	0.0005332
rs7987894	13	87722426	G	29	48	T	0.0005332
rs5805422	13	87723820	TAGAG	29	48	T	0.0005332
rs34773214	13	87724838	TCAAA	29	48	T	0.0005332
rs9645930	13	87726002	C	29	48	T	0.0005332
rs148494630	13	87731770	ATAGCTCTTTCAG	27	46	A	0.0005154
rs34201290	13	87732266	A	28	46	AT	0.0009321
rs199991716	13	93547283	A	16	2	T	0.0008037
rs9523882	13	93651756	G	23	5	A	0.0006807
rs9524483	13	95069280	A	1	12	G	0.0007887
rs7333025	13	95070898	T	1	12	C	0.0007887
rs9524484	13	95072900	T	1	12	C	0.0007887
rs9524485	13	95072976	T	1	12	C	0.0007887
rs149410208	13	95074442	TA	0	12	T	0.00008299
rs72636617	13	95075219	A	1	12	G	0.0007887
rs4145275	13	104352252	A	28	8	G	0.0006092
rs61347079	13	104372780	G	37	12	GT	0.0001447
rs75172355	13	104372787	C	36	12	T	0.0002646
rs149175792	13	113890060	G	0	9	A	0.0009641
rs8013531	14	23247687	T	26	44	C	0.0008911
rs3742491	14	24429249	A	18	3	G	0.0009486
rs139045649	14	24432493	C	19	3	G	0.0005045
rs138982419	14	24435308	A	21	4	C	0.0008104
rs141694248	14	24436047	T	18	3	C	0.0009486

rs184829013	14	24476128	G	22	2	A	0.00003198
rs151035018	14	24477842	T	22	4	C	0.00043
rs7142198	14	24945331	G	31	10	A	0.0006062
rs72679358	14	24960835	C	31	10	G	0.0006062
rs6573990	14	25212506	T	19	38	G	0.0005156
rs1461650	14	25213422	T	19	38	G	0.0005156
14:29935423	14	29935423	AAATATATAAATA	0	10	A	0.0004869
14:38510836	14	38510836	T	12	0	C	0.0004098
rs2415840	14	44650338	C	31	10	T	0.0006062
rs112448176	14	44667587	ATG	20	3	A	0.000269
rs61563993	14	44855154	A	16	2	G	0.0008956
rs12433092	14	44862795	A	16	2	G	0.0008956
rs10148461	14	44866697	A	16	2	G	0.0008956
rs75760252	14	44875805	T	16	2	C	0.0008956
rs11570840	14	50807624	T	29	9	C	0.0008145
rs113252015	14	50808188	C	39	11	G	0.00001931
rs11570836	14	50808965	C	29	9	A	0.0008145
rs11570825	14	50812183	C	29	9	G	0.0008145
rs1890979	14	50812791	T	29	9	C	0.0008145
rs200323076	14	50813375	TTG	29	8	T	0.000328
rs72683953	14	50815259	C	29	9	G	0.0008145
rs11570819	14	50824413	T	29	9	C	0.0008145
rs61981900	14	50835699	G	29	9	C	0.0008145
rs56149746	14	50835834	T	29	9	C	0.0008145
rs4901023	14	50837066	A	29	9	C	0.0008145
rs56381880	14	50838885	A	29	9	G	0.0008145
rs61981904	14	50840007	A	29	9	G	0.0008145
rs58086072	14	50840712	C	29	9	T	0.0008145
rs61424487	14	50840794	T	29	9	C	0.0008145
rs112724011	14	50854706	T	28	8	C	0.0006092
rs9323244	14	53914318	C	43	19	T	0.000761
14:61724348	14	61724348	CA	1	16	C	0.00003625
rs2022663	14	62832374	C	24	42	G	0.0008352
rs8013550	14	62836092	G	43	19	T	0.000761
rs12434562	14	69318352	T	18	3	C	0.0009486
rs2104090	14	69323589	T	18	3	C	0.0009486
rs4899384	14	71625956	T	30	48	A	0.0009571
rs35518531	14	74029894	C	28	7	CTCTT	0.0002393
rs8020801	14	74030964	T	29	7	C	0.0001288
rs7142798	14	74032326	A	28	8	G	0.0006092
rs10138751	14	74038214	G	28	7	A	0.0002393

rs10140438	14	74040562	T	28	7	C	0.0002393
rs33994188	14	74040740	AT	28	8	A	0.0002984
rs4044907	14	74047195	T	28	7	C	0.0002393
rs4044908	14	74047219	C	28	7	T	0.0002393
rs6574128	14	74051889	T	28	7	G	0.0002393
rs2079536	14	74052930	A	28	7	T	0.0002393
rs1859910	14	74054221	A	29	8	G	0.0003391
rs8015976	14	74055115	G	28	7	A	0.0002393
rs34329309	14	74068631	CT	31	50	C	0.0005409
rs10141401	14	74074804	T	31	49	C	0.0009634
rs10150828	14	74077485	T	29	48	C	0.0005332
rs8008001	14	76583051	C	5	19	T	0.0008415
14:77319775	14	77319775	A	0	10	T	0.0004292
14:78492143	14	78492143	TGTGTGTGC	17	35	T	0.0007327
14:83762300	14	83762300	T	16	2	C	0.0008956
rs55968259	14	88653977	C	37	14	A	0.0006739
14:89482375	14	89482375	A	2	14	T	0.0009649
rs71107540	14	90124316	A	39	16	AATTATT	0.0009032
rs10646499	14	90129322	C	51	24	CTG	0.0003012
rs2632191	14	90132482	C	52	24	T	0.0001653
rs2018763	14	90132951	A	39	15	G	0.0004501
rs12587740	14	90135343	T	51	24	C	0.0003012
rs4904595	14	90135472	C	38	14	G	0.0003829
rs8006303	14	90137426	A	38	14	G	0.0003829
rs998564	14	90139311	C	51	23	T	0.000156
rs2896113	14	90139991	C	51	23	T	0.000156
rs1967774	14	90142951	A	29	53	G	0.00004072
rs732914	14	90148522	A	52	23	G	0.00008358
rs8019799	14	90148832	A	36	12	G	0.0002646
rs7158240	14	90150146	G	40	14	A	0.0001172
rs61998683	14	90150796	C	37	12	T	0.0001447
rs2069963	14	95052627	T	14	31	C	0.0008795
rs56348431	14	95174005	A	16	2	C	0.0008956
rs1892224	14	98763349	T	53	26	C	0.0003309
rs10138335	14	98772136	C	53	26	T	0.0003309
rs1807069	14	98773458	G	53	27	A	0.0006109
rs60500823	14	98794793	T	50	25	C	0.0009811
rs34173953	14	98797235	G	50	25	GA	0.0009811
rs4365229	14	98806562	T	53	26	C	0.0003309
rs7145018	14	104626493	C	10	27	T	0.0006386
rs12882239	14	104626811	C	7	23	G	0.0004919

rs141671546	14	104631874	GTC	15	39	G	0.00001206
rs148671298	14	104631988	ATG	6	23	A	0.0001763
14:106587352	14	106587352	A	16	1	AT	0.0001772
14:106784161	14	106784161	T	20	3	C	0.000269
15:21181978	15	21181978	G	0	10	C	0.0004292
rs61999421	15	23158339	C	19	37	G	0.0009069
rs17114595	15	24929290	C	8	25	T	0.0003633
rs201751200	15	30384836	G	16	2	A	0.0008912
15:32679629	15	32679629	G	5	20	A	0.0007845
rs1258795	15	33107305	A	23	41	G	0.0008021
rs146351760	15	34278834	G	5	19	GACCATAAGGCT(	0.0008415
rs200623438	15	34278848	G	5	19	A	0.0008415
rs34976114	15	34278958	A	5	19	AGC	0.0008415
15:35070132	15	35070132	TTATTATTATTATT	2	15	T	0.0004989
rs10744966	15	36027556	A	10	27	C	0.0006386
rs57424653	15	36028766	TAAAACAGGGAT/	10	27	T	0.0006386
rs2647684	15	36031999	C	10	27	T	0.0006386
rs2647683	15	36032032	A	10	27	C	0.0006386
rs201416309	15	37534336	AC	1	12	A	0.0007887
rs28769958	15	37604602	A	32	50	G	0.0009655
rs377524289	15	37695564	C	0	9	A	0.0009641
rs28807491	15	37697517	A	29	9	G	0.0008145
rs377390681	15	37721779	A	0	9	G	0.0009641
rs28464240	15	37940361	G	17	2	A	0.000467
rs11073272	15	38137663	A	4	19	G	0.0004818
rs17647739	15	38517147	G	0	10	A	0.0004292
rs78688956	15	38673907	C	15	1	A	0.0003561
rs1004496	15	38941557	C	30	48	A	0.0009571
rs7173954	15	41275014	G	20	38	T	0.0009963
rs78604303	15	41436999	A	3	16	G	0.0009912
rs692390	15	41486990	A	5	19	G	0.0008415
rs2445765	15	51634898	C	23	5	G	0.0006807
rs113112680	15	51648220	C	14	1	T	0.0007094
rs76564255	15	51998790	A	3	17	G	0.0005045
rs76444462	15	52005282	T	3	16	C	0.0009912
rs113030016	15	52060798	A	2	15	G	0.0005115
rs201337109	15	52977626	G	0	11	GT	0.0002508
rs201699031	15	52986366	TA	0	10	T	0.0004869
rs8030746	15	57766915	C	13	30	A	0.0007578
rs59776586	15	59843987	A	17	35	G	0.0007327
rs144331371	15	59854321	A	16	32	G	0.000944

rs112689995	15	60139296	T	11	0	G	0.0007855
rs77269568	15	60139337	A	11	0	G	0.0007855
rs725463	15	60143079	T	31	51	C	0.0002959
rs60731795	15	60152549	G	18	1	A	0.00004271
rs77682314	15	60155552	T	18	1	G	0.00004271
rs2970397	15	60173234	T	50	25	C	0.0009811
rs2960809	15	60173272	G	51	25	A	0.0005636
rs56278535	15	60176111	T	50	22	C	0.0001461
rs893276	15	60177158	A	50	25	G	0.0009811
rs4774348	15	60178791	A	30	48	C	0.0009571
rs56205983	15	60180167	T	30	48	C	0.0009571
rs11853750	15	60191548	T	30	48	C	0.0009571
rs4774349	15	60193706	T	29	47	C	0.0009467
rs34355056	15	65703625	G	23	5	C	0.0006807
rs623887	15	65710002	T	25	5	C	0.0001938
15:79014065	15	79014065	T	9	25	C	0.0008942
rs10468216	15	83248039	T	14	1	C	0.0007094
rs12441668	15	83249012	A	14	1	G	0.0007094
rs72751665	15	83270419	C	14	1	T	0.0007094
rs201806597	15	83280797	TA	16	1	T	0.0001772
rs199976048	15	86780174	G	23	44	GGGGGGGGGC	0.0003434
rs67074928	15	88106241	GA	18	3	G	0.0009486
rs78256185	15	94454328	G	18	3	C	0.0009486
rs6496098	15	95846311	C	14	31	T	0.0008795
rs8030710	15	95850069	G	10	27	T	0.0006386
rs1837920	15	96221632	G	2	14	C	0.0009649
rs7167125	15	96224785	A	1	13	T	0.0003716
rs12438895	15	96226126	A	2	14	G	0.0009649
rs72764079	15	96230394	G	1	13	A	0.0003716
rs200086610	15	96248963	G	1	13	GCA	0.0003716
rs201009337	15	96248966	TG	1	13	T	0.0003716
rs2012110	15	96250179	G	1	13	A	0.0003716
rs78317776	15	97681623	T	0	9	C	0.0009641
rs72754651	15	98491336	T	15	1	C	0.0003561
rs4966014	15	99248018	C	14	33	T	0.0002703
rs116991589	15	99411911	T	0	9	C	0.0009641
rs59399015	15	100320088	G	5	19	A	0.0008415
rs12899846	15	100324311	T	5	19	C	0.0008415
rs79654877	15	100325738	T	5	19	C	0.0008415
rs75977845	15	100330335	G	5	19	GTATTT	0.0008415
rs9920179	15	100334206	A	5	19	C	0.0008415

rs9920699	15	100334668	C	5	19	T	0.0008415
rs62040189	15	100334872	A	5	19	G	0.0008415
rs79004777	15	100335128	T	5	19	TG	0.0008415
rs71399877	15	100335286	C	5	19	T	0.0008415
rs56401822	15	100335740	G	5	19	A	0.0008415
rs56767751	15	100336365	C	5	24	CAAAAT	0.00002717
rs2958996	15	100336397	A	5	24	G	0.00002717
rs12916037	15	100340976	C	5	19	T	0.0008415
rs12915962	15	100342213	A	5	19	G	0.0008415
rs2958976	15	100342525	C	5	19	T	0.0008415
rs2958975	15	100342635	C	5	19	T	0.0008415
rs113107454	15	100342772	A	5	19	ATCATGGGC	0.0008415
rs199990996	15	100343037	A	5	19	ATAAAG	0.0008415
rs71407353	15	100343060	T	5	19	C	0.0008415
rs112377392	15	100343472	T	5	19	G	0.0008415
rs74038119	15	100343487	G	5	19	T	0.0008415
rs80273863	15	100343761	T	5	19	C	0.0008415
rs62040219	15	100344543	G	5	19	T	0.0008415
rs62040222	15	100345564	T	5	19	G	0.0008415
15:100351251	15	100351251	GT	5	19	G	0.0008415
rs35894461	15	100417009	G	3	16	GA	0.0009912
rs28619387	15	100421261	C	7	23	A	0.0004919
rs11856091	15	100421271	A	7	23	C	0.0004919
rs11852933	15	100421490	G	7	23	A	0.0004919
rs2727170	15	100421840	A	7	23	G	0.0004919
rs28608162	15	100422080	C	7	23	A	0.0004919
rs28647910	15	100422084	C	7	23	T	0.0004919
15:100422275	15	100422275	T	7	23	C	0.0004919
15:100422276	15	100422276	G	7	23	A	0.0004919
rs201333708	15	100422360	G	7	23	GT	0.0004919
rs8030437	15	100422411	T	7	23	A	0.0004919
rs8035690	15	100422874	C	7	23	T	0.0004919
rs10468176	15	100423144	T	7	23	C	0.0004919
rs10468177	15	100423145	A	7	23	G	0.0004919
rs28513757	16	866351	A	2	14	G	0.0009649
rs2335858	16	2972033	A	11	0	C	0.0007855
rs11865663	16	2972534	A	11	0	C	0.0007855
rs59008953	16	2977283	G	12	0	C	0.0003752
16:6703978	16	6703978	G	0	10	A	0.0003596
rs34484063	16	9230940	T	9	27	C	0.0002633
rs12929306	16	9231253	T	9	29	C	0.00007225

rs13339039	16	9231530	C	9	27	G	0.0002633
rs13339041	16	9231534	T	9	27	G	0.0002633
rs72778454	16	9617285	A	7	22	G	0.0009169
rs12919665	16	9707265	C	19	37	T	0.0009069
rs718396	16	9708268	G	19	37	A	0.0009069
rs12927792	16	9713194	T	30	49	C	0.0005383
16:19552980	16	19552980	A	21	4	G	0.0008104
rs26765	16	23700266	C	27	8	A	0.0009854
rs368108	16	24252155	A	41	17	G	0.0005984
rs17815868	16	24283930	T	31	10	A	0.0006062
rs11645882	16	24284136	T	31	10	C	0.0006062
rs2238501	16	24285989	A	31	10	G	0.0006062
rs11640324	16	24287656	A	31	10	G	0.0006062
rs11646923	16	24287692	G	31	10	A	0.0006062
rs2238503	16	24288739	G	31	10	C	0.0006062
rs2238505	16	24296183	A	31	10	G	0.0006062
rs2238506	16	24297983	T	31	10	C	0.0006062
rs62029005	16	24299671	A	31	10	T	0.0006062
rs72781836	16	24300287	A	31	10	G	0.0006062
rs62029006	16	24301475	A	31	10	G	0.0006062
rs17759710	16	24301701	T	31	10	C	0.0006062
rs62029007	16	24301731	T	31	10	C	0.0006062
rs12598137	16	24301897	C	31	10	T	0.0006062
rs11640935	16	24303574	T	31	10	C	0.0006062
rs62029010	16	24305430	A	31	10	G	0.0006062
rs62029013	16	24308704	A	31	9	G	0.0002572
rs111818845	16	26814625	G	12	0	A	0.0004541
16:32600808	16	32600808	A	6	21	T	0.0005959
rs199804107	16	33550174	C	2	14	T	0.0009649
16:35252531	16	35252531	C	1	13	T	0.0003716
16:35252542	16	35252542	G	1	13	A	0.0003716
rs374642173	16	35252546	T	1	13	G	0.0003716
16:35252549	16	35252549	C	1	13	G	0.0003716
16:35252556	16	35252556	G	1	13	A	0.0003716
rs12935596	16	48841681	T	7	22	C	0.0009169
16:52901303	16	52901303	G	0	10	A	0.0004292
rs202037976	16	56161190	G	20	40	A	0.0003188
rs9921494	16	72873537	A	3	16	C	0.0009912
rs200106830	16	72901631	A	4	17	AAAAAG	0.0008872
16:73093273	16	73093273	G	15	0	A	0.00003523
rs2735407	16	78049430	C	25	45	G	0.0002688

rs2707610	16	78049465	T	25	45	C	0.0002688
rs7192716	16	78049521	T	25	45	A	0.0002688
rs2707608	16	78050219	T	25	43	C	0.0008649
rs2072665	16	78065488	C	41	17	T	0.0005984
rs7188702	16	78065673	T	41	17	A	0.0005984
rs11859824	16	78660716	A	8	24	G	0.0006758
rs529223	16	83874823	A	29	9	C	0.0008145
rs3935715	16	85199237	A	18	3	T	0.0009486
rs143574208	16	85509419	C	1	12	CG	0.0007887
rs9933468	16	85572662	G	39	16	C	0.0009032
rs3934891	16	85573772	T	39	16	A	0.0009032
rs4072360	16	85574528	A	39	16	T	0.0009032
rs9932681	16	85575053	C	39	16	T	0.0009032
rs78429171	16	85575718	G	39	16	GT	0.0009032
16:86299406	16	86299406	A	0	11	C	0.0001895
rs1687632	16	86375805	C	2	14	G	0.0009649
rs1687631	16	86375941	A	2	14	G	0.0009649
rs1728419	16	86376876	T	2	14	A	0.0009649
rs3103628	16	86377793	A	2	14	G	0.0009649
rs3103629	16	86377799	A	2	14	T	0.0009649
rs1918824	16	86378264	G	2	14	C	0.0009649
rs1918822	16	86378369	A	2	14	T	0.0009649
rs1728377	16	86378844	T	2	14	C	0.0009649
rs1728375	16	86379460	A	2	14	G	0.0009649
rs1728374	16	86379467	A	2	14	G	0.0009649
rs1619229	16	86379556	A	2	14	G	0.0009649
rs1687629	16	86380283	G	2	14	A	0.0009649
rs1687628	16	86380293	T	2	14	C	0.0009649
rs1728373	16	86380294	G	2	14	A	0.0009649
rs1687627	16	86380421	A	2	14	G	0.0009649
rs8058345	16	86380906	A	2	14	G	0.0009649
rs6540288	16	86380943	T	2	14	A	0.0009649
rs2451862	16	86380964	C	2	14	A	0.0009649
rs2451861	16	86381006	C	2	14	G	0.0009649
rs2463604	16	86381016	C	2	14	T	0.0009649
rs2463603	16	86381023	A	2	14	G	0.0009649
rs7403918	16	86828794	G	14	32	A	0.0004925
rs9940109	16	87266245	A	19	37	C	0.0009069
16:87304715	16	87304715	T	9	28	C	0.0001391
rs113591197	16	89108636	A	0	10	G	0.0004292
rs8058269	16	89110920	C	0	10	T	0.0004292

rs74034553	16	89114993	G	0	10	T	0.0004292
rs9806827	16	89115688	C	0	10	A	0.0004292
rs74034555	16	89116047	A	0	10	G	0.0004292
rs61596186	16	89116584	T	0	10	C	0.0004292
rs111390307	16	89116980	C	0	10	T	0.0004292
rs112273493	16	89117342	G	0	10	C	0.0004292
rs74034558	16	89118492	T	0	10	C	0.0004292
rs74034563	16	89122432	A	0	10	G	0.0004292
rs149078245	16	89123179	T	0	10	TG	0.0004292
rs74034564	16	89123539	T	0	10	C	0.0004292
rs74719380	16	89125848	A	0	10	G	0.0004292
rs188435427	16	89126381	A	0	10	G	0.0004292
rs117769809	16	89129569	T	0	10	C	0.0004292
rs112693041	16	89130022	T	0	10	C	0.0004292
rs117098882	16	89132011	C	0	10	T	0.0004292
rs75167441	16	89378653	T	15	1	C	0.0003561
rs10454069	16	89380666	G	1	13	C	0.0003716
rs35359504	17	989967	G	49	23	C	0.0005083
17:2789470	17	2789470	A	0	9	T	0.0008435
rs17727437	17	5936617	C	0	11	T	0.0001895
rs117076215	17	6300167	T	16	2	C	0.0008956
rs115074709	17	6312274	T	16	2	C	0.0008956
rs4581751	17	6312668	T	22	4	C	0.00043
17:6818065	17	6818065	A	1	14	C	0.0001881
rs117819652	17	9346012	G	0	9	A	0.0009641
17:10053738	17	10053738	T	0	14	C	0.00001855
rs11078044	17	11808491	T	5	19	C	0.000809
rs62061375	17	14899443	T	15	35	C	0.0001708
rs11653343	17	14910499	C	9	25	T	0.0008942
rs4539645	17	14923255	A	20	38	G	0.0009963
rs142548624	17	14924062	A	19	37	ATAAAT	0.0009069
rs79908869	17	15275001	C	1	12	T	0.0007887
17:16677443	17	16677443	G	16	2	T	0.0008956
rs112992326	17	18223822	C	1	13	G	0.0003716
17:18291344	17	18291344	ATATATATATATA(	24	6	A	0.0009794
rs4452012	17	18562691	G	53	26	C	0.0003309
rs4441330	17	18562720	C	54	27	T	0.0003437
rs369619837	17	19571912	G	26	7	A	0.0007902
rs76119305	17	19605569	C	2	14	T	0.0009649
rs157386	17	19699121	A	26	6	G	0.0003002
17:19729309	17	19729309	AT	36	13	A	0.0001386

rs60573446	17	19729312	AT	36	13	A	0.0001217
rs407990	17	19736262	G	34	10	C	0.0001698
rs281357	17	19742514	T	35	13	C	0.000996
rs200473841	17	21560878	C	11	0	T	0.0007855
rs34067029	17	29871409	G	55	28	A	0.0005409
rs1109956	17	35216997	T	17	35	C	0.000674
rs60117359	17	43060051	G	23	5	T	0.0006807
rs4793944	17	46667304	T	48	22	C	0.0004783
rs10597590	17	46746068	TCTC	15	33	T	0.0005689
rs34002327	17	46763580	A	6	22	G	0.000342
rs112734318	17	46764463	A	45	19	G	0.0004142
rs188869607	17	46764522	T	54	22	A	0.00003472
rs10445381	17	46766306	G	54	23	A	0.00002242
rs12941331	17	46769069	T	6	22	C	0.000342
rs8067554	17	46769270	T	6	22	A	0.000342
rs3096643	17	46769448	T	53	23	G	0.00004378
rs4793971	17	46770113	T	6	22	C	0.000342
rs8182366	17	46771366	A	48	21	T	0.0002205
rs10528381	17	46772982	C	51	21	CCTTTT	0.00003225
rs61102347	17	46774809	C	27	47	A	0.0002837
rs4793972	17	46775005	C	7	22	T	0.0009169
rs112609688	17	46775760	C	53	23	T	0.00004378
rs4581744	17	46776341	A	27	50	G	0.00004066
rs113038184	17	46777791	T	51	24	A	0.0003012
rs62065362	17	46778079	T	51	22	G	0.00007816
rs4793973	17	46780151	A	5	20	G	0.0004383
rs4793974	17	46780300	C	12	30	T	0.0005652
rs9897429	17	47518378	G	27	46	A	0.0005154
rs57112168	17	47994255	A	17	35	C	0.0007327
rs56011844	17	47994267	G	17	35	C	0.0007327
rs8066067	17	49407790	C	13	0	A	0.0001783
rs8068355	17	49435579	C	46	21	T	0.0007859
rs9807015	17	49435657	C	46	21	T	0.0007859
rs7208147	17	49437720	G	46	21	C	0.0007859
rs4794236	17	49438873	G	46	21	A	0.0007859
rs4794238	17	49439280	T	46	21	C	0.0007859
rs758520	17	49441127	A	46	21	C	0.0007859
rs2013281	17	49441641	T	46	21	A	0.0007859
rs9903614	17	49442178	G	46	21	T	0.0007859
rs758521	17	49442332	C	46	21	T	0.0007859
rs736757	17	49442630	A	46	21	G	0.0007859

rs736758	17	49442821	T	46	21	C	0.0007859
rs4794241	17	49445074	G	45	21	A	0.0007429
rs4794242	17	49447214	G	46	21	A	0.0007859
rs140520931	17	49480129	CT	5	19	C	0.0008415
rs11652255	17	55468446	A	16	2	G	0.0008956
rs72833004	17	55486740	A	25	6	G	0.0005533
rs62058122	17	55692602	T	1	12	C	0.0007887
rs201550481	17	67961542	T	18	3	C	0.0009486
rs199538105	17	74948103	AG	49	20	A	0.0002086
rs75280128	17	75726185	T	11	0	A	0.0007855
rs4789007	17	75849501	T	14	1	C	0.0007094
rs139586625	17	75916547	C	16	1	T	0.0001772
rs79095850	17	75922079	T	15	1	C	0.0003561
rs60249275	17	76024170	G	47	22	A	0.0008379
rs12103535	17	76500652	T	46	21	C	0.0007859
rs72849844	17	78273610	T	14	0	C	0.00008428
rs117459315	17	78273990	T	14	0	C	0.00008428
rs72849848	17	78275156	G	14	0	A	0.00008428
rs72849850	17	78278663	T	14	0	C	0.00008428
rs112599480	17	78279689	A	14	0	G	0.00008428
rs66578743	18	10679	C	14	29	A	0.0005934
rs12957723	18	325884	A	2	16	G	0.0002312
rs143366568	18	2490684	A	37	14	AAAAAAT	0.0006739
rs2644197	18	2492423	G	53	20	A	0.00000429
rs16943352	18	2495530	A	12	32	G	0.0001005
rs12457488	18	2496329	T	12	32	G	0.0001005
rs17533695	18	2496404	C	42	16	T	0.0001653
rs770226	18	2497437	C	42	16	T	0.0001653
rs770227	18	2497443	C	12	32	T	0.0001005
rs12458240	18	2497517	A	12	32	G	0.0001005
rs5822707	18	2497914	CCACTT	12	32	C	0.0001005
rs12454088	18	2498581	G	12	32	C	0.0001005
rs12458998	18	2498726	A	12	32	G	0.0001005
rs9955413	18	3099402	C	25	43	T	0.0008649
rs7232329	18	3100429	A	25	43	G	0.0008649
rs11081005	18	3101470	T	25	43	C	0.0008649
rs9953889	18	4087117	C	31	48	T	0.0008872
rs1658169	18	4491413	G	13	31	A	0.0004205
rs190474398	18	5499832	G	22	4	A	0.0004962
rs9945119	18	10035436	T	42	18	C	0.0006782
rs625395	18	10036145	T	43	18	C	0.0003905

rs613223	18	10036551	C	43	18	T	0.0003905
rs518096	18	10037213	T	43	18	C	0.0003905
rs517854	18	10037294	C	43	18	T	0.0003905
rs487857	18	10038210	A	42	18	G	0.0006782
rs569812	18	10039025	G	43	18	C	0.0003905
rs568855	18	10039133	G	43	18	C	0.0003905
rs67716474	18	10039259	TG	43	18	T	0.0003905
rs1292308	18	10039331	G	42	18	A	0.0006782
rs565991	18	10039453	A	43	18	G	0.0003905
rs539466	18	10040066	G	42	18	A	0.0006782
rs535823	18	10040445	A	43	18	G	0.0003905
rs541164	18	10041354	G	43	18	C	0.0003905
rs541853	18	10041374	T	42	18	C	0.0006782
rs644778	18	10042023	C	43	18	T	0.0003905
rs572197	18	10042399	A	43	18	G	0.0003905
rs631448	18	10042744	T	42	18	A	0.0006782
rs106763	18	10043959	T	42	18	C	0.0006782
rs1985509	18	10045562	C	52	23	T	0.00008358
rs663130	18	10046837	C	52	23	T	0.00008358
rs647866	18	10047937	G	50	23	A	0.0002847
rs529225	18	10049909	C	50	24	G	0.0005368
rs1787086	18	35309697	G	30	10	T	0.0005063
18:35309700	18	35309700	CGATCTGTCTAT	37	13	C	0.0003209
rs72898715	18	35919503	C	14	1	T	0.0007094
rs11877700	18	37842775	C	12	0	T	0.0003752
rs9949334	18	37844766	C	12	0	T	0.0003752
rs9945673	18	37850426	G	12	0	A	0.0003752
rs35670038	18	37853117	GA	11	0	G	0.0007855
rs72904819	18	37942560	A	11	29	G	0.0004498
rs7240539	18	37951056	T	14	32	C	0.0004925
rs11662516	18	37952153	T	11	29	C	0.0004498
rs11659339	18	37958898	C	11	29	T	0.0004498
rs72904871	18	37959980	T	11	29	G	0.0004498
rs72904878	18	37961767	A	11	29	G	0.0004498
rs56058513	18	37968788	G	11	29	A	0.0004498
rs72890039	18	37973929	T	11	29	C	0.0004498
rs203156	18	38044314	T	30	48	A	0.0009571
rs203191	18	38054299	A	30	49	G	0.0005383
rs169395	18	38054353	G	30	49	T	0.0005383
rs61083958	18	38055471	A	30	48	AAGAATGTGATT[illegible]	0.0009571
rs55562	18	38057808	T	28	48	C	0.0004211

rs61371567	18	38058683	AT	30	49 A	0.0005383
rs203162	18	38059386	C	30	49 T	0.0005383
rs203172	18	38068321	T	30	49 C	0.0005383
rs4132081	18	38084525	C	30	50 T	0.0002951
rs4291983	18	38106410	C	31	50 A	0.0005409
rs9956350	18	38107168	G	31	50 A	0.0005409
rs7227822	18	38107825	C	31	50 G	0.0005409
rs58857930	18	38118024	GA	6	21 G	0.0006518
rs8091417	18	38124303	T	31	50 C	0.0005409
rs112024034	18	39253947	G	7	25 GA	0.0001343
rs10502774	18	39254020	C	2	14 A	0.0009649
rs1865290	18	39255558	A	2	14 T	0.0009649
rs1865289	18	39255600	C	2	14 T	0.0009649
rs8092873	18	39256336	G	3	16 A	0.0009912
rs8093033	18	39256431	G	7	25 A	0.0001343
rs11082244	18	39257033	G	2	14 A	0.0009649
rs1865288	18	39257639	T	3	17 C	0.0005045
rs11873827	18	39261692	C	8	26 G	0.0001919
rs1433931	18	39277952	A	4	18 G	0.0009242
rs7234764	18	39280705	A	8	26 G	0.0001919
rs9304252	18	39282873	A	2	16 T	0.0002312
rs7240040	18	39283106	C	2	16 A	0.0002312
rs1368835	18	39285384	G	2	15 A	0.0004755
rs2314979	18	39287420	C	2	15 T	0.0004755
rs11876458	18	39289536	G	8	25 A	0.0003633
rs4890212	18	39290372	T	2	15 C	0.0004755
rs200786741	18	39782725	G	22	4 GT	0.000223
18:40507336	18	40507336	T	20	45 TGTGTGTG	0.000007324
18:48853562	18	48853562	A	18	3 T	0.0009486
rs367904418	18	48853566	A	23	3 T	0.00006268
rs9946188	18	62202275	T	27	45 G	0.0009136
rs8085758	18	62202713	T	27	45 A	0.0009136
rs377373310	18	62770130	T	12	0 A	0.0004093
rs10871601	18	64364171	G	0	9 A	0.0008435
18:66093134	18	66093134	T	0	10 A	0.0004292
18:66093135	18	66093135	A	0	10 T	0.0004292
rs60749094	18	73824642	G	51	22 C	0.00007816
rs76470163	18	73824644	G	52	23 C	0.00008358
rs62094675	18	73824646	G	53	24 C	0.00008869
rs4890830	18	75257146	T	27	7 G	0.0004381
rs544752	18	77535939	A	13	31 G	0.0004205

rs12965930	18	77676057	T	11	0	C	0.0008455
rs56357228	19	2540253	A	16	2	G	0.0008956
rs71337146	19	2540372	T	16	2	TCTC	0.0008956
rs12977761	19	2540449	C	16	2	T	0.0008956
rs67768503	19	2542552	T	19	3	C	0.0005082
rs35511982	19	2542652	T	19	3	C	0.0005082
rs6510682	19	2545570	G	15	1	A	0.0003561
rs75020370	19	3090719	T	11	28	C	0.0008103
rs6510736	19	3165974	A	2	16	G	0.0002312
rs112301049	19	4300958	AGTTTTT	6	21	A	0.0006518
19:4885160	19	4885160	T	18	3	C	0.0009486
rs10419597	19	7125915	A	3	16	G	0.0009912
rs10408374	19	7127294	T	3	16	C	0.0009912
rs8106714	19	7130686	A	3	16	G	0.0009912
rs8111012	19	7130731	G	3	16	T	0.0009912
rs71873129	19	7130785	C	3	16	CCT	0.0009912
rs75930593	19	7131187	A	3	16	G	0.0009912
rs11878560	19	7131323	C	3	16	T	0.0009912
rs11883285	19	7131380	G	3	16	A	0.0009912
rs2860175	19	7132081	T	3	16	C	0.0009912
rs8112793	19	7288790	A	4	18	G	0.0009242
rs1864009	19	7294931	A	7	23	C	0.0004919
rs11880337	19	7296452	C	7	22	T	0.0009169
rs7507911	19	7298095	T	7	22	C	0.0009169
rs7250537	19	7298723	A	7	22	G	0.0009169
rs41285780	19	7974865	A	0	10	G	0.0004292
rs62117528	19	8486345	T	15	35	C	0.0002806
rs7249094	19	8672000	A	23	43	G	0.0002487
rs7253116	19	8672076	A	24	43	C	0.000471
rs7248613	19	9138317	C	49	22	T	0.0002673
rs11673664	19	9140304	G	47	22	A	0.0008379
rs12720356	19	10469975	C	17	2	A	0.000467
rs2569543	19	11236121	C	12	0	G	0.0004093
rs2569538	19	11238548	A	12	0	G	0.0003752
rs4804567	19	11245472	T	12	0	C	0.0003752
rs7256793	19	11247081	T	12	0	C	0.0003752
rs8105087	19	13250340	T	30	8	A	0.0001859
19:13327476	19	13327476	T	11	29	TC	0.0002293
19:13327478	19	13327478	T	11	29	TATCTATCTATCT/	0.0002293
rs34800714	19	17333532	C	14	1	CA	0.0008899
rs140021996	19	20097971	A	0	9	T	0.0008243

19:29281257	19	29281257	A	14	1	G	0.0006479
rs74994260	19	33638586	G	0	11	C	0.0001895
rs115215688	19	33639422	G	0	12	C	0.00008299
rs2918333	19	36750539	A	0	11	G	0.0001895
rs12985428	19	39414109	A	22	41	G	0.0004309
rs57362195	19	39414613	C	23	41	CA	0.0008021
rs77152874	19	40288189	T	19	3	C	0.0009229
rs10411681	19	48243509	A	34	12	G	0.000839
rs2011339	19	48245780	T	34	12	C	0.000839
rs968579	19	48246401	G	34	12	C	0.000839
rs3826832	19	48247639	G	34	12	A	0.000839
rs11671367	19	48250714	G	35	12	T	0.0004754
rs3745754	19	48253579	A	34	12	G	0.000839
rs10402957	19	48254392	T	34	12	C	0.000839
rs5828313	19	48256785	AC	34	12	A	0.000839
rs10422531	19	48256982	A	34	12	G	0.000839
rs10406879	19	48258551	T	34	12	C	0.000839
rs889142	19	48260079	T	34	12	C	0.000839
rs34134057	19	48265657	G	38	15	T	0.0007851
rs34934272	19	48265660	C	38	15	T	0.0007851
rs34679717	19	48270613	TC	34	12	T	0.000839
rs10412990	19	48273401	T	34	12	C	0.000839
rs1035449	19	48274041	T	34	12	C	0.000839
rs10425789	19	48275098	G	34	12	C	0.000839
rs10414783	19	48277208	G	34	12	C	0.000839
rs10414443	19	48277301	A	34	12	G	0.000839
rs11083897	19	48279789	G	34	12	A	0.000839
rs11670990	19	48279901	C	34	12	T	0.000839
rs2334297	19	48280932	C	34	12	G	0.000839
rs2334298	19	48280978	G	34	12	A	0.000839
rs3815751	19	48282316	G	34	12	A	0.000839
rs200521388	19	48488329	A	0	10	AAAAAAAG	0.0004778
19:48492369	19	48492369	T	2	20	TA	0.00001141
19:48492371	19	48492371	T	2	20	TTA	0.00001233
rs7247872	19	52925727	G	7	22	A	0.0009169
rs7247880	19	52925743	G	7	22	A	0.0009169
rs4802933	19	52925820	A	7	22	G	0.0009169
rs4802934	19	52925832	G	7	22	A	0.0009169
rs369042987	19	53049033	G	6	20	GA	0.0005097
rs78681110	19	54931444	A	26	7	G	0.0007902
rs7259090	19	55392646	A	5	19	G	0.0008415

rs139784954	19	55412459	G	0	9	A	0.0009641
19:55608122	19	55608122	C	18	1	CCGCAGTTCACCA	0.00004235
19:59013833	19	59013833	G	24	6	GAGTAGTGAGTG.	0.0009794
20:530314	20	530314	GAAAGAAAT	0	9	G	0.0009641
rs16986637	20	2515179	A	15	1	C	0.0003561
rs6083577	20	2518238	G	15	1	C	0.0003561
rs6114822	20	2520342	T	15	1	C	0.0003561
rs6114849	20	2524120	G	15	1	A	0.0003561
rs6083611	20	2528940	G	15	1	A	0.0003561
rs78953454	20	3439535	G	4	18	C	0.0009242
rs11087590	20	3594461	CTTTTTTAAATGA(	0	16	C	0.000003438
rs35560557	20	4834894	A	18	3	G	0.0009486
rs7260714	20	6918462	C	46	21	T	0.0007859
rs974100	20	6923401	C	46	21	T	0.0007859
rs974099	20	6923536	T	46	21	C	0.0007859
rs6038994	20	7954930	A	18	3	G	0.0009486
rs7270019	20	7955347	G	18	3	A	0.0009486
rs28416521	20	11991252	A	39	13	G	0.0001767
rs6134432	20	11991888	G	46	19	A	0.0002121
rs2057051	20	11995353	G	44	18	A	0.0002208
rs6131294	20	11995533	A	44	18	G	0.0002208
rs13042149	20	11996643	T	46	21	A	0.0007859
rs6131296	20	11996720	T	44	18	C	0.0002208
rs11699987	20	11996731	G	44	18	C	0.0002208
rs4814057	20	11997323	G	46	21	A	0.0007859
rs35113459	20	11997518	A	44	18	AAT	0.0002208
rs34813755	20	11999401	A	45	18	C	0.0001933
rs73615533	20	11999580	T	44	18	TAGAG	0.0002208
rs35172532	20	11999901	C	44	18	CTG	0.0002208
rs6134436	20	12001080	T	44	18	C	0.0002208
rs6041035	20	12002029	C	46	21	T	0.0007859
rs4814059	20	12002915	G	44	18	A	0.0002208
rs7344790	20	12004394	T	45	18	C	0.0001933
rs7270764	20	12007223	T	41	12	C	0.00001087
20:13179440	20	13179440	T	50	17	TC	0.00000595
20:13179441	20	13179441	T	50	17	C	0.000006748
20:13179442	20	13179442	T	50	17	TTTCTTTC	0.000006748
20:13179448	20	13179448	T	44	15	TC	0.00003811
20:13179449	20	13179449	T	44	15	C	0.00003811
20:13711350	20	13711350	AT	20	1	A	0.00002287
rs68012295	20	13754717	A	11	28	G	0.0008103

rs6079176	20	13780386	A	10	27	G	0.0006386
rs149543954	20	15302488	T	0	10	G	0.0006298
rs6079925	20	15654234	C	40	14	G	0.0001172
rs7362631	20	15656143	C	23	43	T	0.0002487
rs7508866	20	19006113	C	0	9	A	0.0009641
rs139749069	20	19015804	C	0	9	T	0.0009641
rs199732390	20	19112927	T	54	26	TA	0.0008138
rs55952965	20	19112931	T	53	26	TTTG	0.0007605
rs201489682	20	20163809	T	18	36	C	0.0008188
rs2424350	20	20291451	G	9	26	A	0.0004896
rs6075643	20	20298392	A	9	25	G	0.0008942
rs11908058	20	20320836	A	14	33	G	0.0002703
rs6036237	20	22838125	T	17	35	A	0.0007327
rs6048387	20	22840461	T	14	35	A	0.00007667
20:24416229	20	24416229	A	1	13	G	0.0003716
rs73609198	20	29543279	G	0	9	C	0.0009641
rs71189955	20	38089215	CA	9	25	C	0.0009353
rs141267243	20	40060527	A	0	9	G	0.0009641
rs78132140	20	40124285	A	0	9	C	0.0009641
rs3091450	20	42397496	T	6	22	C	0.000342
rs140589268	20	47594416	T	0	9	C	0.0009641
rs113924526	20	47599194	T	0	10	C	0.0004292
rs111589018	20	49449156	C	1	12	G	0.0007887
rs8113870	20	54723247	C	9	25	T	0.0008942
rs74945693	20	54728926	C	0	9	T	0.0009641
rs3044074	20	54730860	CTCTG	1	12	C	0.0007887
rs6024644	20	54731194	C	0	9	T	0.0009641
rs113237261	20	54731908	TA	0	9	T	0.0009641
rs6099014	20	54732154	C	0	9	T	0.0009641
rs6014614	20	54733628	T	0	9	C	0.0009641
rs6024645	20	54736770	T	0	9	C	0.0009641
rs11348175	20	54737714	C	0	9	CT	0.0009641
rs6024648	20	54739879	G	0	9	A	0.0009641
rs11906763	20	54741180	C	0	9	A	0.0009641
rs77061313	20	54744376	C	0	9	A	0.0009641
rs73177764	20	55654497	T	10	27	A	0.0006386
rs6069979	20	55661549	A	26	44	G	0.0008911
rs6099453	20	55661811	A	27	45	C	0.0009136
rs73145227	20	57872527	C	0	9	T	0.0009641
rs28688816	20	59937912	A	5	19	G	0.0008415
rs3936160	20	59938278	A	5	19	G	0.0008415

rs28551468	20	59938740	G	11	28	A	0.0008103
rs4074394	20	59940447	A	7	22	C	0.0009169
rs28535181	20	59941064	A	7	22	G	0.0009169
rs73134330	20	59942013	C	7	22	T	0.0009169
rs34134614	20	59942301	T	7	22	TGA	0.0009169
rs28839264	20	59943881	G	7	22	A	0.0009169
rs28860773	20	59943935	G	7	22	A	0.0009169
20:61398964	20	61398964	G	0	13	GA	0.00003362
20:61398965	20	61398965	AGAGAGAGAAAG	0	13	A	0.00003362
rs370856873	21	9411732	G	0	10	T	0.0004292
rs376015153	21	9411998	C	0	10	T	0.0004292
rs368544187	21	9412105	T	0	9	A	0.0009641
rs374619179	21	9416098	A	0	9	T	0.0009641
rs375752645	21	9425655	C	0	10	T	0.0004292
rs369399988	21	9425841	T	0	10	C	0.0004292
rs373254513	21	9453615	C	0	9	T	0.0009641
rs375903391	21	9476421	G	0	10	A	0.0004292
rs371571587	21	9503111	G	0	9	A	0.0009641
rs369519916	21	9570920	T	0	9	A	0.0009641
rs375391151	21	9594353	T	0	10	A	0.0004292
21:10085464	21	10085464	A	1	12	T	0.0007887
21:10596993	21	10596993	G	32	52	T	0.0001819
rs2821885	21	14868113	T	1	13	G	0.0003716
rs2775606	21	14868962	G	2	14	A	0.0009649
rs1739233	21	14870697	T	1	13	C	0.0003716
rs1997577	21	16371102	T	4	19	A	0.0004818
rs7280064	21	16377650	T	4	19	C	0.0004818
rs139790892	21	16377682	GC	4	19	G	0.0004818
rs2823006	21	16380994	C	4	19	G	0.0004818
rs28574812	21	16384940	G	5	20	A	0.0004383
rs145267297	21	16391634	A	5	19	AAGAT	0.0008415
rs11909793	21	21821153	A	16	2	T	0.0008956
rs57396480	21	24947685	T	4	18	TA	0.0009446
rs8134027	21	31835840	A	2	14	G	0.0009649
rs2298436	21	31864307	G	2	14	T	0.0009649
rs9983828	21	32630308	C	27	48	T	0.0001523
rs117407002	21	34530659	G	0	11	A	0.0001895
rs3787870	21	39285453	G	53	24	A	0.00008869
rs12329791	21	39646635	T	23	5	C	0.0006807
rs2836269	21	39648790	A	23	5	G	0.0006807
rs2836277	21	39652400	A	23	5	G	0.0006807

rs8129961	21	39653928	T	23	5	C	0.0006807
rs2836285	21	39656505	C	23	5	T	0.0006807
rs2836286	21	39657252	T	23	5	C	0.0006807
rs689400	21	40916094	T	22	4	C	0.00043
rs2837074	21	40919955	A	22	4	G	0.00043
rs657833	21	40920903	T	22	4	C	0.00043
rs568840	21	40926000	T	22	4	C	0.00043
rs2989335	21	40927346	C	23	4	A	0.0002248
rs644080	21	40934470	G	22	4	A	0.00043
rs571227	21	40935641	G	22	4	C	0.00043
rs626856	21	40936014	C	22	4	A	0.00043
rs613758	21	40936636	A	22	4	C	0.00043
rs492140	21	40936776	G	22	4	T	0.00043
rs611958	21	40937071	A	22	4	T	0.00043
rs516865	21	40937191	C	22	4	T	0.00043
rs611039	21	40937280	A	22	4	C	0.00043
rs141986742	21	40937368	GTA	19	3	G	0.0004337
rs112619200	21	40937832	AC	28	4	A	0.000007156
rs523372	21	40937929	G	28	4	T	0.000007156
21:40938042	21	40938042	AT	28	4	A	0.000007156
21:40938043	21	40938043	GACGTAGAAAT	28	4	G	0.000007156
rs596320	21	40938269	C	28	4	T	0.000007156
rs550774	21	40938562	C	28	4	T	0.000007156
rs551624	21	40938643	T	28	4	C	0.000007156
rs551830	21	40938715	T	28	4	C	0.000007156
rs503729	21	40939493	C	28	4	G	0.000007156
rs506387	21	40939762	T	28	4	C	0.000007156
rs658066	21	40940223	A	28	4	G	0.000007156
rs646823	21	40940402	A	23	4	T	0.00009765
rs646320	21	40940532	G	33	11	A	0.0006951
rs538333	21	40940939	C	27	4	A	0.00001464
rs561166	21	40941132	G	28	4	T	0.000007156
rs2837083	21	40941250	A	28	4	G	0.000007156
rs59155174	21	40941274	G	28	4	A	0.000007156
rs562285	21	40941308	G	28	4	C	0.000007156
rs563230	21	40941401	T	28	4	C	0.000007156
rs563259	21	40941412	T	28	4	C	0.000007156
rs564126	21	40941498	T	27	4	C	0.00001464
rs630315	21	40941847	T	33	11	C	0.0006951
rs630270	21	40941891	G	28	4	A	0.000007156
rs567837	21	40941914	G	28	4	C	0.000007156

rs629432	21	40942040	G	27	4	A	0.00001464
rs628995	21	40942130	A	27	3	T	0.000003441
rs617259	21	40942493	T	27	4	C	0.00001464
rs602107	21	40943606	A	27	4	T	0.00001464
rs601732	21	40943636	A	31	10	G	0.0006062
rs542531	21	40944237	A	27	4	C	0.00001464
rs34797170	21	40944570	C	27	4	CT	0.00001464
rs585092	21	40945028	T	27	4	G	0.00001464
rs376474175	21	40945600	G	23	3	GTA	0.00006268
rs1734930	21	40946124	A	27	4	G	0.00001464
rs636599	21	40947315	C	27	4	G	0.00001464
rs141072016	21	40947736	C	25	4	CACAT	0.00005893
rs631454	21	40953128	C	27	5	T	0.00005212
rs551680	21	40954708	A	27	5	G	0.00005212
rs665778	21	40956847	A	27	5	C	0.00005212
rs526526	21	40957080	G	27	5	A	0.00005212
rs527343	21	40957134	A	27	5	C	0.00005212
rs651607	21	40957728	T	27	5	C	0.00005212
rs650633	21	40957936	T	27	5	C	0.00005212
rs542574	21	40960292	A	27	5	G	0.00005212
rs606414	21	40960892	T	27	5	C	0.00005212
rs606003	21	40960984	T	27	5	C	0.00005212
rs578349	21	40961915	G	27	5	T	0.00005212
rs523893	21	40963407	A	26	5	G	0.0001012
rs11356190	21	40963510	A	13	34	AT	0.0001142
rs8128025	21	41413744	T	42	18	C	0.0006782
21:42820877	21	42820877	GCC	12	32	G	0.0001005
rs56232589	21	43023699	T	5	19	C	0.0008415
rs10154196	21	43024004	A	5	19	G	0.0008415
rs9285727	21	43026175	G	5	19	C	0.0008415
rs79348973	21	43034276	G	0	9	A	0.0009641
rs7284011	21	43913983	G	7	23	C	0.0004919
rs2051329	21	43922997	C	7	23	T	0.0004919
rs73233025	21	45894462	C	0	12	T	0.00008299
rs73233026	21	45897036	G	0	12	T	0.00008299
rs73233027	21	45897827	G	0	11	T	0.0001895
rs146490633	21	45899411	C	0	11	G	0.0001895
rs73233031	21	45917661	A	0	10	G	0.0004292
rs62214403	21	46664698	A	11	0	G	0.0007855
rs145556652	21	47747302	T	23	41	TTC	0.0008021
rs2839318	21	47966810	C	29	9	G	0.0008145

22:17703382	22	17703382	G	8	30	A	0.000004461
22:17703392	22	17703392	A	8	28	ATATATATATATA	0.00002611
rs376288107	22	18791397	T	0	9	C	0.0009641
rs6005336	22	21391646	A	24	42	G	0.0008352
rs6519738	22	21395756	A	27	46	T	0.0005154
rs7291070	22	21395757	A	27	46	G	0.0005154
rs6519739	22	21395784	C	27	46	T	0.0005154
rs7287719	22	21395811	G	27	46	A	0.0005154
rs5762046	22	21396651	T	28	47	A	0.0005255
rs4586741	22	22740278	G	0	9	A	0.0009641
rs8137706	22	27773685	T	3	19	C	0.0001254
rs9614870	22	45069410	T	1	13	C	0.0003716
rs9626231	22	45072227	A	1	12	C	0.0007119
rs9626508	22	45073584	A	1	12	G	0.0007887
rs9614873	22	45075015	G	1	12	C	0.0007887
rs9614874	22	45075406	T	1	12	G	0.0007887
rs9626514	22	45075854	A	1	12	G	0.0007887
rs9626515	22	45075871	T	1	12	C	0.0007887
rs9626232	22	45075876	A	1	12	G	0.0007887
rs9614553	22	45076044	A	1	12	G	0.0007887
rs9614554	22	45076061	C	1	12	G	0.0007887
rs9614555	22	45076280	T	1	12	G	0.0007887
rs9614875	22	45076306	T	1	12	A	0.0007887
rs59936734	22	45076613	CA	1	12	C	0.0007887
rs9614558	22	45077861	T	1	12	C	0.0007887
rs9614878	22	45078177	A	1	12	G	0.0007887
rs62230575	22	45078240	G	1	12	T	0.0007887
rs13054881	22	45078354	A	1	12	G	0.0007887
rs34504565	22	45078443	A	1	12	G	0.0007887
rs9626517	22	45078460	A	1	12	G	0.0007887
rs9626518	22	45078496	A	1	12	G	0.0007887
rs9626519	22	45078517	A	1	12	C	0.0007887
rs9626520	22	45078578	T	1	12	C	0.0007887
rs9626521	22	45078601	C	1	12	T	0.0007887
rs9626522	22	45078618	T	1	12	C	0.0007887
rs9626523	22	45078742	A	1	12	G	0.0007887
rs56692566	22	45079237	C	1	12	T	0.0007887
rs60280946	22	45079484	C	1	12	CTG	0.0007887
rs12165506	22	45079804	G	1	12	A	0.0007887
rs12169183	22	45079808	G	1	12	C	0.0007887
rs12169228	22	45079901	T	1	12	C	0.0007887

rs12169229	22	45079902	G	1	12	C	0.0007887
rs28376449	22	45079927	G	1	12	C	0.0007887
rs12170468	22	45079980	A	1	12	G	0.0007887
rs12170556	22	45080124	A	1	12	G	0.0007887
rs12169345	22	45080188	T	1	12	C	0.0007887
rs12169373	22	45080205	T	1	12	C	0.0007887
rs12167343	22	45080483	G	1	12	T	0.0007887
rs9614879	22	45080502	G	1	12	A	0.0007887
rs9626524	22	45080508	A	1	12	G	0.0007887
rs9626525	22	45080543	G	1	12	T	0.0007887
rs9626526	22	45080649	G	1	12	A	0.0007887
rs12168856	22	45080989	C	1	12	T	0.0007887
rs41396744	22	45081259	G	1	12	C	0.0007887
rs41515447	22	45081330	G	1	12	A	0.0007887
rs62230599	22	45081398	C	1	12	G	0.0007887
rs62230600	22	45081571	A	1	12	G	0.0007887
rs9627100	22	46582741	A	0	9	G	0.0009641
rs9626751	22	46582838	A	0	9	G	0.0009641
rs62225956	22	46584299	A	0	9	C	0.0009641
rs62225957	22	46584370	G	0	9	C	0.0009641
rs62225958	22	46591725	A	0	10	G	0.0004292
rs59223524	22	46591845	A	0	10	G	0.0004292
rs4253709	22	46594750	T	2	15	G	0.0004755
rs17616262	22	48183889	T	20	40	G	0.0003188
22:49754905	22	49754905	T	16	2	C	0.0008956
X:200525	23	200525	C	16	33	T	0.000596
X:743185	23	743185	C	36	13	T	0.0006334
X:743226	23	743226	T	34	13	C	0.000922
X:876870	23	876870	C	48	23	G	0.0008881
X:1817878	23	1817878	G	0	10	GTAATA	0.0004292
X:2388892	23	2388892	C	2	14	T	0.0009649
X:2388923	23	2388923	C	3	16	T	0.0009912
X:2388955	23	2388955	C	3	16	CTTGA	0.0009912
rs6642024	23	2723666	C	14	32	T	0.0004925
rs11797301	23	2723715	G	10	27	T	0.0006386
rs2694739	23	3127599	T	41	17	C	0.0009864
rs199795850	23	3127935	CTCTA	41	17	C	0.0005984
rs2704885	23	3128043	C	41	17	T	0.0005984
rs2694736	23	3128251	G	41	17	A	0.0005984
rs2694735	23	3128925	G	41	17	A	0.0005984
rs1194562	23	3142180	A	40	15	G	0.0002535

rs1194565	23	3143398	G	35	12	C	0.0004754
rs7473053	23	3457060	T	28	46	C	0.0009321
rs11152506	23	3458348	A	28	46	G	0.0009321
rs6567560	23	3459334	C	28	46	T	0.0009321
rs7474188	23	3459801	T	28	46	C	0.0009321
rs200267680	23	3473257	C	28	47	T	0.0005255
rs34949495	23	4590736	CA	15	33	C	0.0004934
rs5915772	23	4591677	T	10	27	C	0.0006386
rs5916687	23	4596138	A	10	29	G	0.0001875
rs5916688	23	4596415	T	10	29	C	0.0001875
rs12848207	23	4601110	A	11	29	C	0.0004498
rs7881910	23	4628697	A	57	29	C	0.0002928
rs5916697	23	4628916	T	29	47	C	0.0009467
rs72609517	23	6040845	G	0	13	A	0.00004253
rs200717890	23	6041758	GGAAAA	0	13	G	0.00004253
rs16984312	23	6666678	C	37	14	T	0.0006739
rs16984313	23	6666760	T	37	14	C	0.0006739
rs2521390	23	9456529	G	29	47	A	0.0009467
rs2681635	23	9462851	A	29	47	G	0.0009467
rs62918660	23	9463932	C	27	47	CAT	0.0002837
rs17321022	23	9594445	G	18	3	C	0.0009486
rs201682170	23	9599039	A	20	3	AAT	0.000269
rs5934663	23	9601245	A	20	3	T	0.000269
rs2040616	23	9605377	A	20	3	C	0.000269
rs6419032	23	9606946	C	20	3	T	0.000269
rs2040615	23	9627214	A	18	1	G	0.00004271
rs5933750	23	9643364	A	15	33	G	0.0005689
rs73476680	23	9926620	C	12	0	T	0.0003752
rs144584394	23	9928518	A	13	0	G	0.0001783
rs73192426	23	12427935	A	43	16	C	0.00009051
rs16986854	23	12431238	A	40	12	G	0.00002131
rs72614507	23	13587829	G	19	3	T	0.0005082
rs150007863	23	19089203	G	1	13	A	0.0003716
rs7066463	23	21918357	G	37	13	T	0.0005994
rs5951526	23	22284928	T	1	12	C	0.0007887
rs5904523	23	22285006	T	35	13	A	0.000996
rs1911645	23	22498476	A	5	19	G	0.0008415
rs7059501	23	22518710	G	5	19	T	0.0008415
X:22554130	23	22554130	A	5	23	ATTTGACATACCG	0.00005584
rs2040774	23	22821307	A	23	42	G	0.0004519
rs5926337	23	23465760	C	17	1	T	0.00008737

rs5986537	23	25964013 G	8	27 A	0.00009961
rs5986539	23	25968617 G	8	27 C	0.00009961
rs5986294	23	25970209 T	8	27 C	0.00009961
rs5986590	23	26223982 T	13	30 C	0.0007578
rs12394146	23	26225684 T	14	31 C	0.0008795
rs1476724	23	26233177 C	10	29 T	0.0001888
rs977338	23	26266547 T	19	37 C	0.0009069
rs5943880	23	26267490 C	19	37 T	0.0009069
rs35042932	23	26269948 C	30	49 CT	0.0008996
rs4475663	23	26271482 G	13	30 A	0.0007578
rs5944389	23	26273505 A	30	49 G	0.0008996
rs721987	23	26273713 C	13	30 T	0.0007578
rs5943882	23	26274682 A	16	35 T	0.0005888
rs34472916	23	26277903 GT	19	37 G	0.0009069
rs59852885	23	26278248 GT	19	37 G	0.0009069
rs5944534	23	26606227 G	25	43 A	0.0008649
rs10482291	23	26608123 G	25	43 C	0.0008649
rs7054599	23	26609360 A	26	44 G	0.0008911
rs35413652	23	26612217 TC	26	44 T	0.0008911
rs5944535	23	26612639 C	26	44 G	0.0008911
rs5944537	23	26613253 A	26	44 C	0.0008911
rs144349945	23	26613381 CT	26	44 C	0.0008911
rs76835086	23	26613383 A	26	44 C	0.0008911
rs5944539	23	26613671 A	26	44 G	0.0008911
rs2215098	23	26618573 A	26	44 G	0.0008911
rs5901821	23	26620088 GA	26	44 G	0.0008911
X:26620893	23	26620893 TC	18	37 T	0.0008489
rs9887413	23	26621343 G	26	44 A	0.0008911
rs5943945	23	26621949 A	26	44 G	0.0008911
rs5944545	23	26622118 A	26	44 T	0.0008911
rs988431	23	26623255 T	26	44 A	0.0008911
rs5944546	23	26624317 A	26	44 T	0.0008911
rs5944547	23	26624744 G	26	44 A	0.0008911
rs989000	23	26625309 T	26	44 A	0.0008911
rs988999	23	26625337 C	26	44 T	0.0008911
rs988998	23	26625516 G	26	44 A	0.0008911
rs5986627	23	26625579 C	26	44 A	0.0008911
rs78502736	23	26625869 T	26	44 TA	0.0008911
rs35744125	23	26625946 C	26	44 CA	0.0008911
rs5986333	23	26626529 A	26	44 C	0.0008911
rs758175	23	26631265 T	26	44 C	0.0008911

rs5986629	23	26633883	A	26	44	G	0.0008911
rs5986631	23	26635139	T	26	44	C	0.0008911
rs5944548	23	26636386	G	24	44	A	0.0004371
rs60978572	23	26638392	A	26	44	G	0.0008911
rs1921978	23	26641227	T	26	44	C	0.0008911
rs5943947	23	26642574	T	26	44	G	0.0008911
rs4898185	23	26650298	G	47	22	A	0.0008379
rs5944555	23	26653460	G	47	22	T	0.0008379
rs5943950	23	26665926	C	47	22	T	0.0008379
rs5944558	23	26666834	T	47	22	G	0.0008379
rs5986336	23	26669266	A	47	22	T	0.0008379
rs4898186	23	26683055	T	47	22	A	0.0008379
rs12844113	23	26685219	C	17	39	T	0.0002254
rs7880631	23	26685231	C	48	22	G	0.0002216
rs5944563	23	26688068	C	47	22	G	0.0008379
rs7472212	23	29734467	C	24	43	G	0.000471
rs76973499	23	31873313	GTT	43	19	G	0.000761
rs5928085	23	32851060	A	25	43	G	0.0008649
rs5928229	23	33314650	A	5	19	G	0.0008415
rs9887051	23	33393169	C	23	3	T	0.00006268
rs150218268	23	33394045	T	23	3	C	0.00006268
rs62594431	23	33394466	A	23	3	T	0.00006268
rs28731408	23	36990246	G	24	4	T	0.00005999
rs77729295	23	40070772	C	26	45	T	0.0005028
rs12395423	23	40070894	C	26	45	T	0.0005028
rs12395451	23	40070960	C	26	45	T	0.0005028
rs4240043	23	40072652	C	27	45	T	0.0009136
rs5963763	23	40076745	G	26	45	A	0.0005028
rs5963167	23	40076763	T	27	45	C	0.0009136
rs10126799	23	40076904	G	27	45	A	0.0009136
rs201133418	23	40077858	C	19	39	CT	0.0005281
rs6520627	23	40078795	G	30	47	A	0.0008836
rs56284094	23	40079406	C	31	49	T	0.0009634
rs6651731	23	40080990	T	30	50	C	0.0002951
rs5963764	23	40081369	A	31	50	T	0.0005409
rs9785476	23	40086428	G	29	48	A	0.0005332
rs61519777	23	40086585	T	30	48	C	0.0009571
rs60570570	23	40087561	C	30	48	T	0.0009571
rs17148391	23	40088000	C	30	48	T	0.0009571
X:41868970	23	41868970	A	0	10	G	0.0004292
rs190932725	23	41889467	A	0	9	G	0.0009641

rs200527041	23	42065819	C	50	23	T	0.0004802
rs5918304	23	42077458	G	40	16	A	0.0005221
rs6610672	23	42084024	G	40	16	A	0.0005221
rs5905679	23	43957659	T	26	45	C	0.0008375
rs5905680	23	43957749	C	29	49	A	0.0002928
rs5905684	23	43959356	T	26	45	C	0.0005028
rs5953230	23	43959901	C	27	49	T	0.00007972
rs4532735	23	43960365	A	26	47	G	0.0001488
rs5905687	23	43963452	T	27	46	C	0.0005154
rs5905688	23	43964530	T	27	46	C	0.0005154
rs17300214	23	44430573	C	15	1	T	0.0003561
rs143649408	23	45585221	C	26	45	CA	0.0002571
rs56976399	23	45634577	A	13	34	C	0.00006374
rs5906069	23	45638441	G	13	30	A	0.0007578
rs5906073	23	45642604	C	19	37	T	0.0009069
rs12846518	23	46934714	T	16	34	C	0.0006492
rs6520361	23	48413952	T	8	25	C	0.0003633
rs235833	23	48423977	C	4	20	G	0.0001364
rs112733390	23	53388342	CGAAA	45	19	C	0.0003833
rs7058180	23	53392821	T	45	19	G	0.0003833
rs5978131	23	53420510	T	46	21	C	0.0007859
rs1264007	23	53433377	C	45	19	A	0.0003833
rs1264009	23	53446146	A	46	19	G	0.0002121
rs1264011	23	53449568	G	45	19	A	0.0003833
rs1264012	23	53454380	T	45	19	C	0.0003833
rs1264013	23	53457622	T	45	19	C	0.0003833
rs1264014	23	53461359	G	45	19	C	0.0004142
rs1264015	23	53464789	C	47	21	T	0.0004473
rs1268420	23	53465014	A	45	19	G	0.0003833
rs1264016	23	53467212	G	45	19	A	0.0003833
rs1264017	23	53468729	T	45	19	G	0.0003833
rs1264018	23	53471786	A	47	17	G	0.00001863
rs56902142	23	53488796	G	49	20	C	0.00006674
rs57500243	23	53488797	G	49	20	A	0.00006674
rs76672031	23	53488798	T	49	20	TCTCAC	0.00006674
rs57928918	23	53488804	T	49	20	C	0.00006674
rs6638394	23	53507175	A	48	18	G	0.00005283
rs6638395	23	53507192	C	48	18	T	0.00002445
rs6529681	23	53520450	G	48	17	A	0.00001451
rs35423729	23	53521162	C	51	20	CG	0.000003551
rs414960	23	53521570	T	46	20	G	0.0004155

rs6529683	23	53542091	G	52	23	C	0.00008358
rs6529684	23	53542107	A	52	22	G	0.00004088
rs266785	23	53550396	A	52	23	G	0.00008358
rs138150690	23	53550777	T	52	22	TA	0.00004088
rs6638399	23	53553599	C	51	23	G	0.000156
rs12687615	23	53554490	T	52	22	C	0.00004088
rs12688962	23	53554556	C	52	22	G	0.00007255
rs3747437	23	53562653	G	52	23	C	0.00008358
rs7061946	23	53567451	G	52	24	A	0.00008225
rs5978135	23	53576978	G	53	24	C	0.00008869
rs5978136	23	53599113	A	52	24	G	0.0001653
rs35473057	23	53601990	A	52	24	C	0.0001653
rs200129868	23	53606383	G	52	25	A	0.0005313
rs1244412	23	53606926	T	52	23	A	0.0001455
rs266786	23	53607754	C	52	23	A	0.00008358
rs201921493	23	53616093	C	53	23	CCACTT	0.00004378
rs266783	23	53621006	C	46	23	G	0.0004343
rs1244413	23	53622078	A	52	23	T	0.00008358
rs12850534	23	53629204	T	53	23	C	0.00003705
rs6638408	23	53633493	C	52	23	T	0.00008358
rs35007111	23	53635340	CAT	53	23	C	0.00002053
rs12838744	23	53641013	G	52	23	A	0.00008358
rs6638360	23	53641647	T	53	23	C	0.00004378
rs12861474	23	53643671	A	53	23	G	0.00004378
rs414923	23	53646789	A	52	23	G	0.00008358
rs155441	23	53648864	A	53	23	G	0.00004378
rs1858002	23	53649492	G	52	23	A	0.00008358
rs6638409	23	53659047	C	52	23	T	0.00008358
rs6638410	23	53662826	T	53	23	C	0.00004378
rs6638361	23	53667634	C	53	24	T	0.00008869
rs198508	23	53667875	G	52	23	A	0.00008358
rs12851158	23	53670842	C	52	23	A	0.00008358
rs3788947	23	53675478	A	51	23	C	0.00007776
rs3788948	23	53675488	A	49	24	C	0.0002665
rs6638411	23	53682038	G	51	23	A	0.000156
rs2036658	23	53682332	C	50	23	G	0.0002847
rs6638413	23	53686534	G	52	24	A	0.0001653
rs10533508	23	53687844	CTCAAG	53	23	C	0.00004378
rs6638414	23	53689992	G	52	23	A	0.00008358
rs6638417	23	53709964	A	53	23	G	0.00004378
rs6638364	23	53717223	T	51	25	C	0.0002971

rs76983280	23	53772116	TGTAAGATATAAG	53	27	T	0.0006109
rs199990477	23	53772387	G	53	23	GT	0.0001357
rs12383852	23	53772556	C	52	25	T	0.0005313
rs5936593	23	70035880	A	48	23	G	0.0008881
rs5936994	23	70036041	C	48	23	T	0.0008881
rs5936997	23	70039038	A	48	23	G	0.0008881
rs6624505	23	70043864	T	48	23	C	0.0008881
rs6624506	23	70044280	T	48	23	C	0.0008881
rs5936999	23	70044965	A	48	23	C	0.0008881
rs150018032	23	70094743	A	18	2	G	0.0002408
rs201334832	23	70105023	TG	17	2	T	0.000467
rs151194156	23	70122562	A	36	12	G	0.0002646
rs6653183	23	71316493	T	21	3	A	0.0002475
rs6625945	23	71323100	A	21	3	G	0.0002475
rs111997647	23	71325825	C	21	3	T	0.0002475
rs6525588	23	71394102	G	20	2	C	0.00006204
rs55703267	23	71397737	A	20	2	G	0.00006204
rs72630041	23	71399338	G	16	2	A	0.0008037
rs59979642	23	71419655	A	18	2	ATGGACCTC	0.0004898
rs56114250	23	71534308	T	11	0	C	0.0007855
rs7886860	23	78029689	T	18	3	C	0.0009486
rs146390190	23	78032346	G	35	13	GT	0.000996
rs67029763	23	78045223	C	30	10	A	0.0009218
rs5922956	23	83549398	G	0	9	T	0.000946
rs58576073	23	83679457	T	3	17	TA	0.0005279
rs5922048	23	83689867	C	3	19	T	0.0001254
rs201647610	23	83849660	CAT	0	12	C	0.00009666
X:86268314	23	86268314	C	12	31	CTGGTGCATTTAC	0.0006149
rs139097899	23	87238699	G	5	20	A	0.0004383
rs138523332	23	87290113	A	4	20	G	0.0001364
rs6522031	23	87598676	A	1	13	G	0.0003716
rs6617621	23	87650084	C	1	16	T	0.00003625
rs73238213	23	88290481	G	0	11	A	0.0001895
rs73244246	23	88450939	C	0	11	T	0.0001895
rs4243361	23	90192817	G	26	44	A	0.0007829
rs2988819	23	90200597	G	26	44	C	0.0007829
rs138123288	23	90222271	C	23	44	G	0.0003924
rs114041692	23	90245638	A	25	44	T	0.00081
rs112326780	23	90253572	T	24	44	C	0.0007371
rs78423501	23	90406523	A	13	31	G	0.0007873
rs201479842	23	90406997	T	12	31	G	0.0006149

rs4020573	23	91818820	G	26	44	A	0.0008911
rs4020577	23	91818950	A	26	44	G	0.0008911
rs144461863	23	91819375	G	26	44	C	0.0008911
rs2750542	23	91821681	A	26	44	T	0.0008911
rs202129962	23	91827711	C	26	45	T	0.0005028
rs144283232	23	91834197	C	26	45	T	0.0005028
rs3952482	23	91835233	C	24	41	T	0.0007073
rs2506262	23	92119324	T	14	32	G	0.0004925
rs34542839	23	93172070	AT	48	22	A	0.0004783
rs62595208	23	93178452	T	36	12	C	0.0002646
rs12156716	23	93181586	T	48	22	C	0.0004783
rs62595211	23	93192956	T	48	22	C	0.0004783
rs11796448	23	93305179	G	9	26	A	0.0004648
rs11091940	23	93311355	G	11	28	A	0.0008103
rs139562096	23	101097636	C	18	3	G	0.0009486
rs7058718	23	102914790	A	49	24	G	0.000936
rs210434	23	104949928	G	2	19	A	0.00002464
rs5962574	23	104956461	T	1	16	G	0.00003625
rs210439	23	104959059	G	2	19	A	0.00002464
rs79773397	23	104961028	G	2	18	A	0.00005262
rs3038940	23	104963739	TAA	2	15	T	0.0004445
rs73636261	23	104964027	C	2	19	T	0.00002464
rs113785568	23	104965409	CA	2	16	C	0.0001031
rs11449372	23	104971032	AT	2	17	A	0.0001038
rs7891511	23	104973602	G	2	19	T	0.00002464
rs11092543	23	104975253	T	1	16	C	0.00003625
rs5962575	23	104981504	C	2	19	T	0.00002464
rs5962297	23	104983013	T	1	16	C	0.00003625
rs200458290	23	104983337	GATTT	2	19	G	0.00002464
rs112243672	23	104984012	C	1	16	T	0.00003625
rs4347108	23	104984105	T	1	16	G	0.00003625
rs60192032	23	104984989	T	1	16	C	0.00003625
rs5962577	23	104985164	C	1	16	T	0.00003625
rs5962578	23	104985376	A	1	16	G	0.00003625
rs10126447	23	104988706	G	2	19	A	0.00002464
rs10218388	23	104988949	C	2	19	T	0.00002464
rs3764765	23	104992963	C	2	19	T	0.00002464
rs5962298	23	104993513	G	2	19	A	0.00002464
rs5962579	23	104994182	T	2	19	A	0.00002464
rs5903270	23	104994857	GA	3	16	G	0.0005264
rs9887672	23	104996155	T	1	16	C	0.00003625

rs59823277	23	105002403	A	1	16	ATC	0.00003625
rs201435006	23	105050984	GT	1	14	G	0.0001538
rs103026	23	105088008	C	1	15	G	0.00007717
rs77665272	23	105122322	A	1	14	G	0.000173
rs5962597	23	105151664	T	1	14	C	0.000173
rs5962608	23	105236119	T	9	25	G	0.0009353
rs78553510	23	105268812	C	1	14	CT	0.000173
rs150724424	23	109752418	A	11	0	G	0.0007855
rs141911700	23	109770433	G	12	0	A	0.0003752
X:109846096	23	109846096	T	12	0	C	0.0003752
rs149294490	23	109887848	G	12	0	A	0.0003752
rs141590782	23	110212107	A	11	0	G	0.0007855
rs16986963	23	111647953	A	11	0	G	0.0007855
rs137987863	23	113397120	C	0	12	T	0.00008299
rs143901299	23	113452552	T	0	10	C	0.0004292
rs5946279	23	114337482	A	25	43	C	0.0008649
rs150903498	23	114569770	T	26	4	C	0.00002957
rs7884307	23	114574640	A	27	4	C	0.00001464
rs73636279	23	114576213	C	27	4	A	0.00001464
rs78902560	23	114578966	C	29	4	A	0.000003453
rs73636280	23	114579062	T	29	4	C	0.000003453
rs201116393	23	114579152	C	28	4	CCACACGGTA	0.000006572
rs148225485	23	114579323	A	28	4	C	0.000007156
rs73580006	23	114579496	T	29	4	C	0.000003453
rs7891269	23	114580249	C	29	4	T	0.000003453
rs7886209	23	114580270	A	29	4	G	0.000003453
rs7891410	23	114580274	C	29	4	T	0.000003453
rs1884396	23	114580445	C	28	4	T	0.000007156
rs375467466	23	114582884	T	27	4	G	0.000007583
rs73580011	23	114583333	A	29	4	G	0.000003453
rs73580014	23	114583399	C	28	4	A	0.000007156
rs73580016	23	114583776	G	28	4	A	0.000007156
rs73580018	23	114584158	C	29	4	T	0.000003453
rs147457731	23	114584186	C	29	4	T	0.000003453
rs73580019	23	114584656	C	29	4	T	0.000003453
rs10521696	23	114584805	G	29	4	A	0.000003453
rs73580022	23	114587083	A	29	4	T	0.000003453
rs73580023	23	114587527	T	28	4	C	0.000007156
rs5987877	23	114587714	C	28	4	T	0.000007156
rs5987878	23	114587792	C	28	4	T	0.000008243
rs17095493	23	114588167	T	29	4	A	0.000003453

rs112945252	23	114588241	A	28	4	G	0.000007156
rs4911795	23	114589262	G	29	4	T	0.000003453
rs59969921	23	114589858	G	29	4	A	0.000003453
rs4911814	23	114590155	C	28	4	T	0.000007156
rs4911816	23	114590336	A	29	4	G	0.000003453
rs1321125	23	114591500	G	28	4	C	0.000007156
rs2231	23	114591894	C	29	4	T	0.000004064
rs5987726	23	114591955	C	29	4	T	0.000003453
rs73580032	23	114592176	A	28	4	G	0.000007156
rs73580034	23	114592197	A	28	4	G	0.000007156
rs1853000	23	114593037	G	28	4	A	0.000007156
rs1853001	23	114593073	C	29	4	T	0.000003453
rs59121764	23	114594049	C	29	4	T	0.000003453
rs35036780	23	114594708	G	29	4	A	0.000003453
rs113910546	23	114595258	A	29	4	G	0.000003453
rs112652023	23	114596923	A	29	4	G	0.000003453
rs725102	23	114601290	G	29	4	A	0.000003453
rs112071098	23	114604239	A	29	4	C	0.000003453
rs7892307	23	114605041	G	29	4	T	0.000003453
rs1016237	23	114608863	T	29	4	C	0.000003453
rs12014903	23	114611273	A	29	4	C	0.000003453
rs139564840	23	114611882	C	29	4	T	0.000003453
rs73580071	23	114612927	T	29	4	C	0.000003453
rs141250214	23	114616333	C	29	4	G	0.000003453
rs73580080	23	114620385	A	29	6	G	0.00004398
rs73580081	23	114620414	A	29	6	G	0.00004398
rs73580092	23	114625847	T	29	6	C	0.00004398
rs2006034	23	114628701	T	28	6	G	0.00008463
rs112265590	23	114631322	C	28	6	T	0.00008463
rs7878205	23	114633517	A	28	6	G	0.00008463
rs17260670	23	114634535	C	28	6	T	0.00008463
rs28380553	23	114634849	T	29	6	G	0.00004398
rs7892164	23	114635033	C	0	9	T	0.0009641
rs142179773	23	114636247	TTTCACATTTCA	28	6	T	0.00008463
rs147550727	23	114637088	C	28	6	T	0.00008463
rs111670357	23	114637227	C	29	6	T	0.00004398
rs17260677	23	114637278	A	29	6	G	0.00004398
X:114638348	23	114638348	T	18	2	C	0.0005323
X:114638352	23	114638352	T	18	2	C	0.0004916
X:114638353	23	114638353	G	18	2	A	0.0005323
X:114638356	23	114638356	C	18	2	CCT	0.0004797

X:114638361	23	114638361	G	18	2	A	0.0002374
rs113966797	23	114640655	C	29	6	T	0.00004398
rs4911820	23	114641418	T	30	6	C	0.00002254
rs140286738	23	114644640	A	0	9	T	0.0009641
rs17326548	23	114644730	C	29	6	G	0.00004398
rs4911821	23	114645027	T	30	6	C	0.00002254
rs17326555	23	114646245	G	39	14	C	0.0002137
rs73571764	23	114648195	C	35	8	A	0.000007387
rs7066718	23	114649607	C	35	8	T	0.000007387
rs73571769	23	114650999	T	39	14	C	0.0002137
rs17326562	23	114652834	C	39	14	A	0.0002137
rs73571773	23	114654595	G	31	8	A	0.0000853
rs199853590	23	114654636	AT	30	8	A	0.00008574
rs7062146	23	114656318	G	35	8	A	0.000007387
rs7060823	23	114656498	A	35	8	G	0.000007387
rs17326576	23	114657155	C	34	8	T	0.0000145
rs5987730	23	114657188	A	47	22	G	0.0008379
rs2207050	23	114657765	G	35	8	C	0.000007387
X:114658165	23	114658165	CTT	30	8	C	0.0001532
rs7888906	23	114658545	T	35	6	C	0.000001303
rs112844284	23	114659292	A	34	8	T	0.0000145
rs73571785	23	114659368	A	34	8	G	0.0000145
rs60895005	23	114659427	A	35	8	G	0.000007387
rs926705	23	114659687	G	35	8	A	0.000007387
rs926707	23	114659994	A	35	8	G	0.000007387
rs73571789	23	114660377	G	35	6	A	0.000001303
rs113729077	23	114662377	A	35	8	T	0.000007387
rs73571793	23	114663073	G	34	8	A	0.0000145
rs17326583	23	114663116	T	34	8	C	0.0000145
rs7883426	23	114663960	G	34	8	A	0.0000145
rs7887546	23	114664241	C	34	8	T	0.0000145
rs73571802	23	114665460	C	34	8	A	0.0000145
rs17260719	23	114665861	A	35	8	G	0.000007387
rs73573605	23	114665905	A	34	8	G	0.0000145
rs17260733	23	114667356	C	34	8	T	0.0000145
rs7881695	23	114667505	G	34	8	A	0.0000145
rs113970892	23	114669417	A	34	8	T	0.0000145
rs111336133	23	114669445	A	34	8	G	0.0000145
rs111499908	23	114669468	T	35	8	C	0.000007387
rs150111707	23	114669633	TACA	34	8	T	0.0000145
rs12353835	23	114670923	G	35	8	A	0.000007387

rs5987899	23	114671863	G	34	8	C	0.0000145
rs73573616	23	114672632	C	35	8	G	0.000007387
rs2143820	23	114673248	A	35	8	G	0.000007387
rs73573621	23	114676324	C	35	8	T	0.000007387
rs6655312	23	114679180	C	35	8	T	0.000007387
rs6655314	23	114679870	G	35	8	C	0.000007387
rs17326632	23	114680418	A	33	8	G	0.00002805
rs111228309	23	114681030	C	32	8	T	0.00005347
rs4911824	23	114681792	T	33	8	G	0.00002805
rs6655289	23	114685636	G	33	8	T	0.00002805
rs112042949	23	114686065	A	33	8	G	0.00002805
rs149609113	23	114686458	TAG	32	8	T	0.00005347
rs113431943	23	114687392	C	32	8	T	0.00005347
rs17260747	23	114688227	A	33	8	G	0.00002805
rs6655291	23	114689103	C	33	8	T	0.00002805
rs6655316	23	114689468	A	32	10	C	0.0003432
rs17260768	23	114691582	T	34	9	C	0.00007119
rs17326639	23	114692806	C	34	9	G	0.00004073
rs112847071	23	114692966	T	34	9	A	0.00004073
rs17326646	23	114693883	C	34	9	A	0.00004073
rs7881439	23	114695348	G	34	11	C	0.0003893
rs17326660	23	114695671	T	34	9	C	0.00004073
rs2398577	23	114697220	C	33	9	G	0.00007642
rs5987903	23	114698458	G	51	23	A	0.0002672
rs111334138	23	114700313	G	47	21	C	0.0004002
rs17260796	23	114700937	C	34	9	T	0.00004073
rs17260803	23	114702183	G	34	9	A	0.00004073
rs112659073	23	114702395	C	34	9	T	0.00004073
rs112990400	23	114702495	A	51	25	G	0.0005636
rs7064642	23	114702517	G	51	25	A	0.0005636
rs2398578	23	114704901	T	33	9	C	0.00007642
rs1883921	23	114706218	C	49	23	G	0.0005083
rs2398579	23	114706602	C	51	25	A	0.0005636
rs5987736	23	114709751	C	52	26	T	0.0005884
rs5987907	23	114709810	C	52	26	T	0.0005884
rs9887298	23	114710194	C	52	26	T	0.0005884
rs112561107	23	114710567	A	35	9	G	0.00003581
rs5987737	23	114710721	C	52	26	T	0.0005884
rs7050301	23	114712096	A	35	9	G	0.00003581
rs111255211	23	114712752	T	35	9	G	0.00003745
rs5987913	23	114713957	T	39	11	A	0.00001648

rs5987914	23	114713960	A	39	11	G	0.00001648
rs28613559	23	114716113	A	35	9	C	0.00003581
rs5987916	23	114717392	G	35	9	A	0.00003581
rs5987917	23	114717439	A	35	9	G	0.00003581
rs2223825	23	114718982	G	39	11	A	0.00001648
rs113944167	23	114719389	A	34	7	G	0.000008931
rs5987919	23	114720046	C	39	11	T	0.00001648
rs7878501	23	114720306	A	39	11	T	0.00001648
rs12391829	23	114720420	T	35	9	C	0.00003581
rs111949579	23	114721673	A	35	9	G	0.00003581
rs2207046	23	114721793	G	39	11	A	0.00001648
rs371553866	23	114721822	C	38	9	CA	0.000004482
rs2207047	23	114721835	T	38	9	C	0.000004482
rs2207048	23	114722177	T	38	9	C	0.000004482
rs59067332	23	114723192	C	39	9	T	0.000002168
rs73577794	23	114724262	T	37	9	G	0.000004114
rs113480467	23	114724357	G	33	9	A	0.00006226
rs73577800	23	114725164	A	39	9	G	0.000002168
rs2398580	23	114725828	G	39	11	A	0.00001648
rs2895154	23	114725960	G	38	9	T	0.000004482
rs9887208	23	114726150	T	32	7	C	0.00001751
rs6655319	23	114726212	T	39	11	A	0.00001648
rs139261717	23	114727944	C	35	9	CA	0.00003581
rs5987742	23	114728259	C	39	11	T	0.00001648
rs113110371	23	114729165	G	35	9	A	0.00003581
rs12393386	23	114729654	G	32	9	T	0.0001178
rs4911827	23	114731229	G	34	12	T	0.000839
rs61525850	23	114731984	A	34	11	C	0.0003893
rs9724449	23	115020940	C	40	15	T	0.0002535
rs75819721	23	115643799	C	1	17	CT	0.00001859
rs78300430	23	115648351	C	2	19	G	0.00002464
rs149087657	23	115648862	A	2	17	G	0.0001162
rs5905207	23	115653462	T	1	17	C	0.00001632
rs6608524	23	115654928	A	1	17	G	0.00001632
rs988456	23	115659164	A	52	24	G	0.0001653
rs139721366	23	115661289	T	52	24	G	0.0001653
rs1013220	23	115662445	T	52	24	C	0.0001653
rs5905338	23	115663534	T	52	24	C	0.0001653
rs5905342	23	115664640	G	52	24	A	0.0001653
rs6651577	23	115665691	G	52	24	A	0.0001653
rs5905343	23	115666144	C	52	24	T	0.0001653

rs5905211	23	115666246	T	52	24	C	0.0001653
rs5905346	23	115667761	T	52	24	C	0.0001653
rs6608698	23	115668322	C	52	24	T	0.0001653
rs6608699	23	115669288	G	52	24	A	0.0001653
rs4145420	23	115670754	G	52	24	A	0.0001653
rs6608527	23	115670779	C	52	24	G	0.0001653
rs4145421	23	115670837	A	42	18	G	0.0006782
rs5905347	23	115671758	T	52	21	C	0.00003499
X:115672688	23	115672688	T	52	24	TTC	0.0001653
X:115672692	23	115672692	CAAATTAGAG	52	24	C	0.0001653
X:115672695	23	115672695	TGACAAATTGACA	52	24	T	0.0001653
rs2187881	23	115673376	T	52	24	A	0.0001653
rs2157427	23	115673454	G	51	22	A	0.0001366
rs5905348	23	115675146	T	52	24	C	0.0001653
rs11260159	23	115734350	T	52	24	C	0.0001653
rs9723899	23	115740720	C	23	44	G	0.0001337
rs9724300	23	115740847	G	23	44	C	0.00006607
rs9329406	23	115740985	A	26	48	G	0.000078
rs9780253	23	115741598	C	52	22	A	0.00004088
rs12390117	23	115743813	G	52	22	A	0.00004088
rs11260106	23	115744144	C	52	22	T	0.00004088
rs11260118	23	115744897	C	52	22	T	0.00004088
rs11260121	23	115745642	C	52	22	T	0.00004088
rs6655546	23	115747311	T	52	22	C	0.00004088
rs11260135	23	115747885	G	52	22	A	0.00004088
rs112534898	23	115747924	G	52	22	GAT	0.00004088
rs12834189	23	115748233	A	52	22	G	0.00004088
rs12837027	23	115748364	C	52	22	T	0.00004088
rs200565252	23	115749377	G	52	22	GA	0.00004088
rs11260148	23	115749542	C	52	22	A	0.00004088
rs11260149	23	115749593	A	52	22	C	0.00004088
rs11260150	23	115749818	G	52	22	A	0.00004088
rs11260151	23	115749920	A	52	22	G	0.00004088
rs12398681	23	115750080	C	52	22	T	0.00004088
rs12398711	23	115750294	G	52	22	T	0.00004088
rs12400117	23	115750388	T	42	18	C	0.0006782
rs28441538	23	115750497	A	31	52	G	0.00009769
rs66834529	23	115751442	C	52	22	T	0.00004088
rs73226865	23	115751478	A	52	22	T	0.00004088
rs11260156	23	115752193	A	52	22	T	0.00004088
rs12389361	23	115752510	A	23	44	T	0.0001337

rs12398615	23	115752926 T	52	22 C	0.00004088
rs10907033	23	115753053 G	52	22 A	0.00004088
rs10907034	23	115753283 G	52	22 C	0.00004088
rs10907035	23	115753330 A	52	22 G	0.00004088
rs10796347	23	115753621 C	52	22 T	0.00004088
rs11260172	23	115755324 C	54	23 T	0.00004033
rs12400026	23	115755656 A	31	52 T	0.00009769
rs12557454	23	115756177 T	31	52 C	0.00009769
rs74574712	23	115756248 TA	31	52 T	0.00009769
rs73213286	23	115756372 C	31	52 A	0.00009769
rs12398361	23	115757430 G	20	39 A	0.0005694
rs6603394	23	115757476 G	44	17 A	0.000106
rs12400045	23	115758626 A	20	39 G	0.0005694
rs7059336	23	115762843 T	52	23 C	0.00008358
rs7049926	23	115762854 A	52	23 G	0.00008358
rs12835373	23	115764107 C	20	39 T	0.0005694
rs12853107	23	115764937 C	54	23 T	0.00002242
rs7473283	23	115766722 A	13	30 T	0.0007578
rs10658209	23	115769642 G	52	23 GAT	0.00008358
rs7473627	23	115774190 T	12	30 C	0.0005652
rs7391967	23	115774556 A	12	30 G	0.0005652
rs7391753	23	115774601 T	54	23 C	0.00002242
rs12398148	23	115778720 A	12	30 C	0.0005652
rs12847918	23	115780825 A	12	30 G	0.0005652
rs12853855	23	115780844 T	12	30 C	0.0005652
rs12559419	23	115781639 C	24	46 T	0.0000732
rs12395694	23	115781926 C	24	47 T	0.00003747
rs199692781	23	115782112 T	26	46 TAATA	0.000277
rs62605741	23	115782964 C	25	46 T	0.0001445
rs12398468	23	115784630 T	24	46 C	0.0000732
rs11260345	23	115786229 G	25	47 T	0.00007582
rs12395202	23	115786761 T	52	23 C	0.00008358
rs5910167	23	115787601 T	25	46 G	0.0001445
rs5910166	23	115787761 A	24	46 G	0.0000732
rs5912057	23	115788011 G	24	46 C	0.0000732
rs34307386	23	115788942 A	24	47 G	0.00003747
rs5912030	23	115792853 A	54	23 G	0.00002242
rs5912025	23	115794587 A	44	17 G	0.000106
rs7057172	23	115797756 A	56	23 G	0.000005473
rs1506960	23	115798129 T	56	23 C	0.000005473
rs5956740	23	115799907 C	56	23 G	0.000005473

rs369934843	23	115801212	C	12	28	CACAG	0.000945
rs1506958	23	115803356	C	20	38	T	0.0009963
rs5903481	23	115803731	T	46	17	TTATC	0.00004936
rs113858028	23	115806047	A	46	17	ATGT	0.00004936
rs5911731	23	115807323	T	56	23	C	0.000005473
rs1395554	23	115808095	T	20	38	G	0.0009963
rs11798535	23	115808182	C	20	38	G	0.0009963
rs2878305	23	115808704	G	6	21	A	0.0006518
rs1354799	23	115808840	T	20	38	G	0.0009963
rs5911784	23	115809206	C	6	21	T	0.0006518
rs12395259	23	115810023	G	20	38	A	0.0009963
rs7473168	23	115810825	C	20	38	T	0.0009963
rs7473986	23	115811177	C	20	38	T	0.0009963
rs28581957	23	115811962	G	20	38	C	0.0009963
rs2335018	23	115812234	C	20	38	T	0.0009963
rs3965555	23	115812261	C	20	38	A	0.0009963
rs62607578	23	115813719	A	0	10	G	0.0004292
rs145375768	23	115815752	C	0	10	T	0.0004292
rs34339026	23	115816699	C	28	51	CT	0.00001381
rs5909877	23	115825960	G	48	19	A	0.00006091
rs1876296	23	115827591	C	48	19	G	0.00006091
rs141519037	23	116465087	G	3	16	A	0.0009912
rs150999575	23	116746900	A	0	10	C	0.0004292
rs143312856	23	116853186	A	0	10	G	0.0004292
rs2211053	23	116981307	G	13	31	A	0.0004205
rs2211052	23	116981350	C	15	35	G	0.0001708
rs139564216	23	117401280	A	12	0	G	0.0003752
rs438070	23	118625275	T	2	15	C	0.0004445
rs5909632	23	118664198	A	2	15	G	0.0004755
rs66593950	23	118732119	AT	14	33	A	0.0002272
rs802801	23	118734993	G	14	31	A	0.0007479
rs143566226	23	118798931	A	0	9	G	0.0009641
rs5957481	23	119916327	G	27	45	A	0.0009136
rs11314157	23	119917765	T	43	16	TA	0.00009051
rs5909763	23	119918392	G	43	16	A	0.00009051
rs5957482	23	119918735	G	43	16	T	0.00009051
rs5909764	23	119919126	A	43	16	C	0.00009051
rs5911426	23	121954639	T	42	14	G	0.0000334
rs5909997	23	122497307	A	9	25	T	0.0008942
rs34786936	23	122573644	C	3	17	CT	0.0005721
rs62607674	23	122595313	C	39	16	T	0.0009032

rs138621790	23	124622472	A	0	9	C	0.0009641
rs34260006	23	130480304	TAC	8	25	T	0.0003633
rs2073051	23	132473645	A	11	33	G	0.0000209
rs73572505	23	132550175	T	29	7	C	0.0002463
rs7059265	23	132550737	A	29	7	G	0.0001288
rs1546971	23	132554875	G	29	7	T	0.0001288
rs7881013	23	132554944	G	29	7	A	0.0001288
rs1546972	23	132554979	G	29	7	A	0.0001288
rs61405540	23	132557734	A	29	7	G	0.0001288
rs60806152	23	132557796	G	29	8	A	0.0003391
rs7878767	23	132558500	T	26	7	C	0.0003762
rs58213637	23	132558797	A	29	7	G	0.0001288
rs7887463	23	132559557	T	29	7	A	0.0001288
rs73572523	23	132561047	T	29	7	C	0.0001288
rs16993046	23	132561090	T	29	7	A	0.0001288
rs17000449	23	132561910	G	29	7	T	0.0001288
rs58666909	23	132562165	C	29	7	A	0.0001288
rs7876869	23	132562762	C	29	7	A	0.0001288
rs7877025	23	132562894	T	24	6	A	0.0004924
rs67231548	23	133624531	GTA	51	25	G	0.0005636
rs75369405	23	133722328	AT	17	2	A	0.0004125
rs5975654	23	135134722	G	15	1	T	0.0003544
rs2518896	23	135679053	A	20	4	G	0.0007853
rs2518897	23	135679059	A	20	4	G	0.0007853
rs2518904	23	135681823	A	20	2	G	0.0001287
rs141954229	23	138074269	CAT	2	14	C	0.0009649
rs518667	23	138077510	C	0	13	T	0.00003603
rs78974273	23	138079591	A	0	13	AC	0.00003603
rs579260	23	138086137	T	0	13	C	0.00003603
rs471635	23	138086434	A	0	13	C	0.00004253
rs500096	23	138087155	G	0	13	C	0.00003603
rs565127	23	138088701	C	0	13	T	0.00003603
rs472273	23	138097159	G	0	13	C	0.00003603
rs2179589	23	138097310	G	0	13	A	0.00003603
rs566187	23	138098674	A	0	13	T	0.00003603
rs496732	23	138099648	T	0	13	C	0.00002946
rs490787	23	138100179	G	0	13	A	0.00003603
rs512809	23	138101612	C	0	13	A	0.00002946
rs471600	23	138101814	G	0	13	A	0.00003603
rs482328	23	138102649	C	0	13	T	0.00003603
rs483419	23	138105978	C	0	13	T	0.00003603

rs497635	23	138106122	T	0	13	G	0.00003603
rs578264	23	138107101	C	0	13	T	0.00003603
rs515444	23	138107138	G	0	13	A	0.00003603
rs519841	23	138108803	C	0	13	T	0.00003603
rs149173046	23	138110078	C	0	13	CAAGA	0.00003603
rs693310	23	138110176	C	0	13	T	0.00003603
rs5931560	23	138110307	A	0	13	G	0.00003603
rs526093	23	138111126	G	0	13	A	0.00003603
rs477821	23	138111932	A	0	13	G	0.00003603
rs6635762	23	138113107	A	0	13	G	0.00003603
rs67703366	23	138113374	CA	0	13	C	0.00003603
rs79440397	23	138113382	A	0	13	T	0.00003603
rs6634041	23	138113909	C	0	11	A	0.0001604
rs17001950	23	138114091	G	0	13	C	0.00003603
rs60643249	23	138114217	A	0	13	G	0.00003603
rs62602197	23	138114331	A	0	13	G	0.00003603
rs61245857	23	138114383	G	0	13	A	0.00003603
rs6634042	23	138114720	C	0	13	A	0.00003603
rs7884030	23	138115512	C	0	13	T	0.00003603
rs7883424	23	138115569	A	0	13	G	0.00003603
rs17001957	23	138116229	C	0	13	A	0.00003603
rs62602201	23	138116536	A	0	13	G	0.00003603
rs62602202	23	138117015	C	0	13	T	0.00003603
rs16993529	23	138117256	T	0	13	C	0.00003603
rs10521792	23	138117409	G	0	13	A	0.00003603
rs17001960	23	138119216	A	0	13	T	0.00003603
rs62602203	23	138119644	C	0	13	A	0.00003603
rs62602204	23	138119702	A	0	13	T	0.00003603
rs112015472	23	138119748	A	0	13	T	0.00003603
rs7889036	23	138119874	T	0	13	A	0.00003603
rs62602205	23	138120458	A	0	13	G	0.00002946
rs12558011	23	138120702	T	0	13	A	0.00003603
rs12556537	23	138120938	C	0	13	T	0.00003603
rs17001963	23	138122394	T	0	13	G	0.00003603
rs62602208	23	138126601	C	0	13	A	0.00003603
rs62602209	23	138127036	G	0	13	A	0.00003603
rs2103610	23	138127481	A	0	13	G	0.00003603
rs112592286	23	138128310	C	0	13	CCTCAGGAAG	0.00003603
rs62602236	23	138128358	A	0	13	G	0.00003603
rs12560172	23	138130418	C	0	13	T	0.00003603
rs62602238	23	138130921	C	0	13	T	0.00003603

rs62602239	23	138133918	G	0	13	T	0.00003603
rs62602240	23	138134536	G	0	13	A	0.00003603
rs62602241	23	138134795	T	0	13	C	0.00003603
rs1080166	23	138137774	C	0	13	T	0.00003603
rs62602242	23	138140150	C	0	13	T	0.00003603
rs7877464	23	138140408	G	0	13	A	0.00003603
rs12556549	23	138140736	T	0	13	G	0.00003603
rs62602243	23	138142110	A	0	13	G	0.00003603
rs374366794	23	138143770	A	0	13	AT	0.00003603
rs113373639	23	138144111	C	0	13	CTTA	0.00003603
rs143453100	23	138147525	C	0	12	T	0.00008299
rs62602246	23	138148857	A	0	12	G	0.00008299
rs62602247	23	138150491	C	0	10	T	0.0003694
rs138951453	23	138152160	C	0	13	T	0.00003603
rs141403534	23	138152804	A	0	12	G	0.00008299
rs147006243	23	138152877	T	0	12	C	0.00008299
rs112600886	23	138154239	A	0	13	G	0.00003603
rs112886945	23	138155295	T	0	12	TC	0.00008299
rs201924524	23	138155299	G	0	12	C	0.00008299
rs10521793	23	138156505	T	0	12	C	0.00008299
rs17284663	23	138160456	T	0	12	A	0.00008299
rs17331408	23	138160576	C	0	12	T	0.00008299
rs112249167	23	138161557	A	0	12	AC	0.00008299
rs62603179	23	138166155	G	0	13	A	0.00003603
rs62603180	23	138177022	T	0	13	C	0.00003603
rs7890667	23	138178531	A	0	12	G	0.00008299
rs62603183	23	138179958	G	0	12	C	0.00008299
rs111506647	23	138181040	C	0	12	T	0.00008299
rs7884308	23	138182426	A	0	12	G	0.00008299
rs7888266	23	138182784	T	0	12	G	0.00008299
rs62603185	23	138183067	G	0	13	A	0.00004253
rs2206125	23	138185215	T	0	12	C	0.00008299
rs111519164	23	138185531	A	0	12	G	0.00008299
rs17001986	23	138186360	C	0	12	G	0.00008299
rs62603187	23	138186858	T	0	12	C	0.00008299
rs199795206	23	138187174	GT	0	11	G	0.0001604
rs17331422	23	138187934	G	0	13	A	0.00003603
rs979128	23	138188114	C	0	13	A	0.00003603
rs41300307	23	138188496	A	0	12	G	0.00008299
rs201138057	23	138189171	C	0	10	CT	0.0004292
rs200196785	23	138189174	A	0	10	T	0.0004292

rs2227059	23	138236904	C	25	47	T	0.00007582
rs2301396	23	138237094	G	25	44	C	0.000488
rs5908770	23	138650817	G	30	48	T	0.0009571
rs60497947	23	140234395	G	23	3	A	0.00006268
rs57432891	23	140234396	G	23	3	T	0.00006268
rs5908121	23	141160597	T	52	23	A	0.00008358
rs6634356	23	141164624	C	52	23	T	0.00008358
rs4825078	23	141167942	A	45	16	G	0.00002569
rs4825079	23	141168086	G	45	16	A	0.00002569
rs5908132	23	141168290	T	45	18	C	0.0001933
rs2142969	23	141168655	G	50	25	C	0.0009811
rs2179314	23	141168805	G	50	25	A	0.0009811
rs2869073	23	141168838	T	50	24	A	0.0005368
rs2903999	23	141169274	T	50	24	C	0.0005368
rs2869074	23	141169426	G	50	24	A	0.0005368
rs35894125	23	141169501	G	50	24	GA	0.0005368
rs2869075	23	141169550	T	50	24	A	0.0005368
rs5908135	23	141170987	G	50	25	C	0.0009811
rs67628234	23	141171171	A	50	25	G	0.0009811
rs1883397	23	141308325	C	13	30	T	0.0007578
rs62600825	23	141963178	T	29	8	C	0.0003391
rs2223452	23	141964914	A	32	10	G	0.0003432
rs5904083	23	141964932	C	32	10	CT	0.0005752
rs12839812	23	141965807	A	32	10	G	0.0003432
rs139648084	23	141976966	T	29	9	C	0.0008145
rs7885118	23	141978729	A	41	17	T	0.0005984
rs34057690	23	141982184	CTTGA	38	14	C	0.0003829
rs5954986	23	142336450	G	7	23	A	0.0004919
rs5953936	23	143308705	C	26	7	T	0.0007902
rs5953941	23	143325039	T	27	7	C	0.0004381
rs5955281	23	143330493	T	27	7	A	0.0004381
rs12353807	23	143330494	T	27	7	G	0.0004381
rs35584131	23	143333330	TTAAC	27	7	T	0.0004381
rs4268240	23	143333737	G	27	7	A	0.0004381
rs4567183	23	143333901	C	27	7	T	0.0004381
rs56117640	23	143643055	A	1	15	G	0.00007965
rs17309667	23	143644356	T	1	15	G	0.00007965
rs200366744	23	143657153	T	1	14	TAAATGCACAC	0.000173
rs78866384	23	143663615	C	2	15	A	0.0004755
rs56162924	23	143688892	T	1	15	C	0.00007965
rs5920461	23	144294376	C	14	31	T	0.0008795

rs559327	23	144342646	C	17	2	T	0.000467
rs502750	23	144344202	C	17	2	T	0.000467
rs7053970	23	144354539	C	17	2	T	0.000467
rs5920480	23	144359865	T	16	2	C	0.0008956
rs5919820	23	144363288	C	16	2	T	0.0008956
rs2125445	23	144367015	T	16	2	A	0.0008956
rs5965846	23	144369589	C	16	2	A	0.0008956
rs1337635	23	144374307	G	16	2	A	0.0008956
rs5919821	23	144382762	G	16	2	A	0.0008956
rs557943	23	144384458	G	16	2	T	0.0008956
rs599744	23	144385068	C	16	2	A	0.0008956
rs1598987	23	144385268	G	16	2	A	0.0008956
rs5919822	23	144388647	T	17	2	C	0.000467
rs5919823	23	144391681	T	16	2	C	0.0008956
rs2210987	23	144395156	G	16	2	A	0.0008956
rs4454436	23	144398117	C	16	2	G	0.0008956
rs5920488	23	144399577	T	16	2	G	0.0008956
rs75123486	23	144403702	T	16	2	C	0.0008956
rs2152852	23	144406208	C	16	2	T	0.0008956
rs3076258	23	144406771	G	16	2	GTT	0.0008912
rs1974267	23	144406924	C	17	2	T	0.000467
X:144407139	23	144407139	TAC	16	2	T	0.0008956
rs10672257	23	144407215	C	17	2	CTG	0.000467
rs1856207	23	144407841	A	16	2	G	0.0008956
rs1974266	23	144408323	G	16	2	A	0.0008956
rs1007647	23	144408676	A	18	2	T	0.0002092
rs4412509	23	144413331	C	16	2	T	0.0008956
rs1933046	23	144414060	T	17	2	C	0.000467
rs144743683	23	144414309	CTGTT	17	2	C	0.000467
rs1415624	23	144414774	G	16	2	T	0.0008956
rs5919825	23	144415315	G	16	2	T	0.0008956
rs200773179	23	144415343	TTTTTTC	16	2	T	0.0008956
rs5920489	23	144416597	A	16	2	T	0.0008912
rs1538163	23	144417384	A	16	2	G	0.0008956
rs1538162	23	144418490	G	17	2	A	0.000467
rs1415623	23	144418846	C	16	2	T	0.0008956
rs5920490	23	144419479	A	16	2	G	0.0008956
rs375590914	23	144419769	CA	16	2	C	0.0008956
rs5920491	23	144420321	C	17	2	T	0.000467
rs5919826	23	144420383	T	16	2	C	0.0008956
rs5920493	23	144421254	A	16	2	G	0.0008956

rs5919827	23	144421257	C	16	2	T	0.0008956
rs111556485	23	144542821	G	17	2	A	0.000467
rs202237324	23	144591171	C	16	2	CAA	0.0008956
rs4827662	23	144915261	G	49	23	C	0.0005083
rs55876683	23	144961624	G	49	23	A	0.0008445
rs5920019	23	145229419	C	6	21	T	0.0006518
rs111463228	23	145391491	G	1	12	C	0.0007887
rs5920113	23	145550758	T	43	18	A	0.0003905
rs5920114	23	145551278	C	43	19	T	0.000761
rs5920115	23	145551928	G	43	19	T	0.000761
rs112929287	23	145572578	C	41	17	CACACACACACAC	0.0005196
rs12853269	23	145672354	C	29	9	T	0.0008145
rs12855563	23	145672954	G	30	9	T	0.0004612
rs12855872	23	145672989	C	30	9	T	0.0004612
rs7889692	23	147086039	G	11	0	A	0.0007855
rs199528103	23	147091621	AT	11	0	A	0.0007855
rs34993625	23	147767359	A	15	1	G	0.0003561
rs1882727	23	148354706	G	43	19	A	0.000761
rs10709019	23	148354772	TA	43	19	T	0.000761
rs2056833	23	148355171	G	43	19	A	0.000761
rs741733	23	148355251	C	43	19	T	0.000761
rs760051	23	148355553	C	43	19	A	0.000761
rs5936253	23	148358734	C	43	19	T	0.000761
rs5936254	23	148358760	C	43	19	T	0.000761
rs5936255	23	148360525	A	43	19	G	0.000761
rs1007084	23	148361284	A	43	19	G	0.000761
rs373447133	23	148361579	AT	43	19	A	0.000761
rs7051515	23	148362146	T	43	19	C	0.000761
rs5936256	23	148362574	T	43	19	C	0.000761
rs5904262	23	148364087	AG	43	19	A	0.000761
rs4844009	23	148369123	G	43	19	A	0.000761
rs4844010	23	148369188	C	43	19	T	0.000761
rs5936258	23	148369607	C	43	19	T	0.000761
rs4581208	23	148370174	G	43	17	A	0.0003239
rs1882728	23	148370989	A	43	19	C	0.000761
rs6641287	23	148372456	C	45	19	T	0.0003833
rs5980471	23	148553479	A	23	5	G	0.0006807
rs5936284	23	148554165	G	28	5	A	0.00002648
rs5936285	23	148554167	T	28	5	G	0.00002648
rs5936286	23	148555007	A	28	5	G	0.00002648
rs5936186	23	148555199	G	28	5	A	0.00002648

rs1329433	23	148556303	T	28	5	C	0.00002648
rs1329432	23	148556337	A	28	5	G	0.00002648
rs5936287	23	148557940	G	28	5	C	0.00002648
rs5936288	23	148558579	A	28	5	C	0.00002648
rs5936289	23	148558580	G	28	5	T	0.00002648
rs5936290	23	148559165	T	27	5	C	0.00005212
rs5936291	23	148560611	A	26	5	G	0.0001012
rs5936292	23	148560698	A	26	6	G	0.0003002
rs5936188	23	148560766	C	26	5	T	0.0001012
rs11969	23	148561274	C	28	5	T	0.00002648
rs1064463	23	148562599	A	26	5	G	0.0001012
rs685678	23	148565758	C	28	5	A	0.00002648
rs10218066	23	150343248	T	23	4	G	0.0002248
X:151164833	23	151164833	A	3	18	T	0.0002532
rs146310368	23	151705018	G	0	9	A	0.0009641
rs5945320	23	152682605	A	12	0	C	0.0003752
rs6642292	23	1.55E+08	C	32	18	T	0.000005019
rs6642293	23	1.55E+08	C	26	16	T	0.0002858
rs5940512	23	1.55E+08	G	32	18	A	0.000005019
rs5940513	23	1.55E+08	A	26	16	G	0.0002858
rs5940526	23	1.55E+08	T	32	18	A	0.000005019
rs5940397	23	1.55E+08	G	26	16	A	0.0002858
rs12834147	23	1.55E+08	G	26	16	C	0.0002858
rs2890132	23	1.55E+08	G	26	16	A	0.0002858
rs371249305	23	1.55E+08	TA	26	16	T	0.0002858
rs1598125	23	1.55E+08	T	26	16	C	0.0002858
rs139295015	23	1.55E+08	T	32	18	C	0.000005019
rs140600574	23	1.55E+08	C	26	16	T	0.0002858
rs142781423	23	1.55E+08	T	26	16	C	0.0003104
rs1084453	23	1.55E+08	C	26	16	T	0.0002858
rs1084452	23	1.55E+08	C	26	14	T	0.00009952
rs1084451	23	1.55E+08	A	26	16	C	0.0002858
rs1091848	23	1.55E+08	C	26	16	T	0.0003104
rs782311	23	1.55E+08	C	26	16	T	0.0002858
rs782310	23	1.55E+08	T	26	16	C	0.0002858
rs1084450	23	1.55E+08	A	26	16	T	0.0002858
rs782308	23	1.55E+08	T	26	16	A	0.0002858
rs782307	23	1.55E+08	G	32	18	C	0.000005019
rs782306	23	1.55E+08	G	26	16	A	0.0001017
rs809138	23	1.55E+08	T	26	16	C	0.0002858
rs5983745	23	1.55E+08	T	26	16	C	0.0002858

rs5983658	23	1.55E+08	C	26	16	T	0.0002858
rs5940539	23	1.55E+08	A	32	18	C	0.000005019
rs10782079	23	1.55E+08	A	26	16	G	0.0002858
rs522805	23	1.55E+08	T	26	16	C	0.0002858
rs89278	23	1.55E+08	C	26	16	T	0.0002858
rs479901	23	1.55E+08	A	26	16	T	0.0002858
rs476928	23	1.55E+08	C	26	16	G	0.0002858
rs561928	23	1.55E+08	G	26	16	T	0.0002858
rs498000	23	1.55E+08	G	26	16	T	0.0002858
rs580468	23	1.55E+08	A	32	18	G	0.000005019
rs574386	23	1.55E+08	C	26	16	T	0.0002858
rs146884178	23	1.55E+08	T	26	16	TCTTAGAGCC	0.0002858
rs1834433	23	1.55E+08	G	26	16	T	0.00005482
rs672434	23	1.55E+08	T	26	16	A	0.0002858
rs569049	23	1.55E+08	T	26	16	C	0.0002858
rs561841	23	1.55E+08	G	26	16	A	0.0002858
rs75097820	23	1.55E+08	T	26	16	TTA	0.0002858
rs654274	23	1.55E+08	A	26	16	G	0.0002858
rs666267	23	1.55E+08	C	26	16	G	0.0002858
rs666344	23	1.55E+08	A	26	16	C	0.0002858
rs573685	23	1.55E+08	T	26	16	A	0.0002858
rs514136	23	1.55E+08	C	24	16	T	0.0005508
rs513234	23	1.55E+08	T	26	16	C	0.0002858
rs622211	23	1.55E+08	A	26	16	G	0.0002858
rs487307	23	1.55E+08	A	26	16	G	0.0002858
rs601290	23	1.55E+08	G	26	16	A	0.0002858
rs473772	23	1.55E+08	T	26	16	C	0.0002858
rs528286	23	1.55E+08	C	27	16	T	0.0001457
rs612412	23	1.55E+08	G	26	16	A	0.0002858
rs613783	23	1.55E+08	C	32	18	A	0.000005019
rs642362	23	1.55E+08	A	26	16	G	0.0002858
rs372034665	23	1.55E+08	GTACTC	26	16	G	0.0002858
rs150712976	23	1.55E+08	A	26	16	G	0.0002858
rs491661	23	1.55E+08	T	24	16	A	0.0005508
rs494282	23	1.55E+08	C	26	16	T	0.0002858
rs520792	23	1.55E+08	G	32	18	A	0.000005019
rs563690	23	1.55E+08	G	26	16	A	0.0002858
rs608695	23	1.55E+08	A	26	16	G	0.0002858
rs610828	23	1.55E+08	A	26	16	G	0.0002858
rs512918	23	1.55E+08	G	32	18	A	0.000005019
rs636742	23	1.55E+08	C	26	16	T	0.0002858

rs638156	23	1.55E+08	T	26	16	A	0.0002858
rs547437	23	1.55E+08	G	26	16	A	0.0002858
rs671588	23	1.55E+08	C	26	16	T	0.0002858
rs526172	23	1.55E+08	G	26	16	T	0.0002858
rs553678	23	1.55E+08	A	26	16	G	0.0003104
rs513900	23	1.55E+08	C	26	16	T	0.0002858
rs637652	23	1.55E+08	A	26	16	C	0.0002858
rs34440537	23	1.55E+08	TA	26	16	T	0.0002858
rs586843	23	1.55E+08	G	26	16	A	0.0002858
rs549327	23	1.55E+08	C	32	18	A	0.000005019
rs473491	23	1.55E+08	A	32	18	G	0.000005019
rs628761	23	1.55E+08	C	26	16	A	0.0002858
rs627797	23	1.55E+08	C	32	18	G	0.000005019
rs697727	23	1.55E+08	A	26	16	G	0.0002858
rs696316	23	1.55E+08	G	26	16	T	0.0002858
rs697725	23	1.55E+08	A	26	16	T	0.0003104
rs479770	23	1.55E+08	G	26	16	A	0.0002858
rs480725	23	1.55E+08	A	26	16	T	0.0002858
rs674707	23	1.55E+08	G	26	16	A	0.0002858
rs488777	23	1.55E+08	T	26	16	C	0.0002858
rs601837	23	1.55E+08	C	26	16	G	0.0002858
rs473529	23	1.55E+08	G	32	21	C	0.00003887
rs586454	23	1.55E+08	T	25	13	G	0.0000842
rs622581	23	1.55E+08	C	26	16	T	0.0003104
rs35543589	23	1.55E+08	C	26	14	CT	0.00009952
rs571421	23	1.55E+08	A	24	16	G	0.0005508
rs672932	23	1.55E+08	A	26	16	G	0.0002858
rs669237	23	1.55E+08	T	26	16	G	0.0002858
rs642043	23	1.55E+08	T	32	21	C	0.00003887
rs509981	23	1.55E+08	T	26	16	C	0.0002858
rs538470	23	1.55E+08	T	26	16	C	0.0002858
rs645904	23	1.55E+08	T	26	16	C	0.0002858
rs644138	23	1.55E+08	A	32	21	G	0.00003887
rs557132	23	1.55E+08	T	26	16	C	0.0002858
rs4012982	23	1.55E+08	C	25	16	CAA	0.0005585
rs781880	23	1.55E+08	G	22	12	A	0.0002081
X:154981605	23	1.55E+08	A	15	4	T	0.0001557
X:155255914	23	1.55E+08	C	9	1	G	0.0007194

Supplementary Table 4. All MCODE module genes. Indicated by TRUE (T) or FALSE (F) if they were included in the individual modules for neutropenia, leukopenia, and thrombocytopenia, respectively.

Gene Name	EntrezID	Neutropenia	Leukopenia	Thromobocytopenia
AKT3	10000	T	T	F
CDH5	1003	T	F	T
TOM1L1	10040	F	T	F
OCLN	100506658	T	F	F
NR1H3	10062	T	T	F
TRAP1	10131	F	T	F
OPTN	10133	F	T	F
TOB1	10140	F	T	T
SORBS3	10174	F	F	T
TRIB1	10221	F	F	T
STAM2	10254	F	T	F
CDKN1B	1027	T	F	F
CDKN2A	1029	F	F	T
CCNO	10309	T	F	F
ANAPC10	10393	F	F	T
PRMT5	10419	F	T	T
UBAC1	10422	T	F	F
RBM14	10432	F	F	T
SEMA4D	10507	T	F	F
CEBPB	1051	F	F	T
CIB1	10519	F	F	T
BATF	10538	T	F	F
ARFGEF2	10564	F	T	F
RABAC1	10567	T	T	T
SH2B2	10603	F	F	T
PDLIM5	10611	F	F	T
TXNIP	10628	T	F	F
RGS14	10636	T	T	F
EXOC5	10640	F	T	F
GNA13	10672	T	F	F
RBBP9	10741	F	T	F
CFTR	1080	F	F	T
FRS3	10817	F	F	T
PPARGC1A	10891	F	T	F
GADD45G	10912	F	T	T
RALBP1	10928	F	F	T
MORF4L1	10933	F	T	F
CBX1	10951	T	F	F
STIP1	10963	F	T	T
RCC1	1104	F	F	T
WWP1	11059	T	T	F
ADCY5	111	F	T	F
CIT	11113	T	F	F
DMC1	11144	F	T	T
MAP4K1	11184	T	F	F
CHM	1121	T	F	F
AKAP11	11215	F	T	F
AKAP2	11217	F	T	F
DDX20	11218	F	T	F
CTRC	11330	F	T	F

PDAP1	11333	T	F	F
SIGLEC11	114132	T	T	F
SLAMF6	114836	F	F	T
CISH	1154	F	F	T
UHRF2	115426	F	T	F
AGAP2	116986	F	F	T
CPXM2	119587	T	F	F
SERPINA3	12	T	T	F
JDP2	122953	F	T	F
CNGB1	1258	T	F	F
CNN1	1264	F	T	F
CNTN1	1272	F	T	F
COPA	1314	F	F	T
COX11	1353	F	T	F
GAB3	139716	T	F	T
CRKL	1399	T	F	T
PARP1	142	T	F	T
CD300LF	146722	T	F	F
ADRA1A	148	T	F	F
CTBP1	1487	F	T	F
AANAT	15	F	T	F
CTNND1	1500	T	F	T
NFAM1	150372	F	F	T
CTRB1	1504	F	T	F
CTRL	1506	T	F	F
CTSB	1508	T	F	F
BTLA	151888	F	F	T
AMOT	154796	T	F	F
DAB1	1600	T	F	F
DAB2	1601	T	T	F
AP2A2	161	F	T	F
DAPK3	1613	F	T	F
SPRED1	161742	F	F	T
DCTN1	1639	T	T	T
DDX5	1655	F	T	T
DIAPH1	1729	T	F	F
DLG2	1740	F	T	F
DLG4	1742	F	T	T
DLX3	1747	F	F	T
DNM1	1759	F	T	F
DNM2	1785	T	F	F
DOK1	1796	F	T	F
ATN1	1822	T	T	F
DSG3	1830	T	F	F
TSC22D3	1831	F	T	F
DUSP1	1843	F	F	T
DUSP3	1845	F	T	T
DUT	1854	F	T	F
E2F5	1875	F	T	F
EFNA1	1942	T	F	F
EFNA4	1945	T	F	F
EPHA2	1969	T	T	F
ELANE	1991	T	F	F
ELAVL4	1996	F	F	T
ELK1	2002	F	F	T
EP300	2033	T	T	F
EPB42	2038	F	T	F
EPRS	2058	F	T	F
ERBB2	2064	T	T	T

ERF	2077	T	F	F
ERG	2078	T	T	T
ETV3	2117	F	T	F
MECOM	2122	F	T	F
EWSR1	2130	F	T	F
F2	2147	T	T	T
F2RL2	2151	T	F	F
F8	2157	F	T	T
F9	2158	T	F	F
F10	2159	T	F	F
F11	2160	T	F	F
PTK2B	2185	T	F	T
FER	2241	F	T	F
ABCD2	225	F	F	T
FGF9	2254	T	F	F
FGFR2	2263	T	F	F
FGR	2268	T	T	T
FHL3	2275	F	F	T
RRAS2	22800	F	T	F
ICK	22858	T	F	F
FOXG1	2290	F	F	T
HABP4	22927	T	F	T
PAXIP1	22976	T	F	F
AKR1B1	231	F	T	F
FLG	2312	F	F	T
ABRAXAS2	23172	F	F	T
FLNC	2318	T	T	T
FLT1	2321	F	T	F
ITGB3BP	23421	F	T	F
CD2AP	23607	T	F	T
CBLC	23624	T	F	F
TRIM29	23650	T	F	F
KCNE4	23704	F	T	F
MAFF	23764	F	F	T
ABL1	25	T	F	T
NR5A1	2516	F	T	F
GAB1	2549	F	T	F
GABRG2	2566	F	T	F
GAD2	2572	F	T	F
RAD54B	25788	T	F	T
SUMF2	25870	F	F	T
GORASP2	26003	F	F	T
PTPN18	26469	T	T	F
GDI1	2664	T	F	F
GDI2	2665	F	T	T
RGL4	266747	T	F	T
GHR	2690	F	T	F
NBEA	26960	F	T	F
LAT	27040	T	F	F
GJB1	2705	F	T	F
CACYBP	27101	F	F	T
ZBTB11	27107	F	F	T
SIT1	27240	F	F	T
APEX2	27301	F	F	T
ANGPTL3	27329	F	T	F
GLI1	2735	T	F	F
GLI2	2736	F	T	F
GLI3	2737	T	T	T
POLM	27434	F	T	F

GMFB	2764	T	F	F
GOLGA1	2800	F	F	T
GP1BA	2811	F	T	F
GP5	2814	F	F	T
ANG	283	F	T	F
DLL1	28514	F	T	F
GRK5	2869	F	F	T
GRB7	2886	T	T	F
GRB10	2887	T	F	T
GRIA3	2892	T	F	F
GRIN1	2902	F	F	T
GRIN2D	2906	F	F	T
ZBTB44	29068	F	F	T
FHOD1	29109	T	F	F
GTF2I	2969	T	F	F
BLNK	29760	F	F	T
PILRA	29992	T	T	F
GUCY2D	3000	T	F	F
SH3KBP1	30011	F	T	F
HCLS1	3059	T	F	F
HDAC1	3065	F	T	T
HGFAC	3083	T	F	F
HMGB2	3148	T	F	F
HMGA1	3159	F	T	F
APBB1	322	T	F	F
APC	324	T	T	T
HRAS	3265	F	T	F
ERAS	3266	F	T	F
APEX1	328	T	T	T
DNAJB2	3300	F	T	F
HSPA8	3312	F	T	F
TNC	3371	F	F	T
APOB	338	T	F	T
IFNAR1	3454	T	T	T
IFNB1	3456	F	T	F
IFNGR1	3459	T	F	F
APOE	348	T	F	T
KLK3	354	T	F	T
IL6ST	3572	F	T	T
IL13RA1	3597	F	T	F
INS	3630	F	F	T
INPP5D	3635	T	T	F
INPPL1	3636	T	F	F
ITGA2B	3674	T	F	F
ITGB2	3689	T	T	T
ARAF	369	T	F	T
ITGB3	3690	T	T	T
EIF6	3692	F	T	F
JAK1	3716	T	F	F
JUN	3725	T	T	T
JUNB	3726	T	F	F
JUND	3727	T	T	T
JUP	3728	T	T	T
KCNA2	3737	F	T	T
KCNA4	3739	F	T	F
KCND2	3751	F	T	F
KCNJ1	3758	F	F	T
KDR	3791	F	T	F
KIT	3815	T	T	T

KPNB1	3837	F	F	T
KPNA2	3838	T	T	F
KRT7	3855	T	F	F
RHOA	387	T	T	T
RHOB	388	F	T	T
RHOC	389	T	F	T
RHOG	391	T	F	F
LCP2	3937	T	F	F
LDLR	3949	T	F	T
ARHGDIB	397	F	T	F
MYO18A	399687	T	F	T
LRP1	4035	T	T	T
LRP2	4036	F	F	T
LRP5	4041	F	F	T
LSAMP	4045	T	F	F
LYN	4067	T	T	T
NBR1	4077	T	F	T
SMAD1	4086	T	T	F
SMAD2	4087	T	F	T
SMAD3	4088	F	T	T
SMAD4	4089	T	T	T
SMAD5	4090	T	F	T
SMAD7	4092	T	T	T
SMAD9	4093	T	T	T
MAF	4094	T	F	F
MAFG	4097	T	F	F
MATK	4145	F	T	F
MC2R	4158	F	F	T
MDK	4192	F	T	F
MDM4	4194	F	T	F
RAB8A	4218	F	T	F
MEP1B	4225	T	F	F
MMP7	4316	T	F	F
MMP13	4322	T	F	F
MMP15	4324	F	T	F
MNAT1	4331	F	T	F
MOG	4340	F	T	F
MSH3	4437	F	F	T
MSH4	4438	F	T	F
MST1R	4486	F	T	T
MTTP	4547	F	F	T
MUC4	4585	F	T	F
MUTYH	4595	F	F	T
MYC	4609	F	T	T
MYLK	4638	F	F	T
ATF4	468	F	T	F
ATM	472	F	F	T
NEK3	4752	T	F	T
NF2	4771	F	F	T
NFE2L2	4780	F	F	T
NFX1	4799	F	F	T
NOTCH1	4851	T	F	F
NOTCH4	4855	T	F	F
CCN3	4856	T	T	T
NRGN	4900	T	F	F
NTRK1	4914	T	F	F
NTRK3	4916	T	F	F
ROR2	4920	F	F	T
OAZ1	4946	F	F	T

ODC1	4953	F	F	T
OPCML	4978	F	F	T
OPRK1	4986	T	F	F
OSM	5008	F	T	T
PEBP1	5037	T	F	T
FURIN	5045	T	F	F
PCSK6	5046	F	F	T
SERPINE1	5054	T	T	F
PAK1	5058	T	T	T
DEF6	50619	F	F	T
ARHGEF3	50650	F	T	F
PAM	5066	F	F	T
PAPPA	5069	F	T	F
PAX2	5076	T	T	F
PAX5	5079	T	F	F
F11R	50848	F	T	F
TRAT1	50852	T	F	F
SERPINA5	5104	T	T	F
PCNA	5111	T	T	T
GP6	51206	F	T	F
ZDHHC3	51304	F	T	F
PDC	5132	T	F	F
FZR1	51343	F	T	F
CHST15	51363	F	F	T
UBR5	51366	F	T	F
ZNF107	51427	T	F	F
GULP1	51454	F	T	F
PDE6D	5147	F	F	T
PDGFA	5154	T	F	F
PDGFB	5155	F	F	T
PDGFRA	5156	T	T	T
PDGFRB	5159	T	T	T
PDHA1	5160	F	F	T
PDHB	5162	F	F	T
PDPK1	5170	T	F	F
PECAM1	5175	T	F	T
RAB8B	51762	F	F	T
PFKFB2	5208	F	T	F
PFN1	5216	T	F	F
PFN2	5217	T	F	F
PGD	5226	T	F	F
PHB	5245	F	F	T
PHKG1	5260	F	T	F
PHKG2	5261	F	T	F
SERPINA1	5265	T	F	F
SERPINB6	5269	F	T	F
SERPINB8	5271	F	F	T
PIGR	5284	F	T	F
PIK3CB	5291	F	T	T
PIM1	5292	T	F	F
PIK3R1	5295	T	T	T
PIK3R2	5296	T	F	F
PIN1	5300	F	F	T
PKD1	5310	F	F	T
PKD2	5311	T	F	T
PLA2G1B	5319	T	F	T
PLA2G2A	5320	T	F	T
PLAT	5327	T	F	T
PLAU	5328	F	T	T

BCL11A	53335	T	F	F
PLCG1	5335	T	T	T
PLCG2	5336	F	T	F
PLG	5340	T	T	T
PLIN1	5346	F	F	T
PLK1	5347	T	T	F
PLSCR1	5359	T	F	F
PRRX1	5396	T	F	T
RIPK4	54101	T	F	F
POLA1	5422	F	T	F
POLB	5423	F	T	T
POLG	5428	T	F	F
POMC	5443	F	F	T
POU1F1	5449	F	T	F
APBB1IP	54518	T	F	F
PPARA	5465	T	T	T
NDE1	54820	T	F	F
MTMR10	54893	F	T	T
RASIP1	54922	T	F	F
PPM1A	5494	T	T	F
MACO1	55219	F	T	F
PPP2R5A	5525	T	F	F
PPP2R5C	5527	T	F	F
FBXW7	55294	T	F	F
SRGN	5552	F	T	T
PRG2	5553	F	T	F
PPP1R9A	55607	T	F	F
OTUB1	55611	T	F	F
PRKACA	5566	T	T	T
PRPF40A	55660	F	T	F
PRKACB	5567	F	F	T
SCYL2	55681	T	F	F
PKIA	5569	F	F	T
ATF7IP	55729	T	F	T
PRKAR1A	5573	F	F	T
ENAH	55740	T	F	F
PARVA	55742	F	F	T
PRKAR1B	5575	T	F	T
PRKAR2A	5576	T	T	F
EXOC1	55763	F	T	F
PRKAR2B	5577	F	T	F
PRKCA	5578	T	T	T
PRKCB	5579	T	T	T
PRKCD	5580	T	T	T
PRKCE	5581	T	T	T
PRKCG	5582	T	T	T
PAG1	55824	F	F	T
PRKD1	5587	T	F	F
ASH1L	55870	F	T	F
PRKCQ	5588	T	F	F
PRKCZ	5590	T	T	T
PRKDC	5591	F	F	T
MAPK1	5594	T	T	T
MAPK3	5595	T	T	F
MAPK4	5596	T	F	F
MAPK13	5603	F	T	F
PDGFC	56034	F	T	F
MAP2K1	5604	T	F	T
EIF2AK2	5610	T	T	T

DNAJC3	5611	T	F	F
PRKX	5613	T	F	F
PRLR	5618	T	F	T
PRNP	5621	T	T	T
PROC	5624	F	T	T
PROS1	5627	T	T	F
PRPH	5630	F	F	T
PRSS2	5645	T	F	F
PRTN3	5657	F	T	F
PSG9	5678	T	F	F
PSMA3	5684	T	F	T
RAD18	56852	F	T	F
PSMA5	5686	F	T	F
PSMA6	5687	F	F	T
EXOSC5	56915	F	T	F
PSMD11	5717	T	F	F
PTAFR	5724	T	F	F
PTGIR	5739	T	F	F
PTH	5741	T	F	F
PTHLH	5744	F	F	T
PTH1R	5745	T	F	F
PTK2	5747	T	T	T
ESYT2	57488	F	F	T
PTMA	5757	F	T	F
PTPN1	5770	T	T	T
PTPN2	5771	F	F	T
PTPN6	5777	T	T	T
PTPN7	5778	T	F	F
PTPN11	5781	T	T	T
PTPRA	5786	T	T	F
PTPRB	5787	F	T	T
PTPRF	5792	T	T	T
PTPRG	5793	F	F	T
PTPRJ	5795	T	F	F
PTPRK	5796	F	T	F
PTPRM	5797	T	T	F
PTPRO	5800	F	T	F
PTPRR	5801	F	F	T
PTPRS	5802	T	F	F
PVR	5817	T	T	F
ABCD3	5825	F	F	T
PYGM	5837	F	F	T
ZBED5	58486	F	F	T
MYOZ1	58529	T	T	T
SNX6	58533	F	F	T
PZP	5858	F	F	T
RAB1A	5861	F	T	T
RAB2A	5862	F	T	T
RAB3B	5865	F	T	F
RAB4A	5867	F	T	T
RAB5A	5868	T	T	F
RAB5B	5869	T	T	F
RAB6A	5870	T	T	T
RABGGTA	5875	F	T	F
RABGGTB	5876	F	F	T
RAC1	5879	F	T	F
RAD51	5888	T	T	T
RAG1	5896	T	T	F
RAG2	5897	T	F	F

RALA	5898	T	T	T
RALB	5899	T	F	T
BCHE	590	T	F	T
RALGDS	5900	F	T	F
RAP1A	5906	T	T	F
RAP1B	5908	T	F	F
RAP1GDS1	5910	F	T	F
RAP2A	5911	T	T	T
RAP2B	5912	F	F	T
RARA	5914	F	T	F
RARG	5916	F	T	F
RARS	5917	F	T	F
RB1	5925	T	T	T
RBBP6	5930	F	T	F
RBBP8	5932	F	T	F
RBL2	5934	F	T	F
TRPV4	59341	F	T	F
CCND1	595	T	T	T
RBP4	5950	T	F	F
RCVRN	5957	F	F	T
PRPH2	5961	T	F	T
REL	5966	T	T	F
RELA	5970	F	T	F
RFX1	5989	T	F	F
RGS4	5999	T	F	F
RHO	6010	F	T	F
AFAP1	60312	T	F	F
RNASE1	6035	T	T	F
EXOC4	60412	T	F	F
RNH1	6050	T	T	F
ROCK1	6093	F	F	T
ROM1	6094	T	F	F
RP1	6101	T	F	F
RPA1	6117	F	T	F
RPL18A	6142	F	F	T
RPS3A	6189	T	F	F
RPS6KA2	6196	T	F	F
RPS6KA3	6197	F	T	F
RPS7	6201	F	F	T
RRAD	6236	T	T	T
RRAS	6237	T	F	T
RRBP1	6238	F	F	T
RTKN	6242	F	T	F
RXRA	6256	T	T	T
RYR2	6262	T	F	F
S100A1	6271	F	F	T
S100A2	6273	T	T	F
S100A4	6275	F	F	T
S100B	6285	T	F	F
SCN3A	6328	F	T	F
BGN	633	F	F	T
SCTR	6344	F	T	T
CCL3	6348	F	T	F
BICD1	636	T	F	F
SDC4	6385	F	F	T
SELE	6401	T	F	F
SEMG1	6406	F	T	T
SEMG2	6407	T	T	F
BLM	641	F	T	F

SET	6418	T	F	F
COP1	64326	T	T	T
SHB	6461	T	F	F
SHC1	6464	F	T	T
ACBD3	64746	F	F	T
SMURF2	64750	T	T	T
SIAH1	6477	F	F	T
RMND5B	64777	F	F	T
RMND5A	64795	F	T	F
SKIL	6498	T	T	F
SLAMF1	6504	F	T	F
SLC2A4	6517	T	F	T
NCF1	653361	F	F	T
SLC6A8	6535	F	T	F
SFTPA1	653509	T	F	T
SLC9A1	6548	F	F	T
SLPI	6590	T	F	T
SNRNP70	6625	F	T	F
SNRPA	6626	F	F	T
SOX5	6660	T	F	F
SP1	6667	F	T	T
SPARC	6678	T	F	F
SPI1	6688	T	T	T
SPIB	6689	T	T	F
SPTAN1	6709	T	T	F
SRC	6714	T	T	F
SRF	6722	T	F	F
SSBP1	6742	T	F	F
SSTR2	6752	F	T	F
STAT1	6772	F	F	T
STAT5B	6777	F	F	T
SYK	6850	T	T	T
TAF1	6872	F	F	T
TAGLN	6876	T	F	F
TARBP2	6895	F	F	T
TBP	6908	T	T	F
TCF20	6942	F	T	F
BTK	695	F	T	F
TCTA	6988	T	F	F
BUB1	699	F	T	F
DYNLT3	6990	F	T	F
TEC	7006	F	T	T
TEK	7010	F	T	F
TFAP2A	7020	T	T	F
NR2F2	7026	T	F	T
TFRC	7037	T	F	F
TGFBR1	7046	F	F	T
TGIF1	7050	F	F	T
THBS1	7057	F	T	T
TIAM1	7074	T	F	F
NKX2-1	7080	T	F	T
TLE3	7090	F	F	T
TLE4	7091	T	F	F
TLN1	7094	F	F	T
SERPING1	710	F	F	T
TP53	7157	F	T	T
TPM2	7169	T	T	F
TPM3	7170	T	F	F
TPR	7175	T	F	F

TRAF3	7187	T	F	F
TRIO	7204	F	F	T
TRIP6	7205	T	F	F
DNAJC7	7266	F	T	F
TTN	7273	F	F	T
TTR	7276	T	F	F
TUBA4A	7277	T	F	F
PTMAP12	728026	F	T	F
SFTPA2	729238	T	F	F
TXN	7295	F	F	T
TYK2	7297	T	T	T
C7	730	F	T	F
TYRO3	7301	F	F	T
UBE2B	7320	F	T	F
UBE2G1	7326	F	F	T
UBE2I	7329	F	F	T
SUMO1	7341	T	F	F
UBTF	7343	F	T	T
USP4	7375	F	T	F
VAV1	7409	F	T	T
VAV2	7410	T	T	F
VCL	7414	F	T	T
TRPV1	7442	F	T	F
VTN	7448	F	T	F
WNT5A	7474	F	F	T
WRN	7486	F	F	T
WT1	7490	F	T	F
XRCC5	7520	F	F	T
YES1	7525	T	T	T
YWHAG	7532	F	F	T
YWHAZ	7534	T	T	T
ZAP70	7535	T	T	T
ZIC1	7545	T	T	F
ZIC2	7546	F	T	F
DDR1	780	F	T	F
LAPTM5	7805	T	T	F
DAP3	7818	T	F	F
PRKRIP1	79706	T	F	F
CALM1	801	F	T	F
ADAM12	8038	T	F	F
CALM2	805	F	T	F
CALM3	808	F	T	F
ACTN4	81	T	F	F
CALR	811	F	F	T
SF3A2	8175	F	T	F
NCOA3	8202	F	F	T
NRIP1	8204	T	F	F
CANX	821	T	F	F
CAPZA1	829	T	F	F
COLQ	8292	T	F	T
HIST2H2BE	8349	F	F	T
RASSF5	83593	T	F	F
CASP3	836	T	T	F
KRTAP4-12	83755	T	T	F
CASP6	839	T	F	F
CASP7	840	T	F	F
EEA1	8411	T	T	F
SLA2	84174	F	T	F
CASP9	842	T	F	F

NR0B2	8431	F	T	F
NAA38	84316	T	F	F
NCK2	8440	F	T	T
MEGF11	84465	F	T	F
CASR	846	T	F	T
PARD6B	84612	T	F	F
SORBS2	8470	T	F	F
IRS4	8471	F	T	F
PIK3R3	8503	F	T	F
KCNAB2	8514	F	F	T
DGKZ	8525	F	F	T
MICALL1	85377	F	T	F
PPFIA3	8541	F	F	T
BLZF1	8548	F	T	F
PIAS1	8554	T	F	T
THOC5	8563	F	T	F
CAV1	857	F	F	T
RUVBL1	8607	T	F	F
NCOA1	8648	T	F	T
SOCS1	8651	F	F	T
DYNLL1	8655	F	T	F
IRS2	8660	F	F	T
EIF3C	8663	F	T	F
CBL	867	T	T	T
TNK1	8711	F	F	T
ADAM9	8754	T	F	F
MPDZ	8777	F	T	F
ACTN2	88	T	T	F
TRIM24	8805	T	F	F
SOCS2	8835	T	F	F
HDAC3	8841	F	F	T
NR1I2	8856	F	T	F
PROZ	8858	F	F	T
IER3	8870	F	T	F
KRIT1	889	F	T	F
AP1M1	8907	F	T	F
HERC1	8925	F	T	F
BTRC	8945	F	F	T
CCNT2	905	F	T	F
PIGQ	9091	F	T	F
DNAJA3	9093	T	T	F
RABEP1	9135	F	T	T
DYRK1B	9149	T	F	F
UBXN11	91544	F	T	F
CD3E	916	T	F	F
OSMR	9180	F	T	F
RASSF9	9182	F	F	T
BUB3	9184	F	F	T
DDX21	9188	F	F	T
CD4	920	T	T	F
CD7	924	T	F	T
CYTH2	9266	F	T	F
TRIP10	9322	F	T	F
CD163	9332	F	T	F
SLC9A3R1	9368	T	F	F
ZFYVE9	9372	T	T	T
SYTL5	94122	T	F	F
HAND1	9421	F	F	T
PPP1R14A	94274	F	F	T

NCR1	9437	T	F	T
MAPK8IP1	9479	T	F	F
CD36	948	F	T	F
AKAP5	9495	F	T	F
BCAR1	9564	T	T	T
GTPBP1	9567	F	F	T
SOX13	9580	F	T	T
CD44	960	T	F	T
PDIA4	9601	T	F	F
NCOR1	9611	T	F	F
NUP155	9631	T	F	T
FEZ1	9638	T	F	F
CTR9	9646	F	F	T
CD59	966	T	T	T
RIMS2	9699	F	T	F
CD72	971	T	F	F
NOS1AP	9722	F	T	F
CD81	975	F	T	F
RAPGEF5	9771	T	F	F
ELMO1	9844	T	F	F
GAB2	9846	T	F	F
SETDB1	9869	F	T	T
CDC6	990	F	T	F
NR1I3	9970	F	T	F
CDH1	999	T	T	F
DGCR2	9993	T	F	F

Supplementary Table 5a. MCODE module enrichment with rat data.

Module	Algorithm	Rat data	OR	Size of overlap	p-value
Toxicity module	MCODE	Carboplatin 72hours	3.07	5	2.44E-02
Toxicity module	MCODE	Gemcitabine 72hours	4.36	23	3.93E-09
Neutropenia module	MCODE	Carboplatin 72hours	2.19	8	3.07E-02
Neutropenia module	MCODE	Gemcitabine 72hours	3.14	37	8.48E-10
Leukopenia module	MCODE	Carboplatin 72hours	2.78	10	3.43E-03
Leukopenia module	MCODE	Gemcitabine 72hours	2.75	32	2.52E-07
Thrombocytopenia module	MCODE	Carboplatin 72hours	2.76	9	5.68E-03
Thrombocytopenia module	MCODE	Gemcitabine 72hours	2.47	26	2.38E-05
Leukopenia seedlist	MCODE	Carboplatin 72hours	0.08	8	7.86E-01
Leukopenia seedlist	MCODE	Gemcitabine 72hours	1.28	43	5.96E-02
Neutropenia seedlist	MCODE	Carboplatin 72hours	1.26	12	2.49E-01
Neutropenia seedlist	MCODE	Gemcitabine 72hours	1.27	41	6.78E-01
Thrombocytopenia seedlist	MCODE	Carboplatin 72hours	0.57	6	9.57E-01
Thrombocytopenia seedlist	MCODE	Gemcitabine 72hours	0.98	35	5.79E-01

Supplementary Table 5b. The other algorithms, DIAMOnD, CliqueSuM, and ModuleDiscoverer, for module constructions enrichment with rat data.

Toxicity phenotype module	Algorithm	Rat data	OR
Leukopenia module	DIAMOnD	Carboplatin 72hours	2.72
Leukopenia module	CliqueSuM	Carboplatin 72hours	1.54
Leukopenia module	ModuleDiscoverer	Carboplatin 72hours	0.00
Leukopenia module	DIAMOnD	Gemcitabine 72hours	2.35
Leukopenia module	CliqueSuM	Gemcitabine 72hours	1.51
Leukopenia module	ModuleDiscoverer	Gemcitabine 72hours	0.70
Neutropenia module	DIAMOnD	Carboplatin 72hours	2.25
Neutropenia module	CliqueSuM	Carboplatin 72hours	1.68
Neutropenia module	ModuleDiscoverer	Carboplatin 72hours	0.00
Neutropenia module	DIAMOnD	Gemcitabine 72hours	2.00
Neutropenia module	CliqueSuM	Gemcitabine 72hours	1.60
Neutropenia module	ModuleDiscoverer	Gemcitabine 72hours	0.00
Thrombocytopenia module	DIAMOnD	Carboplatin 72hours	2.22
Thrombocytopenia module	CliqueSuM	Carboplatin 72hours	1.49
Thrombocytopenia module	ModuleDiscoverer	Carboplatin 72hours	0.23
Thrombocytopenia module	DIAMOnD	Gemcitabine 72hours	2.23
Thrombocytopenia module	CliqueSuM	Gemcitabine 72hours	1.57
Thrombocytopenia module	ModuleDiscoverer	Gemcitabine 72hours	0.00

Supplementary Table 6. Genes from the toxicity module expressed with a TPM > 1 in ≥ 2 cell line samples.

Gene ID	Gene Name	Ensembl ID
10000	AKT3	ENSG00000117020
10062	NR1H3	ENSG00000025434
10140	TOB1	ENSG00000141232
10419	PRMT5	ENSG00000100462
10567	RABAC1	ENSG00000105404
10636	RGS14	ENSG00000169220
10963	STIP1	ENSG00000168439
11059	WWP1	ENSG00000123124
139716	GAB3	ENSG00000160219
1399	CRKL	ENSG00000099942
142	PARP1	ENSG00000143799
1500	CTNND1	ENSG00000198561
1601	DAB2	ENSG00000153071
1639	DCTN1	ENSG00000204843
1655	DDX5	ENSG00000108654
1742	DLG4	ENSG00000132535
1822	ATN1	ENSG00000111676
1845	DUSP3	ENSG00000108861
2033	EP300	ENSG00000100393
2078	ERG	ENSG00000157554
2185	PTK2B	ENSG00000120899
2268	FGR	ENSG00000000938
2318	FLNC	ENSG00000128591
23607	CD2AP	ENSG00000198087
25	ABL1	ENSG00000097007
2665	GDI2	ENSG00000072135
266747	RGL4	ENSG00000057608
29992	PILRA	ENSG00000106070
3065	HDAC1	ENSG00000085514
324	APC	ENSG00000116478
328	APEX1	ENSG00000134982
338	APOB	ENSG00000100823
348	APOE	ENSG00000142166
354	KLK3	ENSG00000130203
3635	INPP5D	ENSG00000134352
3689	ITGB2	ENSG00000168918
369	ARAF	ENSG00000160255
3690	ITGB3	ENSG00000078061
3725	JUN	ENSG00000259207
3727	JUND	ENSG00000177606
3728	JUP	ENSG00000130522
3737	KCNA2	ENSG00000173801
3838	KPNA2	ENSG00000157404
387	RHOA	ENSG00000182481
388	RHOB	ENSG00000067560
389	RHOC	ENSG00000143878

3949	LDLR	ENSG00000155366
399687	MYO18A	ENSG00000130164
4035	LRP1	ENSG00000196535
4077	NBR1	ENSG00000254087
4086	SMAD1	ENSG00000188554
4087	SMAD2	ENSG00000170365
4088	SMAD3	ENSG00000175387
4089	SMAD4	ENSG00000166949
4090	SMAD5	ENSG00000141646
4092	SMAD7	ENSG00000113658
4093	SMAD9	ENSG00000101665
4752	NEK3	ENSG00000136997
4856	NOV	ENSG00000136098
5037	PEBP1	ENSG00000099985
5054	SERPINE1	ENSG00000089220
5076	PAX2	ENSG00000149269
5156	PDGFRA	ENSG00000132646
5175	PECAM1	ENSG00000113721
5291	PIK3CB	ENSG00000051382
5295	PIK3R1	ENSG00000145675
5311	PKD2	ENSG00000118762
5319	PLA2G1B	ENSG00000170890
5328	PLAU	ENSG00000122861
5335	PLCG1	ENSG00000124181
5347	PLK1	ENSG00000166851
5423	POLB	ENSG00000070501
5465	PPARA	ENSG00000186951
54893	MTMR10	ENSG00000166912
5494	PPM1A	ENSG00000100614
5552	SRGN	ENSG00000122862
5566	PRKACA	ENSG00000072062
55729	ATF7IP	ENSG00000171681
5575	PRKAR1B	ENSG00000188191
5576	PRKAR2A	ENSG00000114302
5578	PRKCA	ENSG00000154229
5579	PRKCB	ENSG00000166501
5580	PRKCD	ENSG00000163932
5594	MAPK1	ENSG00000100030
5595	MAPK3	ENSG00000102882
5604	MAP2K1	ENSG00000169032
5610	EIF2AK2	ENSG00000055332
5621	PRNP	ENSG00000171867
5684	PSMA3	ENSG00000100567
5747	PTK2	ENSG00000169398
5770	PTPN1	ENSG00000196396
5777	PTPN6	ENSG00000111679
5781	PTPN11	ENSG00000179295
5786	PTPRA	ENSG00000132670
5792	PTPRF	ENSG00000142949
5817	PVR	ENSG00000073008

5861	RAB1A	ENSG00000138069
5862	RAB2A	ENSG00000104388
5867	RAB4A	ENSG00000168118
5868	RAB5A	ENSG00000144566
5869	RAB5B	ENSG00000111540
5870	RAB6A	ENSG00000175582
5888	RAD51	ENSG00000051180
5898	RALA	ENSG00000006451
5899	RALB	ENSG00000144118
5906	RAP1A	ENSG00000116473
5911	RAP2A	ENSG00000125249
5925	RB1	ENSG00000139687
5966	REL	ENSG00000162924
6035	RNASE1	ENSG00000129538
6050	RNH1	ENSG00000023191
6237	RRAS	ENSG00000126458
6256	RXRA	ENSG00000186350
64326	RFWD2	ENSG00000143207
6464	SHC1	ENSG00000160691
64750	SMURF2	ENSG00000108854
6498	SKIL	ENSG00000136603
6667	SP1	ENSG00000185591
6688	SPI1	ENSG00000066336
6709	SPTAN1	ENSG00000197694
6714	SRC	ENSG00000197122
6850	SYK	ENSG00000165025
6908	TBP	ENSG00000112592
7006	TEC	ENSG00000135605
7020	TFAP2A	ENSG00000137203
7026	NR2F2	ENSG00000185551
7057	THBS1	ENSG00000137801
7157	TP53	ENSG00000141510
7169	TPM2	ENSG00000198467
7297	TYK2	ENSG00000105397
7343	UBTF	ENSG00000108312
7409	VAV1	ENSG00000141968
7414	VCL	ENSG00000035403
7525	YES1	ENSG00000176105
7534	YWHAZ	ENSG00000164924
7805	LAPTM5	ENSG00000162511
836	CASP3	ENSG00000164305
8411	EEA1	ENSG00000102189
8440	NCK2	ENSG00000071051
8554	PIAS1	ENSG00000033800
8648	NCOA1	ENSG00000084676
867	CBL	ENSG00000110395
9093	DNAJA3	ENSG00000103423
9135	RABEP1	ENSG00000029725
920	CD4	ENSG00000010610
924	CD7	ENSG00000173762

9372	ZFYVE9	ENSG00000157077
9564	BCAR1	ENSG00000050820
960	CD44	ENSG00000026508
9631	NUP155	ENSG00000113569
966	CD59	ENSG00000085063
9869	SETDB1	ENSG00000143379

Supplementary Table 7. Differentially expressed toxicity module genes in the treated cell lines. A negative fold change corresponds to a decreased expression in the treated cells compared to the untreated control cells.

Gene ID	Gene Name	Ensambl ID	log_2(fold change)	p	Cell line	Treatment
2268	FGR	ENSG00000000938	-2.84	0.0215	K562	Carboplatin
4086	SMAD1	ENSG00000170365	-2.37	0.000436	K562	Carboplatin
3635	INPP5D	ENSG00000168918	-1.35	0.00165	K562	Carboplatin
7409	VAV1	ENSG00000141968	-1.2	0.0216	K562	Carboplatin
1601	DAB2	ENSG00000153071	0.76	0.0273	K562	Carboplatin
10140	TOB1	ENSG00000141232	0.77	0.00805	K562	Carboplatin
966	CD59	ENSG00000085063	0.98	0.0225	K562	Carboplatin
5008	OSM	ENSG00000099985	4.62	0.0416	K562	Gemcitabine
5159	PDGFRB	ENSG00000113721	-6.06	0.0123	CMK	Gemcitabine
3725	JUN	ENSG00000177606	3.17	0.0000714	MOLM-1	Carboplatin
5319	PLA2G1B	ENSG00000170890	-7.05	0.0000568	MOLM-1	Gemcitabine
2078	ERG	ENSG00000157554	-0.8	0.0428	MOLM-1	Gemcitabine
5347	PLK1	ENSG00000166851	1.05	0.0263	MOLM-1	Gemcitabine
2887	GRB10	ENSG00000106070	1.21	0.0106	MOLM-1	Gemcitabine
5621	PRNP	ENSG00000171867	1.37	0.0337	MOLM-1	Gemcitabine
3725	JUN	ENSG00000177606	2.83	0.000038	MOLM-1	Gemcitabine
6035	RNASE1	ENSG00000129538	2.98	0.0286	MOLM-1	Gemcitabine
7057	THBS1	ENSG00000137801	3.03	0.0415	MOLM-1	Gemcitabine
348	APOE	ENSG00000130203	3.07	0.00821	MOLM-1	Gemcitabine

Supplementary Table 8. Enriched KEGG pathways and Gene Ontologies (GOs), with an FDR adjusted p-value < 0.01.

ID	Description	GeneRatio	BackgroundRatio	p-value	FDR adjusted p-value (p.adjust)	q-value	geneID	Count	Source
hsa05205	Proteoglycans in cancer	34/168	204/7888	1.52E-21	3.31E-19	1.09E-19	369/387/595/836	34	KEGG
hsa04012	ErbB signaling pathway	22/168	85/7888	1.67E-18	1.81E-16	5.97E-17	25/369/867/1399	22	KEGG
hsa04015	Rap1 signaling pathway	31/168	210/7888	4.43E-18	3.21E-16	1.06E-16	387/999/1399/15	31	KEGG
hsa05220	Chronic myeloid leukemia	20/168	76/7888	4.60E-17	2.50E-15	8.23E-16	25/369/595/867/	20	KEGG
hsa05210	Colorectal cancer	20/168	86/7888	6.44E-16	2.45E-14	8.08E-15	324/369/387/595	20	KEGG
hsa04014	Ras signaling pathway	30/168	232/7888	7.04E-16	2.45E-14	8.08E-15	25/387/1969/381	30	KEGG
hsa04510	Focal adhesion	28/168	199/7888	7.90E-16	2.45E-14	8.08E-15	387/595/1399/20	28	KEGG
hsa04650	Natural killer cell mediated cytotoxicity	23/168	131/7888	2.71E-15	7.36E-14	2.43E-14	369/836/2185/34	23	KEGG
hsa05212	Pancreatic cancer	18/168	75/7888	1.09E-14	2.36E-13	7.80E-14	369/595/2064/40	18	KEGG
hsa05214	Glioma	18/168	75/7888	1.09E-14	2.36E-13	7.80E-14	369/595/5156/51	18	KEGG
hsa05223	Non-small cell lung cancer	17/168	66/7888	1.72E-14	3.40E-13	1.12E-13	369/595/2064/52	17	KEGG
hsa05161	Hepatitis B	24/168	162/7888	3.42E-14	6.18E-13	2.04E-13	369/836/2033/21	24	KEGG
hsa04666	Fc gamma R-mediated phagocytosis	19/168	93/7888	4.60E-14	7.67E-13	2.53E-13	1399/3635/4067/	19	KEGG
hsa04520	Adherens junction	17/168	71/7888	6.48E-14	1.00E-12	3.31E-13	387/999/1500/20	17	KEGG
hsa04919	Thyroid hormone signaling pathway	20/168	119/7888	4.74E-13	6.86E-12	2.26E-12	595/2033/3065/3	20	KEGG
hsa05215	Prostate cancer	18/168	97/7888	1.28E-12	1.73E-11	5.72E-12	354/369/595/203	18	KEGG
hsa04670	Leukocyte transendothelial migration	19/168	112/7888	1.59E-12	2.02E-11	6.68E-12	387/1003/1500/2	19	KEGG
hsa04933	AGE-RAGE signaling pathway in diabetic complications	18/168	100/7888	2.21E-12	2.67E-11	8.79E-12	595/836/3725/40	18	KEGG
hsa05226	Gastric cancer	21/168	149/7888	4.18E-12	4.77E-11	1.57E-11	324/369/595/999	21	KEGG
hsa01521	EGFR tyrosine kinase inhibitor resistance	16/168	79/7888	5.90E-12	6.40E-11	2.11E-11	369/2064/5156/5	16	KEGG
hsa04062	Chemokine signaling pathway	23/168	189/7888	8.44E-12	8.72E-11	2.88E-11	387/1399/2185/2	23	KEGG
hsa05160	Hepatitis C	21/168	155/7888	9.11E-12	8.99E-11	2.97E-11	369/595/836/345	21	KEGG
hsa05213	Endometrial cancer	14/168	58/7888	1.03E-11	9.74E-11	3.21E-11	324/369/595/999	14	KEGG
hsa01522	Endocrine resistance	17/168	98/7888	1.74E-11	1.57E-10	5.19E-11	369/595/2064/37	17	KEGG
hsa05219	Bladder cancer	12/168	41/7888	2.72E-11	2.36E-10	7.78E-11	369/595/999/206	12	KEGG
hsa05203	Viral carcinogenesis	23/168	201/7888	3.05E-11	2.49E-10	8.22E-11	387/595/836/203	23	KEGG
hsa04072	Phospholipase D signaling pathway	20/168	148/7888	3.10E-11	2.49E-10	8.22E-11	387/2147/2185/3	20	KEGG
hsa04660	T cell receptor signaling pathway	17/168	103/7888	3.99E-11	3.09E-10	1.02E-10	387/920/3725/50	17	KEGG
hsa05225	Hepatocellular carcinoma	21/168	168/7888	4.38E-11	3.28E-10	1.08E-10	324/369/595/408	21	KEGG
hsa05163	Human cytomegalovirus infection	24/168	225/7888	4.91E-11	3.55E-10	1.17E-10	387/595/836/139	24	KEGG
hsa05206	MicroRNAs in cancer	28/168	310/7888	5.65E-11	3.96E-10	1.31E-10	25/324/387/595/	28	KEGG
hsa04350	TGF-beta signaling pathway	16/168	94/7888	9.53E-11	6.32E-10	2.09E-10	387/2033/4086/4	16	KEGG
hsa04010	MAPK signaling pathway	27/168	295/7888	9.61E-11	6.32E-10	2.09E-10	369/836/1399/18	27	KEGG
hsa05224	Breast cancer	19/168	147/7888	2.19E-10	1.40E-09	4.62E-10	324/369/595/206	19	KEGG
hsa05218	Melanoma	14/168	72/7888	2.35E-10	1.46E-09	4.80E-10	369/595/999/515	14	KEGG
hsa05167	Kaposi sarcoma-associated herpesvirus infection	21/168	186/7888	3.05E-10	1.84E-09	6.06E-10	595/836/2033/34	21	KEGG
hsa05135	Yersinia infection	17/168	121/7888	5.52E-10	3.23E-09	1.07E-09	387/1399/2185/3	17	KEGG
hsa04810	Regulation of actin cytoskeleton	22/168	213/7888	6.31E-10	3.60E-09	1.19E-09	324/369/387/139	22	KEGG
hsa04664	Fc epsilon RI signaling pathway	13/168	68/7888	1.32E-09	7.36E-09	2.43E-09	3635/4067/5291/	13	KEGG
hsa04662	B cell receptor signaling pathway	14/168	82/7888	1.44E-09	7.80E-09	2.57E-09	3635/3725/4067/	14	KEGG
hsa05211	Renal cell carcinoma	13/168	69/7888	1.60E-09	8.47E-09	2.79E-09	369/1399/2033/3	13	KEGG
hsa04370	VEGF signaling pathway	12/168	59/7888	2.76E-09	1.43E-08	4.71E-09	5291/5295/5335/	12	KEGG
hsa04722	Neurotrophin signaling pathway	16/168	119/7888	3.53E-09	1.78E-08	5.87E-09	25/387/1399/372	16	KEGG
hsa04910	Insulin signaling pathway	17/168	137/7888	3.92E-09	1.93E-08	6.37E-09	369/867/1399/52	17	KEGG
hsa04550	Signaling pathways regulating pluripotency of stem cells	17/168	140/7888	5.48E-09	2.64E-08	8.72E-09	324/3572/4086/4	17	KEGG
hsa05166	Human T-cell leukemia virus 1 infection	21/168	219/7888	6.19E-09	2.92E-08	9.63E-09	595/920/2033/36	21	KEGG
hsa05221	Acute myeloid leukemia	12/168	66/7888	1.07E-08	4.93E-08	1.63E-08	369/595/3728/38	12	KEGG

hsa05231	Choline metabolism in cancer	14/168	98/7888	1.59E-08	7.18E-08	2.37E-08	3725/5156/5159/	14	KEGG
hsa05230	Central carbon metabolism in cancer	12/168	69/7888	1.81E-08	8.01E-08	2.64E-08	2064/3815/4609/	12	KEGG
hsa05100	Bacterial invasion of epithelial cells	12/168	73/7888	3.51E-08	1.52E-07	5.02E-08	387/867/999/139	12	KEGG
hsa05216	Thyroid cancer	9/168	37/7888	5.43E-08	2.31E-07	7.62E-08	595/999/4609/55	9	KEGG
hsa04068	FoxO signaling pathway	15/168	131/7888	1.01E-07	4.21E-07	1.39E-07	369/595/2033/40	15	KEGG
hsa04625	C-type lectin receptor signaling pathway	13/168	104/7888	2.65E-07	1.09E-06	3.58E-07	387/3725/5058/5	13	KEGG
hsa04630	JAK-STAT signaling pathway	16/168	162/7888	2.96E-07	1.19E-06	3.93E-07	595/2033/3454/3	16	KEGG
hsa04110	Cell cycle	14/168	124/7888	3.27E-07	1.26E-06	4.16E-07	25/595/2033/306	14	KEGG
hsa04611	Platelet activation	14/168	124/7888	3.27E-07	1.26E-06	4.16E-07	387/2147/3690/4	14	KEGG
hsa04928	Parathyroid hormone synthesis, secretion and action	13/168	106/7888	3.33E-07	1.26E-06	4.16E-07	369/387/846/372	13	KEGG
hsa05235	PD-L1 expression and PD-1 checkpoint pathway in cancer	12/168	89/7888	3.37E-07	1.26E-06	4.16E-07	920/3725/5291/5	12	KEGG
hsa04151	PI3K-Akt signaling pathway	24/168	354/7888	4.02E-07	1.48E-06	4.87E-07	595/1969/2064/3	24	KEGG
hsa04931	Insulin resistance	13/168	108/7888	4.15E-07	1.50E-06	4.95E-07	5291/5295/5465/	13	KEGG
hsa05165	Human papillomavirus infection	23/168	330/7888	4.41E-07	1.57E-06	5.18E-07	324/595/836/203	23	KEGG
hsa04066	HIF-1 signaling pathway	13/168	109/7888	4.63E-07	1.62E-06	5.34E-07	2033/2064/5054/	13	KEGG
hsa04380	Osteoclast differentiation	14/168	128/7888	4.86E-07	1.67E-06	5.52E-07	3454/3690/3725/	14	KEGG
hsa04610	Complement and coagulation cascades	11/168	79/7888	7.61E-07	2.58E-06	8.51E-07	966/2147/2157/3	11	KEGG
hsa04371	Apelin signaling pathway	14/168	137/7888	1.12E-06	3.74E-06	1.23E-06	595/999/4087/40	14	KEGG
hsa05169	Epstein-Barr virus infection	17/168	201/7888	1.14E-06	3.76E-06	1.24E-06	595/836/960/306	17	KEGG
hsa04720	Long-term potentiation	10/168	67/7888	1.28E-06	4.10E-06	1.35E-06	369/2033/5566/5	10	KEGG
hsa04071	Sphingolipid signaling pathway	13/168	119/7888	1.28E-06	4.10E-06	1.35E-06	387/5291/5295/5	13	KEGG
hsa04360	Axon guidance	16/168	181/7888	1.34E-06	4.21E-06	1.39E-06	25/387/1969/505	16	KEGG
hsa04218	Cellular senescence	15/168	160/7888	1.39E-06	4.29E-06	1.42E-06	595/4087/4088/4	15	KEGG
hsa05146	Amoebiasis	12/168	102/7888	1.51E-06	4.61E-06	1.52E-06	836/3689/5291/5	12	KEGG
hsa04917	Prolactin signaling pathway	10/168	70/7888	1.94E-06	5.84E-06	1.93E-06	595/5291/5295/5	10	KEGG
hsa05170	Human immunodeficiency virus 1 infection	17/168	212/7888	2.40E-06	7.12E-06	2.35E-06	836/920/1399/21	17	KEGG
hsa04659	Th17 cell differentiation	12/168	107/7888	2.52E-06	7.40E-06	2.44E-06	920/3572/3725/4	12	KEGG
hsa04024	cAMP signaling pathway	17/168	214/7888	2.73E-06	7.88E-06	2.60E-06	387/2033/2737/3	17	KEGG
hsa05164	Influenza A	15/168	170/7888	2.98E-06	8.51E-06	2.81E-06	836/2033/3454/3	15	KEGG
hsa04926	Relaxin signaling pathway	13/168	129/7888	3.22E-06	9.07E-06	2.99E-06	3725/4087/5291/	13	KEGG
hsa05222	Small cell lung cancer	11/168	93/7888	3.97E-06	1.10E-05	3.64E-06	595/836/4609/52	11	KEGG
hsa04270	Vascular smooth muscle contraction	13/168	132/7888	4.17E-06	1.14E-05	3.78E-06	369/387/5319/53	13	KEGG
hsa04390	Hippo signaling pathway	14/168	154/7888	4.55E-06	1.23E-05	4.07E-06	324/595/999/174	14	KEGG
hsa04930	Type II diabetes mellitus	8/168	46/7888	4.69E-06	1.26E-05	4.15E-06	5291/5295/5580/	8	KEGG
hsa04915	Estrogen signaling pathway	13/168	138/7888	6.83E-06	1.81E-05	5.97E-06	3725/5291/5295/	13	KEGG
hsa04929	GnRH secretion	9/168	64/7888	7.34E-06	1.91E-05	6.31E-06	5291/5295/5578/	9	KEGG
hsa05418	Fluid shear stress and atherosclerosis	13/168	139/7888	7.40E-06	1.91E-05	6.31E-06	387/1003/3690/3	13	KEGG
hsa05202	Transcriptional misregulation in cancer	15/168	186/7888	9.04E-06	2.31E-05	7.61E-06	1655/2078/3065/	15	KEGG
hsa04960	Aldosterone-regulated sodium reabsorption	7/168	37/7888	1.05E-05	2.66E-05	8.77E-06	5291/5295/5578/	7	KEGG
hsa04144	Endocytosis	17/168	243/7888	1.50E-05	3.74E-05	1.23E-05	387/867/1601/39	17	KEGG
hsa04540	Gap junction	10/168	88/7888	1.58E-05	3.91E-05	1.29E-05	5156/5159/5566/	10	KEGG
hsa04912	GnRH signaling pathway	10/168	93/7888	2.59E-05	6.31E-05	2.08E-05	2185/3725/5566/	10	KEGG
hsa04210	Apoptosis	12/168	136/7888	3.03E-05	7.30E-05	2.41E-05	142/836/3725/52	12	KEGG
hsa04310	Wnt signaling pathway	13/168	160/7888	3.38E-05	8.07E-05	2.66E-05	324/387/595/203	13	KEGG
hsa04730	Long-term depression	8/168	60/7888	3.57E-05	8.42E-05	2.78E-05	369/4067/5578/5	8	KEGG
hsa04750	Inflammatory mediator regulation of TRP channels	10/168	100/7888	4.88E-05	1.14E-04	3.75E-05	5291/5295/5335/	10	KEGG
hsa05020	Prion diseases	6/168	35/7888	8.30E-05	1.92E-04	6.32E-05	5566/5594/5595/	6	KEGG
hsa04725	Cholinergic synapse	10/168	112/7888	1.28E-04	2.92E-04	9.65E-05	5291/5295/5566/	10	KEGG
hsa01524	Platinum drug resistance	8/168	73/7888	1.48E-04	3.35E-04	1.11E-04	836/2064/5291/5	8	KEGG

hsa04914	Progesterone-mediated oocyte maturation	9/168	99/7888	2.44E-04	5.46E-04	1.80E-04	369/5291/5295/5	9	KEGG
hsa04916	Melanogenesis	9/168	101/7888	2.84E-04	6.28E-04	2.07E-04	2033/3815/5566/	9	KEGG
hsa04150	mTOR signaling pathway	11/168	153/7888	4.03E-04	8.75E-04	2.89E-04	387/2887/5291/5	11	KEGG
hsa04921	Oxytocin signaling pathway	11/168	153/7888	4.03E-04	8.75E-04	2.89E-04	387/595/3725/55	11	KEGG
hsa05152	Tuberculosis	12/168	179/7888	4.19E-04	9.01E-04	2.97E-04	387/836/2033/36	12	KEGG
hsa05131	Shigellosis	7/168	67/7888	5.21E-04	1.11E-03	3.65E-04	25/960/1399/559	7	KEGG
hsa04140	Autophagy - animal	10/168	137/7888	6.57E-04	1.38E-03	4.57E-04	5291/5295/5566/	10	KEGG
hsa05120	Epithelial cell signaling in Helicobacter pylori infection	7/168	70/7888	6.81E-04	1.42E-03	4.68E-04	836/3725/4067/5	7	KEGG
hsa05162	Measles	10/168	138/7888	6.96E-04	1.44E-03	4.74E-04	595/836/3454/37	10	KEGG
hsa04726	Serotonergic synapse	9/168	115/7888	7.41E-04	1.52E-03	5.01E-04	369/836/5566/55	9	KEGG
hsa04115	p53 signaling pathway	7/168	72/7888	8.07E-04	1.64E-03	5.40E-04	595/836/5054/70	7	KEGG
hsa05130	Pathogenic Escherichia coli infection	6/168	54/7888	9.46E-04	1.90E-03	6.27E-04	25/387/999/5578	6	KEGG
hsa05133	Pertussis	7/168	76/7888	1.12E-03	2.22E-03	7.33E-04	387/836/3689/37	7	KEGG
hsa04972	Pancreatic secretion	8/168	102/7888	1.44E-03	2.81E-03	9.26E-04	387/5319/5320/5	8	KEGG
hsa05142	Chagas disease (American trypanosomiasis)	8/168	102/7888	1.44E-03	2.81E-03	9.26E-04	3725/4087/5054/	8	KEGG
hsa04620	Toll-like receptor signaling pathway	8/168	104/7888	1.63E-03	3.15E-03	1.04E-03	3454/3725/5291/	8	KEGG
hsa04934	Cushing syndrome	10/168	155/7888	1.69E-03	3.25E-03	1.07E-03	324/595/3949/55	10	KEGG
hsa04137	Mitophagy - animal	6/168	65/7888	2.50E-03	4.75E-03	1.57E-03	3725/4077/6237/	6	KEGG
hsa04668	TNF signaling pathway	8/168	112/7888	2.60E-03	4.91E-03	1.62E-03	836/3725/5291/5	8	KEGG
hsa05031	Amphetamine addiction	6/168	68/7888	3.14E-03	5.87E-03	1.94E-03	3065/3725/5566/	6	KEGG
hsa04530	Tight junction	10/168	169/7888	3.20E-03	5.93E-03	1.96E-03	387/595/2064/37	10	KEGG
hsa04658	Th1 and Th2 cell differentiation	7/168	92/7888	3.37E-03	6.19E-03	2.04E-03	920/3725/5335/5	7	KEGG
hsa03410	Base excision repair	4/168	33/7888	5.02E-03	9.14E-03	3.01E-03	142/328/5111/54	4	KEGG
hsa04070	Phosphatidylinositol signaling system	7/168	99/7888	5.05E-03	9.14E-03	3.01E-03	3635/5291/5295/	7	KEGG
hsa05140	Leishmaniasis	6/168	76/7888	5.45E-03	9.78E-03	3.23E-03	3689/3725/5579/	6	KEGG
GO:0050817	coagulation	40/212	337/17913	1.44E-28	5.91E-25	3.06E-25	APOE/CD59/F2/F	40	GO
GO:0007596	blood coagulation	38/212	331/17913	1.31E-26	2.69E-23	1.39E-23	APOE/CD59/F2/F	38	GO
GO:0007599	hemostasis	38/212	336/17913	2.29E-26	3.13E-23	1.62E-23	APOE/CD59/F2/F	38	GO
GO:0018108	peptidyl-tyrosine phosphorylation	38/212	346/17913	6.76E-26	6.93E-23	3.59E-23	ABL1/CD4/CD44/	38	GO
GO:0018212	peptidyl-tyrosine modification	38/212	349/17913	9.29E-26	7.62E-23	3.95E-23	ABL1/CD4/CD44/	38	GO
GO:0050878	regulation of body fluid levels	42/212	499/17913	5.37E-24	3.67E-21	1.90E-21	APOE/CCND1/CD	42	GO
GO:0070371	ERK1 and ERK2 cascade	33/212	289/17913	4.36E-23	2.56E-20	1.32E-20	ABL1/APOE/CASF	33	GO
GO:0070372	regulation of ERK1 and ERK2 cascade	31/212	271/17913	9.49E-22	4.86E-19	2.52E-19	ABL1/APOE/CASF	31	GO
GO:0071900	regulation of protein serine/threonine kinase activity	38/212	479/17913	7.92E-21	3.61E-18	1.87E-18	ABL1/APC/APOE/	38	GO
GO:0030168	platelet activation	24/212	152/17913	2.23E-20	9.16E-18	4.74E-18	APOE/F2/F8/ITGE	24	GO
GO:0002768	immune response-regulating cell surface receptor signaling pathway	34/212	434/17913	1.62E-18	6.05E-16	3.13E-16	ABL1/CD4/CRKL/I	34	GO
GO:0007179	transforming growth factor beta receptor signaling pathway	24/212	184/17913	2.24E-18	7.65E-16	3.97E-16	PARP1/RHOA/CB	24	GO
GO:0071560	cellular response to transforming growth factor beta stimulus	26/212	233/17913	4.21E-18	1.27E-15	6.60E-16	PARP1/RHOA/CB	26	GO
GO:0022407	regulation of cell-cell adhesion	32/212	389/17913	4.35E-18	1.27E-15	6.60E-16	ABL1/RHOA/CASI	32	GO
GO:0006909	phagocytosis	29/212	308/17913	4.77E-18	1.30E-15	6.76E-16	ABL1/FGR/ITGB2/	29	GO
GO:0071559	response to transforming growth factor beta	26/212	239/17913	7.96E-18	2.04E-15	1.06E-15	PARP1/RHOA/CB	26	GO
GO:0007178	transmembrane receptor protein serine/threonine kinase signaling pathway	29/212	323/17913	1.74E-17	4.20E-15	2.17E-15	ABL1/PARP1/RHC	29	GO
GO:0002429	immune response-activating cell surface receptor signaling pathway	31/212	399/17913	7.68E-17	1.75E-14	9.07E-15	ABL1/CD4/CRKL/I	31	GO
GO:0043410	positive regulation of MAPK cascade	34/212	499/17913	1.17E-16	2.52E-14	1.31E-14	ABL1/APOE/CASF	34	GO
GO:0050730	regulation of peptidyl-tyrosine phosphorylation	25/212	245/17913	1.64E-16	3.37E-14	1.74E-14	ABL1/CD4/CD44/	25	GO
GO:1901653	cellular response to peptide	29/212	364/17913	4.28E-16	8.35E-14	4.33E-14	ACTN2/PARP1/AI	29	GO
GO:0061564	axon development	31/212	438/17913	1.04E-15	1.95E-13	1.01E-13	ABL1/APOE/RHO/	31	GO
GO:1903037	regulation of leukocyte cell-cell adhesion	26/212	295/17913	1.42E-15	2.37E-13	1.23E-13	RHOA/CASP3/CD	26	GO
GO:0042110	T cell activation	31/212	443/17913	1.43E-15	2.37E-13	1.23E-13	ABL1/RHOA/CASI	31	GO

GO:1903034	regulation of response to wounding	21/212	173/17913	1.45E-15	2.37E-13	1.23E-13	APOE/F2/LRP1/LY	21	GO
GO:0007159	leukocyte cell-cell adhesion	27/212	328/17913	2.13E-15	3.36E-13	1.74E-13	RHOA/CASP3/CD	27	GO
GO:0045785	positive regulation of cell adhesion	29/212	388/17913	2.30E-15	3.49E-13	1.81E-13	ABL1/RHOA/CD4/	29	GO
GO:0002694	regulation of leukocyte activation	32/212	492/17913	3.79E-15	5.54E-13	2.87E-13	ABL1/RHOA/CAS	32	GO
GO:0032386	regulation of intracellular transport	29/212	400/17913	5.10E-15	7.20E-13	3.73E-13	ACTN2/CDH1/DA	29	GO
GO:0030193	regulation of blood coagulation	15/212	74/17913	7.50E-15	1.02E-12	5.31E-13	APOE/F2/LYN/SE	15	GO
GO:1900046	regulation of hemostasis	15/212	75/17913	9.28E-15	1.23E-12	6.36E-13	APOE/F2/LYN/SE	15	GO
GO:0043405	regulation of MAP kinase activity	26/212	322/17913	1.16E-14	1.48E-12	7.68E-13	APOE/CRKL/DUS	26	GO
GO:0038093	Fc receptor signaling pathway	23/212	241/17913	1.22E-14	1.52E-12	7.87E-13	ABL1/FGR/JUN/K	23	GO
GO:0071375	cellular response to peptide hormone stimulus	25/212	297/17913	1.49E-14	1.80E-12	9.33E-13	ACTN2/PARP1/A	25	GO
GO:0050818	regulation of coagulation	15/212	79/17913	2.10E-14	2.47E-12	1.28E-12	APOE/F2/LYN/SE	15	GO
GO:1901652	response to peptide	31/212	491/17913	2.38E-14	2.68E-12	1.39E-12	ACTN2/PARP1/A	31	GO
GO:0007409	axonogenesis	28/212	393/17913	2.42E-14	2.68E-12	1.39E-12	ABL1/APOE/RHO	28	GO
GO:0034330	cell junction organization	24/212	277/17913	2.74E-14	2.95E-12	1.53E-12	ABL1/ACTN2/AP	24	GO
GO:0050863	regulation of T cell activation	25/212	307/17913	3.20E-14	3.36E-12	1.74E-12	ABL1/RHOA/CAS	25	GO
GO:0050900	leukocyte migration	30/212	467/17913	4.13E-14	4.24E-12	2.19E-12	APOB/RHOA/CD4	30	GO
GO:0051222	positive regulation of protein transport	28/212	403/17913	4.54E-14	4.54E-12	2.35E-12	ABL1/CASR/CDH1	28	GO
GO:0002433	immune response-regulating cell surface receptor signaling pathway involved in phagocytosis	18/212	139/17913	5.29E-14	5.04E-12	2.61E-12	ABL1/FGR/LYN/P	18	GO
GO:0038096	Fc-gamma receptor signaling pathway involved in phagocytosis	18/212	139/17913	5.29E-14	5.04E-12	2.61E-12	ABL1/FGR/LYN/P	18	GO
GO:1904951	positive regulation of establishment of protein localization	29/212	440/17913	5.94E-14	5.54E-12	2.87E-12	ABL1/CASR/CDH1	29	GO
GO:0038094	Fc-gamma receptor signaling pathway	18/212	141/17913	6.82E-14	6.21E-12	3.22E-12	ABL1/FGR/LYN/P	18	GO
GO:1903829	positive regulation of cellular protein localization	25/212	318/17913	7.14E-14	6.36E-12	3.30E-12	PARP1/APC/CDH	25	GO
GO:0070374	positive regulation of ERK1 and ERK2 cascade	20/212	186/17913	7.38E-14	6.44E-12	3.34E-12	ABL1/APOE/CAS	20	GO
GO:0002431	Fc receptor mediated stimulatory signaling pathway	18/212	142/17913	7.73E-14	6.60E-12	3.42E-12	ABL1/FGR/LYN/P	18	GO
GO:0061041	regulation of wound healing	18/212	143/17913	8.75E-14	7.32E-12	3.79E-12	APOE/F2/LYN/S	18	GO
GO:0031589	cell-substrate adhesion	25/212	322/17913	9.48E-14	7.77E-12	4.03E-12	ABL1/ACTN2/RH	25	GO
GO:0007160	cell-matrix adhesion	21/212	214/17913	1.06E-13	8.35E-12	4.32E-12	ABL1/ACTN2/RH	21	GO
GO:0034329	cell junction assembly	21/212	214/17913	1.06E-13	8.35E-12	4.32E-12	ABL1/ACTN2/AP	21	GO
GO:0010631	epithelial cell migration	26/212	354/17913	1.08E-13	8.35E-12	4.32E-12	ABL1/APOE/RHO	26	GO
GO:0002521	leukocyte differentiation	30/212	485/17913	1.11E-13	8.44E-12	4.37E-12	ABL1/PARP1/RH	30	GO
GO:0090132	epithelium migration	26/212	357/17913	1.31E-13	9.80E-12	5.07E-12	ABL1/APOE/RHO	26	GO
GO:0090130	tissue migration	26/212	363/17913	1.94E-13	1.42E-11	7.35E-12	ABL1/APOE/RHO	26	GO
GO:0032147	activation of protein kinase activity	24/212	303/17913	1.98E-13	1.42E-11	7.37E-12	ABL1/CRKL/PTK2	24	GO
GO:0048667	cell morphogenesis involved in neuron differentiation	30/212	500/17913	2.45E-13	1.73E-11	8.97E-12	ABL1/APOE/RHO	30	GO
GO:0043434	response to peptide hormone	27/212	404/17913	3.38E-13	2.35E-11	1.22E-11	ACTN2/PARP1/A	27	GO
GO:0034332	adherens junction organization	17/212	134/17913	3.84E-13	2.62E-11	1.36E-11	ABL1/ACTN2/RH	17	GO
GO:0060627	regulation of vesicle-mediated transport	29/212	480/17913	5.36E-13	3.60E-11	1.87E-11	ABL1/ACTN2/AP	29	GO
GO:0001667	ameboidal-type cell migration	28/212	446/17913	5.51E-13	3.64E-11	1.89E-11	ABL1/APOE/RHO	28	GO
GO:0007411	axon guidance	20/212	210/17913	7.39E-13	4.68E-11	2.42E-11	ERBB2/GLI3/GRB	20	GO
GO:0046777	protein autophosphorylation	20/212	210/17913	7.39E-13	4.68E-11	2.42E-11	ABL1/ERBB2/PTK	20	GO
GO:0071902	positive regulation of protein serine/threonine kinase activity	24/212	322/17913	7.41E-13	4.68E-11	2.42E-11	RHOA/CCND1/CR	24	GO
GO:0097485	neuron projection guidance	20/212	212/17913	8.83E-13	5.49E-11	2.84E-11	ERBB2/GLI3/GRB	20	GO
GO:1903039	positive regulation of leukocyte cell-cell adhesion	20/212	214/17913	1.05E-12	6.45E-11	3.34E-11	RHOA/CD4/CD44	20	GO
GO:0007264	small GTPase mediated signal transduction	28/212	465/17913	1.51E-12	9.13E-11	4.73E-11	ABL1/APOE/RHO	28	GO
GO:1903706	regulation of hemopoiesis	28/212	468/17913	1.77E-12	1.05E-10	5.44E-11	ABL1/RHOA/CD4/	28	GO
GO:0051249	regulation of lymphocyte activation	26/212	401/17913	1.91E-12	1.12E-10	5.80E-11	ABL1/RHOA/CAS	26	GO
GO:0022409	positive regulation of cell-cell adhesion	21/212	251/17913	2.41E-12	1.39E-10	7.20E-11	RHOA/CD4/CD44	21	GO
GO:0061045	negative regulation of wound healing	13/212	73/17913	2.85E-12	1.63E-10	8.42E-11	APOE/F2/SMAD3	13	GO
GO:1900182	positive regulation of protein localization to nucleus	13/212	74/17913	3.43E-12	1.92E-10	9.97E-11	PARP1/CDH1/F2/	13	GO

GO:0048010	vascular endothelial growth factor receptor signaling pathway	14/212	93/17913	4.48E-12	2.48E-10	1.29E-10	RHOA/PTK2B/GR	14	GO
GO:0050867	positive regulation of cell activation	23/212	319/17913	4.55E-12	2.49E-10	1.29E-10	RHOA/CD4/FGR/(	23	GO
GO:0048732	gland development	26/212	422/17913	6.07E-12	3.27E-10	1.70E-10	ABL1/CCND1/CDI	26	GO
GO:0050731	positive regulation of peptidyl-tyrosine phosphorylation	18/212	184/17913	6.92E-12	3.68E-10	1.91E-10	ABL1/CD4/CD44/	18	GO
GO:0043550	regulation of lipid kinase activity	12/212	62/17913	7.10E-12	3.71E-10	1.92E-10	F2/PTK2B/FGR/KI	12	GO
GO:0048015	phosphatidylinositol-mediated signaling	17/212	160/17913	7.15E-12	3.71E-10	1.92E-10	RHOA/CBL/ERBB2	17	GO
GO:0014065	phosphatidylinositol 3-kinase signaling	16/212	138/17913	7.89E-12	4.04E-10	2.09E-10	CBL/ERBB2/F2/FC	16	GO
GO:0001952	regulation of cell-matrix adhesion	15/212	117/17913	8.31E-12	4.15E-10	2.15E-10	ABL1/RHOA/DUS	15	GO
GO:0014066	regulation of phosphatidylinositol 3-kinase signaling	15/212	117/17913	8.31E-12	4.15E-10	2.15E-10	CBL/F2/FGR/KIT/I	15	GO
GO:0090218	positive regulation of lipid kinase activity	10/212	36/17913	8.47E-12	4.18E-10	2.17E-10	F2/PTK2B/FGR/KI	10	GO
GO:0048017	inositol lipid-mediated signaling	17/212	163/17913	9.66E-12	4.61E-10	2.39E-10	RHOA/CBL/ERBB2	17	GO
GO:0030195	negative regulation of blood coagulation	11/212	49/17913	9.67E-12	4.61E-10	2.39E-10	APOE/F2/SERPINI	11	GO
GO:0060395	SMAD protein signal transduction	11/212	49/17913	9.67E-12	4.61E-10	2.39E-10	PARP1/DAB2/JUN	11	GO
GO:0014068	positive regulation of phosphatidylinositol 3-kinase signaling	13/212	81/17913	1.14E-11	5.39E-10	2.79E-10	CBL/F2/FGR/KIT/I	13	GO
GO:1900047	negative regulation of hemostasis	11/212	50/17913	1.23E-11	5.72E-10	2.96E-10	APOE/F2/SERPINI	11	GO
GO:0002696	positive regulation of leukocyte activation	22/212	305/17913	1.35E-11	6.24E-10	3.23E-10	RHOA/CD4/FGR/(	22	GO
GO:0043542	endothelial cell migration	21/212	275/17913	1.38E-11	6.29E-10	3.26E-10	ABL1/APOE/RHO.	21	GO
GO:0045834	positive regulation of lipid metabolic process	16/212	144/17913	1.52E-11	6.86E-10	3.55E-10	APOE/F2/PTK2B/	16	GO
GO:0043406	positive regulation of MAP kinase activity	20/212	250/17913	1.86E-11	8.31E-10	4.30E-10	CRKL/ERBB2/PTK	20	GO
GO:0050851	antigen receptor-mediated signaling pathway	19/212	224/17913	2.20E-11	9.70E-10	5.03E-10	ABL1/CD4/CRKL/I	19	GO
GO:0050870	positive regulation of T cell activation	18/212	198/17913	2.38E-11	1.04E-09	5.39E-10	RHOA/CD4/GLI3/	18	GO
GO:0050819	negative regulation of coagulation	11/212	53/17913	2.43E-11	1.05E-09	5.43E-10	APOE/F2/SERPINI	11	GO
GO:1903035	negative regulation of response to wounding	13/212	87/17913	2.93E-11	1.25E-09	6.48E-10	APOE/F2/SMAD3	13	GO
GO:0010810	regulation of cell-substrate adhesion	18/212	201/17913	3.07E-11	1.30E-09	6.72E-10	ABL1/RHOA/CRKI	18	GO
GO:0032388	positive regulation of intracellular transport	18/212	203/17913	3.62E-11	1.51E-09	7.84E-10	ACTN2/CDH1/DA	18	GO
GO:0071901	negative regulation of protein serine/threonine kinase activity	15/212	136/17913	7.47E-11	3.09E-09	1.60E-09	ABL1/APC/APOE/	15	GO
GO:0007162	negative regulation of cell adhesion	20/212	271/17913	8.01E-11	3.29E-09	1.70E-09	ABL1/RHOA/CASI	20	GO
GO:0043552	positive regulation of phosphatidylinositol 3-kinase activity	9/212	32/17913	8.51E-11	3.46E-09	1.79E-09	PTK2B/FGR/KIT/L	9	GO
GO:0038127	ERBB signaling pathway	15/212	138/17913	9.22E-11	3.70E-09	1.92E-09	ABL1/CBL/DUSP3	15	GO
GO:0046824	positive regulation of nucleocytoplasmic transport	11/212	60/17913	1.02E-10	4.04E-09	2.09E-09	CDH1/GLI3/JUP/S	11	GO
GO:0033627	cell adhesion mediated by integrin	11/212	61/17913	1.23E-10	4.83E-09	2.50E-09	CRKL/EPHA2/ITGI	11	GO
GO:0032868	response to insulin	19/212	252/17913	1.69E-10	6.59E-09	3.42E-09	PARP1/APC/GRB1	19	GO
GO:0042326	negative regulation of phosphorylation	25/212	455/17913	1.83E-10	7.09E-09	3.67E-09	ABL1/APC/APOE/	25	GO
GO:1903727	positive regulation of phospholipid metabolic process	10/212	48/17913	1.93E-10	7.38E-09	3.82E-09	PTK2B/FGR/KIT/L	10	GO
GO:0032869	cellular response to insulin stimulus	17/212	197/17913	1.98E-10	7.53E-09	3.90E-09	PARP1/APC/GRB1	17	GO
GO:0009636	response to toxic substance	26/212	499/17913	2.44E-10	9.17E-09	4.75E-09	ABL1/APEX1/APC	26	GO
GO:0051251	positive regulation of lymphocyte activation	19/212	258/17913	2.52E-10	9.40E-09	4.87E-09	RHOA/CD4/GLI3/	19	GO
GO:0007265	Ras protein signal transduction	21/212	324/17913	2.94E-10	1.09E-08	5.63E-09	ABL1/APOE/RHO.	21	GO
GO:0050804	modulation of chemical synaptic transmission	23/212	393/17913	3.02E-10	1.10E-08	5.70E-09	ABL1/APOE/BCHE	23	GO
GO:0090316	positive regulation of intracellular protein transport	15/212	150/17913	3.03E-10	1.10E-08	5.70E-09	CDH1/ERBB2/GLI	15	GO
GO:0030098	lymphocyte differentiation	21/212	325/17913	3.11E-10	1.12E-08	5.80E-09	ABL1/RHOA/CD4,	21	GO
GO:0099177	regulation of trans-synaptic signaling	23/212	394/17913	3.17E-10	1.13E-08	5.85E-09	ABL1/APOE/BCHE	23	GO
GO:0030100	regulation of endocytosis	19/212	262/17913	3.28E-10	1.16E-08	6.00E-09	ABL1/APOE/CBL/	19	GO
GO:0010632	regulation of epithelial cell migration	20/212	294/17913	3.41E-10	1.20E-08	6.19E-09	ABL1/APOE/RHO.	20	GO
GO:0002576	platelet degranulation	14/212	128/17913	3.65E-10	1.27E-08	6.57E-09	SERPINA3/ACTN2	14	GO
GO:1903725	regulation of phospholipid metabolic process	12/212	86/17913	3.92E-10	1.35E-08	7.00E-09	PTK2B/FGR/KIT/L	12	GO
GO:1902105	regulation of leukocyte differentiation	19/212	267/17913	4.51E-10	1.54E-08	7.99E-09	ABL1/RHOA/CD4,	19	GO
GO:0019216	regulation of lipid metabolic process	23/212	402/17913	4.70E-10	1.59E-08	8.24E-09	SERPINA3/APOB/	23	GO
GO:0043551	regulation of phosphatidylinositol 3-kinase activity	10/212	53/17913	5.46E-10	1.83E-08	9.50E-09	PTK2B/FGR/KIT/L	10	GO

GO:0010001	glial cell differentiation	17/212	212/17913	6.22E-10	2.07E-08	1.07E-08	ABL1/RHOA/ERBI	17 GO
GO:0043254	regulation of protein complex assembly	23/212	409/17913	6.57E-10	2.17E-08	1.13E-08	ABL1/PARP1/APC	23 GO
GO:0034333	adherens junction assembly	12/212	90/17913	6.74E-10	2.21E-08	1.14E-08	ABL1/ACTN2/RH(	12 GO
GO:0048008	platelet-derived growth factor receptor signaling pathway	10/212	55/17913	8.02E-10	2.61E-08	1.35E-08	ABL1/CBL/LRP1/F	10 GO
GO:0032102	negative regulation of response to external stimulus	21/212	343/17913	8.30E-10	2.66E-08	1.38E-08	APOE/CDH5/DUS	21 GO
GO:0009755	hormone-mediated signaling pathway	17/212	216/17913	8.31E-10	2.66E-08	1.38E-08	PARP1/RHOA/DA	17 GO
GO:0001933	negative regulation of protein phosphorylation	23/212	416/17913	9.13E-10	2.90E-08	1.50E-08	ABL1/APC/APOE/	23 GO
GO:0033157	regulation of intracellular protein transport	17/212	220/17913	1.10E-09	3.47E-08	1.80E-08	CDH1/ERBB2/GLI	17 GO
GO:0007229	integrin-mediated signaling pathway	12/212	94/17913	1.13E-09	3.53E-08	1.83E-08	ABL1/PTK2B/FGR	12 GO
GO:1900180	regulation of protein localization to nucleus	13/212	117/17913	1.30E-09	4.03E-08	2.09E-08	PARP1/CDH1/F2/	13 GO
GO:0042063	gliogenesis	19/212	285/17913	1.35E-09	4.16E-08	2.16E-08	ABL1/RHOA/CRKI	19 GO
GO:1901342	regulation of vasculature development	23/212	425/17913	1.38E-09	4.22E-08	2.19E-08	ABL1/KLK3/RHOA	23 GO
GO:0048511	rhythmic process	19/212	287/17913	1.52E-09	4.61E-08	2.39E-08	CASP3/DDX5/EP3	19 GO
GO:0010543	regulation of platelet activation	8/212	30/17913	1.58E-09	4.76E-08	2.47E-08	APOE/F2/LYN/PD	8 GO
GO:0010522	regulation of calcium ion transport into cytosol	12/212	98/17913	1.84E-09	5.50E-08	2.85E-08	ABL1/CD4/F2/PTI	12 GO
GO:0030278	regulation of ossification	16/212	199/17913	1.95E-09	5.78E-08	3.00E-08	DDX5/PTK2B/GLI:	16 GO
GO:0035690	cellular response to drug	21/212	362/17913	2.19E-09	6.45E-08	3.34E-08	ABL1/APEX1/RHC	21 GO
GO:0046677	response to antibiotic	20/212	328/17913	2.30E-09	6.73E-08	3.48E-08	ABL1/APEX1/RHC	20 GO
GO:0010594	regulation of endothelial cell migration	17/212	231/17913	2.32E-09	6.73E-08	3.48E-08	ABL1/APOE/RHO/	17 GO
GO:0046822	regulation of nucleocytoplasmic transport	12/212	100/17913	2.33E-09	6.73E-08	3.48E-08	CDH1/GLI3/JUP/S	12 GO
GO:0033628	regulation of cell adhesion mediated by integrin	9/212	45/17913	2.35E-09	6.75E-08	3.50E-08	CRKL/EPHA2/LYN	9 GO
GO:1904018	positive regulation of vasculature development	17/212	232/17913	2.48E-09	7.06E-08	3.66E-08	ABL1/RHOB/CDH	17 GO
GO:0051098	regulation of binding	21/212	368/17913	2.93E-09	8.29E-08	4.30E-08	ABL1/PARP1/APC	21 GO
GO:0050852	T cell receptor signaling pathway	15/212	180/17913	3.87E-09	1.09E-07	5.64E-08	ABL1/CD4/CRKL/I	15 GO
GO:0030099	myeloid cell differentiation	22/212	411/17913	3.93E-09	1.10E-07	5.68E-08	PARP1/CASP3/CD	22 GO
GO:0008286	insulin receptor signaling pathway	13/212	128/17913	3.96E-09	1.10E-07	5.68E-08	APC/GRB10/PAK1	13 GO
GO:0007183	SMAD protein complex assembly	6/212	13/17913	4.10E-09	1.13E-07	5.85E-08	PARP1/SMAD1/S	6 GO
GO:0043401	steroid hormone mediated signaling pathway	15/212	181/17913	4.18E-09	1.14E-07	5.90E-08	PARP1/RHOA/DA	15 GO
GO:0051348	negative regulation of transferase activity	18/212	272/17913	4.19E-09	1.14E-07	5.90E-08	ABL1/APC/APOE/	18 GO
GO:0031532	actin cytoskeleton reorganization	11/212	85/17913	4.88E-09	1.32E-07	6.82E-08	ABL1/RHOA/PTK2	11 GO
GO:0051924	regulation of calcium ion transport	17/212	244/17913	5.32E-09	1.43E-07	7.39E-08	ABL1/RHOA/CASI	17 GO
GO:0045765	regulation of angiogenesis	21/212	383/17913	5.95E-09	1.58E-07	8.20E-08	ABL1/KLK3/RHOA	21 GO
GO:2000146	negative regulation of cell motility	20/212	347/17913	6.01E-09	1.59E-07	8.24E-08	APEX1/APOE/RH(	20 GO
GO:0035821	modification of morphology or physiology of other organism	14/212	160/17913	6.93E-09	1.82E-07	9.44E-08	APOE/CD4/EP30(	14 GO
GO:0050673	epithelial cell proliferation	22/212	425/17913	7.25E-09	1.89E-07	9.81E-08	APOE/CCND1/EPI	22 GO
GO:0048608	reproductive structure development	22/212	426/17913	7.57E-09	1.96E-07	1.02E-07	CCND1/CASP3/CE	22 GO
GO:0070482	response to oxygen levels	21/212	389/17913	7.82E-09	2.02E-07	1.04E-07	RHOA/CASP3/CA:	21 GO
GO:0007568	aging	19/212	318/17913	8.20E-09	2.10E-07	1.09E-07	ABL1/APEX1/INPI	19 GO
GO:0061458	reproductive system development	22/212	429/17913	8.60E-09	2.18E-07	1.13E-07	CCND1/CASP3/CE	22 GO
GO:0018209	peptidyl-serine modification	17/212	252/17913	8.63E-09	2.18E-07	1.13E-07	PARP1/ARAF/CD4	17 GO
GO:0031032	actomyosin structure organization	14/212	163/17913	8.81E-09	2.22E-07	1.15E-07	ABL1/ACTN2/RH(	14 GO
GO:1901888	regulation of cell junction assembly	11/212	90/17913	9.04E-09	2.26E-07	1.17E-07	ABL1/RHOA/DUS	11 GO
GO:0060326	cell chemotaxis	17/212	253/17913	9.16E-09	2.28E-07	1.18E-07	CRKL/EPHA2/PTK	17 GO
GO:0051817	modification of morphology or physiology of other organism involved in symbiotic interaction	12/212	113/17913	9.58E-09	2.37E-07	1.23E-07	APOE/CD4/EP30(	12 GO
GO:0042307	positive regulation of protein import into nucleus	8/212	37/17913	9.70E-09	2.38E-07	1.23E-07	CDH1/GLI3/JUP/S	8 GO
GO:0034504	protein localization to nucleus	16/212	223/17913	1.00E-08	2.45E-07	1.27E-07	PARP1/CDH1/F2/	16 GO
GO:0051493	regulation of cytoskeleton organization	23/212	472/17913	1.01E-08	2.45E-07	1.27E-07	ABL1/ACTN2/APC	23 GO
GO:0042730	fibrinolysis	7/212	25/17913	1.18E-08	2.85E-07	1.48E-07	F2/SERPINE1/PLA	7 GO
GO:0045088	regulation of innate immune response	22/212	440/17913	1.36E-08	3.26E-07	1.69E-07	APOB/APOE/EP3(	22 GO

GO:0038095	Fc-epsilon receptor signaling pathway	14/212	169/17913	1.40E-08	3.34E-07	1.73E-07	JUN/LYN/PAK1/P	14	GO
GO:0051271	negative regulation of cellular component movement	20/212	365/17913	1.41E-08	3.34E-07	1.73E-07	APEX1/APOE/RH	20	GO
GO:0060191	regulation of lipase activity	11/212	94/17913	1.44E-08	3.39E-07	1.76E-07	ABL1/RHOA/RHO	11	GO
GO:1904591	positive regulation of protein import	8/212	39/17913	1.51E-08	3.55E-07	1.84E-07	CDH1/GLI3/JUP/S	8	GO
GO:0000302	response to reactive oxygen species	16/212	230/17913	1.56E-08	3.63E-07	1.88E-07	ABL1/APEX1/APC	16	GO
GO:0007050	cell cycle arrest	16/212	231/17913	1.66E-08	3.81E-07	1.98E-07	ABL1/APC/CCND1	16	GO
GO:0018105	peptidyl-serine phosphorylation	16/212	231/17913	1.66E-08	3.81E-07	1.98E-07	ARAF/CD44/SMA	16	GO
GO:0045766	positive regulation of angiogenesis	15/212	203/17913	1.99E-08	4.55E-07	2.36E-07	ABL1/RHOB/CDH	15	GO
GO:0006874	cellular calcium ion homeostasis	20/212	375/17913	2.22E-08	5.02E-07	2.60E-07	ABL1/APOE/CASP	20	GO
GO:0048545	response to steroid hormone	20/212	375/17913	2.22E-08	5.02E-07	2.60E-07	PARP1/RHOA/BC	20	GO
GO:0034109	homotypic cell-cell adhesion	10/212	77/17913	2.40E-08	5.40E-07	2.80E-07	ITGB3/JUP/LYN/P	10	GO
GO:0040013	negative regulation of locomotion	20/212	377/17913	2.42E-08	5.42E-07	2.81E-07	APEX1/APOE/RH	20	GO
GO:0046626	regulation of insulin receptor signaling pathway	9/212	58/17913	2.48E-08	5.49E-07	2.84E-07	GRB10/PAK1/PIK	9	GO
GO:0070527	platelet aggregation	9/212	58/17913	2.48E-08	5.49E-07	2.84E-07	ITGB3/LYN/PDGF	9	GO
GO:0002446	neutrophil mediated immunity	23/212	499/17913	2.83E-08	6.23E-07	3.23E-07	SERPINA3/RHOA/	23	GO
GO:0048872	homeostasis of number of cells	16/212	241/17913	3.01E-08	6.59E-07	3.42E-07	ABL1/CASP3/INP	16	GO
GO:0090287	regulation of cellular response to growth factor stimulus	17/212	276/17913	3.32E-08	7.24E-07	3.75E-07	ABL1/DAB2/EP30	17	GO
GO:0070997	neuron death	19/212	347/17913	3.34E-08	7.24E-07	3.75E-07	ABL1/PARP1/APC	19	GO
GO:1905475	regulation of protein localization to membrane	14/212	181/17913	3.36E-08	7.25E-07	3.76E-07	CRKL/DAB2/EPHA	14	GO
GO:0031098	stress-activated protein kinase signaling cascade	17/212	278/17913	3.69E-08	7.91E-07	4.10E-07	RHOA/CASR/CRK	17	GO
GO:1902903	regulation of supramolecular fiber organization	18/212	313/17913	3.70E-08	7.91E-07	4.10E-07	ABL1/ACTN2/APC	18	GO
GO:0042742	defense response to bacterium	16/212	245/17913	3.79E-08	8.01E-07	4.15E-07	KLK3/CD4/EPHA2	16	GO
GO:0006606	protein import into nucleus	11/212	103/17913	3.79E-08	8.01E-07	4.15E-07	CDH1/GLI3/JUP/K	11	GO
GO:0001503	ossification	20/212	388/17913	3.90E-08	8.12E-07	4.21E-07	RHOA/CASR/DDX	20	GO
GO:0055074	calcium ion homeostasis	20/212	388/17913	3.90E-08	8.12E-07	4.21E-07	ABL1/APOE/CASP	20	GO
GO:0006875	cellular metal ion homeostasis	22/212	467/17913	3.93E-08	8.12E-07	4.21E-07	ABL1/APOE/CASP	22	GO
GO:0007045	cell-substrate adherens junction assembly	10/212	81/17913	3.94E-08	8.12E-07	4.21E-07	ABL1/ACTN2/RH	10	GO
GO:0048041	focal adhesion assembly	10/212	81/17913	3.94E-08	8.12E-07	4.21E-07	ABL1/ACTN2/RH	10	GO
GO:0007611	learning or memory	16/212	247/17913	4.24E-08	8.70E-07	4.51E-07	APOE/BCHE/CASP	16	GO
GO:0090092	regulation of transmembrane receptor protein serine/threonine kinase signaling pathway	15/212	215/17913	4.28E-08	8.73E-07	4.52E-07	ABL1/PARP1/DAB	15	GO
GO:0072503	cellular divalent inorganic cation homeostasis	20/212	392/17913	4.61E-08	9.36E-07	4.85E-07	ABL1/APOE/CASP	20	GO
GO:0050866	negative regulation of cell activation	14/212	187/17913	5.07E-08	1.02E-06	5.30E-07	APOE/CASP3/DUS	14	GO
GO:0051235	maintenance of location	17/212	286/17913	5.58E-08	1.12E-06	5.81E-07	ABL1/APOB/APO	17	GO
GO:0061614	pri-miRNA transcription by RNA polymerase II	8/212	46/17913	5.98E-08	1.20E-06	6.20E-07	DDX5/JUN/SMAD	8	GO
GO:0006469	negative regulation of protein kinase activity	15/212	222/17913	6.54E-08	1.30E-06	6.74E-07	ABL1/APC/APOE/	15	GO
GO:0030509	BMP signaling pathway	12/212	134/17913	6.57E-08	1.30E-06	6.74E-07	ABL1/DDX5/SMA	12	GO
GO:0006644	phospholipid metabolic process	20/212	402/17913	6.97E-08	1.37E-06	7.12E-07	PTK2B/FGR/INPP	20	GO
GO:0048013	ephrin receptor signaling pathway	10/212	86/17913	7.05E-08	1.38E-06	7.14E-07	RHOA/EPHA2/LY	10	GO
GO:0006979	response to oxidative stress	21/212	442/17913	7.06E-08	1.38E-06	7.14E-07	ABL1/PARP1/APE	21	GO
GO:0070849	response to epidermal growth factor	8/212	47/17913	7.13E-08	1.39E-06	7.18E-07	CBL/DUSP3/ERBB	8	GO
GO:0036293	response to decreased oxygen levels	19/212	365/17913	7.43E-08	1.44E-06	7.44E-07	RHOA/CASP3/CAS	19	GO
GO:0038128	ERBB2 signaling pathway	7/212	32/17913	7.71E-08	1.48E-06	7.69E-07	ERBB2/GRB7/PIK	7	GO
GO:1900076	regulation of cellular response to insulin stimulus	9/212	66/17913	7.93E-08	1.52E-06	7.88E-07	GRB10/PAK1/PIK	9	GO
GO:0030336	negative regulation of cell migration	18/212	332/17913	9.04E-08	1.72E-06	8.93E-07	APEX1/APOE/RH	18	GO
GO:0032956	regulation of actin cytoskeleton organization	17/212	297/17913	9.62E-08	1.83E-06	9.46E-07	ABL1/ACTN2/RH	17	GO
GO:0072507	divalent inorganic cation homeostasis	20/212	412/17913	1.04E-07	1.96E-06	1.02E-06	ABL1/APOE/CASP	20	GO
GO:0017038	protein import	12/212	140/17913	1.07E-07	2.01E-06	1.04E-06	APOE/CDH1/GLI3	12	GO
GO:0022408	negative regulation of cell-cell adhesion	13/212	169/17913	1.12E-07	2.10E-06	1.09E-06	ABL1/CASP3/CDH	13	GO
GO:0050708	regulation of protein secretion	21/212	456/17913	1.19E-07	2.21E-06	1.14E-06	ABL1/APOE/CASP	21	GO

GO:0007173	epidermal growth factor receptor signaling pathway	11/212	115/17913	1.19E-07	2.21E-06	1.14E-06	ABL1/CBL/DUSP3	11	GO
GO:0017015	regulation of transforming growth factor beta receptor signaling pathway	11/212	115/17913	1.19E-07	2.21E-06	1.14E-06	DAB2/EP300/SM/	11	GO
GO:0009991	response to extracellular stimulus	22/212	498/17913	1.21E-07	2.22E-06	1.15E-06	APOE/BCHE/CCN	22	GO
GO:0030217	T cell differentiation	15/212	234/17913	1.30E-07	2.39E-06	1.24E-06	ABL1/RHOA/CD4,	15	GO
GO:0043491	protein kinase B signaling	16/212	268/17913	1.31E-07	2.39E-06	1.24E-06	EPHA2/ERBB2/KI	16	GO
GO:0060193	positive regulation of lipase activity	9/212	70/17913	1.34E-07	2.43E-06	1.26E-06	RHOA/RHOC/KIT/	9	GO
GO:0032970	regulation of actin filament-based process	18/212	341/17913	1.35E-07	2.44E-06	1.26E-06	ABL1/ACTN2/RH	18	GO
GO:1903844	regulation of cellular response to transforming growth factor beta stimulus	11/212	117/17913	1.43E-07	2.57E-06	1.33E-06	DAB2/EP300/SM/	11	GO
GO:0010634	positive regulation of epithelial cell migration	13/212	173/17913	1.48E-07	2.63E-06	1.36E-06	ABL1/RHOB/PTK	13	GO
GO:0051897	positive regulation of protein kinase B signaling	13/212	173/17913	1.48E-07	2.63E-06	1.36E-06	ERBB2/KIT/MST1	13	GO
GO:0060402	calcium ion transport into cytosol	12/212	145/17913	1.57E-07	2.79E-06	1.45E-06	ABL1/CD4/F2/PT	12	GO
GO:0030522	intracellular receptor signaling pathway	16/212	272/17913	1.60E-07	2.83E-06	1.47E-06	PARP1/RHOA/CR	16	GO
GO:1901522	positive regulation of transcription from RNA polymerase II promoter involved in cellular response to chemical stimulus	6/212	22/17913	1.63E-07	2.87E-06	1.49E-06	EP300/SMAD1/S	6	GO
GO:0031667	response to nutrient levels	21/212	466/17913	1.71E-07	3.00E-06	1.56E-06	APOE/BCHE/CCN	21	GO
GO:0000187	activation of MAPK activity	12/212	147/17913	1.83E-07	3.16E-06	1.64E-06	CRKL/KIT/PIK3CB,	12	GO
GO:0071772	response to BMP	12/212	147/17913	1.83E-07	3.16E-06	1.64E-06	ABL1/DDX5/SMA	12	GO
GO:0071773	cellular response to BMP stimulus	12/212	147/17913	1.83E-07	3.16E-06	1.64E-06	ABL1/DDX5/SMA	12	GO
GO:0050853	B cell receptor signaling pathway	8/212	53/17913	1.89E-07	3.26E-06	1.69E-06	ABL1/LYN/PRKCB	8	GO
GO:0051896	regulation of protein kinase B signaling	15/212	241/17913	1.91E-07	3.28E-06	1.70E-06	EPHA2/ERBB2/KI	15	GO
GO:0007044	cell-substrate junction assembly	10/212	96/17913	2.03E-07	3.46E-06	1.79E-06	ABL1/ACTN2/RH	10	GO
GO:0097193	intrinsic apoptotic signaling pathway	16/212	277/17913	2.05E-07	3.49E-06	1.81E-06	ABL1/PARP1/CAS	16	GO
GO:0071383	cellular response to steroid hormone stimulus	15/212	243/17913	2.13E-07	3.61E-06	1.87E-06	PARP1/RHOA/DA	15	GO
GO:0033673	negative regulation of kinase activity	15/212	244/17913	2.24E-07	3.78E-06	1.96E-06	ABL1/APC/APOE/	15	GO
GO:0009896	positive regulation of catabolic process	19/212	392/17913	2.25E-07	3.78E-06	1.96E-06	APC/APOE/DAB2,	19	GO
GO:0051701	interaction with host	14/212	211/17913	2.27E-07	3.80E-06	1.97E-06	CBL/CD4/CDH1/C	14	GO
GO:0001666	response to hypoxia	18/212	354/17913	2.35E-07	3.92E-06	2.03E-06	RHOA/CASP3/CA	18	GO
GO:0001657	ureteric bud development	10/212	98/17913	2.47E-07	4.09E-06	2.12E-06	GLI3/SMAD1/SM	10	GO
GO:0031295	T cell costimulation	8/212	55/17913	2.55E-07	4.19E-06	2.17E-06	LYN/PAK1/PIK3R	8	GO
GO:0042306	regulation of protein import into nucleus	8/212	55/17913	2.55E-07	4.19E-06	2.17E-06	CDH1/GLI3/JUP/S	8	GO
GO:0072163	mesonephric epithelium development	10/212	99/17913	2.72E-07	4.43E-06	2.29E-06	GLI3/SMAD1/SM	10	GO
GO:0072164	mesonephric tubule development	10/212	99/17913	2.72E-07	4.43E-06	2.29E-06	GLI3/SMAD1/SM	10	GO
GO:0045022	early endosome to late endosome transport	7/212	38/17913	2.72E-07	4.43E-06	2.29E-06	DAB2/MAPK1/M	7	GO
GO:0048844	artery morphogenesis	9/212	76/17913	2.76E-07	4.47E-06	2.31E-06	APOB/APOE/LDL	9	GO
GO:0051170	import into nucleus	11/212	125/17913	2.81E-07	4.53E-06	2.35E-06	CDH1/GLI3/JUP/	11	GO
GO:0042113	B cell activation	14/212	215/17913	2.86E-07	4.59E-06	2.38E-06	ABL1/CASP3/EP3	14	GO
GO:0031294	lymphocyte costimulation	8/212	56/17913	2.94E-07	4.71E-06	2.44E-06	LYN/PAK1/PIK3R	8	GO
GO:0060840	artery development	10/212	100/17913	2.99E-07	4.76E-06	2.47E-06	APOB/APOE/GLI3	10	GO
GO:0030010	establishment of cell polarity	11/212	126/17913	3.04E-07	4.82E-06	2.50E-06	RHOA/CDH5/CRK	11	GO
GO:0051668	localization within membrane	11/212	126/17913	3.04E-07	4.82E-06	2.50E-06	APOE/CRKL/DLG	11	GO
GO:0048708	astrocyte differentiation	9/212	77/17913	3.09E-07	4.87E-06	2.52E-06	ABL1/F2/IL6ST/L	9	GO
GO:0002791	regulation of peptide secretion	21/212	483/17913	3.10E-07	4.87E-06	2.52E-06	ABL1/APOE/CAS	21	GO
GO:0050890	cognition	16/212	286/17913	3.16E-07	4.95E-06	2.57E-06	APOE/BCHE/CAS	16	GO
GO:0043393	regulation of protein binding	14/212	217/17913	3.20E-07	4.99E-06	2.58E-06	ABL1/APOE/DAB	14	GO
GO:0043312	neutrophil degranulation	21/212	485/17913	3.32E-07	5.15E-06	2.67E-06	SERPINA3/RHOA/	21	GO
GO:0051279	regulation of release of sequestered calcium ion into cytosol	9/212	78/17913	3.46E-07	5.35E-06	2.77E-06	ABL1/F2/PTK2B/	9	GO
GO:0045667	regulation of osteoblast differentiation	11/212	128/17913	3.57E-07	5.51E-06	2.85E-06	DDX5/GLI3/IL6ST,	11	GO
GO:0051480	regulation of cytosolic calcium ion concentration	16/212	289/17913	3.64E-07	5.59E-06	2.90E-06	ABL1/CD4/DLG4/	16	GO
GO:0002283	neutrophil activation involved in immune response	21/212	488/17913	3.67E-07	5.61E-06	2.91E-06	SERPINA3/RHOA/	21	GO
GO:0010959	regulation of metal ion transport	18/212	365/17913	3.69E-07	5.62E-06	2.91E-06	ABL1/ACTN2/RH	18	GO

GO:0060401	cytosolic calcium ion transport	12/212	157/17913	3.74E-07	5.68E-06	2.94E-06	ABL1/CD4/F2/PTI	12 GO
GO:0030518	intracellular steroid hormone receptor signaling pathway	11/212	129/17913	3.87E-07	5.84E-06	3.03E-06	PARP1/RHOA/DA	11 GO
GO:0030521	androgen receptor signaling pathway	8/212	58/17913	3.89E-07	5.84E-06	3.03E-06	RHOA/DAB2/DDX	8 GO
GO:1904589	regulation of protein import	8/212	58/17913	3.89E-07	5.84E-06	3.03E-06	CDH1/GLI3/JUP/S	8 GO
GO:0001823	mesonephros development	10/212	103/17913	3.95E-07	5.90E-06	3.05E-06	GLI3/SMAD1/SM/	10 GO
GO:0007204	positive regulation of cytosolic calcium ion concentration	15/212	255/17913	3.95E-07	5.90E-06	3.05E-06	ABL1/CD4/DLG4/	15 GO
GO:0035791	platelet-derived growth factor receptor-beta signaling pathway	5/212	14/17913	4.07E-07	6.04E-06	3.13E-06	ABL1/LRP1/PDGF	5 GO
GO:0051054	positive regulation of DNA metabolic process	14/212	222/17913	4.22E-07	6.22E-06	3.22E-06	PARP1/PTK2B/JU	14 GO
GO:0070302	regulation of stress-activated protein kinase signaling cascade	14/212	222/17913	4.22E-07	6.22E-06	3.22E-06	DUSP3/PTK2B/LY	14 GO
GO:0001101	response to acid chemical	17/212	330/17913	4.29E-07	6.31E-06	3.27E-06	APOB/RHOA/BCH	17 GO
GO:0006869	lipid transport	17/212	332/17913	4.67E-07	6.84E-06	3.54E-06	APOB/APOE/ITGE	17 GO
GO:0009743	response to carbohydrate	14/212	224/17913	4.70E-07	6.84E-06	3.54E-06	APOB/RHOA/CAS	14 GO
GO:0098927	vesicle-mediated transport between endosomal compartments	7/212	41/17913	4.71E-07	6.84E-06	3.54E-06	DAB2/MAPK1/M/	7 GO
GO:0042119	neutrophil activation	21/212	499/17913	5.28E-07	7.65E-06	3.96E-06	SERPINA3/RHOA/	21 GO
GO:0010038	response to metal ion	17/212	335/17913	5.30E-07	7.65E-06	3.96E-06	PARP1/CCND1/C/	17 GO
GO:2000377	regulation of reactive oxygen species metabolic process	13/212	194/17913	5.54E-07	7.97E-06	4.13E-06	RHOA/F2/PTK2B/	13 GO
GO:0051893	regulation of focal adhesion assembly	8/212	61/17913	5.80E-07	8.28E-06	4.29E-06	ABL1/RHOA/DUS	8 GO
GO:0090109	regulation of cell-substrate junction assembly	8/212	61/17913	5.80E-07	8.28E-06	4.29E-06	ABL1/RHOA/DUS	8 GO
GO:0045619	regulation of lymphocyte differentiation	12/212	164/17913	5.99E-07	8.52E-06	4.41E-06	ABL1/RHOA/ERBI	12 GO
GO:0034349	glial cell apoptotic process	5/212	15/17913	6.04E-07	8.54E-06	4.42E-06	CASP3/PRKCA/PR	5 GO
GO:0072075	metanephric mesenchyme development	5/212	15/17913	6.04E-07	8.54E-06	4.42E-06	SMAD4/MYC/PAX	5 GO
GO:0034614	cellular response to reactive oxygen species	12/212	165/17913	6.39E-07	9.00E-06	4.66E-06	ABL1/APEX1/RHC	12 GO
GO:0006913	nucleocytoplasmic transport	16/212	302/17913	6.55E-07	9.20E-06	4.77E-06	CDH1/GLI3/JUP/K	16 GO
GO:0031331	positive regulation of cellular catabolic process	17/212	341/17913	6.78E-07	9.49E-06	4.92E-06	APOE/DAB2/PTK2	17 GO
GO:0097305	response to alcohol	14/212	232/17913	7.18E-07	1.00E-05	5.19E-06	PARP1/RHOA/CC	14 GO
GO:0051169	nuclear transport	16/212	305/17913	7.47E-07	1.04E-05	5.38E-06	CDH1/GLI3/JUP/K	16 GO
GO:0070661	leukocyte proliferation	15/212	270/17913	8.17E-07	1.13E-05	5.86E-06	ABL1/CASP3/CD4	15 GO
GO:0002573	myeloid leukocyte differentiation	13/212	201/17913	8.29E-07	1.14E-05	5.93E-06	PARP1/CD4/EPH/	13 GO
GO:0051403	stress-activated MAPK cascade	15/212	271/17913	8.56E-07	1.18E-05	6.10E-06	CASR/CRKL/DUSF	15 GO
GO:0043062	extracellular structure organization	18/212	387/17913	8.63E-07	1.18E-05	6.13E-06	ABL1/APOB/APOI	18 GO
GO:0097529	myeloid leukocyte migration	12/212	170/17913	8.79E-07	1.19E-05	6.17E-06	PTK2B/ITGB2/KIT	12 GO
GO:0007259	JAK-STAT cascade	11/212	140/17913	8.79E-07	1.19E-05	6.17E-06	F2/IFNAR1/IL6ST/	11 GO
GO:0034250	positive regulation of cellular amide metabolic process	11/212	140/17913	8.79E-07	1.19E-05	6.17E-06	RHOA/CASP3/ERI	11 GO
GO:0072073	kidney epithelium development	11/212	140/17913	8.79E-07	1.19E-05	6.17E-06	GLI3/SMAD1/SM/	11 GO
GO:0030183	B cell differentiation	10/212	113/17913	9.37E-07	1.26E-05	6.55E-06	ABL1/EP300/PTK:	10 GO
GO:0060537	muscle tissue development	18/212	392/17913	1.04E-06	1.39E-05	7.23E-06	ACTN2/RHOA/DD	18 GO
GO:0060562	epithelial tube morphogenesis	16/212	313/17913	1.05E-06	1.40E-05	7.27E-06	ABL1/RHOA/RHO	16 GO
GO:1901214	regulation of neuron death	16/212	313/17913	1.05E-06	1.40E-05	7.27E-06	ABL1/PARP1/APC	16 GO
GO:0060324	face development	7/212	46/17913	1.07E-06	1.42E-05	7.35E-06	EP300/PDGFRA/N	7 GO
GO:0010517	regulation of phospholipase activity	8/212	66/17913	1.07E-06	1.43E-05	7.39E-06	ABL1/KIT/LRP1/P	8 GO
GO:1902107	positive regulation of leukocyte differentiation	11/212	143/17913	1.09E-06	1.44E-05	7.44E-06	RHOA/CD4/GLI3/	11 GO
GO:0032103	positive regulation of response to external stimulus	16/212	315/17913	1.14E-06	1.50E-05	7.78E-06	CASR/F2/PTK2B/I	16 GO
GO:0032535	regulation of cellular component size	16/212	315/17913	1.14E-06	1.50E-05	7.78E-06	ABL1/ACTN2/APC	16 GO
GO:0042542	response to hydrogen peroxide	11/212	144/17913	1.16E-06	1.52E-05	7.87E-06	ABL1/APEX1/RHC	11 GO
GO:1903169	regulation of calcium ion transmembrane transport	11/212	144/17913	1.16E-06	1.52E-05	7.87E-06	ABL1/F2/PTK2B/L	11 GO
GO:1903391	regulation of adherens junction organization	8/212	67/17913	1.21E-06	1.57E-05	8.15E-06	ABL1/RHOA/DUS	8 GO
GO:2000641	regulation of early endosome to late endosome transport	5/212	17/17913	1.22E-06	1.58E-05	8.21E-06	DAB2/MAPK1/M/	5 GO
GO:0052547	regulation of peptidase activity	18/212	398/17913	1.29E-06	1.67E-05	8.64E-06	RHOA/CD44/HDA	18 GO
GO:0014909	smooth muscle cell migration	9/212	91/17913	1.30E-06	1.68E-05	8.69E-06	APEX1/ITGB3/LRI	9 GO

GO:0048754	branching morphogenesis of an epithelial tube	11/212	146/17913	1.33E-06	1.71E-05	8.88E-06	ABL1/CASR/EPHA	11	GO
GO:0090066	regulation of anatomical structure size	19/212	442/17913	1.38E-06	1.77E-05	9.16E-06	ABL1/ACTN2/APC	19	GO
GO:0061138	morphogenesis of a branching epithelium	12/212	178/17913	1.43E-06	1.83E-05	9.47E-06	ABL1/CASR/EPHA	12	GO
GO:0051052	regulation of DNA metabolic process	18/212	403/17913	1.54E-06	1.96E-05	1.02E-05	PARP1/PTK2B/JU	18	GO
GO:1901216	positive regulation of neuron death	9/212	93/17913	1.56E-06	1.99E-05	1.03E-05	ABL1/PARP1/RHO	9	GO
GO:1905477	positive regulation of protein localization to membrane	10/212	120/17913	1.63E-06	2.06E-05	1.07E-05	EPHA2/ERBB2/IT	10	GO
GO:0001655	urogenital system development	16/212	324/17913	1.65E-06	2.08E-05	1.08E-05	CRKL/GLI3/SMAD	16	GO
GO:0019058	viral life cycle	16/212	324/17913	1.65E-06	2.08E-05	1.08E-05	APOE/CD4/DDX5/	16	GO
GO:0003012	muscle system process	19/212	448/17913	1.68E-06	2.11E-05	1.09E-05	ACTN2/PARP1/RH	19	GO
GO:0007015	actin filament organization	16/212	325/17913	1.72E-06	2.15E-05	1.11E-05	ABL1/ACTN2/RHO	16	GO
GO:0097696	STAT cascade	11/212	150/17913	1.74E-06	2.17E-05	1.12E-05	F2/IFNAR1/IL6ST/	11	GO
GO:0045637	regulation of myeloid cell differentiation	14/212	250/17913	1.75E-06	2.17E-05	1.12E-05	CD4/EP300/PTK2	14	GO
GO:0090101	negative regulation of transmembrane receptor protein serine/threonine kinase signaling pathway	10/212	121/17913	1.76E-06	2.18E-05	1.13E-05	ABL1/SMAD2/SM	10	GO
GO:0010876	lipid localization	17/212	366/17913	1.79E-06	2.21E-05	1.15E-05	APOB/APOE/ITGE	17	GO
GO:0052548	regulation of endopeptidase activity	17/212	370/17913	2.08E-06	2.55E-05	1.32E-05	RHOA/CD44/HDA	17	GO
GO:0050769	positive regulation of neurogenesis	19/212	455/17913	2.11E-06	2.59E-05	1.34E-05	APOE/RHOA/CRK	19	GO
GO:0001935	endothelial cell proliferation	12/212	185/17913	2.15E-06	2.62E-05	1.36E-05	APOE/EPHA2/ITG	12	GO
GO:1902905	positive regulation of supramolecular fiber organization	12/212	185/17913	2.15E-06	2.62E-05	1.36E-05	ABL1/ACTN2/APC	12	GO
GO:0097242	amyloid-beta clearance	6/212	33/17913	2.17E-06	2.64E-05	1.37E-05	APOE/HDAC1/ITG	6	GO
GO:0060249	anatomical structure homeostasis	18/212	413/17913	2.18E-06	2.64E-05	1.37E-05	SERPINA3/PARP1	18	GO
GO:0072074	kidney mesenchyme development	5/212	19/17913	2.25E-06	2.72E-05	1.41E-05	SMAD4/MYC/PAX	5	GO
GO:0032872	regulation of stress-activated MAPK cascade	13/212	220/17913	2.28E-06	2.75E-05	1.42E-05	DUSP3/PTK2B/NE	13	GO
GO:0006816	calcium ion transport	18/212	415/17913	2.33E-06	2.80E-05	1.45E-05	ABL1/RHOA/CASI	18	GO
GO:0045862	positive regulation of proteolysis	16/212	333/17913	2.36E-06	2.82E-05	1.46E-05	APOE/RHOA/DAE	16	GO
GO:0007043	cell-cell junction assembly	9/212	98/17913	2.43E-06	2.91E-05	1.51E-05	APC/RHOA/RHOC	9	GO
GO:0097553	calcium ion transmembrane import into cytosol	10/212	126/17913	2.55E-06	3.03E-05	1.57E-05	ABL1/F2/PTK2B/L	10	GO
GO:0090288	negative regulation of cellular response to growth factor stimulus	11/212	156/17913	2.56E-06	3.04E-05	1.58E-05	ABL1/SMAD2/SM	11	GO
GO:0014706	striated muscle tissue development	17/212	376/17913	2.58E-06	3.06E-05	1.58E-05	ACTN2/RHOA/DD	17	GO
GO:0045216	cell-cell junction organization	10/212	128/17913	2.94E-06	3.47E-05	1.80E-05	APC/RHOA/RHOC	10	GO
GO:0030878	thyroid gland development	5/212	20/17913	2.97E-06	3.50E-05	1.81E-05	SMAD3/MAPK1/I	5	GO
GO:1903532	positive regulation of secretion by cell	17/212	381/17913	3.08E-06	3.62E-05	1.87E-05	ABL1/CASR/FGR/	17	GO
GO:0035335	peptidyl-tyrosine dephosphorylation	9/212	101/17913	3.13E-06	3.66E-05	1.90E-05	DUSP3/PTPN1/PT	9	GO
GO:2000379	positive regulation of reactive oxygen species metabolic process	9/212	101/17913	3.13E-06	3.66E-05	1.90E-05	F2/PTK2B/ITGB2/	9	GO
GO:0010595	positive regulation of endothelial cell migration	10/212	129/17913	3.15E-06	3.66E-05	1.90E-05	ABL1/RHOB/PTK2	10	GO
GO:1903364	positive regulation of cellular protein catabolic process	10/212	129/17913	3.15E-06	3.66E-05	1.90E-05	APOE/DAB2/PTK2	10	GO
GO:0001763	morphogenesis of a branching structure	12/212	192/17913	3.16E-06	3.66E-05	1.90E-05	ABL1/CASR/EPHA	12	GO
GO:0010611	regulation of cardiac muscle hypertrophy	8/212	76/17913	3.19E-06	3.68E-05	1.91E-05	PARP1/IL6ST/SM/	8	GO
GO:0001701	in utero embryonic development	16/212	342/17913	3.32E-06	3.82E-05	1.98E-05	APOB/GLI3/SMAI	16	GO
GO:0031099	regeneration	12/212	194/17913	3.52E-06	4.04E-05	2.09E-05	CCND1/GLI3/JUN	12	GO
GO:0014812	muscle cell migration	9/212	103/17913	3.69E-06	4.22E-05	2.19E-05	APEX1/ITGB3/LRI	9	GO
GO:0010742	macrophage derived foam cell differentiation	6/212	36/17913	3.71E-06	4.22E-05	2.19E-05	APOB/EP300/ITG	6	GO
GO:0090077	foam cell differentiation	6/212	36/17913	3.71E-06	4.22E-05	2.19E-05	APOB/EP300/ITG	6	GO
GO:1903362	regulation of cellular protein catabolic process	13/212	230/17913	3.72E-06	4.23E-05	2.19E-05	APOE/ARAF/DAB	13	GO
GO:0022604	regulation of cell morphogenesis	18/212	430/17913	3.83E-06	4.34E-05	2.25E-05	ABL1/APOE/RHO/	18	GO
GO:0060396	growth hormone receptor signaling pathway	5/212	21/17913	3.86E-06	4.36E-05	2.26E-05	LYN/PIK3R1/PRLF	5	GO
GO:0033143	regulation of intracellular steroid hormone receptor signaling pathway	8/212	78/17913	3.89E-06	4.38E-05	2.27E-05	PARP1/RHOA/DA	8	GO
GO:0007548	sex differentiation	14/212	269/17913	4.11E-06	4.60E-05	2.38E-05	CCND1/CASP3/CE	14	GO
GO:0072593	reactive oxygen species metabolic process	14/212	269/17913	4.11E-06	4.60E-05	2.38E-05	RHOA/F2/PTK2B/	14	GO
GO:0014743	regulation of muscle hypertrophy	8/212	79/17913	4.28E-06	4.78E-05	2.48E-05	PARP1/IL6ST/SM/	8	GO

GO:0030260	entry into host cell	10/212	134/17913	4.44E-06	4.90E-05	2.54E-05	CBL/CD4/CDH1/C	10	GO
GO:0044409	entry into host	10/212	134/17913	4.44E-06	4.90E-05	2.54E-05	CBL/CD4/CDH1/C	10	GO
GO:0051806	entry into cell of other organism involved in symbiotic interaction	10/212	134/17913	4.44E-06	4.90E-05	2.54E-05	CBL/CD4/CDH1/C	10	GO
GO:0051828	entry into other organism involved in symbiotic interaction	10/212	134/17913	4.44E-06	4.90E-05	2.54E-05	CBL/CD4/CDH1/C	10	GO
GO:0001501	skeletal system development	19/212	479/17913	4.46E-06	4.90E-05	2.54E-05	RHOA/CD44/EPH.	19	GO
GO:0050727	regulation of inflammatory response	19/212	479/17913	4.46E-06	4.90E-05	2.54E-05	APOE/CD59/CDH.	19	GO
GO:0009314	response to radiation	18/212	435/17913	4.50E-06	4.93E-05	2.55E-05	PARP1/RHOB/CCI	18	GO
GO:0060390	regulation of SMAD protein signal transduction	5/212	22/17913	4.95E-06	5.40E-05	2.80E-05	PARP1/DAB2/SM	5	GO
GO:0071378	cellular response to growth hormone stimulus	5/212	22/17913	4.95E-06	5.40E-05	2.80E-05	LYN/PIK3R1/PRLF	5	GO
GO:0007623	circadian rhythm	12/212	201/17913	5.07E-06	5.51E-05	2.86E-05	EP300/HDAC1/JU	12	GO
GO:0042176	regulation of protein catabolic process	16/212	354/17913	5.15E-06	5.59E-05	2.89E-05	APC/APOE/ARAF/	16	GO
GO:0051402	neuron apoptotic process	13/212	238/17913	5.41E-06	5.85E-05	3.03E-05	PARP1/RHOA/CA	13	GO
GO:0001822	kidney development	14/212	276/17913	5.52E-06	5.96E-05	3.09E-05	GLI3/SMAD1/SM.	14	GO
GO:0051056	regulation of small GTPase mediated signal transduction	15/212	316/17913	5.69E-06	6.12E-05	3.17E-05	ABL1/APOE/RHO.	15	GO
GO:0050728	negative regulation of inflammatory response	11/212	170/17913	5.88E-06	6.31E-05	3.27E-05	APOE/CDH5/F2/L	11	GO
GO:0045580	regulation of T cell differentiation	10/212	139/17913	6.16E-06	6.59E-05	3.42E-05	ABL1/RHOA/ERBI	10	GO
GO:0071216	cellular response to biotic stimulus	12/212	206/17913	6.52E-06	6.96E-05	3.61E-05	ABL1/RHOA/LYN/	12	GO
GO:0007163	establishment or maintenance of cell polarity	11/212	172/17913	6.57E-06	7.00E-05	3.63E-05	RHOA/CDH5/CRK	11	GO
GO:0051656	establishment of organelle localization	18/212	448/17913	6.74E-06	7.16E-05	3.71E-05	CBL/CD59/DCTN1	18	GO
GO:1902893	regulation of pri-miRNA transcription by RNA polymerase II	6/212	40/17913	7.02E-06	7.44E-05	3.86E-05	JUN/SMAD1/SM/	6	GO
GO:0046651	lymphocyte proliferation	13/212	244/17913	7.09E-06	7.49E-05	3.88E-05	ABL1/CASP3/CD4	13	GO
GO:0051209	release of sequestered calcium ion into cytosol	9/212	112/17913	7.36E-06	7.74E-05	4.01E-05	ABL1/F2/PTK2B/L	9	GO
GO:0051283	negative regulation of sequestering of calcium ion	9/212	112/17913	7.36E-06	7.74E-05	4.01E-05	ABL1/F2/PTK2B/L	9	GO
GO:0046427	positive regulation of JAK-STAT cascade	8/212	85/17913	7.43E-06	7.79E-05	4.04E-05	F2/PTK2B/IL6ST/I	8	GO
GO:0010256	endomembrane system organization	16/212	365/17913	7.58E-06	7.92E-05	4.11E-05	DCTN1/PLK1/SRG	16	GO
GO:0045861	negative regulation of proteolysis	15/212	324/17913	7.68E-06	8.01E-05	4.15E-05	CD44/CD59/F2/P	15	GO
GO:0032943	mononuclear cell proliferation	13/212	246/17913	7.74E-06	8.05E-05	4.17E-05	ABL1/CASP3/CD4	13	GO
GO:0016055	Wnt signaling pathway	19/212	498/17913	7.77E-06	8.07E-05	4.18E-05	ABL1/APC/APOE/	19	GO
GO:1903649	regulation of cytoplasmic transport	5/212	24/17913	7.84E-06	8.11E-05	4.20E-05	DAB2/MAPK1/M/	5	GO
GO:0051047	positive regulation of secretion	17/212	409/17913	7.88E-06	8.14E-05	4.22E-05	ABL1/CASR/FGR/	17	GO
GO:0070838	divalent metal ion transport	18/212	454/17913	8.09E-06	8.33E-05	4.32E-05	ABL1/RHOA/CASI	18	GO
GO:0198738	cell-cell signaling by wnt	19/212	500/17913	8.23E-06	8.45E-05	4.38E-05	ABL1/APC/APOE/	19	GO
GO:0010212	response to ionizing radiation	10/212	144/17913	8.43E-06	8.64E-05	4.48E-05	PARP1/RHOB/CCI	10	GO
GO:0051282	regulation of sequestering of calcium ion	9/212	114/17913	8.51E-06	8.70E-05	4.51E-05	ABL1/F2/PTK2B/L	9	GO
GO:0042098	T cell proliferation	11/212	177/17913	8.64E-06	8.79E-05	4.55E-05	ABL1/CASP3/CD4	11	GO
GO:0043112	receptor metabolic process	11/212	177/17913	8.64E-06	8.79E-05	4.55E-05	APOE/DLG4/HDA	11	GO
GO:0050678	regulation of epithelial cell proliferation	16/212	369/17913	8.68E-06	8.81E-05	4.57E-05	APOE/CCND1/ERI	16	GO
GO:0048568	embryonic organ development	17/212	413/17913	8.95E-06	9.06E-05	4.69E-05	EPHA2/GLI3/KIT/!	17	GO
GO:0016311	dephosphorylation	18/212	458/17913	9.11E-06	9.18E-05	4.76E-05	APEX1/DUSP3/IN	18	GO
GO:0072511	divalent inorganic cation transport	18/212	458/17913	9.11E-06	9.18E-05	4.76E-05	ABL1/RHOA/CASI	18	GO
GO:0090257	regulation of muscle system process	13/212	251/17913	9.61E-06	9.65E-05	5.00E-05	PARP1/RHOA/IL6	13	GO
GO:1904894	positive regulation of STAT cascade	8/212	88/17913	9.63E-06	9.65E-05	5.00E-05	F2/PTK2B/IL6ST/I	8	GO
GO:0045787	positive regulation of cell cycle	16/212	373/17913	9.93E-06	9.93E-05	5.15E-05	ABL1/APEX1/RHC	16	GO
GO:0072001	renal system development	14/212	291/17913	1.01E-05	1.01E-04	5.21E-05	GLI3/SMAD1/SM.	14	GO
GO:0071236	cellular response to antibiotic	10/212	147/17913	1.01E-05	1.01E-04	5.22E-05	ABL1/APEX1/RHC	10	GO
GO:0045727	positive regulation of translation	9/212	117/17913	1.05E-05	1.04E-04	5.40E-05	RHOA/ERBB2/PTI	9	GO
GO:0051208	sequestering of calcium ion	9/212	117/17913	1.05E-05	1.04E-04	5.40E-05	ABL1/F2/PTK2B/L	9	GO
GO:0071364	cellular response to epidermal growth factor stimulus	6/212	43/17913	1.08E-05	1.07E-04	5.54E-05	CBL/DUSP3/ERBE	6	GO
GO:0008406	gonad development	12/212	217/17913	1.10E-05	1.09E-04	5.64E-05	CCND1/CASP3/CE	12	GO

GO:0043534	blood vessel endothelial cell migration	11/212	182/17913	1.12E-05	1.10E-04	5.71E-05	ABL1/APOE/RHO/	11	GO
GO:1903708	positive regulation of hemopoiesis	11/212	182/17913	1.12E-05	1.10E-04	5.71E-05	RHOA/CD4/GLI3/	11	GO
GO:1904888	cranial skeletal system development	7/212	65/17913	1.15E-05	1.12E-04	5.81E-05	CRKL/GLI3/SMAD	7	GO
GO:0016049	cell growth	18/212	466/17913	1.15E-05	1.12E-04	5.83E-05	ABL1/APOE/RHO/	18	GO
GO:0016485	protein processing	14/212	295/17913	1.18E-05	1.14E-04	5.92E-05	PARP1/CASP3/CD	14	GO
GO:0032496	response to lipopolysaccharide	14/212	295/17913	1.18E-05	1.14E-04	5.92E-05	ABL1/APOB/RHO	14	GO
GO:0050714	positive regulation of protein secretion	13/212	256/17913	1.19E-05	1.15E-04	5.96E-05	ABL1/CASR/FGR/	13	GO
GO:0034599	cellular response to oxidative stress	14/212	296/17913	1.22E-05	1.18E-04	6.12E-05	ABL1/PARP1/APE	14	GO
GO:0030225	macrophage differentiation	6/212	44/17913	1.24E-05	1.20E-04	6.19E-05	PARP1/CD4/PRK(	6	GO
GO:0035196	production of miRNAs involved in gene silencing by miRNA	6/212	44/17913	1.24E-05	1.20E-04	6.19E-05	DDX5/SMAD1/SN	6	GO
GO:0048308	organelle inheritance	4/212	13/17913	1.25E-05	1.20E-04	6.21E-05	MAPK1/MAPK3/N	4	GO
GO:0048313	Golgi inheritance	4/212	13/17913	1.25E-05	1.20E-04	6.21E-05	MAPK1/MAPK3/N	4	GO
GO:0051917	regulation of fibrinolysis	4/212	13/17913	1.25E-05	1.20E-04	6.21E-05	F2/SERPINE1/PLG	4	GO
GO:1905952	regulation of lipid localization	10/212	151/17913	1.28E-05	1.22E-04	6.33E-05	APOB/APOE/ITGE	10	GO
GO:0060485	mesenchyme development	13/212	258/17913	1.29E-05	1.23E-04	6.35E-05	DAB2/DDX5/SMA	13	GO
GO:0008630	intrinsic apoptotic signaling pathway in response to DNA damage	8/212	92/17913	1.34E-05	1.27E-04	6.58E-05	ABL1/CD44/EPHA	8	GO
GO:0048871	multicellular organismal homeostasis	18/212	472/17913	1.37E-05	1.30E-04	6.71E-05	SERPINA3/APC/P	18	GO
GO:0050920	regulation of chemotaxis	11/212	186/17913	1.38E-05	1.30E-04	6.75E-05	CASR/DUSP3/PTK	11	GO
GO:0043500	muscle adaptation	9/212	121/17913	1.38E-05	1.30E-04	6.75E-05	PARP1/IL6ST/SM/	9	GO
GO:0001649	osteoblast differentiation	12/212	222/17913	1.39E-05	1.31E-04	6.77E-05	DDX5/EPHA2/GLI	12	GO
GO:0016579	protein deubiquitination	13/212	260/17913	1.40E-05	1.31E-04	6.80E-05	APC/RHOA/EP30(	13	GO
GO:0010812	negative regulation of cell-substrate adhesion	7/212	67/17913	1.40E-05	1.31E-04	6.81E-05	RHOA/LRP1/SERF	7	GO
GO:0030111	regulation of Wnt signaling pathway	15/212	341/17913	1.41E-05	1.32E-04	6.82E-05	ABL1/APC/APOE/	15	GO
GO:0045137	development of primary sexual characteristics	12/212	223/17913	1.45E-05	1.35E-04	7.01E-05	CCND1/CASP3/CE	12	GO
GO:0051090	regulation of DNA-binding transcription factor activity	17/212	429/17913	1.46E-05	1.36E-04	7.05E-05	EP300/ITGB2/JUN	17	GO
GO:0006898	receptor-mediated endocytosis	14/212	301/17913	1.48E-05	1.36E-04	7.06E-05	APOB/APOE/CBL/	14	GO
GO:0032368	regulation of lipid transport	9/212	122/17913	1.48E-05	1.36E-04	7.06E-05	APOE/ITGB3/LRP	9	GO
GO:0035270	endocrine system development	9/212	122/17913	1.48E-05	1.36E-04	7.06E-05	CDH1/CRKL/SMA	9	GO
GO:0030595	leukocyte chemotaxis	11/212	188/17913	1.53E-05	1.41E-04	7.28E-05	PTK2B/ITGB2/KIT	11	GO
GO:0007030	Golgi organization	8/212	94/17913	1.57E-05	1.44E-04	7.47E-05	MAPK1/MAPK3/N	8	GO
GO:0048538	thymus development	6/212	46/17913	1.62E-05	1.48E-04	7.68E-05	ABL1/CRKL/MAPI	6	GO
GO:0009749	response to glucose	11/212	190/17913	1.68E-05	1.54E-04	7.99E-05	RHOA/CASP3/CA:	11	GO
GO:0098542	defense response to other organism	18/212	481/17913	1.76E-05	1.61E-04	8.33E-05	KLK3/CD4/EPHA2	18	GO
GO:0110053	regulation of actin filament organization	12/212	228/17913	1.81E-05	1.65E-04	8.56E-05	ABL1/ACTN2/RH(	12	GO
GO:0042116	macrophage activation	8/212	96/17913	1.83E-05	1.67E-04	8.63E-05	ITGB2/JUN/LDLR/	8	GO
GO:0045732	positive regulation of protein catabolic process	11/212	192/17913	1.86E-05	1.68E-04	8.73E-05	APC/APOE/DAB2,	11	GO
GO:0043535	regulation of blood vessel endothelial cell migration	10/212	158/17913	1.90E-05	1.72E-04	8.92E-05	ABL1/APOE/RHO/	10	GO
GO:0002237	response to molecule of bacterial origin	14/212	309/17913	1.98E-05	1.78E-04	9.21E-05	ABL1/APOB/RHO	14	GO
GO:0030038	contractile actin filament bundle assembly	8/212	97/17913	1.98E-05	1.78E-04	9.21E-05	ABL1/RHOA/RHO	8	GO
GO:0043149	stress fiber assembly	8/212	97/17913	1.98E-05	1.78E-04	9.21E-05	ABL1/RHOA/RHO	8	GO
GO:0045471	response to ethanol	9/212	127/17913	2.04E-05	1.83E-04	9.48E-05	RHOA/CCND1/CB	9	GO
GO:0071214	cellular response to abiotic stimulus	14/212	310/17913	2.05E-05	1.83E-04	9.48E-05	PARP1/RHOB/CA:	14	GO
GO:0104004	cellular response to environmental stimulus	14/212	310/17913	2.05E-05	1.83E-04	9.48E-05	PARP1/RHOB/CA:	14	GO
GO:0001707	mesoderm formation	7/212	71/17913	2.06E-05	1.83E-04	9.50E-05	EPHA2/ITGB3/SN	7	GO
GO:0043407	negative regulation of MAP kinase activity	7/212	71/17913	2.06E-05	1.83E-04	9.50E-05	APOE/DUSP3/LYN	7	GO
GO:0031050	dsRNA processing	6/212	48/17913	2.08E-05	1.84E-04	9.51E-05	DDX5/SMAD1/SN	6	GO
GO:0070918	production of small RNA involved in gene silencing by RNA	6/212	48/17913	2.08E-05	1.84E-04	9.51E-05	DDX5/SMAD1/SN	6	GO
GO:0001558	regulation of cell growth	16/212	396/17913	2.08E-05	1.84E-04	9.51E-05	ABL1/APOE/RHO/	16	GO
GO:0046627	negative regulation of insulin receptor signaling pathway	5/212	29/17913	2.09E-05	1.84E-04	9.51E-05	GRB10/PRKCB/PF	5	GO

GO:0070102	interleukin-6-mediated signaling pathway	5/212	29/17913	2.09E-05	1.84E-04	9.51E-05	CBL/IL6ST/SMAD4	5 GO
GO:0051604	protein maturation	15/212	353/17913	2.11E-05	1.86E-04	9.62E-05	PARP1/CASP3/CD	15 GO
GO:0046661	male sex differentiation	10/212	160/17913	2.12E-05	1.86E-04	9.63E-05	CCND1/CBL/CRKL	10 GO
GO:0050848	regulation of calcium-mediated signaling	8/212	98/17913	2.13E-05	1.86E-04	9.65E-05	CD4/PTK2B/PKD2	8 GO
GO:0009746	response to hexose	11/212	195/17913	2.15E-05	1.87E-04	9.70E-05	RHOA/CASP3/CAS	11 GO
GO:0006470	protein dephosphorylation	14/212	313/17913	2.28E-05	1.98E-04	1.03E-04	DUSP3/PDGFRB/I	14 GO
GO:0070646	protein modification by small protein removal	13/212	273/17913	2.34E-05	2.02E-04	1.05E-04	APC/RHOA/EP300	13 GO
GO:0043113	receptor clustering	6/212	49/17913	2.34E-05	2.02E-04	1.05E-04	APOE/CRKL/DLG4	6 GO
GO:0035635	entry of bacterium into host cell	4/212	15/17913	2.35E-05	2.02E-04	1.05E-04	CBL/CDH1/CTNNI	4 GO
GO:0048012	hepatocyte growth factor receptor signaling pathway	4/212	15/17913	2.35E-05	2.02E-04	1.05E-04	MST1R/PAK1/PTF	4 GO
GO:0090136	epithelial cell-cell adhesion	4/212	15/17913	2.35E-05	2.02E-04	1.05E-04	JUP/KIT/CCN3/VC	4 GO
GO:0001819	positive regulation of cytokine production	17/212	446/17913	2.41E-05	2.07E-04	1.07E-04	ABL1/CD4/EP300/	17 GO
GO:0048259	regulation of receptor-mediated endocytosis	8/212	100/17913	2.47E-05	2.11E-04	1.09E-04	CBL/DAB2/DLG4/	8 GO
GO:1903050	regulation of proteolysis involved in cellular protein catabolic process	11/212	198/17913	2.47E-05	2.11E-04	1.09E-04	APOE/ARAF/DAB	11 GO
GO:0048332	mesoderm morphogenesis	7/212	73/17913	2.48E-05	2.11E-04	1.09E-04	EPHA2/ITGB3/SM	7 GO
GO:0010743	regulation of macrophage derived foam cell differentiation	5/212	30/17913	2.48E-05	2.11E-04	1.09E-04	APOB/ITGB3/PLA	5 GO
GO:1902895	positive regulation of pri-miRNA transcription by RNA polymerase II	5/212	30/17913	2.48E-05	2.11E-04	1.09E-04	JUN/SMAD1/SMA	5 GO
GO:0002695	negative regulation of leukocyte activation	10/212	163/17913	2.49E-05	2.11E-04	1.09E-04	CASP3/DUSP3/ER	10 GO
GO:0002793	positive regulation of peptide secretion	13/212	275/17913	2.53E-05	2.14E-04	1.11E-04	ABL1/CASR/FGR/	13 GO
GO:0042692	muscle cell differentiation	15/212	359/17913	2.57E-05	2.17E-04	1.13E-04	ABL1/ACTN2/CAS	15 GO
GO:1990778	protein localization to cell periphery	13/212	276/17913	2.62E-05	2.21E-04	1.15E-04	ACTN2/CDH1/DA	13 GO
GO:0018107	peptidyl-threonine phosphorylation	8/212	101/17913	2.65E-05	2.23E-04	1.15E-04	SMAD7/PLK1/PRI	8 GO
GO:0031348	negative regulation of defense response	12/212	237/17913	2.66E-05	2.23E-04	1.15E-04	APOE/CDH5/F2/L	12 GO
GO:0048863	stem cell differentiation	12/212	237/17913	2.66E-05	2.23E-04	1.15E-04	ABL1/BCHE/KIT/S	12 GO
GO:0060291	long-term synaptic potentiation	7/212	74/17913	2.71E-05	2.26E-04	1.17E-04	ABL1/APOE/PTK2	7 GO
GO:0009612	response to mechanical stimulus	11/212	200/17913	2.71E-05	2.26E-04	1.17E-04	RHOA/PTK2B/JUN	11 GO
GO:0034284	response to monosaccharide	11/212	200/17913	2.71E-05	2.26E-04	1.17E-04	RHOA/CASP3/CAS	11 GO
GO:0002683	negative regulation of immune system process	17/212	451/17913	2.77E-05	2.30E-04	1.19E-04	CASP3/CD59/DUS	17 GO
GO:0045165	cell fate commitment	12/212	238/17913	2.77E-05	2.30E-04	1.19E-04	RHOA/CASP3/GLI	12 GO
GO:0031346	positive regulation of cell projection organization	15/212	362/17913	2.83E-05	2.34E-04	1.21E-04	APC/APOE/RHOA	15 GO
GO:2001020	regulation of response to DNA damage stimulus	11/212	201/17913	2.84E-05	2.35E-04	1.22E-04	ABL1/PARP1/CD4	11 GO
GO:1900077	negative regulation of cellular response to insulin stimulus	5/212	31/17913	2.93E-05	2.42E-04	1.25E-04	GRB10/PRKCB/PF	5 GO
GO:0071887	leukocyte apoptotic process	8/212	103/17913	3.06E-05	2.52E-04	1.31E-04	CASP3/CRKL/GLI3	8 GO
GO:0071248	cellular response to metal ion	10/212	167/17913	3.07E-05	2.52E-04	1.31E-04	PARP1/CDH1/DLC	10 GO
GO:0030397	membrane disassembly	4/212	16/17913	3.10E-05	2.54E-04	1.32E-04	DCTN1/PLK1/PRK	4 GO
GO:0051081	nuclear envelope disassembly	4/212	16/17913	3.10E-05	2.54E-04	1.32E-04	DCTN1/PLK1/PRK	4 GO
GO:0050792	regulation of viral process	11/212	204/17913	3.25E-05	2.66E-04	1.38E-04	APOE/CD4/DDX5/	11 GO
GO:0010975	regulation of neuron projection development	17/212	458/17913	3.36E-05	2.74E-04	1.42E-04	ABL1/APOE/RHOA	17 GO
GO:0048167	regulation of synaptic plasticity	10/212	169/17913	3.40E-05	2.76E-04	1.43E-04	ABL1/APOE/DLG4	10 GO
GO:0097421	liver regeneration	5/212	32/17913	3.44E-05	2.79E-04	1.45E-04	CCND1/GLI3/PCN	5 GO
GO:0051851	modification by host of symbiont morphology or physiology	7/212	77/17913	3.51E-05	2.85E-04	1.47E-04	APOE/EP300/F2/I	7 GO
GO:0001936	regulation of endothelial cell proliferation	10/212	170/17913	3.57E-05	2.89E-04	1.50E-04	APOE/ITGB3/JUN	10 GO
GO:0008584	male gonad development	9/212	137/17913	3.72E-05	3.00E-04	1.55E-04	CCND1/CBL/CRKL	9 GO
GO:0015718	monocarboxylic acid transport	9/212	137/17913	3.72E-05	3.00E-04	1.55E-04	APOE/CASR/PLA2	9 GO
GO:0043502	regulation of muscle adaptation	8/212	106/17913	3.77E-05	3.03E-04	1.57E-04	PARP1/IL6ST/SMA	8 GO
GO:0010827	regulation of glucose transmembrane transport	7/212	78/17913	3.82E-05	3.06E-04	1.58E-04	GRB10/PIK3R1/PI	7 GO
GO:0030512	negative regulation of transforming growth factor beta receptor signaling pathway	7/212	78/17913	3.82E-05	3.06E-04	1.58E-04	SMAD2/SMAD3/S	7 GO
GO:0046546	development of primary male sexual characteristics	9/212	138/17913	3.94E-05	3.15E-04	1.63E-04	CCND1/CBL/CRKL	9 GO
GO:0045089	positive regulation of innate immune response	15/212	373/17913	3.99E-05	3.18E-04	1.65E-04	APOB/EP300/ITG	15 GO

GO:0007616	long-term memory	5/212	33/17913	4.01E-05	3.19E-04	1.65E-04	APOE/LDLR/PRKC	5 GO
GO:0055094	response to lipoprotein particle	5/212	33/17913	4.01E-05	3.19E-04	1.65E-04	APOE/CASR/ITGB	5 GO
GO:0035855	megakaryocyte development	4/212	17/17913	4.02E-05	3.19E-04	1.65E-04	EP300/KIT/PTPN6	4 GO
GO:1904659	glucose transmembrane transport	8/212	107/17913	4.03E-05	3.19E-04	1.65E-04	GRB10/PIK3R1/PI	8 GO
GO:0010524	positive regulation of calcium ion transport into cytosol	6/212	54/17913	4.12E-05	3.25E-04	1.69E-04	ABL1/CD4/F2/PKI	6 GO
GO:0097581	lamellipodium organization	7/212	79/17913	4.15E-05	3.27E-04	1.69E-04	CD44/EPHA2/KIT/	7 GO
GO:0003300	cardiac muscle hypertrophy	8/212	108/17913	4.31E-05	3.39E-04	1.76E-04	PARP1/IL6ST/SMA	8 GO
GO:0016482	cytosolic transport	9/212	140/17913	4.41E-05	3.47E-04	1.80E-04	DAB2/DCTN1/MA	9 GO
GO:1903845	negative regulation of cellular response to transforming growth factor beta stimulus	7/212	80/17913	4.50E-05	3.53E-04	1.83E-04	SMAD2/SMAD3/S	7 GO
GO:0018210	peptidyl-threonine modification	8/212	109/17913	4.60E-05	3.60E-04	1.87E-04	SMAD7/PLK1/PRI	8 GO
GO:0030198	extracellular matrix organization	14/212	334/17913	4.64E-05	3.62E-04	1.88E-04	ABL1/CD44/CDH1	14 GO
GO:0071222	cellular response to lipopolysaccharide	10/212	176/17913	4.80E-05	3.74E-04	1.94E-04	ABL1/RHOA/LYN/	10 GO
GO:0046849	bone remodeling	7/212	81/17913	4.88E-05	3.79E-04	1.96E-04	EPHA2/PTK2B/IN	7 GO
GO:0051702	interaction with symbiont	7/212	81/17913	4.88E-05	3.79E-04	1.96E-04	APOE/EP300/F2/I	7 GO
GO:0070663	regulation of leukocyte proliferation	11/212	214/17913	5.04E-05	3.90E-04	2.02E-04	CASP3/CD4/ERBB	11 GO
GO:0038083	peptidyl-tyrosine autophosphorylation	4/212	18/17913	5.12E-05	3.96E-04	2.05E-04	ABL1/PTK2B/MAI	4 GO
GO:0090330	regulation of platelet aggregation	4/212	18/17913	5.12E-05	3.96E-04	2.05E-04	LYN/PRKCA/PRKC	4 GO
GO:0043010	camera-type eye development	13/212	295/17913	5.22E-05	4.02E-04	2.08E-04	EPHA2/GLI3/HDA	13 GO
GO:0014897	striated muscle hypertrophy	8/212	111/17913	5.24E-05	4.03E-04	2.09E-04	PARP1/IL6ST/SMA	8 GO
GO:0007369	gastrulation	10/212	178/17913	5.28E-05	4.05E-04	2.10E-04	EPHA2/ITGB2/ITG	10 GO
GO:2000060	positive regulation of ubiquitin-dependent protein catabolic process	7/212	82/17913	5.28E-05	4.05E-04	2.10E-04	DAB2/PTK2B/SMA	7 GO
GO:0007528	neuromuscular junction development	5/212	35/17913	5.38E-05	4.10E-04	2.13E-04	CRKL/DCTN1/ERB	5 GO
GO:0060416	response to growth hormone	5/212	35/17913	5.38E-05	4.10E-04	2.13E-04	LYN/PIK3R1/PRLR	5 GO
GO:0071402	cellular response to lipoprotein particle stimulus	5/212	35/17913	5.38E-05	4.10E-04	2.13E-04	APOE/CASR/ITGB	5 GO
GO:0043409	negative regulation of MAPK cascade	10/212	179/17913	5.53E-05	4.21E-04	2.18E-04	ABL1/APOE/DUSI	10 GO
GO:0008645	hexose transmembrane transport	8/212	112/17913	5.59E-05	4.23E-04	2.19E-04	GRB10/PIK3R1/PI	8 GO
GO:0030968	endoplasmic reticulum unfolded protein response	8/212	112/17913	5.59E-05	4.23E-04	2.19E-04	CCND1/DCTN1/EI	8 GO
GO:0010518	positive regulation of phospholipase activity	6/212	57/17913	5.62E-05	4.23E-04	2.19E-04	KIT/PDGFRA/PDG	6 GO
GO:0043525	positive regulation of neuron apoptotic process	6/212	57/17913	5.62E-05	4.23E-04	2.19E-04	RHOA/CASP3/JUN	6 GO
GO:0045453	bone resorption	6/212	57/17913	5.62E-05	4.23E-04	2.19E-04	PTK2B/INPP5D/IT	6 GO
GO:0000079	regulation of cyclin-dependent protein serine/threonine kinase activity	7/212	83/17913	5.71E-05	4.30E-04	2.23E-04	APC/CCND1/CASI	7 GO
GO:0002758	innate immune response-activating signal transduction	13/212	298/17913	5.79E-05	4.35E-04	2.25E-04	APOB/EP300/ITG	13 GO
GO:0050806	positive regulation of synaptic transmission	9/212	145/17913	5.81E-05	4.35E-04	2.25E-04	ABL1/APOE/DLG4	9 GO
GO:0002223	stimulatory C-type lectin receptor signaling pathway	8/212	113/17913	5.96E-05	4.44E-04	2.30E-04	EP300/LYN/PAK1	8 GO
GO:0014896	muscle hypertrophy	8/212	113/17913	5.96E-05	4.44E-04	2.30E-04	PARP1/IL6ST/SMA	8 GO
GO:1904375	regulation of protein localization to cell periphery	8/212	113/17913	5.96E-05	4.44E-04	2.30E-04	DAB2/EPHA2/LRF	8 GO
GO:0001776	leukocyte homeostasis	7/212	84/17913	6.17E-05	4.59E-04	2.38E-04	ABL1/CASP3/LYN	7 GO
GO:0051057	positive regulation of small GTPase mediated signal transduction	6/212	58/17913	6.20E-05	4.61E-04	2.39E-04	CRKL/LYN/PDGFR	6 GO
GO:0015749	monosaccharide transmembrane transport	8/212	114/17913	6.34E-05	4.70E-04	2.44E-04	GRB10/PIK3R1/PI	8 GO
GO:0010544	negative regulation of platelet activation	4/212	19/17913	6.43E-05	4.75E-04	2.46E-04	APOE/F2/PDGFRA	4 GO
GO:0023019	signal transduction involved in regulation of gene expression	4/212	19/17913	6.43E-05	4.75E-04	2.46E-04	PARP1/SMAD2/S	4 GO
GO:0014910	regulation of smooth muscle cell migration	7/212	85/17913	6.65E-05	4.90E-04	2.54E-04	APEX1/LRP1/SERI	7 GO
GO:0071219	cellular response to molecule of bacterial origin	10/212	183/17913	6.66E-05	4.90E-04	2.54E-04	ABL1/RHOA/LYN/	10 GO
GO:0001941	postsynaptic membrane organization	5/212	37/17913	7.09E-05	5.19E-04	2.69E-04	APOE/CRKL/DLG4	5 GO
GO:0034405	response to fluid shear stress	5/212	37/17913	7.09E-05	5.19E-04	2.69E-04	PTK2B/SMAD7/PI	5 GO
GO:0045124	regulation of bone resorption	5/212	37/17913	7.09E-05	5.19E-04	2.69E-04	INPP5D/ITGB3/PI	5 GO
GO:0062012	regulation of small molecule metabolic process	16/212	439/17913	7.13E-05	5.21E-04	2.70E-04	PARP1/APOB/APC	16 GO
GO:0042129	regulation of T cell proliferation	9/212	149/17913	7.17E-05	5.21E-04	2.70E-04	CASP3/CD4/ERBB	9 GO
GO:0002220	innate immune response activating cell surface receptor signaling pathway	8/212	116/17913	7.17E-05	5.21E-04	2.70E-04	EP300/LYN/PAK1	8 GO

GO:0034219	carbohydrate transmembrane transport	8/212	116/17913	7.17E-05	5.21E-04	2.70E-04	GRB10/PIK3R1/PI	8	GO
GO:0051146	striated muscle cell differentiation	12/212	263/17913	7.30E-05	5.28E-04	2.74E-04	ACTN2/CASP3/FL	12	GO
GO:0002703	regulation of leukocyte mediated immunity	10/212	185/17913	7.30E-05	5.28E-04	2.74E-04	FGR/ITGB2/LYN/S	10	GO
GO:0002697	regulation of immune effector process	16/212	441/17913	7.52E-05	5.43E-04	2.81E-04	CD59/F2/FGR/ITG	16	GO
GO:0002260	lymphocyte homeostasis	6/212	60/17913	7.53E-05	5.43E-04	2.81E-04	ABL1/CASP3/LYN/	6	GO
GO:0060828	regulation of canonical Wnt signaling pathway	12/212	264/17913	7.56E-05	5.45E-04	2.82E-04	APC/APOE/CTNN	12	GO
GO:0001704	formation of primary germ layer	8/212	117/17913	7.62E-05	5.47E-04	2.84E-04	EPHA2/ITGB2/ITG	8	GO
GO:0002761	regulation of myeloid leukocyte differentiation	8/212	117/17913	7.62E-05	5.47E-04	2.84E-04	CD4/INPP5D/JUN	8	GO
GO:1904029	regulation of cyclin-dependent protein kinase activity	7/212	87/17913	7.72E-05	5.54E-04	2.87E-04	APC/CCND1/CASI	7	GO
GO:0009416	response to light stimulus	13/212	307/17913	7.83E-05	5.60E-04	2.90E-04	PARP1/CCND1/C/	13	GO
GO:0010869	regulation of receptor biosynthetic process	4/212	20/17913	7.96E-05	5.66E-04	2.93E-04	HDAC1/ITGB3/PP	4	GO
GO:0030220	platelet formation	4/212	20/17913	7.96E-05	5.66E-04	2.93E-04	CASP3/EP300/PTI	4	GO
GO:0045655	regulation of monocyte differentiation	4/212	20/17913	7.96E-05	5.66E-04	2.93E-04	CD4/INPP5D/JUN	4	GO
GO:0046628	positive regulation of insulin receptor signaling pathway	4/212	20/17913	7.96E-05	5.66E-04	2.93E-04	PAK1/PRKCZ/PTP	4	GO
GO:0017157	regulation of exocytosis	10/212	187/17913	7.98E-05	5.66E-04	2.93E-04	FGR/ITGB2/LYN/I	10	GO
GO:0051928	positive regulation of calcium ion transport	8/212	119/17913	8.59E-05	6.08E-04	3.15E-04	ABL1/CASR/CD4/	8	GO
GO:0060348	bone development	10/212	189/17913	8.72E-05	6.16E-04	3.19E-04	RHOA/EP300/GLI	10	GO
GO:0033044	regulation of chromosome organization	13/212	311/17913	8.92E-05	6.28E-04	3.25E-04	PARP1/APC/SMA	13	GO
GO:0060070	canonical Wnt signaling pathway	13/212	311/17913	8.92E-05	6.28E-04	3.25E-04	APC/APOE/CTNN	13	GO
GO:0046425	regulation of JAK-STAT cascade	8/212	120/17913	9.11E-05	6.41E-04	3.32E-04	F2/PTK2B/IL6ST/I	8	GO
GO:0038179	neurotrophin signaling pathway	5/212	39/17913	9.19E-05	6.44E-04	3.34E-04	CASP3/PTPN11/R	5	GO
GO:0090279	regulation of calcium ion import	5/212	39/17913	9.19E-05	6.44E-04	3.34E-04	CASR/PDGFRB/PI	5	GO
GO:1904062	regulation of cation transmembrane transport	13/212	312/17913	9.21E-05	6.44E-04	3.34E-04	ABL1/ACTN2/DLG	13	GO
GO:0008361	regulation of cell size	9/212	154/17913	9.25E-05	6.45E-04	3.34E-04	ABL1/APOE/RHO/	9	GO
GO:0035051	cardiocyte differentiation	9/212	154/17913	9.25E-05	6.45E-04	3.34E-04	ACTN2/SMAD4/P	9	GO
GO:0050663	cytokine secretion	11/212	230/17913	9.62E-05	6.70E-04	3.47E-04	ABL1/FGR/LRP1/I	11	GO
GO:1903038	negative regulation of leukocyte cell-cell adhesion	8/212	121/17913	9.66E-05	6.71E-04	3.48E-04	CASP3/DUSP3/ER	8	GO
GO:0036120	cellular response to platelet-derived growth factor stimulus	4/212	21/17913	9.75E-05	6.75E-04	3.50E-04	CBL/PDGFRB/SRC	4	GO
GO:0036344	platelet morphogenesis	4/212	21/17913	9.75E-05	6.75E-04	3.50E-04	CASP3/EP300/PTI	4	GO
GO:0043297	apical junction assembly	6/212	63/17913	9.92E-05	6.85E-04	3.55E-04	APC/RHOA/RHOC	6	GO
GO:0046637	regulation of alpha-beta T cell differentiation	6/212	63/17913	9.92E-05	6.85E-04	3.55E-04	RHOA/GLI3/SMAI	6	GO
GO:0034248	regulation of cellular amide metabolic process	16/212	452/17913	1.00E-04	6.90E-04	3.58E-04	APOE/RHOA/CAS	16	GO
GO:0031647	regulation of protein stability	12/212	272/17913	1.00E-04	6.91E-04	3.58E-04	CASP3/EP300/SM	12	GO
GO:0001656	metanephros development	7/212	91/17913	1.03E-04	7.06E-04	3.66E-04	GLI3/SMAD4/MY	7	GO
GO:0043903	regulation of symbiosis, encompassing mutualism through parasitism	11/212	232/17913	1.04E-04	7.12E-04	3.69E-04	APOE/CD4/DDX5/	11	GO
GO:0071241	cellular response to inorganic substance	10/212	194/17913	1.08E-04	7.41E-04	3.84E-04	PARP1/CDH1/DLG	10	GO
GO:0034620	cellular response to unfolded protein	8/212	123/17913	1.08E-04	7.41E-04	3.84E-04	CCND1/DCTN1/EI	8	GO
GO:0007389	pattern specification process	15/212	408/17913	1.09E-04	7.44E-04	3.86E-04	CRKL/EP300/GLI3	15	GO
GO:0043900	regulation of multi-organism process	15/212	408/17913	1.09E-04	7.44E-04	3.86E-04	APOE/CD4/DDX5/	15	GO
GO:2001022	positive regulation of response to DNA damage stimulus	7/212	92/17913	1.10E-04	7.49E-04	3.88E-04	PARP1/DDX5/MY	7	GO
GO:0003158	endothelium development	8/212	124/17913	1.15E-04	7.78E-04	4.03E-04	RHOA/RHOB/CDH	8	GO
GO:0002218	activation of innate immune response	13/212	319/17913	1.15E-04	7.79E-04	4.04E-04	APOB/EP300/ITG	13	GO
GO:0048562	embryonic organ morphogenesis	12/212	276/17913	1.15E-04	7.80E-04	4.04E-04	EPHA2/GLI3/SMA	12	GO
GO:0019048	modulation by virus of host morphology or physiology	5/212	41/17913	1.17E-04	7.92E-04	4.10E-04	CD4/KPNA2/SMA	5	GO
GO:0045621	positive regulation of lymphocyte differentiation	7/212	93/17913	1.18E-04	7.92E-04	4.10E-04	RHOA/GLI3/INPP	7	GO
GO:0031639	plasminogen activation	4/212	22/17913	1.18E-04	7.92E-04	4.10E-04	SERPINE1/PLAT/F	4	GO
GO:0036119	response to platelet-derived growth factor	4/212	22/17913	1.18E-04	7.92E-04	4.10E-04	CBL/PDGFRB/SRC	4	GO
GO:1900078	positive regulation of cellular response to insulin stimulus	4/212	22/17913	1.18E-04	7.92E-04	4.10E-04	PAK1/PRKCZ/PTP	4	GO
GO:0072659	protein localization to plasma membrane	11/212	237/17913	1.25E-04	8.40E-04	4.35E-04	ACTN2/CDH1/DA	11	GO

GO:0045778	positive regulation of ossification	7/212	94/17913	1.26E-04	8.42E-04	4.36E-04	GLI3/IL6ST/JUND/	7	GO
GO:0110020	regulation of actomyosin structure organization	7/212	94/17913	1.26E-04	8.42E-04	4.36E-04	ABL1/RHOA/RHO	7	GO
GO:0042476	odontogenesis	8/212	126/17913	1.28E-04	8.53E-04	4.42E-04	RHOA/GLI3/HDA(	8	GO
GO:0060359	response to ammonium ion	8/212	126/17913	1.28E-04	8.53E-04	4.42E-04	ABL1/CASP3/CRK	8	GO
GO:2000573	positive regulation of DNA biosynthetic process	6/212	66/17913	1.29E-04	8.56E-04	4.43E-04	PTK2B/MYC/PDG	6	GO
GO:0006986	response to unfolded protein	9/212	161/17913	1.30E-04	8.62E-04	4.46E-04	CCND1/DCTN1/EI	9	GO
GO:0001953	negative regulation of cell-matrix adhesion	5/212	42/17913	1.32E-04	8.74E-04	4.53E-04	LRP1/SERPINE1/F	5	GO
GO:0048762	mesenchymal cell differentiation	10/212	199/17913	1.33E-04	8.79E-04	4.55E-04	DAB2/DDX5/SMA	10	GO
GO:0050670	regulation of lymphocyte proliferation	10/212	199/17913	1.33E-04	8.79E-04	4.55E-04	CASP3/CD4/ERBB	10	GO
GO:0050679	positive regulation of epithelial cell proliferation	10/212	199/17913	1.33E-04	8.79E-04	4.55E-04	CCND1/ERBB2/IT	10	GO
GO:0071229	cellular response to acid chemical	10/212	199/17913	1.33E-04	8.79E-04	4.55E-04	APOB/PTK2B/LDL	10	GO
GO:0031334	positive regulation of protein complex assembly	11/212	239/17913	1.35E-04	8.88E-04	4.60E-04	PARP1/RHOA/RH	11	GO
GO:0051100	negative regulation of binding	9/212	162/17913	1.36E-04	8.93E-04	4.63E-04	DAB2/JUN/PPAR/	9	GO
GO:0032944	regulation of mononuclear cell proliferation	10/212	200/17913	1.39E-04	9.11E-04	4.72E-04	CASP3/CD4/ERBB	10	GO
GO:0071404	cellular response to low-density lipoprotein particle stimulus	4/212	23/17913	1.42E-04	9.26E-04	4.80E-04	CASR/ITGB2/LDLI	4	GO
GO:0007498	mesoderm development	8/212	128/17913	1.43E-04	9.31E-04	4.82E-04	EPHA2/ITGB3/SM	8	GO
GO:0014013	regulation of gliogenesis	8/212	128/17913	1.43E-04	9.31E-04	4.82E-04	CRKL/F2/HDAC1/	8	GO
GO:0032355	response to estradiol	8/212	128/17913	1.43E-04	9.31E-04	4.82E-04	APOB/CCND1/CA	8	GO
GO:0050707	regulation of cytokine secretion	10/212	201/17913	1.45E-04	9.41E-04	4.88E-04	ABL1/FGR/LRP1/I	10	GO
GO:1904892	regulation of STAT cascade	8/212	129/17913	1.51E-04	9.80E-04	5.07E-04	F2/PTK2B/IL6ST/I	8	GO
GO:0046323	glucose import	6/212	68/17913	1.52E-04	9.84E-04	5.10E-04	GRB10/PIK3R1/PI	6	GO
GO:0071674	mononuclear cell migration	6/212	68/17913	1.52E-04	9.84E-04	5.10E-04	LYN/CCN3/SERPII	6	GO
GO:0001889	liver development	8/212	130/17913	1.59E-04	1.03E-03	5.33E-04	CCND1/GLI3/JUN	8	GO
GO:0021549	cerebellum development	7/212	98/17913	1.64E-04	1.06E-03	5.47E-04	ABL1/CRKL/PAK1/	7	GO
GO:0033077	T cell differentiation in thymus	6/212	69/17913	1.65E-04	1.06E-03	5.48E-04	ERBB2/GLI3/RAG	6	GO
GO:0019731	antibacterial humoral response	5/212	44/17913	1.65E-04	1.06E-03	5.48E-04	KLK3/PLA2G1B/SI	5	GO
GO:0032459	regulation of protein oligomerization	5/212	44/17913	1.65E-04	1.06E-03	5.48E-04	APOE/RHOC/EP3(	5	GO
GO:0048701	embryonic cranial skeleton morphogenesis	5/212	44/17913	1.65E-04	1.06E-03	5.48E-04	SMAD2/SMAD3/I	5	GO
GO:1900274	regulation of phospholipase C activity	5/212	44/17913	1.65E-04	1.06E-03	5.48E-04	ABL1/KIT/PDGFR/	5	GO
GO:0032800	receptor biosynthetic process	4/212	24/17913	1.68E-04	1.07E-03	5.57E-04	HDAC1/ITGB3/PP	4	GO
GO:0051495	positive regulation of cytoskeleton organization	10/212	205/17913	1.70E-04	1.08E-03	5.62E-04	ABL1/ACTN2/RH(	10	GO
GO:0061136	regulation of proteasomal protein catabolic process	9/212	167/17913	1.71E-04	1.09E-03	5.65E-04	APOE/ARAF/DAB	9	GO
GO:0046632	alpha-beta T cell differentiation	7/212	99/17913	1.75E-04	1.11E-03	5.73E-04	ABL1/RHOA/GLI3	7	GO
GO:0070301	cellular response to hydrogen peroxide	7/212	99/17913	1.75E-04	1.11E-03	5.73E-04	ABL1/APEX1/RHC	7	GO
GO:1902106	negative regulation of leukocyte differentiation	7/212	99/17913	1.75E-04	1.11E-03	5.73E-04	ERBB2/GLI3/INPF	7	GO
GO:0050715	positive regulation of cytokine secretion	8/212	132/17913	1.77E-04	1.12E-03	5.79E-04	ABL1/FGR/LRP1/(	8	GO
GO:0051250	negative regulation of lymphocyte activation	8/212	132/17913	1.77E-04	1.12E-03	5.79E-04	CASP3/DUSP3/ER	8	GO
GO:0006998	nuclear envelope organization	5/212	45/17913	1.84E-04	1.15E-03	5.98E-04	DCTN1/PLK1/PRK	5	GO
GO:0045058	T cell selection	5/212	45/17913	1.84E-04	1.15E-03	5.98E-04	RHOA/CD4/GLI3/	5	GO
GO:0046850	regulation of bone remodeling	5/212	45/17913	1.84E-04	1.15E-03	5.98E-04	INPP5D/ITGB3/PI	5	GO
GO:0071675	regulation of mononuclear cell migration	5/212	45/17913	1.84E-04	1.15E-03	5.98E-04	LYN/CCN3/SERPII	5	GO
GO:0060439	trachea morphogenesis	3/212	10/17913	1.84E-04	1.15E-03	5.98E-04	MAPK1/MAPK3/N	3	GO
GO:1903800	positive regulation of production of miRNAs involved in gene silencing by miRNA	3/212	10/17913	1.84E-04	1.15E-03	5.98E-04	DDX5/MAP2K1/T	3	GO
GO:0097530	granulocyte migration	7/212	100/17913	1.86E-04	1.16E-03	6.02E-04	ITGB2/PECAM1/F	7	GO
GO:0061008	hepaticobiliary system development	8/212	133/17913	1.86E-04	1.16E-03	6.03E-04	CCND1/GLI3/JUN	8	GO
GO:0030324	lung development	9/212	169/17913	1.87E-04	1.17E-03	6.05E-04	EP300/GLI3/SMA	9	GO
GO:0070664	negative regulation of leukocyte proliferation	6/212	71/17913	1.93E-04	1.20E-03	6.22E-04	CASP3/ERBB2/INI	6	GO
GO:1903036	positive regulation of response to wounding	6/212	71/17913	1.93E-04	1.20E-03	6.22E-04	F2/LRP1/SERPINE	6	GO
GO:0048771	tissue remodeling	9/212	170/17913	1.96E-04	1.21E-03	6.29E-04	EPHA2/PTK2B/IN	9	GO

GO:0030194	positive regulation of blood coagulation	4/212	25/17913	1.98E-04	1.22E-03	6.34E-04	F2/SERPINE1/PLG	4	GO
GO:0034110	regulation of homotypic cell-cell adhesion	4/212	25/17913	1.98E-04	1.22E-03	6.34E-04	LYN/PRKCA/PRKC	4	GO
GO:0070897	transcription preinitiation complex assembly	4/212	25/17913	1.98E-04	1.22E-03	6.34E-04	TBP/TP53/UBTF/	4	GO
GO:1900048	positive regulation of hemostasis	4/212	25/17913	1.98E-04	1.22E-03	6.34E-04	F2/SERPINE1/PLG	4	GO
GO:0071354	cellular response to interleukin-6	5/212	46/17913	2.05E-04	1.26E-03	6.53E-04	CBL/IL6ST/SMAD	5	GO
GO:0035967	cellular response to topologically incorrect protein	8/212	135/17913	2.07E-04	1.27E-03	6.58E-04	CCND1/DCTN1/E	8	GO
GO:0043523	regulation of neuron apoptotic process	10/212	210/17913	2.07E-04	1.27E-03	6.58E-04	PARP1/RHOA/CA	10	GO
GO:0023061	signal release	15/212	433/17913	2.09E-04	1.28E-03	6.63E-04	CASR/LRP1/LYN/	15	GO
GO:0051017	actin filament bundle assembly	8/212	136/17913	2.17E-04	1.33E-03	6.88E-04	ABL1/RHOA/RHO	8	GO
GO:1903305	regulation of regulated secretory pathway	8/212	136/17913	2.17E-04	1.33E-03	6.88E-04	FGR/ITGB2/LYN/	8	GO
GO:0030323	respiratory tube development	9/212	173/17913	2.23E-04	1.36E-03	7.04E-04	EP300/GLI3/SMA	9	GO
GO:0032091	negative regulation of protein binding	7/212	103/17913	2.23E-04	1.36E-03	7.04E-04	DAB2/PPARA/PRI	7	GO
GO:0032526	response to retinoic acid	7/212	103/17913	2.23E-04	1.36E-03	7.04E-04	PTK2B/LYN/PAX2	7	GO
GO:0007492	endoderm development	6/212	73/17913	2.25E-04	1.37E-03	7.09E-04	HDAC1/ITGB2/SN	6	GO
GO:0046638	positive regulation of alpha-beta T cell differentiation	5/212	47/17913	2.27E-04	1.38E-03	7.13E-04	RHOA/GLI3/PRKC	5	GO
GO:0001654	eye development	13/212	342/17913	2.28E-04	1.38E-03	7.14E-04	EPHA2/GLI3/HDA	13	GO
GO:0009411	response to UV	8/212	137/17913	2.28E-04	1.38E-03	7.14E-04	PARP1/CCND1/C	8	GO
GO:0061572	actin filament bundle organization	8/212	137/17913	2.28E-04	1.38E-03	7.14E-04	ABL1/RHOA/RHO	8	GO
GO:2000058	regulation of ubiquitin-dependent protein catabolic process	8/212	137/17913	2.28E-04	1.38E-03	7.14E-04	ARAF/DAB2/PTK2	8	GO
GO:0034976	response to endoplasmic reticulum stress	11/212	254/17913	2.29E-04	1.38E-03	7.16E-04	CCND1/DCTN1/E	11	GO
GO:0050820	positive regulation of coagulation	4/212	26/17913	2.32E-04	1.39E-03	7.21E-04	F2/SERPINE1/PLG	4	GO
GO:0061437	renal system vasculature development	4/212	26/17913	2.32E-04	1.39E-03	7.21E-04	PDGFRA/PDGFRB	4	GO
GO:0061440	kidney vasculature development	4/212	26/17913	2.32E-04	1.39E-03	7.21E-04	PDGFRA/PDGFRB	4	GO
GO:1903579	negative regulation of ATP metabolic process	4/212	26/17913	2.32E-04	1.39E-03	7.21E-04	PARP1/RHOA/PP	4	GO
GO:0050868	negative regulation of T cell activation	7/212	104/17913	2.37E-04	1.42E-03	7.34E-04	CASP3/DUSP3/ER	7	GO
GO:0001894	tissue homeostasis	10/212	214/17913	2.41E-04	1.44E-03	7.45E-04	SERPINA3/PTK2B	10	GO
GO:0070373	negative regulation of ERK1 and ERK2 cascade	6/212	74/17913	2.43E-04	1.45E-03	7.49E-04	ABL1/DUSP3/LYN	6	GO
GO:0035966	response to topologically incorrect protein	9/212	175/17913	2.43E-04	1.45E-03	7.50E-04	CCND1/DCTN1/E	9	GO
GO:0044003	modification by symbiont of host morphology or physiology	5/212	48/17913	2.51E-04	1.49E-03	7.70E-04	CD4/KPNA2/SMA	5	GO
GO:0051972	regulation of telomerase activity	5/212	48/17913	2.51E-04	1.49E-03	7.70E-04	MYC/MAPK1/MA	5	GO
GO:0033327	Leydig cell differentiation	3/212	11/17913	2.51E-04	1.49E-03	7.70E-04	CCND1/PDGFRA/	3	GO
GO:0072584	caveolin-mediated endocytosis	3/212	11/17913	2.51E-04	1.49E-03	7.70E-04	MAPK1/MAPK3/	3	GO
GO:1904396	regulation of neuromuscular junction development	3/212	11/17913	2.51E-04	1.49E-03	7.70E-04	CRKL/DCTN1/CO	3	GO
GO:0150063	visual system development	13/212	346/17913	2.55E-04	1.50E-03	7.80E-04	EPHA2/GLI3/HDA	13	GO
GO:0046879	hormone secretion	12/212	301/17913	2.57E-04	1.52E-03	7.86E-04	CASR/LRP1/LYN/	12	GO
GO:0019730	antimicrobial humoral response	7/212	106/17913	2.67E-04	1.57E-03	8.11E-04	KLK3/F2/PLA2G1	7	GO
GO:2000278	regulation of DNA biosynthetic process	7/212	106/17913	2.67E-04	1.57E-03	8.11E-04	PTK2B/MYC/PDG	7	GO
GO:0043921	modulation by host of viral transcription	4/212	27/17913	2.70E-04	1.58E-03	8.20E-04	EP300/HDAC1/JU	4	GO
GO:0052472	modulation by host of symbiont transcription	4/212	27/17913	2.70E-04	1.58E-03	8.20E-04	EP300/HDAC1/JU	4	GO
GO:0045807	positive regulation of endocytosis	8/212	141/17913	2.78E-04	1.63E-03	8.42E-04	APOE/CBL/DAB2/	8	GO
GO:0014015	positive regulation of gliogenesis	6/212	76/17913	2.81E-04	1.64E-03	8.50E-04	CRKL/HDAC1/IL6	6	GO
GO:0022037	metencephalon development	7/212	107/17913	2.82E-04	1.65E-03	8.53E-04	ABL1/CRKL/PAK1	7	GO
GO:2001233	regulation of apoptotic signaling pathway	14/212	397/17913	2.84E-04	1.65E-03	8.56E-04	PARP1/CD44/HD	14	GO
GO:0035265	organ growth	9/212	179/17913	2.88E-04	1.67E-03	8.66E-04	SMAD1/SMAD2/	9	GO
GO:0072006	nephron development	8/212	142/17913	2.92E-04	1.69E-03	8.77E-04	GLI3/SMAD4/MY	8	GO
GO:0048880	sensory system development	13/212	351/17913	2.93E-04	1.70E-03	8.80E-04	EPHA2/GLI3/HDA	13	GO
GO:0032271	regulation of protein polymerization	9/212	180/17913	3.00E-04	1.73E-03	8.98E-04	ABL1/RHOA/DCT	9	GO
GO:0043902	positive regulation of multi-organism process	9/212	180/17913	3.00E-04	1.73E-03	8.98E-04	APOE/CD4/EP300	9	GO
GO:0034644	cellular response to UV	6/212	77/17913	3.02E-04	1.74E-03	9.03E-04	PARP1/EP300/MY	6	GO

GO:0070741	response to interleukin-6	5/212	50/17913	3.04E-04	1.76E-03	9.09E-04	CBL/IL6ST/SMAD	5	GO
GO:0048738	cardiac muscle tissue development	10/212	221/17913	3.11E-04	1.78E-03	9.22E-04	ACTN2/SMAD1/S	10	GO
GO:0000002	mitochondrial genome maintenance	4/212	28/17913	3.12E-04	1.78E-03	9.22E-04	PARP1/TP53/DN	4	GO
GO:0030511	positive regulation of transforming growth factor beta receptor signaling pathway	4/212	28/17913	3.12E-04	1.78E-03	9.22E-04	DAB2/EP300/SM	4	GO
GO:0033688	regulation of osteoblast proliferation	4/212	28/17913	3.12E-04	1.78E-03	9.22E-04	ABL1/RHOA/SMA	4	GO
GO:0044030	regulation of DNA methylation	4/212	28/17913	3.12E-04	1.78E-03	9.22E-04	PARP1/MYC/SPI1	4	GO
GO:0052312	modulation of transcription in other organism involved in symbiotic interaction	4/212	28/17913	3.12E-04	1.78E-03	9.22E-04	EP300/HDAC1/JU	4	GO
GO:0060765	regulation of androgen receptor signaling pathway	4/212	28/17913	3.12E-04	1.78E-03	9.22E-04	DAB2/DDX5/EP3	4	GO
GO:1900543	negative regulation of purine nucleotide metabolic process	4/212	28/17913	3.12E-04	1.78E-03	9.22E-04	PARP1/RHOA/PP	4	GO
GO:1903846	positive regulation of cellular response to transforming growth factor beta stimulus	4/212	28/17913	3.12E-04	1.78E-03	9.22E-04	DAB2/EP300/SM	4	GO
GO:1903052	positive regulation of proteolysis involved in cellular protein catabolic process	7/212	109/17913	3.16E-04	1.80E-03	9.32E-04	DAB2/PTK2B/SM	7	GO
GO:0055013	cardiac muscle cell development	6/212	78/17913	3.24E-04	1.84E-03	9.52E-04	ACTN2/SMAD4/P	6	GO
GO:0010976	positive regulation of neuron projection development	11/212	265/17913	3.30E-04	1.87E-03	9.68E-04	APOE/RHOA/DLG	11	GO
GO:0007077	mitotic nuclear envelope disassembly	3/212	12/17913	3.32E-04	1.87E-03	9.68E-04	PLK1/PRKCA/PRK	3	GO
GO:0035641	locomotory exploration behavior	3/212	12/17913	3.32E-04	1.87E-03	9.68E-04	APOE/DLG4/PRK	3	GO
GO:0038110	interleukin-2-mediated signaling pathway	3/212	12/17913	3.32E-04	1.87E-03	9.68E-04	PTK2B/SHC1/SYK	3	GO
GO:0060391	positive regulation of SMAD protein signal transduction	3/212	12/17913	3.32E-04	1.87E-03	9.68E-04	PARP1/DAB2/SM	3	GO
GO:0071340	skeletal muscle acetylcholine-gated channel clustering	3/212	12/17913	3.32E-04	1.87E-03	9.68E-04	CRKL/COLQ/DNA	3	GO
GO:0001954	positive regulation of cell-matrix adhesion	5/212	51/17913	3.34E-04	1.87E-03	9.68E-04	ABL1/PTK2B/JUP/	5	GO
GO:0030239	myofibril assembly	5/212	51/17913	3.34E-04	1.87E-03	9.68E-04	ACTN2/SMAD4/P	5	GO
GO:0046579	positive regulation of Ras protein signal transduction	5/212	51/17913	3.34E-04	1.87E-03	9.68E-04	CRKL/LYN/PDGFR	5	GO
GO:0048146	positive regulation of fibroblast proliferation	5/212	51/17913	3.34E-04	1.87E-03	9.68E-04	JUN/MYC/PDGFR	5	GO
GO:0051496	positive regulation of stress fiber assembly	5/212	51/17913	3.34E-04	1.87E-03	9.68E-04	ABL1/RHOA/RHO	5	GO
GO:0010811	positive regulation of cell-substrate adhesion	7/212	110/17913	3.34E-04	1.87E-03	9.68E-04	ABL1/CRKL/PTK2	7	GO
GO:0002262	myeloid cell homeostasis	8/212	145/17913	3.36E-04	1.87E-03	9.68E-04	CASP3/INPP5D/K	8	GO
GO:0055002	striated muscle cell development	8/212	145/17913	3.36E-04	1.87E-03	9.68E-04	ACTN2/FLNC/SM	8	GO
GO:0071774	response to fibroblast growth factor	8/212	145/17913	3.36E-04	1.87E-03	9.68E-04	CASR/CBL/CD44/	8	GO
GO:0034765	regulation of ion transmembrane transport	15/212	453/17913	3.38E-04	1.88E-03	9.73E-04	ABL1/ACTN2/DLG	15	GO
GO:0043270	positive regulation of ion transport	11/212	266/17913	3.41E-04	1.89E-03	9.79E-04	ABL1/ACTN2/AP	11	GO
GO:0009914	hormone transport	12/212	311/17913	3.46E-04	1.92E-03	9.93E-04	CASR/LRP1/LYN/	12	GO
GO:0021766	hippocampus development	6/212	79/17913	3.47E-04	1.92E-03	9.93E-04	CASP3/CRKL/GLI	6	GO
GO:0072028	nephron morphogenesis	6/212	79/17913	3.47E-04	1.92E-03	9.93E-04	GLI3/SMAD4/MY	6	GO
GO:0021602	cranial nerve morphogenesis	4/212	29/17913	3.59E-04	1.98E-03	1.03E-03	GLI3/KCNA2/PAX	4	GO
GO:0045980	negative regulation of nucleotide metabolic process	4/212	29/17913	3.59E-04	1.98E-03	1.03E-03	PARP1/RHOA/PP	4	GO
GO:0043536	positive regulation of blood vessel endothelial cell migration	6/212	80/17913	3.71E-04	2.04E-03	1.06E-03	ABL1/PLCG1/PRK	6	GO
GO:0019932	second-messenger-mediated signaling	13/212	360/17913	3.73E-04	2.05E-03	1.06E-03	APOE/CD4/PTK2	13	GO
GO:0030218	erythrocyte differentiation	7/212	112/17913	3.73E-04	2.05E-03	1.06E-03	CASP3/INPP5D/K	7	GO
GO:0042157	lipoprotein metabolic process	7/212	112/17913	3.73E-04	2.05E-03	1.06E-03	APOB/APOE/ITG	7	GO
GO:0008643	carbohydrate transport	8/212	148/17913	3.85E-04	2.11E-03	1.09E-03	GRB10/PIK3R1/P	8	GO
GO:0050770	regulation of axonogenesis	8/212	148/17913	3.85E-04	2.11E-03	1.09E-03	ABL1/APOE/RHO	8	GO
GO:0030510	regulation of BMP signaling pathway	6/212	81/17913	3.97E-04	2.17E-03	1.12E-03	ABL1/SMAD2/SM	6	GO
GO:0051899	membrane depolarization	6/212	81/17913	3.97E-04	2.17E-03	1.12E-03	ABL1/PARP1/JUN	6	GO
GO:0015850	organic hydroxy compound transport	10/212	228/17913	3.99E-04	2.17E-03	1.12E-03	APOB/APOE/KCN	10	GO
GO:0060325	face morphogenesis	4/212	30/17913	4.11E-04	2.23E-03	1.15E-03	EP300/PDGFRA/	4	GO
GO:0090314	positive regulation of protein targeting to membrane	4/212	30/17913	4.11E-04	2.23E-03	1.15E-03	ERBB2/ITGB2/PA	4	GO
GO:1902991	regulation of amyloid precursor protein catabolic process	4/212	30/17913	4.11E-04	2.23E-03	1.15E-03	APOE/CASP3/LYN	4	GO
GO:0019722	calcium-mediated signaling	9/212	188/17913	4.12E-04	2.23E-03	1.16E-03	CD4/PTK2B/PKD2	9	GO
GO:0030902	hindbrain development	8/212	150/17913	4.22E-04	2.28E-03	1.18E-03	ABL1/CRKL/SMA	8	GO
GO:0010745	negative regulation of macrophage derived foam cell differentiation	3/212	13/17913	4.28E-04	2.29E-03	1.19E-03	ITGB3/PPARA/NR	3	GO

GO:0010763	positive regulation of fibroblast migration	3/212	13/17913	4.28E-04	2.29E-03	1.19E-03	PAK1/PRKCE/THB	3	GO
GO:0021554	optic nerve development	3/212	13/17913	4.28E-04	2.29E-03	1.19E-03	GLI3/KCNA2/PAX	3	GO
GO:0034374	low-density lipoprotein particle remodeling	3/212	13/17913	4.28E-04	2.29E-03	1.19E-03	APOB/APOE/PLA	3	GO
GO:0060397	JAK-STAT cascade involved in growth hormone signaling pathway	3/212	13/17913	4.28E-04	2.29E-03	1.19E-03	LYN/PRLR/PTPN1	3	GO
GO:0061029	eyelid development in camera-type eye	3/212	13/17913	4.28E-04	2.29E-03	1.19E-03	HDAC1/JUN/TFA	3	GO
GO:0071352	cellular response to interleukin-2	3/212	13/17913	4.28E-04	2.29E-03	1.19E-03	PTK2B/SHC1/SYK	3	GO
GO:1900272	negative regulation of long-term synaptic potentiation	3/212	13/17913	4.28E-04	2.29E-03	1.19E-03	ABL1/APOE/PRN	3	GO
GO:0001890	placenta development	8/212	151/17913	4.41E-04	2.36E-03	1.22E-03	PKD2/MAPK1/M	8	GO
GO:0006352	DNA-templated transcription, initiation	10/212	231/17913	4.42E-04	2.36E-03	1.22E-03	CCND1/JUN/PPA	10	GO
GO:0071496	cellular response to external stimulus	12/212	320/17913	4.47E-04	2.38E-03	1.24E-03	CASR/CBL/JUN/L	12	GO
GO:0045582	positive regulation of T cell differentiation	6/212	83/17913	4.53E-04	2.41E-03	1.25E-03	RHOA/GLI3/PRKC	6	GO
GO:0051492	regulation of stress fiber assembly	6/212	83/17913	4.53E-04	2.41E-03	1.25E-03	ABL1/RHOA/RHO	6	GO
GO:1903707	negative regulation of hemopoiesis	8/212	152/17913	4.61E-04	2.44E-03	1.27E-03	ERBB2/PTK2B/GL	8	GO
GO:1990845	adaptive thermogenesis	8/212	152/17913	4.61E-04	2.44E-03	1.27E-03	APC/GRB10/PRLR	8	GO
GO:0007569	cell aging	7/212	116/17913	4.62E-04	2.45E-03	1.27E-03	ABL1/SERPINE1/	7	GO
GO:1901654	response to ketone	9/212	191/17913	4.63E-04	2.45E-03	1.27E-03	PARP1/CCND1/C	9	GO
GO:0033687	osteoblast proliferation	4/212	31/17913	4.67E-04	2.46E-03	1.28E-03	ABL1/RHOA/SMA	4	GO
GO:0070570	regulation of neuron projection regeneration	4/212	31/17913	4.67E-04	2.46E-03	1.28E-03	LRP1/PRRX1/MA	4	GO
GO:0010332	response to gamma radiation	5/212	55/17913	4.76E-04	2.50E-03	1.30E-03	PARP1/CBL/MYC/	5	GO
GO:0031102	neuron projection regeneration	5/212	55/17913	4.76E-04	2.50E-03	1.30E-03	JUN/LRP1/PRRX1	5	GO
GO:0031663	lipopolysaccharide-mediated signaling pathway	5/212	55/17913	4.76E-04	2.50E-03	1.30E-03	LYN/PRKCA/PRKC	5	GO
GO:0071897	DNA biosynthetic process	9/212	192/17913	4.80E-04	2.52E-03	1.31E-03	PTK2B/MYC/PCN	9	GO
GO:0055006	cardiac cell development	6/212	84/17913	4.83E-04	2.53E-03	1.31E-03	ACTN2/SMAD4/P	6	GO
GO:0071621	granulocyte chemotaxis	6/212	84/17913	4.83E-04	2.53E-03	1.31E-03	ITGB2/PLA2G1B/	6	GO
GO:0090100	positive regulation of transmembrane receptor protein serine/threonine kinase signaling pathway	6/212	84/17913	4.83E-04	2.53E-03	1.31E-03	PARP1/DAB2/EP	6	GO
GO:0034605	cellular response to heat	7/212	117/17913	4.86E-04	2.54E-03	1.32E-03	EP300/LYN/PRKA	7	GO
GO:0051346	negative regulation of hydrolase activity	14/212	420/17913	4.98E-04	2.60E-03	1.35E-03	ABL1/CD44/PEBP	14	GO
GO:0060541	respiratory system development	9/212	193/17913	4.99E-04	2.60E-03	1.35E-03	EP300/GLI3/SMA	9	GO
GO:0000723	telomere maintenance	8/212	154/17913	5.03E-04	2.61E-03	1.35E-03	PARP1/APEX1/M	8	GO
GO:0045931	positive regulation of mitotic cell cycle	8/212	154/17913	5.03E-04	2.61E-03	1.35E-03	ABL1/APEX1/CCN	8	GO
GO:0048704	embryonic skeletal system morphogenesis	6/212	85/17913	5.15E-04	2.67E-03	1.38E-03	GLI3/SMAD2/SM	6	GO
GO:0060674	placenta blood vessel development	4/212	32/17913	5.29E-04	2.74E-03	1.42E-03	PKD2/MAPK1/M	4	GO
GO:0010885	regulation of cholesterol storage	3/212	14/17913	5.40E-04	2.78E-03	1.44E-03	APOB/PPARA/NR	3	GO
GO:0051123	RNA polymerase II preinitiation complex assembly	3/212	14/17913	5.40E-04	2.78E-03	1.44E-03	TBP/TP53/ATF7IP	3	GO
GO:0070669	response to interleukin-2	3/212	14/17913	5.40E-04	2.78E-03	1.44E-03	PTK2B/SHC1/SYK	3	GO
GO:0072216	positive regulation of metanephros development	3/212	14/17913	5.40E-04	2.78E-03	1.44E-03	MYC/PAX2/PDGF	3	GO
GO:1903351	cellular response to dopamine	3/212	14/17913	5.40E-04	2.78E-03	1.44E-03	ABL1/MAPK1/MA	3	GO
GO:0034103	regulation of tissue remodeling	6/212	86/17913	5.48E-04	2.81E-03	1.45E-03	INPP5D/ITGB3/P	6	GO
GO:0034446	substrate adhesion-dependent cell spreading	6/212	86/17913	5.48E-04	2.81E-03	1.45E-03	ABL1/CRKL/ITGB	6	GO
GO:0048145	regulation of fibroblast proliferation	6/212	86/17913	5.48E-04	2.81E-03	1.45E-03	JUN/MYC/PDGFR	6	GO
GO:0061097	regulation of protein tyrosine kinase activity	6/212	86/17913	5.48E-04	2.81E-03	1.45E-03	DLG4/PRNP/PTP	6	GO
GO:0046686	response to cadmium ion	5/212	57/17913	5.62E-04	2.88E-03	1.49E-03	JUN/KIT/PCNA/M	5	GO
GO:0034101	erythrocyte homeostasis	7/212	120/17913	5.66E-04	2.89E-03	1.50E-03	CASP3/INPP5D/K	7	GO
GO:0090068	positive regulation of cell cycle process	11/212	283/17913	5.73E-04	2.92E-03	1.52E-03	APEX1/RHOA/CC	11	GO
GO:0048144	fibroblast proliferation	6/212	87/17913	5.83E-04	2.97E-03	1.54E-03	JUN/MYC/PDGFR	6	GO
GO:0060021	roof of mouth development	6/212	87/17913	5.83E-04	2.97E-03	1.54E-03	GLI3/SMAD2/SM	6	GO
GO:0055007	cardiac muscle cell differentiation	7/212	121/17913	5.95E-04	3.01E-03	1.56E-03	ACTN2/SMAD4/P	7	GO
GO:0002244	hematopoietic progenitor cell differentiation	8/212	158/17913	5.95E-04	3.01E-03	1.56E-03	ABL1/KIT/OSM/E	8	GO
GO:0009408	response to heat	8/212	158/17913	5.95E-04	3.01E-03	1.56E-03	EP300/LYN/PRKA	8	GO

GO:0030947	regulation of vascular endothelial growth factor receptor signaling pathway	4/212	33/17913	5.96E-04	3.01E-03	1.56E-03	GRB10/ITGB3/PR	4	GO
GO:0045736	negative regulation of cyclin-dependent protein serine/threonine kinase activity	4/212	33/17913	5.96E-04	3.01E-03	1.56E-03	APC/CASP3/PLK1,	4	GO
GO:0048011	neurotrophin TRK receptor signaling pathway	4/212	33/17913	5.96E-04	3.01E-03	1.56E-03	CASP3/PTPN11/S	4	GO
GO:0098751	bone cell development	4/212	33/17913	5.96E-04	3.01E-03	1.56E-03	EP300/KIT/PTPN6	4	GO
GO:0002699	positive regulation of immune effector process	9/212	198/17913	6.00E-04	3.02E-03	1.57E-03	FGR/ITGB2/LYN/F	9	GO
GO:0030178	negative regulation of Wnt signaling pathway	9/212	198/17913	6.00E-04	3.02E-03	1.57E-03	APC/APOE/CTNN	9	GO
GO:0032233	positive regulation of actin filament bundle assembly	5/212	58/17913	6.10E-04	3.07E-03	1.59E-03	ABL1/RHOA/RHO	5	GO
GO:0097237	cellular response to toxic substance	10/212	241/17913	6.15E-04	3.09E-03	1.60E-03	ABL1/APEX1/APC	10	GO
GO:0007266	Rho protein signal transduction	8/212	159/17913	6.21E-04	3.11E-03	1.61E-03	ABL1/APOE/RHO/	8	GO
GO:0046488	phosphatidylinositol metabolic process	8/212	159/17913	6.21E-04	3.11E-03	1.61E-03	INPP5D/PDGFRB/	8	GO
GO:0055001	muscle cell development	8/212	159/17913	6.21E-04	3.11E-03	1.61E-03	ACTN2/FLNC/SM/	8	GO
GO:0034763	negative regulation of transmembrane transport	7/212	122/17913	6.25E-04	3.13E-03	1.62E-03	ACTN2/GRB10/PI	7	GO
GO:0007254	JNK cascade	9/212	200/17913	6.45E-04	3.22E-03	1.67E-03	CASR/CRKL/DUSF	9	GO
GO:0061448	connective tissue development	10/212	243/17913	6.55E-04	3.27E-03	1.69E-03	CASR/CD44/GLI3/	10	GO
GO:0044728	DNA methylation or demethylation	6/212	89/17913	6.58E-04	3.28E-03	1.70E-03	PARP1/APEX1/MY	6	GO
GO:0090303	positive regulation of wound healing	5/212	59/17913	6.60E-04	3.28E-03	1.70E-03	F2/SERPINE1/PLG	5	GO
GO:0032486	Rap protein signal transduction	3/212	15/17913	6.69E-04	3.30E-03	1.71E-03	CBL/RAP1A/RAP2	3	GO
GO:1903350	response to dopamine	3/212	15/17913	6.69E-04	3.30E-03	1.71E-03	ABL1/MAPK1/MA	3	GO
GO:1903358	regulation of Golgi organization	3/212	15/17913	6.69E-04	3.30E-03	1.71E-03	MAPK1/MAPK3/N	3	GO
GO:0034142	toll-like receptor 4 signaling pathway	4/212	34/17913	6.70E-04	3.30E-03	1.71E-03	ITGB2/LYN/PRKCI	4	GO
GO:0071868	cellular response to monoamine stimulus	4/212	34/17913	6.70E-04	3.30E-03	1.71E-03	ABL1/PRKACA/M/	4	GO
GO:0071870	cellular response to catecholamine stimulus	4/212	34/17913	6.70E-04	3.30E-03	1.71E-03	ABL1/PRKACA/M/	4	GO
GO:1904030	negative regulation of cyclin-dependent protein kinase activity	4/212	34/17913	6.70E-04	3.30E-03	1.71E-03	APC/CASP3/PLK1,	4	GO
GO:2000249	regulation of actin cytoskeleton reorganization	4/212	34/17913	6.70E-04	3.30E-03	1.71E-03	ABL1/PTK2B/PDG	4	GO
GO:0016569	covalent chromatin modification	14/212	433/17913	6.72E-04	3.31E-03	1.71E-03	EP300/HDAC1/SN	14	GO
GO:0015908	fatty acid transport	6/212	90/17913	6.98E-04	3.42E-03	1.77E-03	APOE/PLA2G1B/F	6	GO
GO:0046634	regulation of alpha-beta T cell activation	6/212	90/17913	6.98E-04	3.42E-03	1.77E-03	RHOA/GLI3/SMAI	6	GO
GO:0050764	regulation of phagocytosis	6/212	90/17913	6.98E-04	3.42E-03	1.77E-03	FGR/LRP1/PRKCG	6	GO
GO:0046486	glycerolipid metabolic process	13/212	385/17913	7.00E-04	3.43E-03	1.78E-03	APOB/APOE/INPF	13	GO
GO:0001658	branching involved in ureteric bud morphogenesis	5/212	60/17913	7.13E-04	3.48E-03	1.80E-03	GLI3/SMAD4/MY	5	GO
GO:0009880	embryonic pattern specification	5/212	60/17913	7.13E-04	3.48E-03	1.80E-03	SMAD1/SMAD2/S	5	GO
GO:0046324	regulation of glucose import	5/212	60/17913	7.13E-04	3.48E-03	1.80E-03	GRB10/PIK3R1/PI	5	GO
GO:1905953	negative regulation of lipid localization	5/212	60/17913	7.13E-04	3.48E-03	1.80E-03	ITGB3/PPARA/PT	5	GO
GO:2001242	regulation of intrinsic apoptotic signaling pathway	8/212	163/17913	7.31E-04	3.56E-03	1.84E-03	PARP1/CD44/HD/	8	GO
GO:0010717	regulation of epithelial to mesenchymal transition	6/212	91/17913	7.40E-04	3.59E-03	1.86E-03	DAB2/SMAD2/SN	6	GO
GO:1901184	regulation of ERBB signaling pathway	6/212	91/17913	7.40E-04	3.59E-03	1.86E-03	CBL/DUSP3/ERBB	6	GO
GO:0001893	maternal placenta development	4/212	35/17913	7.49E-04	3.62E-03	1.88E-03	MAPK1/MAPK3/F	4	GO
GO:0030224	monocyte differentiation	4/212	35/17913	7.49E-04	3.62E-03	1.88E-03	CD4/INPP5D/JUN	4	GO
GO:0060323	head morphogenesis	4/212	35/17913	7.49E-04	3.62E-03	1.88E-03	EP300/PDGFRA/F	4	GO
GO:1903131	mononuclear cell differentiation	4/212	35/17913	7.49E-04	3.62E-03	1.88E-03	CD4/INPP5D/JUN	4	GO
GO:0051592	response to calcium ion	7/212	126/17913	7.57E-04	3.66E-03	1.89E-03	CCND1/CASR/PTK	7	GO
GO:0070588	calcium ion transmembrane transport	11/212	293/17913	7.63E-04	3.68E-03	1.91E-03	ABL1/F2/PTK2B/L	11	GO
GO:0030032	lamellipodium assembly	5/212	61/17913	7.69E-04	3.70E-03	1.92E-03	EPHA2/KIT/VAV2,	5	GO
GO:0046635	positive regulation of alpha-beta T cell activation	5/212	61/17913	7.69E-04	3.70E-03	1.92E-03	RHOA/GLI3/PRKC	5	GO
GO:0048645	animal organ formation	5/212	61/17913	7.69E-04	3.70E-03	1.92E-03	GLI3/PAX2/MAPK	5	GO
GO:0090596	sensory organ morphogenesis	10/212	250/17913	8.15E-04	3.89E-03	2.02E-03	EPHA2/GLI3/HDA	10	GO
GO:2000027	regulation of animal organ morphogenesis	10/212	250/17913	8.15E-04	3.89E-03	2.02E-03	ABL1/RHOA/DAB	10	GO
GO:0010875	positive regulation of cholesterol efflux	3/212	16/17913	8.17E-04	3.89E-03	2.02E-03	APOE/LRP1/NR1H	3	GO
GO:0010878	cholesterol storage	3/212	16/17913	8.17E-04	3.89E-03	2.02E-03	APOB/PPARA/NR	3	GO

GO:0070242	thymocyte apoptotic process	3/212	16/17913	8.17E-04	3.89E-03	2.02E-03	GLI3/RAG1/TP53	3	GO
GO:0072224	metanephric glomerulus development	3/212	16/17913	8.17E-04	3.89E-03	2.02E-03	PAX2/PDGFRA/PI	3	GO
GO:1900221	regulation of amyloid-beta clearance	3/212	16/17913	8.17E-04	3.89E-03	2.02E-03	APOE/HDAC1/LRI	3	GO
GO:0021543	pallium development	8/212	166/17913	8.23E-04	3.92E-03	2.03E-03	RHOA/CASP3/CRI	8	GO
GO:1901215	negative regulation of neuron death	9/212	207/17913	8.24E-04	3.92E-03	2.03E-03	APOE/RHOA/CBL,	9	GO
GO:0046825	regulation of protein export from nucleus	4/212	36/17913	8.35E-04	3.96E-03	2.05E-03	PPM1A/PRKACA/	4	GO
GO:0071634	regulation of transforming growth factor beta production	4/212	36/17913	8.35E-04	3.96E-03	2.05E-03	SMAD3/SMAD4/	4	GO
GO:0090313	regulation of protein targeting to membrane	4/212	36/17913	8.35E-04	3.96E-03	2.05E-03	ERBB2/ITGB2/PA	4	GO
GO:0031668	cellular response to extracellular stimulus	10/212	251/17913	8.40E-04	3.98E-03	2.06E-03	CASR/CBL/JUN/L	10	GO
GO:0032200	telomere organization	8/212	167/17913	8.56E-04	4.05E-03	2.10E-03	PARP1/APEX1/M	8	GO
GO:0050808	synapse organization	13/212	394/17913	8.66E-04	4.09E-03	2.12E-03	ABL1/APOE/RHO	13	GO
GO:0051260	protein homooligomerization	12/212	345/17913	8.66E-04	4.09E-03	2.12E-03	ACTN2/APC/APO	12	GO
GO:0032231	regulation of actin filament bundle assembly	6/212	94/17913	8.77E-04	4.13E-03	2.14E-03	ABL1/RHOA/RHO	6	GO
GO:0048709	oligodendrocyte differentiation	6/212	94/17913	8.77E-04	4.13E-03	2.14E-03	ERBB2/GLI3/HDA	6	GO
GO:0050854	regulation of antigen receptor-mediated signaling pathway	5/212	63/17913	8.92E-04	4.19E-03	2.17E-03	DUSP3/LYN/PRK	5	GO
GO:0050921	positive regulation of chemotaxis	7/212	130/17913	9.11E-04	4.28E-03	2.22E-03	CASR/PTK2B/SM	7	GO
GO:0010951	negative regulation of endopeptidase activity	9/212	210/17913	9.12E-04	4.28E-03	2.22E-03	CD44/PEBP1/SER	9	GO
GO:0010614	negative regulation of cardiac muscle hypertrophy	4/212	37/17913	9.27E-04	4.33E-03	2.24E-03	SMAD3/SMAD4/	4	GO
GO:0010955	negative regulation of protein processing	4/212	37/17913	9.27E-04	4.33E-03	2.24E-03	CD59/SERPINE1/I	4	GO
GO:1903318	negative regulation of protein maturation	4/212	37/17913	9.27E-04	4.33E-03	2.24E-03	CD59/SERPINE1/I	4	GO
GO:0008585	female gonad development	6/212	95/17913	9.28E-04	4.33E-03	2.24E-03	CASP3/KIT/SMAD	6	GO
GO:0060993	kidney morphogenesis	6/212	95/17913	9.28E-04	4.33E-03	2.24E-03	GLI3/SMAD4/MY	6	GO
GO:0042770	signal transduction in response to DNA damage	7/212	131/17913	9.53E-04	4.44E-03	2.30E-03	ABL1/CD44/DDX	7	GO
GO:0015909	long-chain fatty acid transport	5/212	64/17913	9.58E-04	4.45E-03	2.31E-03	APOE/PLA2G1B/	5	GO
GO:0019915	lipid storage	5/212	64/17913	9.58E-04	4.45E-03	2.31E-03	APOB/APOE/ITGE	5	GO
GO:0030177	positive regulation of Wnt signaling pathway	8/212	170/17913	9.61E-04	4.46E-03	2.31E-03	ABL1/DAB2/JUP/	8	GO
GO:0043583	ear development	9/212	212/17913	9.75E-04	4.51E-03	2.34E-03	GLI3/PAX2/PDGF	9	GO
GO:0030219	megakaryocyte differentiation	6/212	96/17913	9.80E-04	4.51E-03	2.34E-03	EP300/HDAC1/KI	6	GO
GO:0034199	activation of protein kinase A activity	3/212	17/17913	9.83E-04	4.51E-03	2.34E-03	PRKACA/PRKAR1	3	GO
GO:0042953	lipoprotein transport	3/212	17/17913	9.83E-04	4.51E-03	2.34E-03	APOB/LRP1/PRK	3	GO
GO:0043923	positive regulation by host of viral transcription	3/212	17/17913	9.83E-04	4.51E-03	2.34E-03	EP300/JUN/SP1	3	GO
GO:0044872	lipoprotein localization	3/212	17/17913	9.83E-04	4.51E-03	2.34E-03	APOB/LRP1/PRK	3	GO
GO:0045056	transcytosis	3/212	17/17913	9.83E-04	4.51E-03	2.34E-03	LRP1/RAB5A/SRC	3	GO
GO:0048532	anatomical structure arrangement	3/212	17/17913	9.83E-04	4.51E-03	2.34E-03	KCNA2/PAX2/TF	3	GO
GO:0055003	cardiac myofibril assembly	3/212	17/17913	9.83E-04	4.51E-03	2.34E-03	SMAD4/PDGFRA/	3	GO
GO:0060438	trachea development	3/212	17/17913	9.83E-04	4.51E-03	2.34E-03	MAPK1/MAPK3/	3	GO
GO:0048565	digestive tract development	7/212	132/17913	9.96E-04	4.57E-03	2.37E-03	CRKL/GLI3/KIT/S	7	GO
GO:0006284	base-excision repair	4/212	38/17913	1.03E-03	4.67E-03	2.42E-03	APEX1/PCNA/PO	4	GO
GO:0016572	histone phosphorylation	4/212	38/17913	1.03E-03	4.67E-03	2.42E-03	PRKCA/PRKCB/P	4	GO
GO:0043277	apoptotic cell clearance	4/212	38/17913	1.03E-03	4.67E-03	2.42E-03	ITGB3/LRP1/THB	4	GO
GO:0071604	transforming growth factor beta production	4/212	38/17913	1.03E-03	4.67E-03	2.42E-03	SMAD3/SMAD4/	4	GO
GO:0071867	response to monoamine	4/212	38/17913	1.03E-03	4.67E-03	2.42E-03	ABL1/PRKACA/M	4	GO
GO:0071869	response to catecholamine	4/212	38/17913	1.03E-03	4.67E-03	2.42E-03	ABL1/PRKACA/M	4	GO
GO:0032945	negative regulation of mononuclear cell proliferation	5/212	65/17913	1.03E-03	4.67E-03	2.42E-03	CASP3/ERBB2/IN	5	GO
GO:0050672	negative regulation of lymphocyte proliferation	5/212	65/17913	1.03E-03	4.67E-03	2.42E-03	CASP3/ERBB2/IN	5	GO
GO:0071242	cellular response to ammonium ion	5/212	65/17913	1.03E-03	4.67E-03	2.42E-03	ABL1/CASP3/CRK	5	GO
GO:0048193	Golgi vesicle transport	11/212	304/17913	1.03E-03	4.67E-03	2.42E-03	CD59/DCTN1/F2/	11	GO
GO:0006888	ER to Golgi vesicle-mediated transport	8/212	172/17913	1.04E-03	4.69E-03	2.43E-03	CD59/DCTN1/F2/	8	GO
GO:0043123	positive regulation of I-kappaB kinase/NF-kappaB signaling	8/212	172/17913	1.04E-03	4.69E-03	2.43E-03	ABL1/RHOA/RHO	8	GO

GO:0046631	alpha-beta T cell activation	7/212	133/17913	1.04E-03	4.71E-03	2.44E-03	ABL1/RHOA/GLI3	7	GO
GO:0007517	muscle organ development	13/212	403/17913	1.06E-03	4.80E-03	2.49E-03	RHOA/DDX5/EP3	13	GO
GO:0070613	regulation of protein processing	8/212	173/17913	1.08E-03	4.84E-03	2.51E-03	CD59/F2/SERPIN	8	GO
GO:0071478	cellular response to radiation	8/212	173/17913	1.08E-03	4.84E-03	2.51E-03	PARP1/RHOB/EP	8	GO
GO:0046578	regulation of Ras protein signal transduction	9/212	215/17913	1.08E-03	4.84E-03	2.51E-03	ABL1/APOE/CBL/	9	GO
GO:0010769	regulation of cell morphogenesis involved in differentiation	10/212	260/17913	1.10E-03	4.93E-03	2.56E-03	ABL1/APOE/RHO	10	GO
GO:0043030	regulation of macrophage activation	5/212	66/17913	1.10E-03	4.94E-03	2.56E-03	ITGB2/LDLR/THB	5	GO
GO:0060675	ureteric bud morphogenesis	5/212	66/17913	1.10E-03	4.94E-03	2.56E-03	GLI3/SMAD4/MY	5	GO
GO:0045666	positive regulation of neuron differentiation	12/212	355/17913	1.11E-03	4.96E-03	2.57E-03	APOE/RHOA/DLG	12	GO
GO:0014741	negative regulation of muscle hypertrophy	4/212	39/17913	1.13E-03	5.08E-03	2.63E-03	SMAD3/SMAD4/	4	GO
GO:0045930	negative regulation of mitotic cell cycle	11/212	308/17913	1.14E-03	5.11E-03	2.65E-03	ABL1/APC/CCND	11	GO
GO:0010596	negative regulation of endothelial cell migration	6/212	99/17913	1.15E-03	5.14E-03	2.66E-03	APOE/RHOA/JUP	6	GO
GO:1903317	regulation of protein maturation	8/212	175/17913	1.16E-03	5.17E-03	2.68E-03	CD59/F2/SERPIN	8	GO
GO:0006346	methylation-dependent chromatin silencing	3/212	18/17913	1.17E-03	5.20E-03	2.69E-03	HDAC1/SETDB1/	3	GO
GO:0010888	negative regulation of lipid storage	3/212	18/17913	1.17E-03	5.20E-03	2.69E-03	ITGB3/PPARA/NR	3	GO
GO:0051895	negative regulation of focal adhesion assembly	3/212	18/17913	1.17E-03	5.20E-03	2.69E-03	LRP1/SRC/THBS1	3	GO
GO:0042531	positive regulation of tyrosine phosphorylation of STAT protein	5/212	67/17913	1.18E-03	5.22E-03	2.71E-03	IL6ST/KIT/LYN/O	5	GO
GO:0045669	positive regulation of osteoblast differentiation	5/212	67/17913	1.18E-03	5.22E-03	2.71E-03	GLI3/IL6ST/JUND	5	GO
GO:0070227	lymphocyte apoptotic process	5/212	67/17913	1.18E-03	5.22E-03	2.71E-03	CRKL/GLI3/RAG1	5	GO
GO:0072171	mesonephric tubule morphogenesis	5/212	67/17913	1.18E-03	5.22E-03	2.71E-03	GLI3/SMAD4/MY	5	GO
GO:0050807	regulation of synapse organization	9/212	218/17913	1.19E-03	5.24E-03	2.72E-03	ABL1/APOE/RHO	9	GO
GO:0046545	development of primary female sexual characteristics	6/212	100/17913	1.21E-03	5.35E-03	2.77E-03	CASP3/KIT/SMAD	6	GO
GO:2000045	regulation of G1/S transition of mitotic cell cycle	8/212	177/17913	1.25E-03	5.50E-03	2.85E-03	APEX1/CCND1/E	8	GO
GO:0042987	amyloid precursor protein catabolic process	4/212	40/17913	1.25E-03	5.50E-03	2.85E-03	APOE/CASP3/LYN	4	GO
GO:0051281	positive regulation of release of sequestered calcium ion into cytosol	4/212	40/17913	1.25E-03	5.50E-03	2.85E-03	ABL1/F2/PKD2/P	4	GO
GO:0032436	positive regulation of proteasomal ubiquitin-dependent protein catabolic process	5/212	68/17913	1.26E-03	5.54E-03	2.87E-03	DAB2/SMAD7/PL	5	GO
GO:0042698	ovulation cycle	5/212	68/17913	1.26E-03	5.54E-03	2.87E-03	CASP3/PCNA/PD	5	GO
GO:0031669	cellular response to nutrient levels	9/212	220/17913	1.26E-03	5.54E-03	2.87E-03	CASR/CBL/JUN/	9	GO
GO:0036294	cellular response to decreased oxygen levels	9/212	220/17913	1.26E-03	5.54E-03	2.87E-03	CASR/CBL/EP300	9	GO
GO:0010770	positive regulation of cell morphogenesis involved in differentiation	7/212	138/17913	1.29E-03	5.64E-03	2.92E-03	ABL1/RHOA/CRK	7	GO
GO:0030879	mammary gland development	7/212	138/17913	1.29E-03	5.64E-03	2.92E-03	CCND1/EPHA2/G	7	GO
GO:0044344	cellular response to fibroblast growth factor stimulus	7/212	138/17913	1.29E-03	5.64E-03	2.92E-03	CBL/CD44/CRKL/	7	GO
GO:0031623	receptor internalization	6/212	102/17913	1.34E-03	5.85E-03	3.03E-03	DLG4/ITGB2/ITG	6	GO
GO:0050803	regulation of synapse structure or activity	9/212	222/17913	1.35E-03	5.85E-03	3.03E-03	ABL1/APOE/RHO	9	GO
GO:0071322	cellular response to carbohydrate stimulus	7/212	139/17913	1.35E-03	5.85E-03	3.03E-03	CASR/LRP1/PAX2	7	GO
GO:0000281	mitotic cytokinesis	5/212	69/17913	1.35E-03	5.85E-03	3.03E-03	APC/RHOA/RHO	5	GO
GO:0070509	calcium ion import	5/212	69/17913	1.35E-03	5.85E-03	3.03E-03	CASR/PDGFRB/P	5	GO
GO:0072210	metanephric nephron development	4/212	41/17913	1.37E-03	5.94E-03	3.08E-03	PAX2/PDGFRA/P	4	GO
GO:1903053	regulation of extracellular matrix organization	4/212	41/17913	1.37E-03	5.94E-03	3.08E-03	ABL1/LRP1/SMA	4	GO
GO:0030949	positive regulation of vascular endothelial growth factor receptor signaling pathway	3/212	19/17913	1.38E-03	5.94E-03	3.08E-03	GRB10/ITGB3/PR	3	GO
GO:0032042	mitochondrial DNA metabolic process	3/212	19/17913	1.38E-03	5.94E-03	3.08E-03	PARP1/TP53/DN	3	GO
GO:0050860	negative regulation of T cell receptor signaling pathway	3/212	19/17913	1.38E-03	5.94E-03	3.08E-03	DUSP3/PRNP/PT	3	GO
GO:1903392	negative regulation of adherens junction organization	3/212	19/17913	1.38E-03	5.94E-03	3.08E-03	LRP1/SRC/THBS1	3	GO
GO:1903798	regulation of production of miRNAs involved in gene silencing by miRNA	3/212	19/17913	1.38E-03	5.94E-03	3.08E-03	DDX5/MAP2K1/T	3	GO
GO:0010466	negative regulation of peptidase activity	9/212	223/17913	1.39E-03	5.98E-03	3.10E-03	CD44/PEBP1/SER	9	GO
GO:0001837	epithelial to mesenchymal transition	7/212	140/17913	1.40E-03	6.04E-03	3.13E-03	DAB2/DDX5/SM	7	GO
GO:0048524	positive regulation of viral process	6/212	103/17913	1.41E-03	6.07E-03	3.15E-03	APOE/CD4/EP30	6	GO
GO:0000082	G1/S transition of mitotic cell cycle	10/212	269/17913	1.42E-03	6.09E-03	3.15E-03	APEX1/CCND1/E	10	GO
GO:0043122	regulation of I-kappaB kinase/NF-kappaB signaling	9/212	224/17913	1.43E-03	6.14E-03	3.18E-03	ABL1/RHOA/RHO	9	GO

GO:0010721	negative regulation of cell development	11/212	317/17913	1.44E-03	6.18E-03	3.20E-03	APOE/RHOA/F2/(	11	GO
GO:0003002	regionalization	11/212	318/17913	1.48E-03	6.33E-03	3.28E-03	CRKL/EP300/GLI3	11	GO
GO:0009952	anterior/posterior pattern specification	8/212	182/17913	1.49E-03	6.36E-03	3.29E-03	CRKL/EP300/GLI3	8	GO
GO:0051216	cartilage development	8/212	182/17913	1.49E-03	6.36E-03	3.29E-03	CD44/GLI3/SMAD	8	GO
GO:0010863	positive regulation of phospholipase C activity	4/212	42/17913	1.50E-03	6.40E-03	3.32E-03	KIT/PDGFRA/PDG	4	GO
GO:0045429	positive regulation of nitric oxide biosynthetic process	4/212	42/17913	1.50E-03	6.40E-03	3.32E-03	PTK2B/ITGB2/SM	4	GO
GO:0001678	cellular glucose homeostasis	7/212	142/17913	1.52E-03	6.48E-03	3.36E-03	CASR/LRP1/PAX2	7	GO
GO:0007612	learning	7/212	142/17913	1.52E-03	6.48E-03	3.36E-03	BCHE/DLG4/JUN/	7	GO
GO:0006305	DNA alkylation	5/212	71/17913	1.53E-03	6.50E-03	3.37E-03	PARP1/MYC/SPI1	5	GO
GO:0006306	DNA methylation	5/212	71/17913	1.53E-03	6.50E-03	3.37E-03	PARP1/MYC/SPI1	5	GO
GO:0030500	regulation of bone mineralization	5/212	71/17913	1.53E-03	6.50E-03	3.37E-03	PTK2B/NBR1/SM/	5	GO
GO:0016570	histone modification	13/212	420/17913	1.54E-03	6.53E-03	3.38E-03	EP300/HDAC1/SN	13	GO
GO:0002688	regulation of leukocyte chemotaxis	6/212	105/17913	1.56E-03	6.59E-03	3.42E-03	PTK2B/LYN/CCN3	6	GO
GO:0045446	endothelial cell differentiation	6/212	105/17913	1.56E-03	6.59E-03	3.42E-03	CDH5/SMAD4/PE	6	GO
GO:0042593	glucose homeostasis	9/212	227/17913	1.57E-03	6.63E-03	3.43E-03	CASR/LRP1/PAX2	9	GO
GO:0055123	digestive system development	7/212	143/17913	1.58E-03	6.68E-03	3.46E-03	CRKL/GLI3/KIT/SN	7	GO
GO:0106106	cold-induced thermogenesis	7/212	143/17913	1.58E-03	6.68E-03	3.46E-03	APC/GRB10/PRLF	7	GO
GO:0120161	regulation of cold-induced thermogenesis	7/212	143/17913	1.58E-03	6.68E-03	3.46E-03	APC/GRB10/PRLF	7	GO
GO:0043586	tongue development	3/212	20/17913	1.61E-03	6.76E-03	3.50E-03	GLI3/HDAC1/KIT	3	GO
GO:0033500	carbohydrate homeostasis	9/212	228/17913	1.62E-03	6.80E-03	3.52E-03	CASR/LRP1/PAX2	9	GO
GO:0035019	somatic stem cell population maintenance	5/212	72/17913	1.63E-03	6.80E-03	3.52E-03	KIT/SMAD2/SMA	5	GO
GO:0050772	positive regulation of axonogenesis	5/212	72/17913	1.63E-03	6.80E-03	3.52E-03	RHOA/LRP1/PAK1	5	GO
GO:1990266	neutrophil migration	5/212	72/17913	1.63E-03	6.80E-03	3.52E-03	ITGB2/PECAM1/F	5	GO
GO:0021761	limbic system development	6/212	106/17913	1.64E-03	6.80E-03	3.52E-03	CASP3/CRKL/GLI3	6	GO
GO:0043279	response to alkaloid	6/212	106/17913	1.64E-03	6.80E-03	3.52E-03	BCHE/CASP3/PTK	6	GO
GO:0098693	regulation of synaptic vesicle cycle	6/212	106/17913	1.64E-03	6.80E-03	3.52E-03	PRKAR1B/PRKCB/	6	GO
GO:0010613	positive regulation of cardiac muscle hypertrophy	4/212	43/17913	1.64E-03	6.80E-03	3.52E-03	PARP1/IL6ST/PRK	4	GO
GO:0010761	fibroblast migration	4/212	43/17913	1.64E-03	6.80E-03	3.52E-03	CCN3/PAK1/PRKC	4	GO
GO:0010883	regulation of lipid storage	4/212	43/17913	1.64E-03	6.80E-03	3.52E-03	APOB/ITGB3/PPA	4	GO
GO:0043300	regulation of leukocyte degranulation	4/212	43/17913	1.64E-03	6.80E-03	3.52E-03	FGR/ITGB2/LYN/S	4	GO
GO:0043303	mast cell degranulation	4/212	43/17913	1.64E-03	6.80E-03	3.52E-03	CBL/FGR/KIT/LYN	4	GO
GO:0050850	positive regulation of calcium-mediated signaling	4/212	43/17913	1.64E-03	6.80E-03	3.52E-03	CD4/PKD2/SYK/Z/	4	GO
GO:0051154	negative regulation of striated muscle cell differentiation	4/212	43/17913	1.64E-03	6.80E-03	3.52E-03	SMAD4/CCN3/PA	4	GO
GO:1904407	positive regulation of nitric oxide metabolic process	4/212	43/17913	1.64E-03	6.80E-03	3.52E-03	PTK2B/ITGB2/SM	4	GO
GO:0043271	negative regulation of ion transport	7/212	144/17913	1.65E-03	6.83E-03	3.54E-03	ACTN2/PTK2B/PK	7	GO
GO:0002685	regulation of leukocyte migration	8/212	186/17913	1.71E-03	7.06E-03	3.66E-03	RHOA/CRKL/PTK2	8	GO
GO:0006959	humoral immune response	10/212	276/17913	1.71E-03	7.08E-03	3.67E-03	KLK3/CD59/F2/PL	10	GO
GO:0001938	positive regulation of endothelial cell proliferation	6/212	107/17913	1.72E-03	7.08E-03	3.67E-03	ITGB3/JUN/PLCG1	6	GO
GO:0062014	negative regulation of small molecule metabolic process	6/212	107/17913	1.72E-03	7.08E-03	3.67E-03	PARP1/APOE/RH(	6	GO
GO:0042391	regulation of membrane potential	12/212	374/17913	1.72E-03	7.09E-03	3.67E-03	ABL1/ACTN2/PAF	12	GO
GO:0043627	response to estrogen	5/212	73/17913	1.73E-03	7.13E-03	3.69E-03	CCND1/EP300/GL	5	GO
GO:0014742	positive regulation of muscle hypertrophy	4/212	44/17913	1.79E-03	7.34E-03	3.80E-03	PARP1/IL6ST/PRK	4	GO
GO:0071827	plasma lipoprotein particle organization	4/212	44/17913	1.79E-03	7.34E-03	3.80E-03	APOB/APOE/PLA2	4	GO
GO:0030282	bone mineralization	6/212	108/17913	1.80E-03	7.38E-03	3.82E-03	PTK2B/FGR/NBR1	6	GO
GO:0071156	regulation of cell cycle arrest	6/212	108/17913	1.80E-03	7.38E-03	3.82E-03	CCND1/EP300/PC	6	GO
GO:1901990	regulation of mitotic cell cycle phase transition	13/212	428/17913	1.82E-03	7.45E-03	3.86E-03	APC/APEX1/CCND	13	GO
GO:0051258	protein polymerization	9/212	232/17913	1.82E-03	7.45E-03	3.86E-03	ABL1/RHOA/DCTI	9	GO
GO:0021675	nerve development	5/212	74/17913	1.84E-03	7.52E-03	3.90E-03	GLI3/KCNA2/PAX	5	GO
GO:0007093	mitotic cell cycle checkpoint	7/212	147/17913	1.85E-03	7.52E-03	3.90E-03	APC/CCND1/EP30	7	GO

GO:0008064	regulation of actin polymerization or depolymerization	7/212	147/17913	1.85E-03	7.52E-03	3.90E-03	ACTN2/RHOA/PT	7	GO
GO:0032373	positive regulation of sterol transport	3/212	21/17913	1.86E-03	7.52E-03	3.90E-03	APOE/LRP1/NR1	3	GO
GO:0032376	positive regulation of cholesterol transport	3/212	21/17913	1.86E-03	7.52E-03	3.90E-03	APOE/LRP1/NR1	3	GO
GO:0032462	regulation of protein homooligomerization	3/212	21/17913	1.86E-03	7.52E-03	3.90E-03	APOE/RHOC/SRC	3	GO
GO:0032928	regulation of superoxide anion generation	3/212	21/17913	1.86E-03	7.52E-03	3.90E-03	ITGB2/PRKCD/SY	3	GO
GO:0046827	positive regulation of protein export from nucleus	3/212	21/17913	1.86E-03	7.52E-03	3.90E-03	PPM1A/PRKACA/	3	GO
GO:0070920	regulation of production of small RNA involved in gene silencing by RNA	3/212	21/17913	1.86E-03	7.52E-03	3.90E-03	DDX5/MAP2K1/T	3	GO
GO:0071498	cellular response to fluid shear stress	3/212	21/17913	1.86E-03	7.52E-03	3.90E-03	PTK2B/PKD2/SRC	3	GO
GO:0008543	fibroblast growth factor receptor signaling pathway	6/212	109/17913	1.89E-03	7.62E-03	3.95E-03	CBL/CRKL/MAPK	6	GO
GO:0072009	nephron epithelium development	6/212	109/17913	1.89E-03	7.62E-03	3.95E-03	GLI3/SMAD4/MY	6	GO
GO:0016050	vesicle organization	10/212	280/17913	1.90E-03	7.69E-03	3.98E-03	CD59/DLG4/F8/S	10	GO
GO:0030832	regulation of actin filament length	7/212	148/17913	1.93E-03	7.78E-03	4.03E-03	ACTN2/RHOA/PT	7	GO
GO:0002279	mast cell activation involved in immune response	4/212	45/17913	1.94E-03	7.82E-03	4.05E-03	CBL/FGR/KIT/LYN	4	GO
GO:0002448	mast cell mediated immunity	4/212	45/17913	1.94E-03	7.82E-03	4.05E-03	CBL/FGR/KIT/LYN	4	GO
GO:0032570	response to progesterone	4/212	45/17913	1.94E-03	7.82E-03	4.05E-03	SRC/THBS1/NCO	4	GO
GO:0031100	animal organ regeneration	5/212	75/17913	1.95E-03	7.84E-03	4.06E-03	CCND1/GLI3/PCN	5	GO
GO:0048678	response to axon injury	5/212	75/17913	1.95E-03	7.84E-03	4.06E-03	JUN/LRP1/LYN/M	5	GO
GO:0072078	nephron tubule morphogenesis	5/212	75/17913	1.95E-03	7.84E-03	4.06E-03	GLI3/SMAD4/MY	5	GO
GO:0032434	regulation of proteasomal ubiquitin-dependent protein catabolic process	6/212	110/17913	1.98E-03	7.92E-03	4.10E-03	ARAF/DAB2/SMA	6	GO
GO:1901991	negative regulation of mitotic cell cycle phase transition	9/212	235/17913	1.99E-03	7.96E-03	4.12E-03	APC/CCND1/EP3	9	GO
GO:1902803	regulation of synaptic vesicle transport	5/212	76/17913	2.07E-03	8.28E-03	4.29E-03	PRKCB/PRKCG/R	5	GO
GO:0071453	cellular response to oxygen levels	9/212	237/17913	2.10E-03	8.39E-03	4.35E-03	CASR/CBL/EP300	9	GO
GO:0030514	negative regulation of BMP signaling pathway	4/212	46/17913	2.11E-03	8.39E-03	4.35E-03	ABL1/SMAD7/PP	4	GO
GO:0031638	zymogen activation	4/212	46/17913	2.11E-03	8.39E-03	4.35E-03	SERPINE1/PLAT/	4	GO
GO:0031648	protein destabilization	4/212	46/17913	2.11E-03	8.39E-03	4.35E-03	EP300/PLK1/PRN	4	GO
GO:0032369	negative regulation of lipid transport	4/212	46/17913	2.11E-03	8.39E-03	4.35E-03	ITGB3/PTPN11/T	4	GO
GO:0070231	T cell apoptotic process	4/212	46/17913	2.11E-03	8.39E-03	4.35E-03	GLI3/RAG1/TP53	4	GO
GO:0021987	cerebral cortex development	6/212	112/17913	2.16E-03	8.60E-03	4.45E-03	RHOA/CRKL/GLI3	6	GO
GO:0030279	negative regulation of ossification	5/212	77/17913	2.19E-03	8.68E-03	4.50E-03	PTK2B/NBR1/SM	5	GO
GO:0045685	regulation of glial cell differentiation	5/212	77/17913	2.19E-03	8.68E-03	4.50E-03	F2/HDAC1/IL6ST/	5	GO
GO:0072088	nephron epithelium morphogenesis	5/212	77/17913	2.19E-03	8.68E-03	4.50E-03	GLI3/SMAD4/MY	5	GO
GO:0090398	cellular senescence	5/212	77/17913	2.19E-03	8.68E-03	4.50E-03	ABL1/PRKCD/MA	5	GO
GO:0022411	cellular component disassembly	14/212	492/17913	2.24E-03	8.87E-03	4.60E-03	ACTN2/APC/CAS	14	GO
GO:0071482	cellular response to light stimulus	6/212	113/17913	2.26E-03	8.94E-03	4.63E-03	PARP1/EP300/M	6	GO
GO:0008542	visual learning	4/212	47/17913	2.28E-03	8.99E-03	4.66E-03	KIT/RAG1/PIAS1/	4	GO
GO:0014911	positive regulation of smooth muscle cell migration	4/212	47/17913	2.28E-03	8.99E-03	4.66E-03	LRP1/PAK1/PDG	4	GO
GO:0043124	negative regulation of I-kappaB kinase/NF-kappaB signaling	4/212	47/17913	2.28E-03	8.99E-03	4.66E-03	ABL1/RHOA/HDA	4	GO
GO:0051438	regulation of ubiquitin-protein transferase activity	4/212	47/17913	2.28E-03	8.99E-03	4.66E-03	ABL1/SMAD7/PL	4	GO
GO:0071824	protein-DNA complex subunit organization	9/212	240/17913	2.29E-03	9.00E-03	4.66E-03	PARP1/MYC/RAD	9	GO
GO:1902806	regulation of cell cycle G1/S phase transition	8/212	195/17913	2.29E-03	9.00E-03	4.66E-03	APEX1/CCND1/E	8	GO
GO:0008589	regulation of smoothened signaling pathway	5/212	78/17913	2.32E-03	9.10E-03	4.71E-03	GLI3/PRRX1/PRK	5	GO
GO:1905954	positive regulation of lipid localization	5/212	78/17913	2.32E-03	9.10E-03	4.71E-03	APOB/APOE/LRP	5	GO
GO:0044843	cell cycle G1/S phase transition	10/212	288/17913	2.34E-03	9.15E-03	4.74E-03	APEX1/CCND1/E	10	GO
GO:0030072	peptide hormone secretion	9/212	241/17913	2.36E-03	9.22E-03	4.78E-03	CASR/LRP1/SMA	9	GO
GO:0043281	regulation of cysteine-type endopeptidase activity involved in apoptotic process	8/212	196/17913	2.37E-03	9.22E-03	4.78E-03	RHOA/CD44/SM	8	GO
GO:0006304	DNA modification	6/212	114/17913	2.37E-03	9.22E-03	4.78E-03	PARP1/APEX1/M	6	GO
GO:0042752	regulation of circadian rhythm	6/212	114/17913	2.37E-03	9.22E-03	4.78E-03	KCNA2/PPARA/P	6	GO
GO:0046660	female sex differentiation	6/212	114/17913	2.37E-03	9.22E-03	4.78E-03	CASP3/KIT/SMAD	6	GO
GO:0031214	biomineral tissue development	7/212	154/17913	2.41E-03	9.39E-03	4.87E-03	PTK2B/FGR/NBR	7	GO

GO:0001945	lymph vessel development	3/212	23/17913	2.43E-03	9.39E-03	4.87E-03	EPHA2/SYK/NR2F	3	GO
GO:0010758	regulation of macrophage chemotaxis	3/212	23/17913	2.43E-03	9.39E-03	4.87E-03	PTK2B/MAPK3/TI	3	GO
GO:0051882	mitochondrial depolarization	3/212	23/17913	2.43E-03	9.39E-03	4.87E-03	ABL1/PARP1/SRC	3	GO
GO:0060055	angiogenesis involved in wound healing	3/212	23/17913	2.43E-03	9.39E-03	4.87E-03	ITGB3/SERPINE1/	3	GO
GO:0072215	regulation of metanephros development	3/212	23/17913	2.43E-03	9.39E-03	4.87E-03	MYC/PAX2/PDGF	3	GO
GO:1902003	regulation of amyloid-beta formation	3/212	23/17913	2.43E-03	9.39E-03	4.87E-03	APOE/CASP3/PRN	3	GO
GO:2000637	positive regulation of gene silencing by miRNA	3/212	23/17913	2.43E-03	9.39E-03	4.87E-03	DDX5/MAP2K1/T	3	GO
GO:2000726	negative regulation of cardiac muscle cell differentiation	3/212	23/17913	2.43E-03	9.39E-03	4.87E-03	SMAD4/PAK1/PP.	3	GO
GO:0000075	cell cycle checkpoint	8/212	197/17913	2.44E-03	9.42E-03	4.88E-03	APC/CCND1/EP30	8	GO
GO:0010833	telomere maintenance via telomere lengthening	5/212	79/17913	2.46E-03	9.43E-03	4.88E-03	PARP1/MAPK1/M	5	GO
GO:0032204	regulation of telomere maintenance	5/212	79/17913	2.46E-03	9.43E-03	4.88E-03	PARP1/MYC/MAF	5	GO
GO:0042509	regulation of tyrosine phosphorylation of STAT protein	5/212	79/17913	2.46E-03	9.43E-03	4.88E-03	IL6ST/KIT/LYN/OS	5	GO
GO:0043154	negative regulation of cysteine-type endopeptidase activity involved in apoptotic process	5/212	79/17913	2.46E-03	9.43E-03	4.88E-03	CD44/PAX2/RAG1	5	GO
GO:0061333	renal tubule morphogenesis	5/212	79/17913	2.46E-03	9.43E-03	4.88E-03	GLI3/SMAD4/MY(	5	GO
GO:0001818	negative regulation of cytokine production	10/212	290/17913	2.46E-03	9.43E-03	4.88E-03	EPHA2/F2/INPP5I	10	GO
GO:0002762	negative regulation of myeloid leukocyte differentiation	4/212	48/17913	2.47E-03	9.43E-03	4.88E-03	INPP5D/LYN/MY(	4	GO
GO:0010171	body morphogenesis	4/212	48/17913	2.47E-03	9.43E-03	4.88E-03	EP300/PDGFRA/F	4	GO
GO:0031103	axon regeneration	4/212	48/17913	2.47E-03	9.43E-03	4.88E-03	JUN/LRP1/MAP2I	4	GO
GO:0048546	digestive tract morphogenesis	4/212	48/17913	2.47E-03	9.43E-03	4.88E-03	GLI3/SMAD2/SM/	4	GO
GO:0071825	protein-lipid complex subunit organization	4/212	48/17913	2.47E-03	9.43E-03	4.88E-03	APOB/APOE/PLA2	4	GO
GO:0042471	ear morphogenesis	6/212	115/17913	2.47E-03	9.43E-03	4.88E-03	PAX2/PRRX1/MA	6	GO
GO:0043620	regulation of DNA-templated transcription in response to stress	6/212	115/17913	2.47E-03	9.43E-03	4.88E-03	EP300/JUN/PSMA	6	GO
GO:0021915	neural tube development	7/212	155/17913	2.50E-03	9.53E-03	4.94E-03	ABL1/EPHA2/GLI3	7	GO
GO:0031960	response to corticosteroid	7/212	155/17913	2.50E-03	9.53E-03	4.94E-03	PARP1/RHOA/BC	7	GO
GO:0010633	negative regulation of epithelial cell migration	6/212	116/17913	2.58E-03	9.81E-03	5.08E-03	APOE/RHOA/JUP/	6	GO
GO:0032273	positive regulation of protein polymerization	6/212	116/17913	2.58E-03	9.81E-03	5.08E-03	RHOA/DCTN1/PT	6	GO
GO:0001906	cell killing	7/212	156/17913	2.59E-03	9.84E-03	5.10E-03	CD59/F2/PTPN6/	7	GO
GO:0070304	positive regulation of stress-activated protein kinase signaling cascade	7/212	156/17913	2.59E-03	9.84E-03	5.10E-03	PTK2B/LYN/PAK1	7	GO
GO:0034764	positive regulation of transmembrane transport	8/212	199/17913	2.60E-03	9.84E-03	5.10E-03	ABL1/ACTN2/F2/	8	GO

Supplementary Table 9. Coefficients for all prediction models using the toxicity module.

Model coefficients	Maximal toxicity	Neutropenia	Leukopenia	Thrombocytopenia
(Intercept)	-10.4875342	-12.01963533	-2.508590599	-0.323189363
rs10898940	0.497946656	0.55195109	0.090056227	0.069250252
rs11629627	0.187098557	0.370523357	0.065096288	0.041418253
rs11629694	0.182897089	0.364594862	0.069821684	0.045260368
rs11794611	-0.183345821	-0.496607834	-0.06210544	-0.049861904
rs11996440	-0.250042367	-0.250393934	-0.050363849	-0.028587538
rs12034981	-0.287606968	-0.268034264	-0.050384676	-0.028587378
rs12046258	-0.824095051	-0.309870137	-0.038373666	-0.06282415
rs12117788	-0.892453588	-0.695199766	-0.080109069	-0.125644995
rs1247572	-1.281081728	-0.452196964	-0.074173234	-0.125640864
rs12981050	-0.223789244	0.016180507	-0.018274816	-0.078694735
rs13431476	-0.349797118	0.353866724	-0.022702559	-0.062714409
rs1367216	0.561322604	0.46149159	0.073192813	0.067967017
rs1498830	0.246854276	0.309196126	0.030136621	0.061410388
rs1652456	0.254474915	0.323808562	0.03735319	0.069053027
rs1740430	0.25496169	0.325358739	0.037352971	0.0690503
rs17404301	0.255595641	0.326844238	0.037352761	0.069047794
rs2465836	0.370742434	0.258490844	0.021081853	0.061407768
rs2569538	0.265999967	0.257349992	0.034689589	0.067674781
rs2569543	0.265593055	0.256278287	0.034689354	0.067672591
rs2590763	-1.158371309	-0.876418935	-0.056411876	-0.119210835
rs2590807	0.2579832	0.371930189	0.064941948	0.051974573
rs2590827	0.397774028	0.262369442	0.053678483	0.052484634
rs2590829	0.398275511	0.262195275	0.053678026	0.052482244
rs2616405	0.398900691	0.262240756	0.053677564	0.052480021
rs2616431	0.399303316	0.262433697	0.053677103	0.052477959
rs2616433	-0.232232756	-0.388160803	-0.022422458	-0.080886555
rs34285220	1.32941569	0.863159688	0.057540746	0.12517169
rs4864488	0.913501478	1.333991665	0.171027056	0.054145953
rs4864820	0.007836103	0.214937319	0.054695302	0.00654473
rs531650	0.867333871	0.913848959	0.109611374	0.007313413
rs5858236	0.868616326	0.912977532	0.109610769	0.007313781
rs5858241	0.870270853	0.912631962	0.109610223	0.007314117
rs58982282	-0.431942613	-0.159113741	-0.012668171	-0.058236367
rs59065201	0.080999027	-0.970944868	-0.043108517	-0.035923388
rs59481386	0.211350701	0.445594974	0.06360493	0.029834988
rs61825739	0.211378623	0.445398835	0.063604534	0.029833633
rs62225956	0.211650594	0.445675441	0.063604131	0.029832363
rs62225957	0.211925305	0.44593192	0.063603729	0.029831159
rs62225958	0.465695378	-0.238929746	0.082159532	0.068456301
rs62436699	-0.01589277	-1.004746862	-0.071438525	-0.08164559
rs66628040	-0.52764896	-0.712643953	-0.032358733	-0.084026152
rs67425308	-0.030931153	-0.68844837	-0.067672847	-0.046493077
rs6815433	0.598636455	0.406298085	0.090414168	0.067232386
rs6826915	0.59849942	0.405917604	0.090413768	0.067230446
rs6828755	-0.164030703	-0.533748646	-0.059957538	-0.041154058
rs6842780	-0.11788161	-0.341937487	-0.064294807	-0.052828328
rs6877573	0.367420051	0.007005274	0.067588765	0.050870926

rs7184277	0.896987063	-0.38973972	-0.085924231	0.020246592
rs7249753	1.453066675	0.997226712	0.134119299	0.1051765
rs7256793	0.636793827	0.620085165	0.078125715	0.087178651
rs72808733	0.266613733	0.449030187	0.07081102	0.059025779
rs73559035	0.567884676	1.255515983	0.155659201	0.095151419
rs7378471	0.568025535	1.255529112	0.155658571	0.095149521
rs74464209	0.568108799	1.255950539	0.155657962	0.095147737
rs7703879	-0.235410184	0.085274883	0.003796867	-0.0828088
rs77321629	-0.379354191	-0.263518776	-0.059143421	-0.114600925
rs77456162	-0.379285209	-0.263008857	-0.059142983	-0.11459542
rs783176	-0.379833438	-0.262685648	-0.059142552	-0.114590176
rs904414	-0.380379555	-0.262573743	-0.059142141	-0.114585238
rs9626751	-0.134605739	-0.24944032	-0.063269748	-0.115993071
rs9627100	-0.134555191	-0.249494629	-0.063269394	-0.115988646
rs9759545	-0.329501186	-0.162577883	-0.044399702	-0.142632739

Supplementary Table 10. Number of high, low and intermediate toxicity based on the Common Terminology Criteria for Adverse Events (CTCAE) for the maximal toxicity, neutropenia, leukopenia, and thrombocytopenia, respectively. The table also shows the number of samples that were used for training and testing the model.

	Maximal Toxicity			Nuetropenia			Leukopenia			Thrombocytopenia		
	**Total**	**Training**	**Test**	**Total**	**Training**	**Test**	**Total**	**Training**	**Test**	**Total**	**Training**	**Test**
**High toxicity (CTCAE 3-4)**	54	43	11	46	37	9	28	22	6	43	34	9
**Low toxicity (CTCAE 0-1)**	34	27	7	44	35	9	46	37	9	39	31	8
**Intermediate toxicity (CTCAE 2)**	8	-	-	6	-	-	22	-	-	14	-	-
