# Supplementary material for: Whole-genome sequencing and gene network modules predict gemcitabine/carboplatin-induced myelosuppression in non-small cell lung cancer patients
Source: NPJ Syst Biol Appl. 2020 Aug 24;6:25. doi: 10.1038/s41540-020-00146-6 (PMC7445166; doi:10.1038/s41540-020-00146-6)
Supplement: Supplementary file 2 — Reporting summary [file 41540_2020_146_MOESM2_ESM.pdf]

nature research

Corresponding author(s): Niclas Björn

Last updated by author(s): Jul 3, 2020

Reporting Summary

Nature Research wishes to improve the reproducibility of the work that we publish. This form provides structure for consistency and transparency in reporting. For further information on Nature Research policies, see Authors & Referees and the Editorial Policy Checklist.

Statistics

For all statistical analyses, confirm that the following items are present in the figure legend, table legend, main text, or Methods section.

n/a	Confirmed	
☐	☒	The exact sample size (*n*) for each experimental group/condition, given as a discrete number and unit of measurement
☐	☒	A statement on whether measurements were taken from distinct samples or whether the same sample was measured repeatedly
☐	☒	The statistical test(s) used AND whether they are one- or two-sided<br>*Only common tests should be described solely by name; describe more complex techniques in the Methods section.*
☒	☐	A description of all covariates tested
☒	☐	A description of any assumptions or corrections, such as tests of normality and adjustment for multiple comparisons
☐	☒	A full description of the statistical parameters including central tendency (e.g. means) or other basic estimates (e.g. regression coefficient) AND variation (e.g. standard deviation) or associated estimates of uncertainty (e.g. confidence intervals)
☐	☒	For null hypothesis testing, the test statistic (e.g. *F*, *t*, *r*) with confidence intervals, effect sizes, degrees of freedom and *P* value noted<br>*Give P values as exact values whenever suitable.*
☒	☐	For Bayesian analysis, information on the choice of priors and Markov chain Monte Carlo settings
☒	☐	For hierarchical and complex designs, identification of the appropriate level for tests and full reporting of outcomes
☒	☐	Estimates of effect sizes (e.g. Cohen's *d*, Pearson's *r*), indicating how they were calculated

Our web collection on statistics for biologists contains articles on many of the points above.

Software and code

Policy information about availability of computer code

Data collection	We utilized freely available open sourse data for the bone marrow enrichment analysis accessed under the accession number GSE59894 in the NCBI Gene Expression Omnibus database (https://www.ncbi.nlm.nih.gov/geo/).
Data analysis	We utilized freely available open source functions and programs all referred to along with the specific version numbers indicated in the Methods section

For manuscripts utilizing custom algorithms or software that are central to the research but not yet described in published literature, software must be made available to editors/reviewers. We strongly encourage code deposition in a community repository (e.g. GitHub). See the Nature Research guidelines for submitting code & software for further information.

Data

Policy information about availability of data

All manuscripts must include a data availability statement. This statement should provide the following information, where applicable:

- Accession codes, unique identifiers, or web links for publicly available datasets
- A list of figures that have associated raw data
- A description of any restrictions on data availability

The datasets generated during and/or analyzed during the current study are not publicly available due to that this is not permitted by the ethical approval of the study but are available from the corresponding author (NB, niclas.bjorn@liu.se) on reasonable request together with the appropriate ethical approval.

Field-specific reporting

Please select the one below that is the best fit for your research. If you are not sure, read the appropriate sections before making your selection.

☒ Life sciences ☐ Behavioural & social sciences ☐ Ecological, evolutionary & environmental sciences

For a reference copy of the document with all sections, see nature.com/documents/nr-reporting-summary-flat.pdf

Life sciences study design

All studies must disclose on these points even when the disclosure is negative.

Sample size	As the focus of this explorative study was to establish models for predicting toxicity by combining several genetic variants the determination of a pre-specified effect size for single genetic variants was not a major concern during the planning of the study. However, a sample size of >90 was chosen to be able to detect the effect even of the most common genetic variants in the whole genome.
Data exclusions	No data was excluded.
Replication	96 samples were included in the anlysis. For the prediction models we utilized a cross-vallidation approach and withheld 20% of the data for testing the models. All RNA-seq experiments were run in duplicates.
Randomization	Splitting the samples into 80% and 20% for training and testing purposes was randomized.
Blinding	Not applicable.

Reporting for specific materials, systems and methods

We require information from authors about some types of materials, experimental systems and methods used in many studies. Here, indicate whether each material, system or method listed is relevant to your study. If you are not sure if a list item applies to your research, read the appropriate section before selecting a response.

Materials & experimental systems

n/a	Involved in the study	
☒	☐	Antibodies
☐	☒	Eukaryotic cell lines
☒	☐	Palaeontology
☒	☐	Animals and other organisms
☐	☒	Human research participants
☒	☐	Clinical data

Methods

n/a	Involved in the study	
☒	☐	ChIP-seq
☒	☐	Flow cytometry
☒	☐	MRI-based neuroimaging

Eukaryotic cell lines

Policy information about cell lines

Cell line source(s)	CMK (ACC-392) and MOLM-1 (ACC-720) from the Leibniz-Institute DSMZ - German Collection of Microorganisms and Cell Cultures, and K562 (CCL-243) from the American Type Culture Collection were used.
Authentication	None of the cell lines were authenticated. However, their passage numbers were kept below 15 passages from their acquisition.
Mycoplasma contamination	We confirm that all cell lines tested negative for mycoplasma contamination.
Commonly misidentified lines (See ICLAC register)	*Name any commonly misidentified cell lines used in the study and provide a rationale for their use.*

Human research participants

Policy information about studies involving human research participants

Population characteristics	All relevant patient characteristics are listed in Table 1
Recruitment	Patients diagnosed with NSCLC between 2006 and 2008 at Karolinska University Hospital, Stockholm, Sweden, were continuously recruited for the study and included after providing written informed consent, in accordance with the Helsinki Declaration.

Ethics oversight	The study received ethical approval from the regional ethics committee in Stockholm (DNR-03-413 with amendment 2016/258-32/1).

Note that full information on the approval of the study protocol must also be provided in the manuscript.
